# Catalytic asymmetric synthesis of a nitrogen heterocycle through stereocontrolled direct photoreaction from electronically excited state

**DOI:** 10.1038/s41467-017-02148-1

**Published:** 2017-12-21

**Authors:** Xiaoqiang Huang, Xinyao Li, Xiulan Xie, Klaus Harms, Radostan Riedel, Eric Meggers

**Affiliations:** 1 Fachbereich Chemie, Philipps-Universität Marburg, Hans-Meerwein-Straße 4, 35043 Marburg, Germany; 2 State Key Laboratory of Natural and Biomimetic Drugs, Peking University, Xue Yuan Road 38, Beijing, 100191 China

## Abstract

The reactivity of photoexcited molecules has been extensively studied for decades but until today direct bond-forming reactions of such excited states in a catalytic and asymmetric fashion are restricted to the synthesis of cyclobutanes via [2 + 2] photocycloadditions. Herein, we demonstrate a previously elusive visible-light-induced catalytic asymmetric [2 + 3] photocycloaddition of alkenes with vinyl azides. A wide range of complex 1-pyrrolines are obtained as single diastereoisomers and with up to >99% enantiomeric excess using a simple reaction setup and mild reaction conditions. The reaction is proposed to proceed through the photoexcitation of a complex out of chiral rhodium catalyst coordinated to α,β-unsaturated *N*-acylpyrazole substrates. All reactive intermediates remain bound to the catalysts thereby providing a robust catalytic scheme (no exclusion of air necessary) with excellent stereocontrol. This work expands the scope of stereocontrolled bond-forming reactions of photoexcited intermediates by providing catalytic asymmetric access to a key nitrogen heterocycle in organic chemistry.

## Abstract (web summary)

Despite intensive research on photoexcited molecules, stereocontrol of direct bond formation upon photoexcitation remains limited. Here the authors expand the research on stereocontrolled bond forming photochemistry and introduce the catalytic asymmetric synthesis of chiral nitrogen heterocycles.

## Introduction

**Fig. 1:**
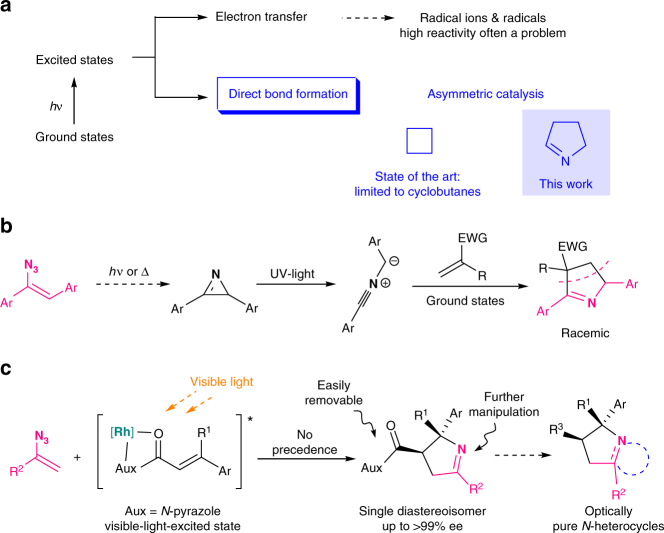
Different reactivity of photochemically excited states. **a** Charge transfer versus direct bond-forming reactions from a photoexcited state, whereas direct stereocontrolled reactions of excited states limited to [2 + 2] photocycloadditions. **b** Previous report on the photochemical construction of 1-pyrrolines via ground state 1,3-dipolar cycloaddition. **c** This work: visible-light-activated catalytic asymmetric [2 + 3] photocycloaddition via the direct reaction of a photochemically excited intermediate en route to enantioenriched 1-pyrrolines and functionalized pyrrolidines

The induction or activation of chemical reactions by visible light is an extremely valuable tool for synthetic organic chemistry^1,2^. Typically, photochemically excited species engage in single electron transfer with a substrate or reagent to generate radical ions and/or free radicals, which then participate in a multitude of reaction schemes (Fig. 1a) ^3^. However, reactions through radical ions and free radicals have severe drawbacks. For example, radical ions are extremely sensitive to solvent effects, placing restriction to the reaction parameters, whereas the high reactivity of free radicals renders a control of their reaction pathway challenging, especially the stereocontrol, and often leads to a narrow substrate scope^4,5^. A highly attractive alternative are therefore bond-forming reactions that occur directly from a photoexcited state without prior charge transfer to circumvent the mentioned disadvantages arising from intermediate free radicals and radical ions^6^. Due to the demand of optically pure compounds in the chemical and pharmaceutical industry, catalytic stereocontrolled reactions are of particular interest. Unfortunately, currently available methods for catalytic asymmetric reactions that occur from a photochemically excited state without any intermediate charge separation are limited to [2 + 2] photocycloadditions^7–12^, probably owing to the lack of suitable, robust catalysts to enable new reaction patterns of such short-lived excited states and at the same time to control their stereochemistry. Although cyclobutanes built through [2 + 2] photocycloadditions are present in natural products, a direct access to more prevalent structural motifs, such as the important class of nitrogen hetereocycles, by direct stereocontrolled reactions from photoexcited states would be highly desirable and furnish new powerful synthetic methodology.

1-Pyrrolines are widely found in biologically active molecules such as natural products and drugs, and constitute versatile synthetic intermediates by conversion to functionalized pyrrolidines^13,14^. Catalytic asymmetric methods to directly access this important heterocycle are little developed and limited to a few reports including the partial hydrogenation of pyrroles^15^, 1,3-cycloadditions of azlactones^16,17^ or α-isocyano esters^18^ with acceptor-substituted alkenes, and some other addition/cyclization protocols^19,20^. We envisioned to access this heterocycle in a completely different way by exploiting the photochemical reactivity of vinyl azides as shown in Fig. 1. Padwa reported in pioneering work^21,22^ that the UV-photolysis of aryl azirines, accessible from vinyl azides by photolysis or heat^23,24^, could form nitrile ylides which were subsequently trapped by ground-state electron-deficient alkenes to afford racemic 1-pyrrolines (Fig. 1b). Inspired by Padwa’s work and other reports on the diverse reactivity of vinyl azides^25^, we develop a herein reported direct [2 + 3] photocycloaddition of photoexcited acceptor-substituted alkenes with vinyl azides in a catalytic and asymmetric manner toward the synthesis of nonracemic chiral 1-pyrrolines with virtually perfect diastereoselectivities and enantioselectivities of up > 99% ee. Although vinyl azides are well-known to act as radical acceptors^26–28^ or triplet energy acceptors^23,29^, their ability to direct undergo cycloaddition with photoexcited alkenes has no precedence. All mechanistic experiments support a scenario in which a visible-light-excited complex out of α,β-unsaturated carbonyl compound and rhodium catalyst directly engages in a reaction with vinyl azides, thereby providing a convenient and unique access to nonracemic chiral 1-pyrrolines with a constitution that differs from related ground-state chemistry.

## Results

### Optimization of reaction conditions

Recently, we introduced a visible-light-activated intermolecular [2 + 2] photocycloaddition of photoexcited substrate-bound rhodium-based Lewis acid complexes with alkenes providing a practical synthetic route to enantioenriched cyclobutanes^12^. However, our previous work and related reports from other groups^8–11^ did not address the prevalent restriction to cyclobutanes as structural motifs for such stereocontrolled bond-forming reactions out of a photoexcited state. To explore new reaction manifolds of photoexcited molecules, we tested the reaction of *N*-acyl-3,5-dimethylpyrazole **1a** with vinyl azide **2a** catalyzed by a single-chiral bis-cyclometalated rhodium complex (Λ–**RhS**)^30^ developed in our group (Table 1). Gratifyingly, the expected 1-pyrroline **3a** formed as a single diastereoisomer, albeit in only 54% yield with an unsatisfactory enantioselectivity of 86% ee and conditions of high catalyst loading (8.0 mol%) (Table 1, entry 1). Subsequently, we found that substituents within the pyrazole auxiliary have a profound influence on the reaction performance (entries 1–3). With a single phenyl substituent at the 3-position (R^1^) of the pyrazole, the yield of 1-pyrroline **3c** was increased to 83% with 87% ee (entry 3). Optimization of solvent and concentration led to an improved outcome of **3c** with 98% yield and 95% ee in CDCl_3_ under more diluted conditions (entries 3–6). We continued to evaluate the effect of the auxiliary in order to reduce the catalyst loading (entries 7–10, for more details see Supplementary Tables 1–3). As a result, 3-(4-methoxyphenyl) pyrazole provide the best choice for the present [2 + 3] photocycloaddition, allowing to reduce the catalyst loading to 4 mol% (entry 10). No differences were found between CDCl_3_, which was used to conveniently determine NMR yields, and CHCl_3_ (entry 11). Importantly, this simple and mild protocol can be conducted under air without the need for inert conditions, which renders it very practical (entry 12). It is noteworthy that the iridium analog Λ–**IrS** ^31^ also provided the 1-pyrroline **3f** (50% yield) albeit without any enantioselectivity (entry 13). Control experiments demonstrated that the current transformation relies both on rhodium catalyst (entry 14) and visible light (entry 15).

**Table 1:** Conditions optimization for the direct asymmetric visible-light-excited [2 + 3] photocycloaddition^a^


Entry	Catalyst^b^	1	Solvent	Yield (%)^c^	ee (%)^d^
1	Λ–**RhS** (8.0)	**1a** (R^1^ = R^2^ = Me)	acetone (0.2 M)	54 (**3a**)	86
2	Λ–**RhS** (8.0)	**1b** (R^1^ = R^2^ = H)	acetone (0.2 M)	75 (**3b**)	72
3	Λ–**RhS** (8.0)	**1c** (R^1^ = C_6_H_5_; R^2^ = H)	acetone (0.2 M)	83 (**3c**)	87
4	Λ–**RhS** (8.0)	**1c**	CH_2_Cl_2_ (0.2 M)	55 (**3c**)	84
5	Λ–**RhS** (8.0)	**1c**	CDCl_3_ (0.2 M)	93 (**3c**)	92
6	Λ–**RhS** (8.0)	**1c**	CDCl_3_ (0.1 M)	98 (**3c**)	95
7	Λ–**RhS** (4.0)	**1c**	CDCl_3_ (0.1 M)	80 (**3c**)	92
8	Λ–**RhS** (4.0)	**1d** (R^1^ = Me; R^2^ = H)	CDCl_3_ (0.1 M)	82 (**3d**)	92
9	Λ–**RhS** (4.0)	**1e** (R^1^ = 4-FC_6_H_4_; R^2^ = H)	CDCl_3_ (0.1 M)	80 (**3e**)	92
10	Λ–**RhS** (4.0)	**1f** (R^1^ = PMP; R^2^ = H)	CDCl_3_ (0.1 M)	92 (90)^e^ (**3f**)	94
11	Λ–**RhS** (4.0)	**1f**	CHCl_3_ (0.1 M)	90 (**3f**)	94
12^f^	Λ–**RhS** (4.0)	**1f**	CDCl_3_ (0.1 M)	90 (**3f**)	94
13^f^	Λ–**IrS** (4.0)	**1f**	CDCl_3_ (0.1 M)	50 (**3f**)	0
14^f^	None	**1f**	CDCl_3_ (0.1 M)	7 (**3f**)	n.a.
15^f,g^	Λ–**RhS** (4.0)	**1f**	CDCl_3_ (0.1 M)	0 (**3f**)	n.a.

*PMP* 4-methoxyphenyl. *n.a*. not applicable

^a^Reaction conditions: *N*-Acylpyrazole **1** (0.10 mmol), vinyl azide **2a** (0.125 mmol) and the shown amount of catalyst in solvent were stirred at room temperature under an atmosphere of nitrogen with irradiation of blue LEDs (24 W)

^b^Catalyst loadings in mol% provided in brackets

^c^NMR yields

^d^Only one diastereoisomer observed judged by NMR and enantiomeric excess determined by HPLC analysis on chiral stationary phase

^e^Isolated yield provided in parenthesis

^f^Assembled in air, then sealed the tube

^g^Under dark

### Mechanistic investigation

**Fig. 2:**
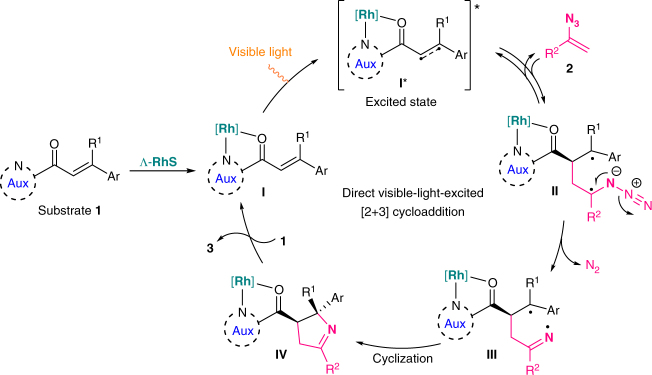
Proposed catalytic cycle. Direct visible-light-excited [2 + 3] photocycloaddition enabled by a single catalyst

The proposed mechanism for this [2 + 3] photocycloaddition is shown in Fig. 2. The coordination of substrate **1** to the chiral rhodium catalyst generates the substrate/catalyst complex **I**, which upon visible-light excitation generates the excited state I*, most likely in its triplet excited state. The following direct reaction of the excited state I* with the vinyl azide cosubstrate **2** affords the catalyst-bound diradical intermediate **II** under generation of a new C–C bond, with the stereochemistry of this bond formation being controlled by the metal-centred chirality of the rhodium complex. Subsequent extrusion of dinitrogen delivers the catalyst-bound iminyl radical intermediate **III** ^32^, which then undergoes a stereoselective cyclization generating the product coordinated complex **IV**. Release of product from the rhodium catalyst and coordination of new substrate **1** then initiates a new catalytic cycle. Since the reaction is insensitive to air, it can be assumed that the diradical intermediates **II** and **III** are very short-lived and thus cannot engage in any side reactions, thus revealing the advantages of such direct bond-forming reactions from a photoexcited state over processes that occur through initial electron transfer.

**Fig. 3:**
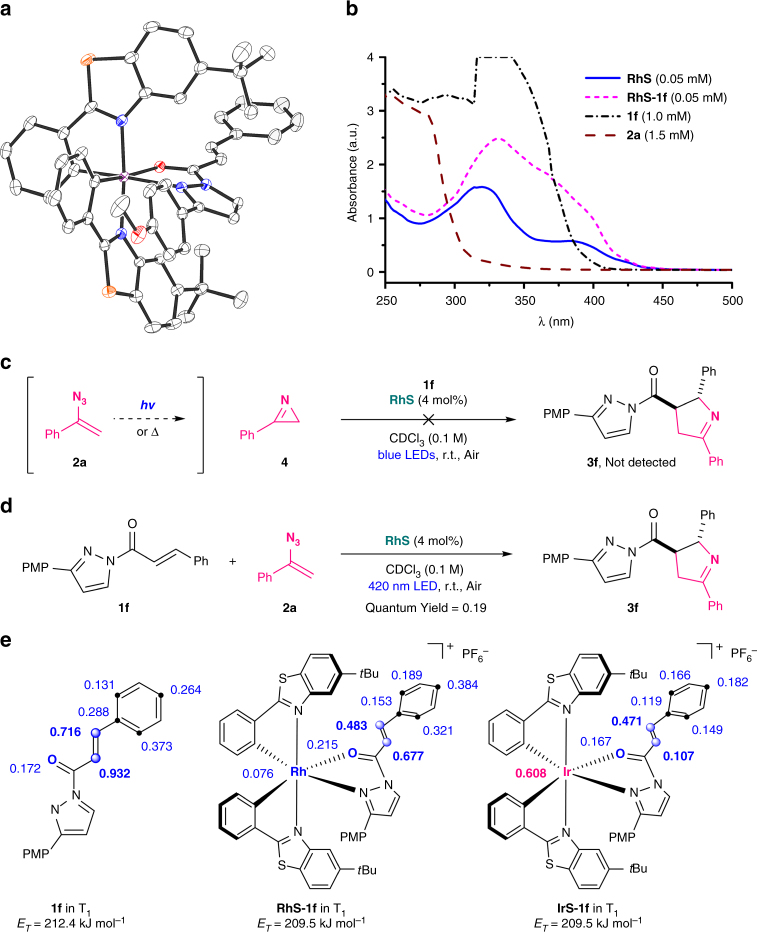
Mechanistic studies. **a** Crystal structure of the key intermediate **RhS**–**1f**. **b** UV/Vis absorption spectra of **RhS** (0.05 mM), **RhS–1f** (0.05 mM), **1f** (1.0 mM), and **2a** (1.5 mM) in CH_2_Cl_2_. **c** Control experiment which rules out 2*H*-azirines as reaction intermediates. **d** Determination of quantum yield of 0.19. **e** Calculated spin distribution and energy of the excited triplet states of **1f**, **RhS**–**1f**, and **IrS**–**1f**

The proposed mechanism is supported by a series of experiments. First, a crystal structure of the pyrazole substrate **1f** bound to the (racemic) catalyst **RhS** shown in Fig. 3a (**RhS**–**1f**) confirms the proposed formation of intermediate **I** of the catalytic cycle. Interestingly, it further reveals a *π*–*π* stacking between the cyclometalating benzothiazole moiety of the rhodium catalyst and the *p*-methoxyphenyl moiety of the pyrazole auxiliary, which might explain the superiority of this auxiliary by modulating binding constant, steric and/or electronic effects^33^. Second, UV-Vis absorption spectra demonstrate that the visible-light absorption of substrate **1f** is greatly enhanced upon binding the rhodium catalyst (**RhS**–**1f**) so that in the reaction mixture the intermediate **I** is selectively photoexcited to generate the photoexcited intermediate I* from which the reaction (I* + **2** → **II**) occurs (Fig. 3b). This is crucial in order to suppress any background reaction resulting from the photoactivation of the free α,β-unsaturated *N*-acylpyrazole substrate, which would deteriorate the overall enantioselectivity. On the other hand, the excited intermediate I* apparently undergoes a direct cycloaddition instead of triplet energy transfer to the vinyl azide cosubstrate **2**, so that no undesired photoinduced rearrangement^23^ to 2*H-*azirine **4** can occur. In fact, we ruled out that such azirines are viable intermediates in this catalytic cycle since independently synthesized 2*H*-azirine **4** was demonstrated to not react with **1f** in the presence of **RhS** and visible light (Fig. 3c). Furthermore, the quantum yield for the reaction **1f** + **2a** → **3f** was determined as Φ = 0.19, supporting our proposal that the photoreaction does not involve a chain process and instead a single photon is required for each formed product molecule (Fig. 3d). Finally, the problem remained why the iridium congener **IrS** cannot provide any enantioselectivity (Table 1, entry 13). Computational studies revealed that there is a significant difference between the spin distribution of excited **RhS**–**1f** and **IrS**–**1f**. As shown in Fig. 3e, most of the spin density in the triplet state of **RhS**–**1f** is localized at the alkene carbons of **1f**, at which position the chemoselective cycloaddition occurs, whereas in the triplet state of **IrS**–**1f**, the iridium center possesses the majority of the spin density. This distinct difference in the nature of the excited state might account for the reactivity differences. Photoexcited **IrS**–**1f** is not capable of undergoing a direct reaction with vinyl azides, but instead serves as a photosensitizer to transfer its triplet energy to free substrate **1**, and thereby serving as a photosensitizer for the formation of racemic [2 + 3] photocycloaddition product.

### Scope of the methodology

**Fig. 4:**
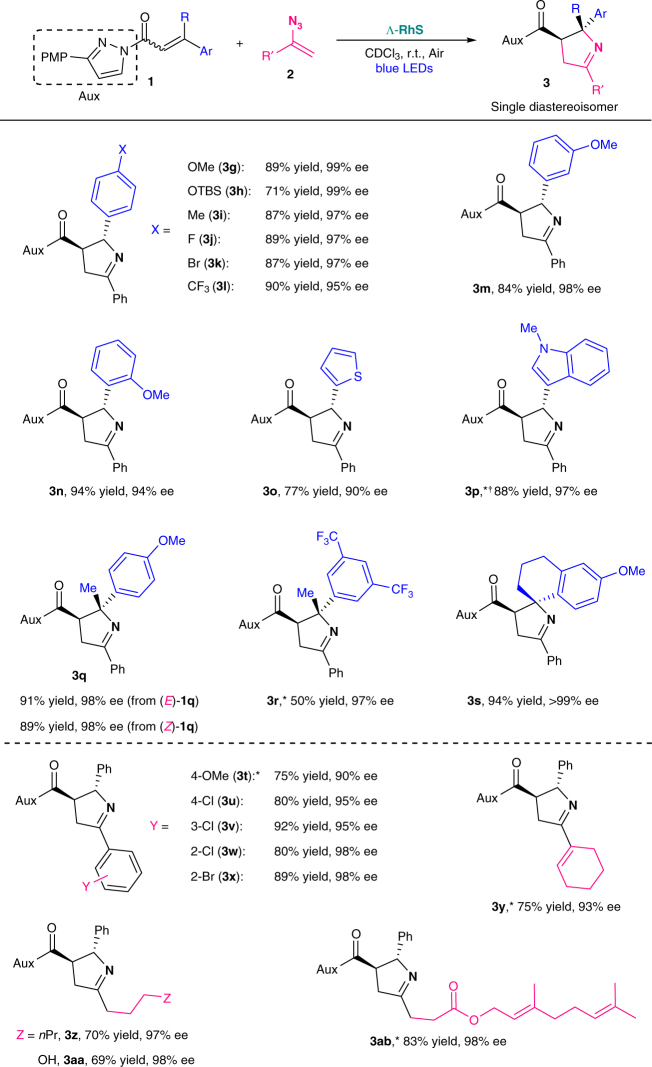
Substrate scope. Reaction conditions: **1** (0.10 mmol), **2** (0.125 mmol) and Λ–**RhS** (4.0 mol%) in CDCl_3_ (1.0 mL) were assembled under air and stirred at room temperature under irradiation with blue LEDs (24 W). ^*^Performed with 8.0 mol% of Λ–**RhS** under nitrogen atmosphere. ^†^Performed in CDCl_3_ (2.0 mL). Configurations were assigned with crystal structure of **3k**

With the mechanistic picture in mind, we next evaluated the generality of the [2 + 3] photocycloaddition with a single chiral rhodium catalyst using 3-(4-methoxyphenyl)pyrazole as the auxiliary. As shown in Fig. 4, different patterns of substitution at the β-aryl moiety of *N*-acylpyrazoles **1** were well tolerated, regardless of electronic nature (**3g**–**l**) or position (**3g**, **m**, **n**) of the substituents. Heteroaryl frameworks were proved to be compatible as demonstrated by the effective formation of thienyl- and indolyl- substituted 1-pyrrolines (**3o**, **p**). Notably, this protocol is amenable to the construction of complex 1-pyrrolines bearing a quaternary stereocenter (**3q**–**s**), including a spiro center (**3s**), reflecting the robustness of this single rhodium catalysis. Additionally, α,β-unsaturated *N*-acylpyrazoles (*E*)-**1q** and (*Z*)-**1q** gave the product **3q** in similar yields with identical stereochemistry as an indicator for the involvement of the diradical intermediates **II**/**III**. To our delight, various functionalized vinyl azides were readily accommodated, including not only aryl vinyl azides (**3t**–**x**) but also more challenging alkyl vinyl azides (**3z**–**ab**). Although vinyl azides bearing an electron-rich substitution at phenyl group gave a decreased ee value (**3t**, 90% ee), the reaction outcome was not influenced by steric hindrance (**3v**–**x**). Furthermore, a free hydroxyl group (**3aa**) as well as C = C double bonds (**3ab**) were tolerated, thereby highlighting the good functional group compatibility of the current protocol.

### Synthetic applications

**Fig. 5:**
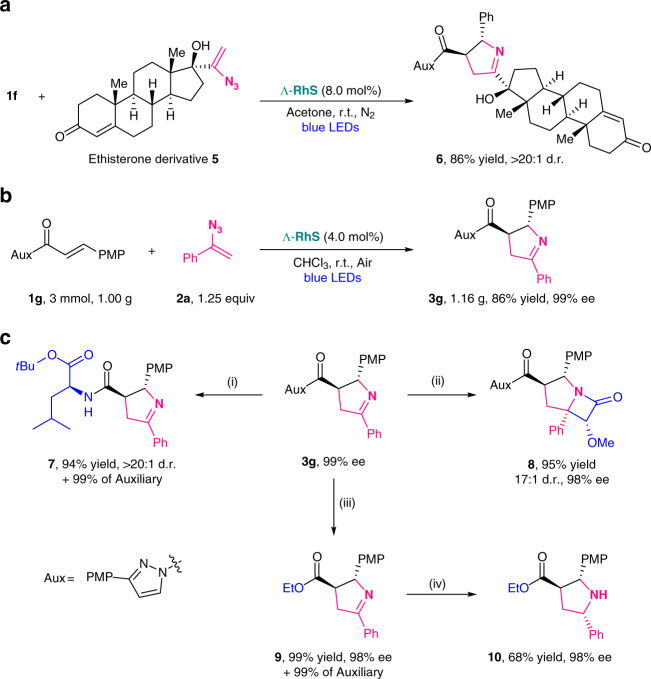
Synthetic applications. **a** Late-stage modification of ethisterone derivative. **b** Gram scale synthesis. **c** Functional group conversions. Reaction conditions are as follows: (i) *L*-Leucin-*tert*-butylester hydrochloride, Et_3_N, HOBt, toluene, 50 °C; (ii) 2-Methoxyacetyl chloride, Et_3_N, CH_2_Cl_2_, 50 °C; (iii) LiCl, Et_3_N, EtOH/THF (4:1), r.t.; (iv) Pd/C, H_2_, EtOAc, r.t.

With the practical conditions and broad substrate scope in hand, we next evaluated the synthetic potential of this protocol (Fig. 5). We were delighted to find a highly complex steroid substituted 1-pyrroline **6** could be obtained by the [2 + 3] photocycloaddition with ethisterone derived vinyl azide **5** in 86% yield with almost a single stereoisomer (Fig. 5a). Besides, a gram scale synthesis without loss of efficiency under air conditions highlights the practicality of this developed transformation without the requirement of inert conditions (Fig. 5b). Although the present methodology relies on a bidentate coordination mode, *N*-acylpyrazoles constitute very desirable synthons which can easily be transformed into other functionalities as shown in Fig. 5c for a clean and mild conversion to the amide **7** and ester **9**. Importantly, the auxiliary 3-(4-methoxyphenyl)pyrazole was fully recovered in these conversions. Furthermore, 1-pyrrolines with a prochiral cyclic imine group is applicable for further stereoselective synthetic manipulation as highlighted by the synthesis of a carbapenem analog **8** and pyrrolidine **10**.

## Discussion

In summary, we here introduced an important expansion of catalytic asymmetric reactions occurring via direct stereocontrolled bond-forming reactions of photoexcited states (Fig. 1c). Previously restricted to the class of cyclobutanes, we here reveal an economical^34^ access to the highly relevant class of chiral 1-pyrrolines based on a visible-light-activated [2 + 3] photocycloaddition of alkenes with vinyl azides catalyzed by a chiral rhodium complex. The employed robust rhodium-based chiral Lewis acid catalyst is key to the successful implementation of this methodology. Upon binding to the alkene substrate, it provides a unique handle for selective visible-light excitation of the substrate/catalyst complex with the triplet spin being mainly localized at the alkene carbons so that an efficient reaction with the vinyl azide cosubstrate can occur, while the metal-centered chirality provides outstanding stereocontrol. In addition to the conceptual appeal, the synthetic usefulness of this protocol is indicated by the simple reaction setup with a single catalyst and no necessity for inert reaction conditions, exceptional stereoselectivity with virtual perfect diastereoselectivity and up to >99% ee, a broad substrate scope that gives access to a large variety of chiral 1-pyrrolines, including quaternary stereocenters, and the ability to use these 1-pyrrolines as building blocks for conversions into biologically relevant chiral pyrrolidines. We anticipate that this report will stimulate the further discovery of new types of stereocontrolled reactions with electronically excited molecules.

## Methods

### General

For the emission spectrum of the blue LEDs lamp and the exemplary reaction setup, see Supplementary Figs 1 and 2. For more information about conditions screening and substrate scope, see Supplementary Tables 1–4. For the product analysis with HPLC on chiral stationary phase, see Supplementary Figs 8–38. For ^1^H and ^13^C NMR spectra of the compounds in this article, see Supplementary Figs 39–146, and more experimental details and compound characterization data, see Supplementary Methods. For details of the computational study see, Supplementary Fig. 5, Supplementary Table 7, Supplementary Methods and Supplementary Data. For crystallographic information, see the Supplementary Figs 3 and 4; Supplementary Tables 5 and 6; and Supplementary Methods. For configuration assignment of compounds **8** and **10**, see Supplementary Figs 6 and 7, Supplementary Table 8 and Supplementary Methods.

### General procedure for the reaction

Exemplary, to an oven-dried 10 mL Schlenk tube with a stirring bar, α,β-unsaturated *N*-acylpyrazole **1g** (33.4 mg, 0.10 mmol), Λ–**RhS** (3.5 mg, 4 mol%), CDCl_3_ (1.0 mL) and vinyl azide **2a** (18.2 mg, 0.125 mmol) were added in sequence under open air conditions. Then, the tube was sealed and positioned at ~8 cm from a 24 W blue LEDs lamp. After stirred at room temperature for 24 h (monitored by TLC), the mixture was directly subjected to ^1^H NMR to determine the diastereoselectivity of **3g** as only a single diastereoisomer formed. Then, all the mixture was collected and purified by flash chromatography on silica gel (*n*-hexane/EtOAc) to afford the product **3g** as a white solid (40.2 mg, 89% yield). The enantiomeric excess of **3g** was determined as 99% ee by HPLC using a Chiralpak OD-H column (mobile phase = *n*-hexane/isopropanol 95:5).

### Data availability

The X-ray crystallographic coordinates for the structure of **RhS**–**1f** and **3k** reported in this article have been deposited at the Cambridge Crystallographic Data Centre (CCDC) under deposition numbers CCDC 1568748 and 1568749, respectively. The data can be obtained free of charge from The Cambridge Crystallographic Data Centre via http://www.ccdc.cam.ac.uk/data_request/cif. The authors declare that the data supporting the findings of this study are available within the article and its Supplementary Information Files. All other data are available from the authors upon reasonable request.

## Electronic supplementary material

Supplementary Information

Peer Review File

Description of Additional Supplementary Files

Supplementary Data 1
