# Supplementary material for: Catalytic asymmetric synthesis of a nitrogen heterocycle through stereocontrolled direct photoreaction from electronically excited state
Source: Nat Commun. 2017 Dec 21;8:2245. doi: 10.1038/s41467-017-02148-1 (PMC5740077; doi:10.1038/s41467-017-02148-1)
Supplement: Supplementary file 1 — Supplementary Information [file 41467_2017_2148_MOESM1_ESM.pdf]

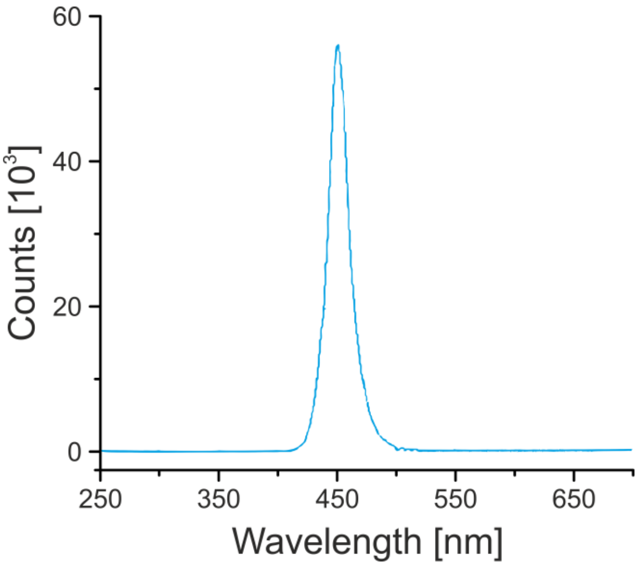

Supplementary Figure 1. Emission spectrum of the 24 W blue LEDs lamp (Hongchangzhaoming from Chinese Taobao, https://hongchang-led.taobao.com).

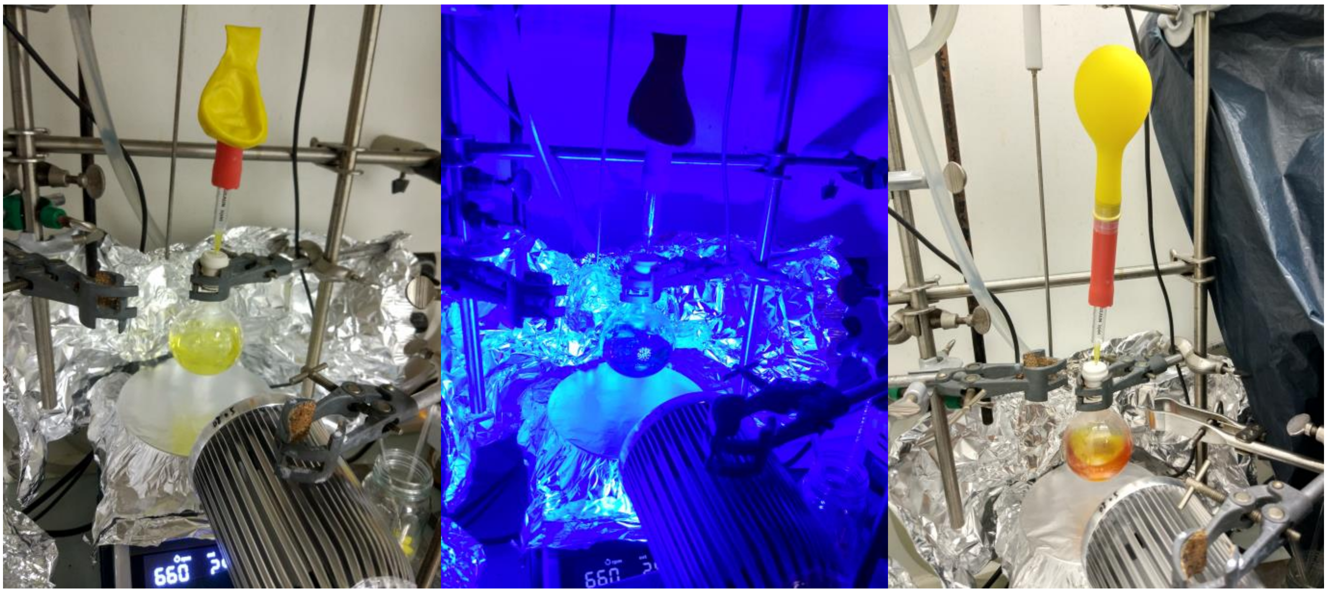

Before irradiation **Under irradiation** **After irradiation**

Supplementary Figure 2. Reaction setup for the large scale reaction.

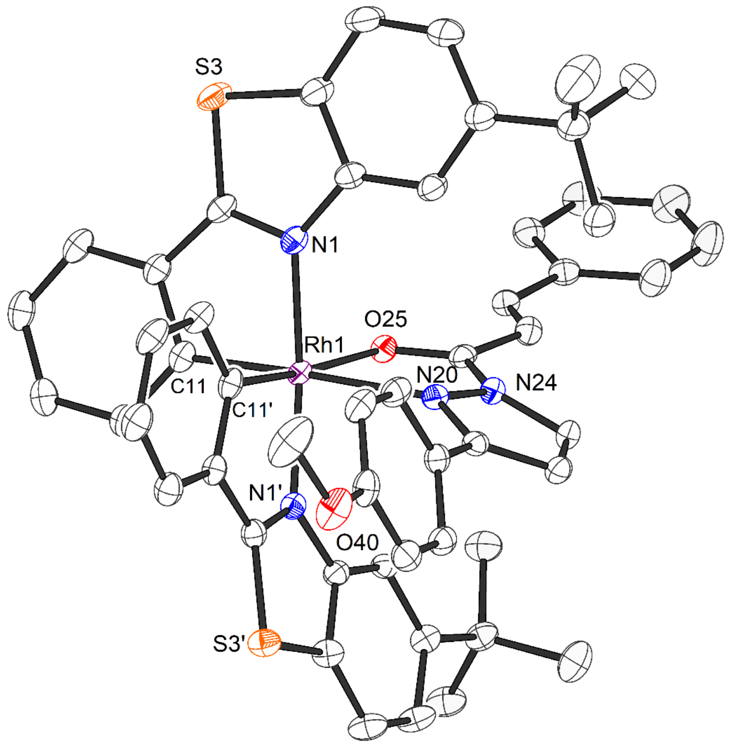

Supplementary Figure 3. Crystal structure of **RhS-1f**.

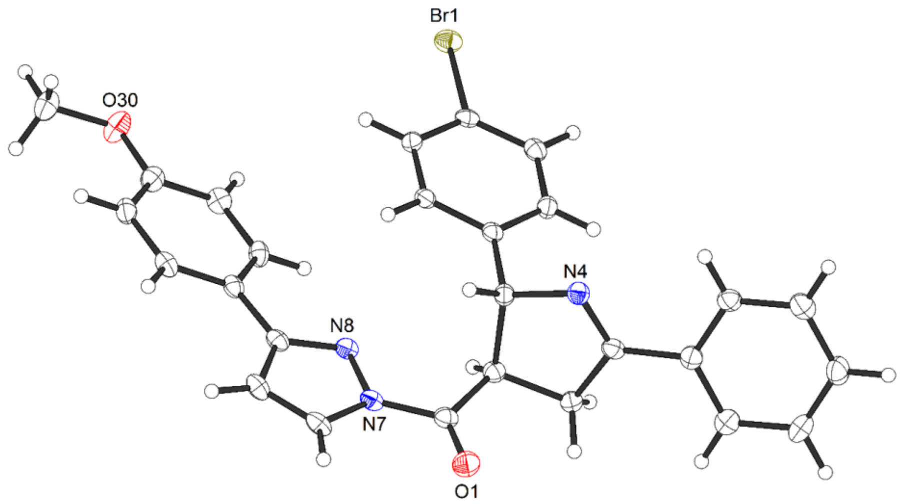

Supplementary Figure 4. Crystal structure of **3k**.

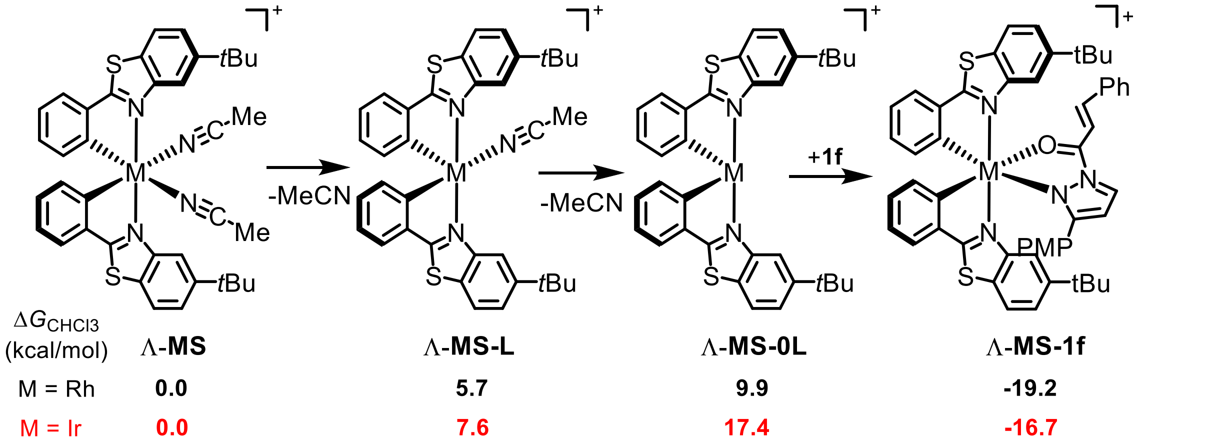

Supplementary Figure 5. Calculated energy profiles for ligand exchanges with **RhS** and **IrS**. For the coordinates of intermediates, see Supplementary Data.

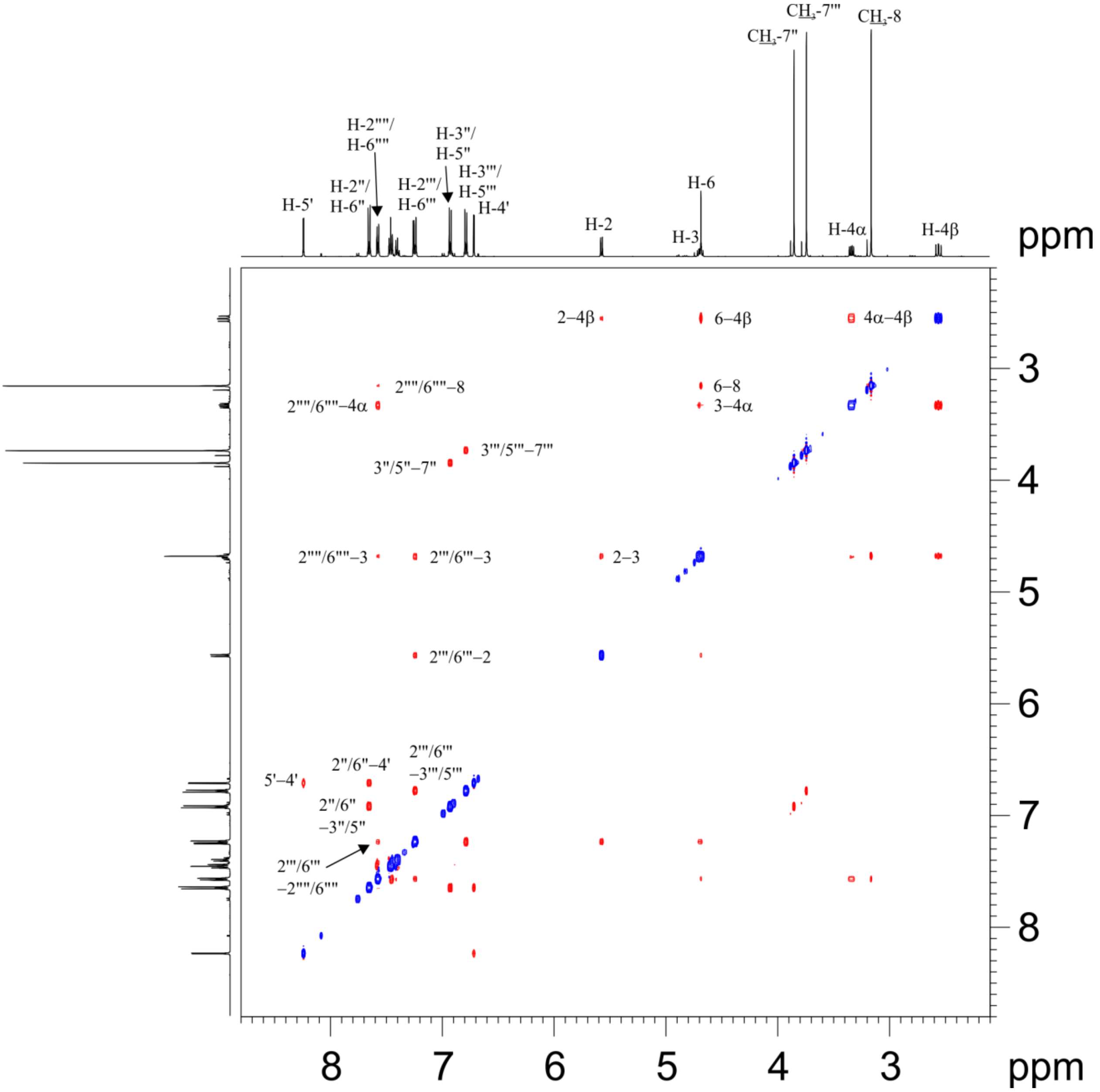

Supplementary Figure 6. NOESY spectrum of **8** in $CDCl_3$ at 300 K, mixing time 1.5 s.

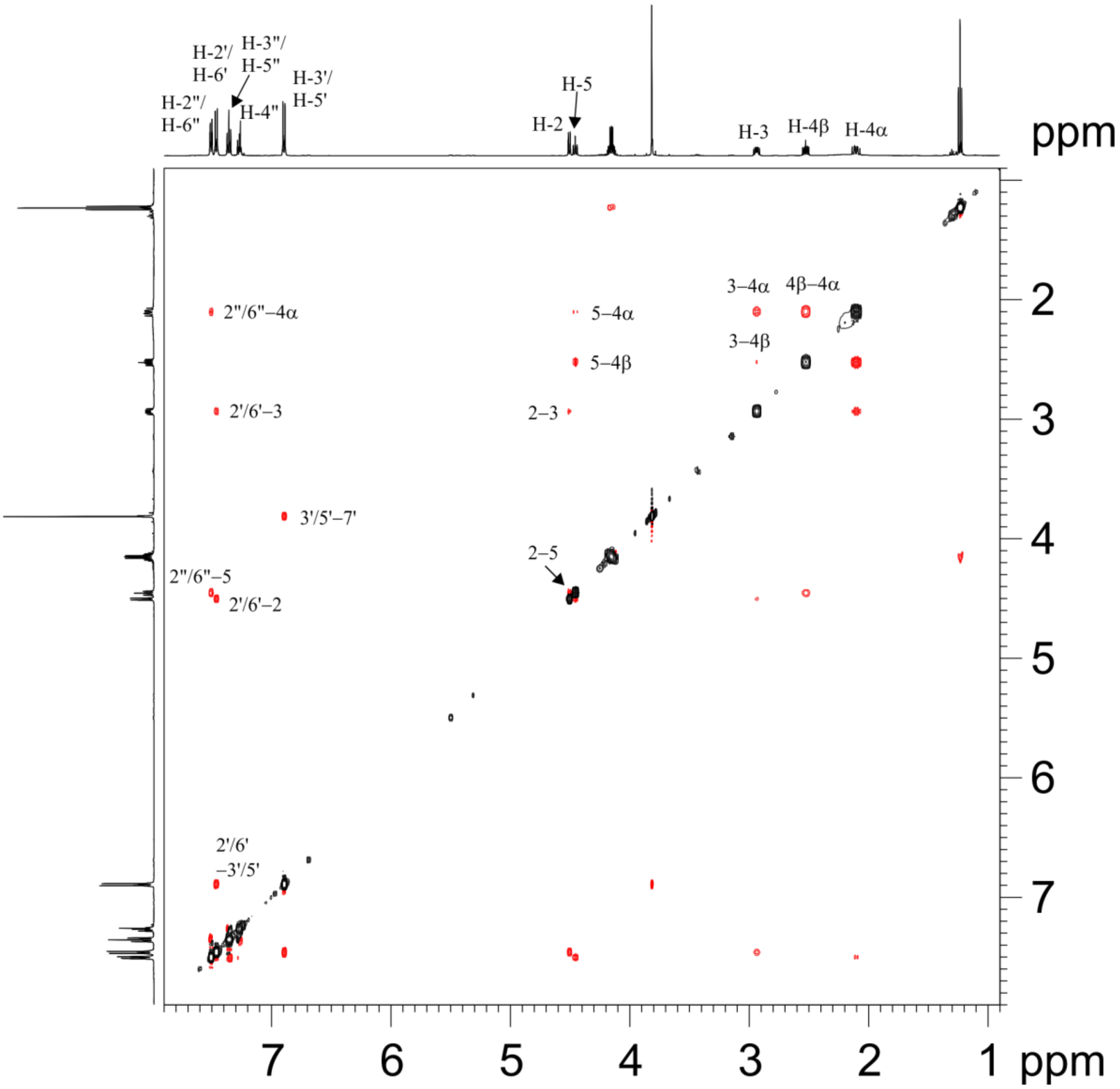

Supplementary Figure 7. NOESY spectrum of **10** in $CDCl_3$ at 300 K, mixing time 1.5 s.

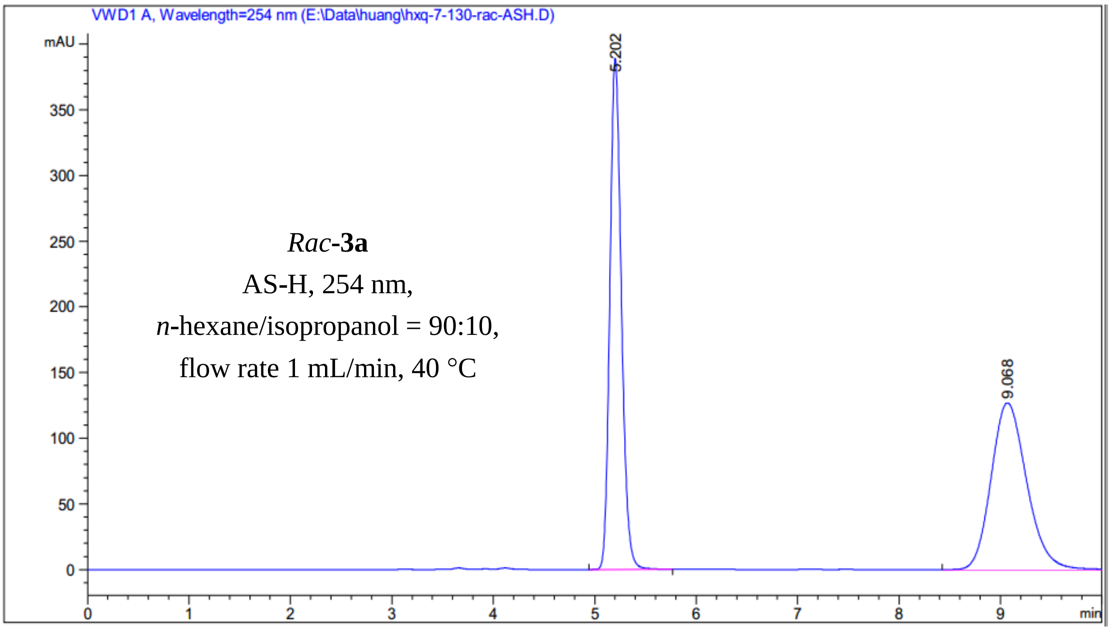

Peak #	RetTime [min]	Type	Width [min]	Area [mAU*s]	Height [mAU]	Area %
1	5.202	BB	0.1220	3067.24048	389.00543	49.8191
2	9.068	BB	0.3770	3089.51074	126.92117	50.1809

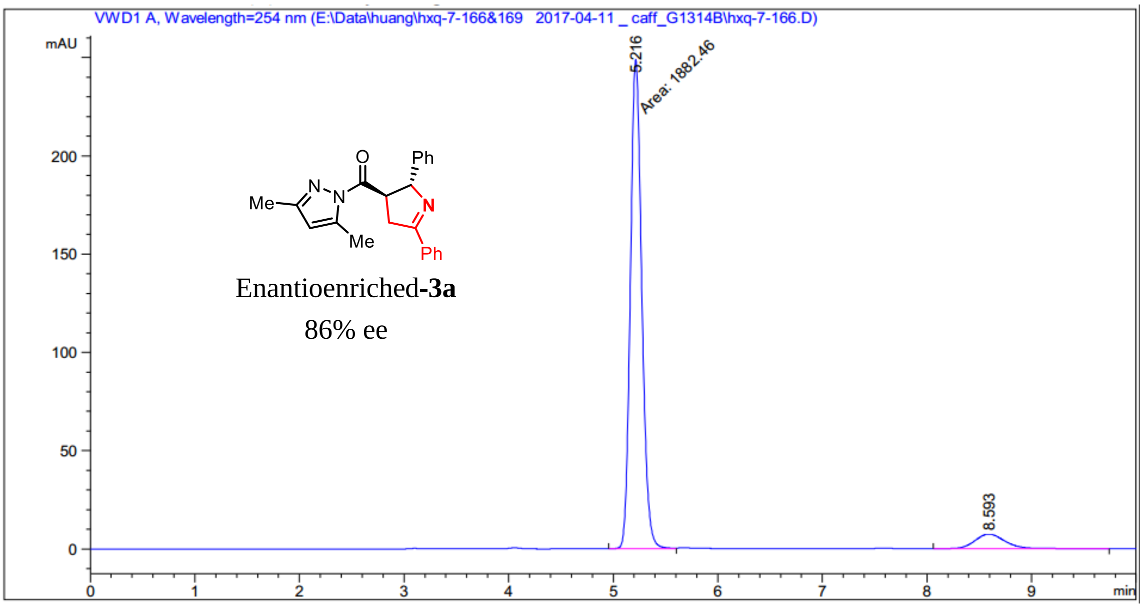

Peak #	RetTime [min]	Type	Width [min]	Area [mAU*s]	Height [mAU]	Area %
1	5.216	MF	0.1261	1882.46423	248.86484	92.8608
2	8.593	BB	0.3059	144.72408	7.34275	7.1392

Supplementary Figure 8. HPLC traces of *rac*-**3a** (reference) and enantioenriched-**3a**.

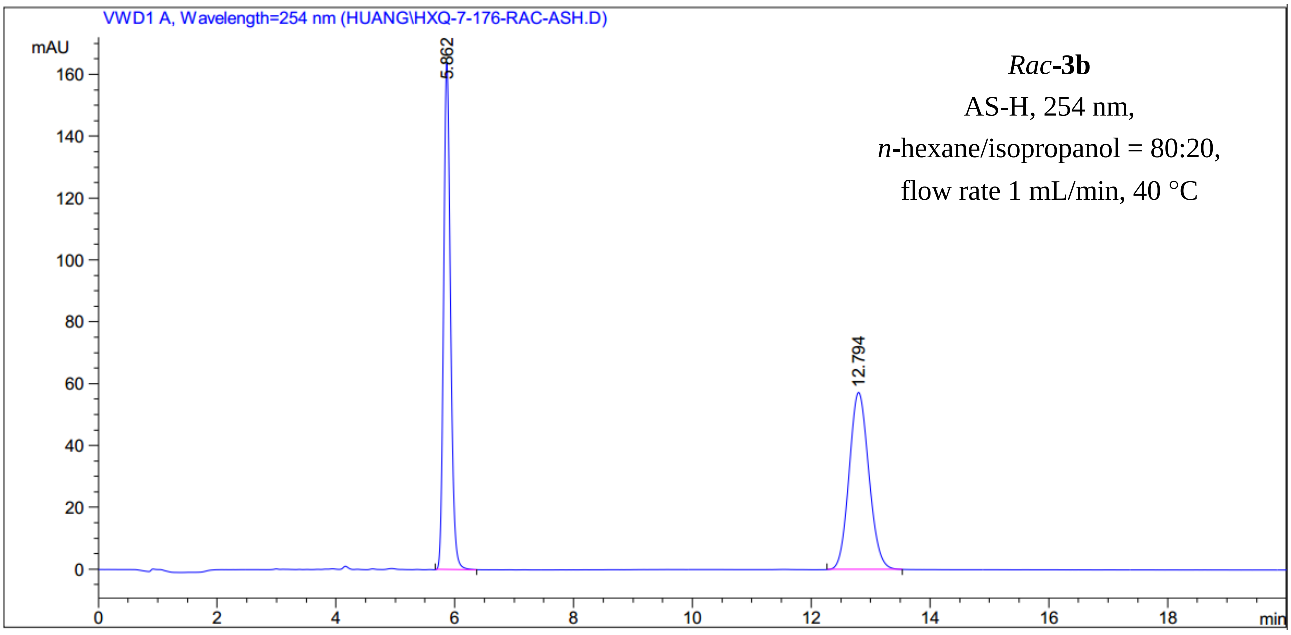

Peak #	RetTime [min]	Type	Width [min]	Area mAU *s	Height [mAU]	Area %
1	5.862	BB	0.1244	1319.06323	163.97075	49.9447
2	12.794	BB	0.3580	1321.98486	57.29019	50.0553

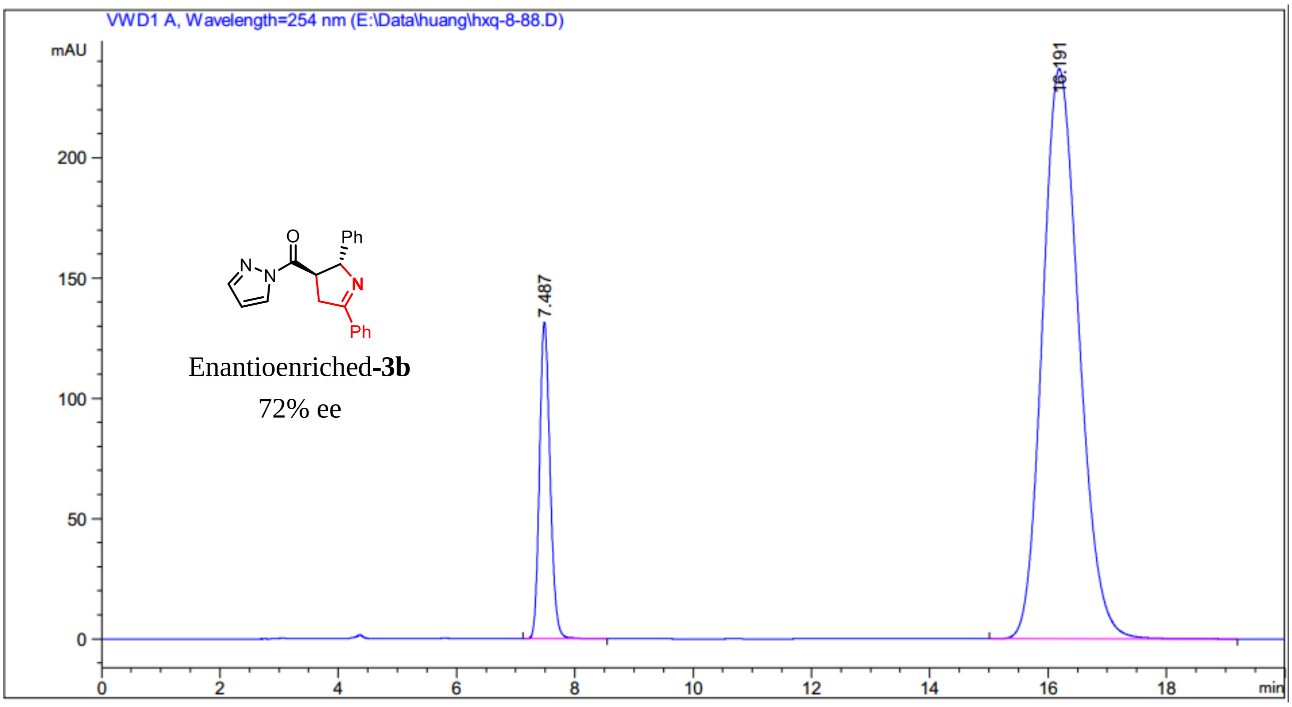

Peak #	RetTime [min]	Type	Width [min]	Area [mAU*s]	Height [mAU]	Area %
1	7.487	BB	0.1919	1634.28467	131.57304	13.8515
2	16.191	BB	0.6686	1.01643e4	236.87003	86.1485

Supplementary Figure 9. HPLC traces of *rac*-**3b** (reference) and enantioenriched-**3b**.

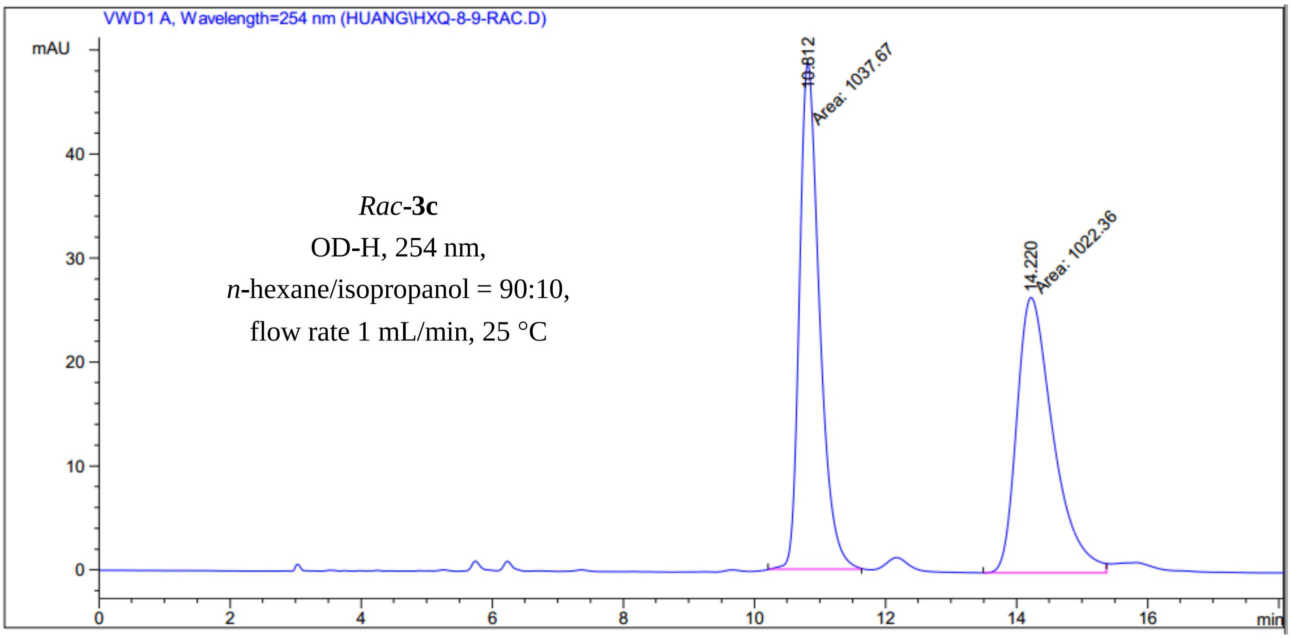

Peak #	RetTime [min]	Type	Width [min]	Area mAU *s	Height [mAU]	Area %
1	10.812	MM	0.3550	1037.67065	48.71235	50.3716
2	14.220	MM	0.6431	1022.35864	26.49449	49.6284

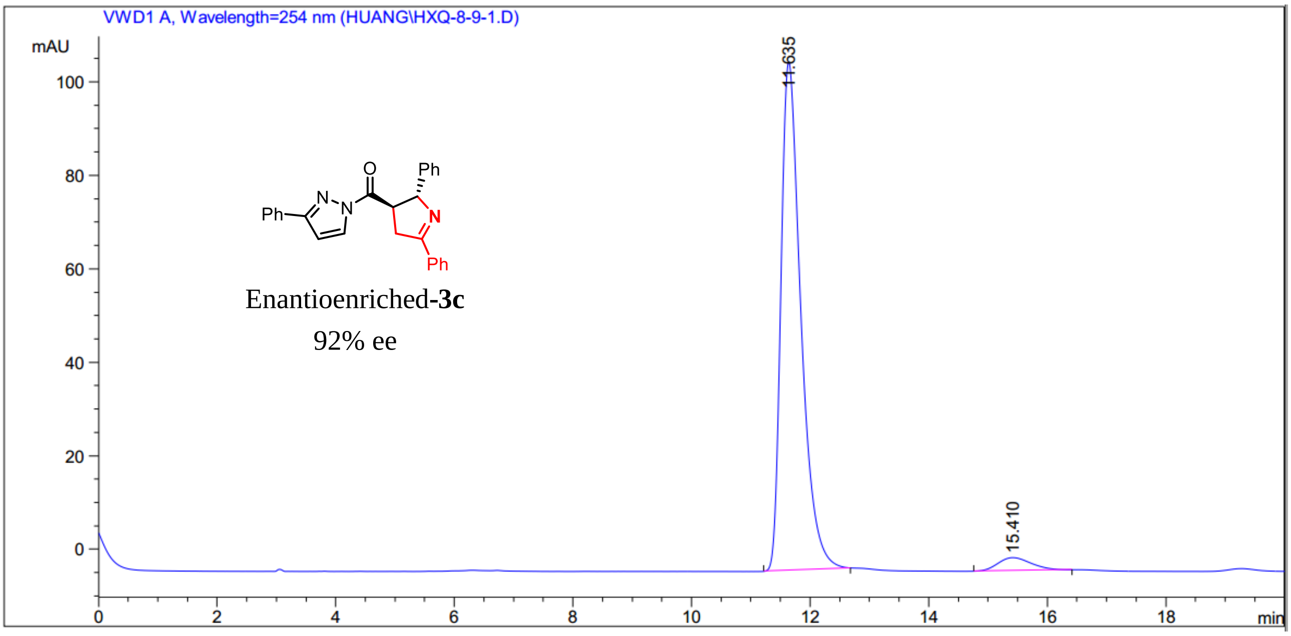

Peak #	RetTime [min]	Type	Width [min]	Area mAU *s	Height [mAU]	Area %
1	11.635	BB	0.3536	2537.93774	108.84882	95.8897
2	15.410	BB	0.5778	108.78958	2.78650	4.1103

Supplementary Figure 10. HPLC traces of *rac*-**3c** (reference) and enantioenriched-**3c**.

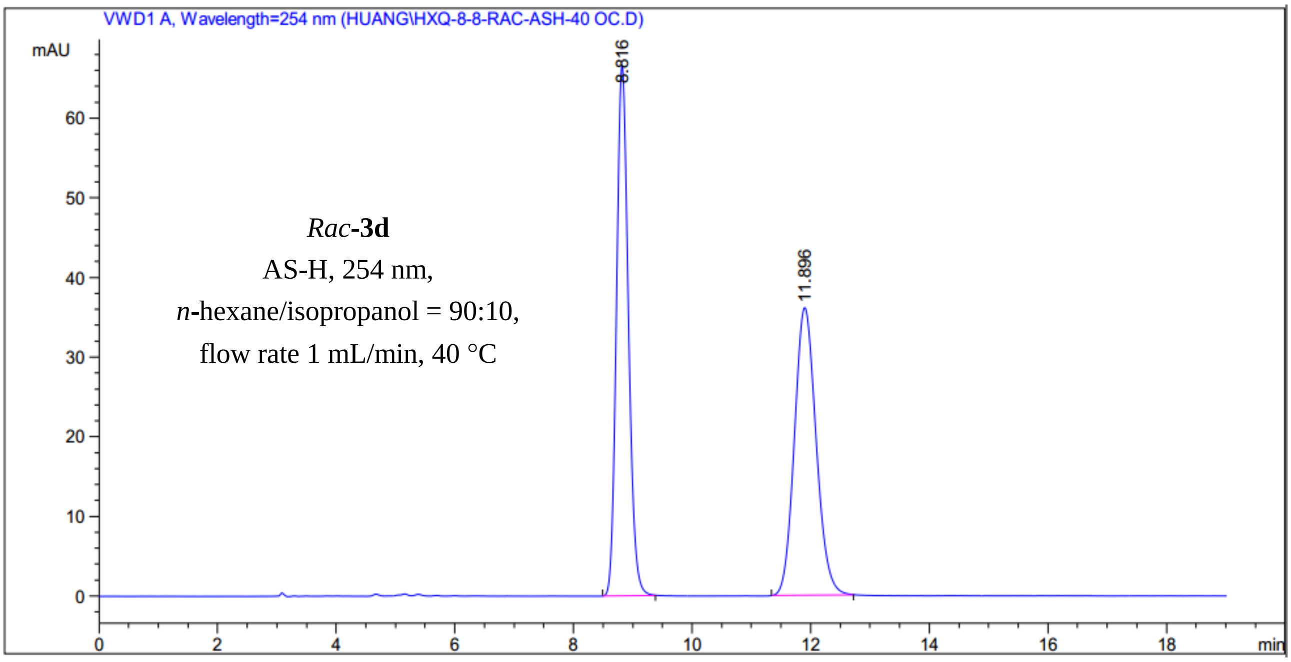

Peak #	RetTime [min]	Type	Width [min]	Area mAU *s	Height [mAU]	Area %
1	8.816	BB	0.2151	928.17261	66.57808	50.1780
2	11.896	BB	0.3950	921.58887	36.14841	49.8220

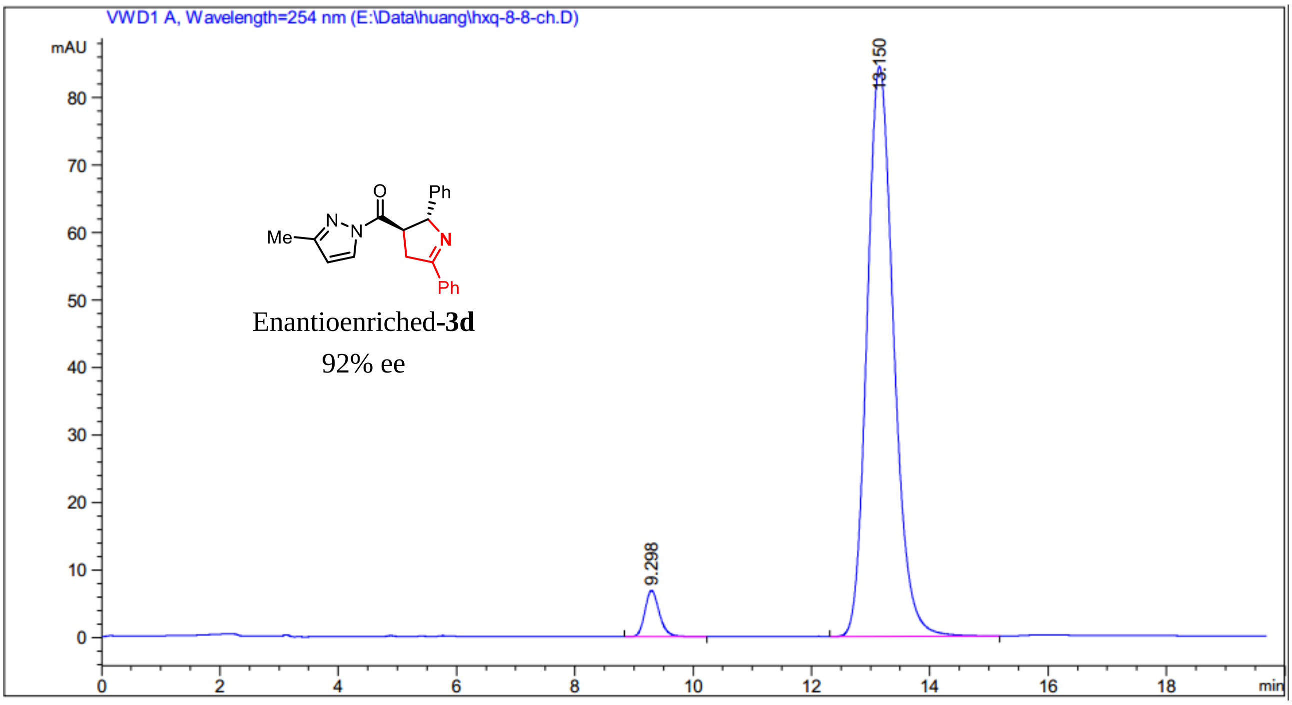

Peak #	RetTime [min]	Type	Width [min]	Area [mAU*s]	Height [mAU]	Area %
1	9.298	BB	0.2602	115.41849	6.82962	4.1833
2	13.150	BB	0.4834	2643.64282	84.40820	95.8167

Supplementary Figure 11. HPLC traces of *rac*-**3d** (reference) and enantioenriched-**3d**.

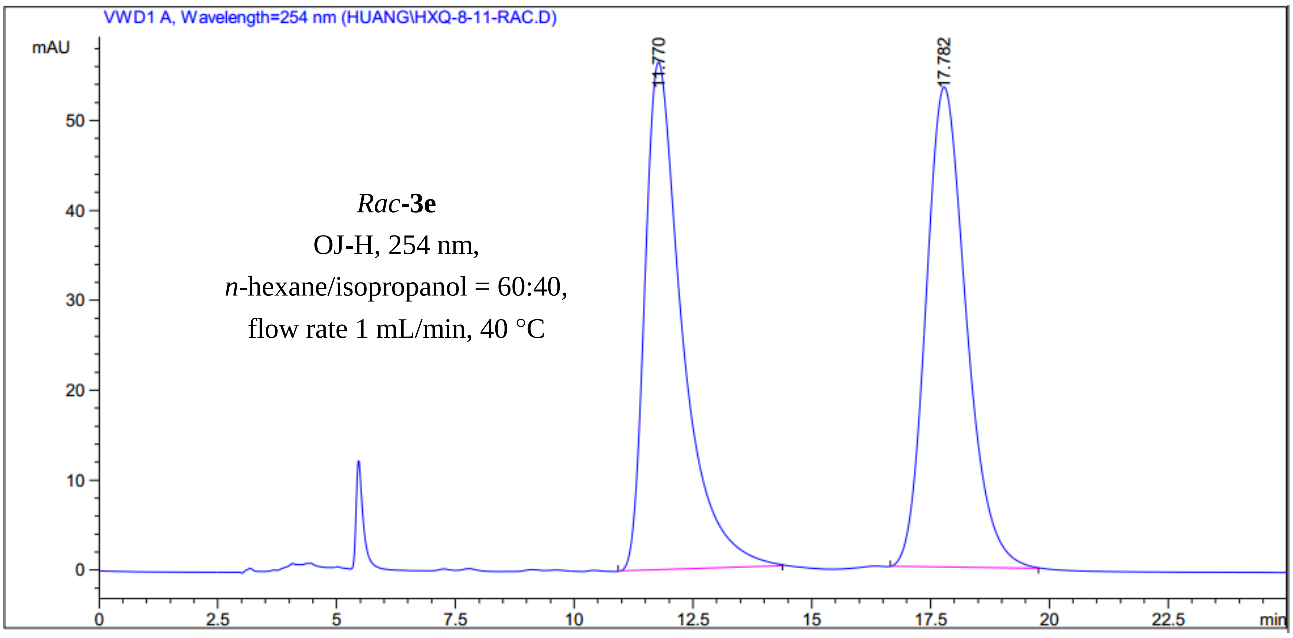

Peak #	RetTime [min]	Type	Width [min]	Area mAU *s	Height [mAU]	Area %
1	11.770	BB	0.8156	3103.68042	56.49596	49.6091
2	17.782	BB	0.9083	3152.59497	53.42775	50.3909

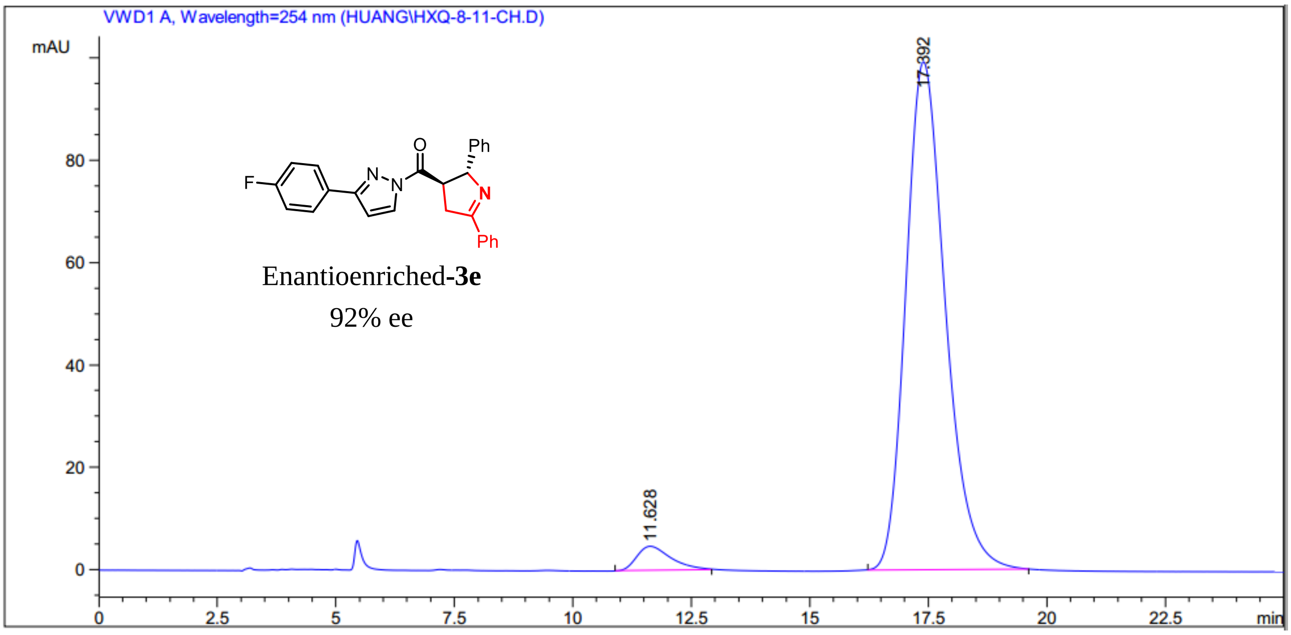

Peak #	RetTime [min]	Type	Width [min]	Area mAU *s	Height [mAU]	Area %
1	11.628	BB	0.7389	237.36055	4.72509	3.9585
2	17.392	BB	0.8927	5758.85156	99.21601	96.0415

Supplementary Figure 12. HPLC traces of *rac*-**3e** (reference) and enantioenriched-**3e**.

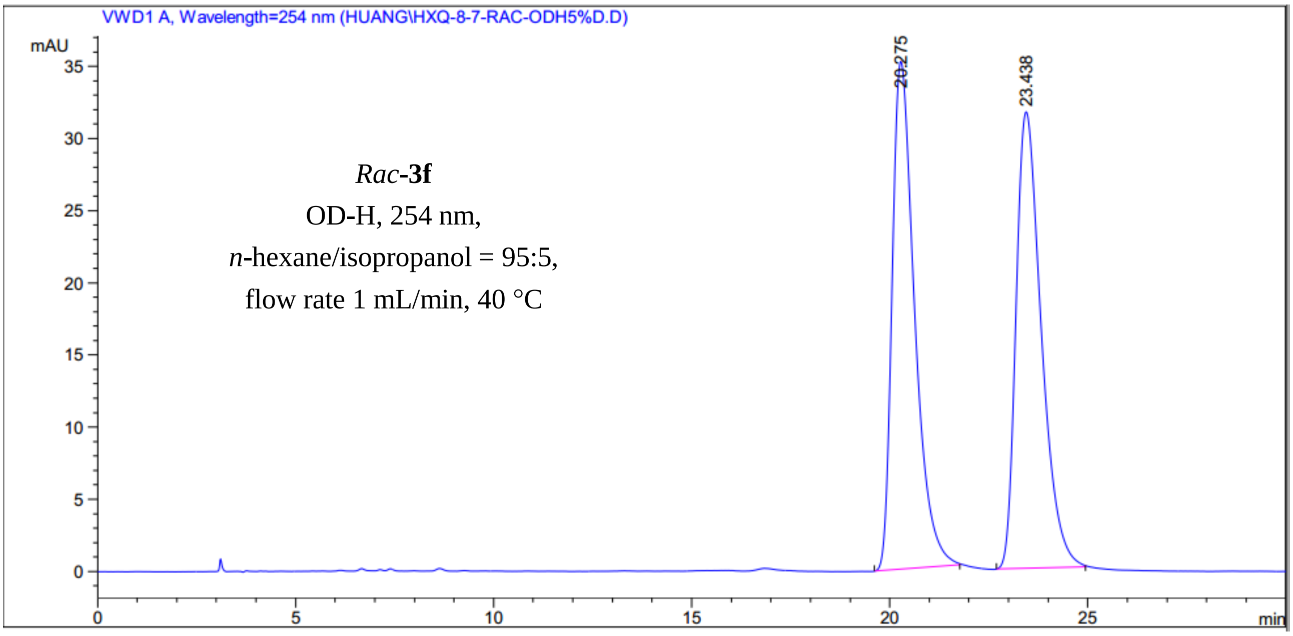

Peak #	RetTime [min]	Type	Width [min]	Area mAU *s	Height [mAU]	Area %
1	20.275	BB	0.6023	1386.52234	35.19300	49.7248
2	23.438	BB	0.6743	1401.86987	31.63187	50.2752

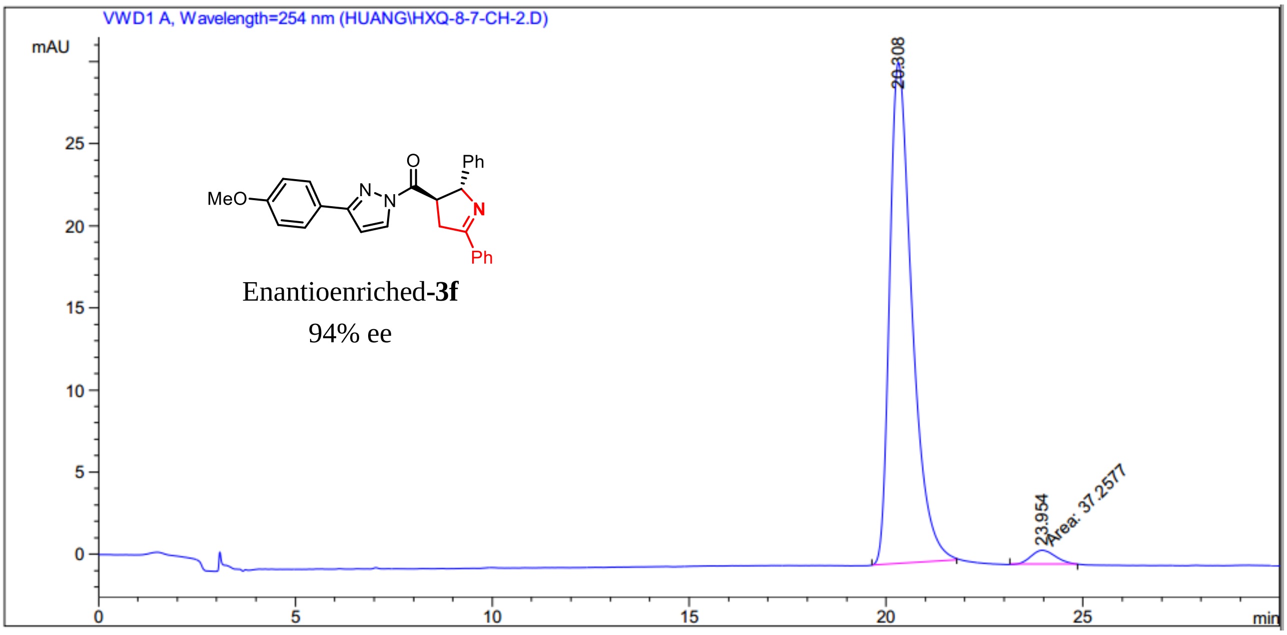

Peak #	RetTime [min]	Type	Width [min]	Area mAU *s	Height [mAU]	Area %
1	20.308	BB	0.6019	1204.86731	30.51107	97.0005
2	23.954	MM	0.7276	37.25768	8.53430e-1	2.9995

Supplementary Figure 13. HPLC traces of *rac*-**3f** (reference) and enantioenriched-**3f**.

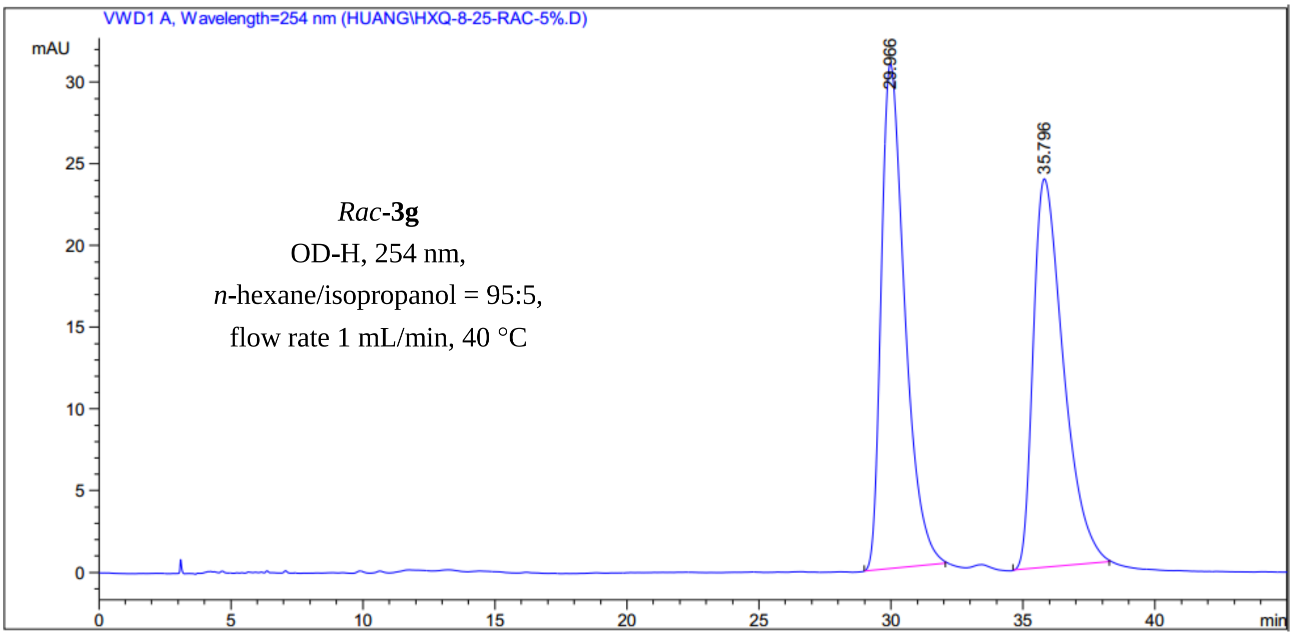

Peak #	RetTime [min]	Type	Width [min]	Area mAU *s	Height [mAU]	Area %
1	29.966	BB	0.9390	1921.63757	30.92322	50.3553
2	35.796	BB	1.1820	1894.52197	23.80040	49.6447

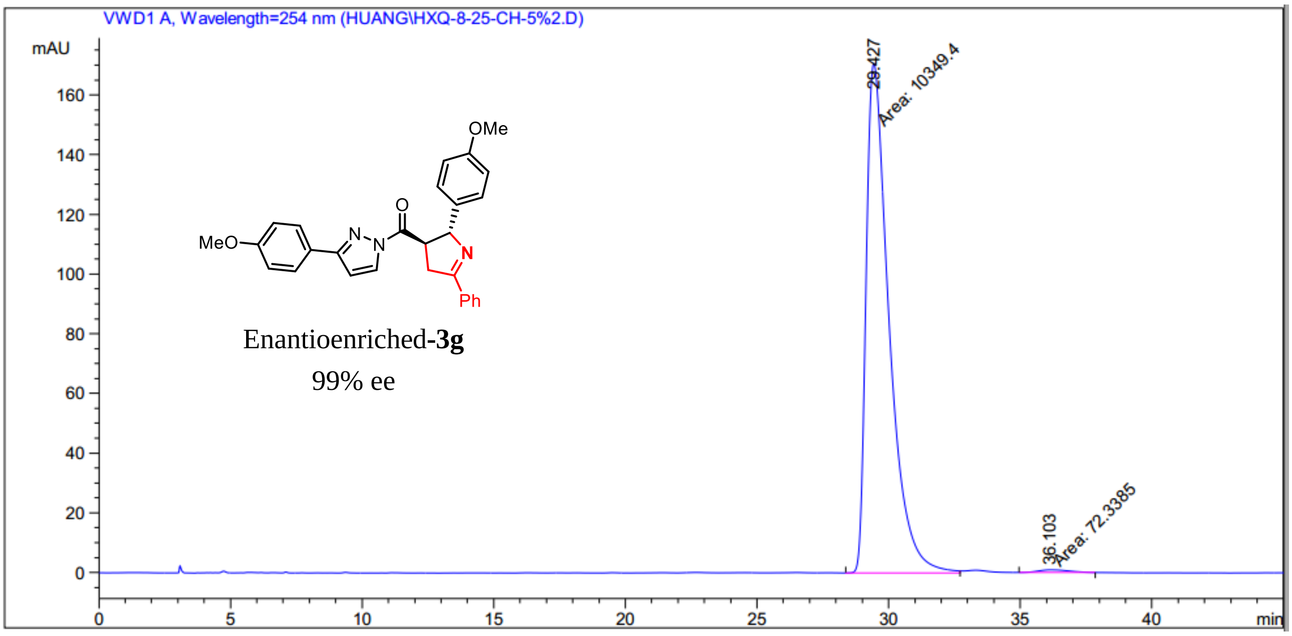

Peak #	RetTime [min]	Type	Width [min]	Area mAU *s	Height [mAU]	Area %
1	29.427	MM	1.0103	1.03494e4	170.73683	99.3059
2	36.103	MM	1.3523	72.33855	8.91540e-1	0.6941

Supplementary Figure 14. HPLC traces of *rac*-**3g** (reference) and enantioenriched-**3g**.

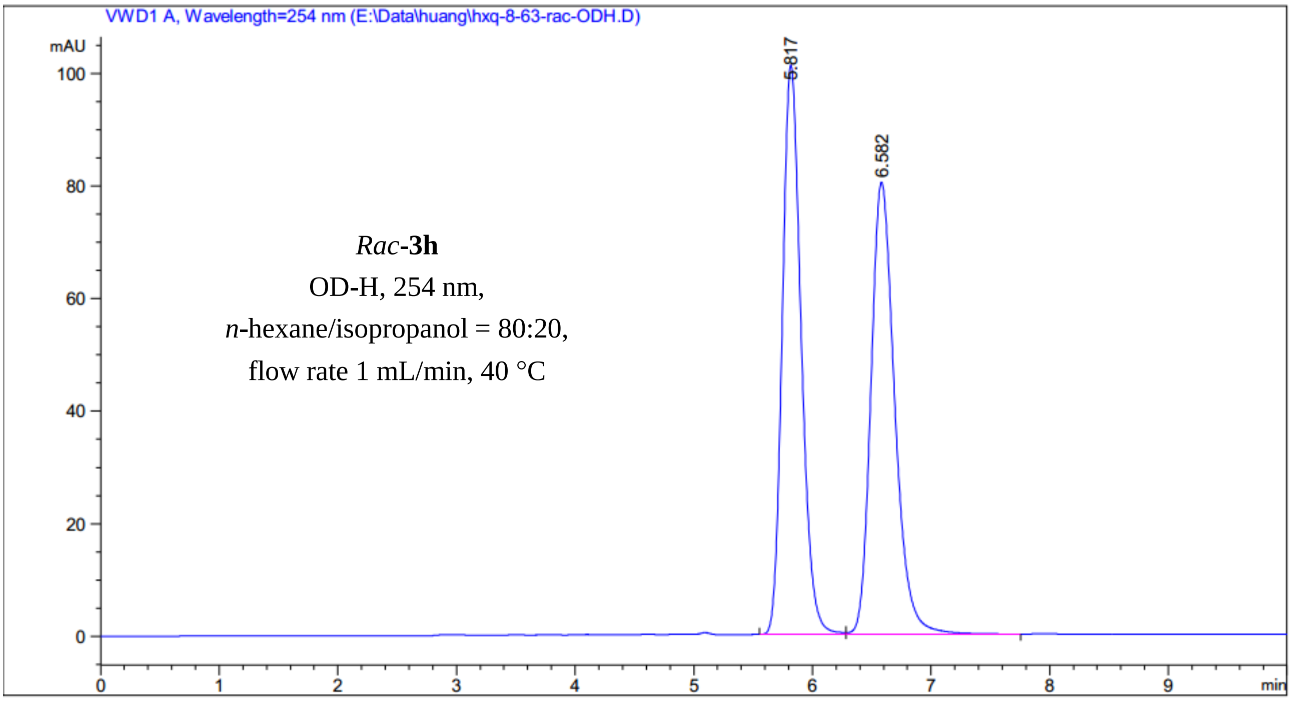

Peak #	RetTime [min]	Type	Width [min]	Area [mAU*s]	Height [mAU]	Area %
1	5.817	BV	0.1707	1116.00549	101.17491	49.9708
2	6.582	VB	0.2133	1117.30957	80.30215	50.0292

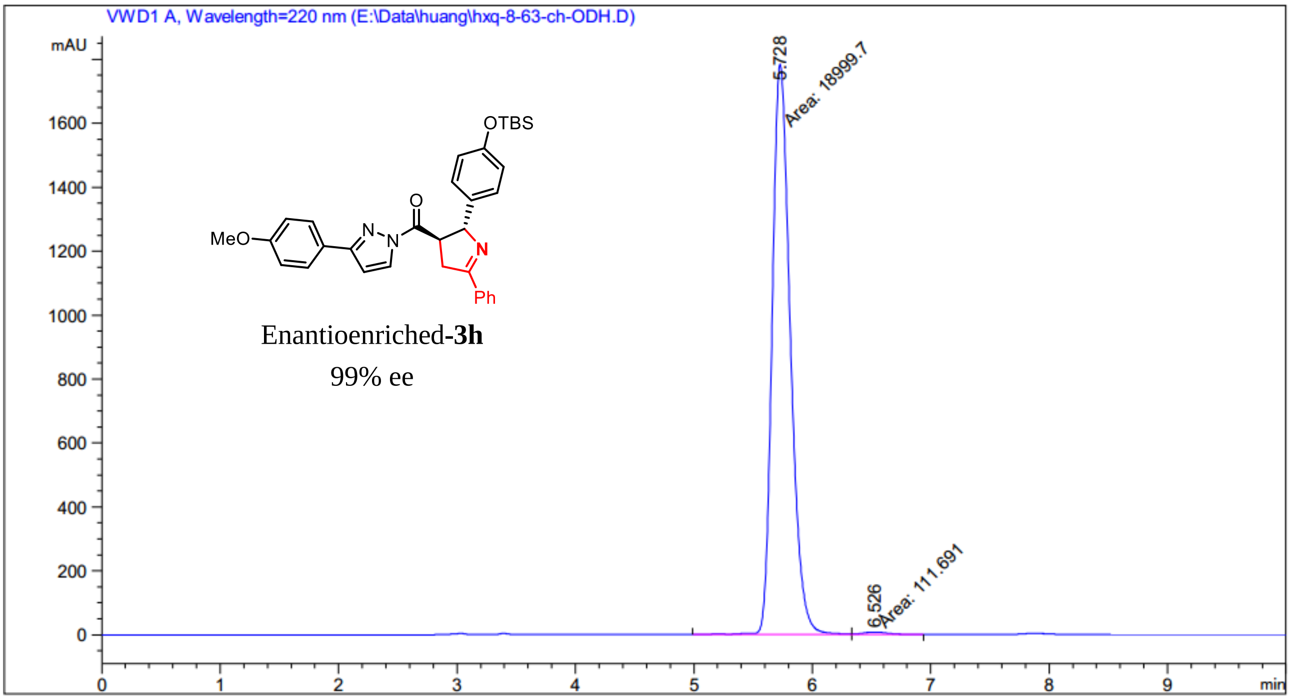

Peak #	RetTime [min]	Type	Width [min]	Area [mAU*s]	Height [mAU]	Area %
1	5.728	MF	0.1775	1.89997e4	1783.57654	99.4156
2	6.526	FM	0.2407	111.69097	7.73289	0.5844

Supplementary Figure 15. HPLC traces of *rac*-**3h** (reference) and enantioenriched-**3h**.

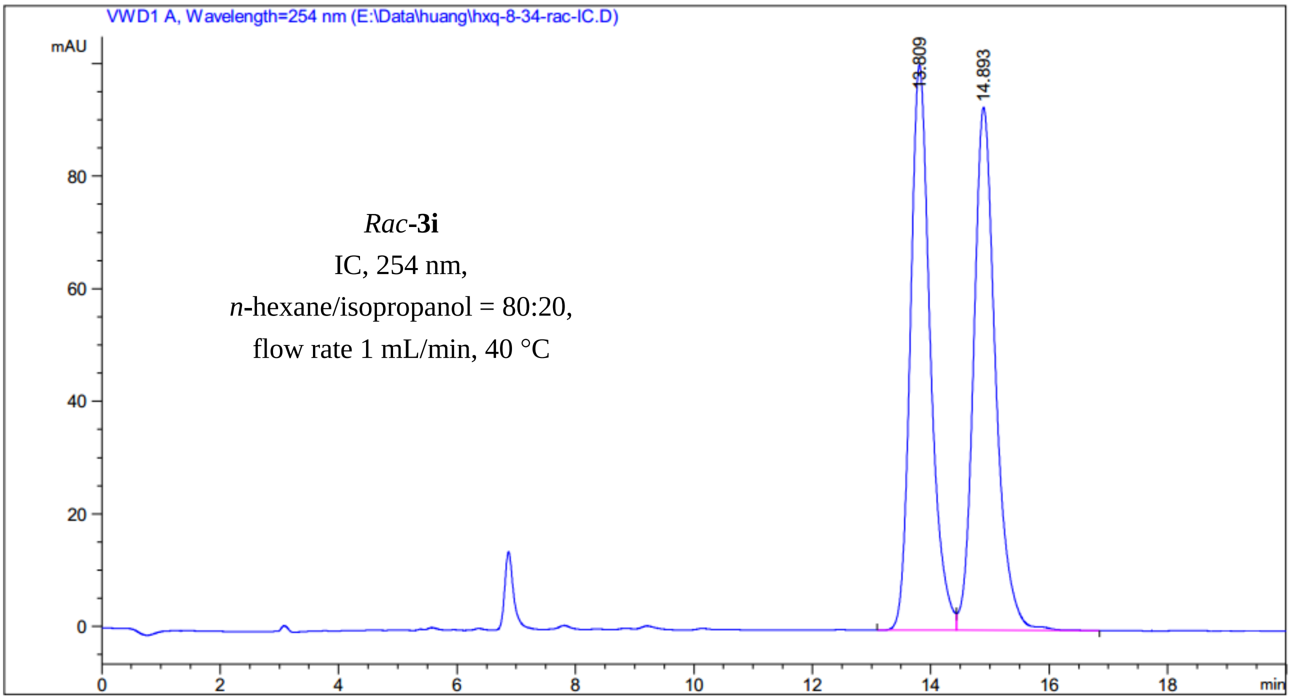

Peak #	RetTime [min]	Type	Width [min]	Area [mAU*s]	Height [mAU]	Area %
1	13.809	BV	0.3540	2350.08008	100.43159	49.5758
2	14.893	VB	0.3890	2390.29419	92.94417	50.4242

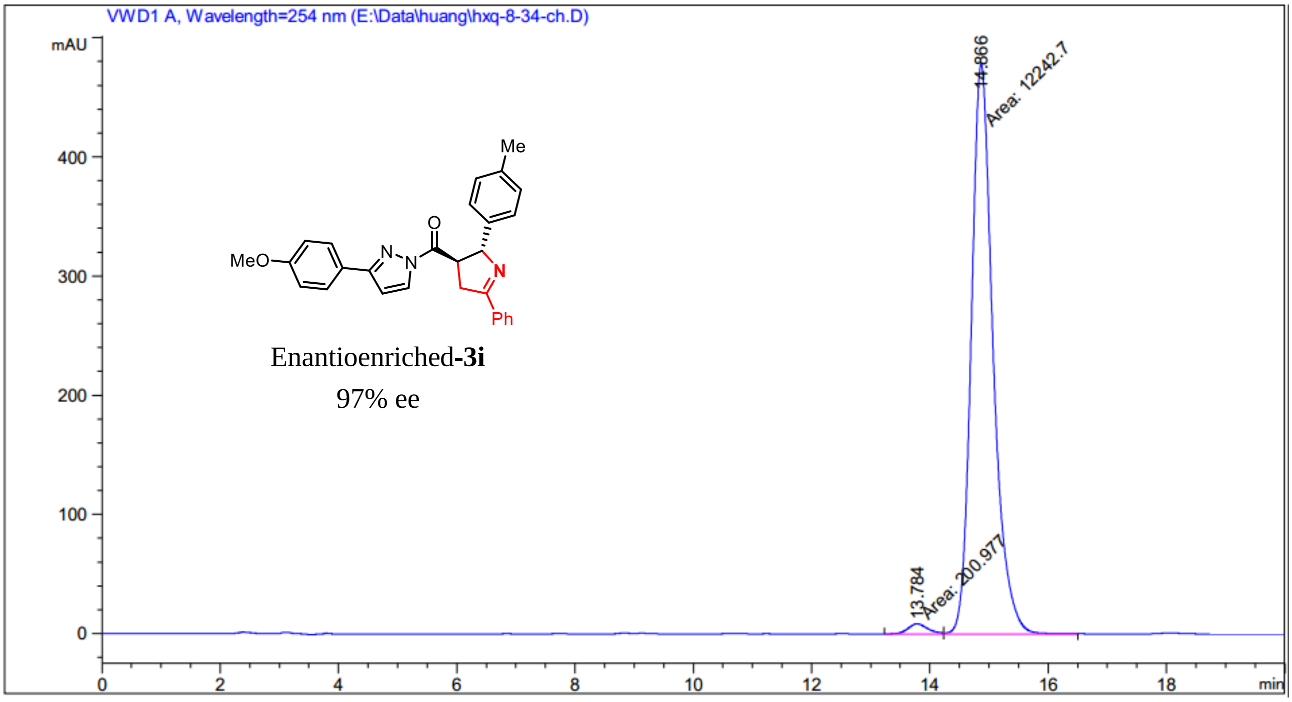

Peak #	RetTime [min]	Type	Width [min]	Area [mAU*s]	Height [mAU]	Area %
1	13.784	MF	0.3941	200.97655	8.49877	1.6151
2	14.866	FM	0.4267	1.22427e4	478.14899	98.3849

Supplementary Figure 16. HPLC traces of *rac*-**3i** (reference) and enantioenriched-**3i**.

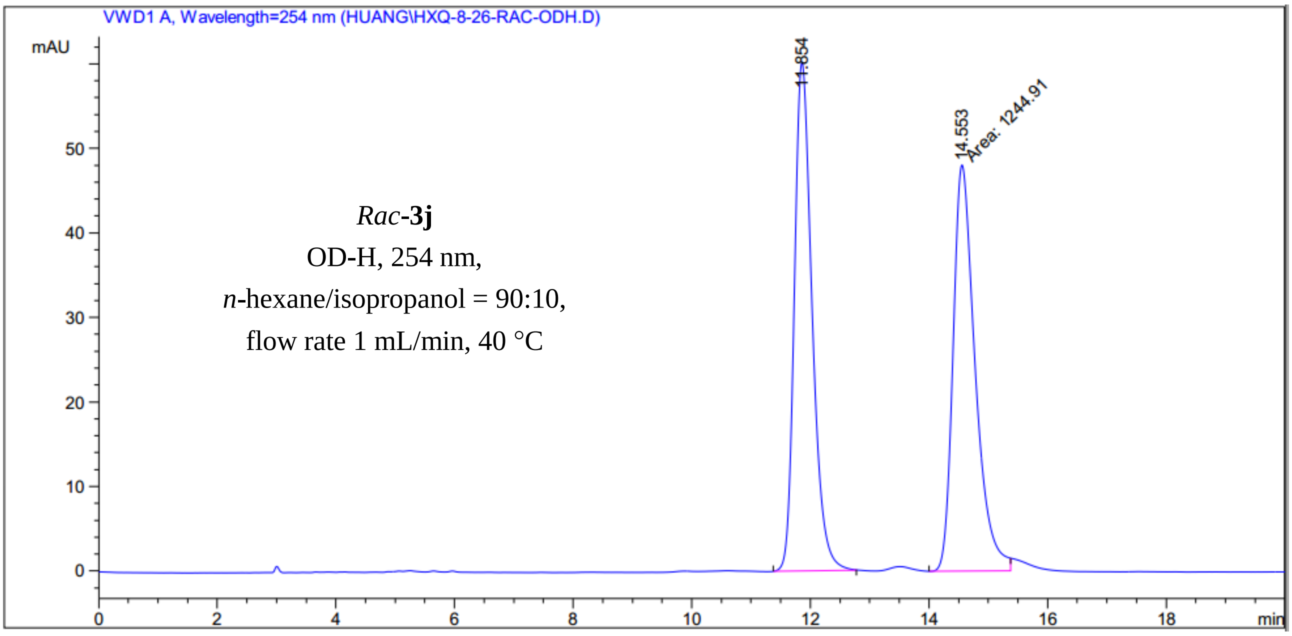

Peak #	RetTime [min]	Type	Width [min]	Area mAU *s	Height [mAU]	Area %
1	11.854	BB	0.3179	1250.33899	60.22337	50.1088
2	14.553	MF	0.4318	1244.91052	48.05161	49.8912

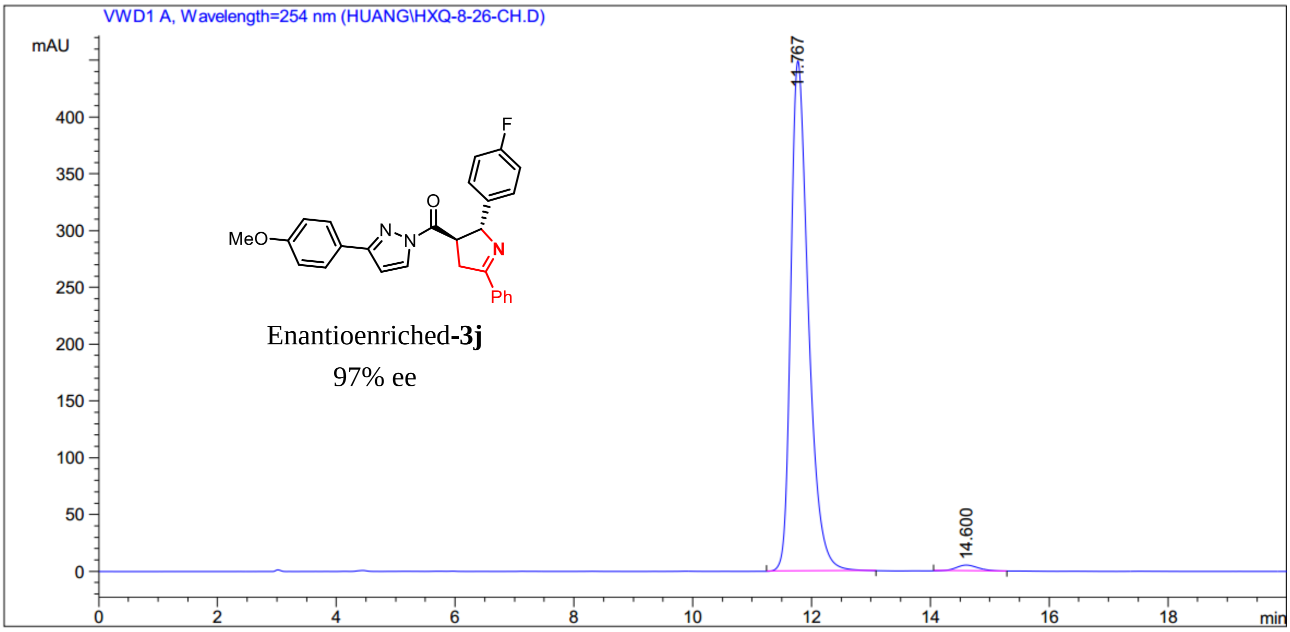

Peak #	RetTime [min]	Type	Width [min]	Area mAU *s	Height [mAU]	Area %
1	11.767	BB	0.3149	9265.60742	449.29520	98.5551
2	14.600	BB	0.4045	135.84334	5.13921	1.4449

Supplementary Figure 17. HPLC traces of *rac*-**3j** (reference) and enantioenriched-**3j**.

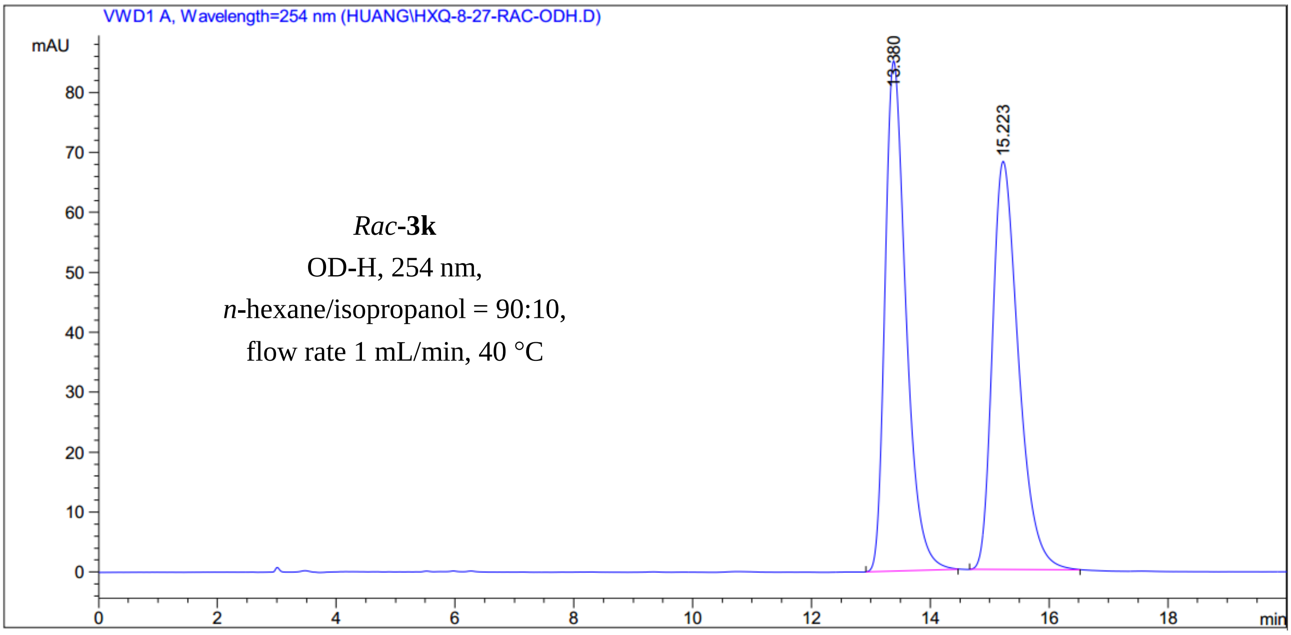

Peak #	RetTime [min]	Type	Width [min]	Area mAU *s	Height [mAU]	Area %
1	13.380	BB	0.3752	2088.49316	85.09290	50.0998
2	15.223	BB	0.4672	2080.17651	68.07524	49.9002

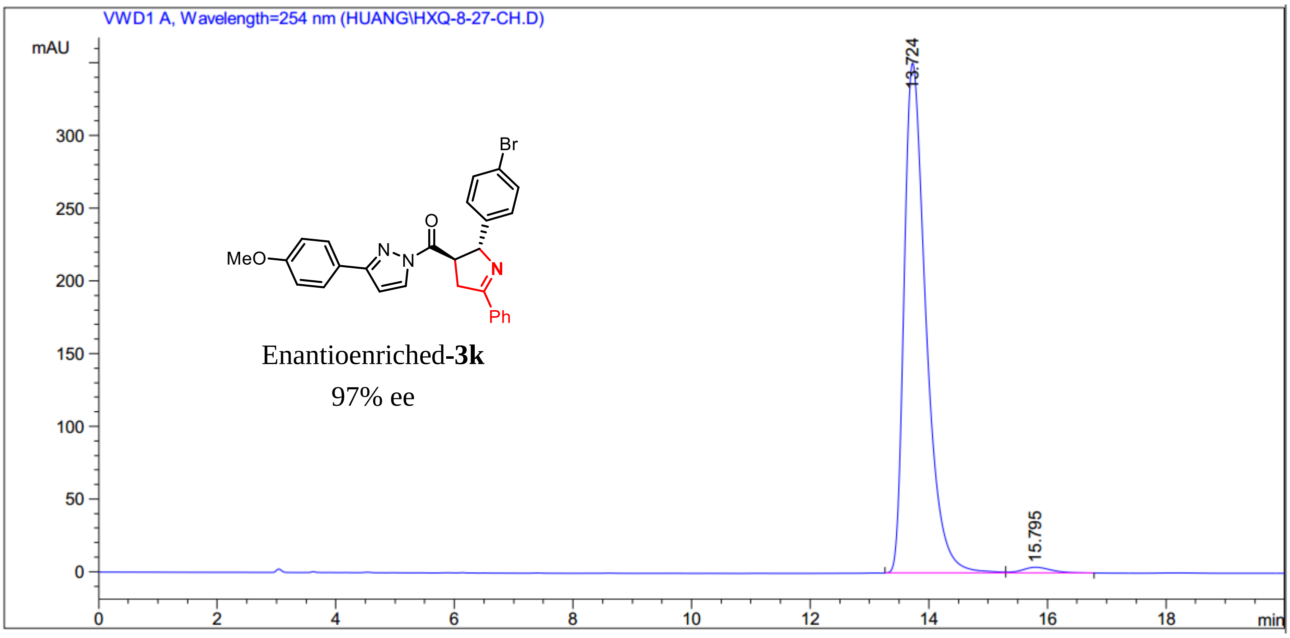

Peak #	RetTime [min]	Type	Width [min]	Area mAU *s	Height [mAU]	Area %
1	13.724	BB	0.3895	8953.82715	350.77350	98.4752
2	15.795	BB	0.5283	138.64171	3.96951	1.5248

Supplementary Figure 18. HPLC traces of *rac*-**3k** (reference) and enantioenriched-**3k**.

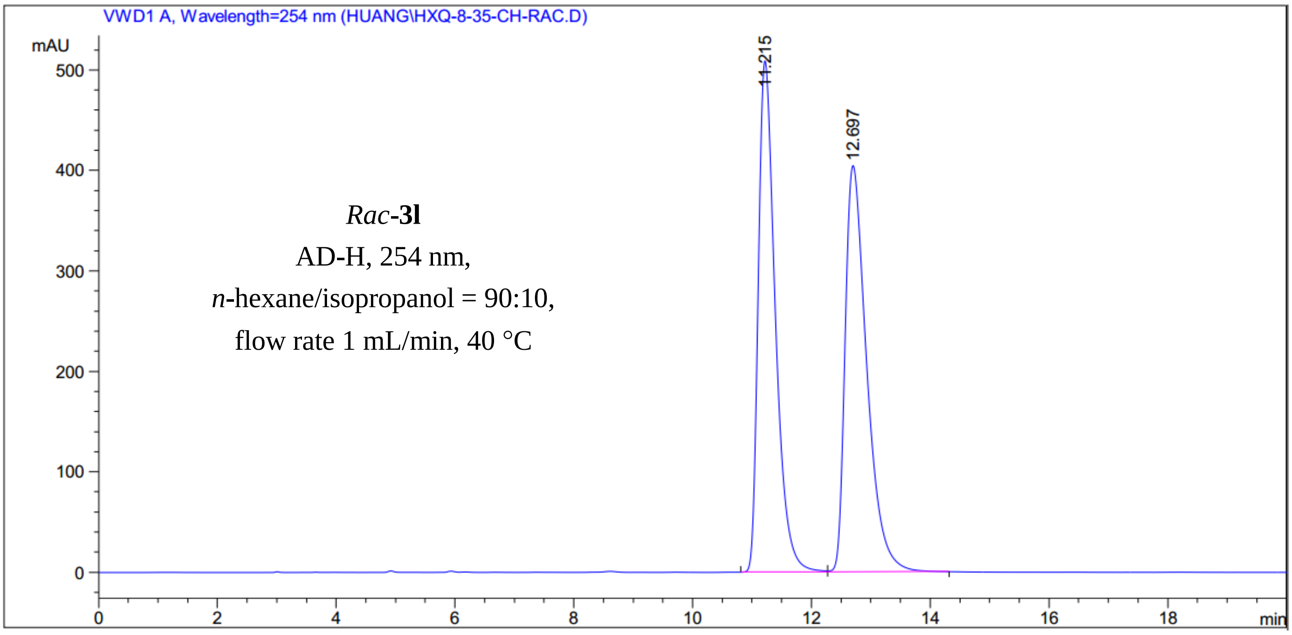

Peak #	RetTime [min]	Type	Width [min]	Area mAU *s	Height [mAU]	Area %
1	11.215	BV	0.3042	1.01571e4	508.96582	49.8128
2	12.697	VB	0.3854	1.02335e4	404.57022	50.1872

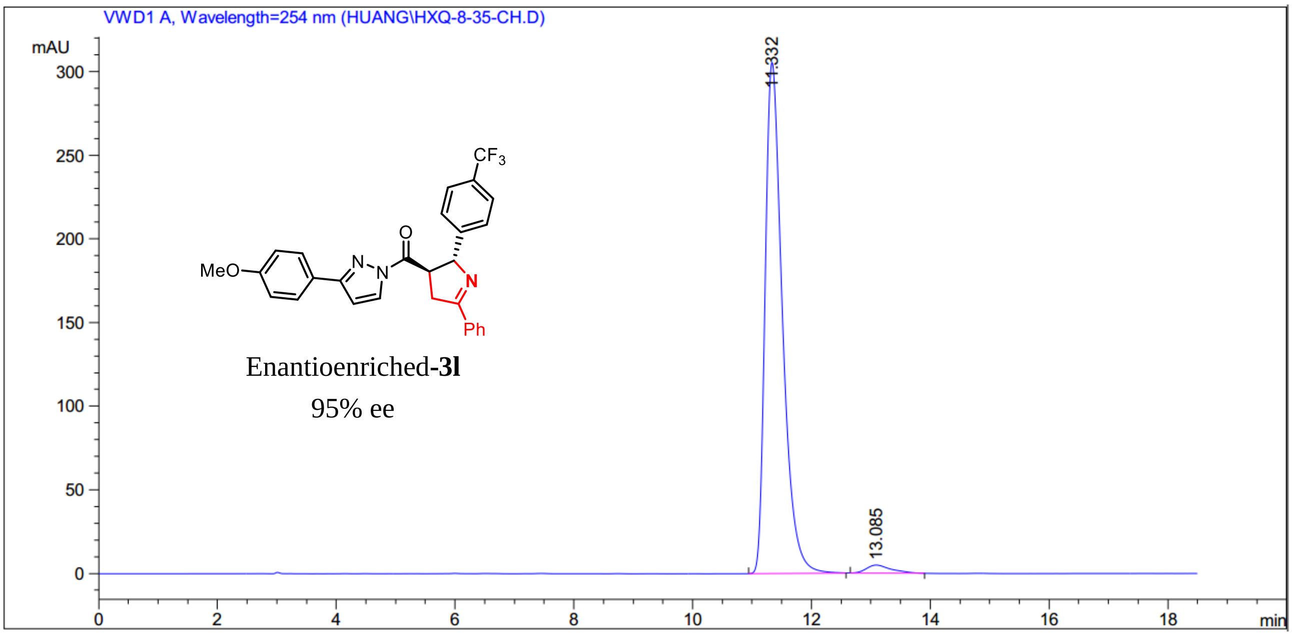

Peak #	RetTime [min]	Type	Width [min]	Area mAU *s	Height [mAU]	Area %
1	11.332	BB	0.3086	6174.15918	305.52954	97.5804
2	13.085	BB	0.4481	153.09477	5.03243	2.4196

Supplementary Figure 19. HPLC traces of *rac*-**3l** (reference) and enantioenriched-**3l**.

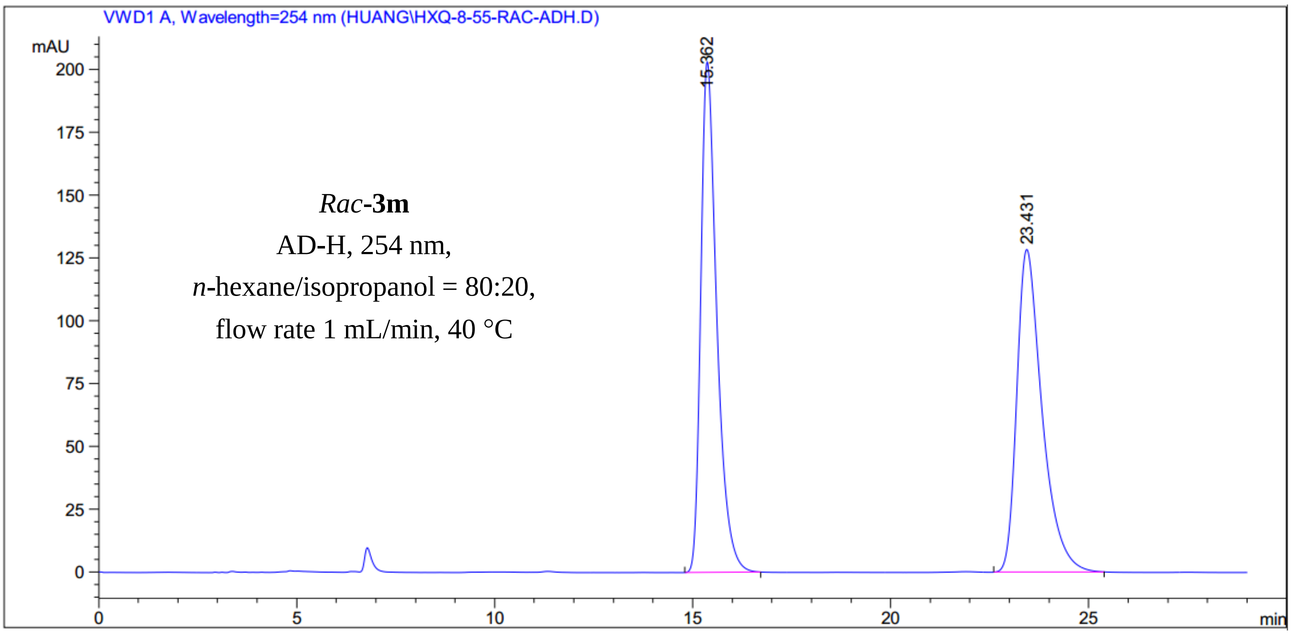

Peak #	RetTime [min]	Type	Width [min]	Area mAU *s	Height [mAU]	Area %
1	15.362	BB	0.4234	5724.49121	203.14597	50.0751
2	23.431	BB	0.6656	5707.32715	128.41513	49.9249

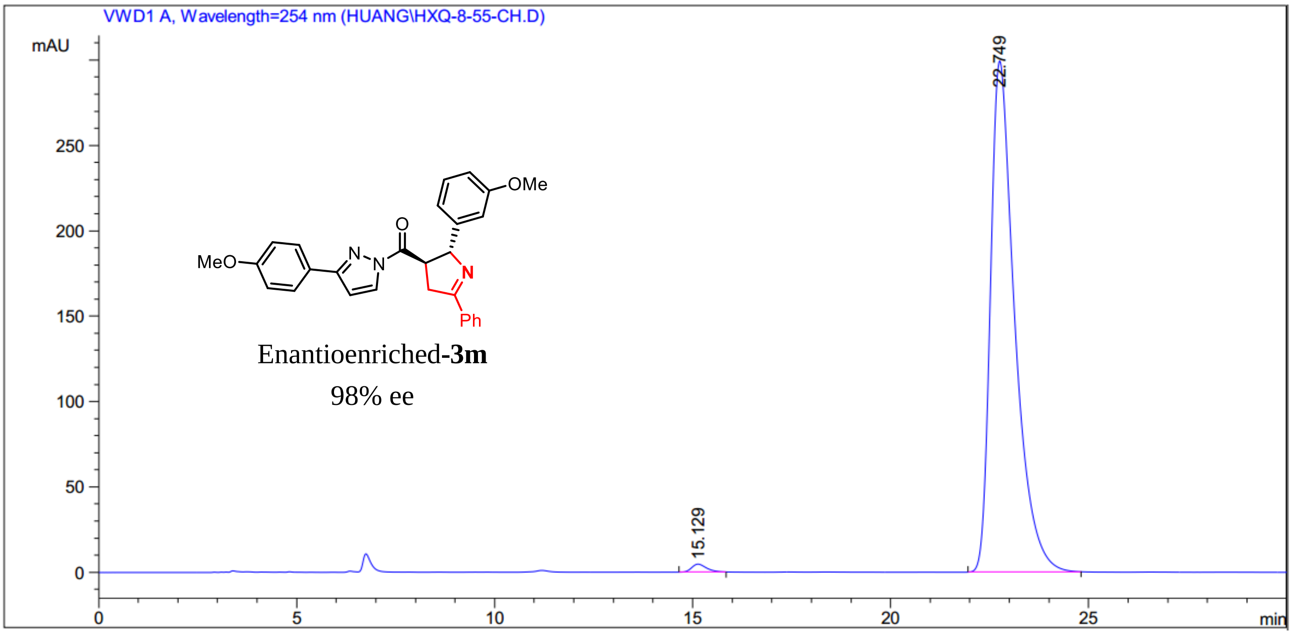

Peak #	RetTime [min]	Type	Width [min]	Area mAU *s	Height [mAU]	Area %
1	15.129	BB	0.4019	122.76633	4.68294	0.9550
2	22.749	BB	0.6360	1.27324e4	299.36273	99.0450

Supplementary Figure 20. HPLC traces of *rac*-**3m** (reference) and enantioenriched-**3m**.

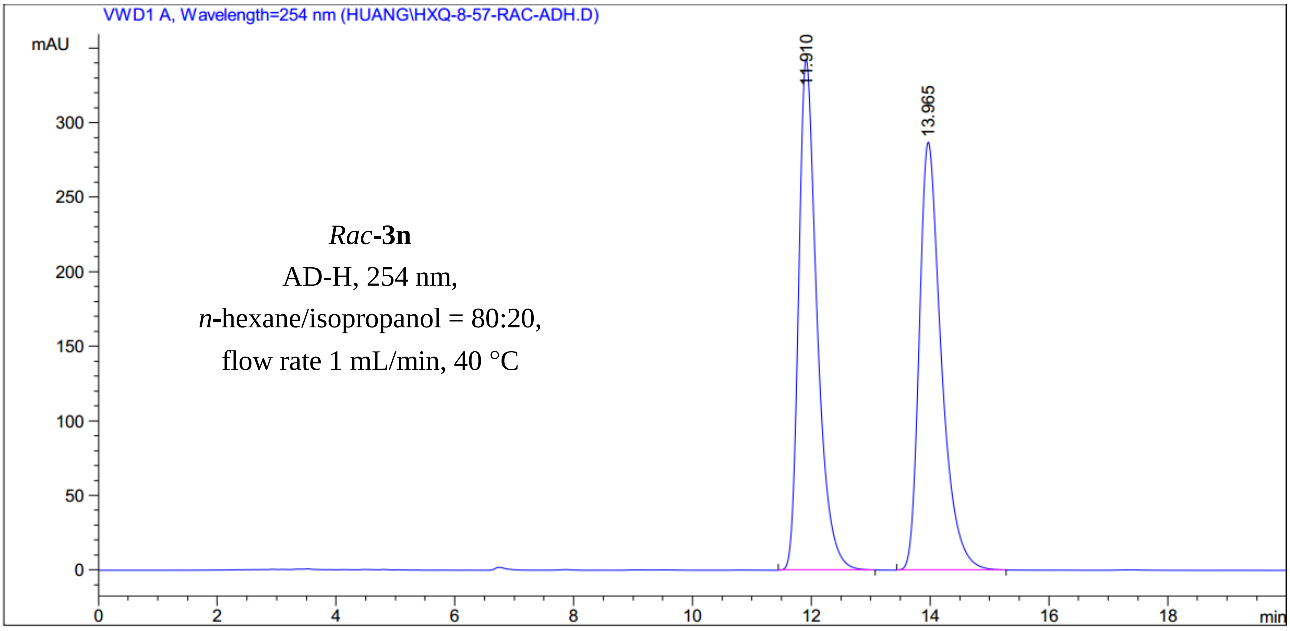

Peak #	RetTime [min]	Type	Width [min]	Area mAU *s	Height [mAU]	Area %
1	11.910	BB	0.3244	7382.64258	342.36911	49.9768
2	13.965	BB	0.3864	7389.50537	286.89661	50.0232

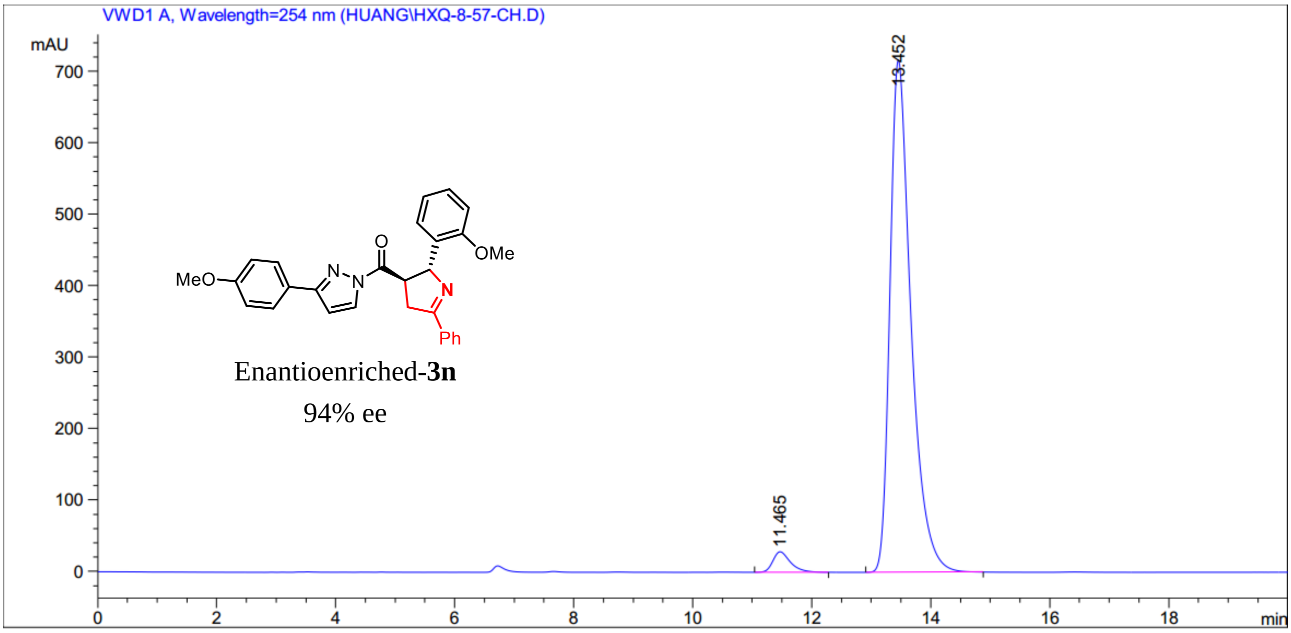

Peak #	RetTime [min]	Type	Width [min]	Area mAU *s	Height [mAU]	Area %
1	11.465	BB	0.3082	590.78223	28.74282	3.2493
2	13.452	BB	0.3678	1.75913e4	717.04144	96.7507

Supplementary Figure 21. HPLC traces of *rac*-**3n** (reference) and enantioenriched-**3n**.

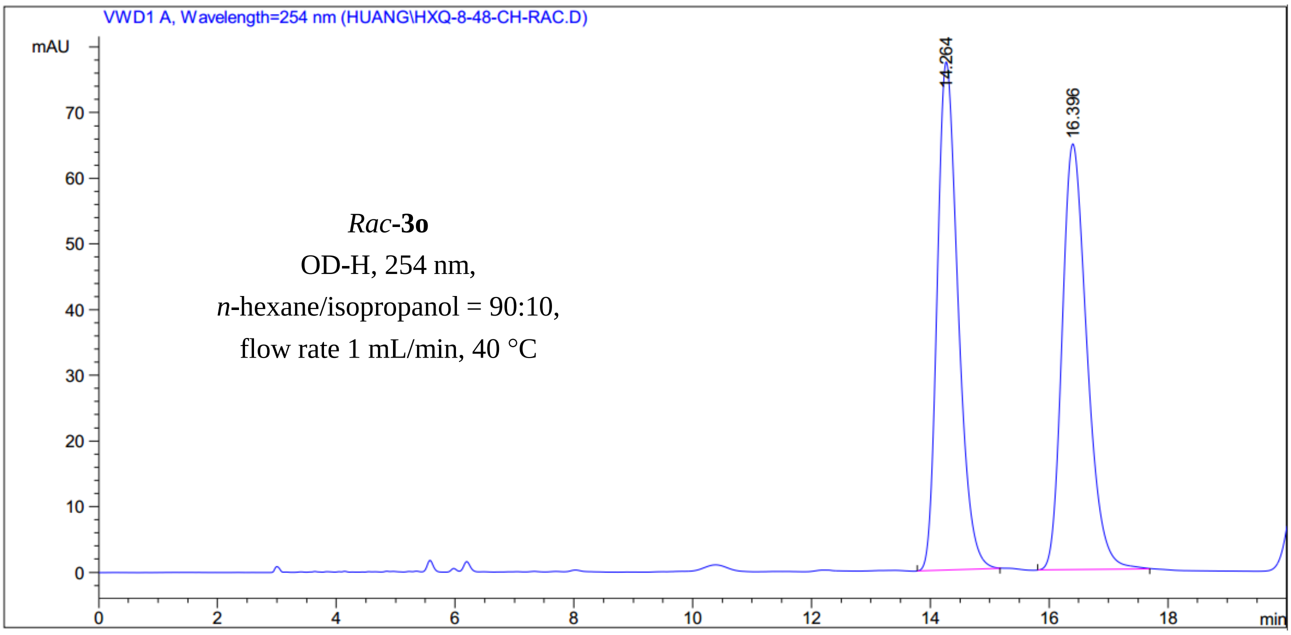

Peak #	RetTime [min]	Type	Width [min]	Area mAU *s	Height [mAU]	Area %
1	14.264	BB	0.3749	1888.00488	77.37908	50.0372
2	16.396	BB	0.4484	1885.19446	64.84287	49.9628

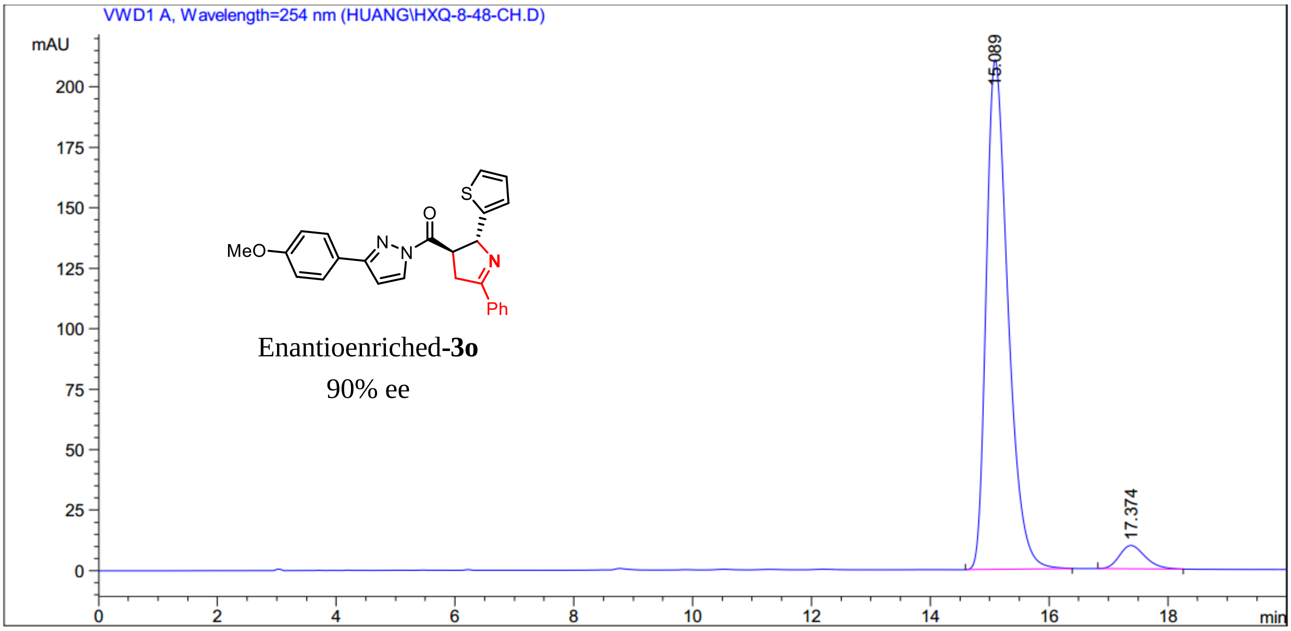

Peak #	RetTime [min]	Type	Width [min]	Area mAU *s	Height [mAU]	Area %
1	15.089	BB	0.3901	5360.51025	210.65033	94.8274
2	17.374	BB	0.4626	292.40430	9.65187	5.1726

Supplementary Figure 22. HPLC traces of *rac*-**3o** (reference) and enantioenriched-**3o**.

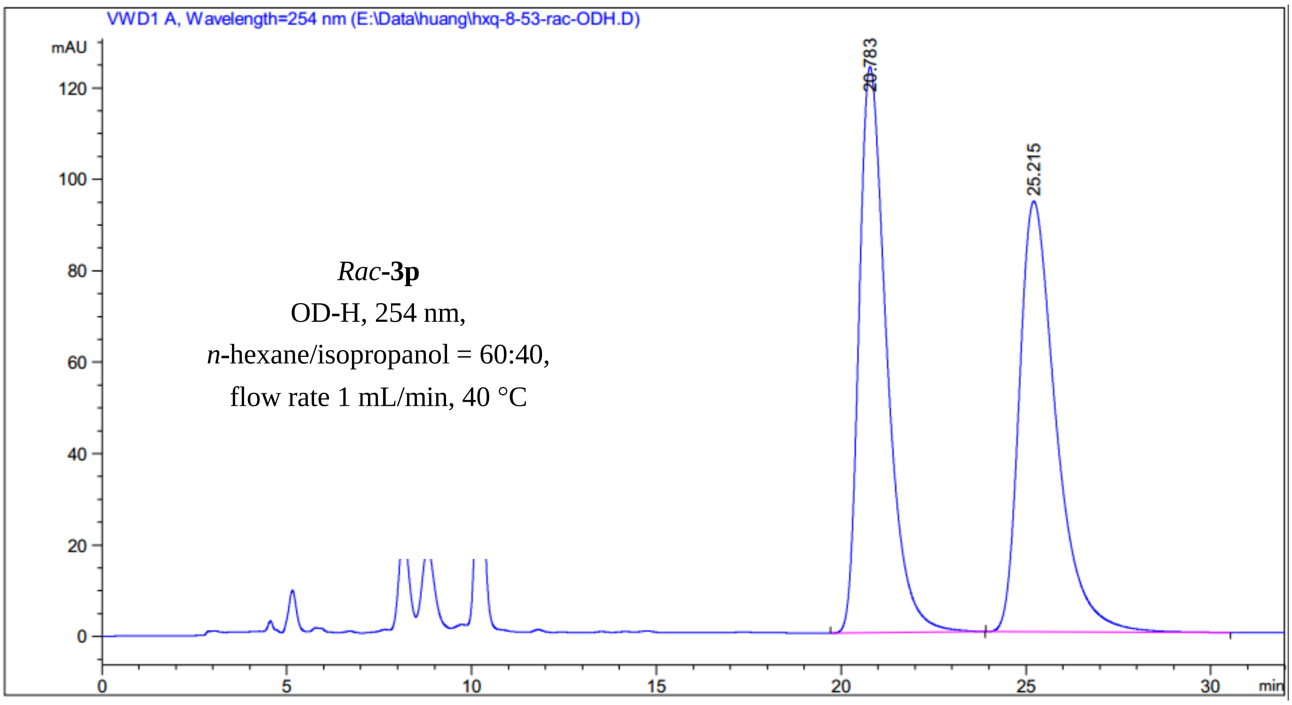

Peak #	RetTime [min]	Type	Width [min]	Area [mAU*s]	Height [mAU]	Area %
1	20.783	BB	0.7962	6466.20117	123.83692	49.9492
2	25.215	BB	1.0393	6479.36035	94.22034	50.0508

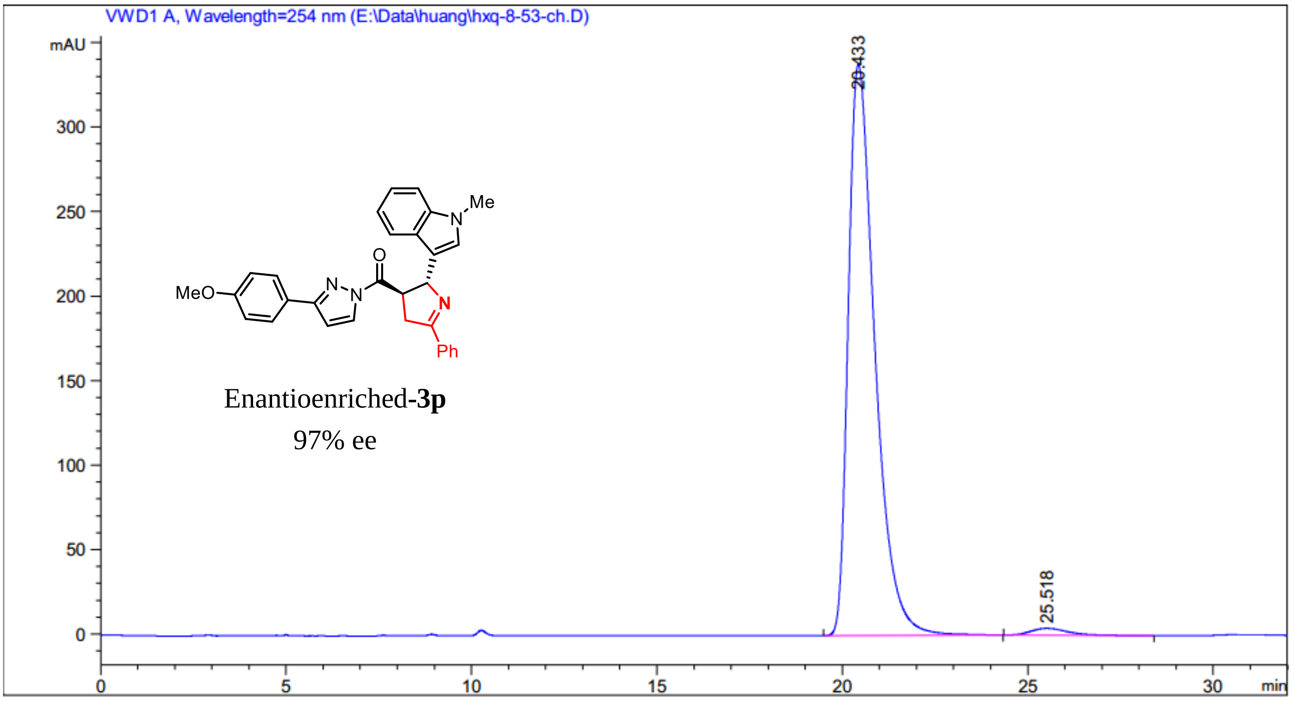

Peak #	RetTime [min]	Type	Width [min]	Area [mAU*s]	Height [mAU]	Area %
1	20.433	BB	0.7575	1.68459e4	337.96231	98.3145
2	25.518	BB	0.9935	288.80246	4.11164	1.6855

Supplementary Figure 23. HPLC traces of *rac*-**3p** (reference) and enantioenriched-**3p**.

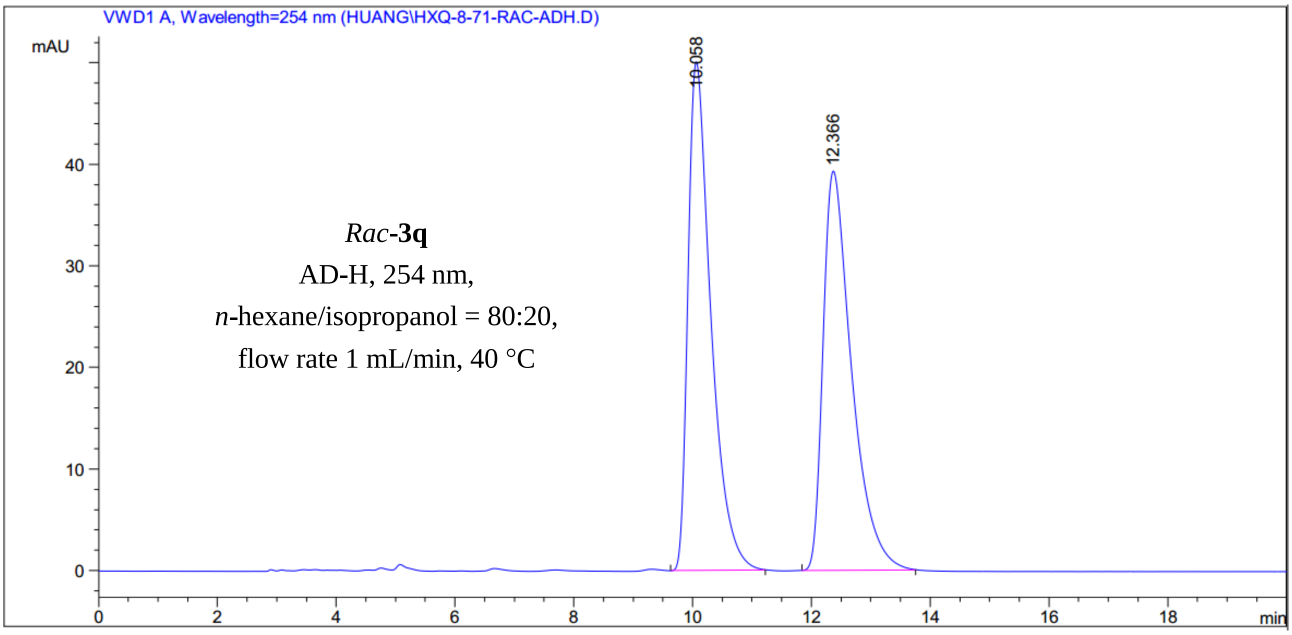

Peak #	RetTime [min]	Type	Width [min]	Area mAU *s	Height [mAU]	Area %
1	10.058	BB	0.4013	1342.13489	50.11173	50.5007
2	12.366	BB	0.5010	1315.52100	39.31803	49.4993

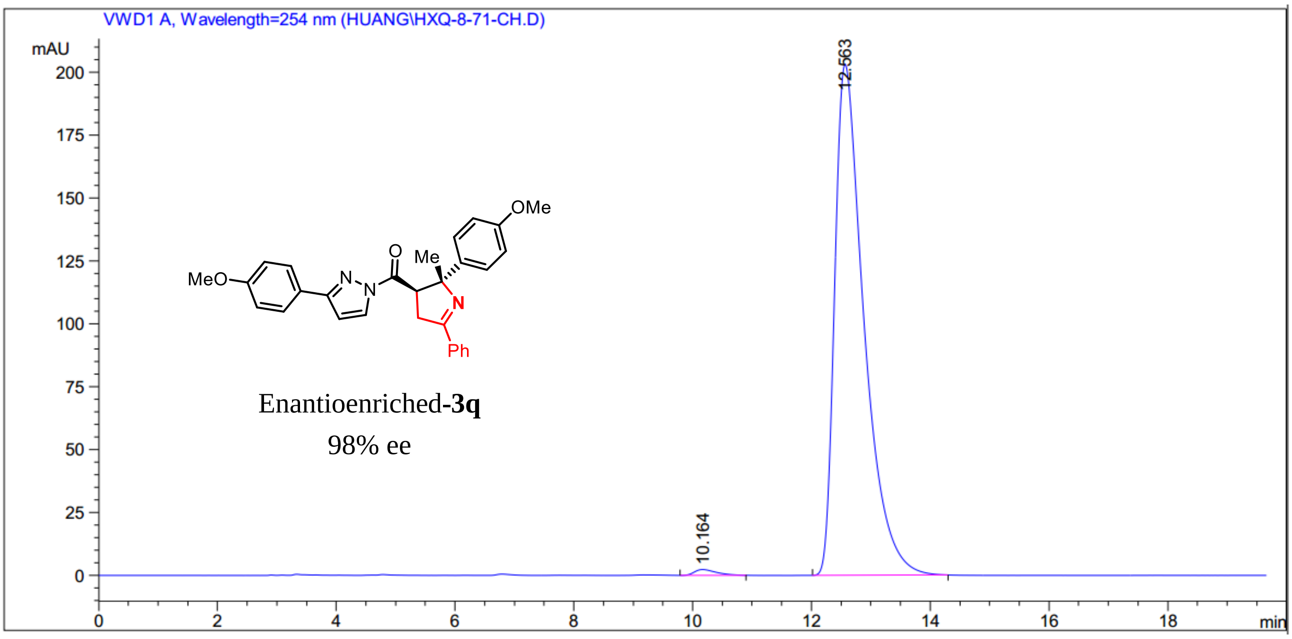

Peak #	RetTime [min]	Type	Width [min]	Area mAU *s	Height [mAU]	Area %
1	10.164	BB	0.3872	60.44700	2.32935	0.8632
2	12.563	BB	0.5119	6942.58838	203.32722	99.1368

Supplementary Figure 24. HPLC traces of *rac*-**3q** (reference) and enantioenriched-**3q**.

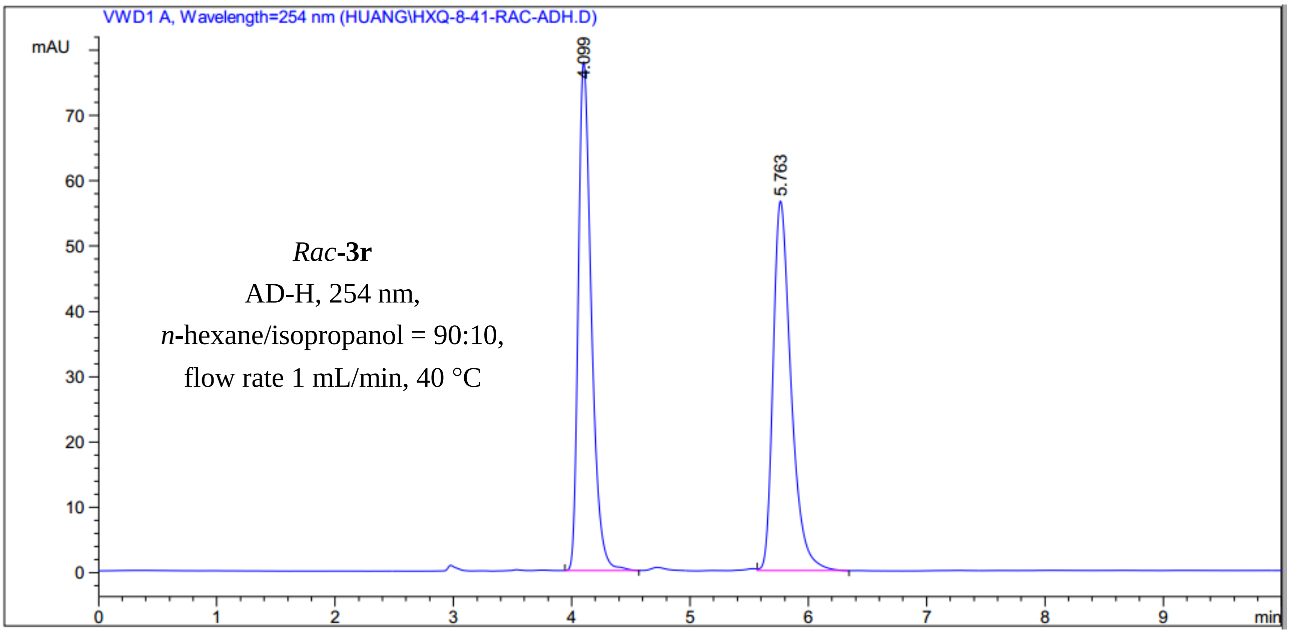

Peak #	RetTime [min]	Type	Width [min]	Area mAU *s	Height [mAU]	Area %
1	4.099	BB	0.1172	597.67120	77.91147	50.0055
2	5.763	VB	0.1611	597.53925	56.56620	49.9945

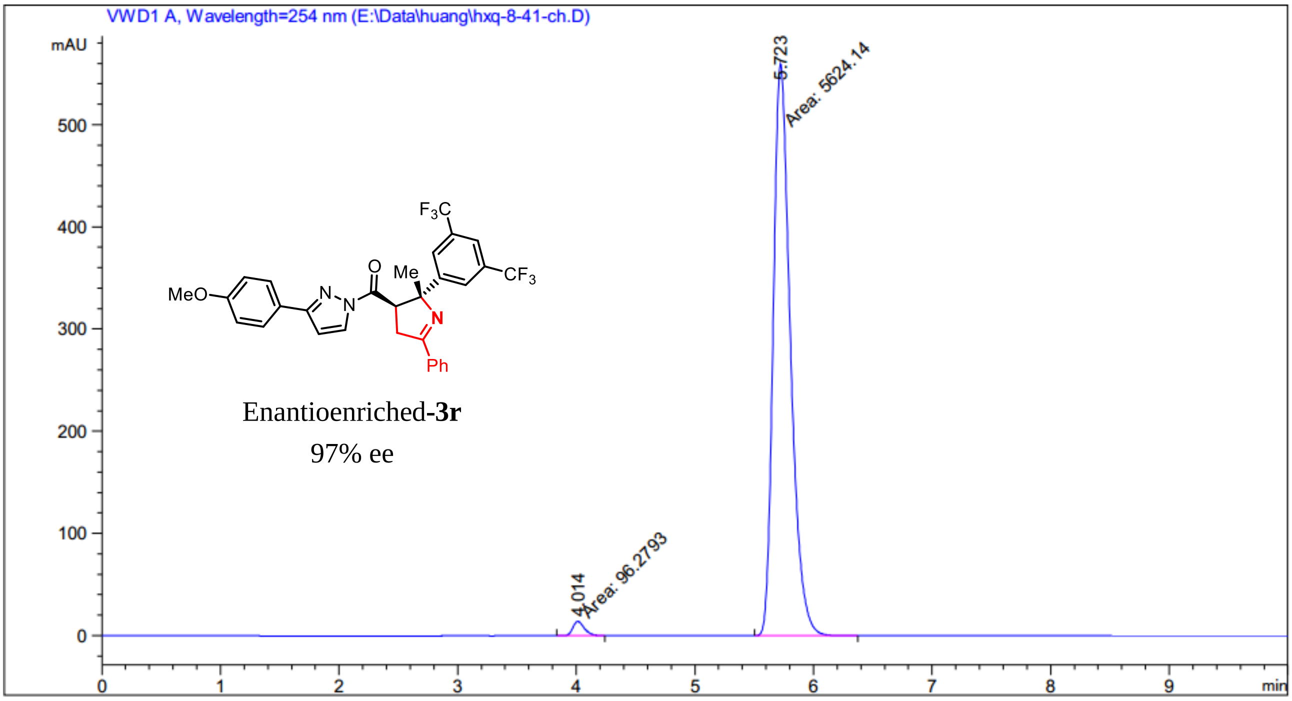

Peak #	RetTime [min]	Type	Width [min]	Area [mAU*s]	Height [mAU]	Area %
1	4.014	MF	0.1137	96.27927	14.11182	1.6831
2	5.723	FM	0.1675	5624.14453	559.55475	98.3169

Supplementary Figure 25. HPLC traces of *rac*-**3r** (reference) and Enantioenriched-**3r**.

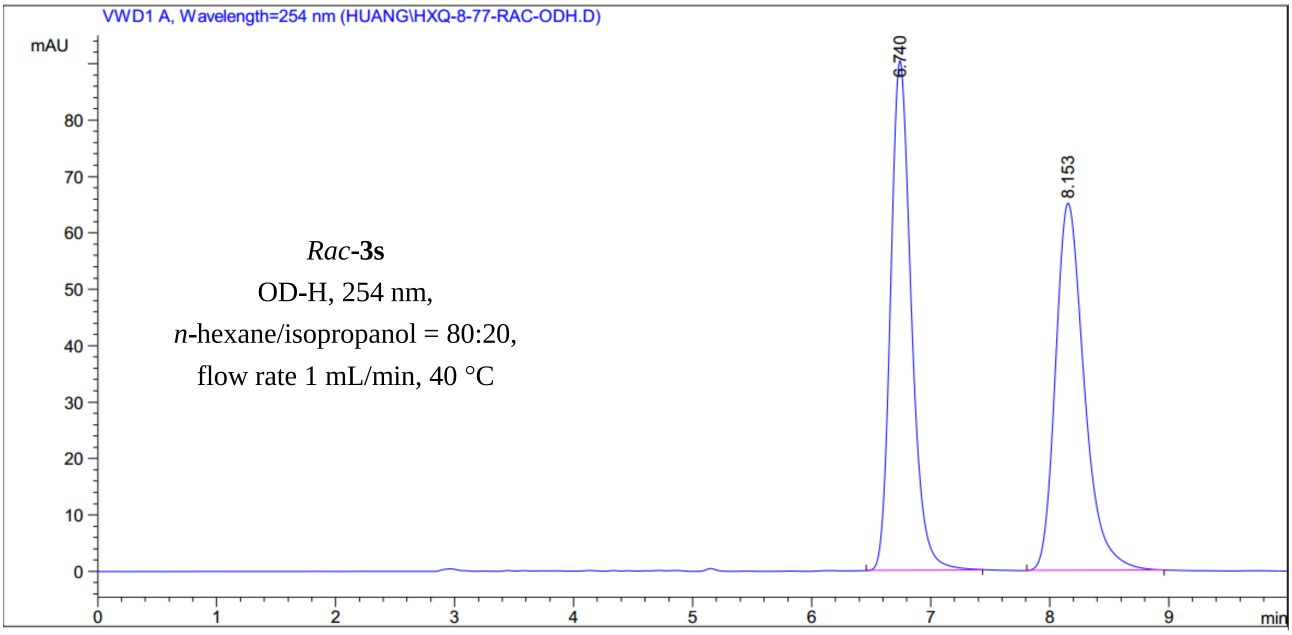

Peak #	RetTime [min]	Type	Width [min]	Area mAU *s	Height [mAU]	Area %
1	6.740	BB	0.1851	1094.99817	90.36444	50.0006
2	8.153	BB	0.2574	1094.97290	65.08418	49.9994

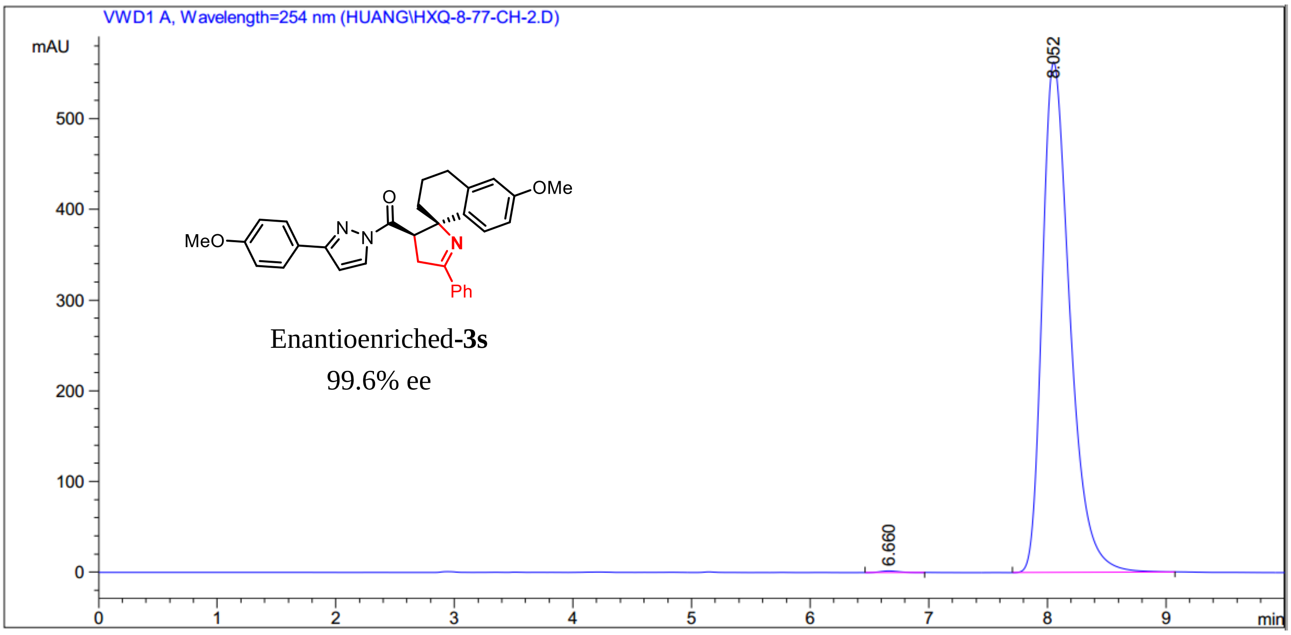

Peak #	RetTime [min]	Type	Width [min]	Area mAU *s	Height [mAU]	Area %
1	6.660	BB	0.1722	19.84501	1.76095	0.2172
2	8.052	BB	0.2472	9116.07910	562.96997	99.7828

Supplementary Figure 26. HPLC traces of *rac*-**3s** (reference) and enantioenriched-**3s**.

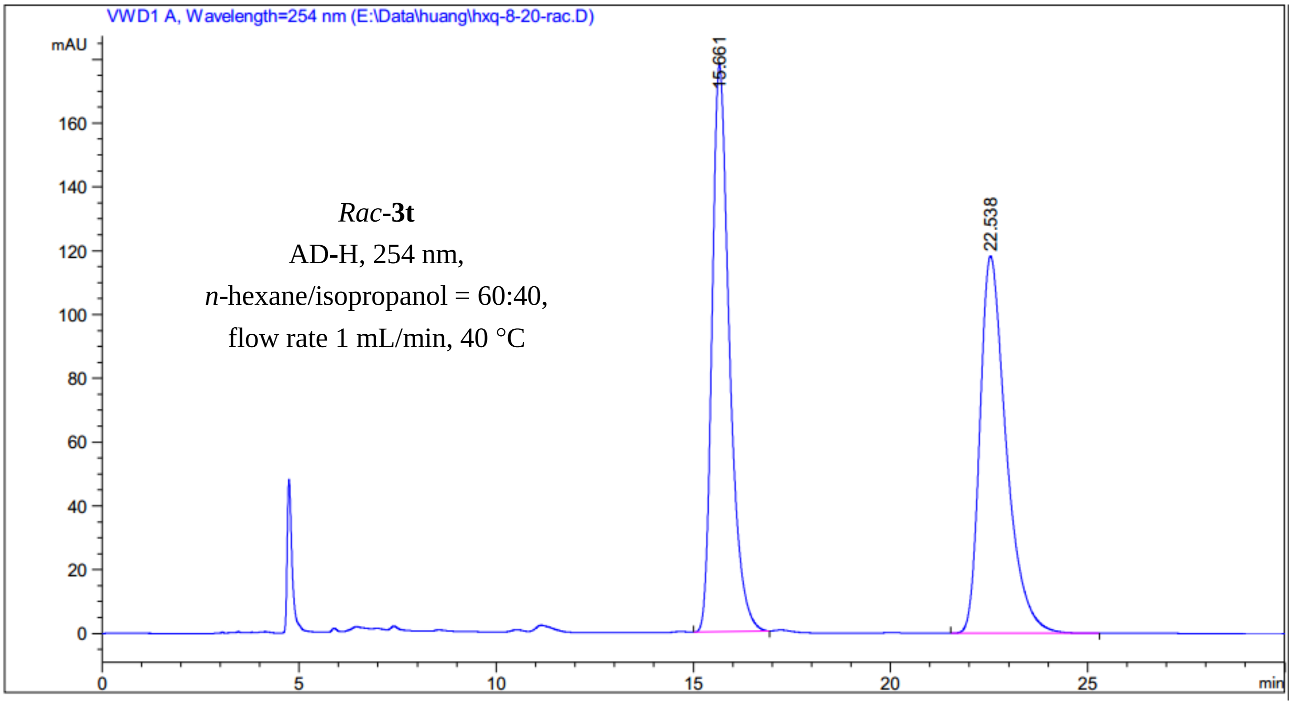

Peak #	RetTime [min]	Type	Width [min]	Area [mAU*s]	Height [mAU]	Area %
1	15.661	BB	0.4641	5463.56689	177.99493	49.9402
2	22.538	BB	0.6973	5476.64795	118.25566	50.0598

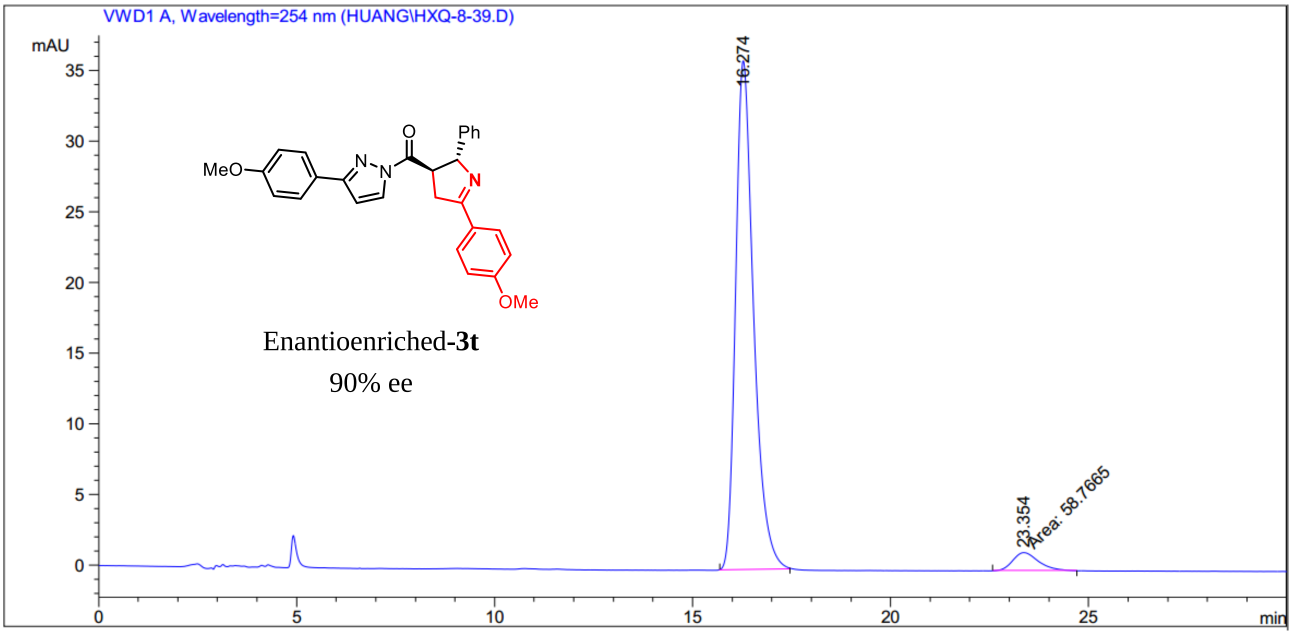

Peak #	RetTime [min]	Type	Width [min]	Area mAU *s	Height [mAU]	Area %
1	16.274	BB	0.4798	1138.97900	35.99582	95.0936
2	23.354	MM	0.7629	58.76653	1.28377	4.9064

Supplementary Figure 27. HPLC traces of *rac*-**3t** (reference) and enantioenriched-**3t**.

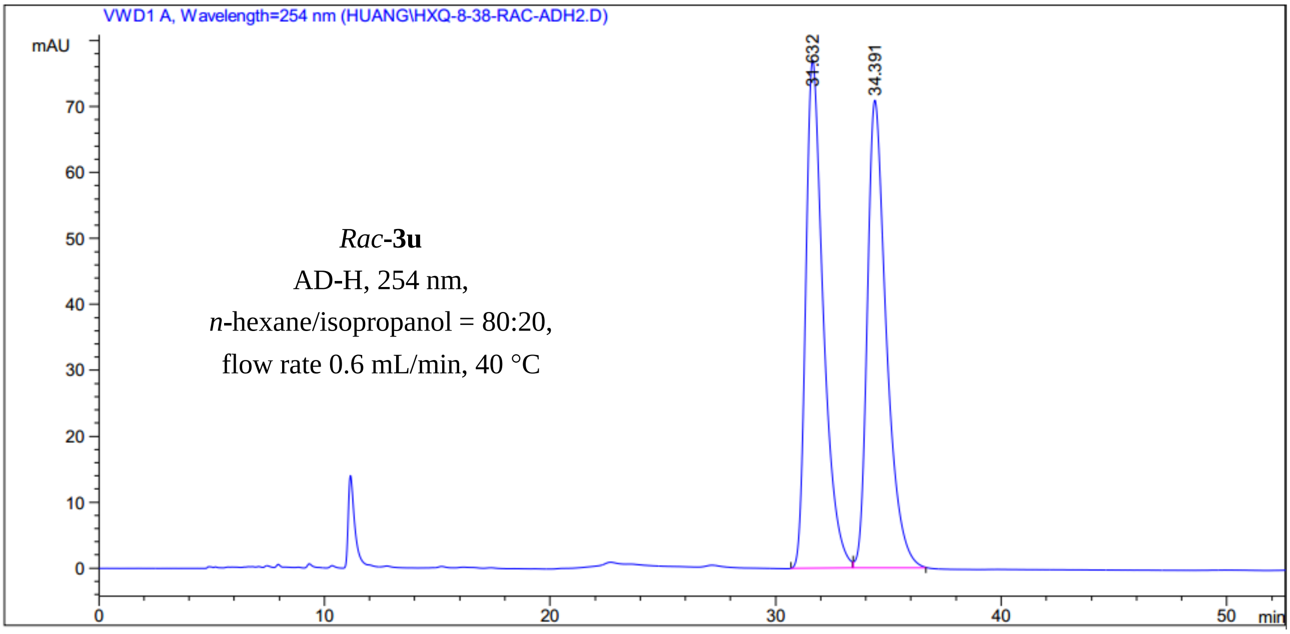

Peak #	RetTime [min]	Type	Width [min]	Area mAU *s	Height [mAU]	Area %
1	31.632	BB	0.8097	4175.02051	77.06068	49.9920
2	34.391	BB	0.8803	4176.34961	70.96404	50.0080

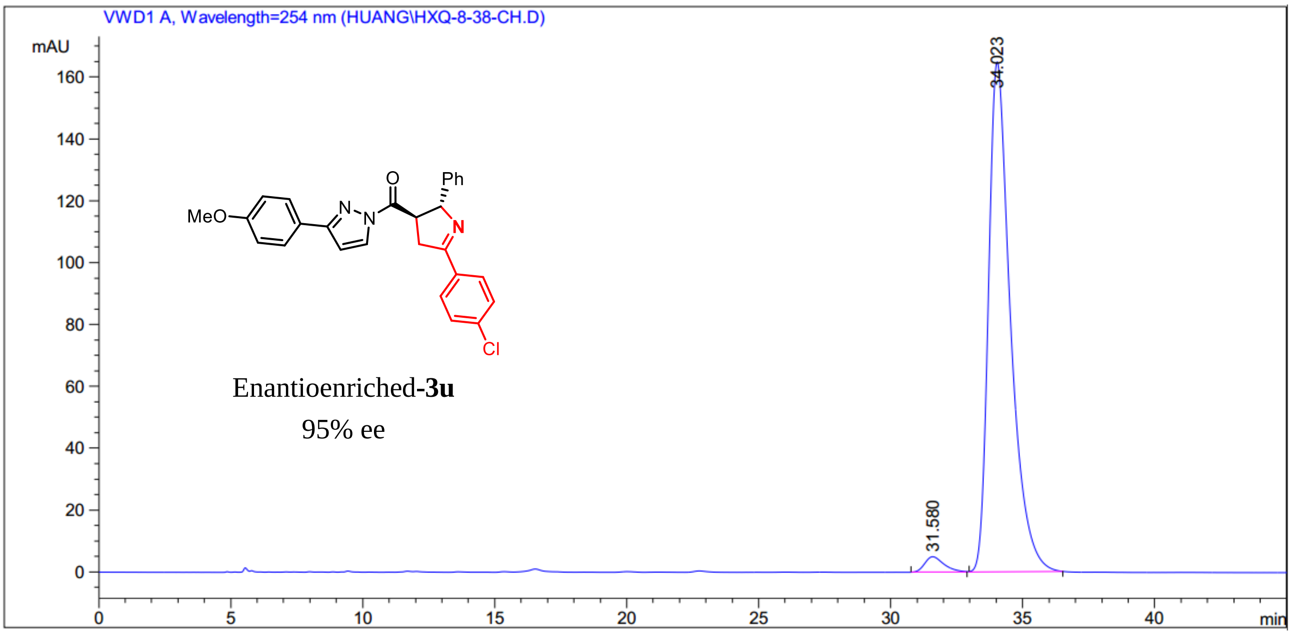

Peak #	RetTime [min]	Type	Width [min]	Area mAU *s	Height [mAU]	Area %
1	31.580	BB	0.7443	254.94449	5.00463	2.6071
2	34.023	BB	0.8675	9524.09180	164.88298	97.3929

Supplementary Figure 28. HPLC traces of *rac*-**3u** (reference) and enantioenriched-**3u**.

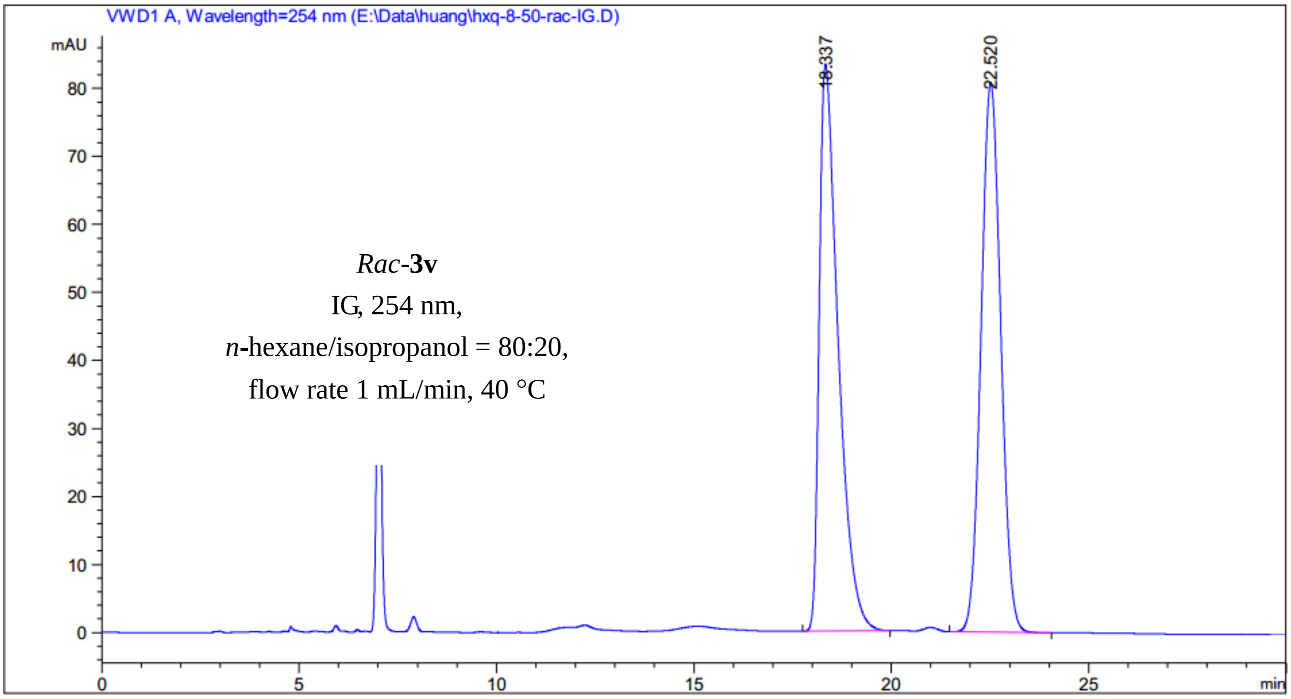

Peak #	RetTime [min]	Type	Width [min]	Area [mAU*s]	Height [mAU]	Area %
1	18.337	BB	0.4980	2787.35498	83.38284	49.8841
2	22.520	BB	0.5403	2800.30225	80.80841	50.1159

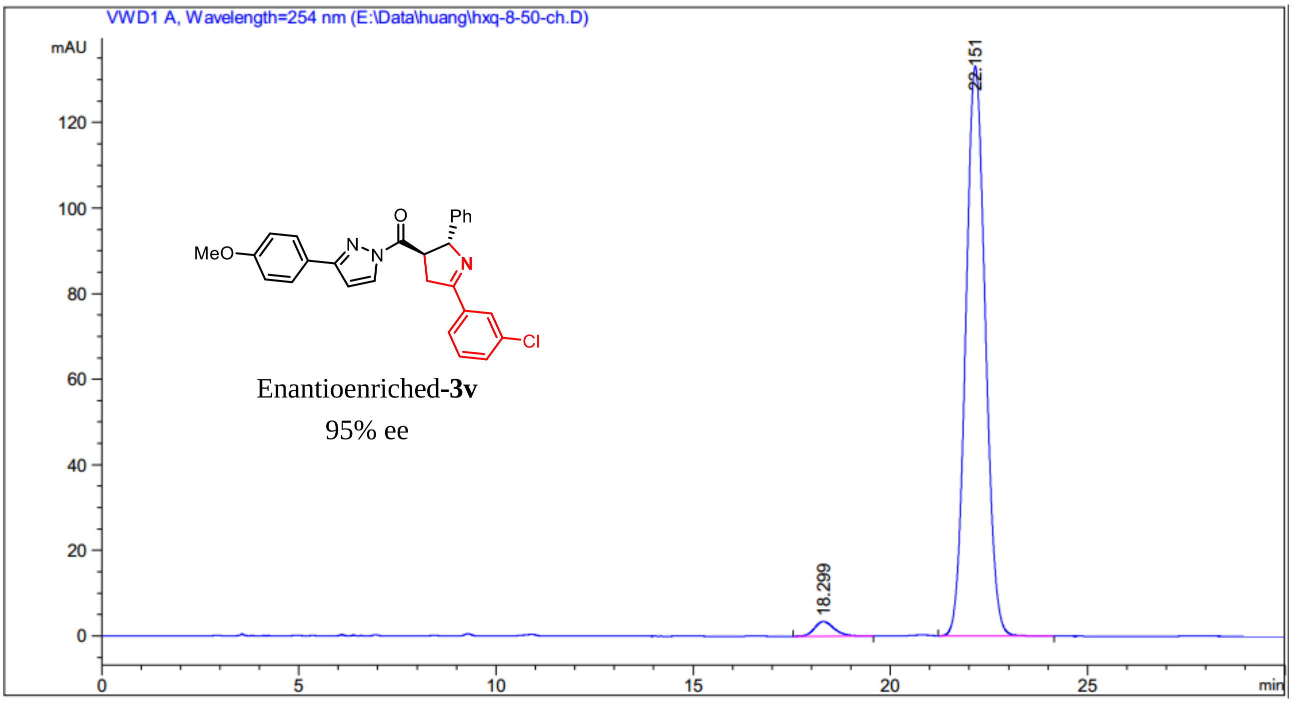

Peak #	RetTime [min]	Type	Width [min]	Area [mAU*s]	Height [mAU]	Area %
1	18.299	BB	0.5246	120.09737	3.44581	2.5526
2	22.151	BB	0.5378	4584.85205	133.11864	97.4474

Supplementary Figure 29. HPLC traces of *rac*-**3v** (reference) and enantioenriched-**3v**.

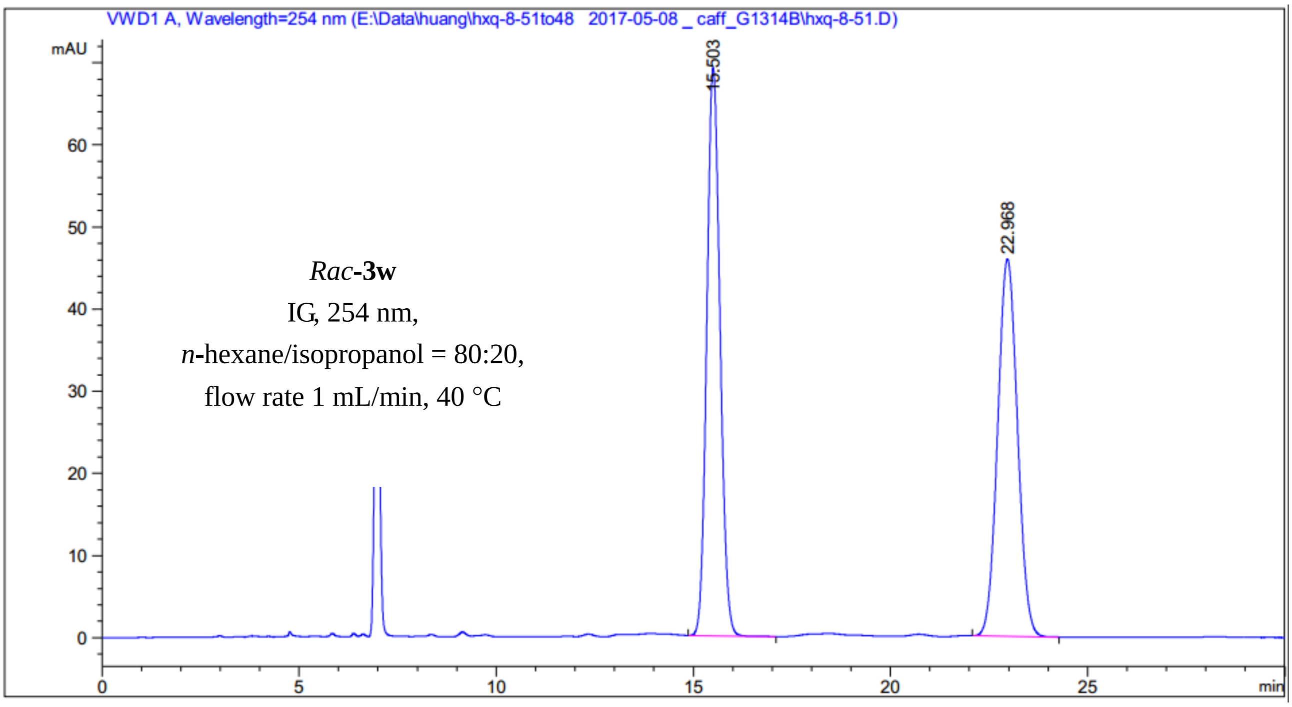

Peak #	RetTime [min]	Type	Width [min]	Area [mAU*s]	Height [mAU]	Area %
1	15.503	BB	0.3628	1610.92224	69.15440	50.1156
2	22.968	BB	0.5463	1603.49255	45.93108	49.8844

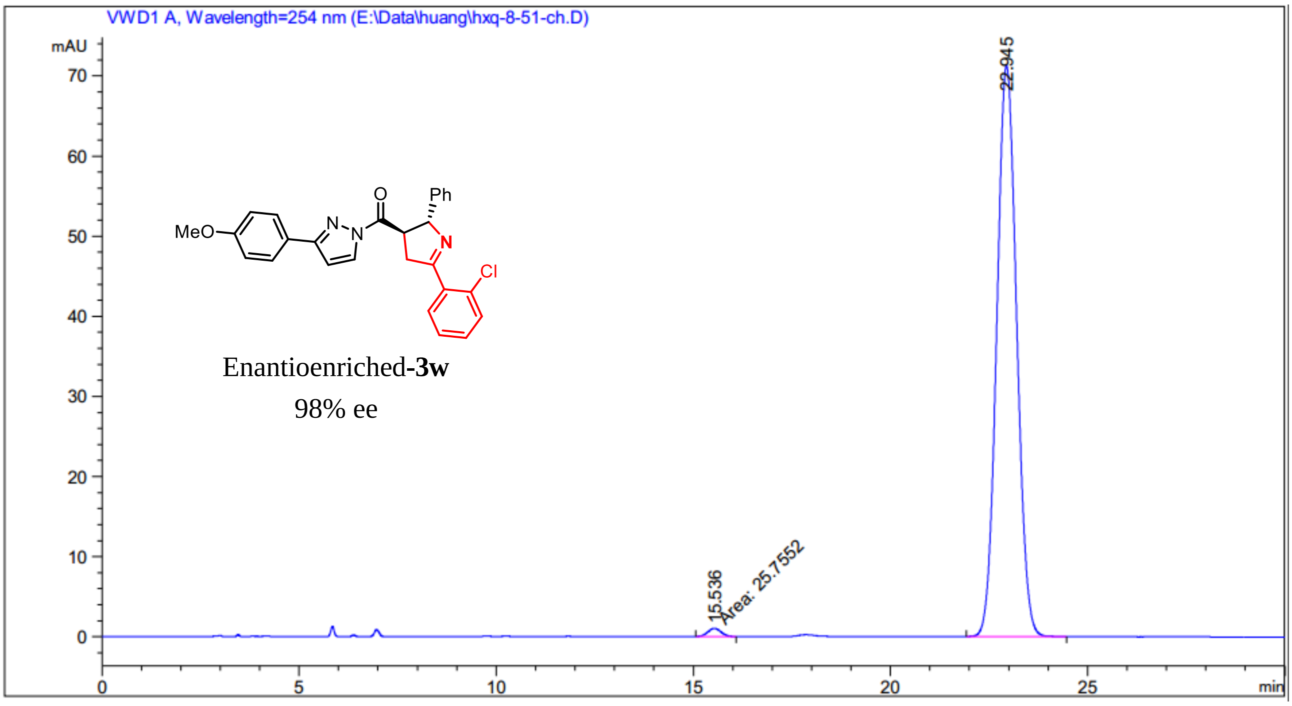

Peak #	RetTime [min]	Type	Width [min]	Area [mAU*s]	Height [mAU]	Area %
1	15.536	MM	0.4082	25.75523	1.05163	1.0231
2	22.945	BB	0.5470	2491.65576	71.25217	98.9769

Supplementary Figure 30. HPLC traces of *rac*-**3w** (reference) and enantioenriched-**3w**.

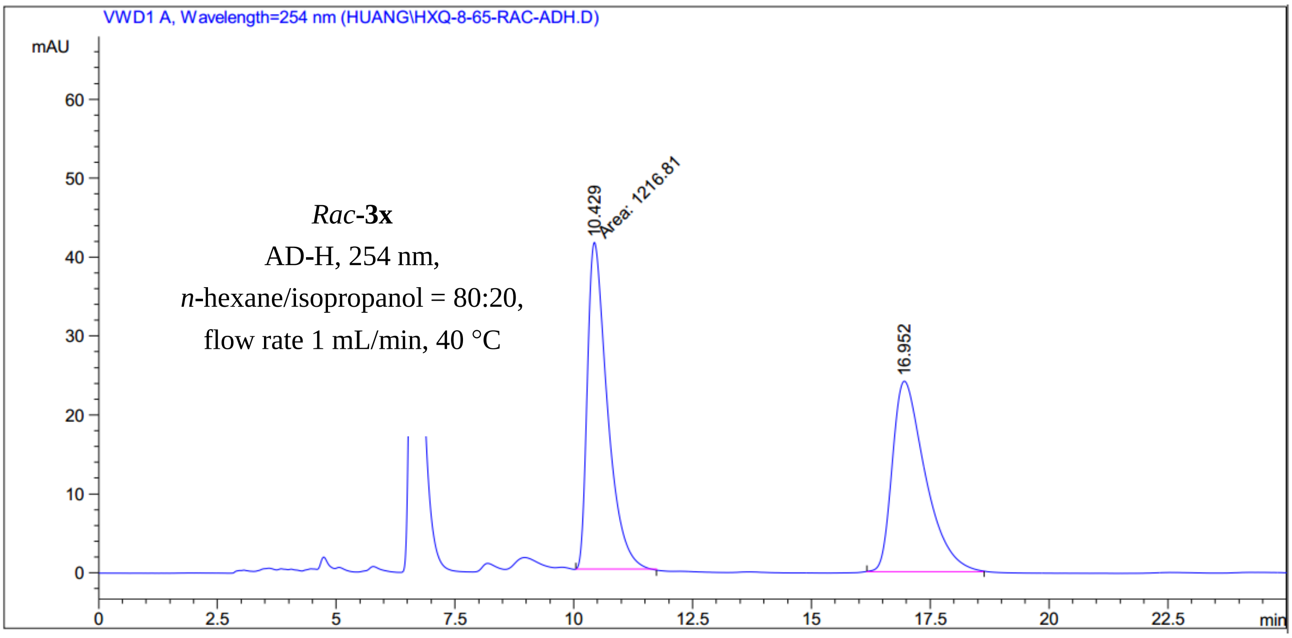

Peak #	RetTime [min]	Type	Width [min]	Area mAU *s	Height [mAU]	Area %
1	10.429	MM	0.4894	1216.81238	41.43752	50.9646
2	16.952	BB	0.7208	1170.75208	24.17505	49.0354

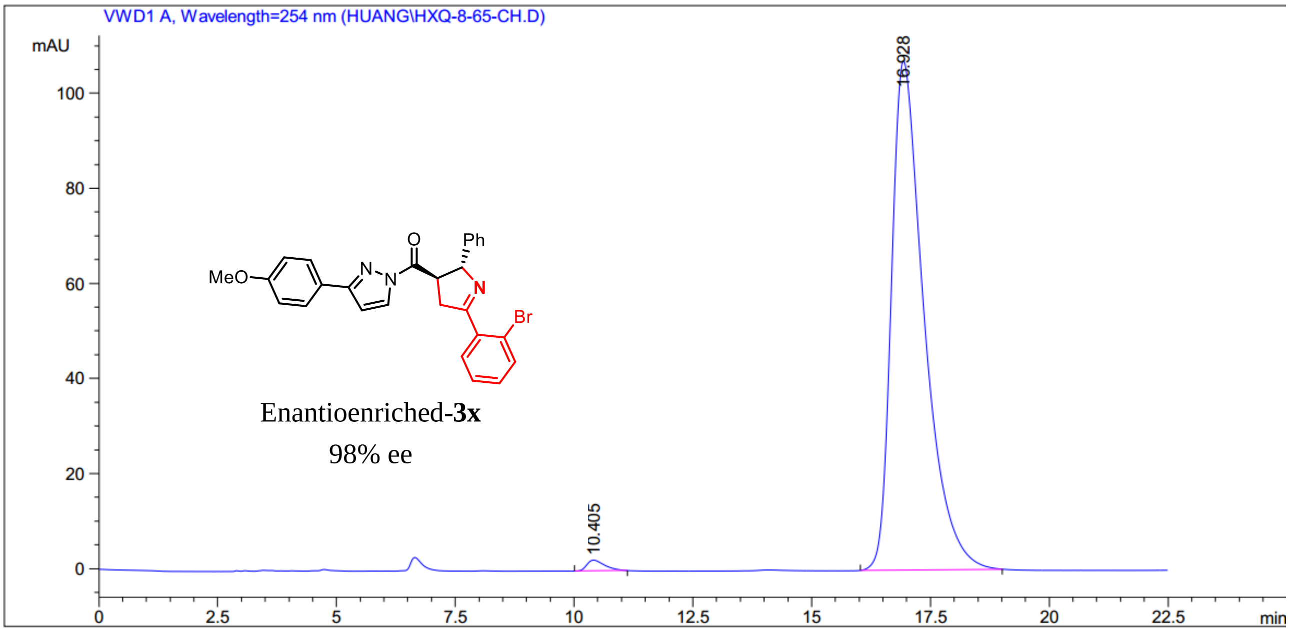

Peak #	RetTime [min]	Type	Width [min]	Area mAU *s	Height [mAU]	Area %
1	10.405	BB	0.3920	60.87057	2.28747	1.1723
2	16.928	BB	0.7146	5131.43945	107.13801	98.8277

Supplementary Figure 31. HPLC traces of *rac*-**3x** (reference) and enantioenriched-**3x**.

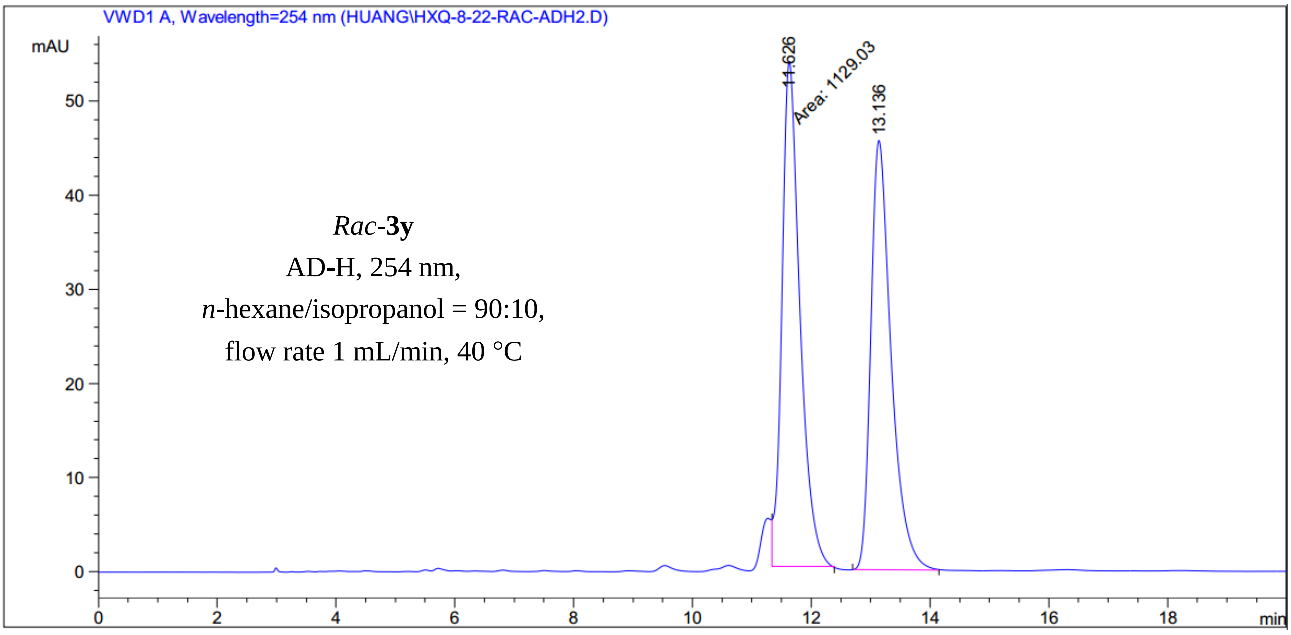

Peak #	RetTime [min]	Type	Width [min]	Area mAU *s	Height [mAU]	Area %
1	11.626	FM	0.3507	1129.03125	53.66072	50.9556
2	13.136	BB	0.3566	1086.68445	45.61584	49.0444

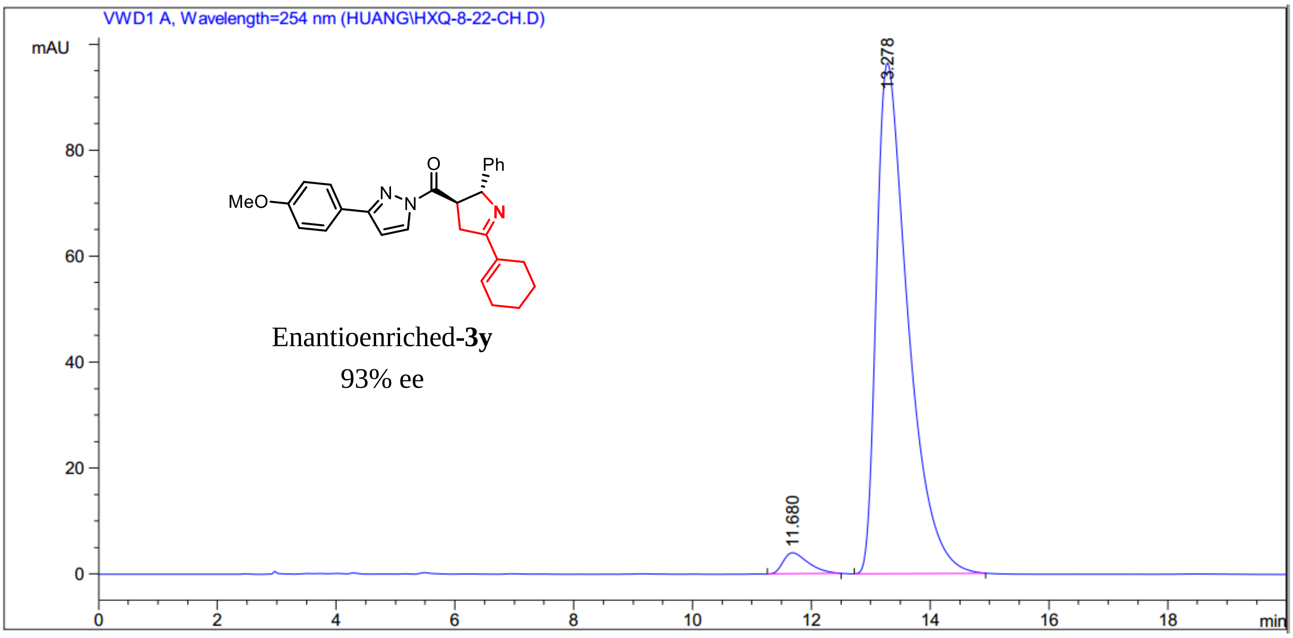

Peak #	RetTime [min]	Type	Width [min]	Area mAU *s	Height [mAU]	Area %
1	11.680	BB	0.4449	119.54857	3.99809	3.2504
2	13.278	BB	0.5545	3558.36353	96.38797	96.7496

Supplementary Figure 32. HPLC traces of *rac*-**3y** (reference) and enantioenriched-**3y**.

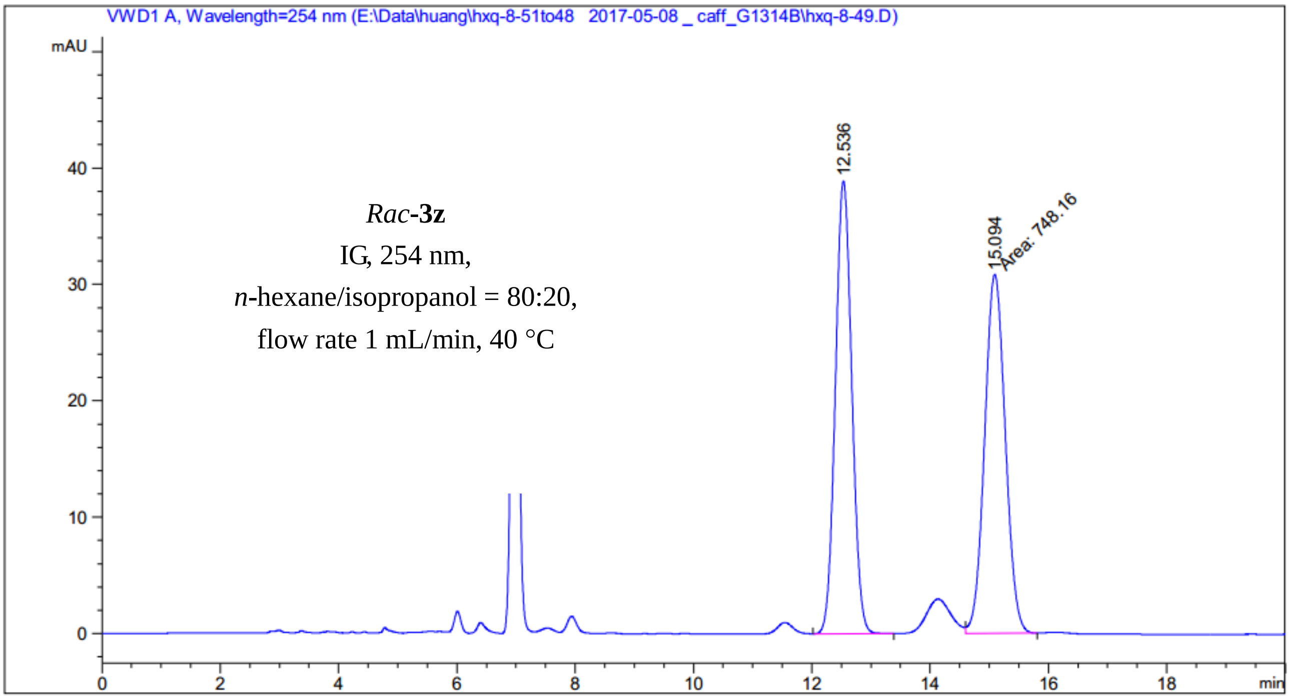

Peak #	RetTime [min]	Type	Width [min]	Area [mAU*s]	Height [mAU]	Area %
1	12.536	BB	0.3008	749.73889	38.90879	50.0527
2	15.094	FM	0.4044	748.15997	30.83371	49.9473

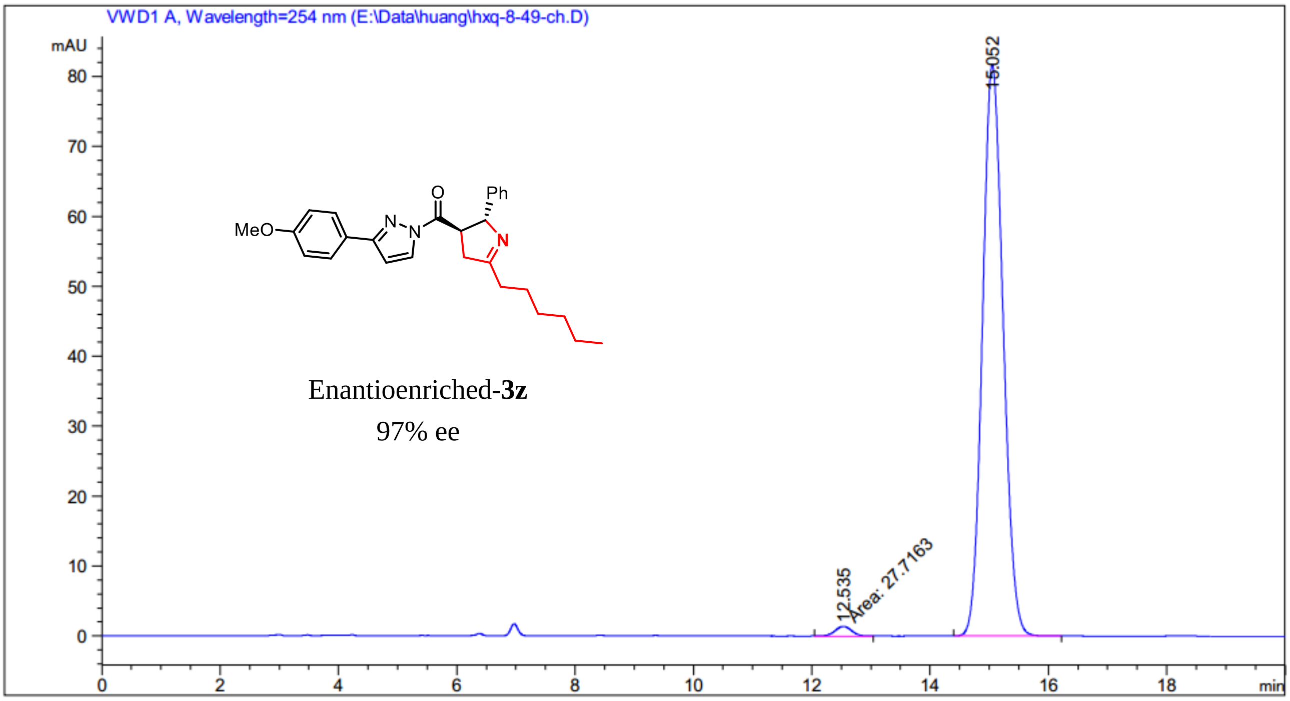

Peak #	RetTime [min]	Type	Width [min]	Area [mAU*s]	Height [mAU]	Area %
1	12.535	MM	0.3289	27.71632	1.40450	1.3816
2	15.052	BB	0.3776	1978.38293	81.68462	98.6184

Supplementary Figure 33. HPLC traces of *rac*-**3z** (reference) and enantioenriched-**3z**.

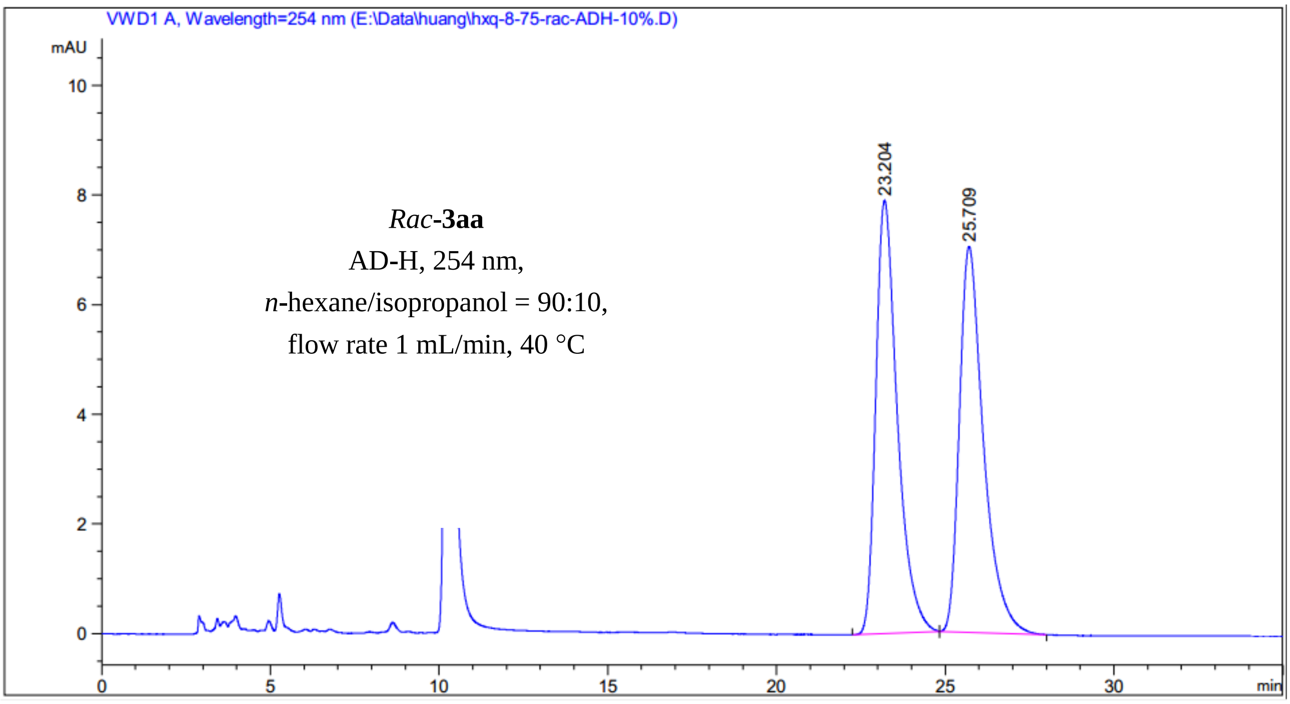

Peak #	RetTime [min]	Type	Width [min]	Area mAU *s	Height [mAU]	Area %
1	11.626	FM	0.3507	1129.03125	53.66072	50.9556
2	13.136	BB	0.3566	1086.68445	45.61584	49.0444

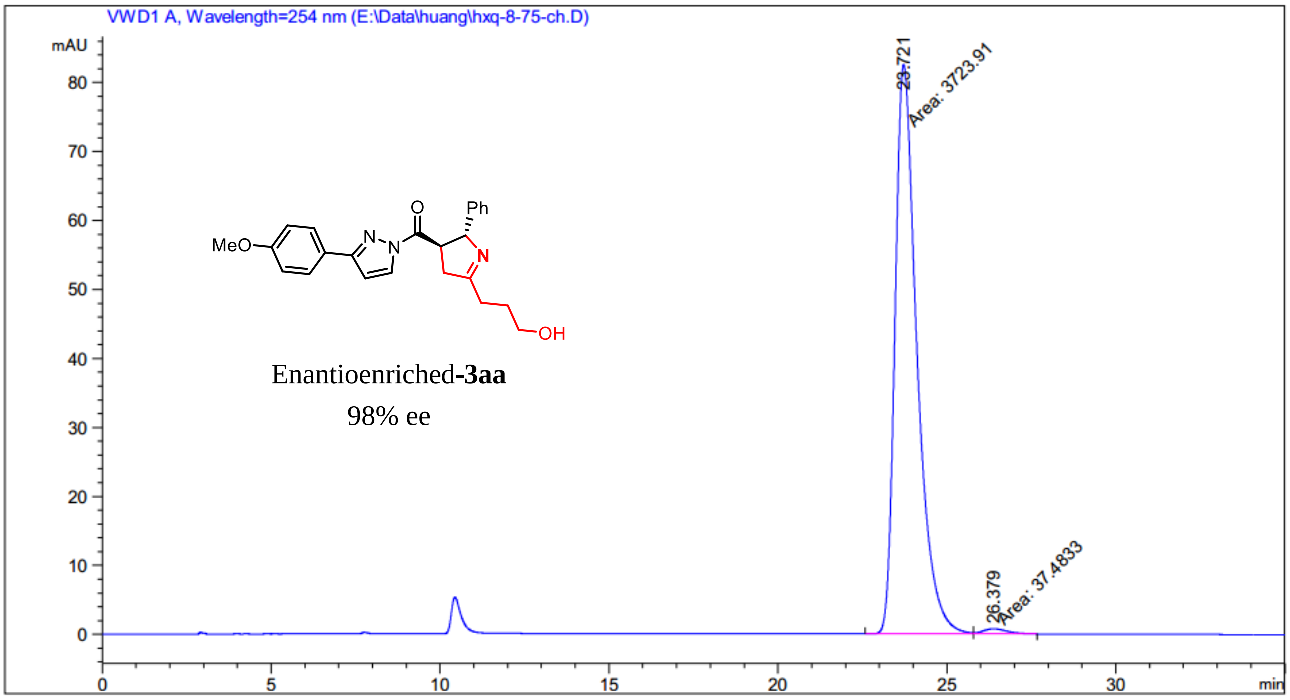

Peak #	RetTime [min]	Type	Width [min]	Area [mAU*s]	Height [mAU]	Area %
1	23.721	MF	0.7521	3723.91406	82.51953	99.0035
2	26.379	FM	0.8223	37.48328	7.59710e-1	0.9965

Supplementary Figure 34. HPLC traces of *rac*-**3aa** (reference) and enantioenriched-**3aa**.

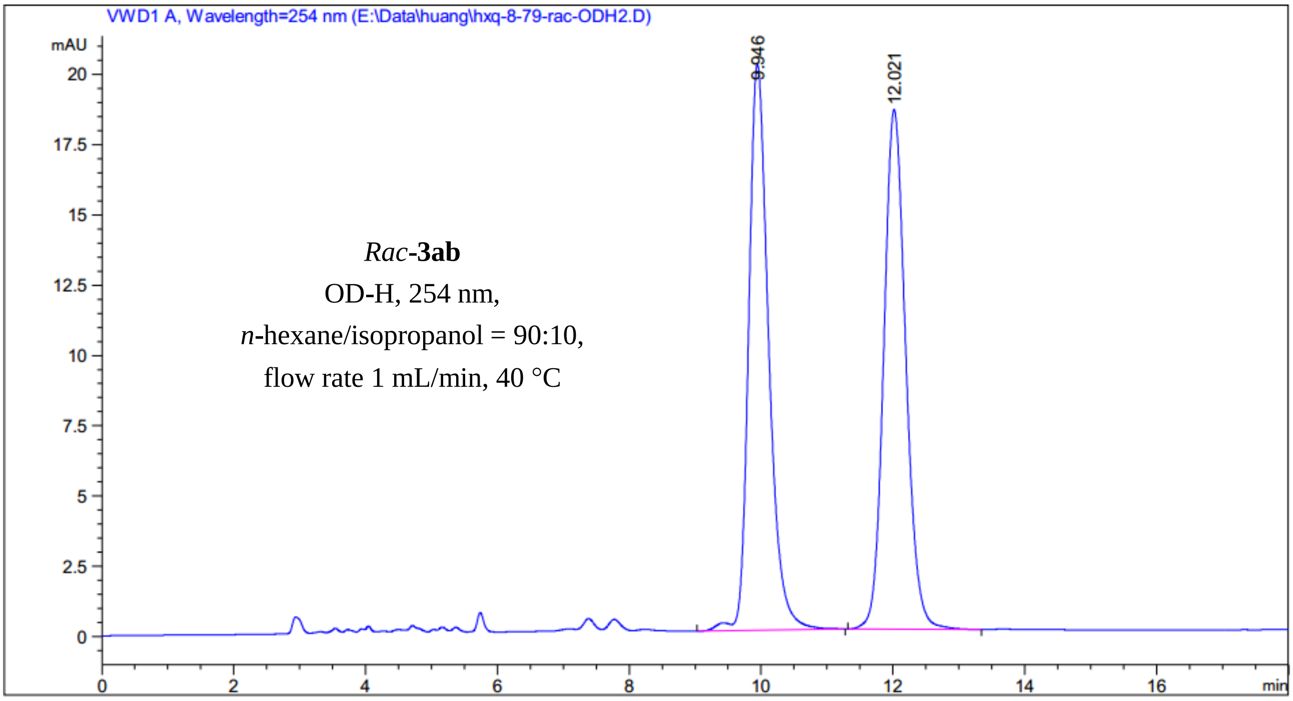

Peak #	RetTime [min]	Type	Width [min]	Area [mAU*s]	Height [mAU]	Area %
1	9.946	BB	0.3233	427.16196	20.15332	49.9033
2	12.021	BB	0.3574	428.81784	18.49925	50.0967

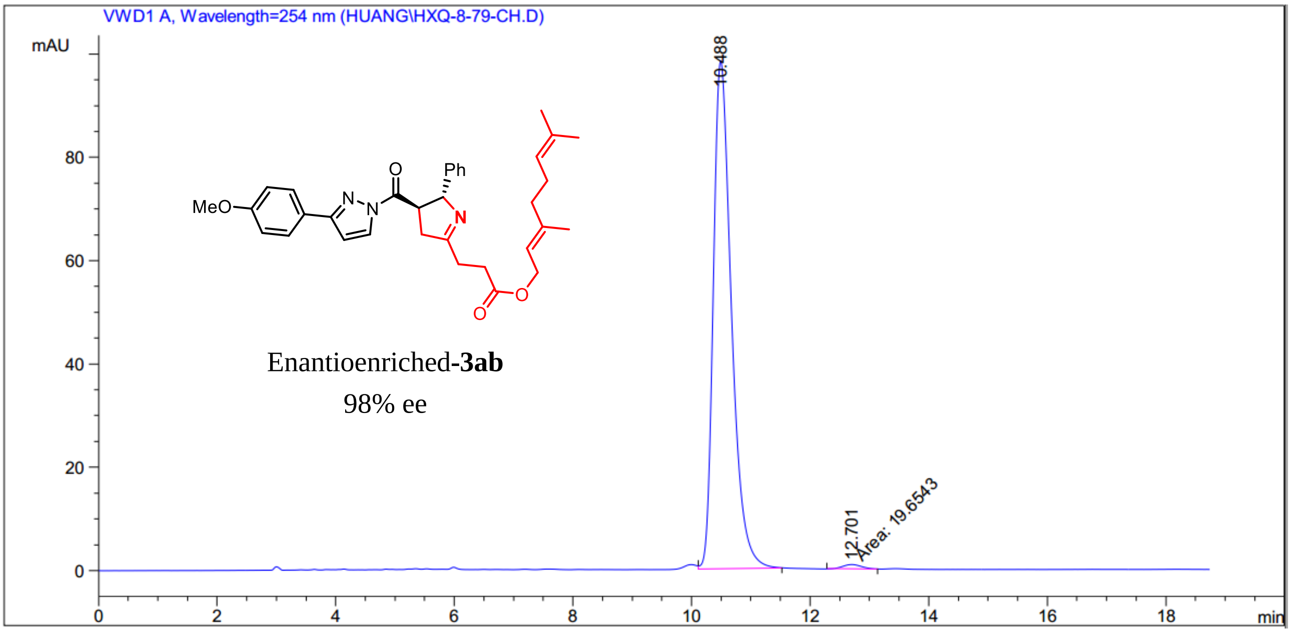

Peak #	RetTime [min]	Type	Width [min]	Area mAU *s	Height [mAU]	Area %
1	10.488	VB	0.3228	2083.80640	98.37585	99.0656
2	12.701	MM	0.3761	19.65430	8.70940e-1	0.9344

Supplementary Figure 35. HPLC traces of *rac*-**3ab** (reference) and enantioenriched-**3ab**.

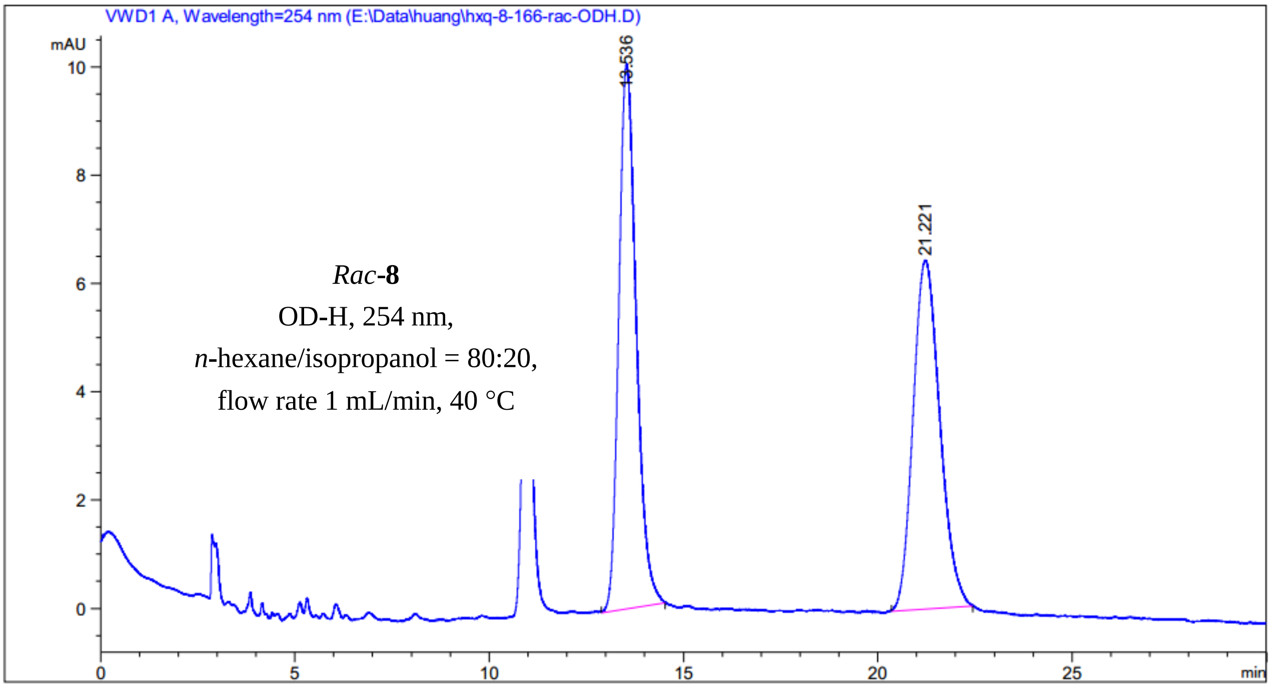

Peak #	RetTime [min]	Type	Width [min]	Area [mAU*s]	Height [mAU]	Area %
1	13.536	BV R	0.3667	314.13849	10.06380	50.5030
2	21.221	BB	0.5591	307.88120	6.44167	49.4970

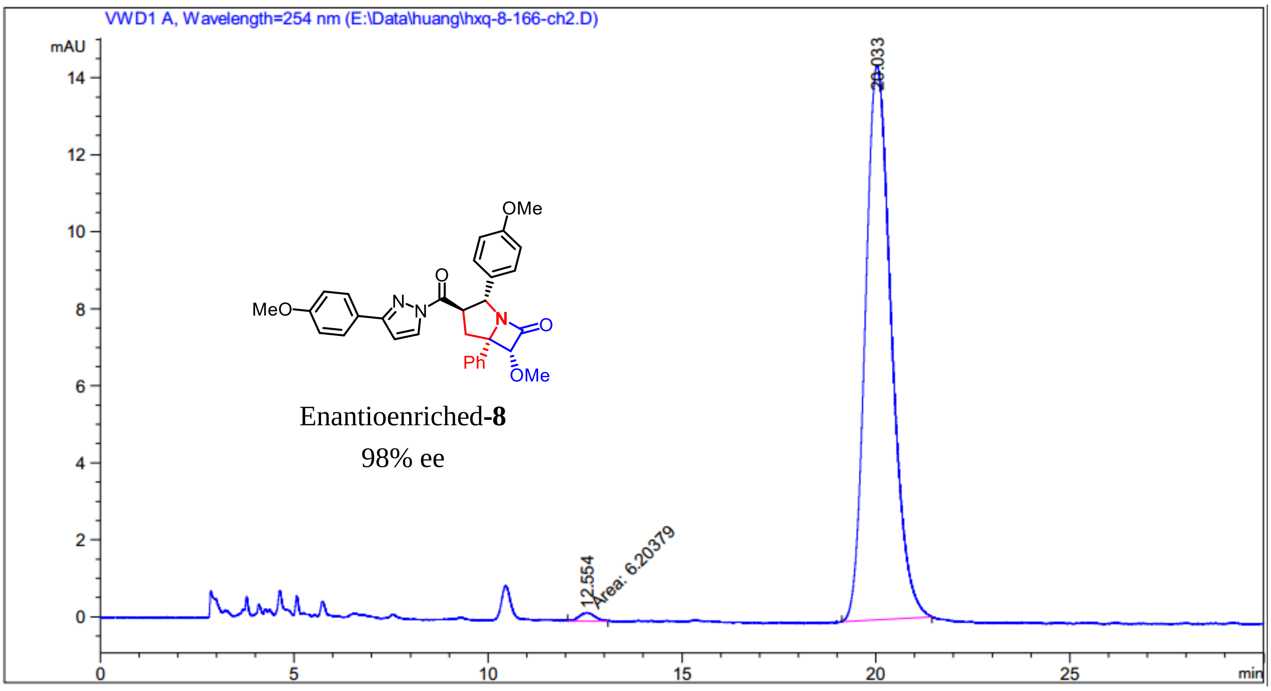

Peak #	RetTime [min]	Type	Width [min]	Area [mAU*s]	Height [mAU]	Area %
1	12.554	MM	0.4564	6.20379	2.26543e-1	0.9189
2	20.033	BB	0.5457	668.95770	14.39492	99.0811

Supplementary Figure 36. HPLC traces of *rac*-**8** (reference) and enantioenriched-**8**.

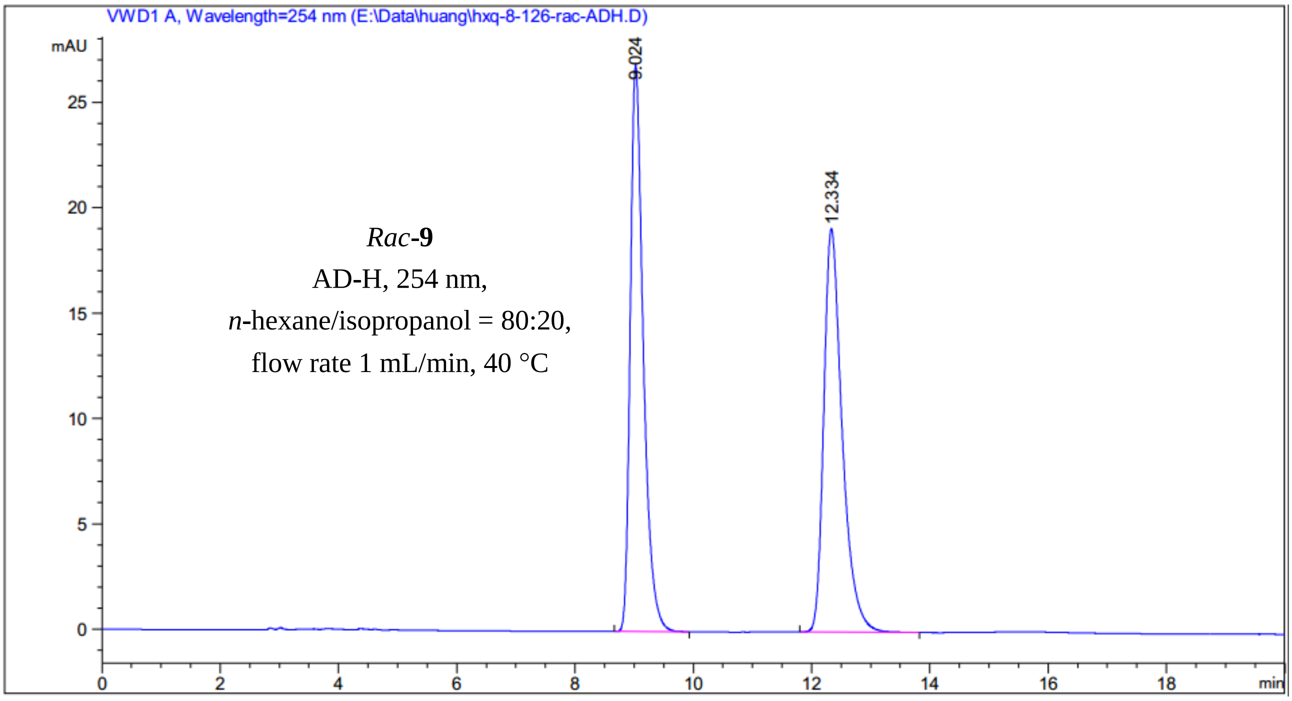

Peak #	RetTime [min]	Type	Width [min]	Area [mAU*s]	Height [mAU]	Area %
1	9.024	BB	0.2307	411.81540	26.89006	49.8844
2	12.334	BB	0.3252	413.72391	19.13801	50.1156

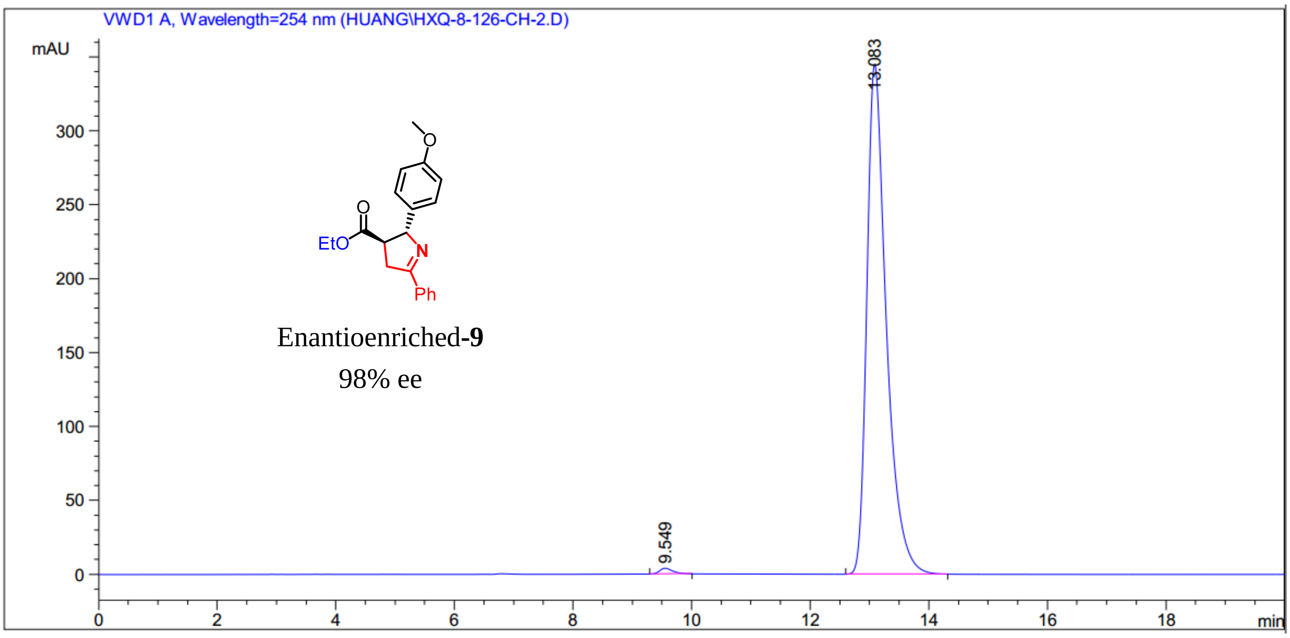

Peak #	RetTime [min]	Type	Width [min]	Area mAU *s	Height [mAU]	Area %
1	9.549	BB	0.2362	60.98370	3.87052	0.7677
2	13.083	BB	0.3407	7882.19824	345.13055	99.2323

Supplementary Figure 37. HPLC traces of *rac*-**9** (reference) and enantioenriched-**9**.

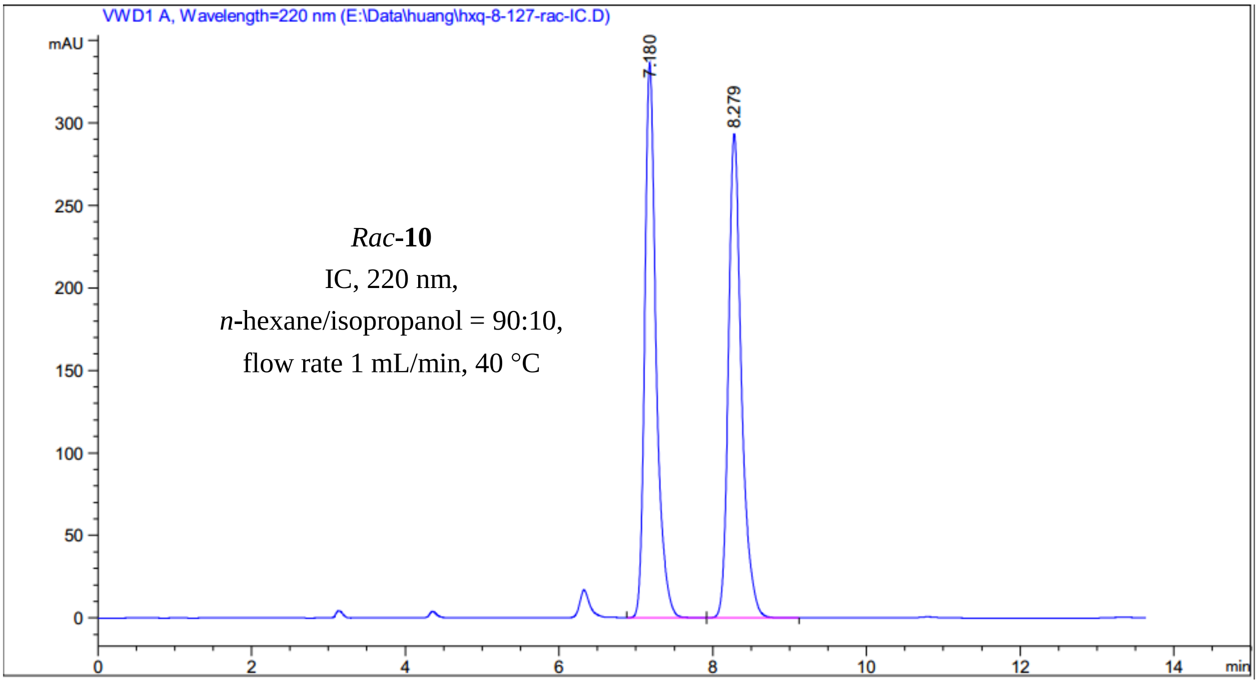

Peak #	RetTime [min]	Type	Width [min]	Area [mAU*s]	Height [mAU]	Area %
1	7.180	BB	0.1539	3456.26978	336.39508	49.9873
2	8.279	BB	0.1775	3458.03223	293.34857	50.0127

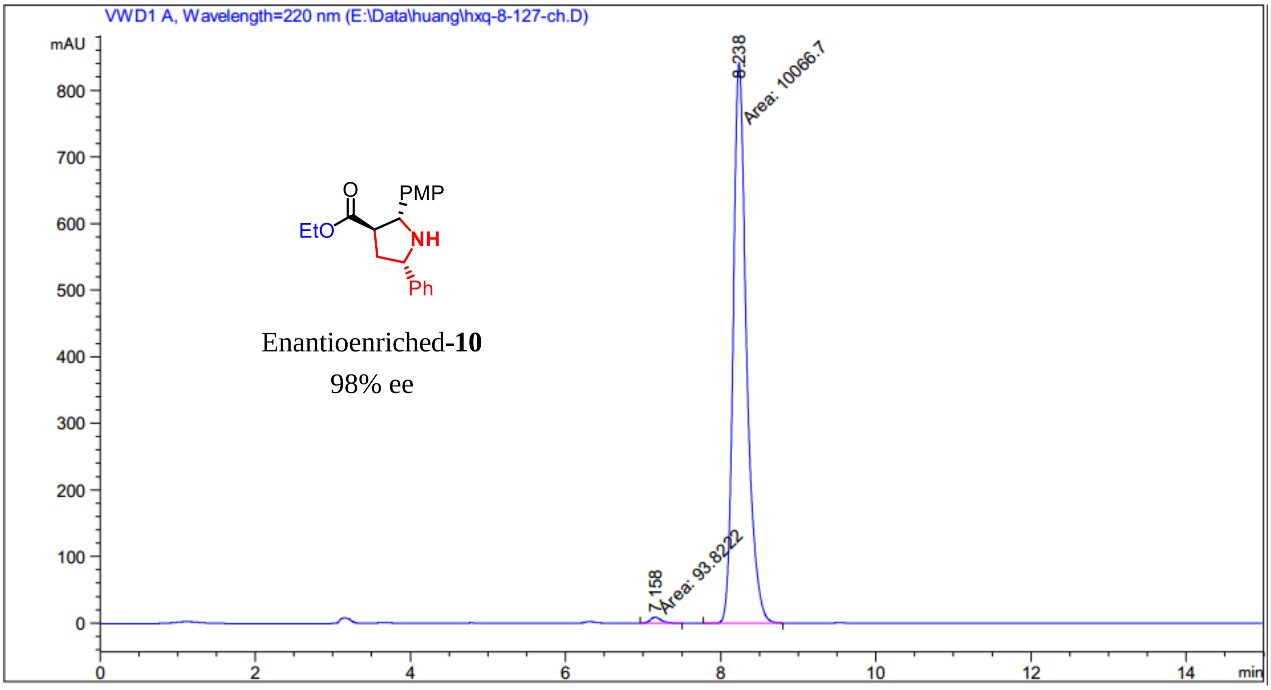

Peak #	RetTime [min]	Type	Width [min]	Area [mAU*s]	Height [mAU]	Area %
1	7.158	FM	0.1710	93.82224	9.14689	0.9234
2	8.238	MF	0.1994	1.00667e4	841.32440	99.0766

Supplementary Figure 38. HPLC traces of *rac*-**10** (reference) and enantioenriched-**10**.

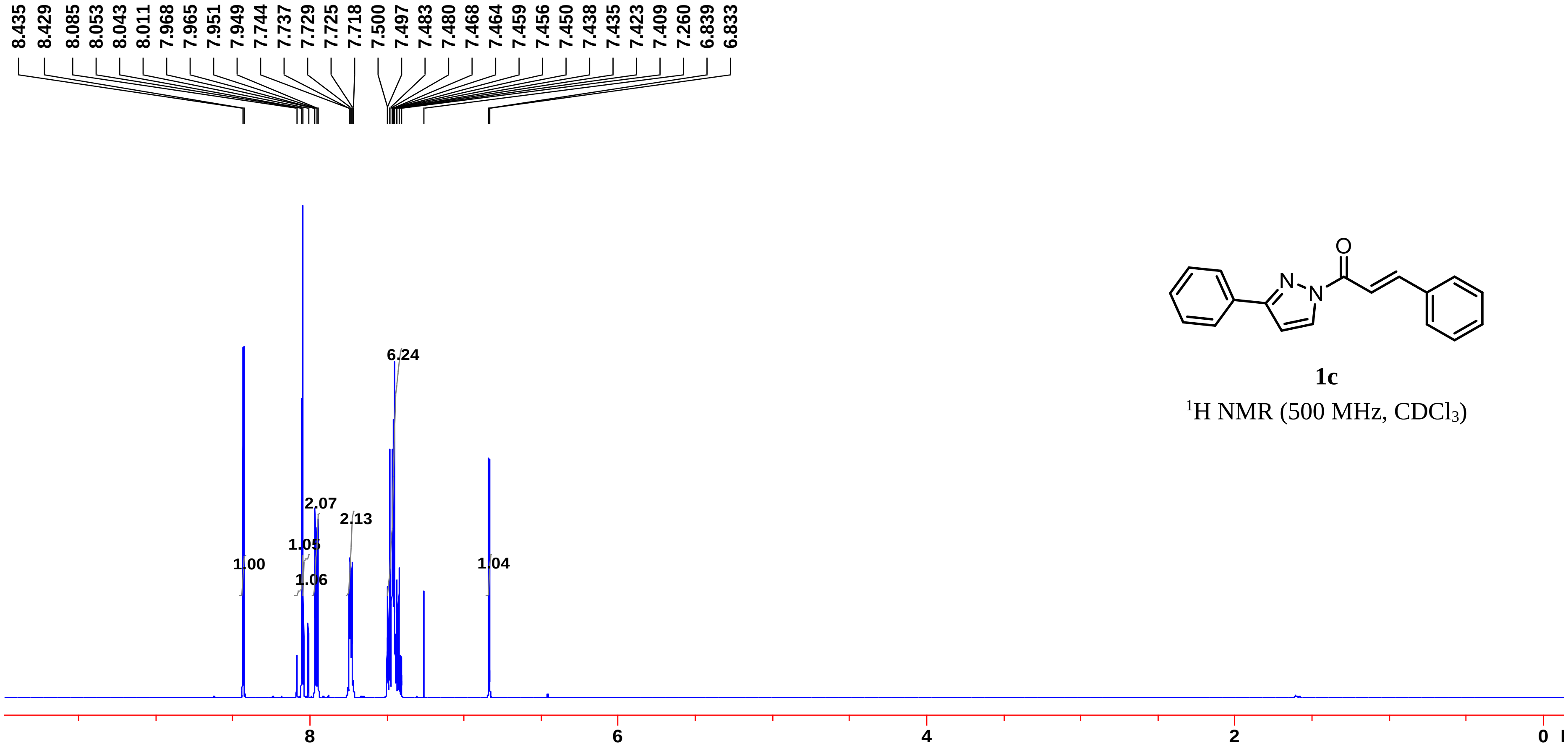

Supplementary Figure 39. ^{1}H NMR spectrum of compound **1c**.

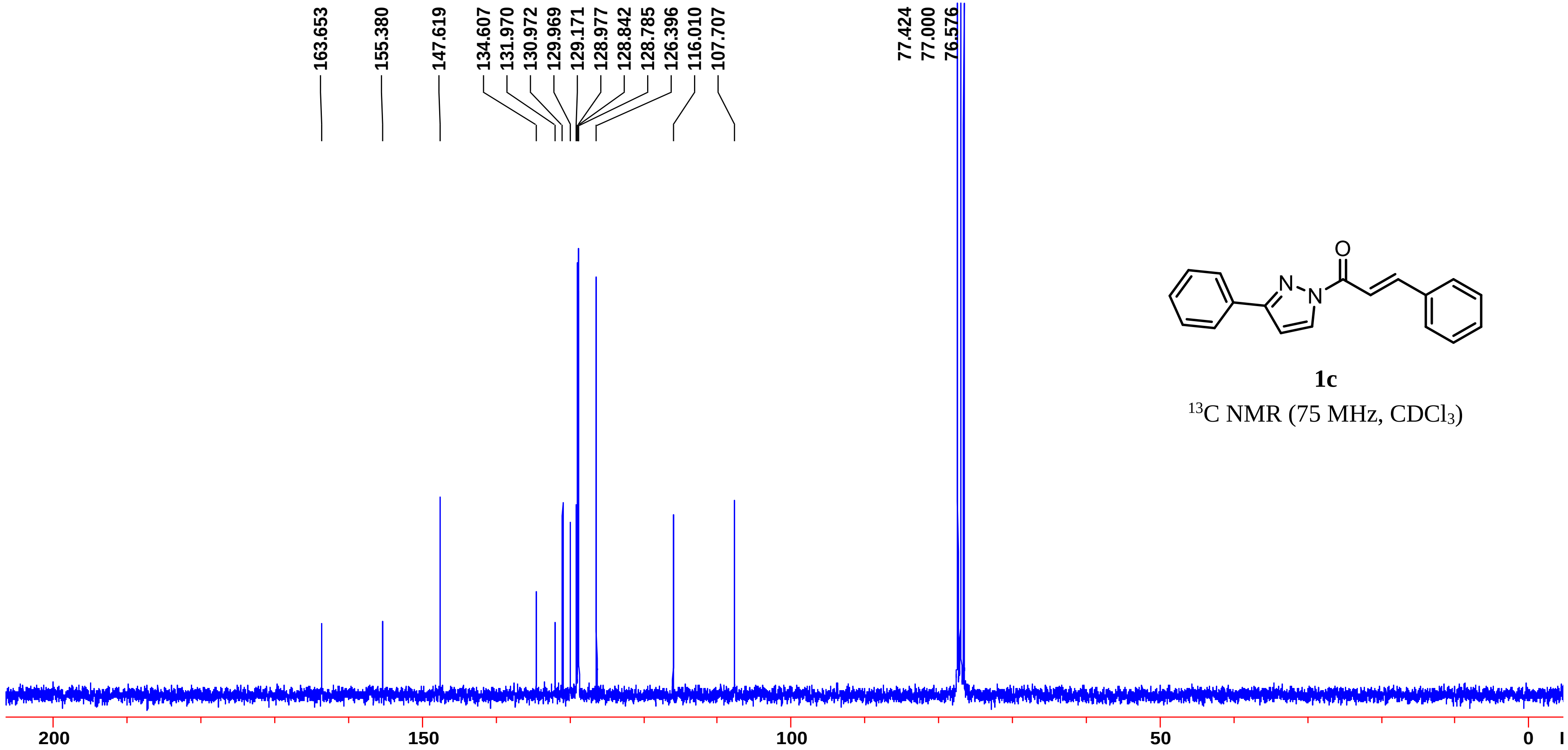

1c

^{13}C NMR (75 MHz, $CDCl_3$)

Supplementary Figure 40. ^{13}C NMR spectrum of compound **1c**.

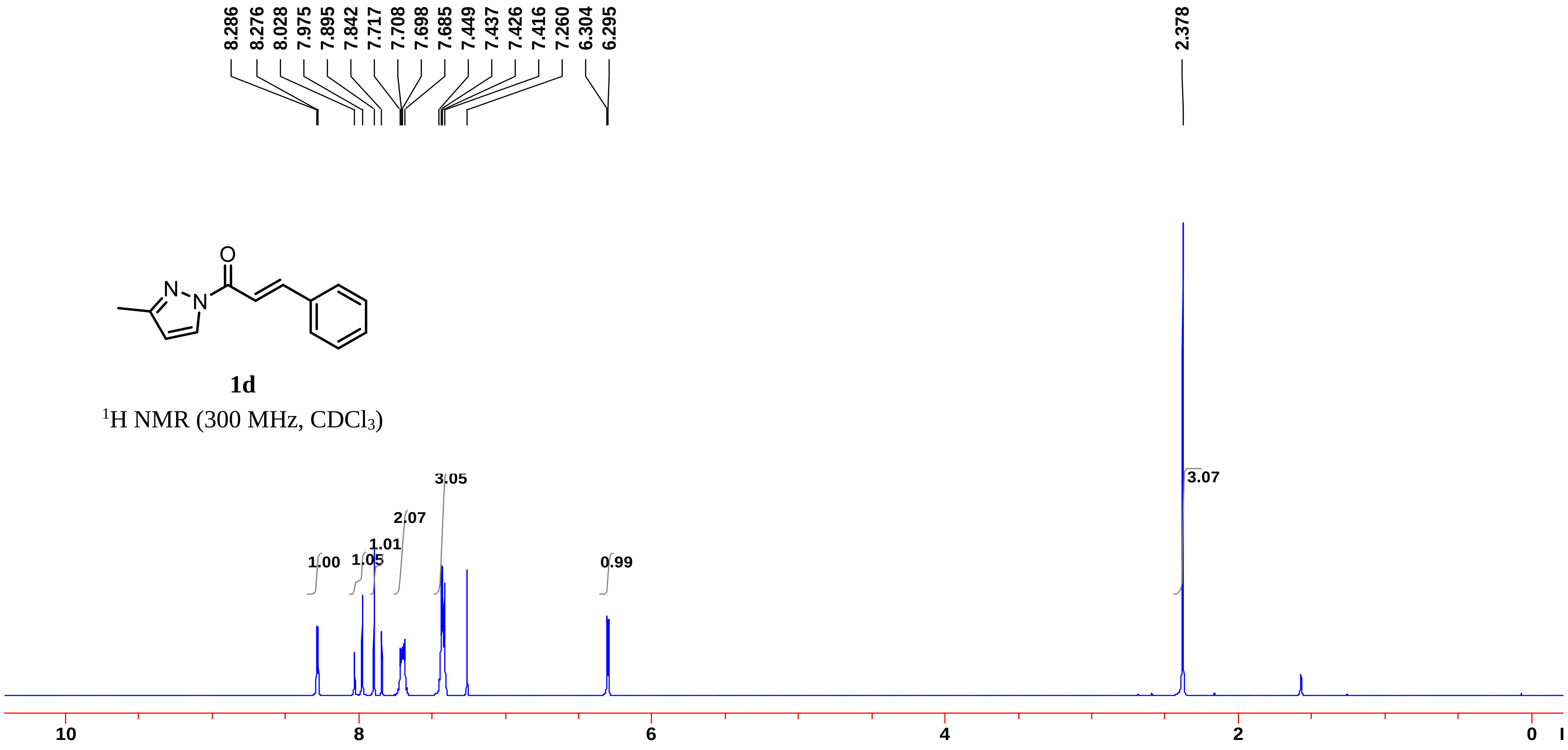

Supplementary Figure 41. ^{1}H NMR spectrum of compound **1d**.

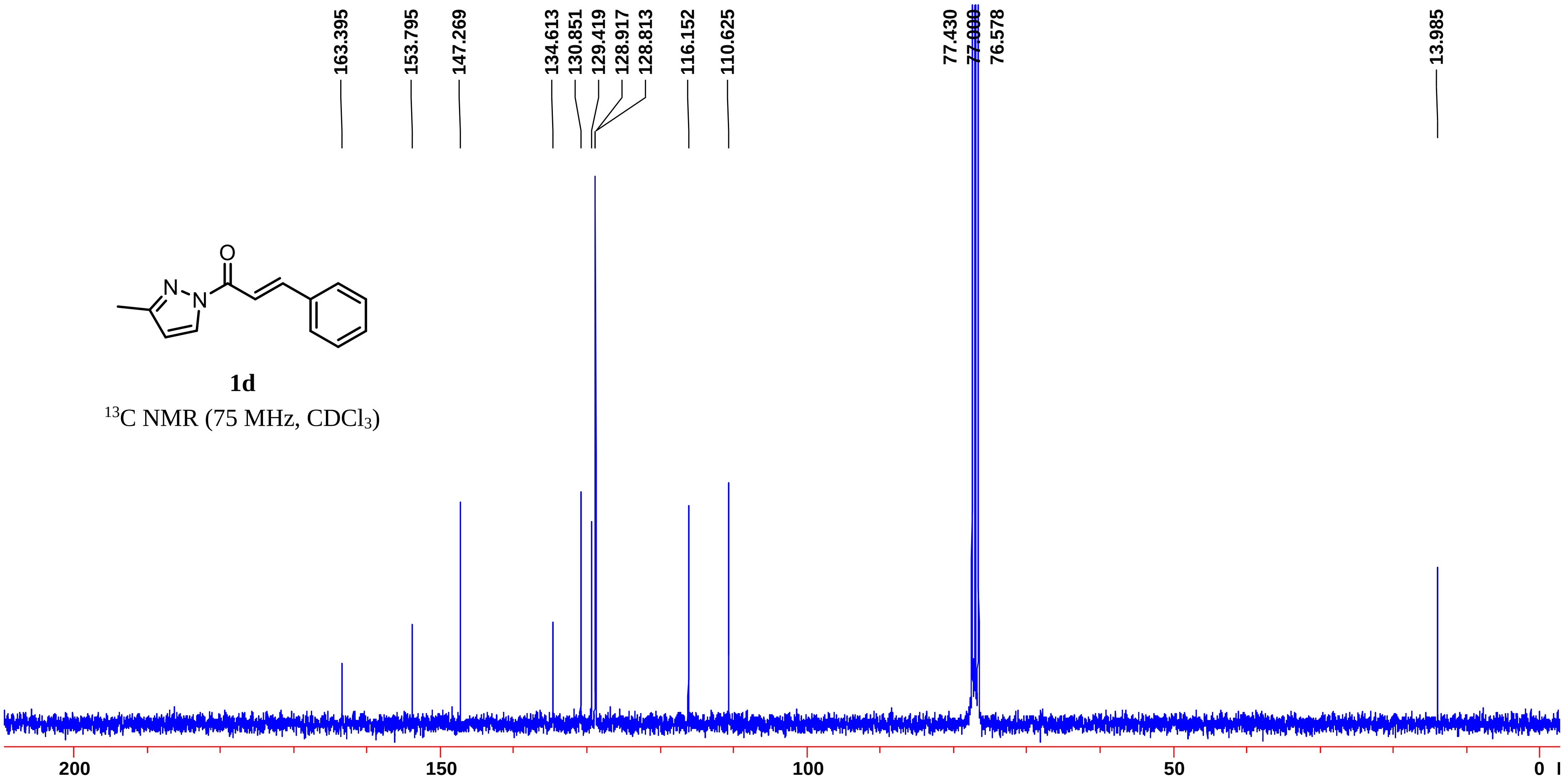

1d

^{13}C NMR (75 MHz, $CDCl_3$)

Supplementary Figure 42. ^{13}C NMR spectrum of compound **1d**.

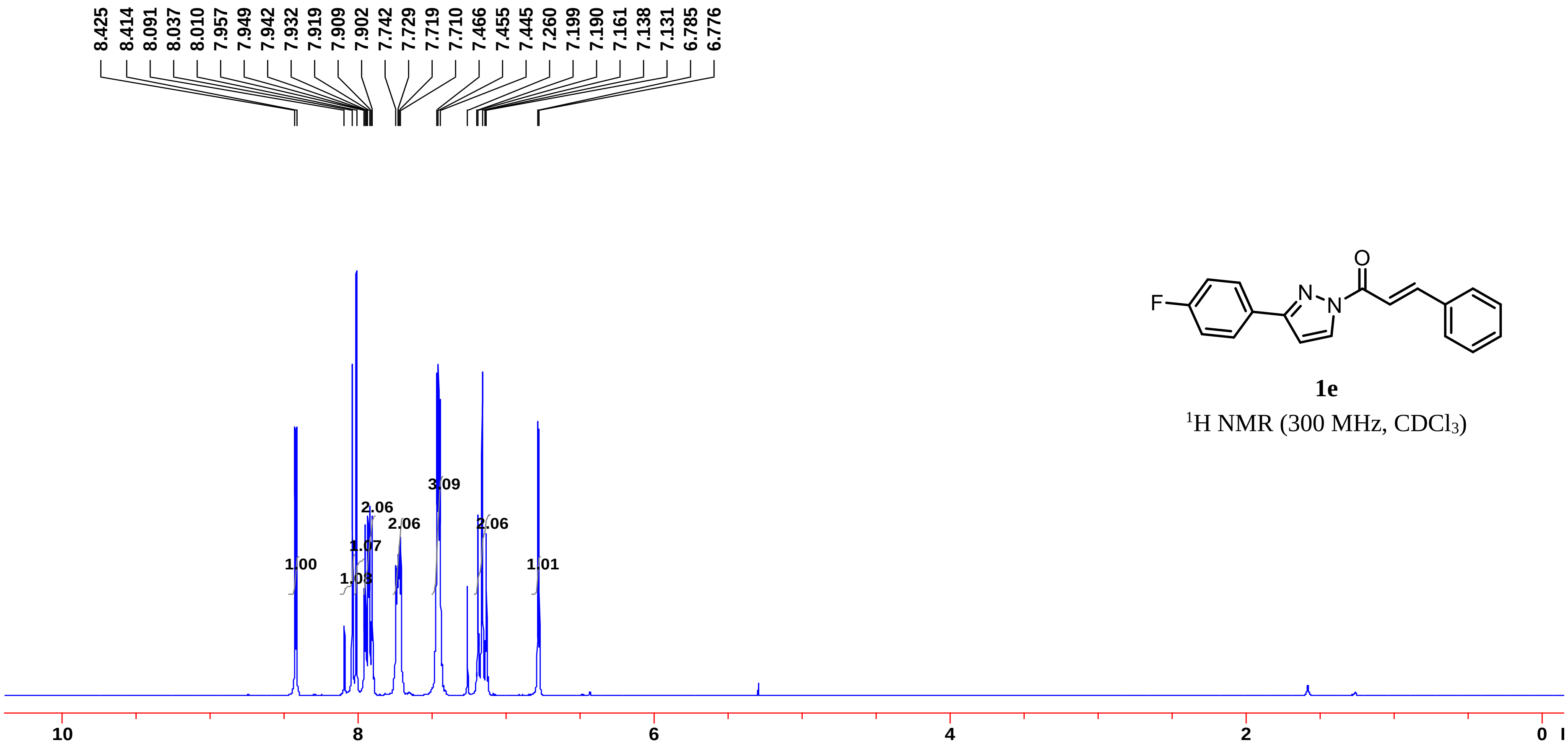

1e

^{1}H NMR (300 MHz, $CDCl_3$)

Supplementary Figure 43. ^{1}H NMR spectrum of compound **1e**.

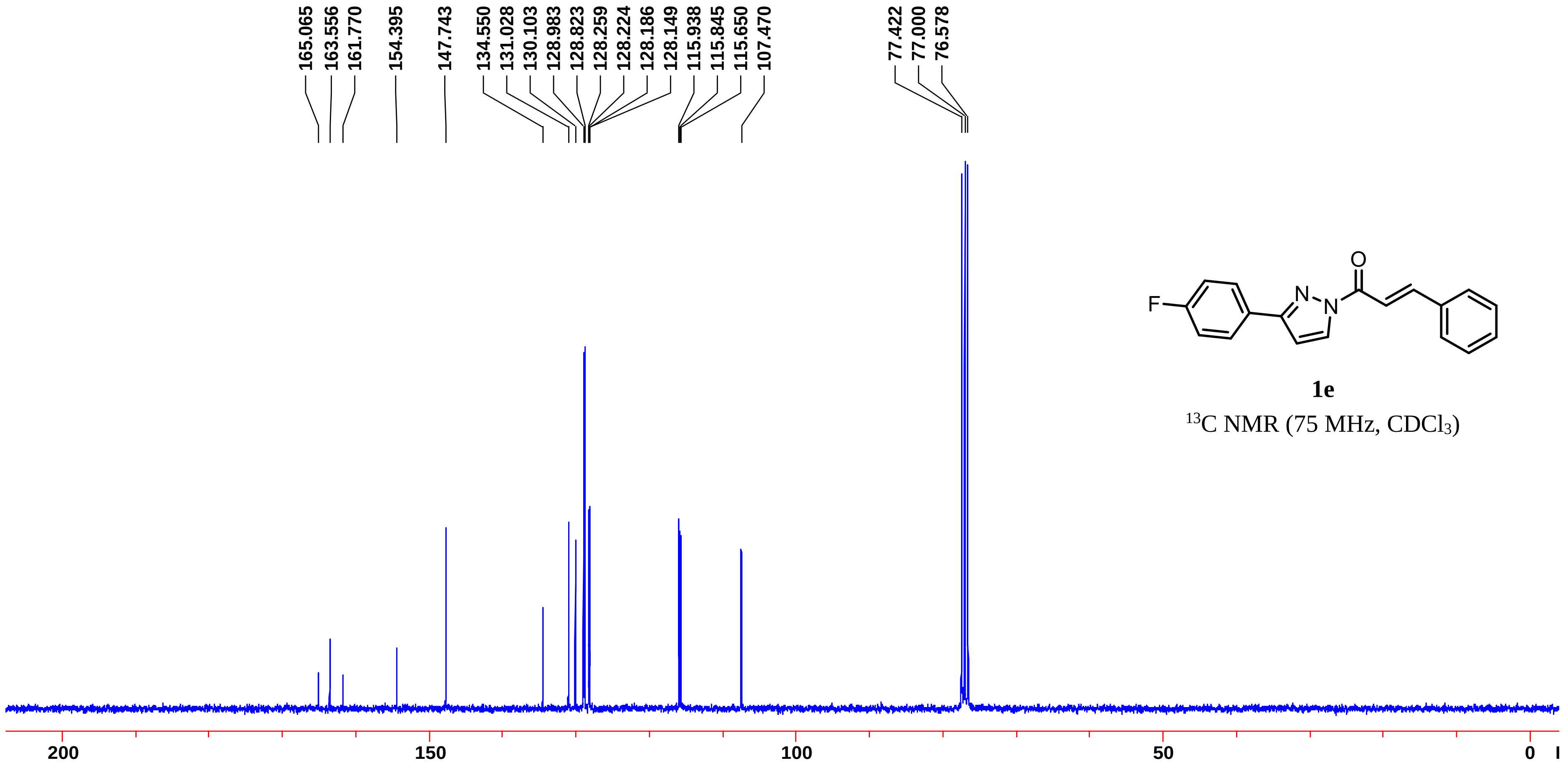

Supplementary Figure 44. ^{13}C NMR spectrum of compound **1e**.

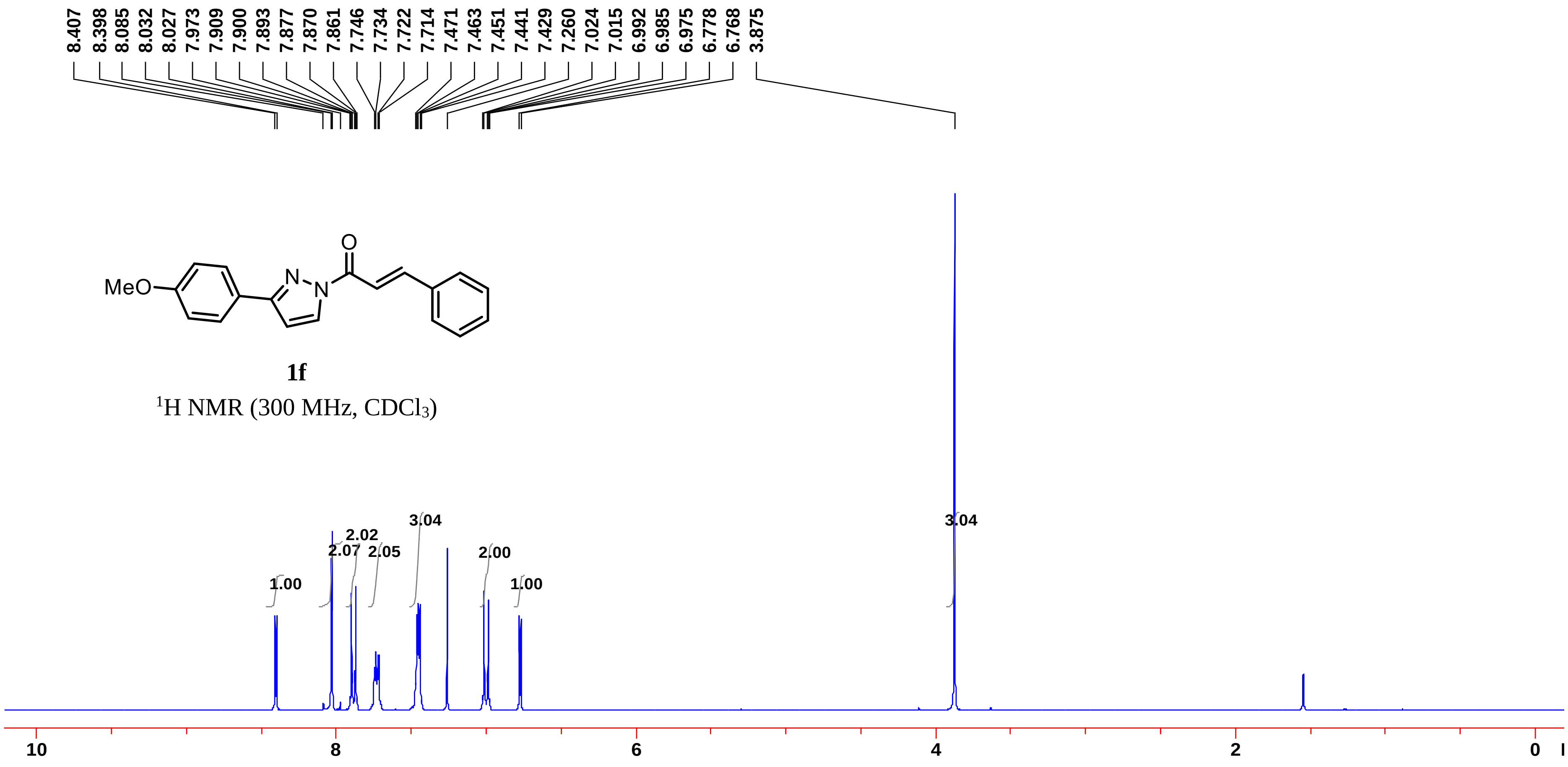

Supplementary Figure 45. ^{1}H NMR spectrum of compound **1f**.

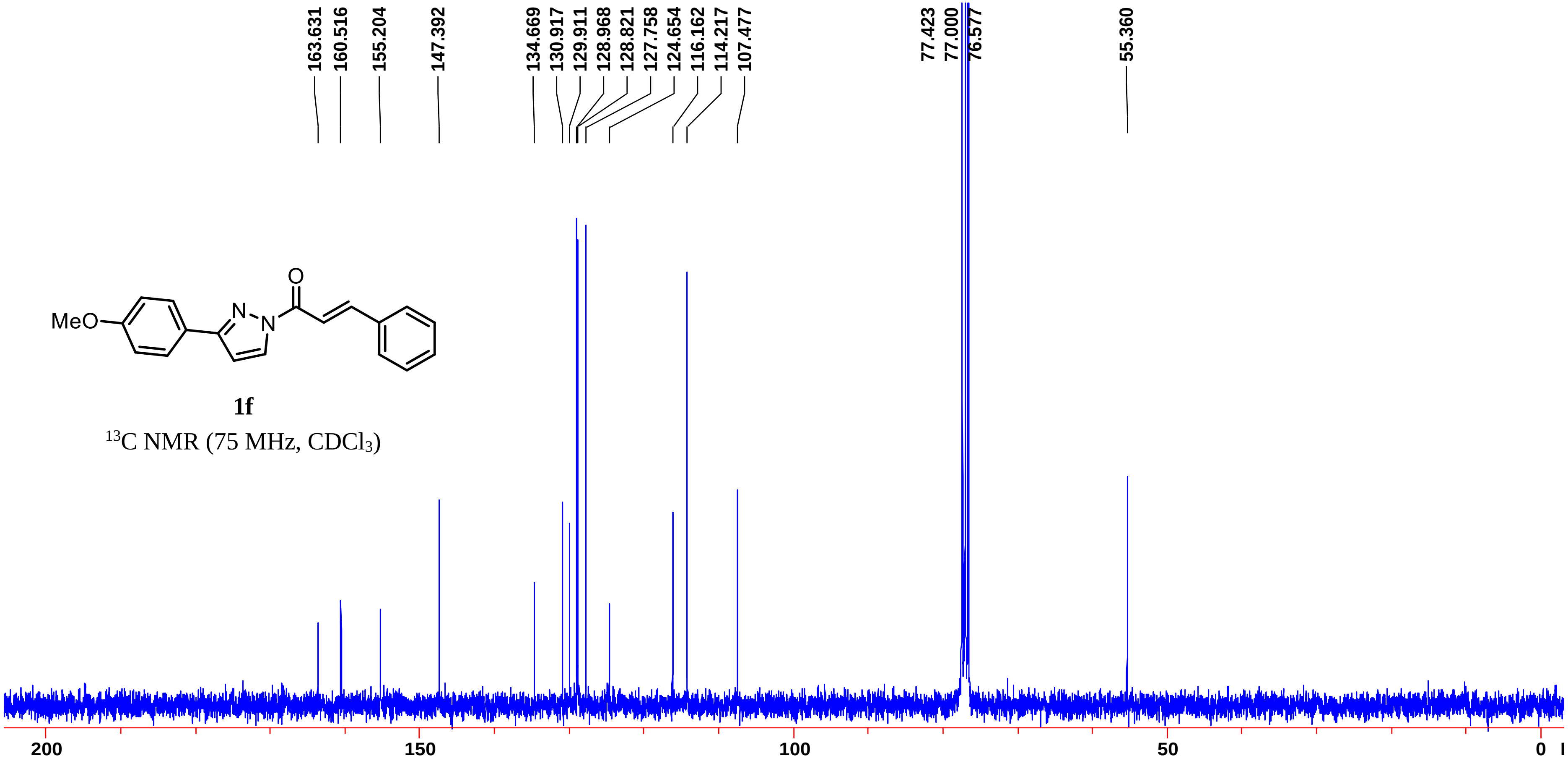

1f

^{13}C NMR (75 MHz, $CDCl_3$)

Supplementary Figure 46. ^{13}C NMR spectrum of compound **1f**.

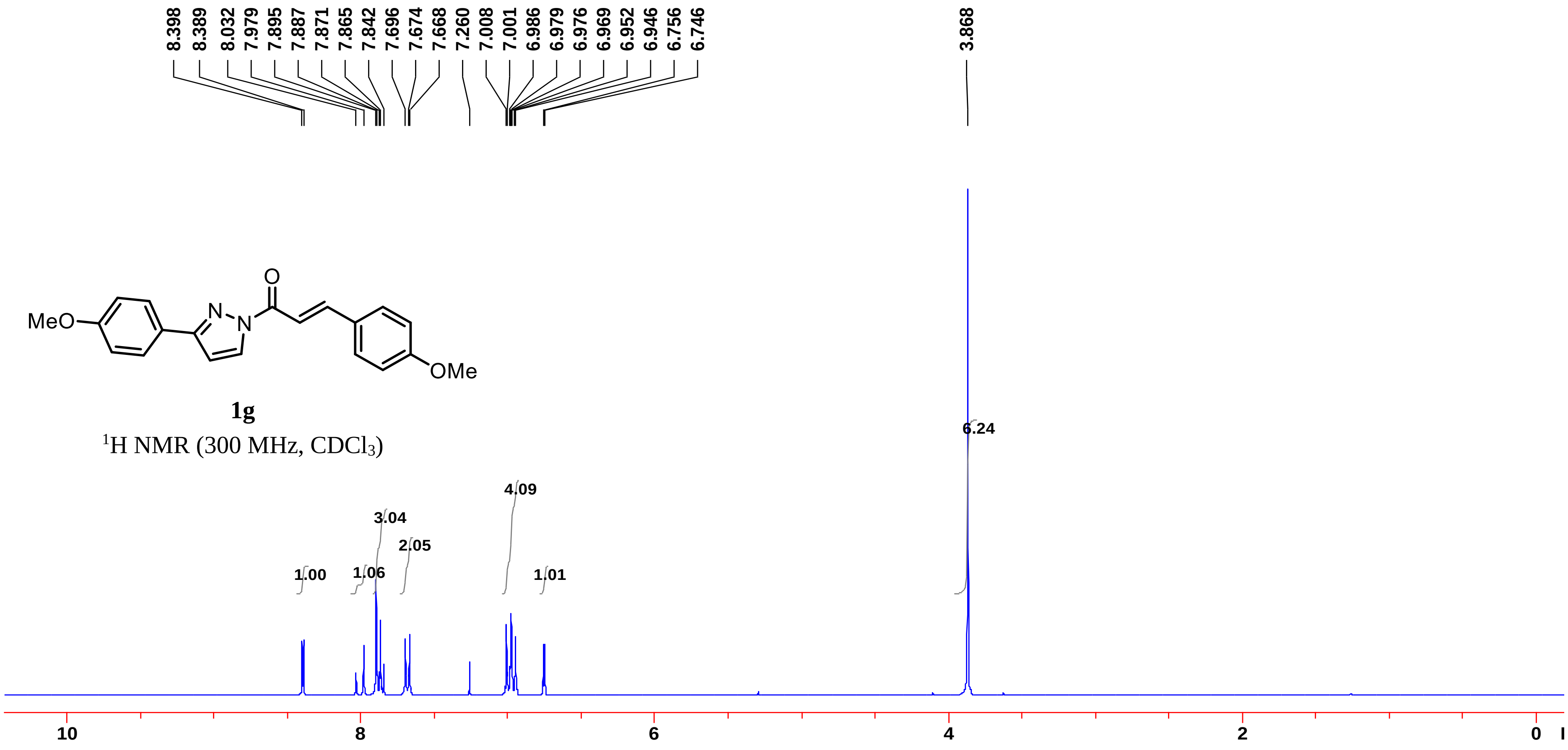

Supplementary Figure 47. ^{1}H NMR spectrum of compound **1g**.

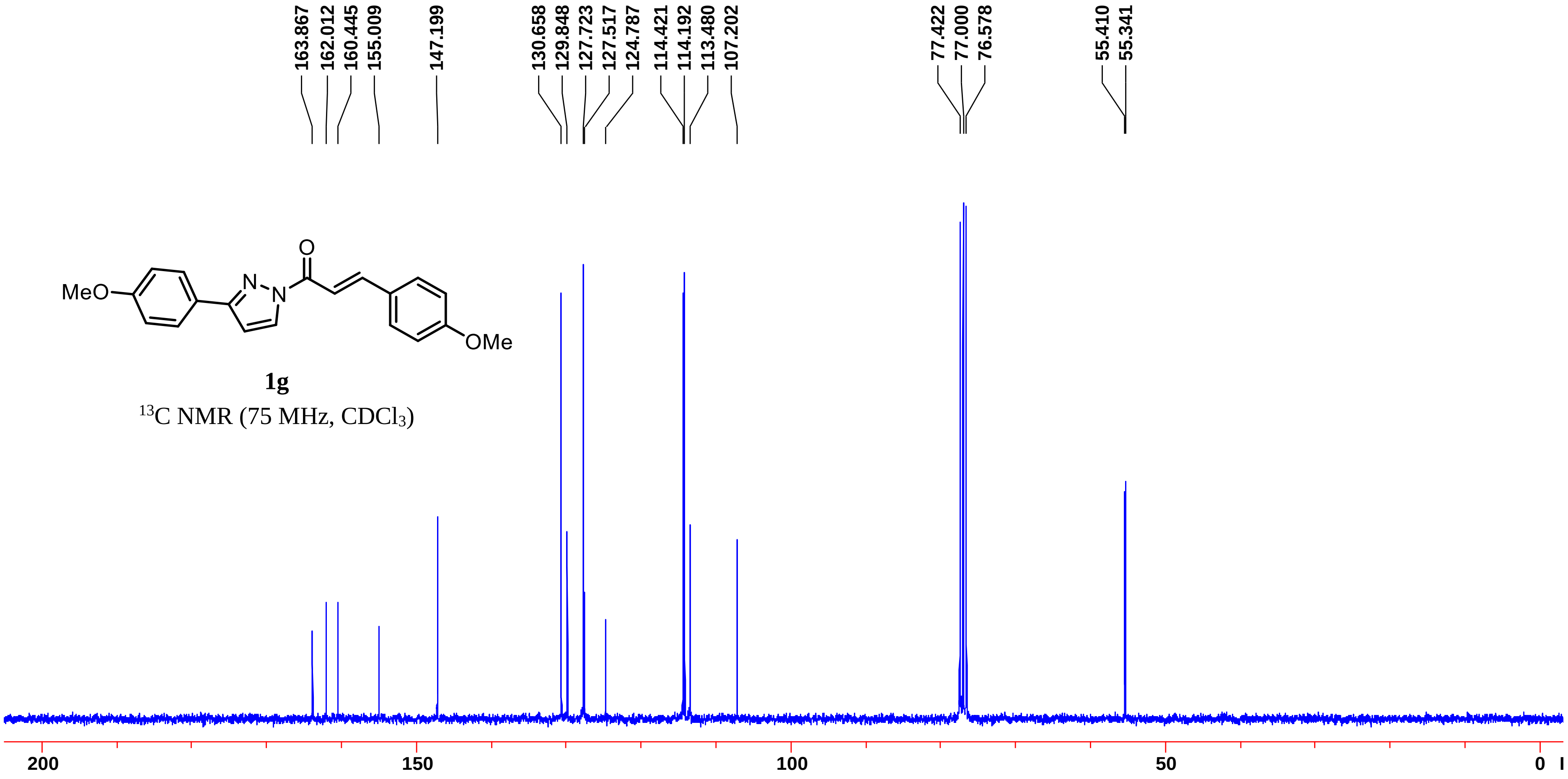

Supplementary Figure 48. ^{13}C NMR spectrum of compound **1g**.

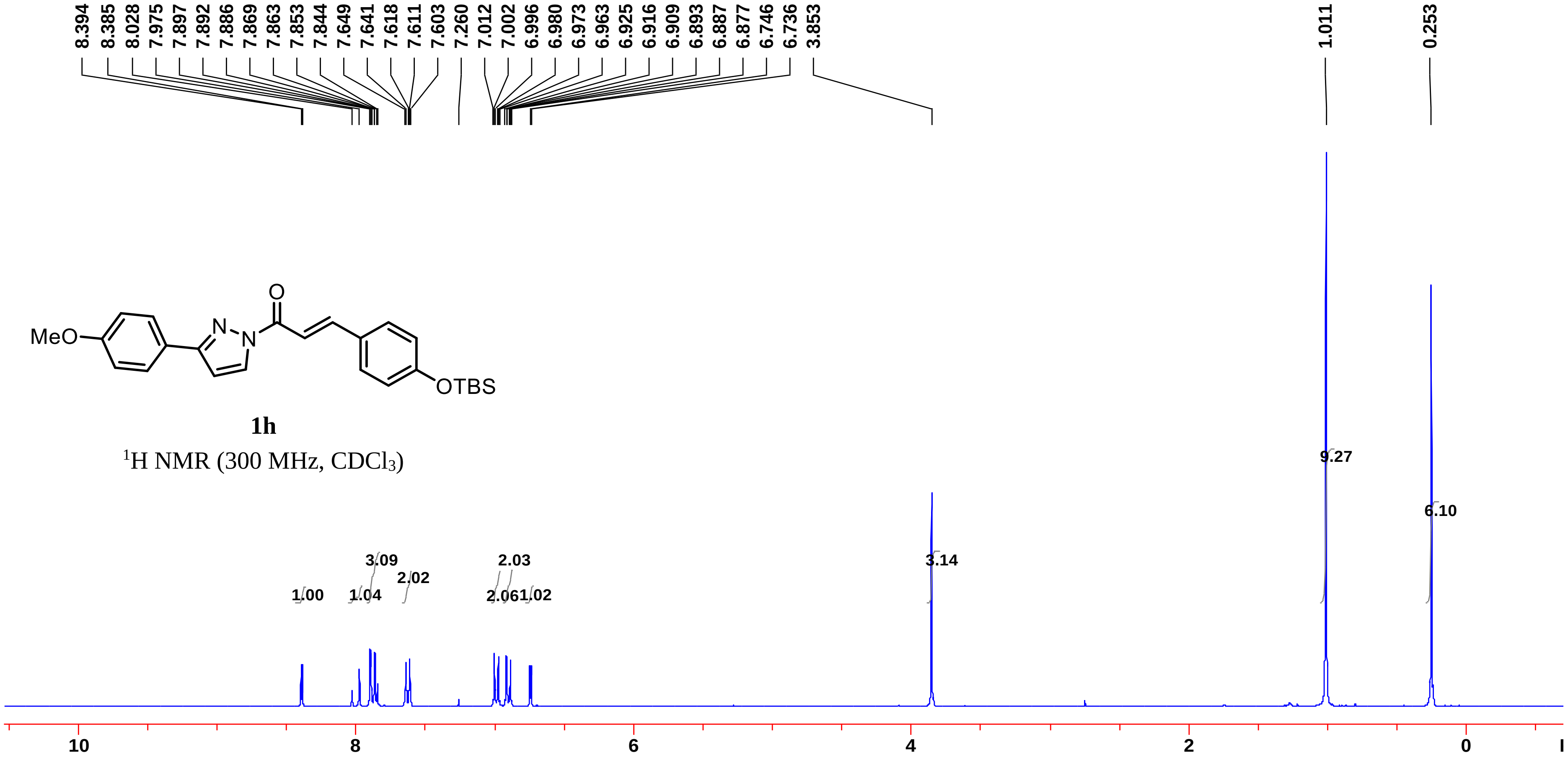

Supplementary Figure 49. ^{1}H NMR spectrum of compound **1h**.

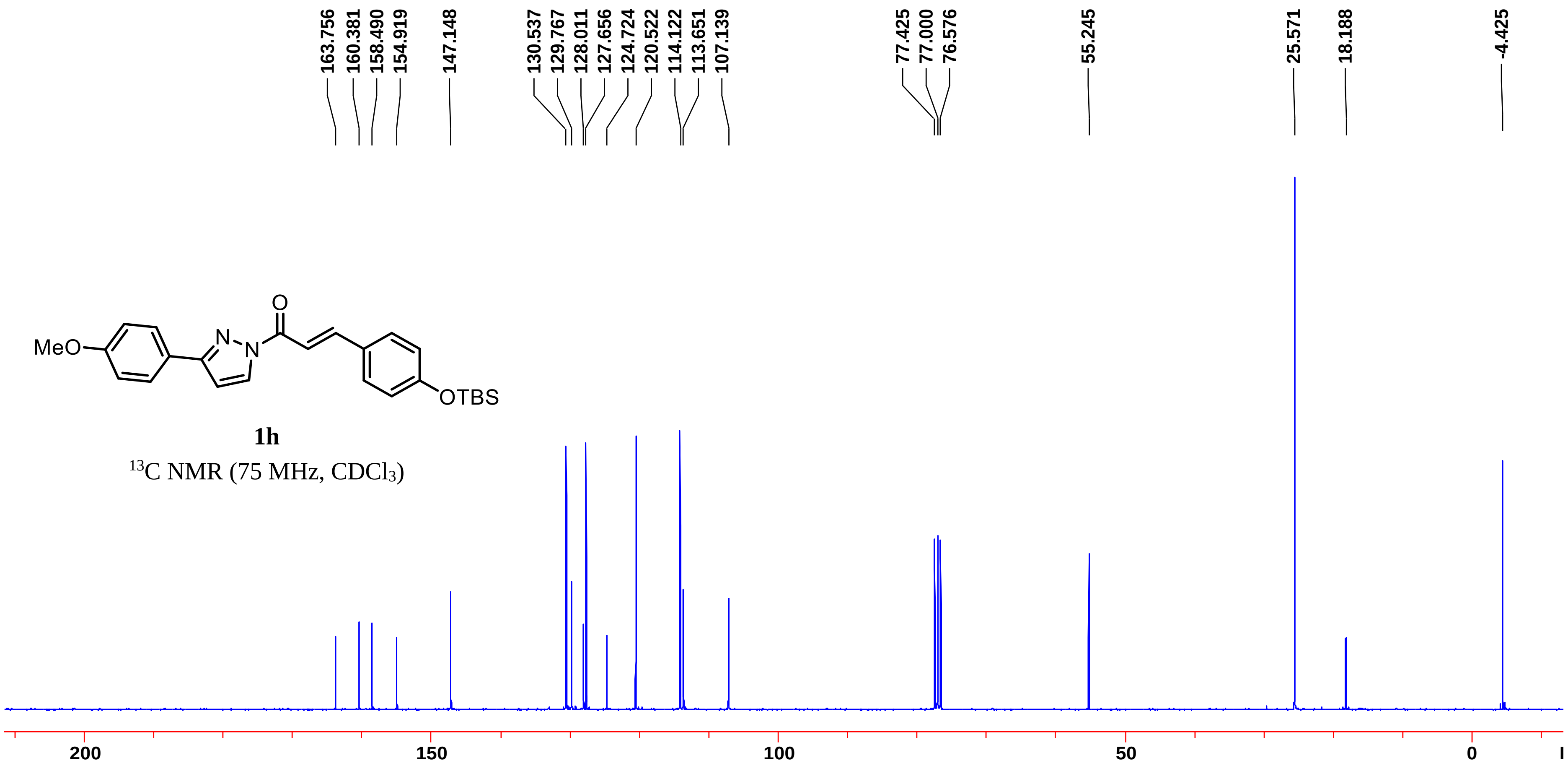

Supplementary Figure 50. ^{13}C NMR spectrum of compound **1h**.

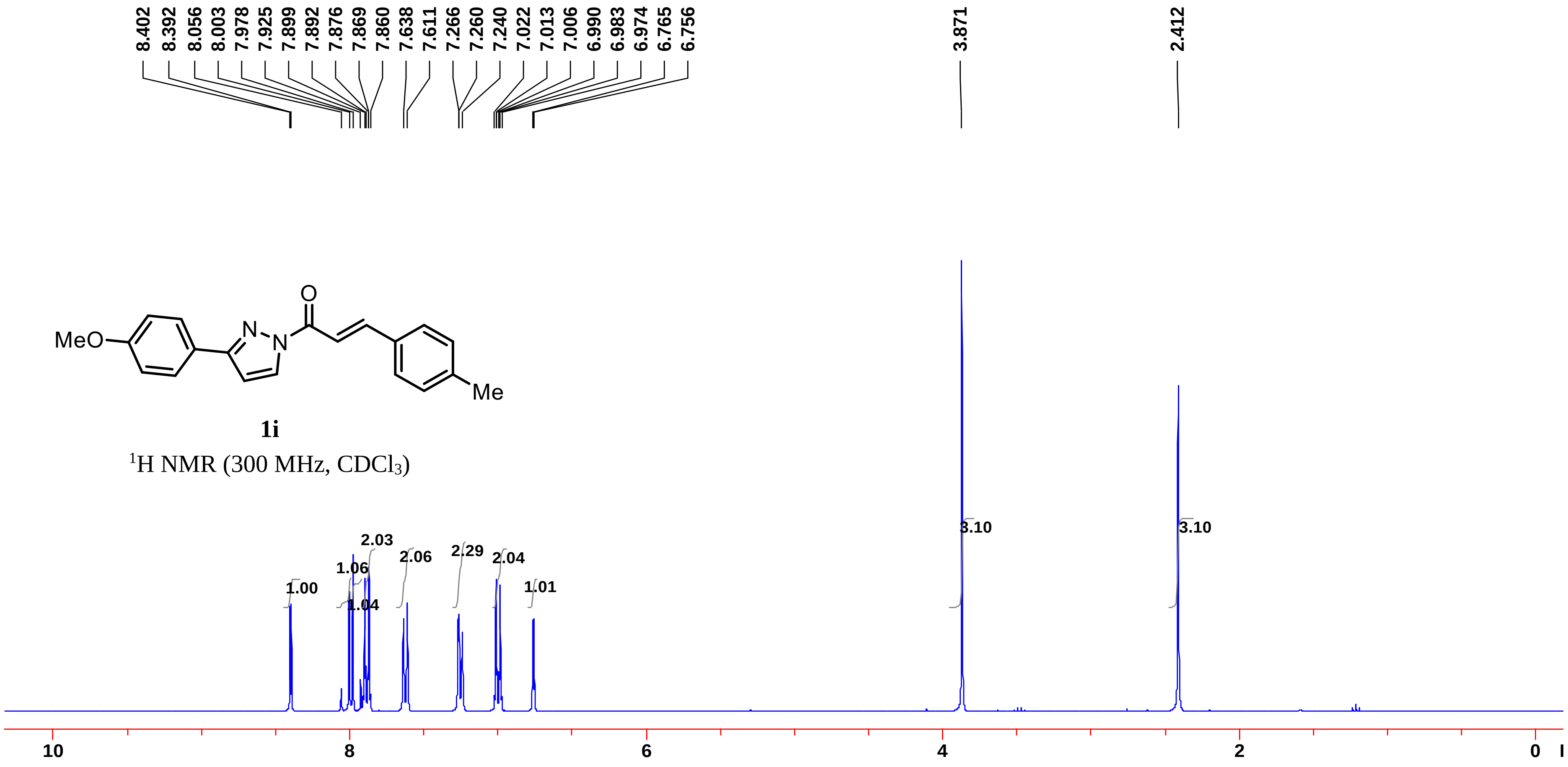

Supplementary Figure 51. ^{1}H NMR spectrum of compound **1i**.

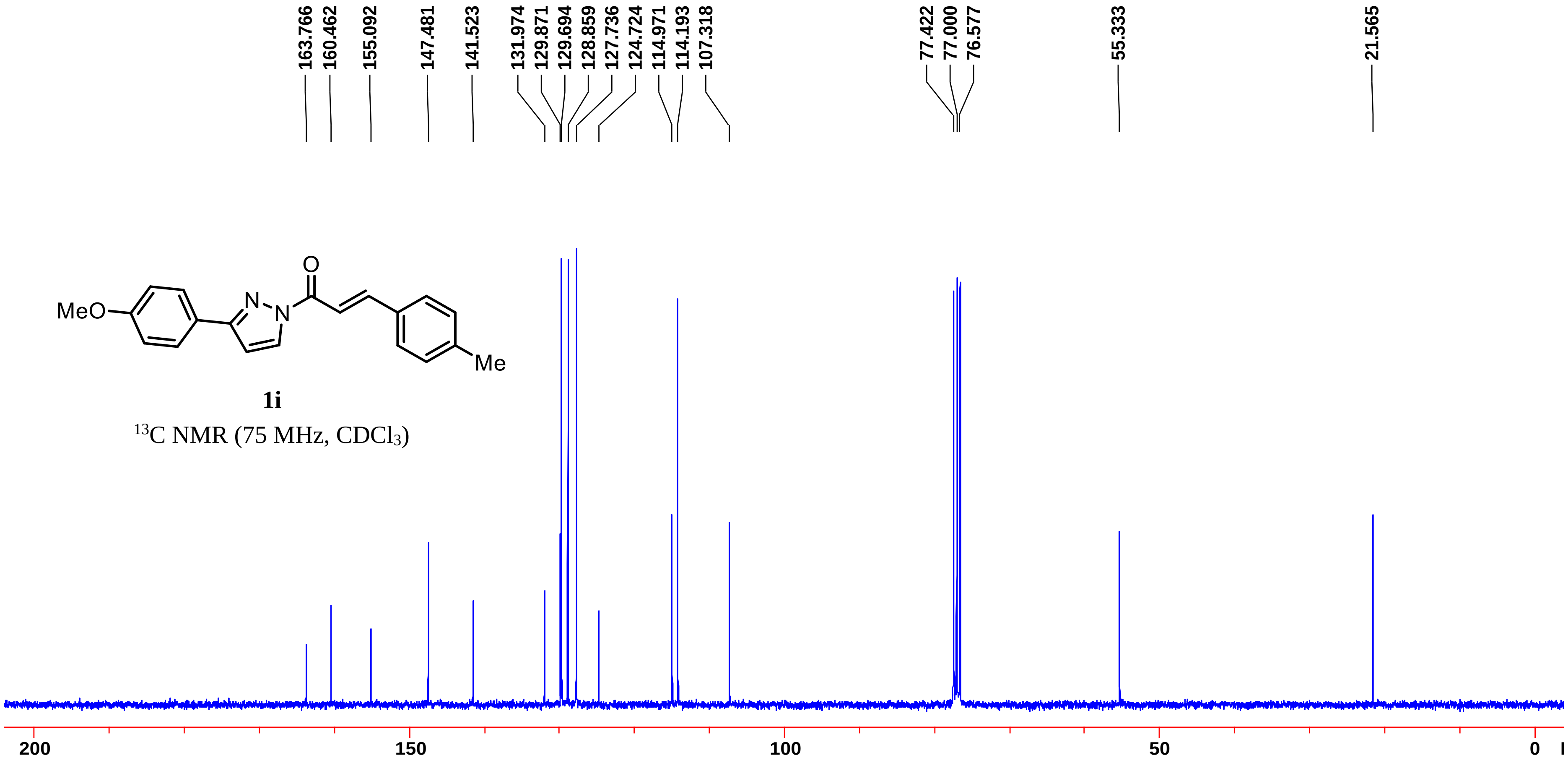

Supplementary Figure 52. ^{13}C NMR spectrum of compound **1i**.

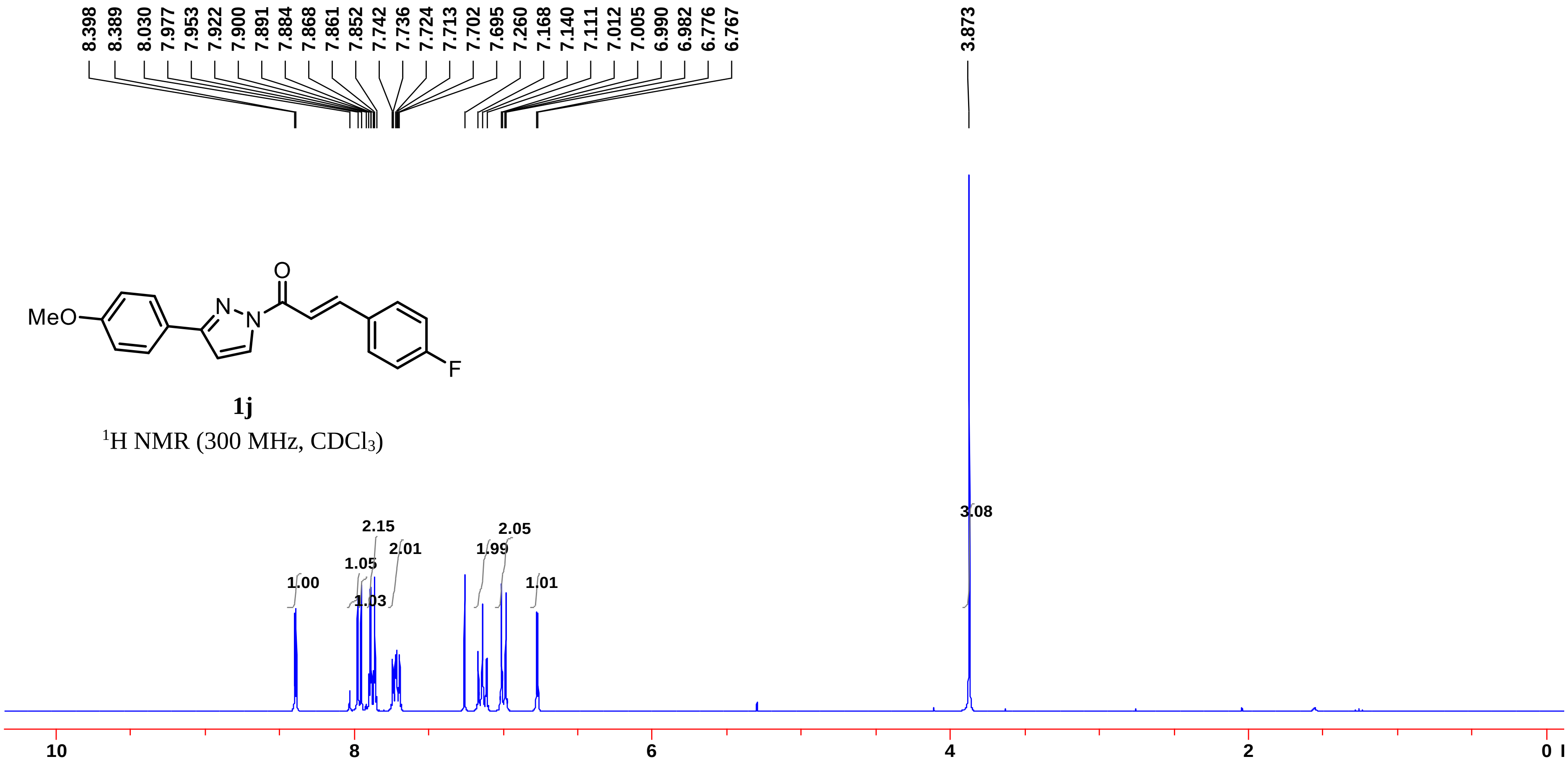

Supplementary Figure 53. ^{1}H NMR spectrum of compound **1j**.

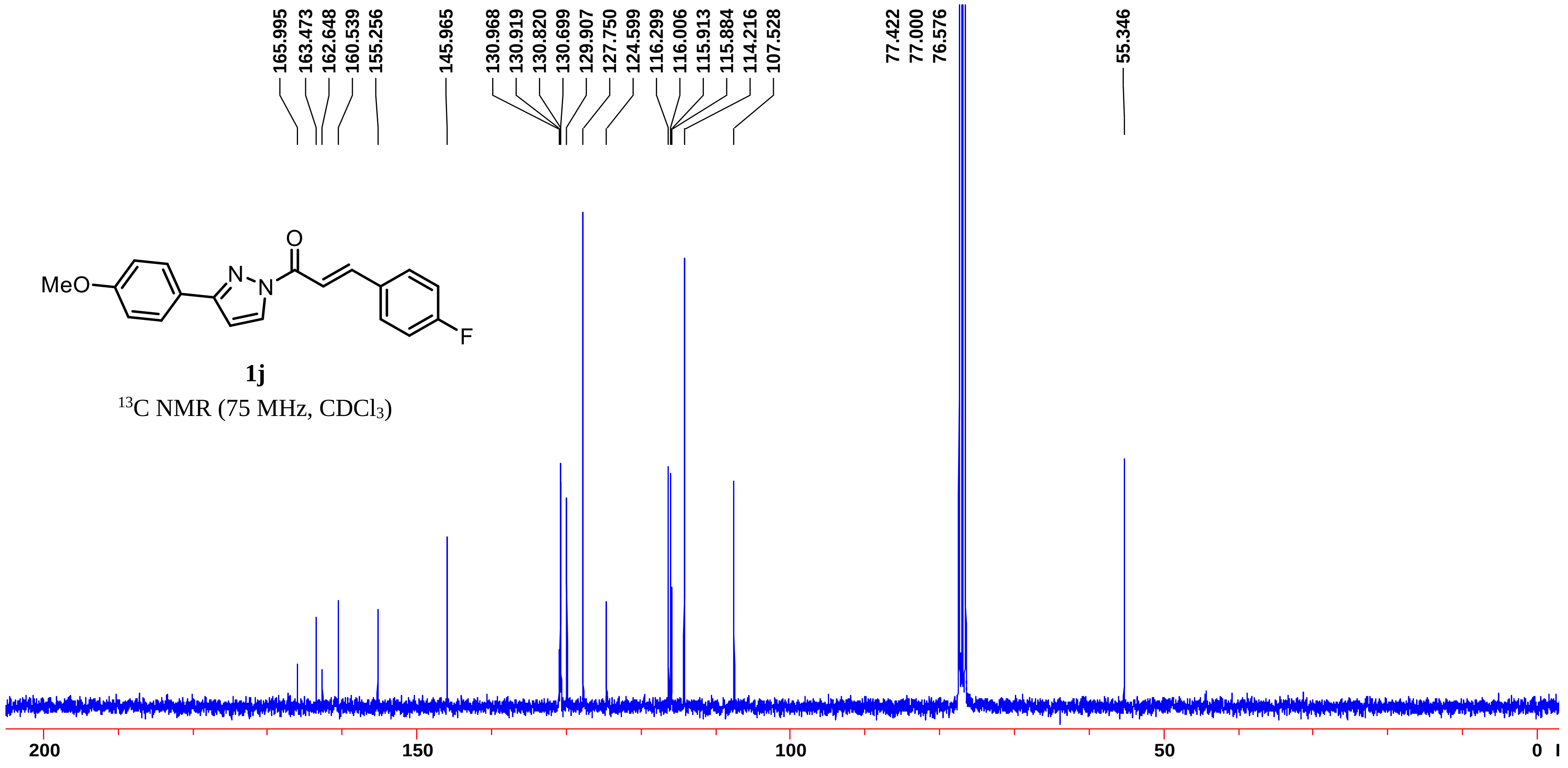

Supplementary Figure 54. ^{13}C NMR spectrum of compound **1j**.

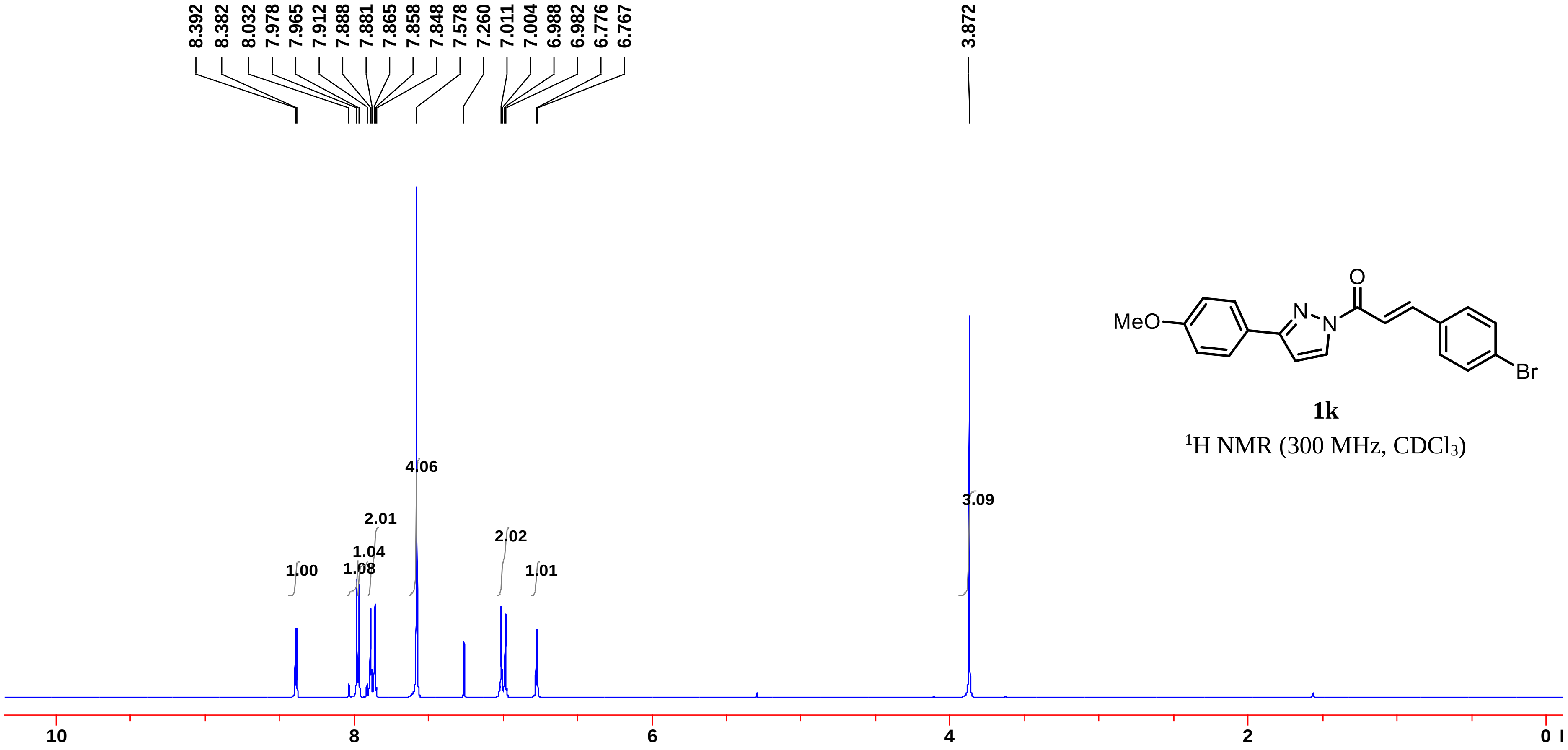

Supplementary Figure 55. ^{1}H NMR spectrum of compound **1k**.

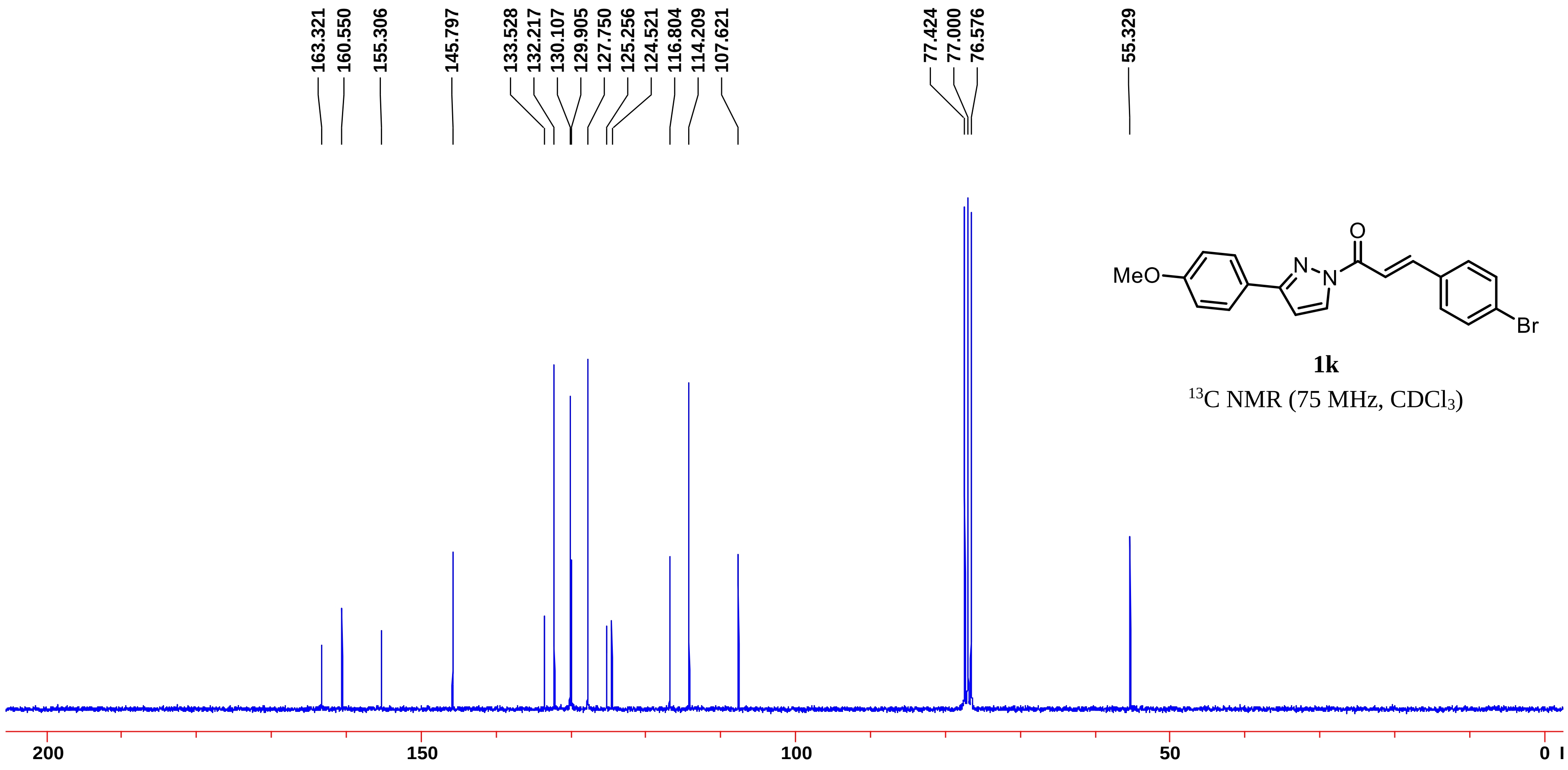

Supplementary Figure 56. ^{13}C NMR spectrum of compound **1k**.

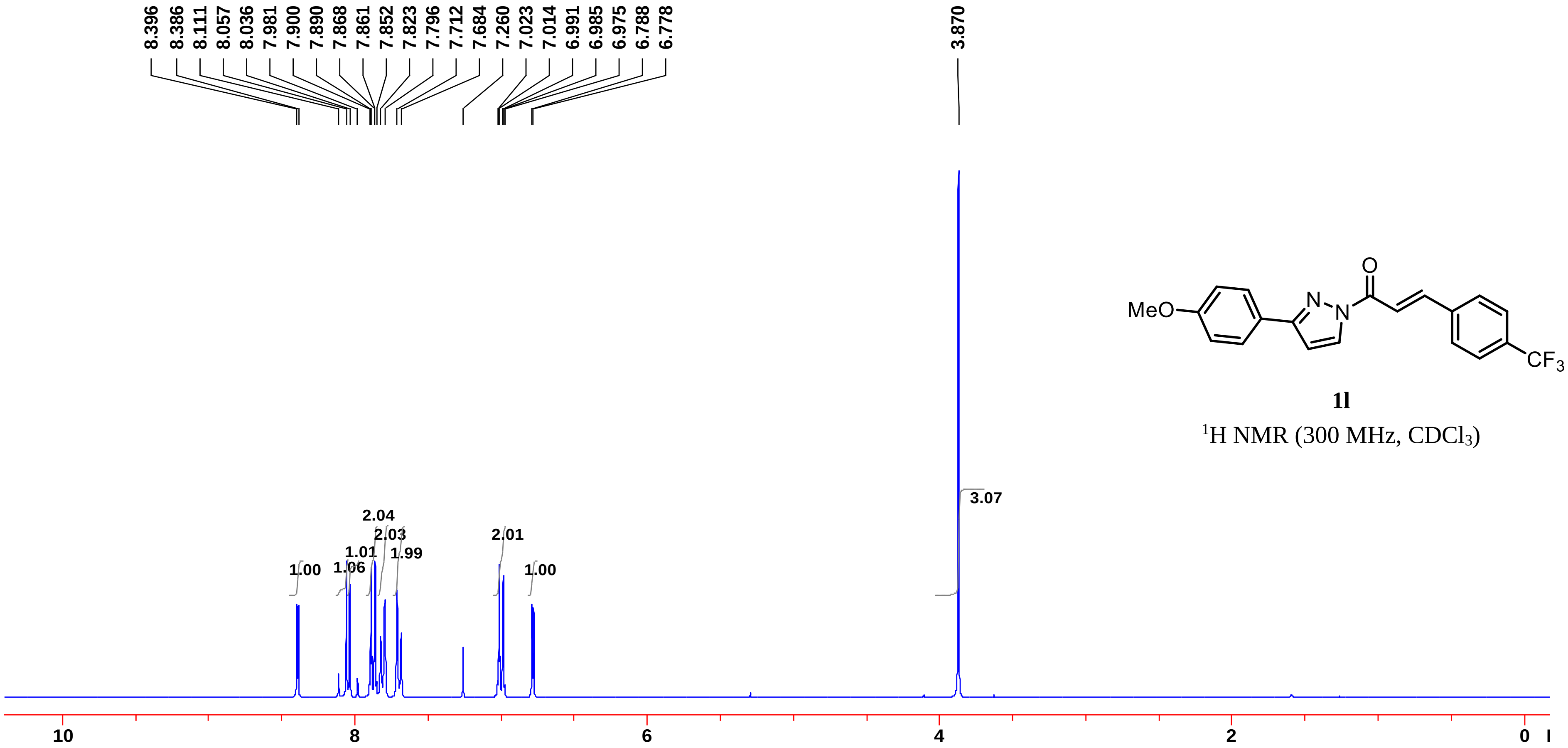

1l

^{1}H NMR (300 MHz, $CDCl_3$)

Supplementary Figure 57. ^{1}H NMR spectrum of compound **1l**.

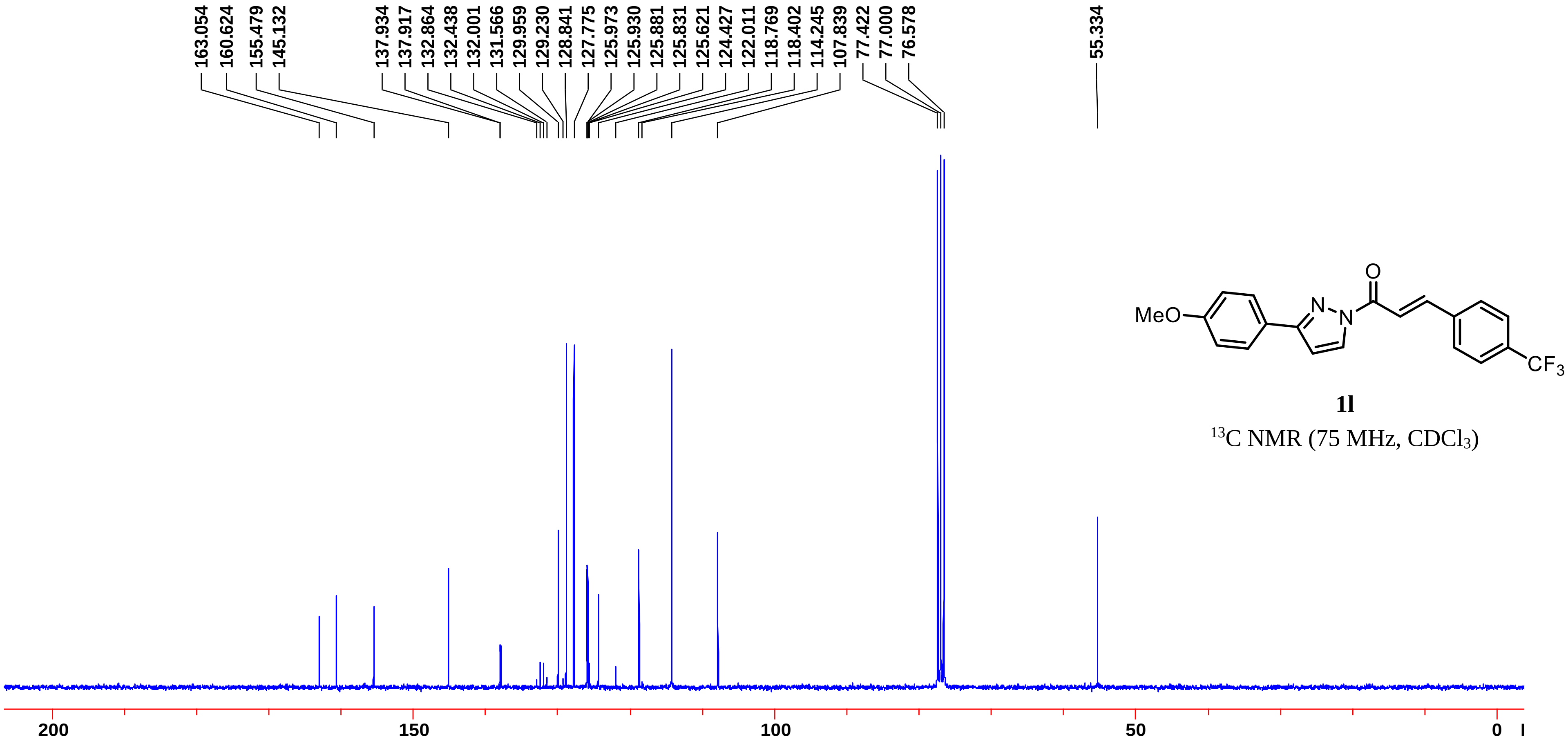

1l

^{13}C NMR (75 MHz, $CDCl_3$)

Supplementary Figure 58. ^{13}C NMR spectrum of compound **1l**.

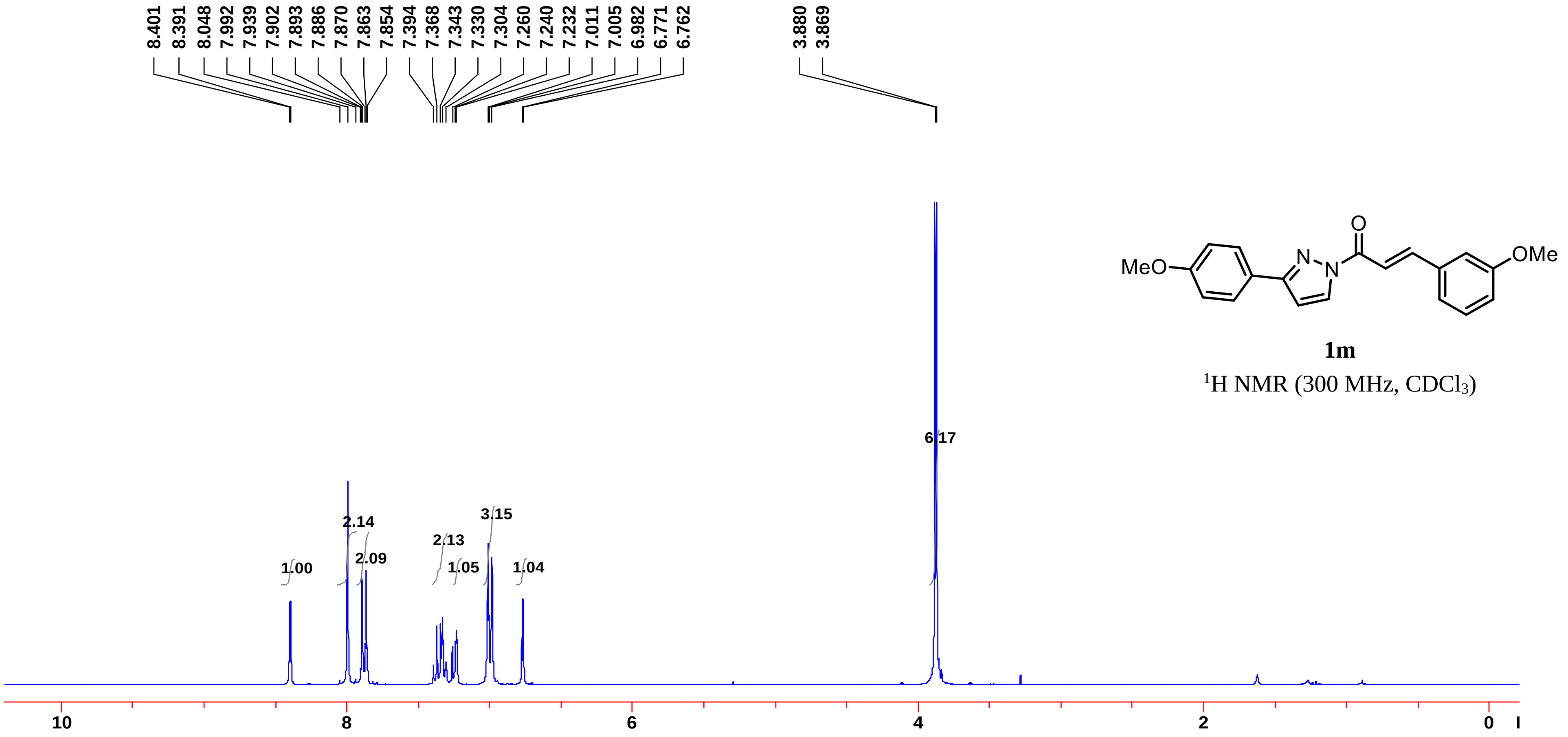

1m

^{1}H NMR (300 MHz, $CDCl_3$)

Supplementary Figure 59. ^{1}H NMR spectrum of compound **1m**.

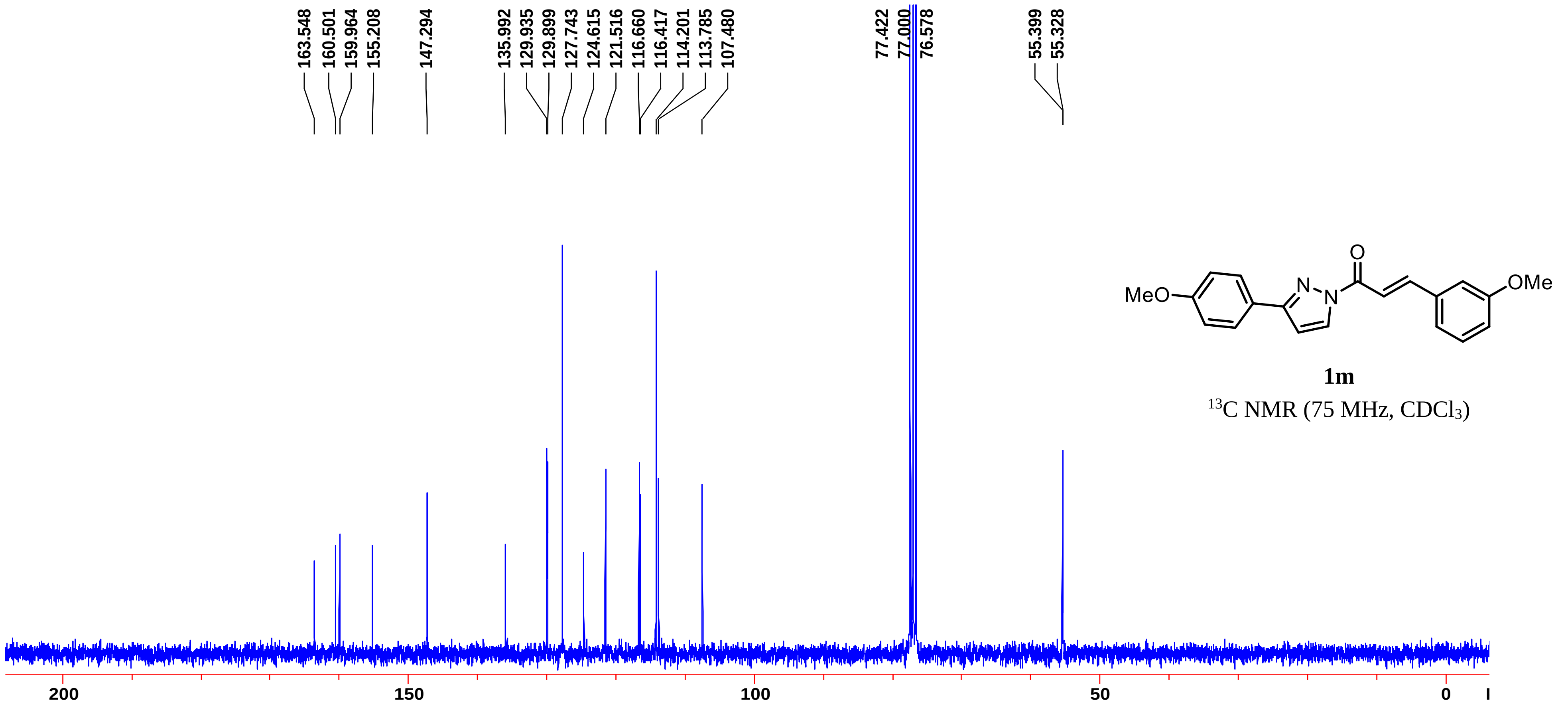

1m

^{13}C NMR (75 MHz, $CDCl_3$)

Supplementary Figure 60. ^{13}C NMR spectrum of compound **1m**.

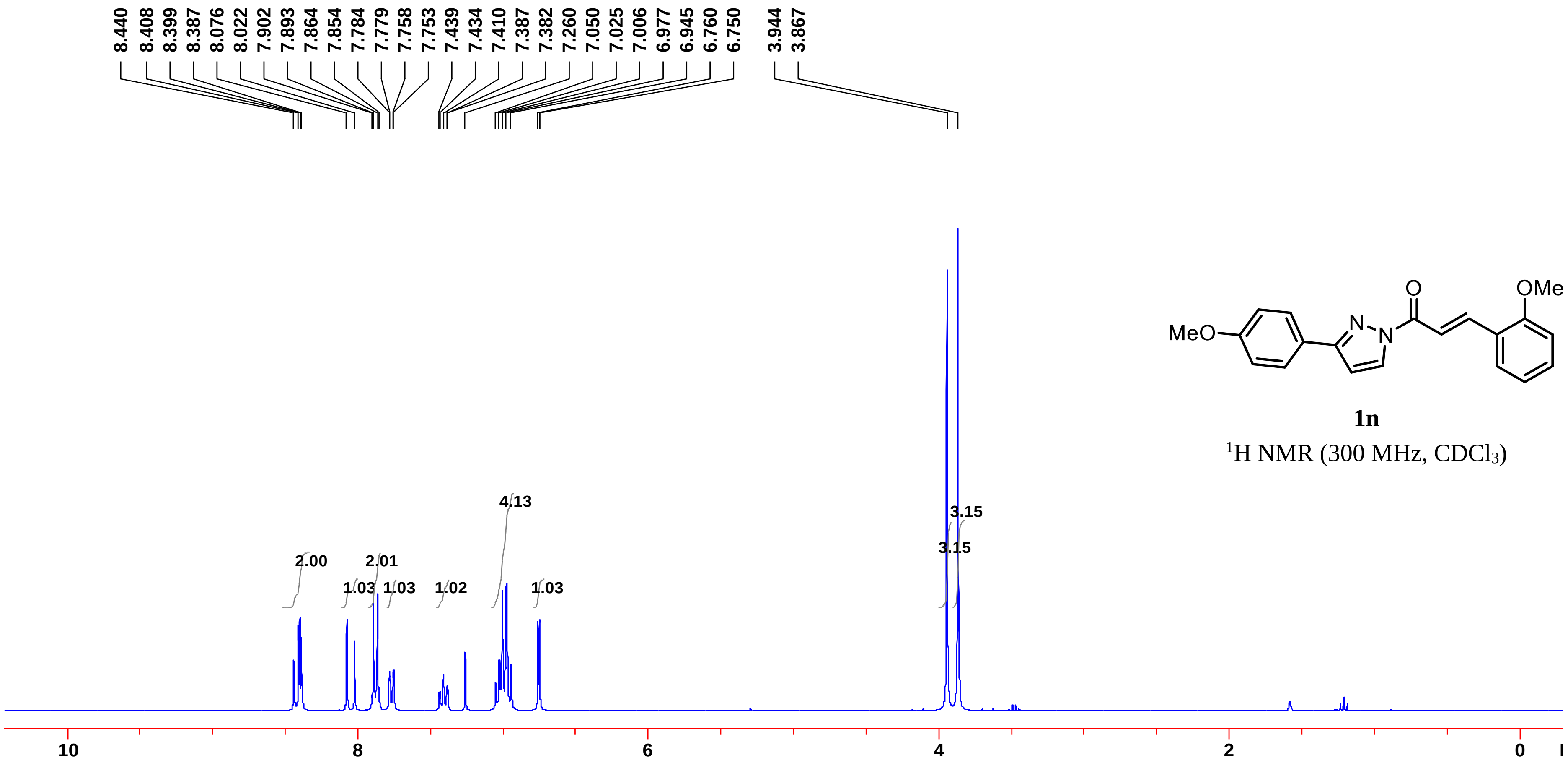

Supplementary Figure 61. ¹H NMR spectrum of compound **1n**.

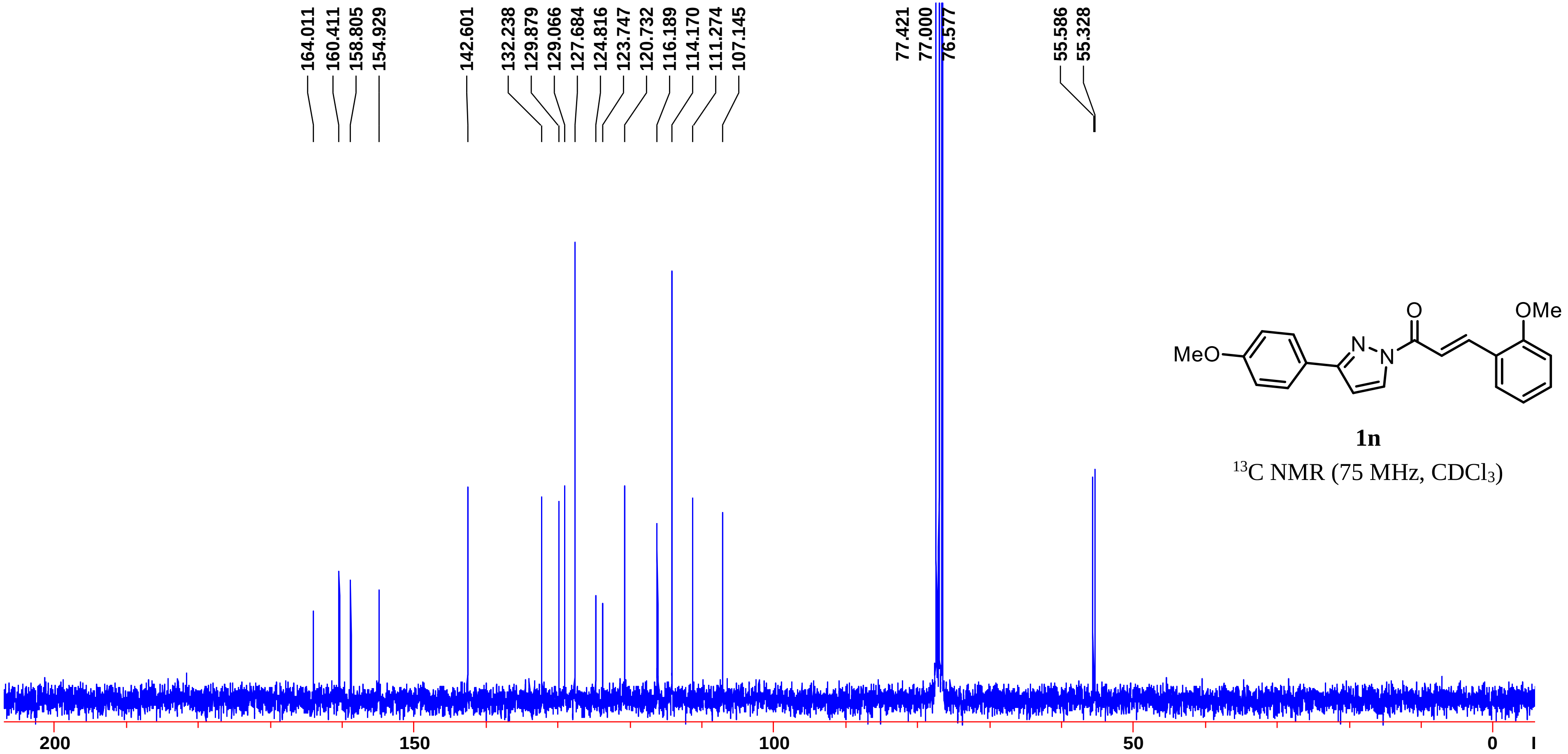

Supplementary Figure 62. ^{13}C NMR spectrum of compound **1n**.

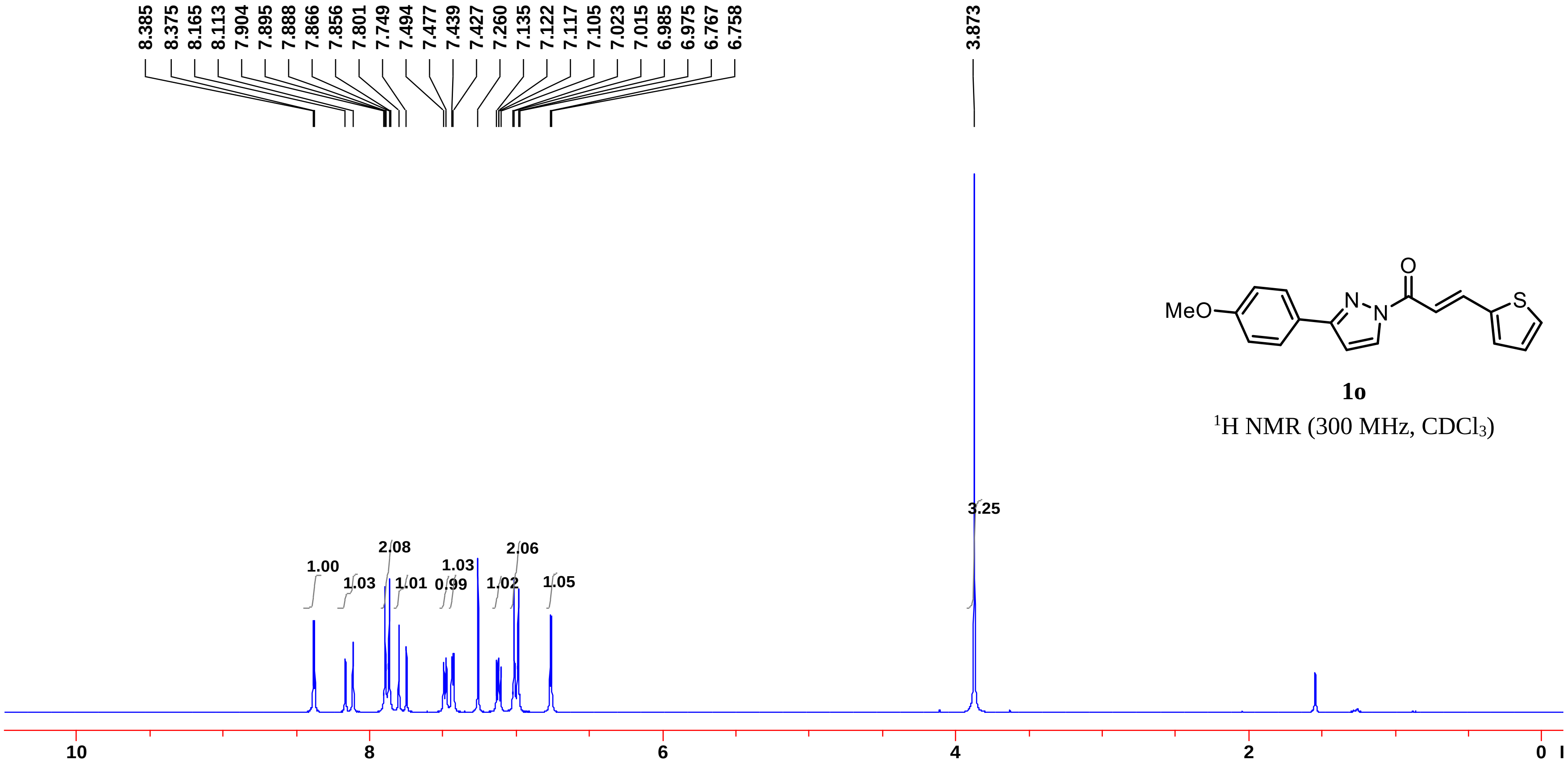

Supplementary Figure 63. ^{1}H NMR spectrum of compound **1o**.

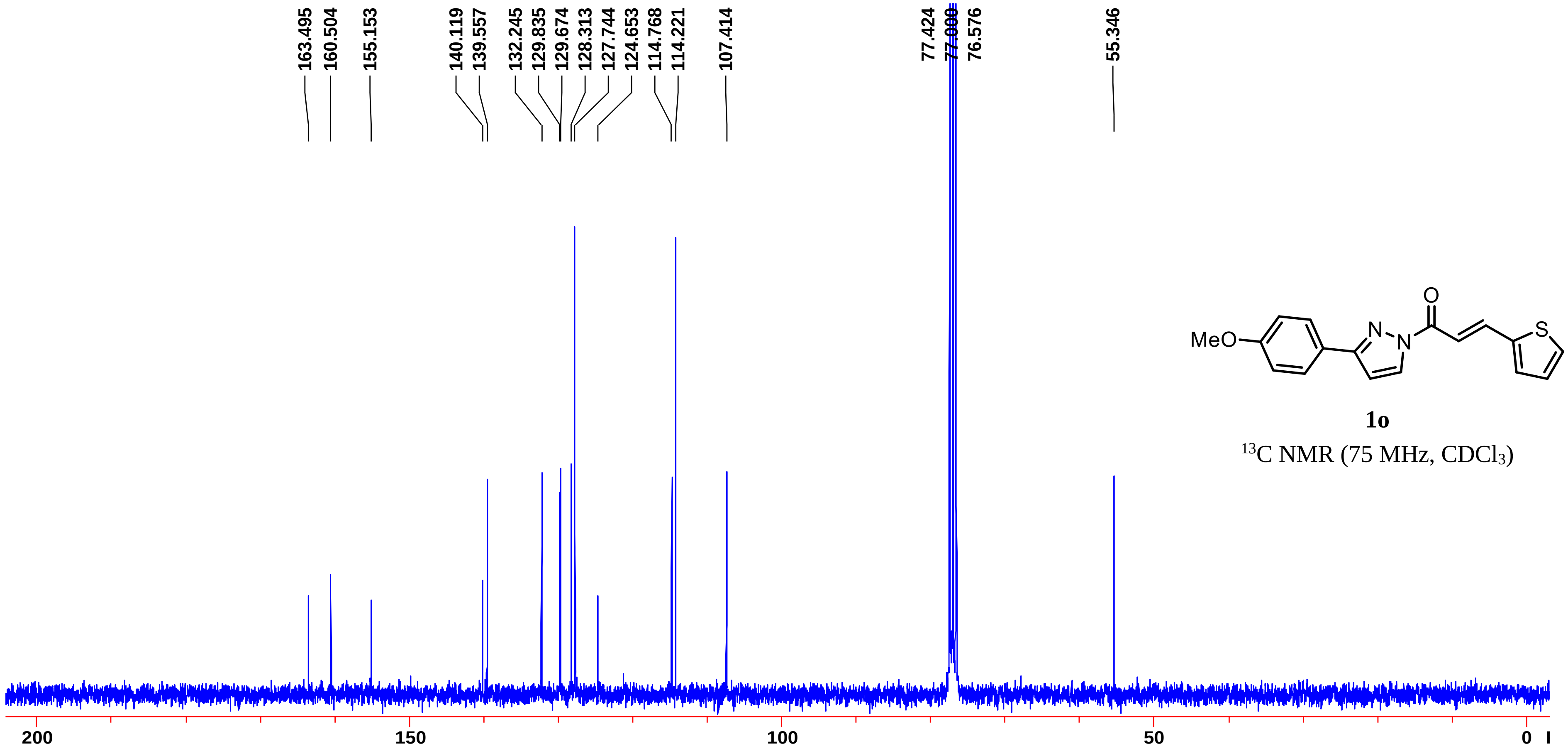

Supplementary Figure 64. ^{13}C NMR spectrum of compound **1o**.

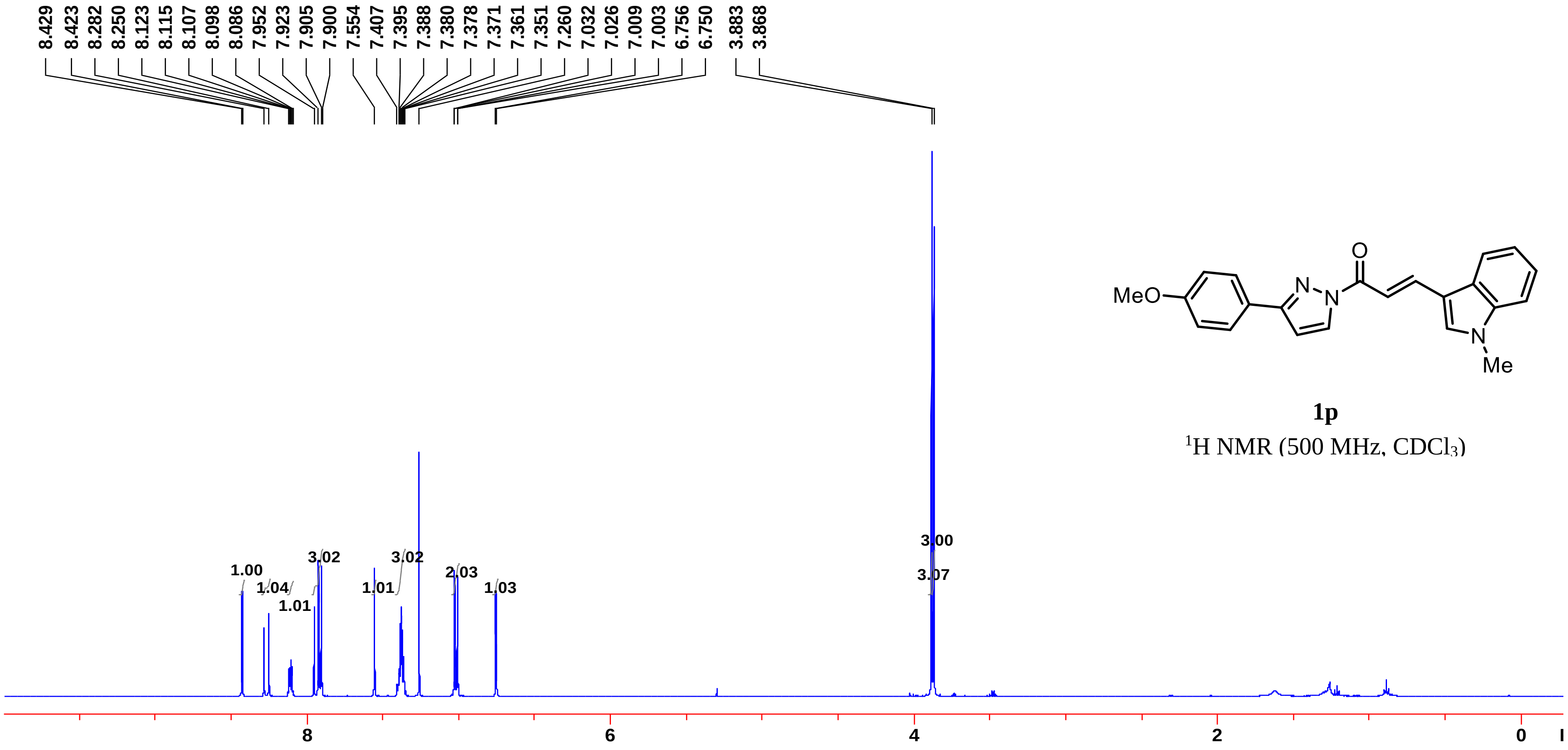

Supplementary Figure 65. ^{1}H NMR spectrum of compound **1p**.

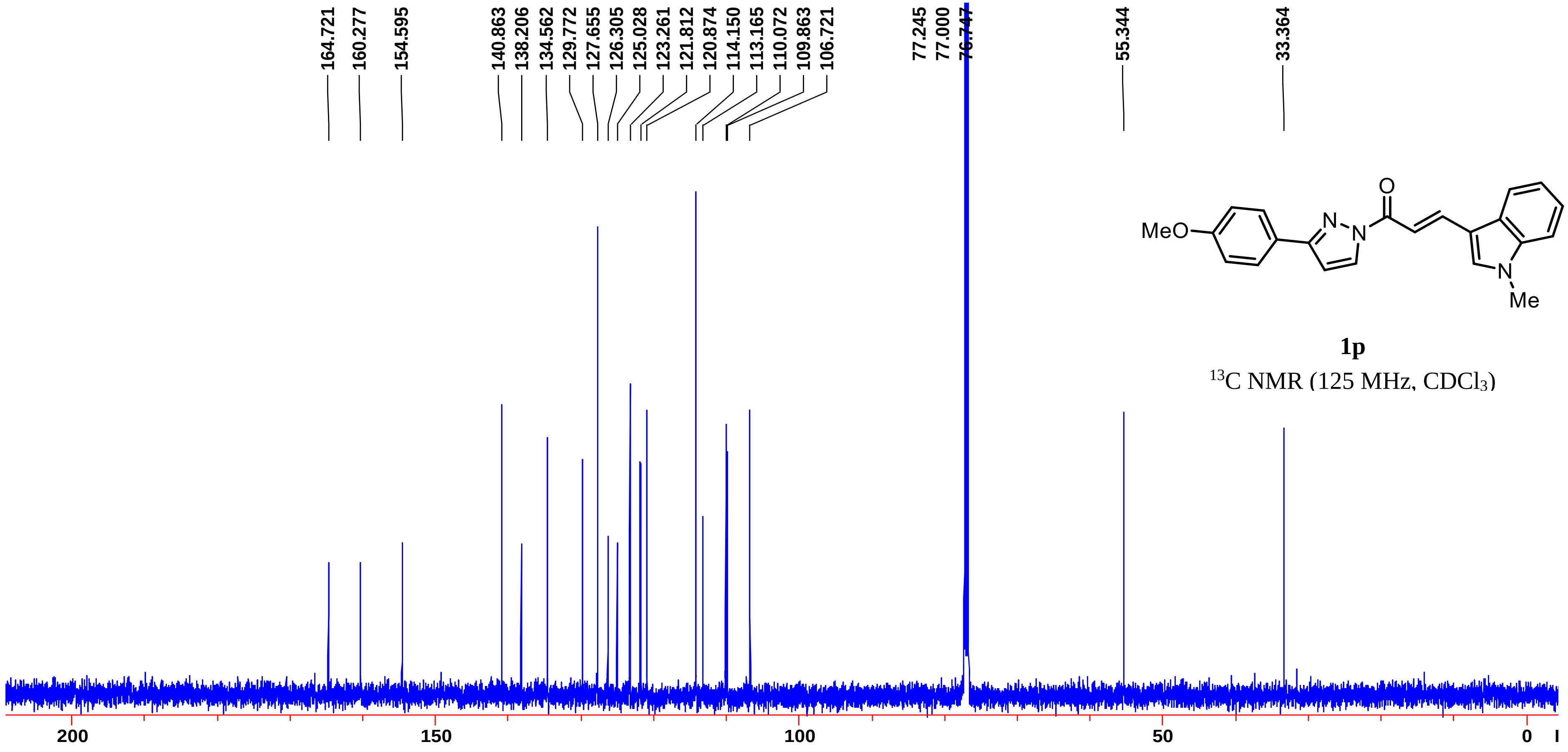

1p

^{13}C NMR (125 MHz, $CDCl_3$)

Supplementary Figure 66. ^{13}C NMR spectrum of compound **1p**.

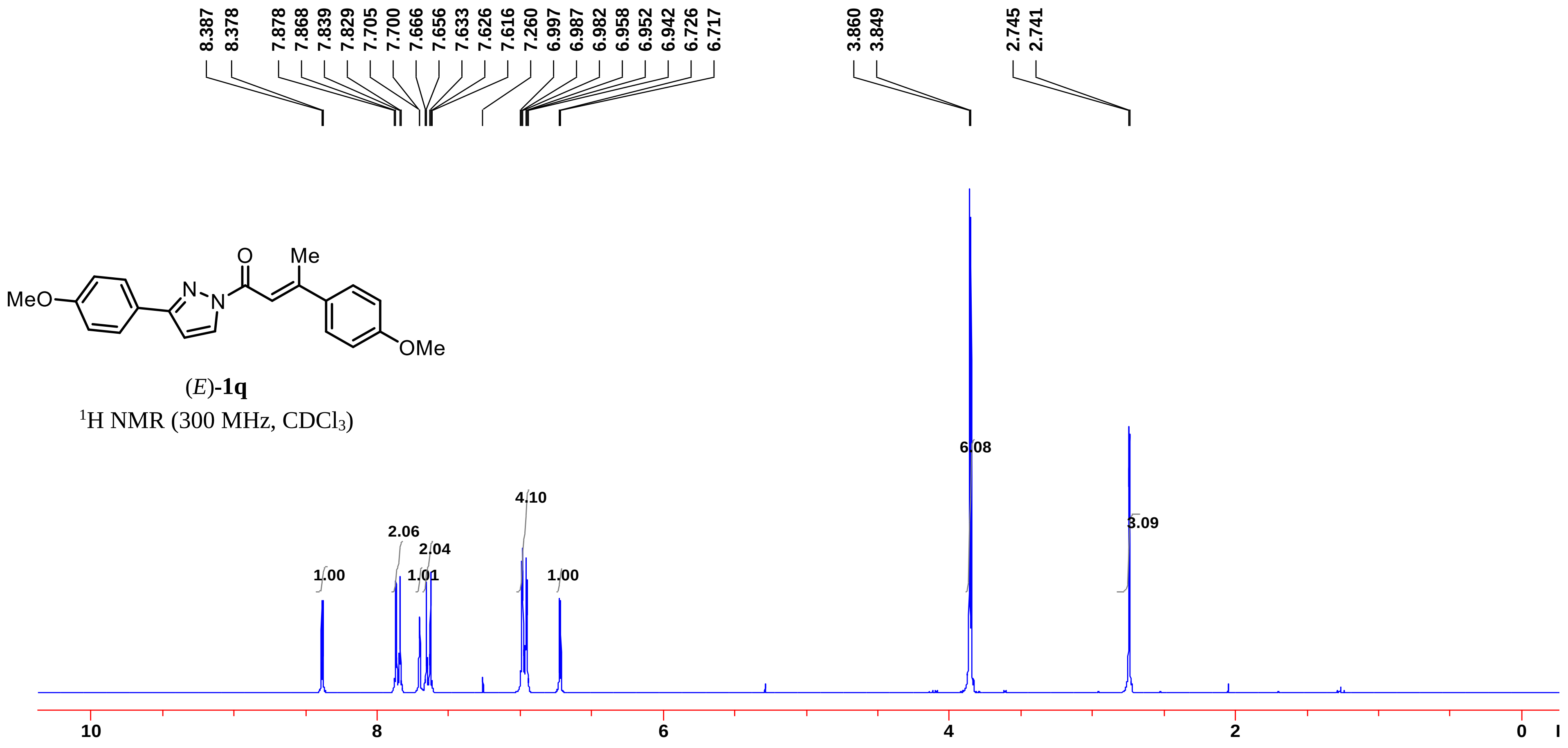

Supplementary Figure 67. ^{1}H NMR spectrum of compound (*E*)-**1q**.

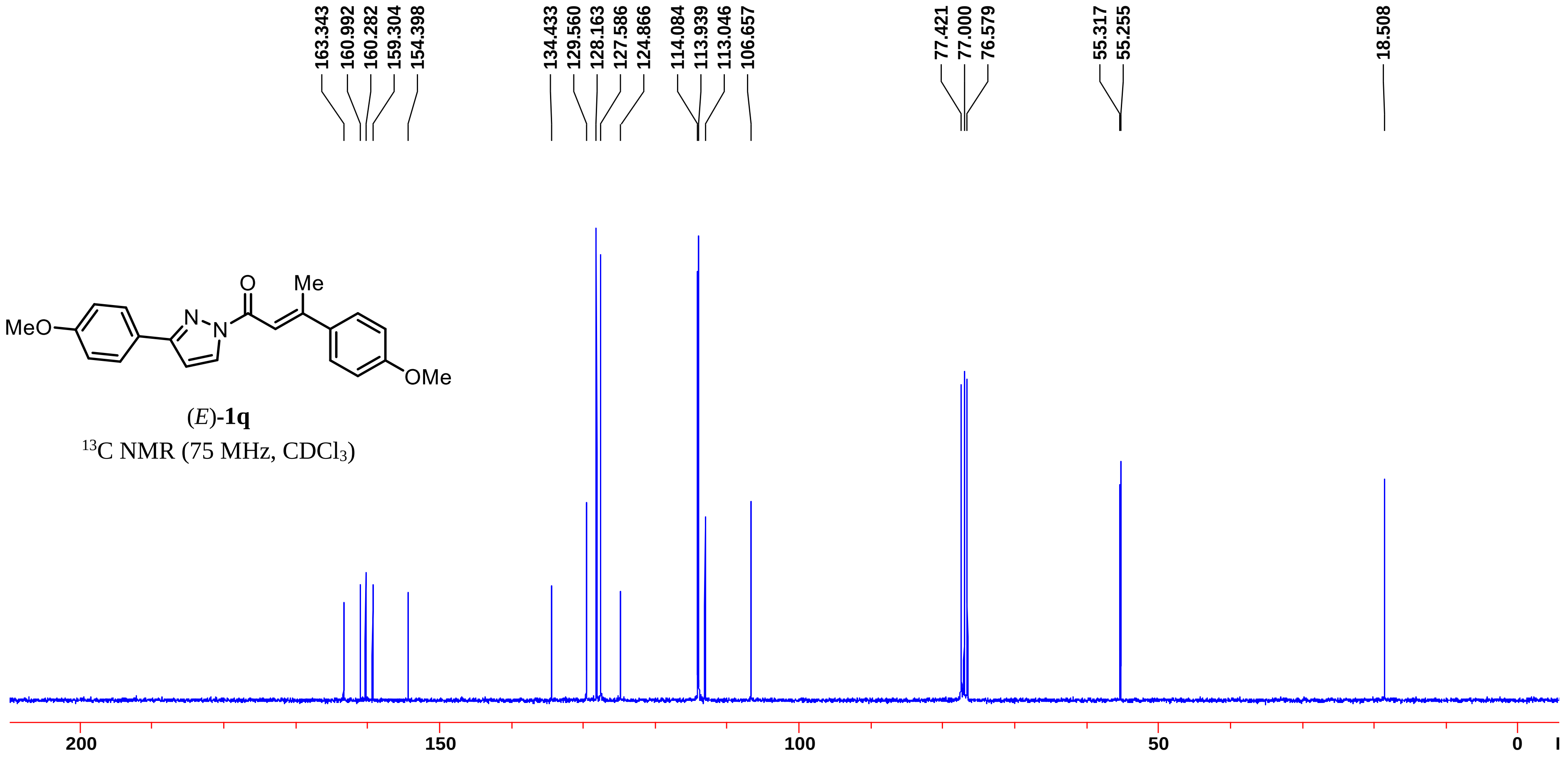

Supplementary Figure 68. ^{13}C NMR spectrum of compound (*E*)-**1q**.

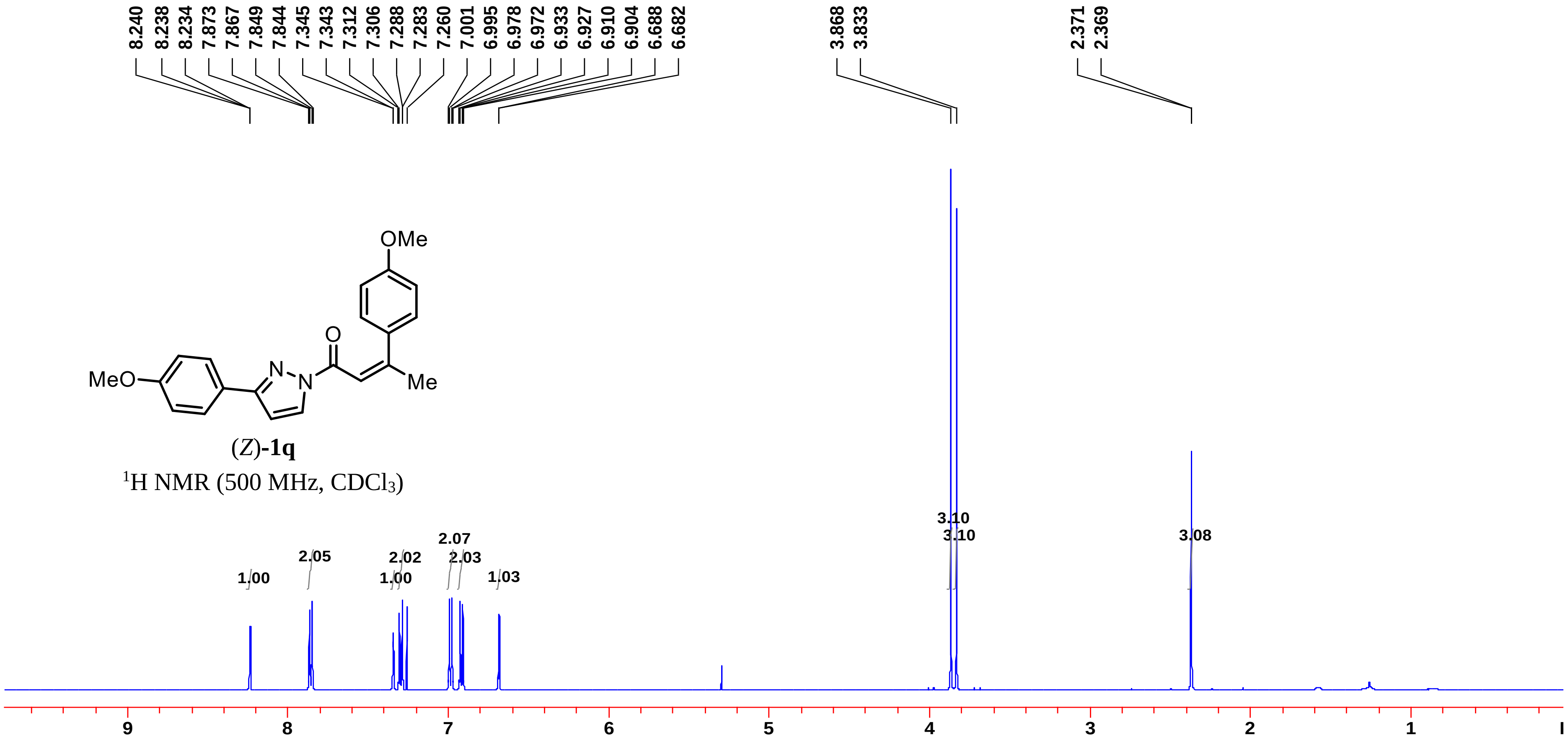

Supplementary Figure 69. ^{1}H NMR spectrum of compound (*Z*)-**1q**.

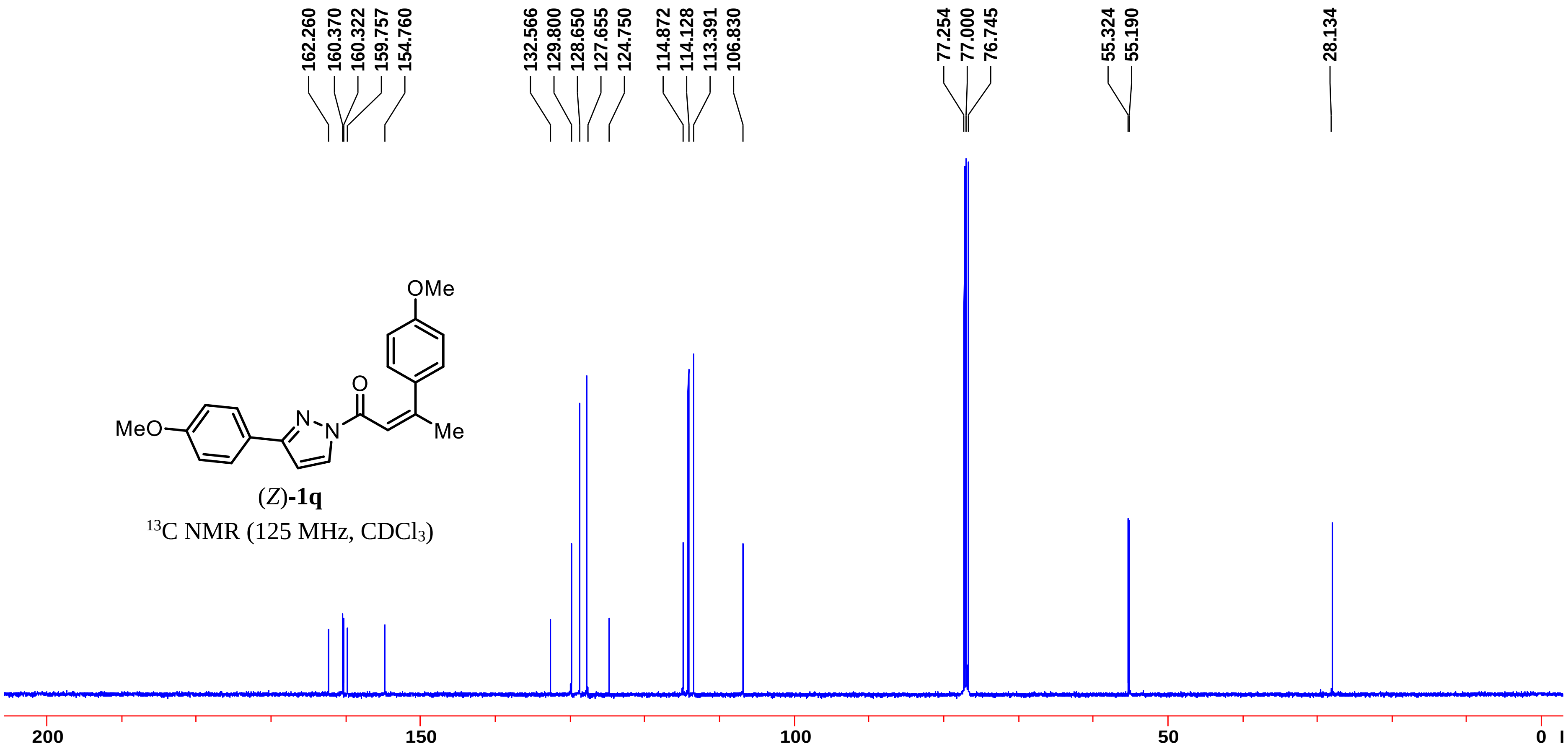

Supplementary Figure 70. ^{13}C NMR spectrum of compound (*Z*)-**1q**.

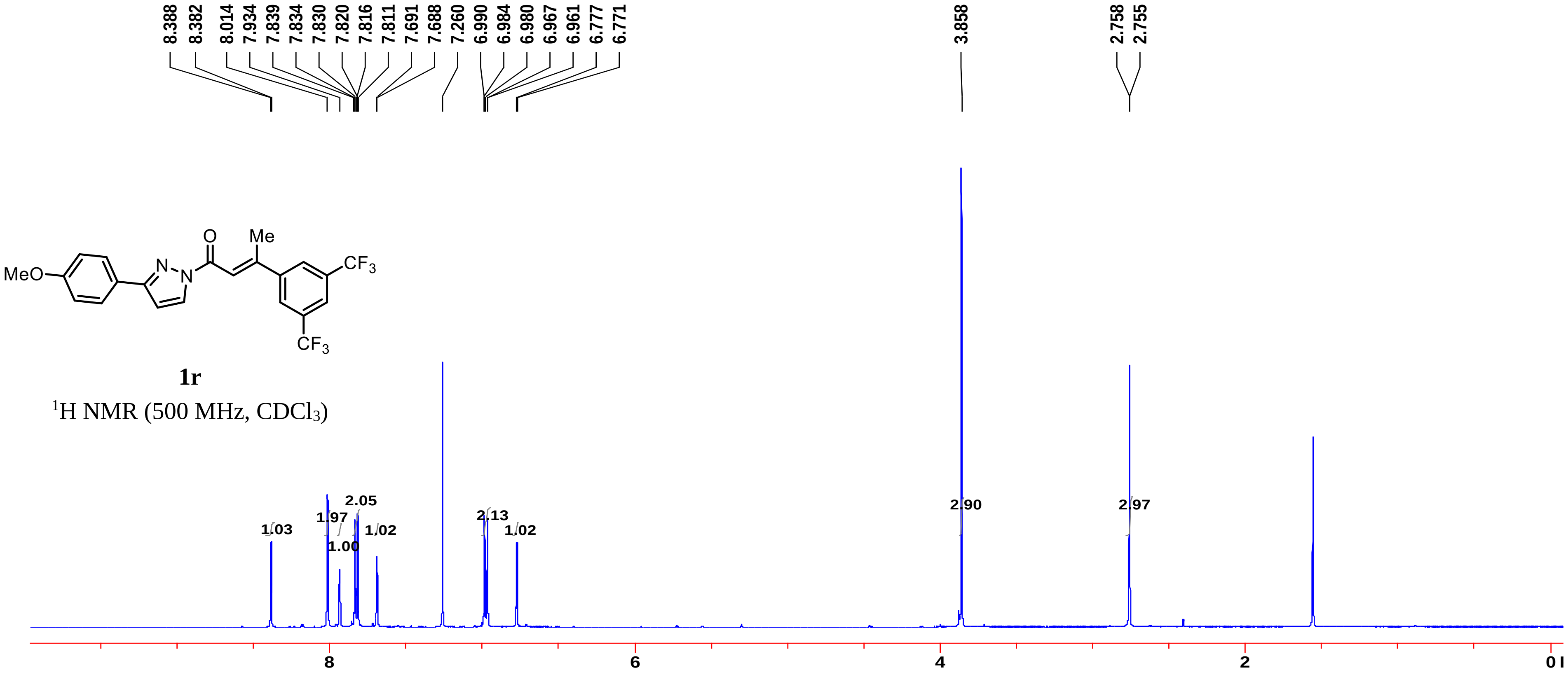

1r

^{1}H NMR (500 MHz, CDCl$_3$)

Supplementary Figure 71. ^{1}H NMR spectrum of compound **1r**.

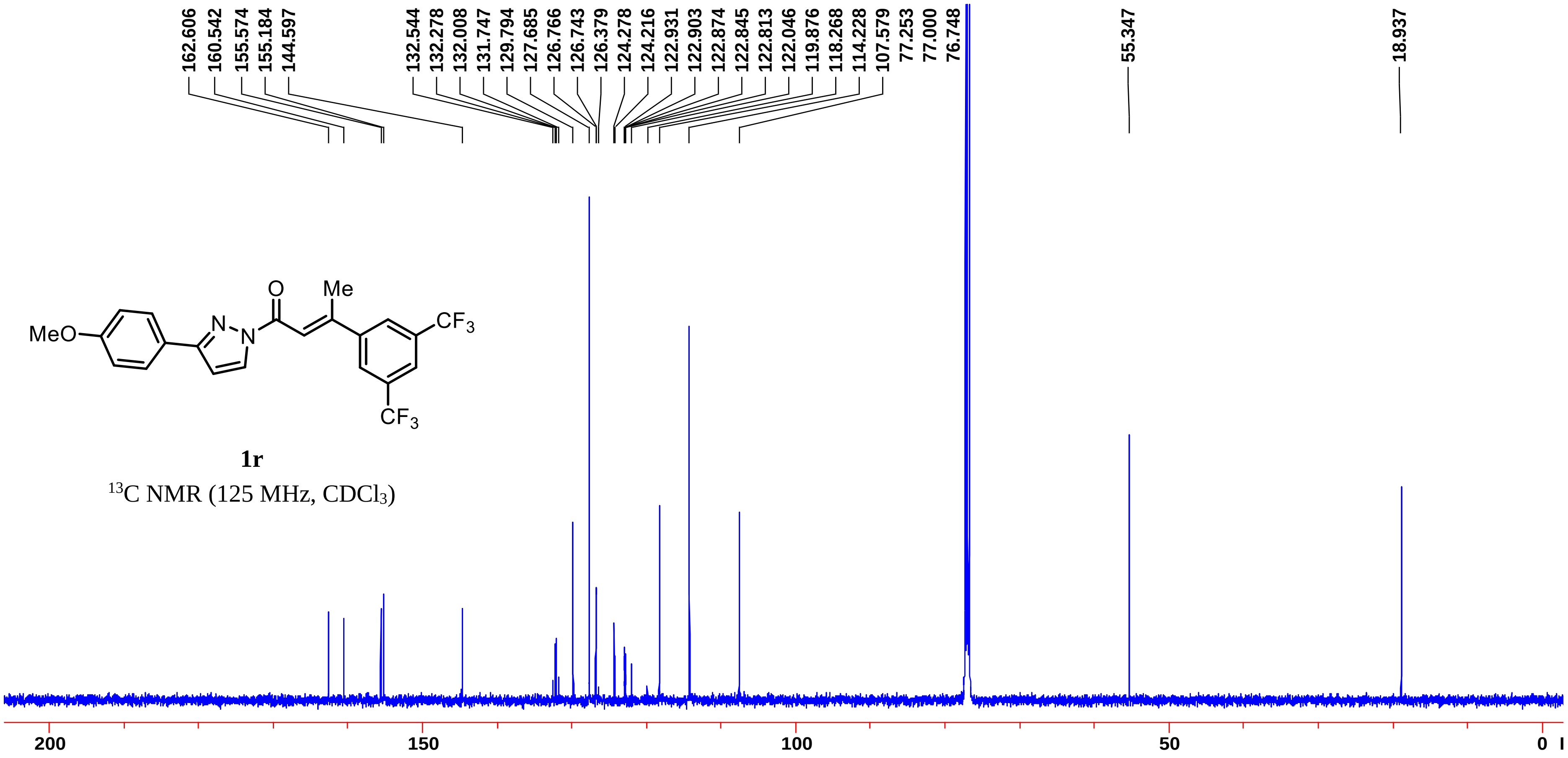

Supplementary Figure 72. ^{13}C NMR spectrum of compound **1r**.

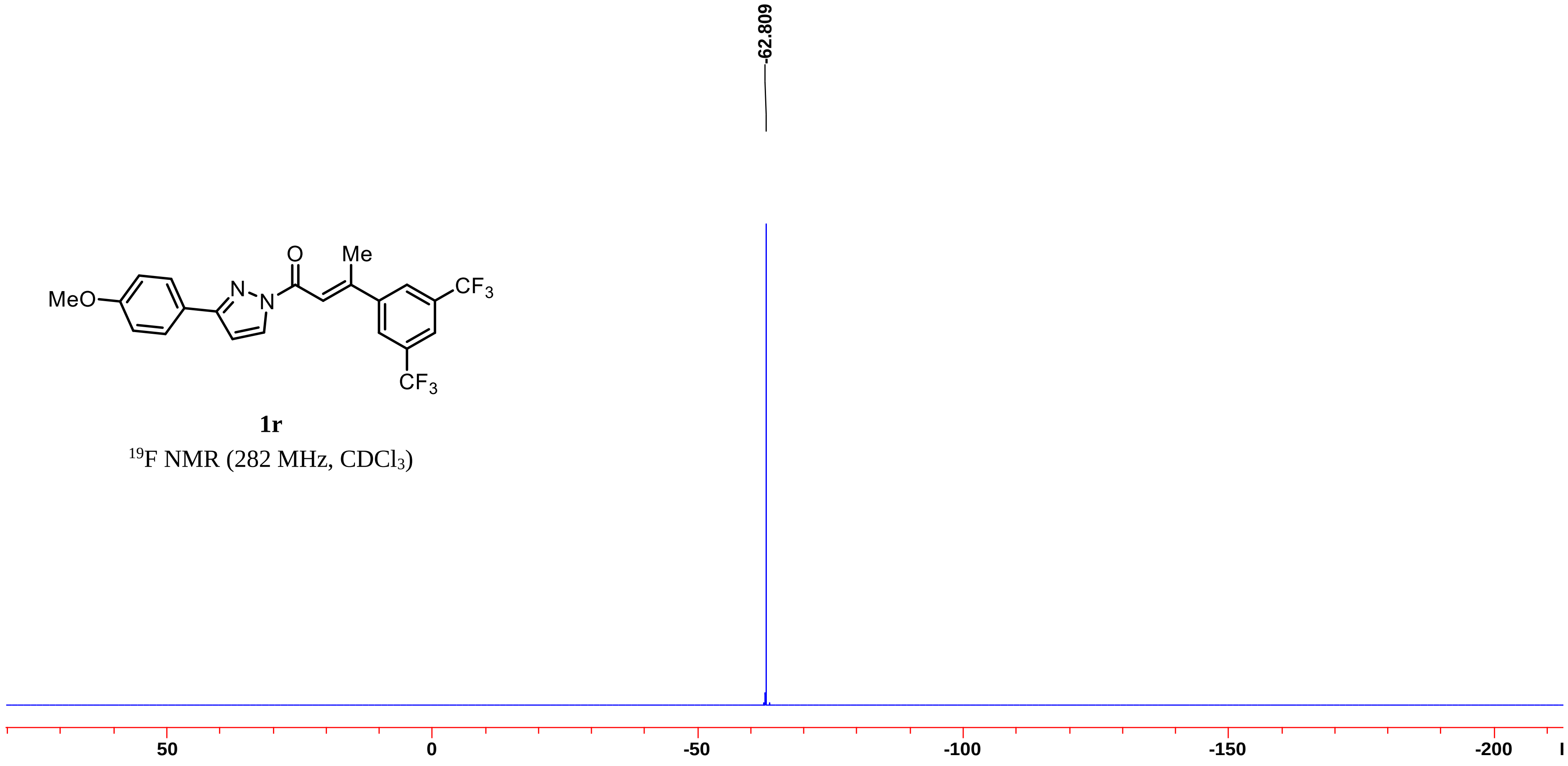

Supplementary Figure 73. ^{19}F NMR spectrum of compound **1r**.

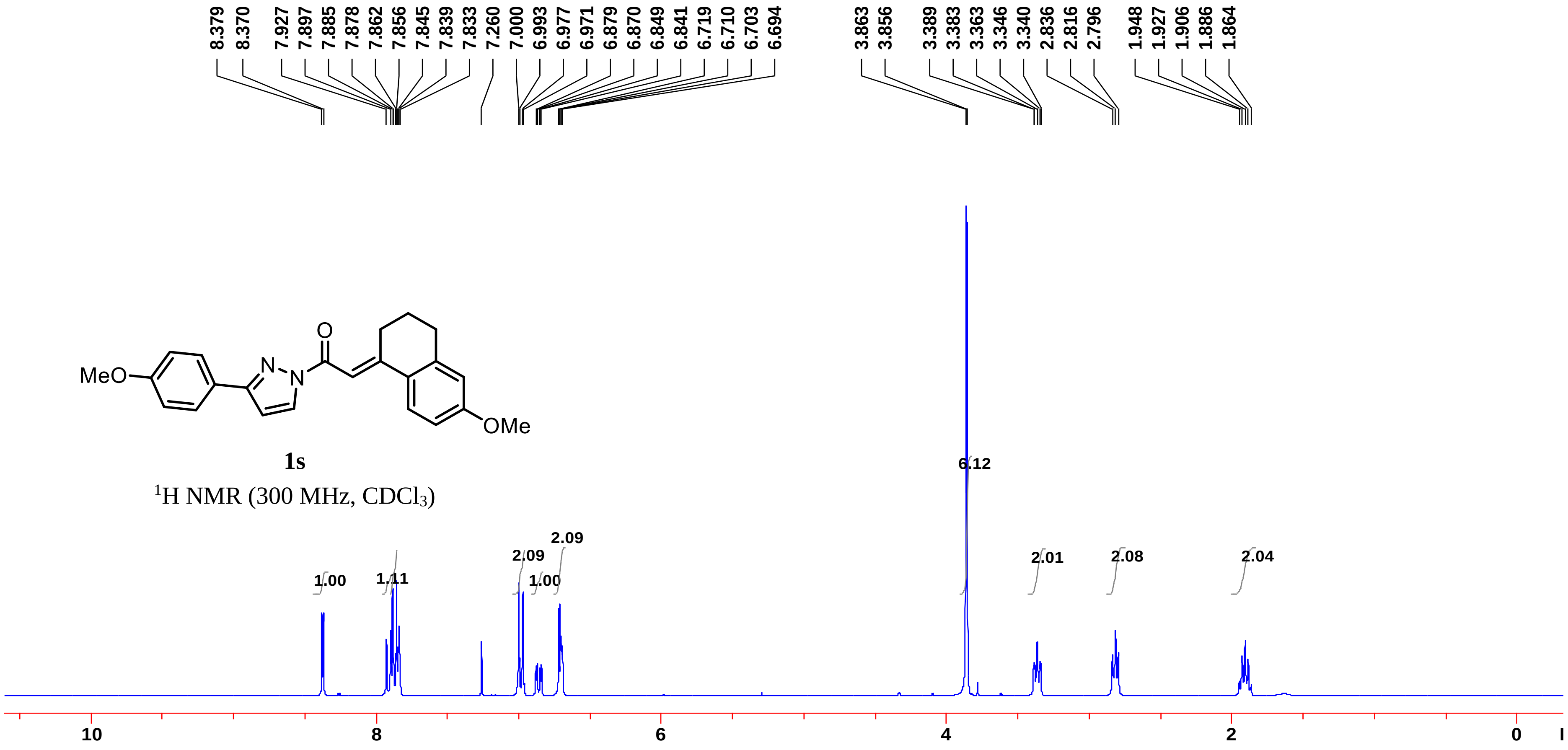

1s

^{1}H NMR (300 MHz, $CDCl_3$)

Supplementary Figure 74. ^{1}H NMR spectrum of compound **1s**.

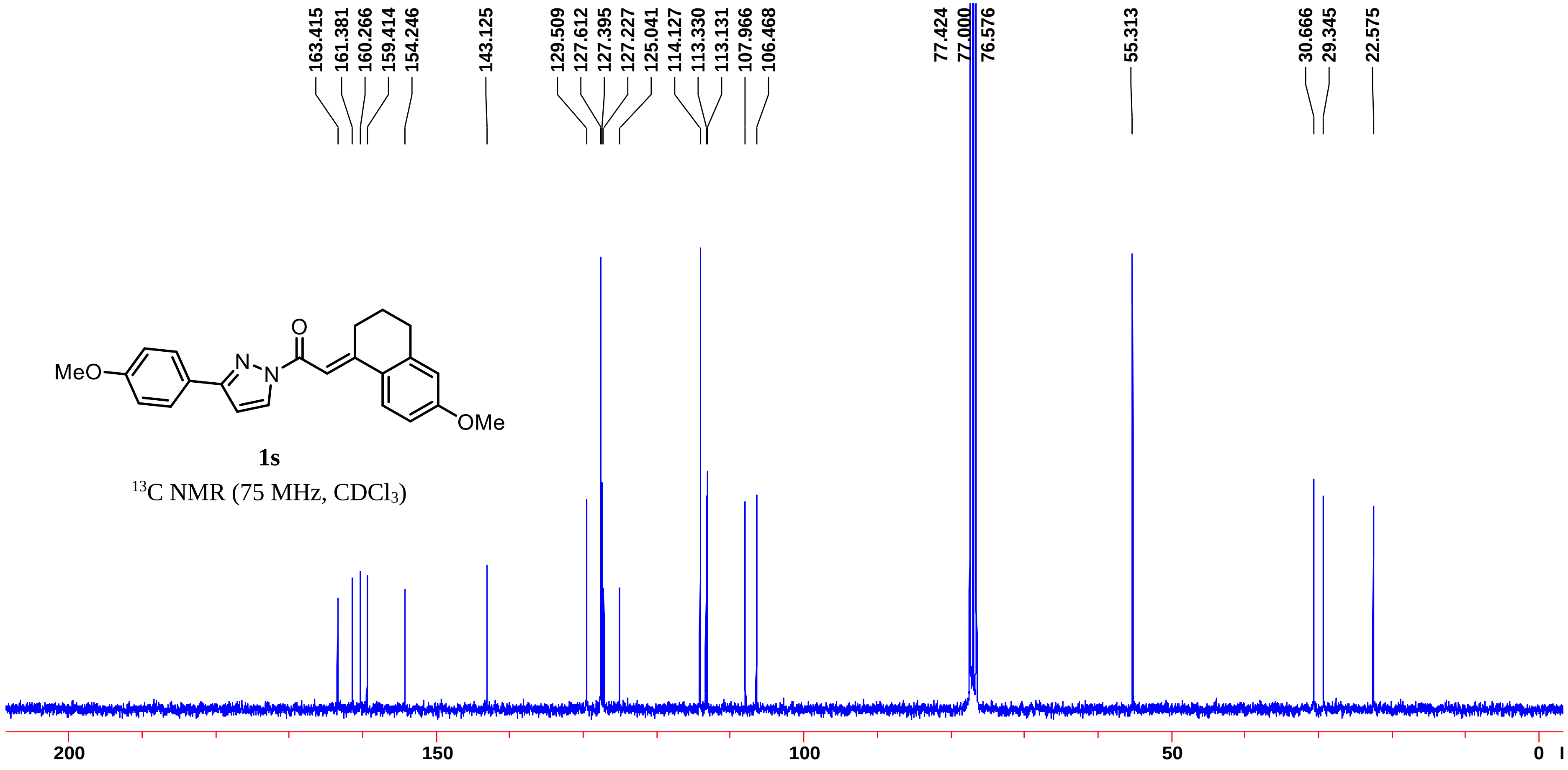

Supplementary Figure 75. ^{13}C NMR spectrum of compound **1s**.

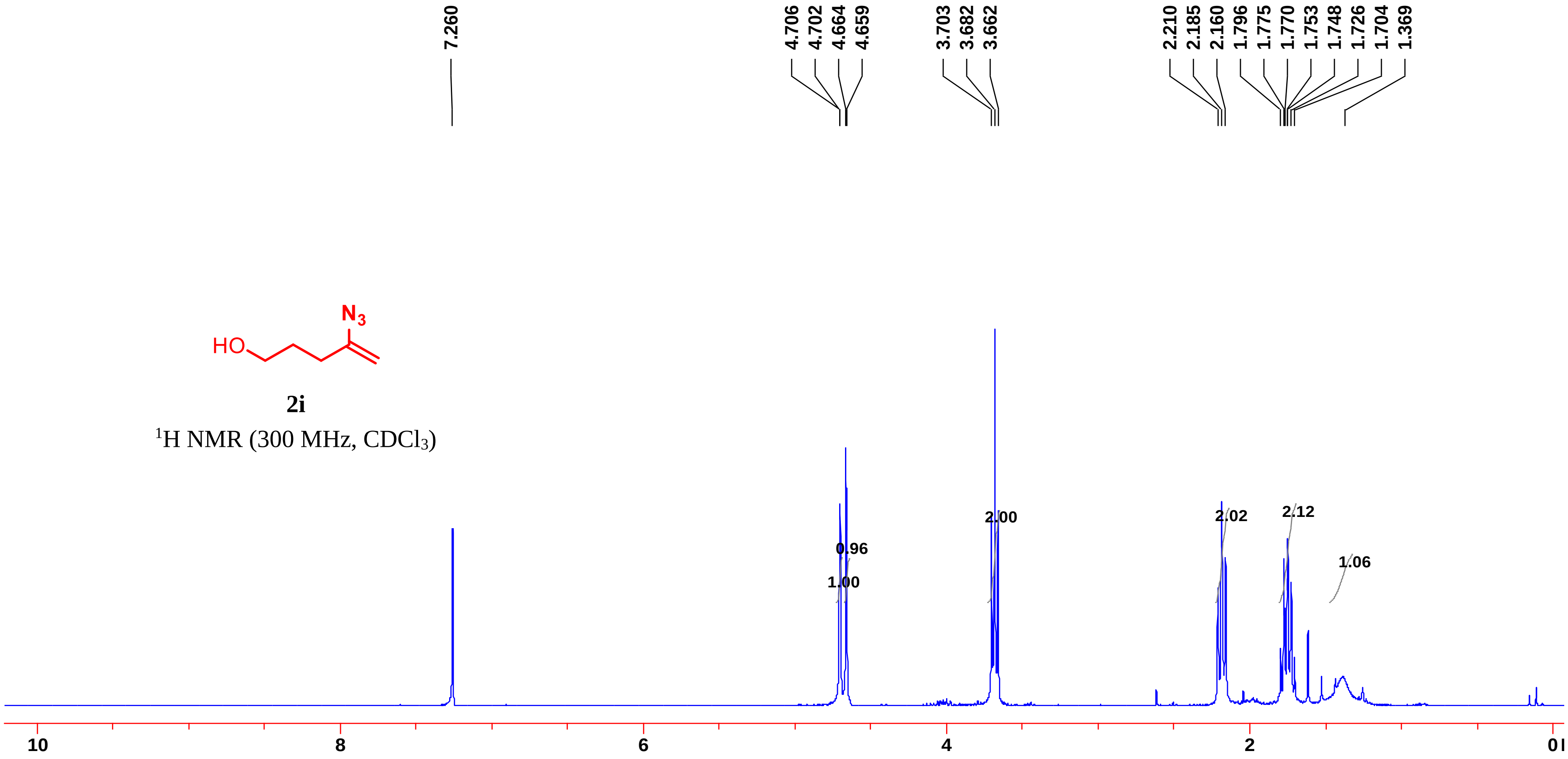

Supplementary Figure 76. ^{1}H NMR spectrum of compound **2i**.

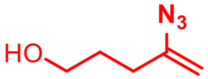

2i

^{13}C NMR (75 MHz, $CDCl_3$)

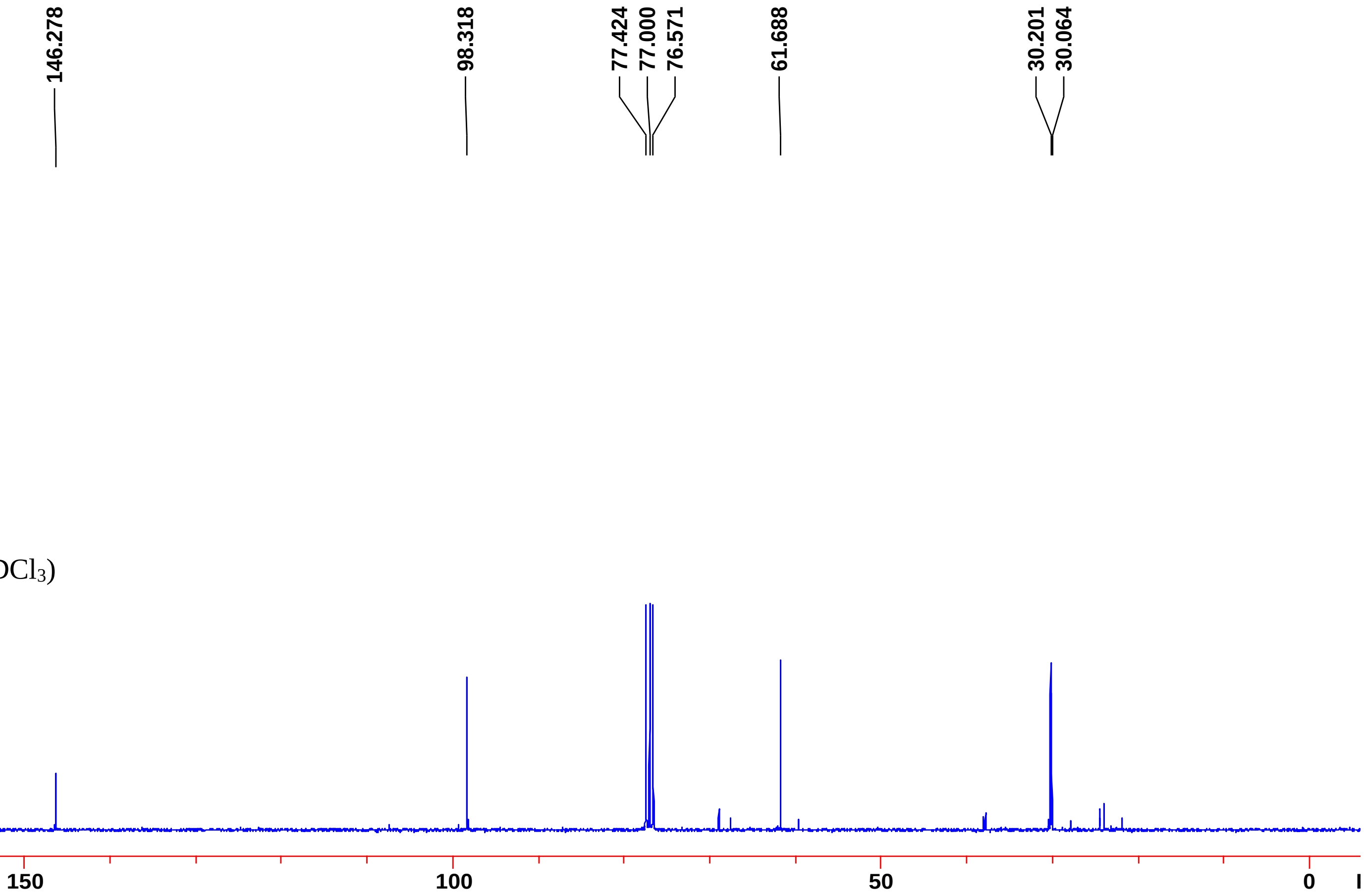

Supplementary Figure 77. ^{13}C NMR spectrum of compound **2i**.

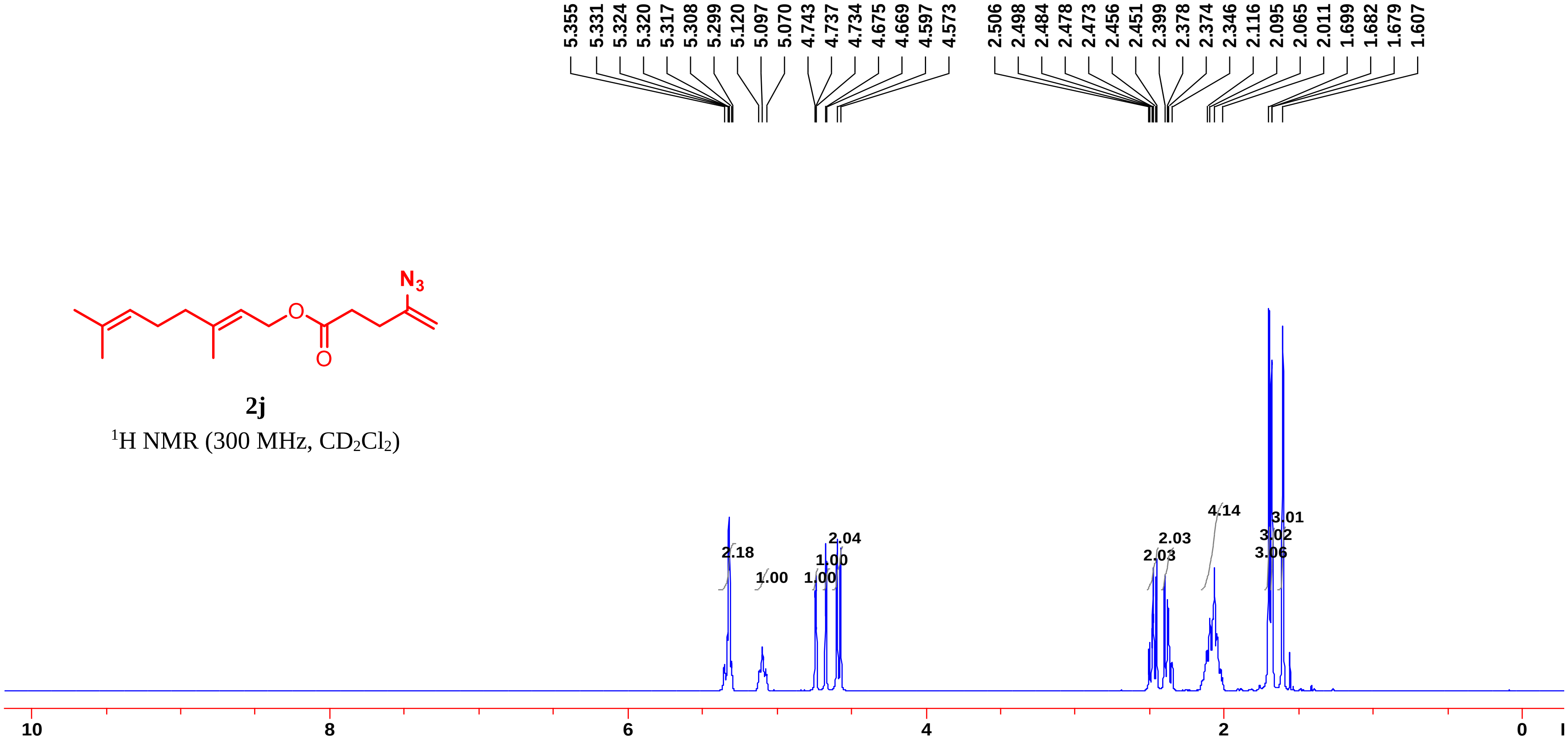

2j

^{1}H NMR (300 MHz, CD_2Cl_2)

Supplementary Figure 78. ^{1}H NMR spectrum of compound **2j**.

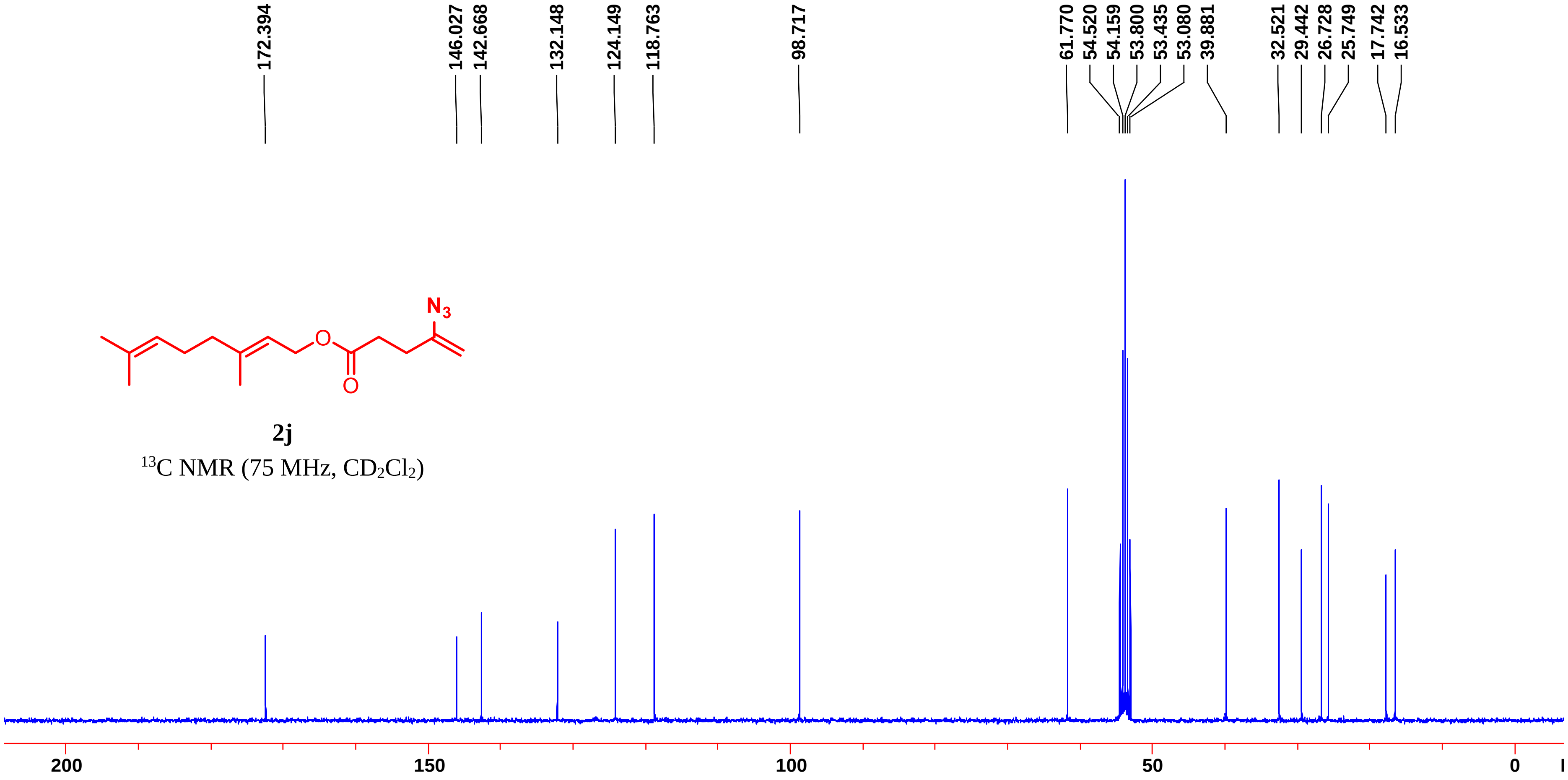

Supplementary Figure 79. ^{13}C NMR spectrum of compound **2j.**

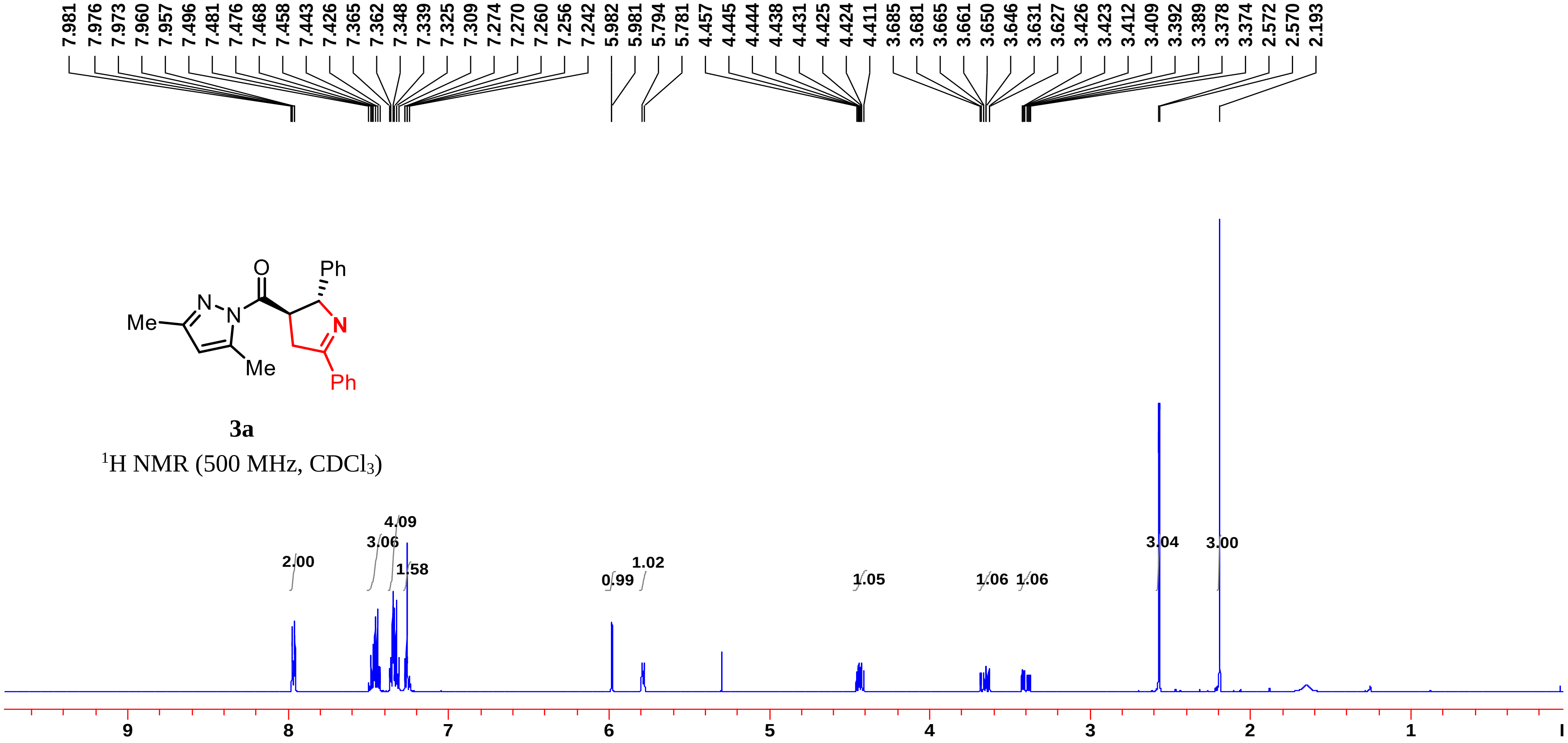

Supplementary Figure 80. ^{1}H NMR spectrum of compound **3a**.

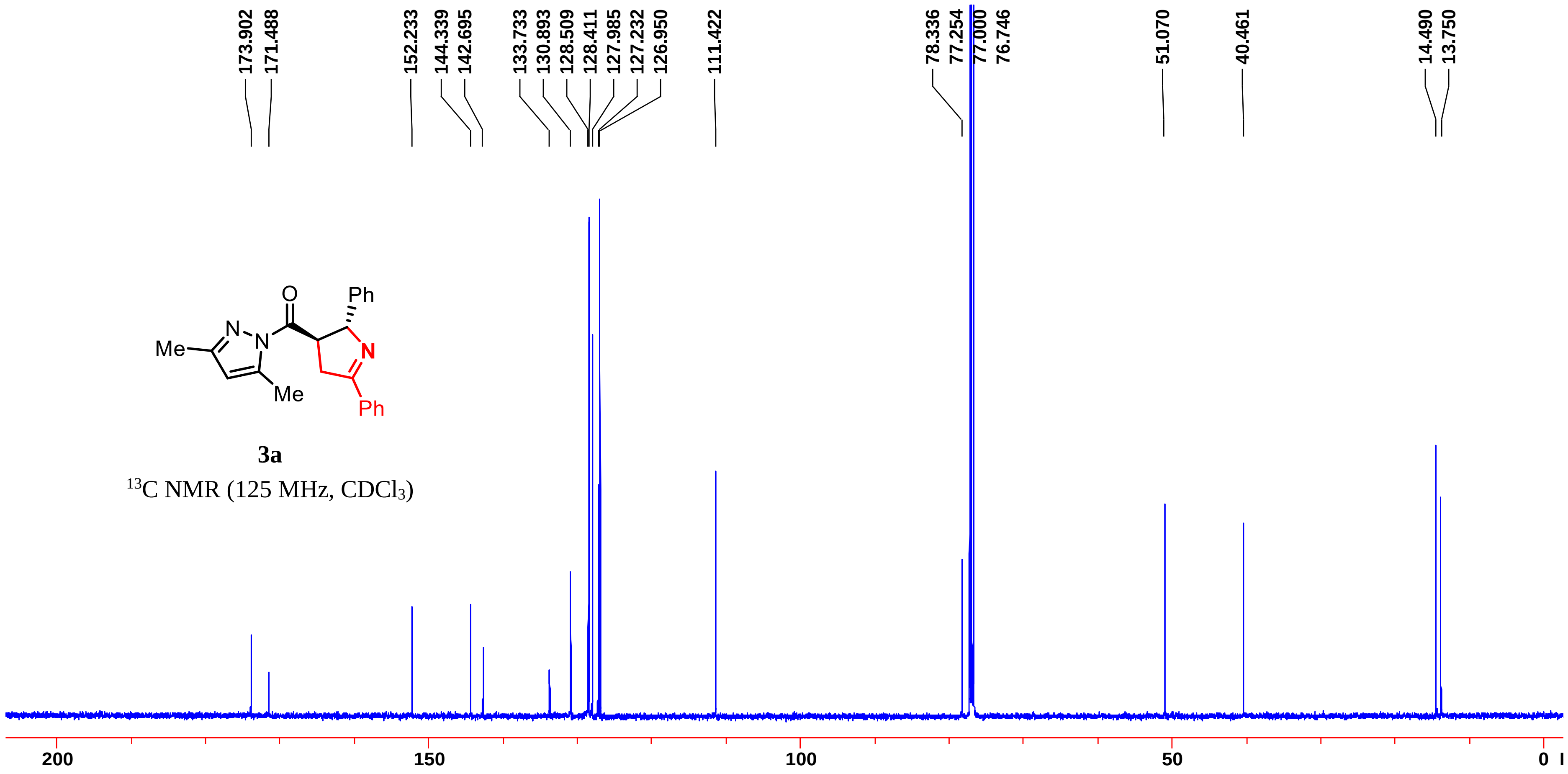

Supplementary Figure 81. ^{13}C NMR spectrum of compound **3a.**

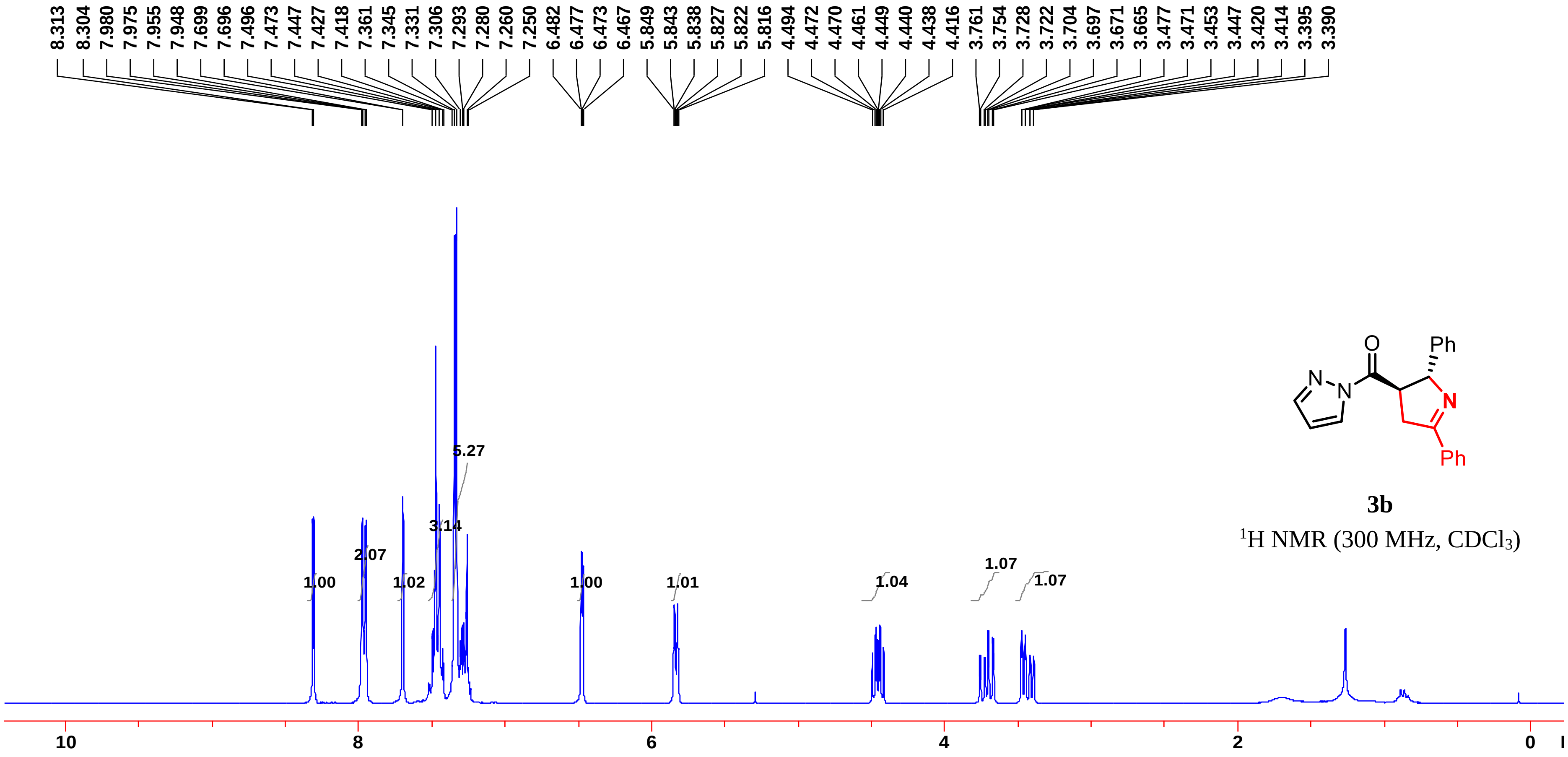

Supplementary Figure 82. ^{1}H NMR spectrum of compound **3b**.

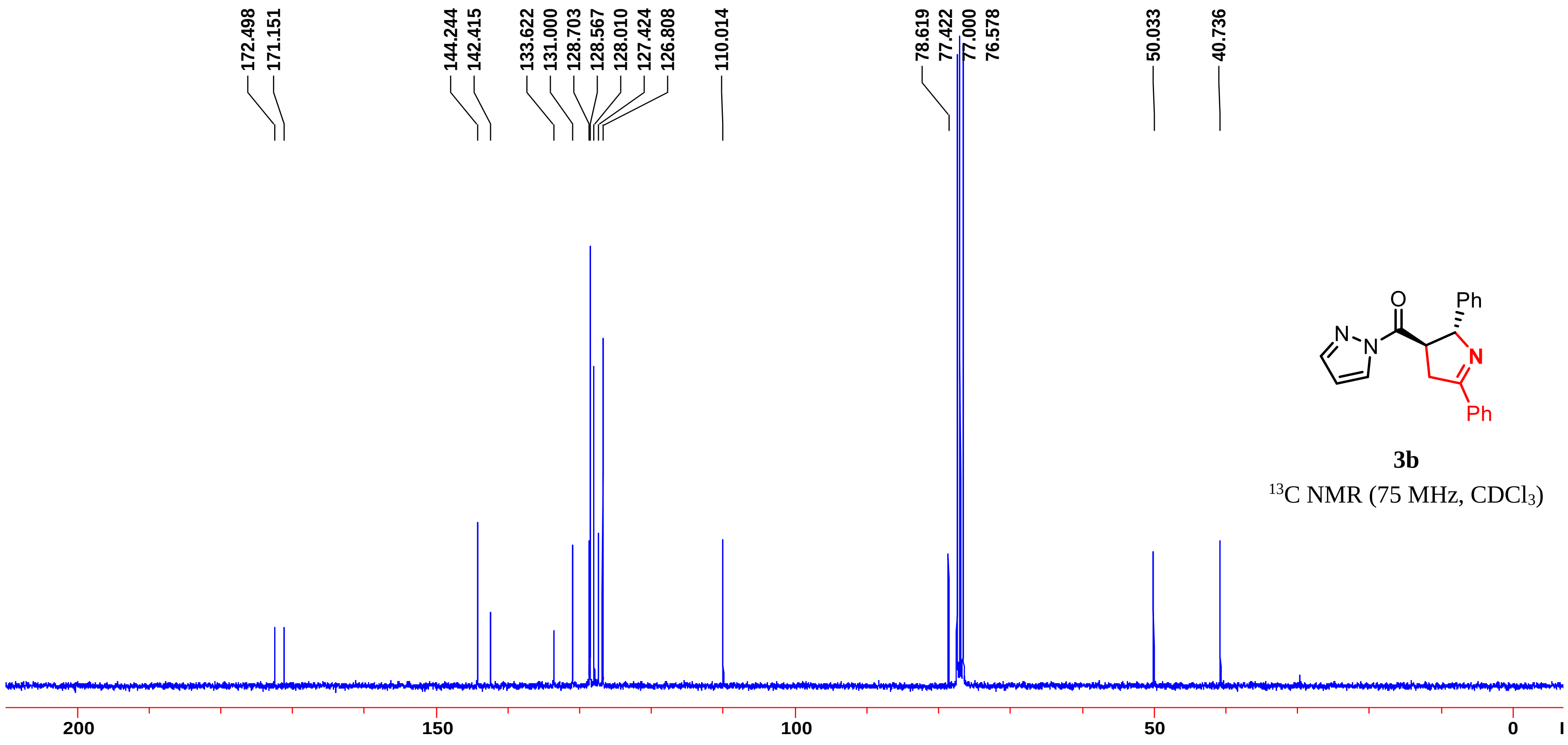

Supplementary Figure 83. ^{13}C NMR spectrum of compound **3b.**

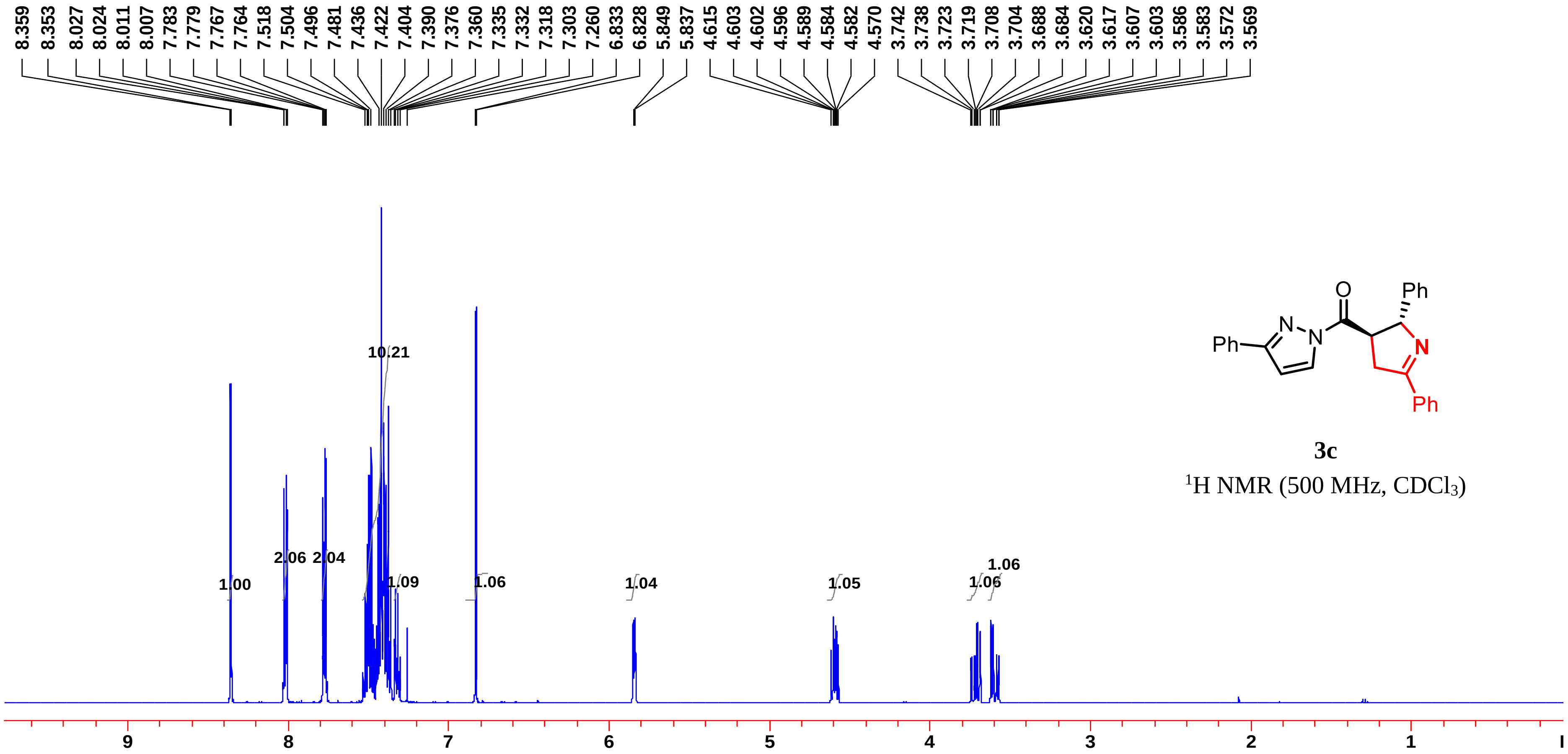

Supplementary Figure 84. ^{1}H NMR spectrum of compound **3c**.

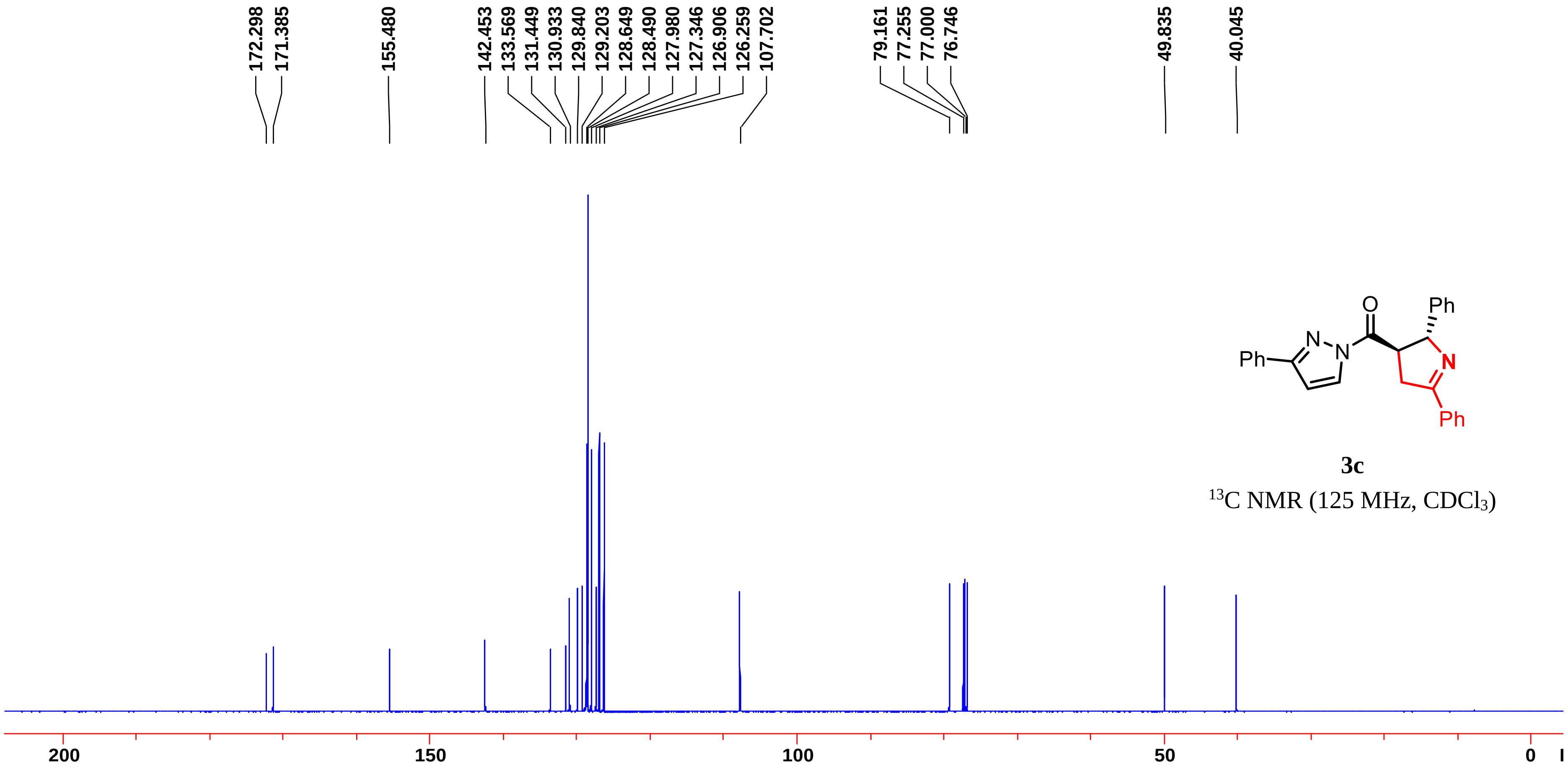

Supplementary Figure 85. ^{13}C NMR spectrum of compound **3c**.

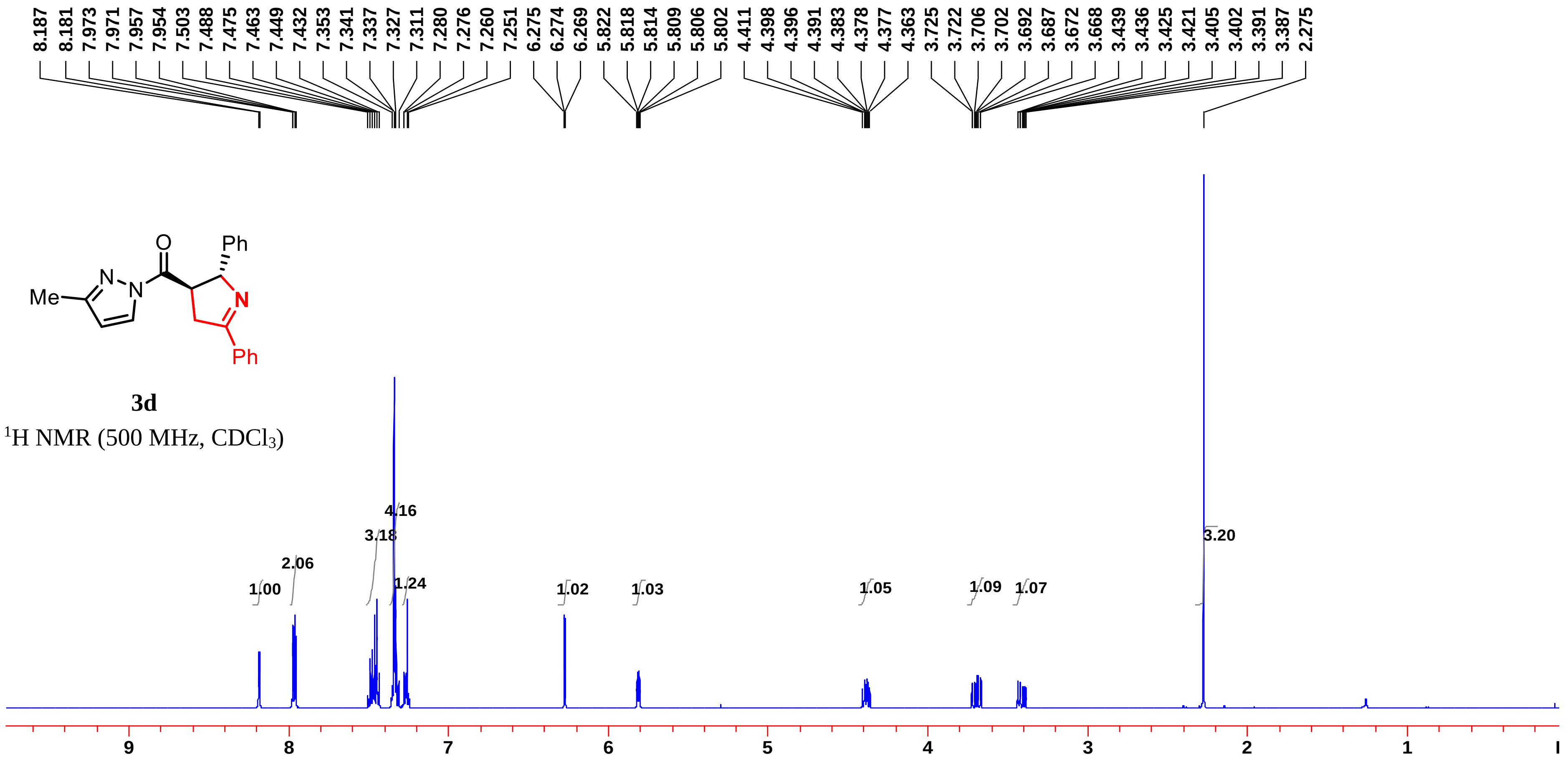

Supplementary Figure 86. ^{1}H NMR spectrum of compound **3d**.

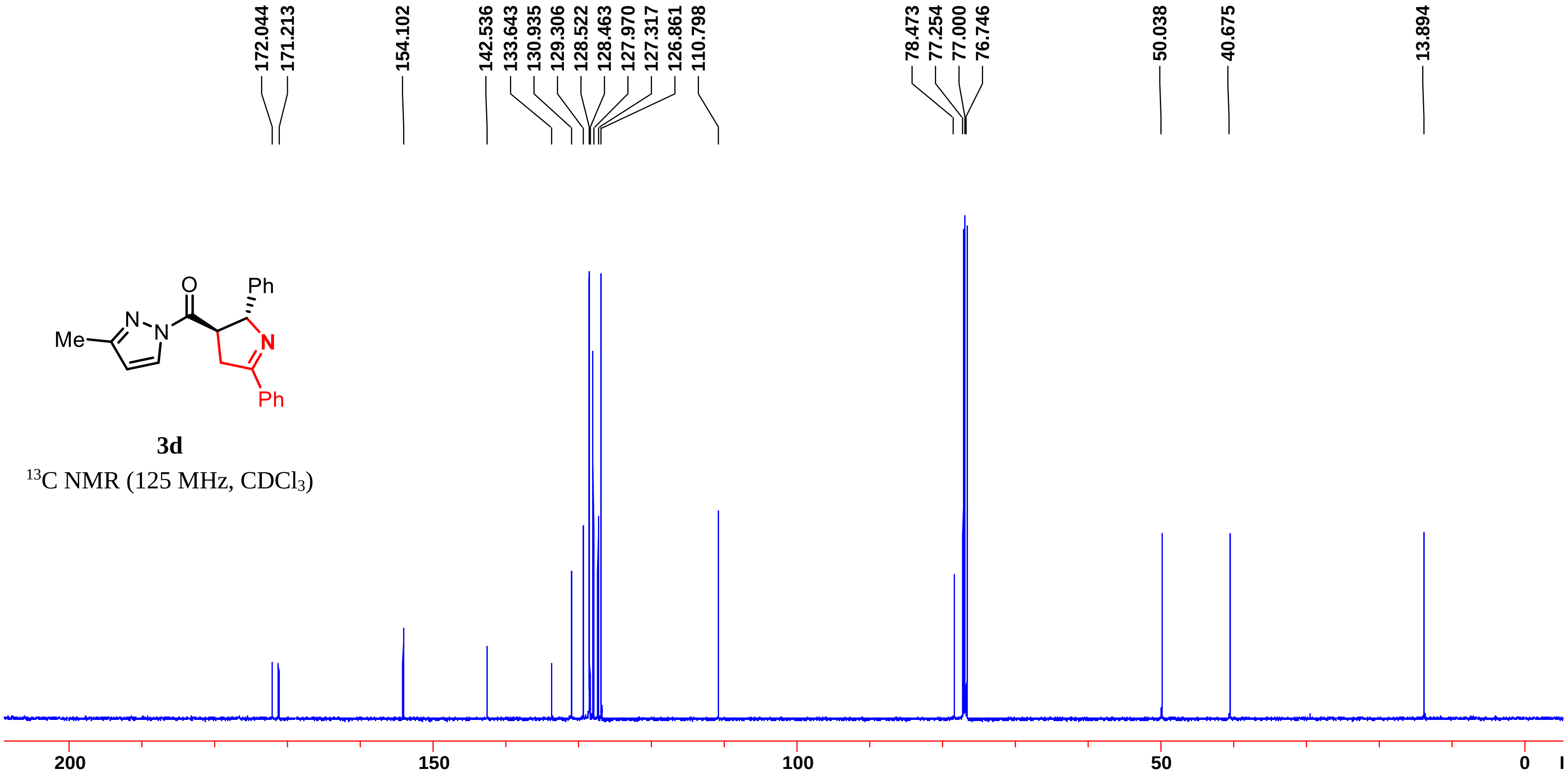

Supplementary Figure 87. ^{13}C NMR spectrum of compound **3d.**

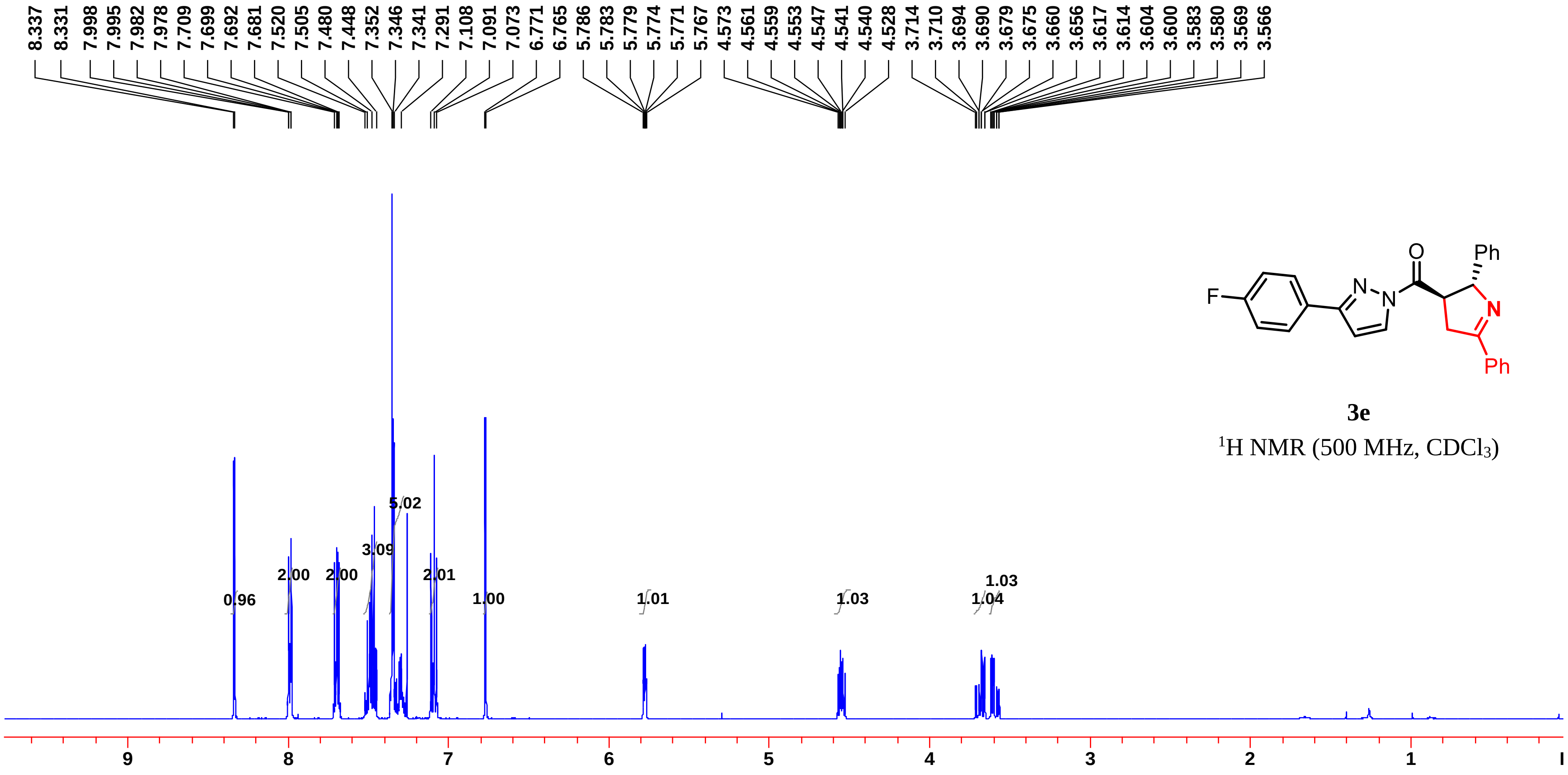

Supplementary Figure 88. ^{1}H NMR spectrum of compound **3e**.

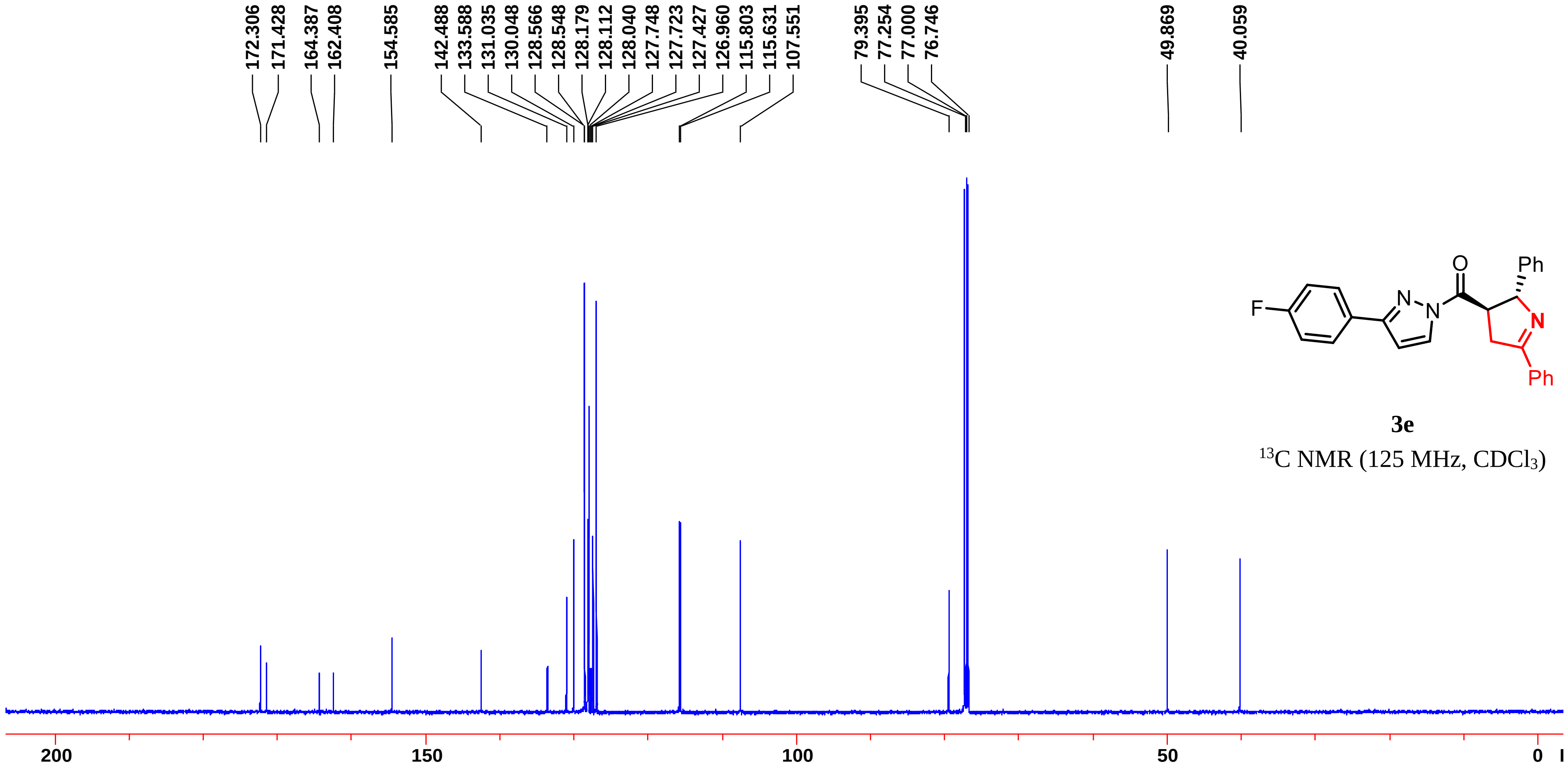

Supplementary Figure 89. ^{13}C NMR spectrum of compound **3e.**

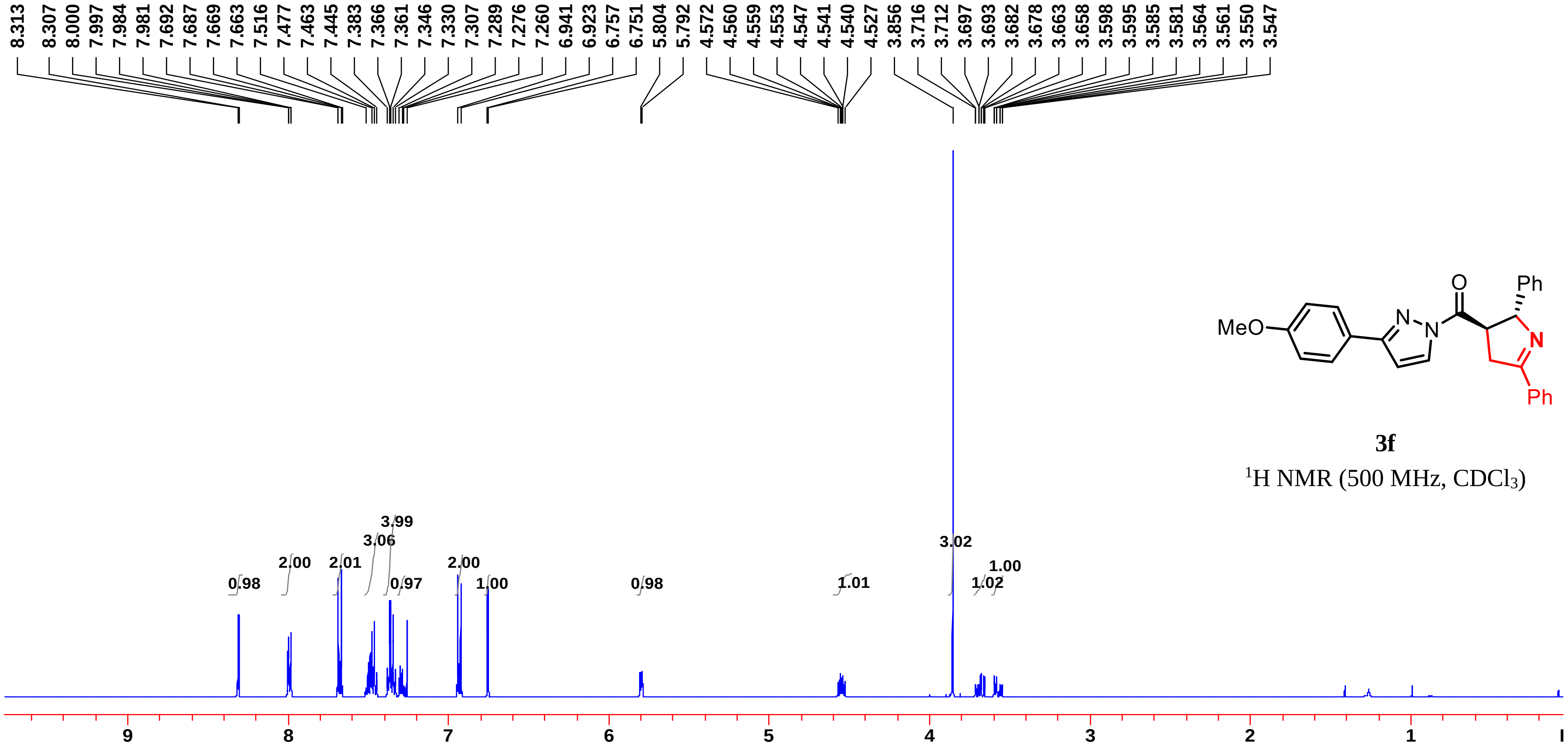

3f

^{1}H NMR (500 MHz, $CDCl_3$)

Supplementary Figure 90. ^{1}H NMR spectrum of compound **3f**.

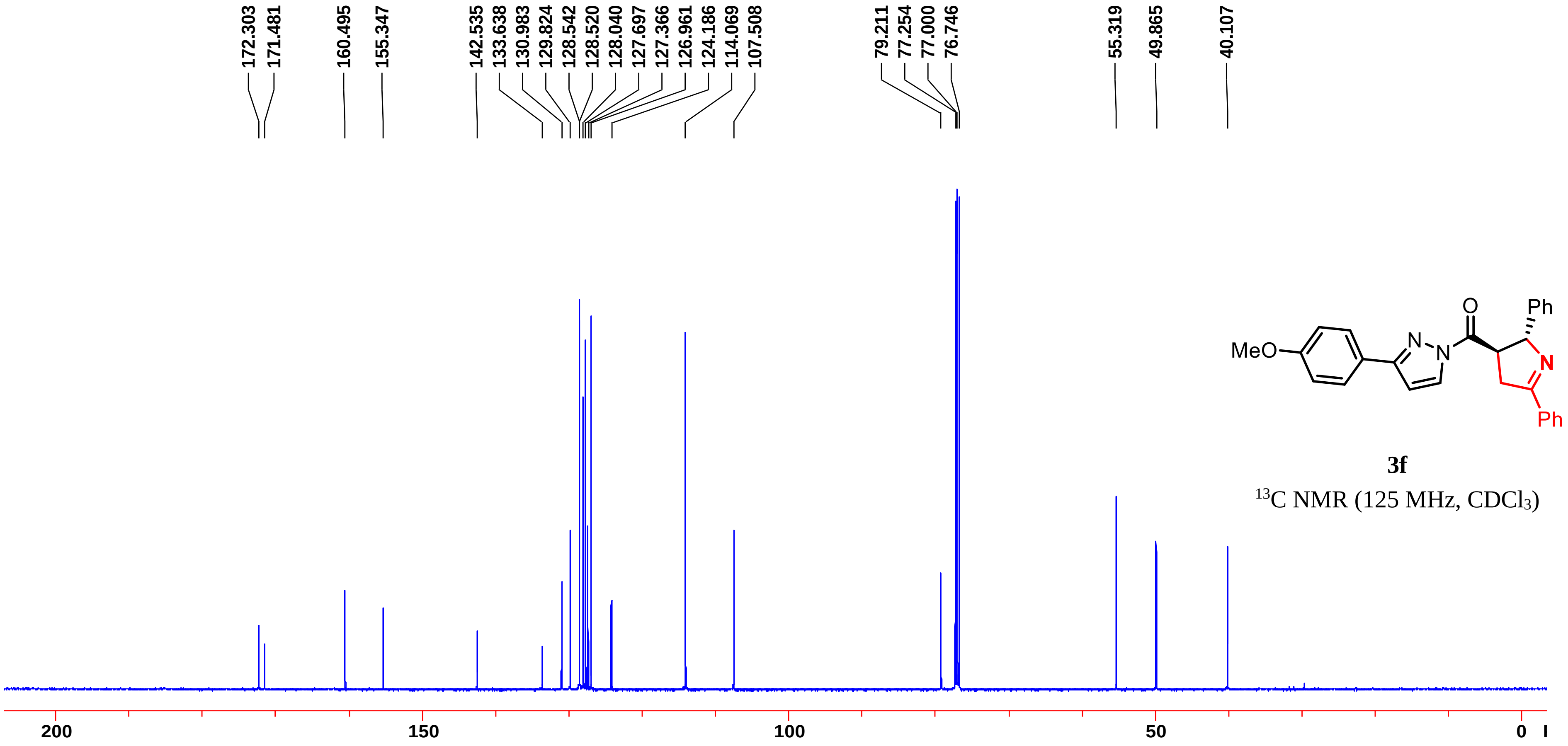

Supplementary Figure 91. ^{13}C NMR spectrum of compound **3f**.

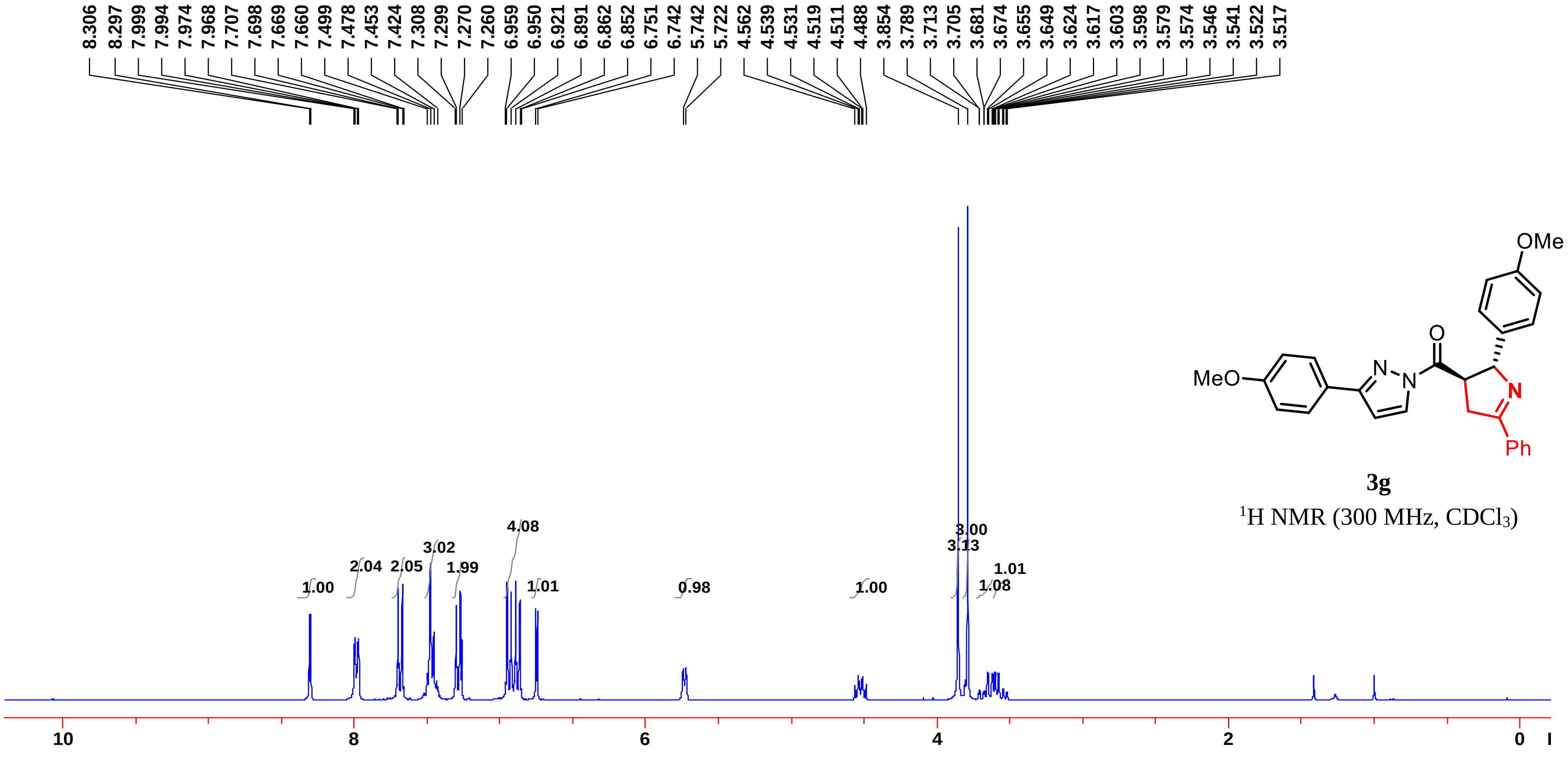

Supplementary Figure 92. ^{1}H NMR spectrum of compound **3g**.

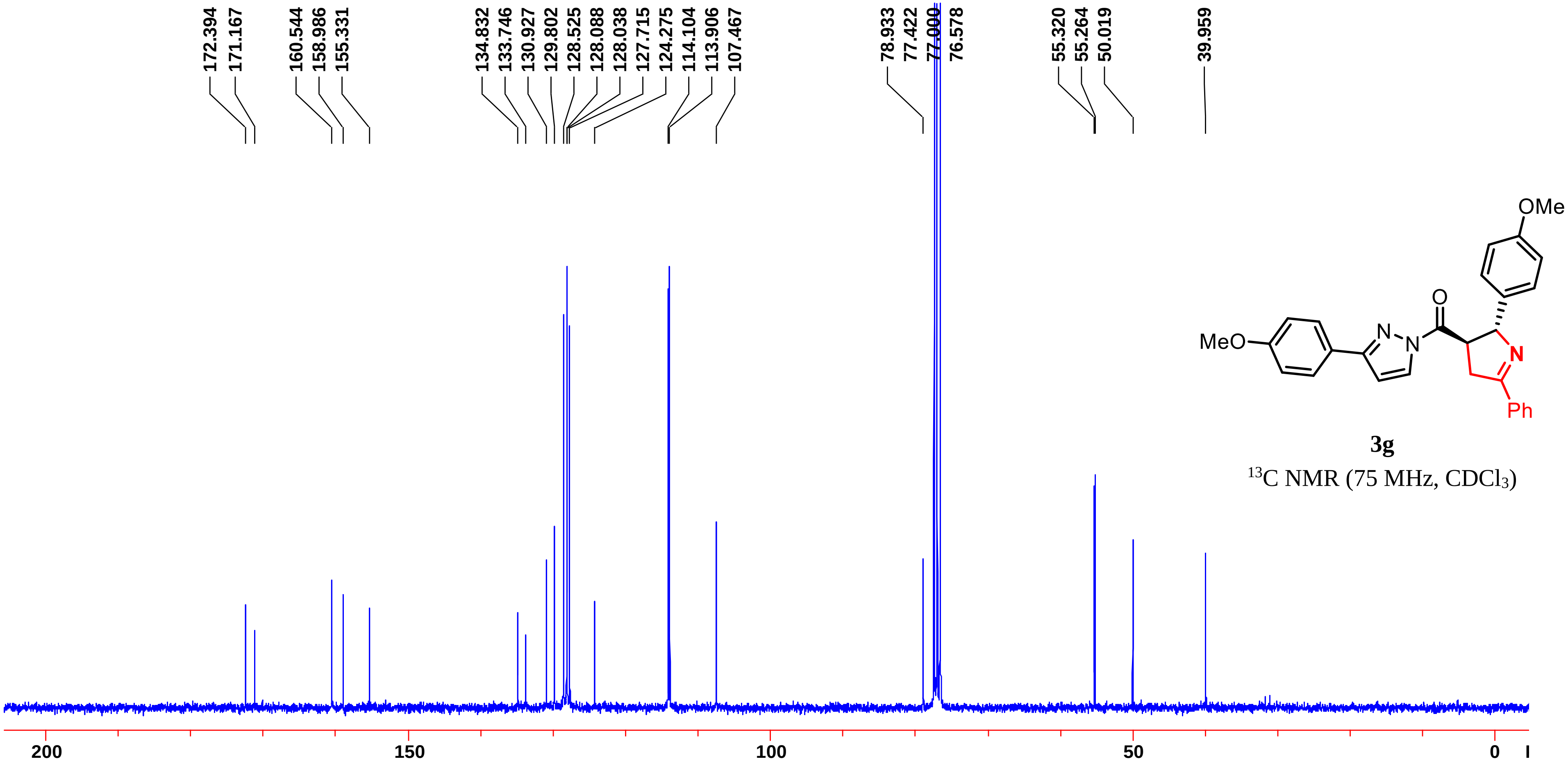

Supplementary Figure 93. ^{13}C NMR spectrum of compound **3g**.

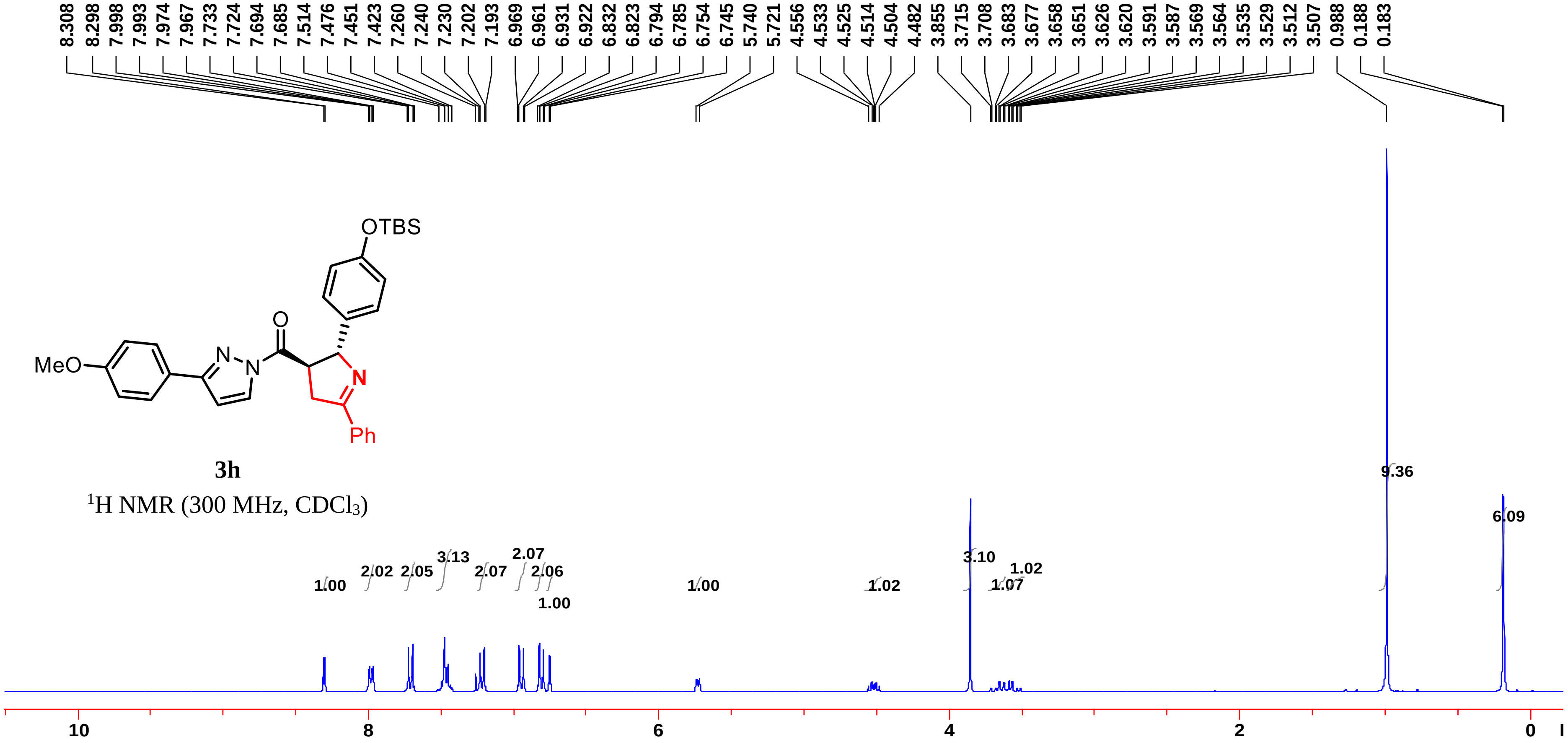

Supplementary Figure 94. ^{1}H NMR spectrum of compound **3h**.

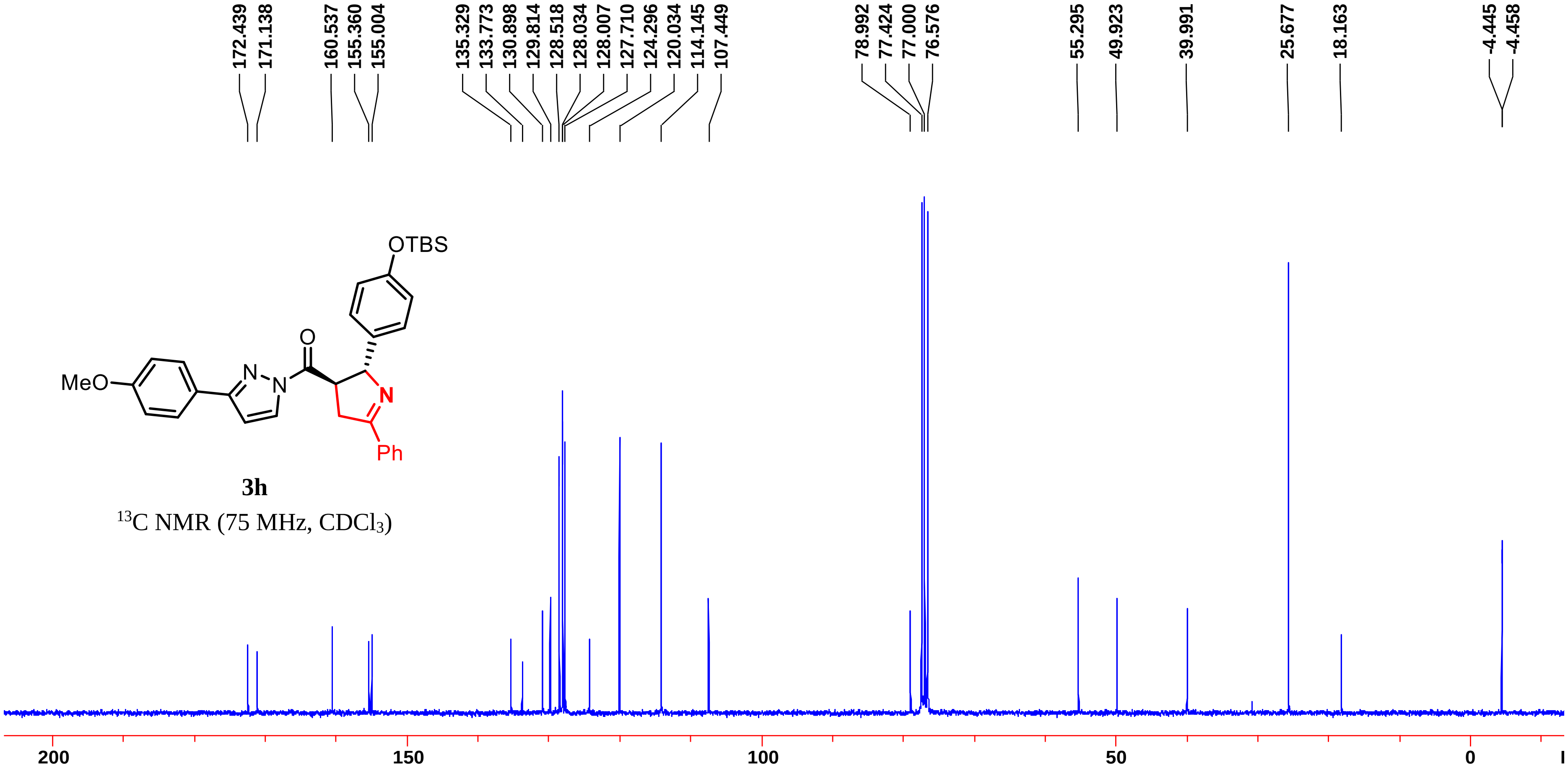

Supplementary Figure 95. ^{13}C NMR spectrum of compound **3h**.

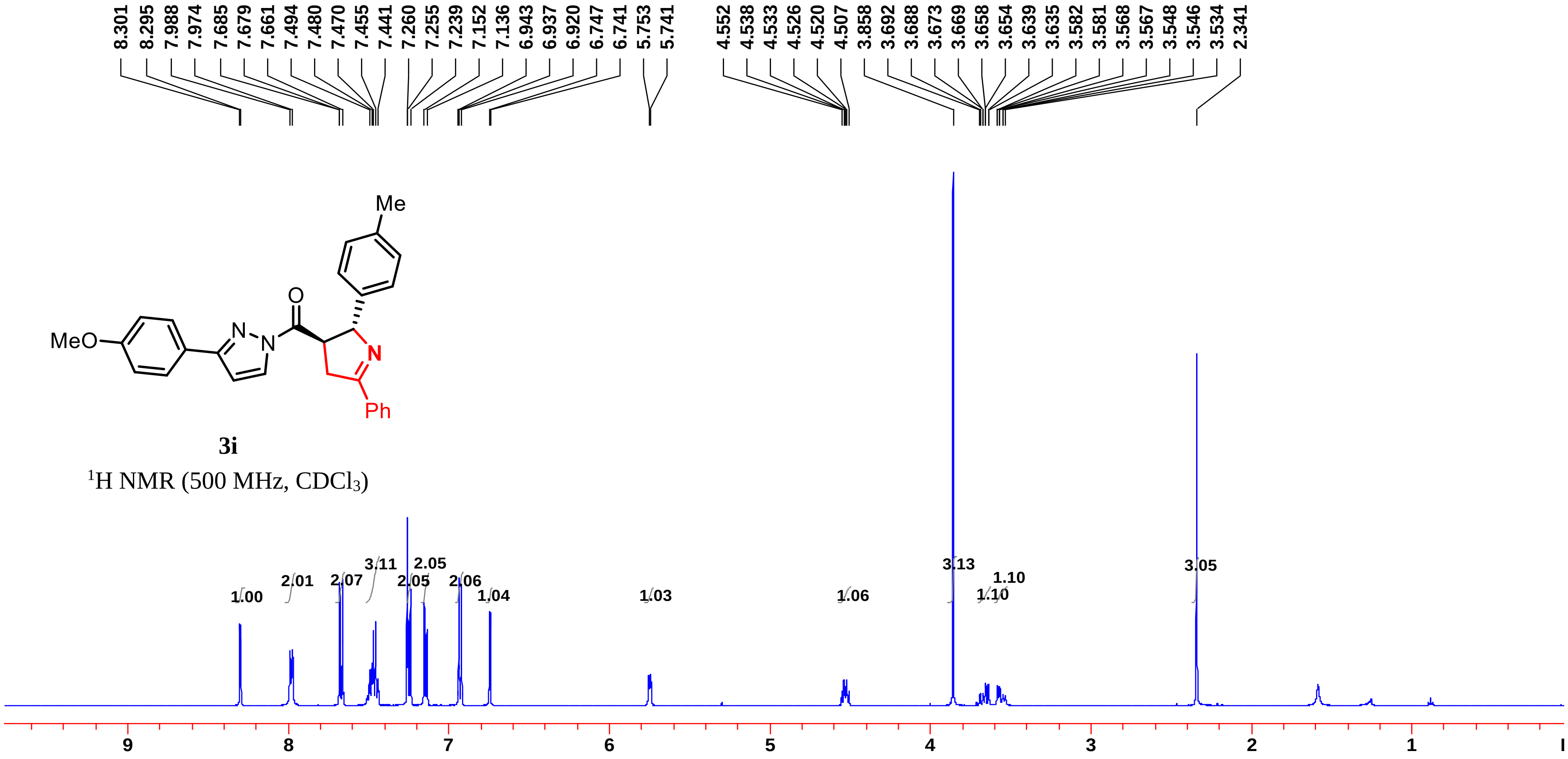

Supplementary Figure 96. ^{1}H NMR spectrum of compound **3i**.

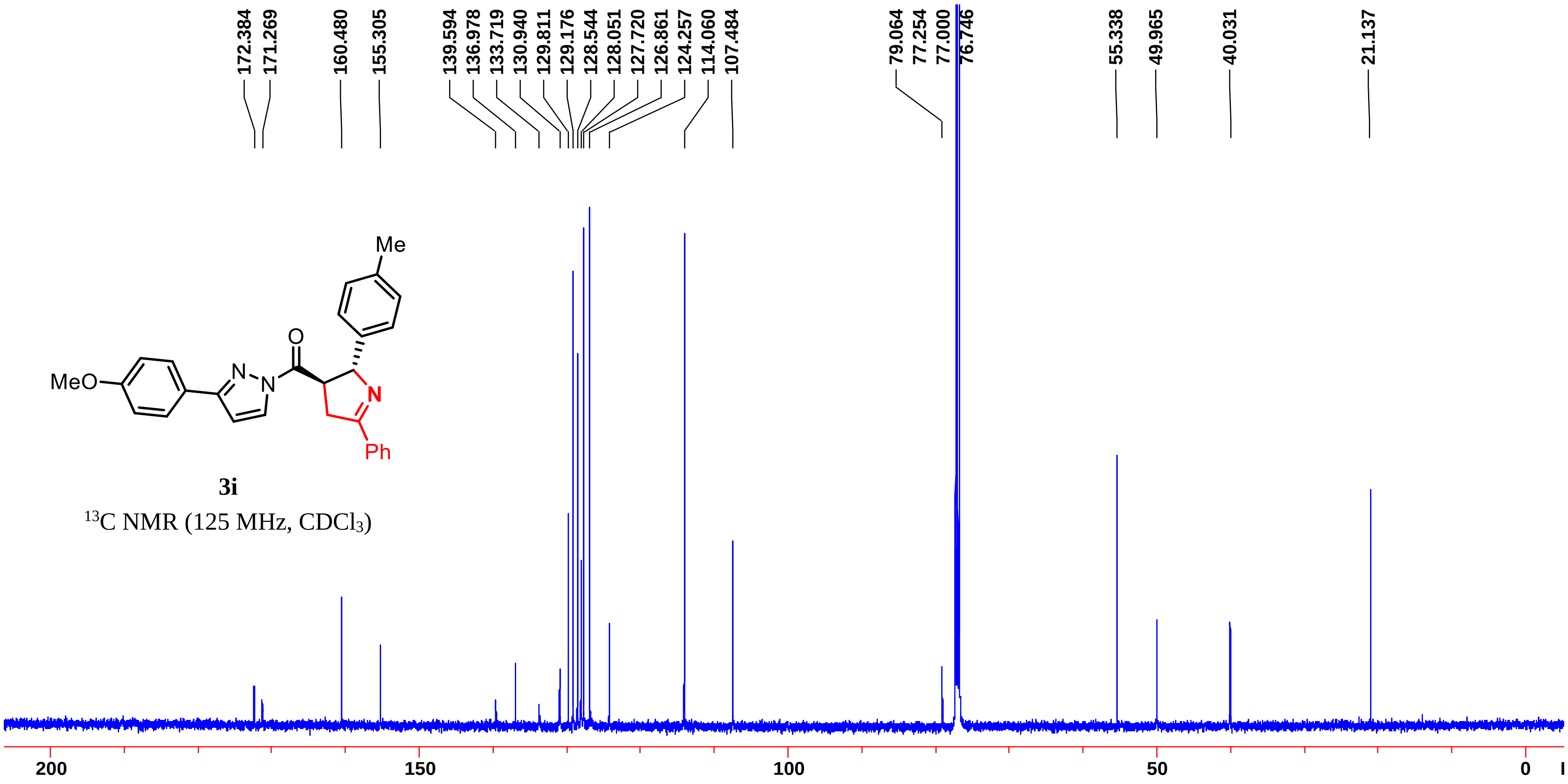

Supplementary Figure 97. ^{13}C NMR spectrum of compound **3i**.

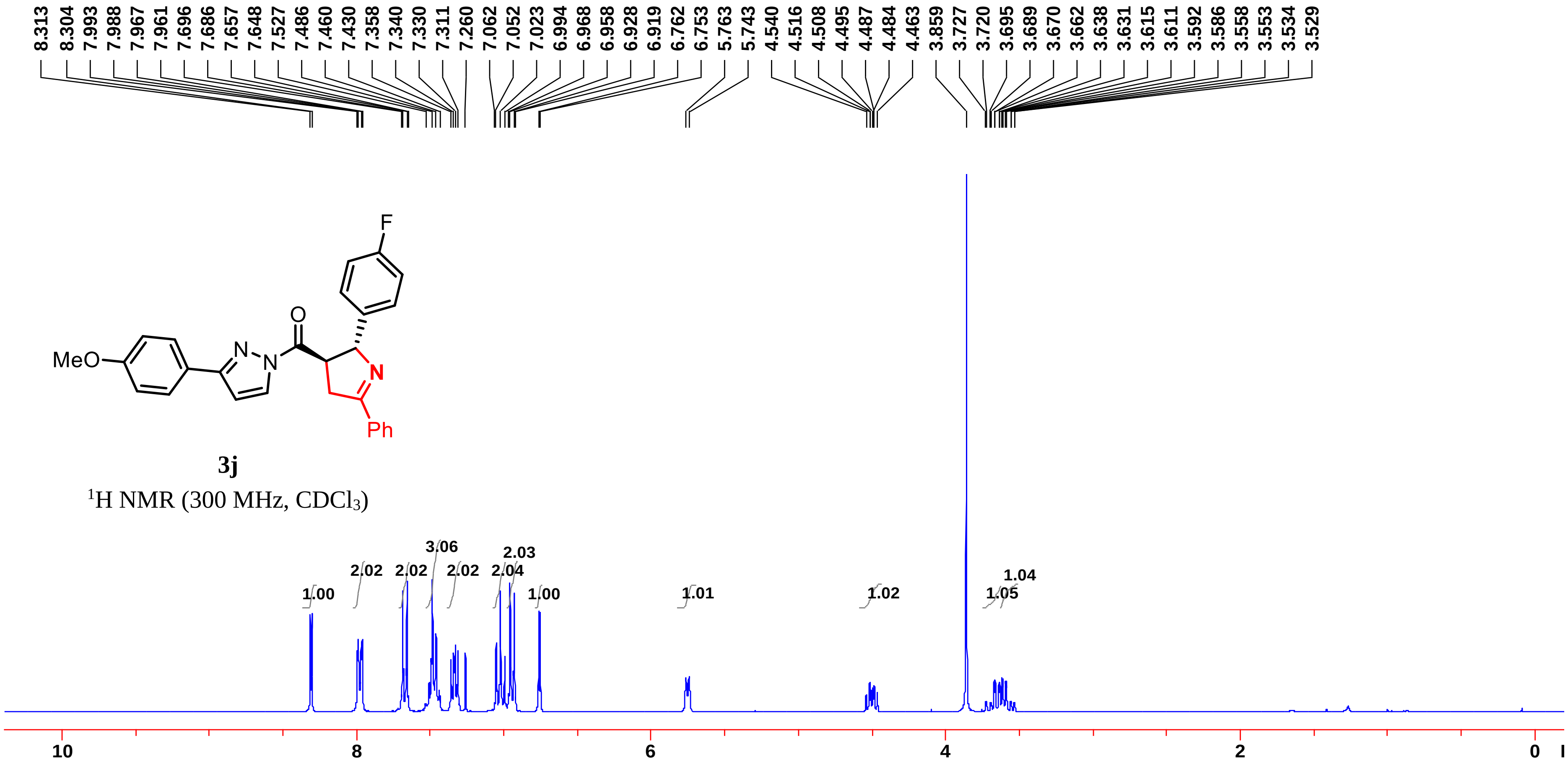

Supplementary Figure 98. ^{1}H NMR spectrum of compound **3j**.

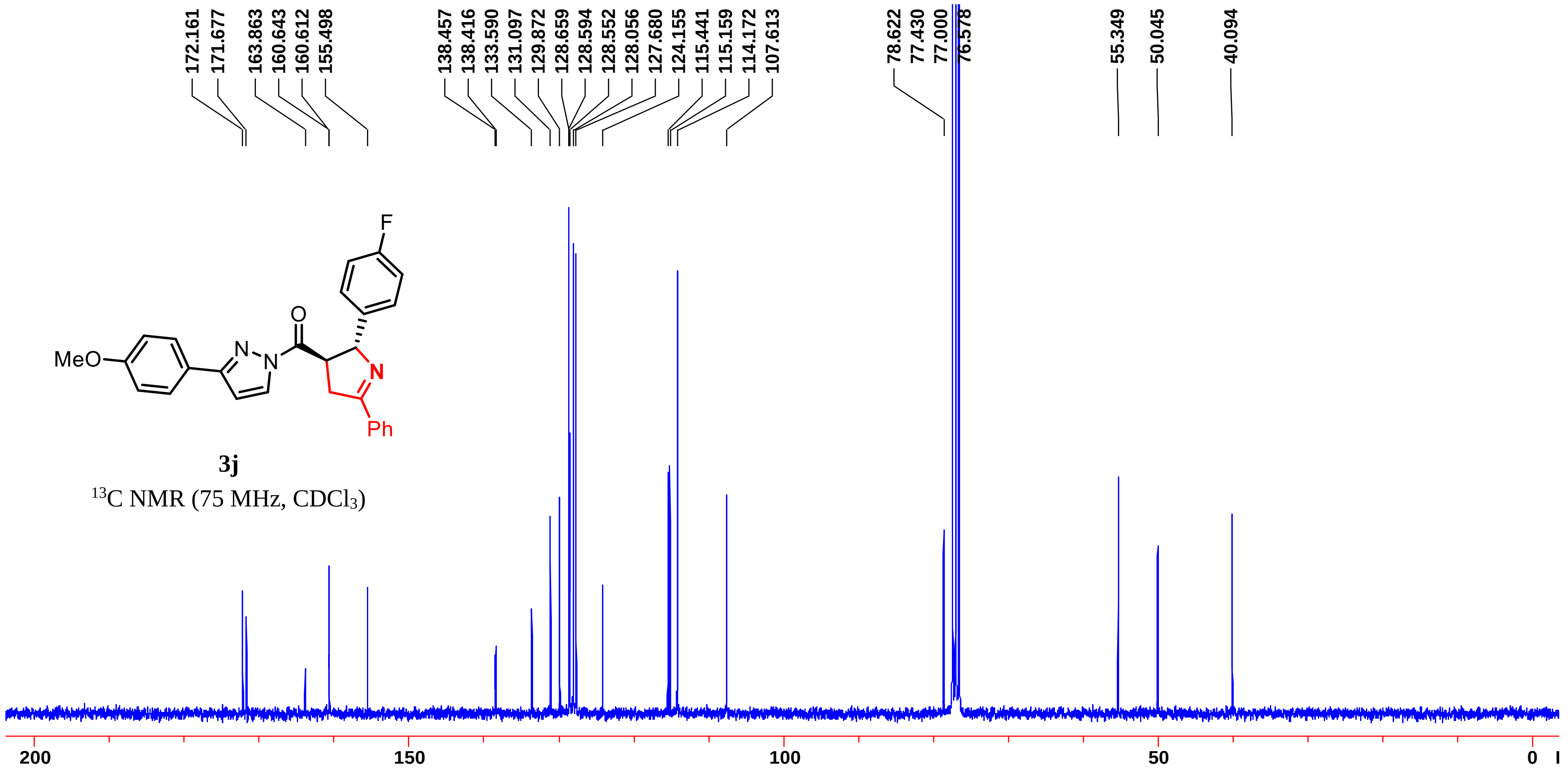

Supplementary Figure 99. ^{13}C NMR spectrum of compound **3j**.

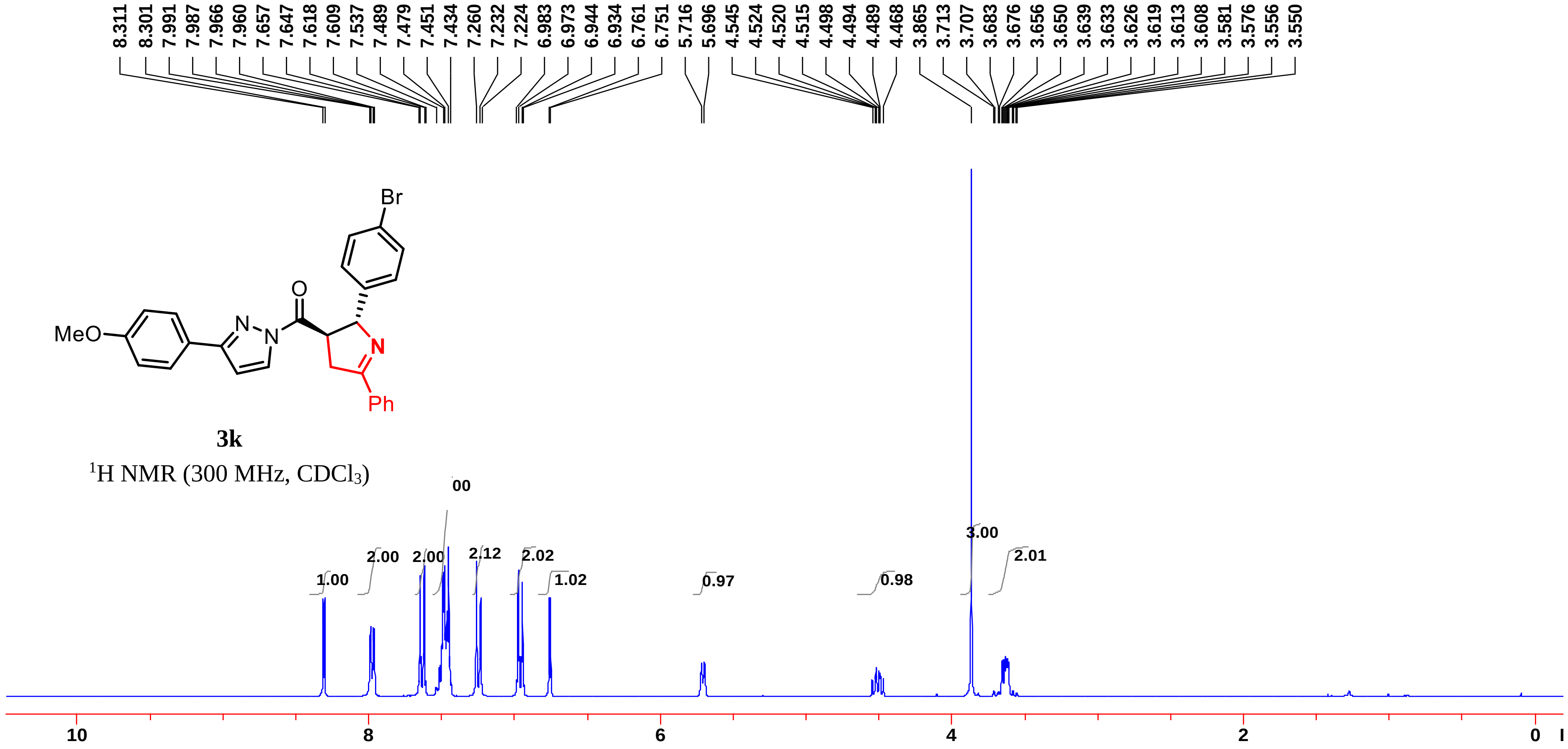

Supplementary Figure 100. ^{1}H NMR spectrum of compound **3k**.

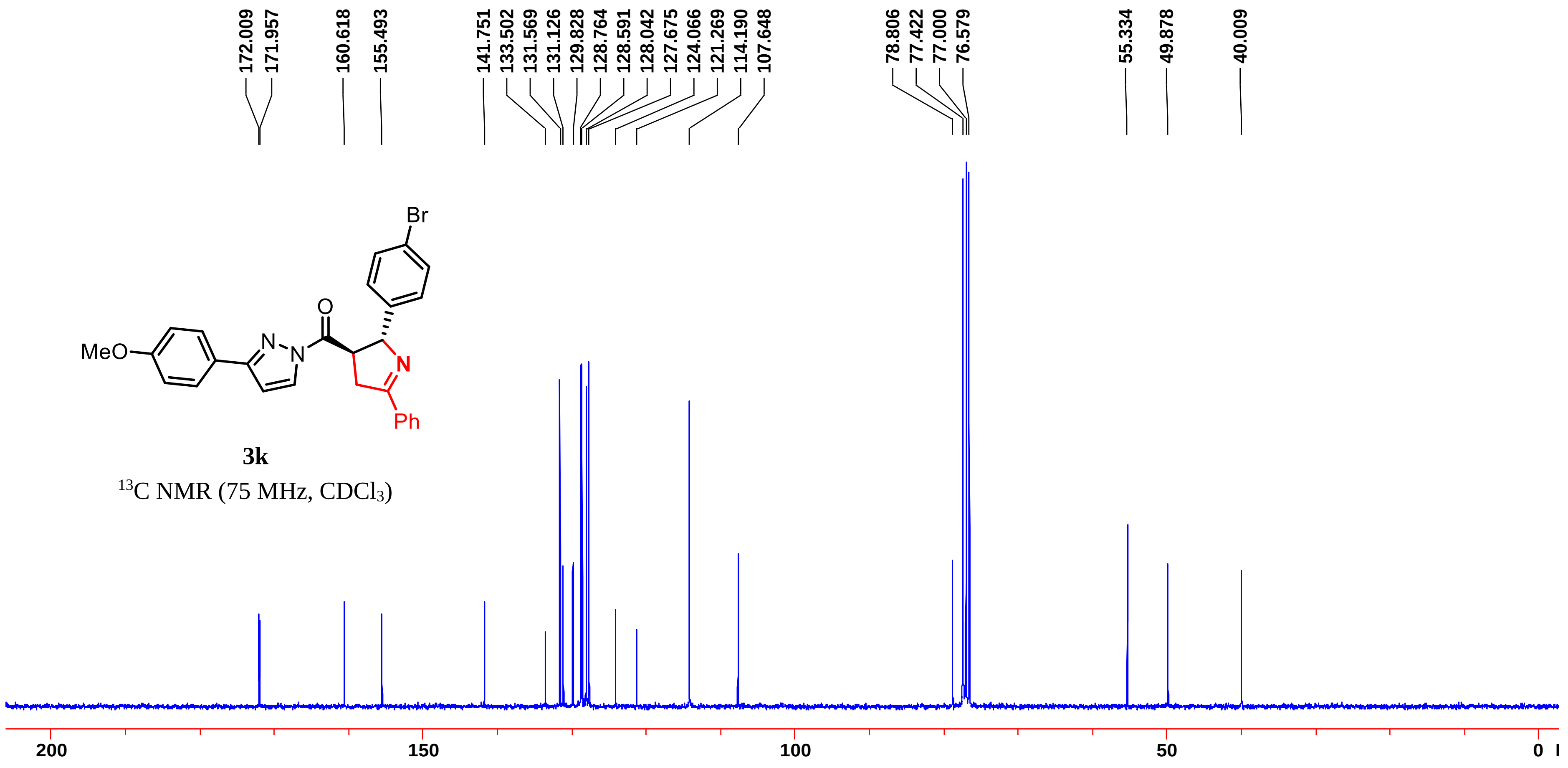

Supplementary Figure 101. ^{13}C NMR spectrum of compound **3k**.

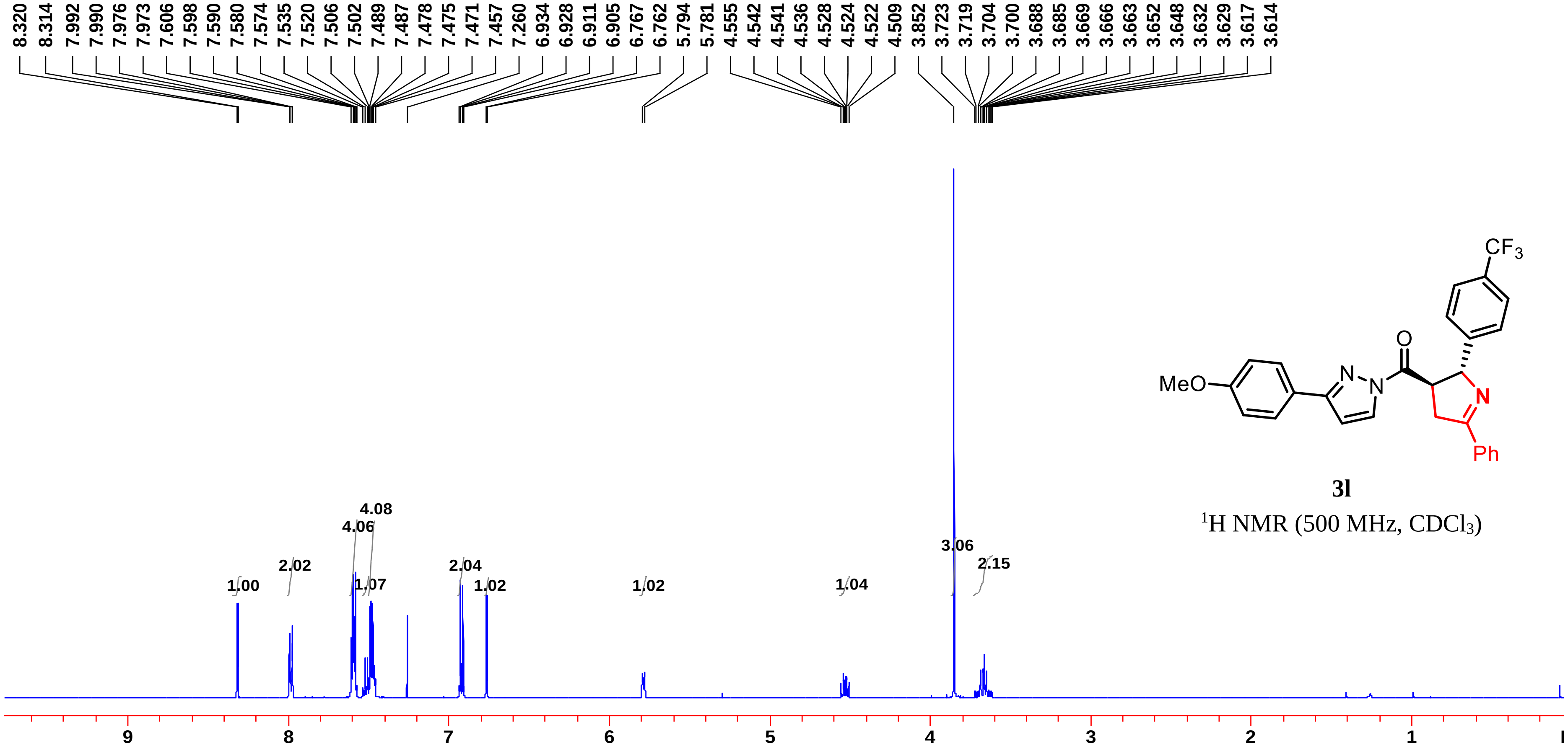

Supplementary Figure 102. ^{1}H NMR spectrum of compound **3l**.

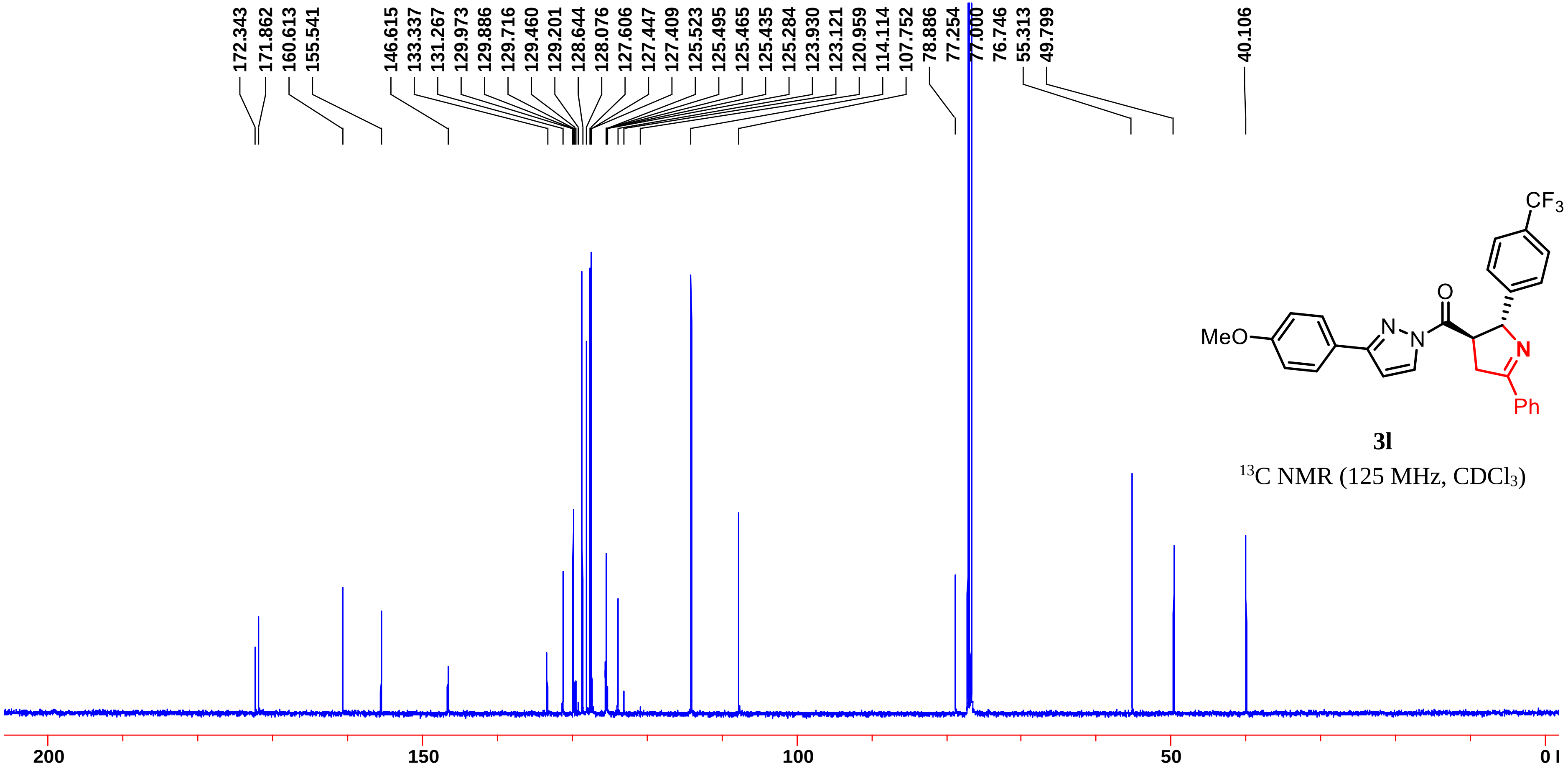

Supplementary Figure 103. ^{13}C NMR spectrum of compound **3l**.

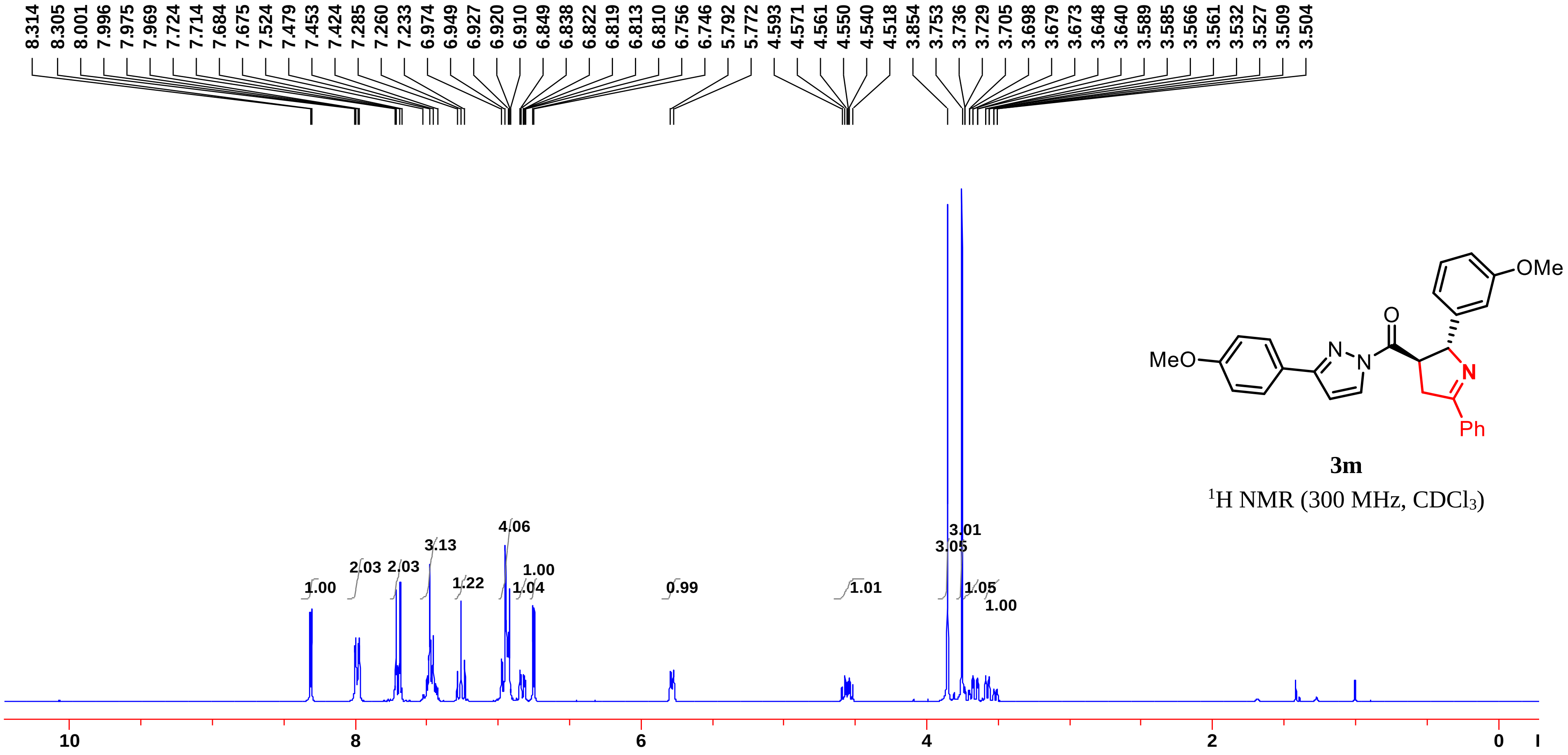

Supplementary Figure 104. ^{1}H NMR spectrum of compound **3m**.

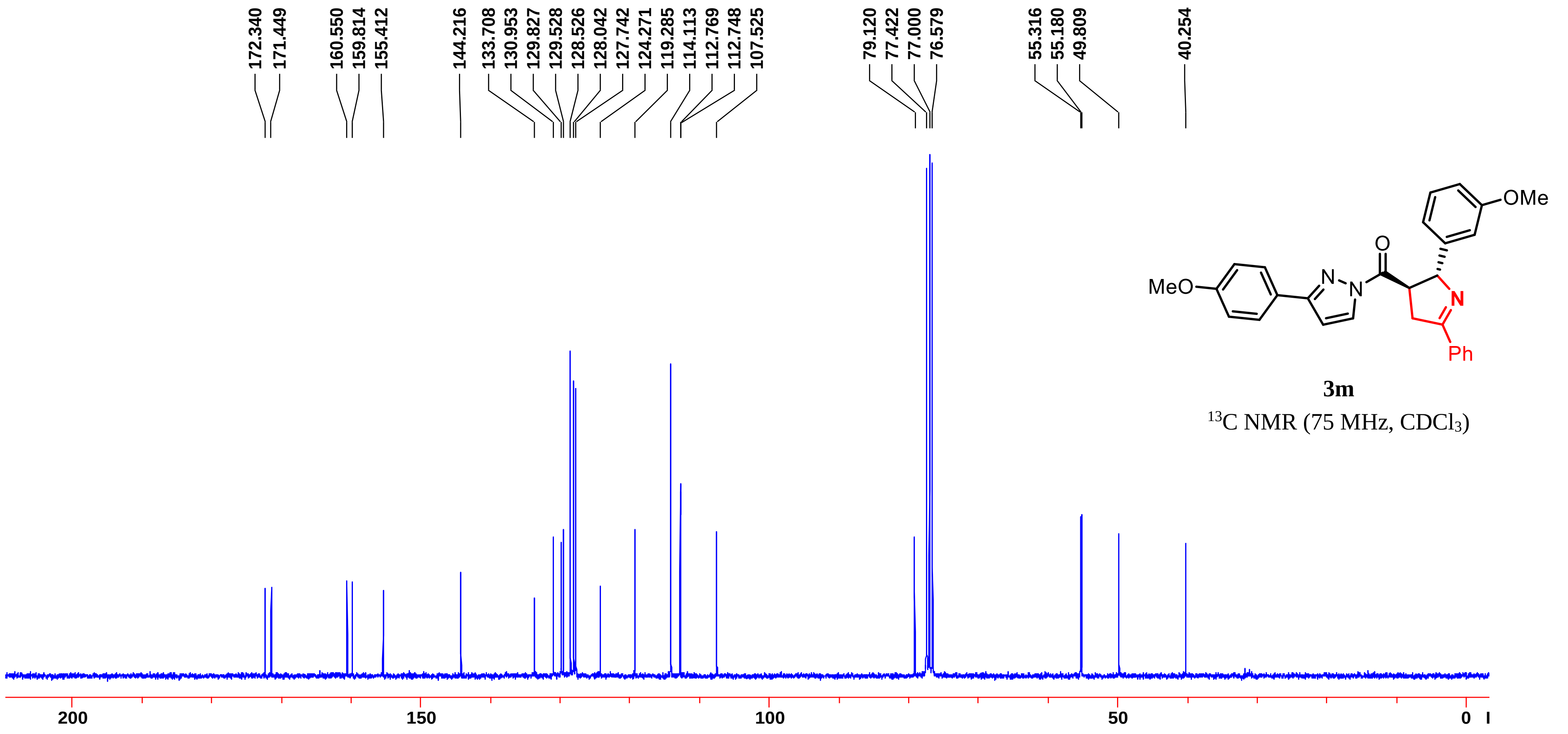

Supplementary Figure 105. ^{13}C NMR spectrum of compound **3m**.

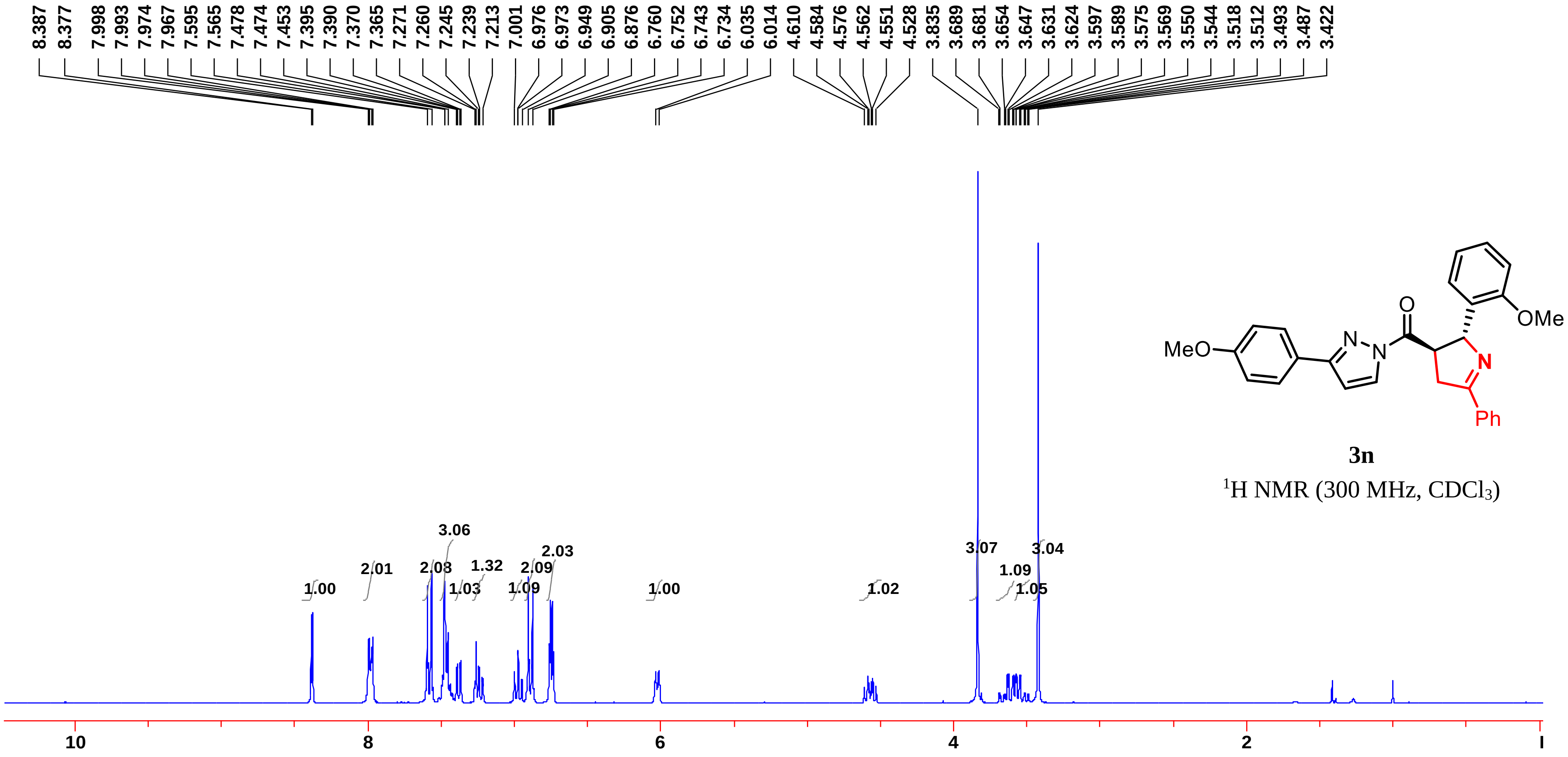

Supplementary Figure 106. ^{1}H NMR spectrum of compound **3n**.

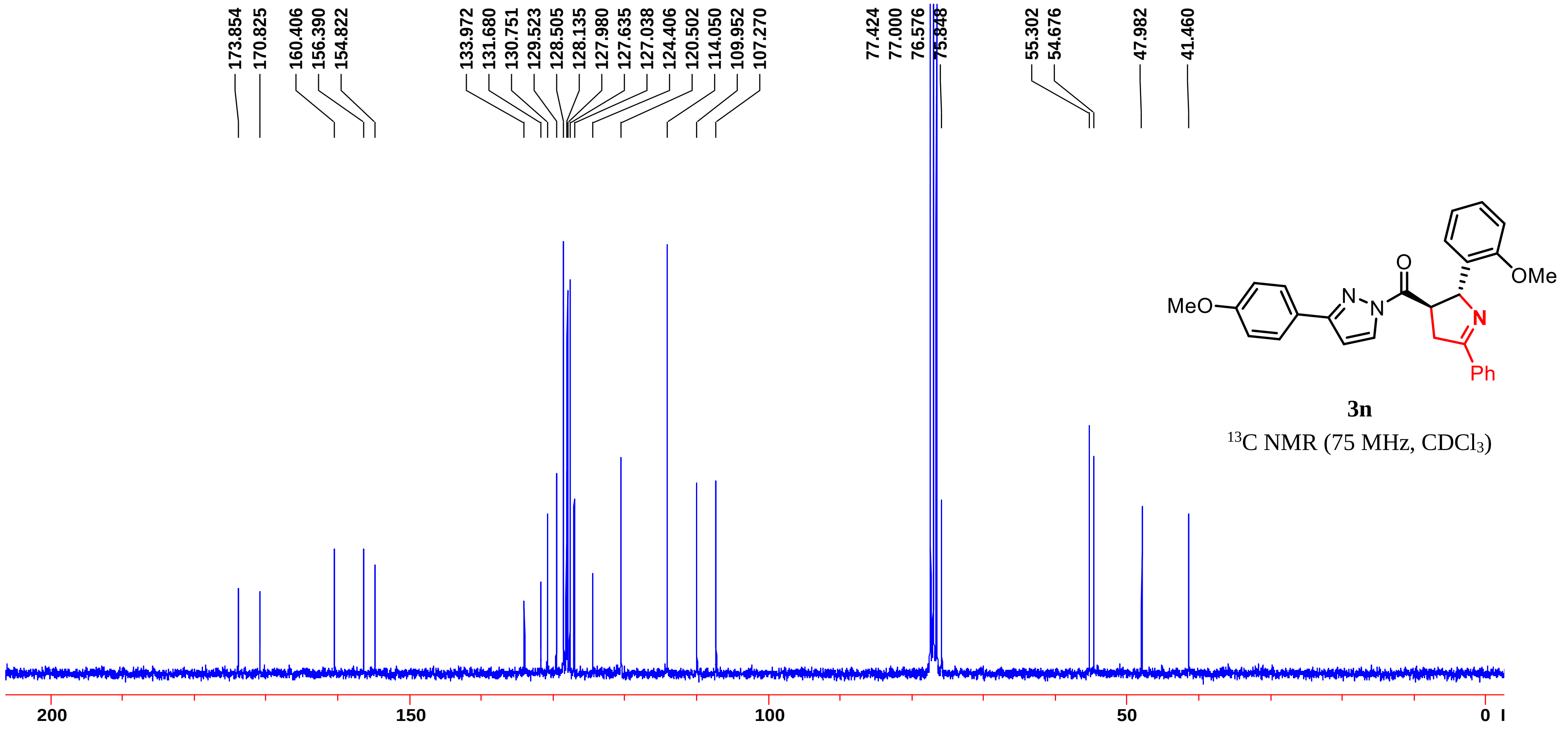

3n

^{13}C NMR (75 MHz, $CDCl_3$)

Supplementary Figure 107. ^{13}C NMR spectrum of compound **3n**.

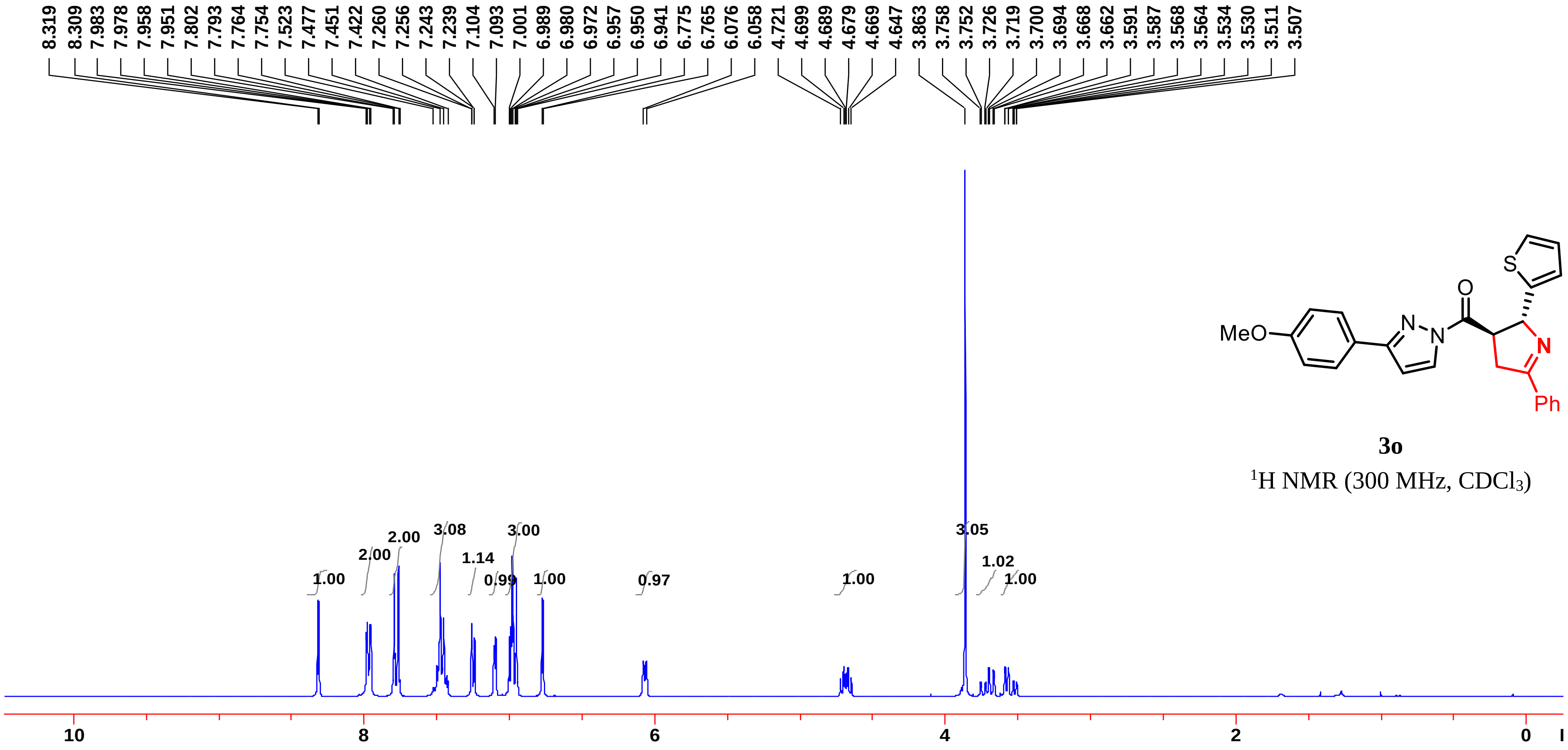

Supplementary Figure 108. ^{1}H NMR spectrum of compound **3o**.

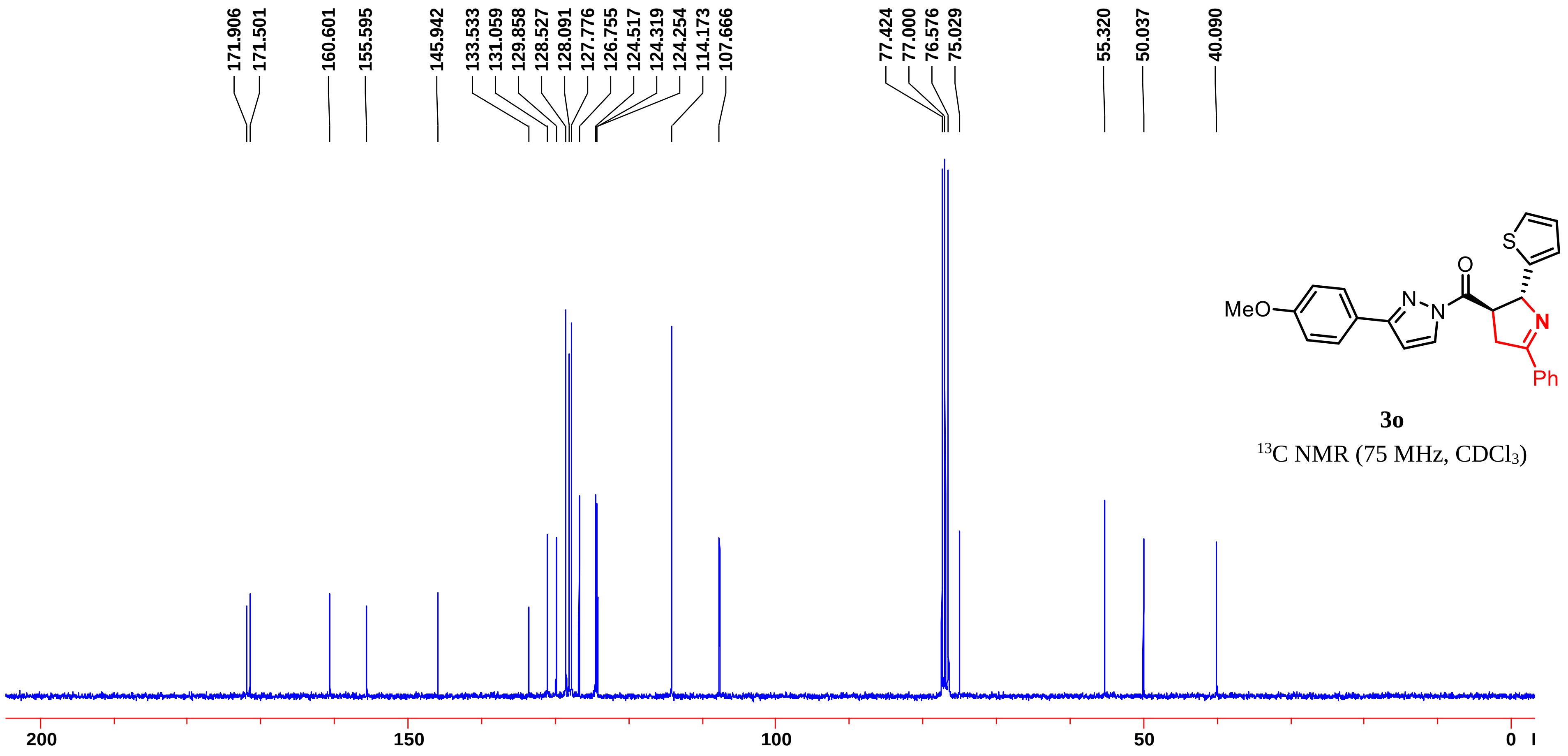

Supplementary Figure 109. ^{13}C NMR spectrum of compound **3o**.

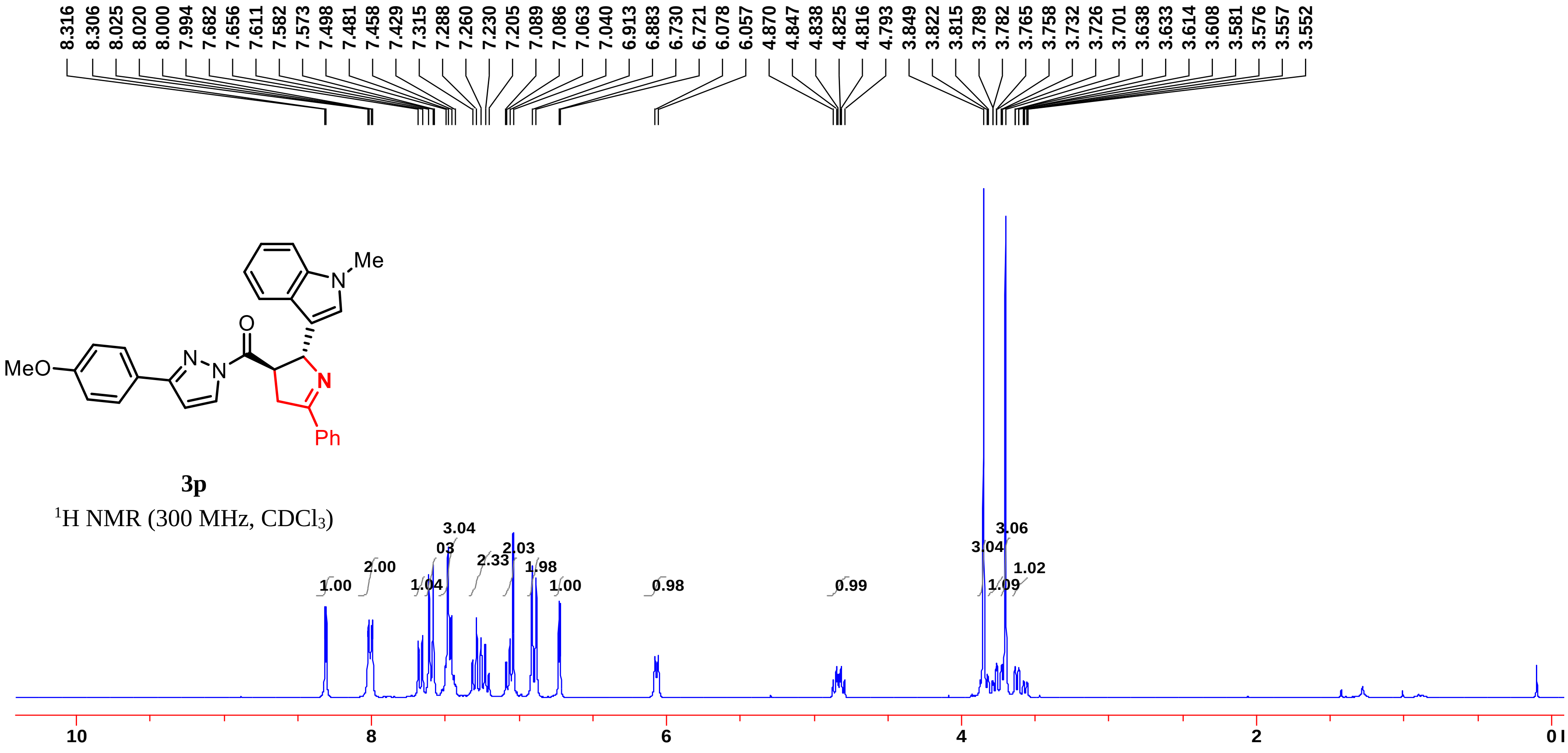

Supplementary Figure 110. ^{1}H NMR spectrum of compound **3p**.

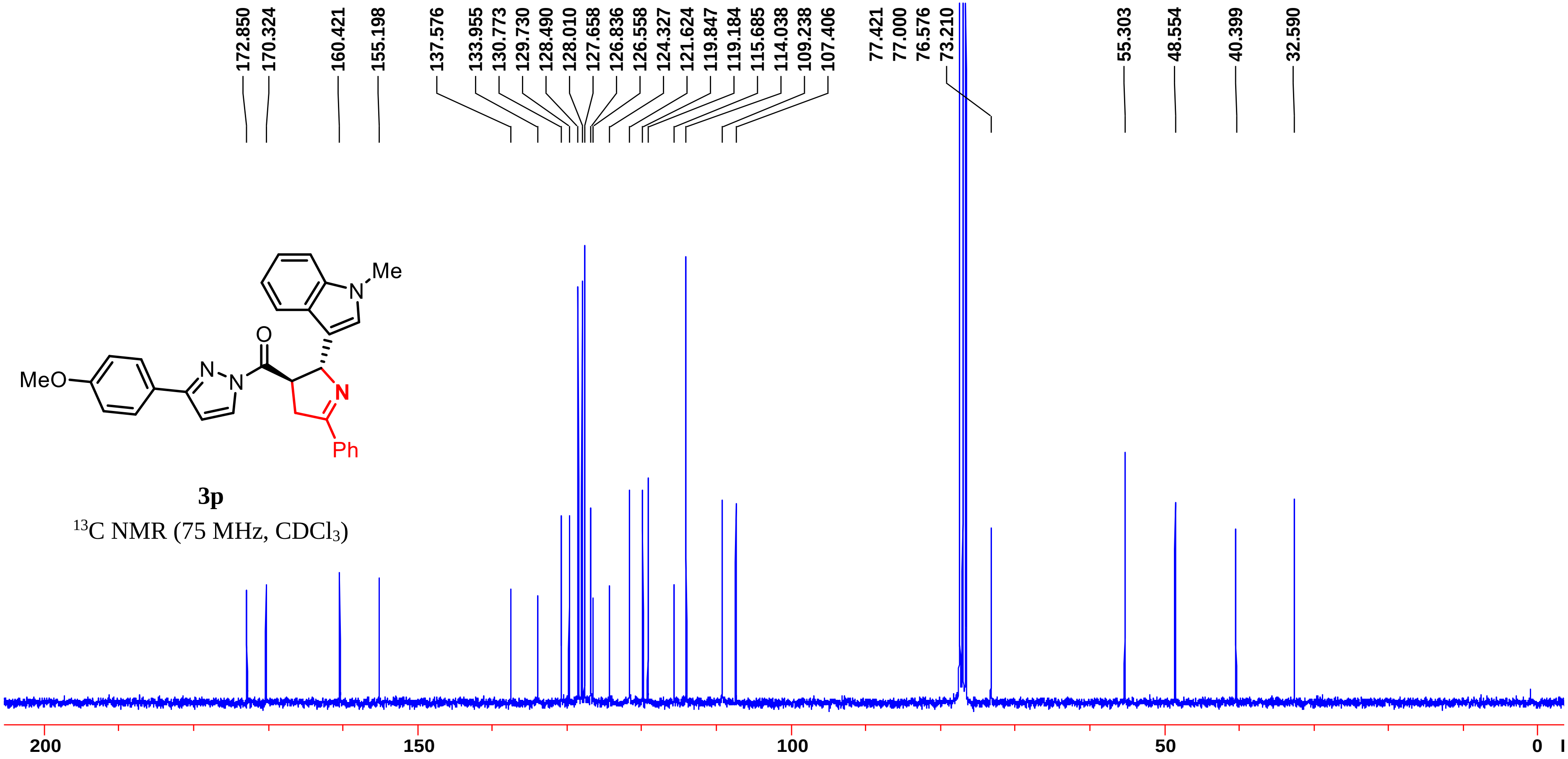

Supplementary Figure 111. ^{13}C NMR spectrum of compound **3p**.

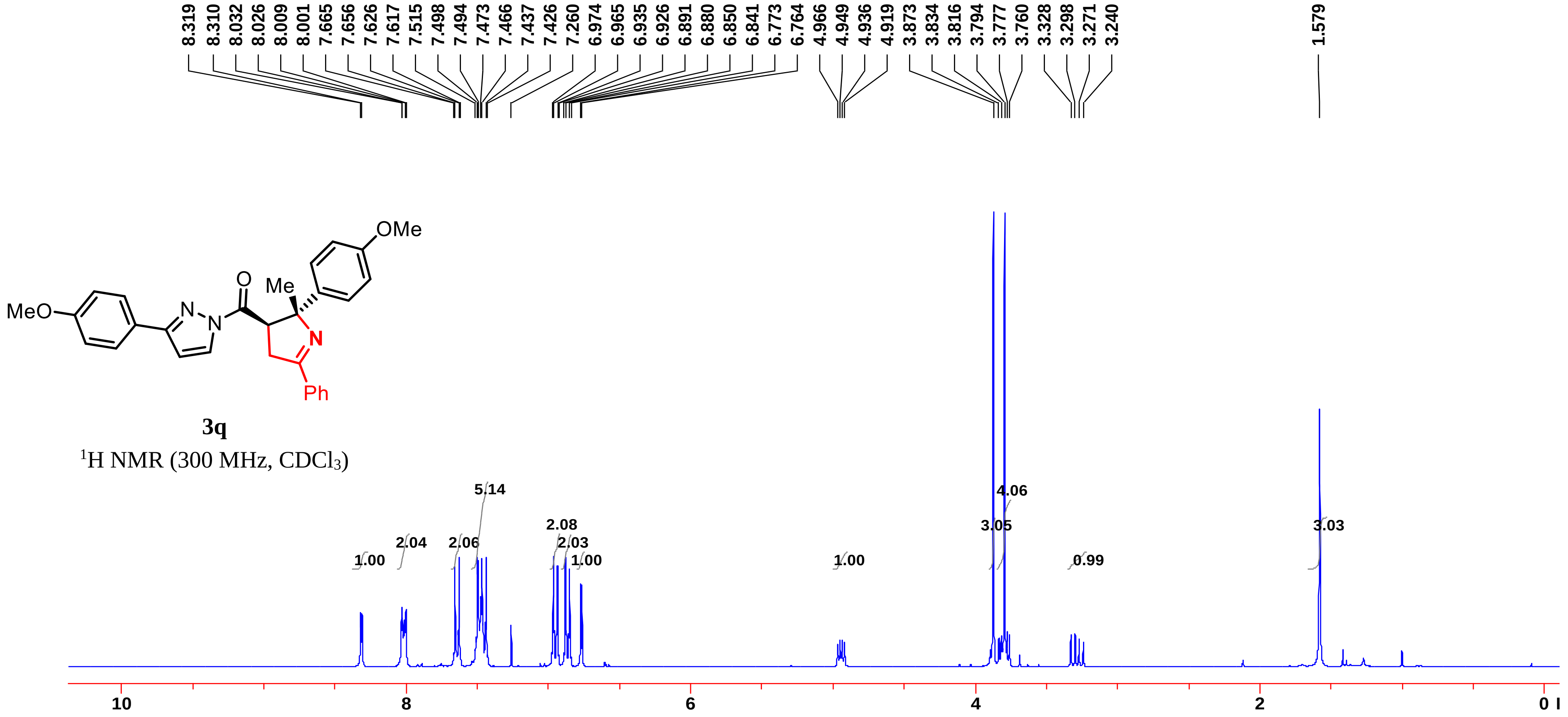

Supplementary Figure 112. ^{1}H NMR spectrum of compound **3q**.

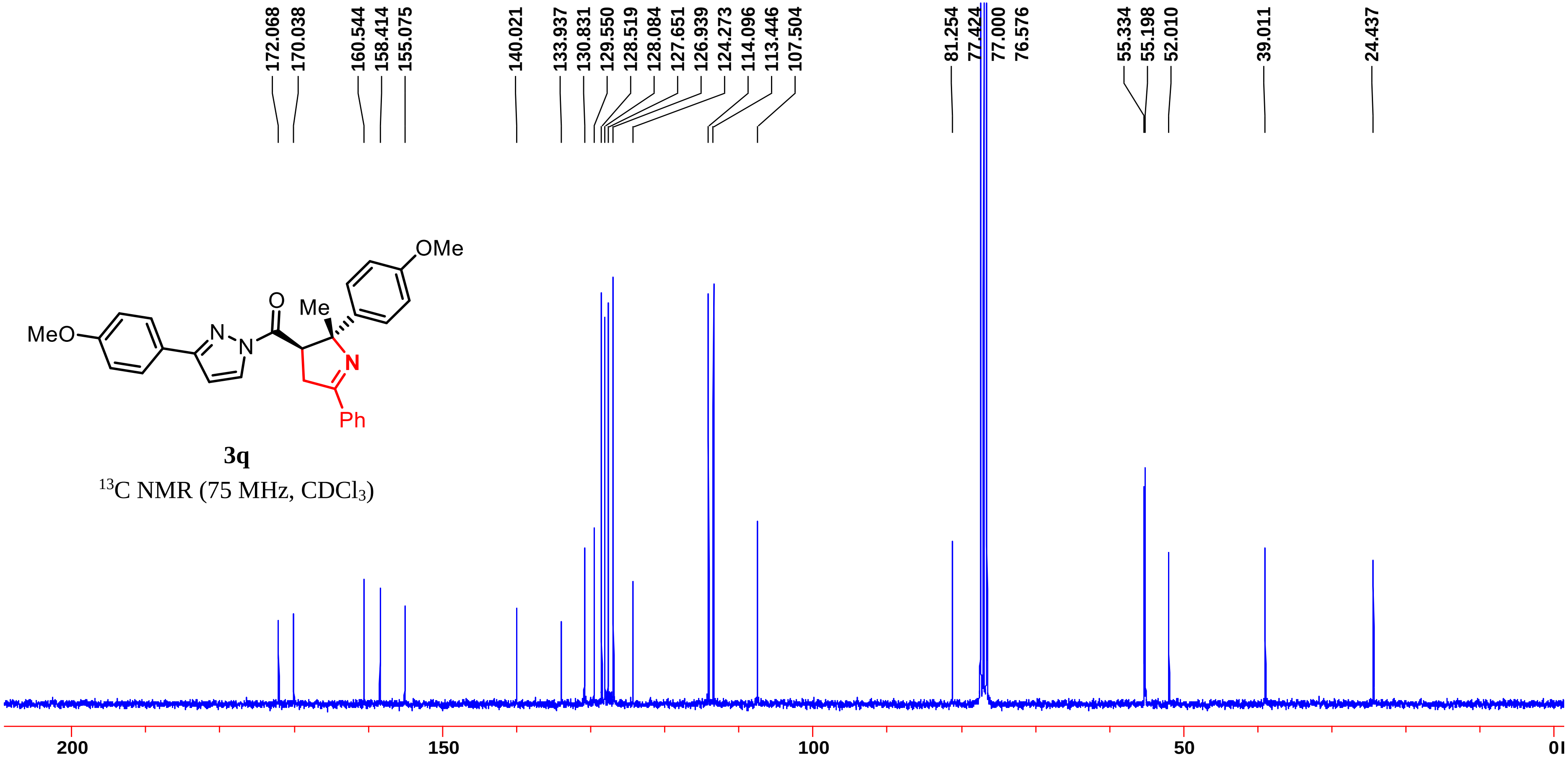

Supplementary Figure 113. ^{13}C NMR spectrum of compound **3q**.

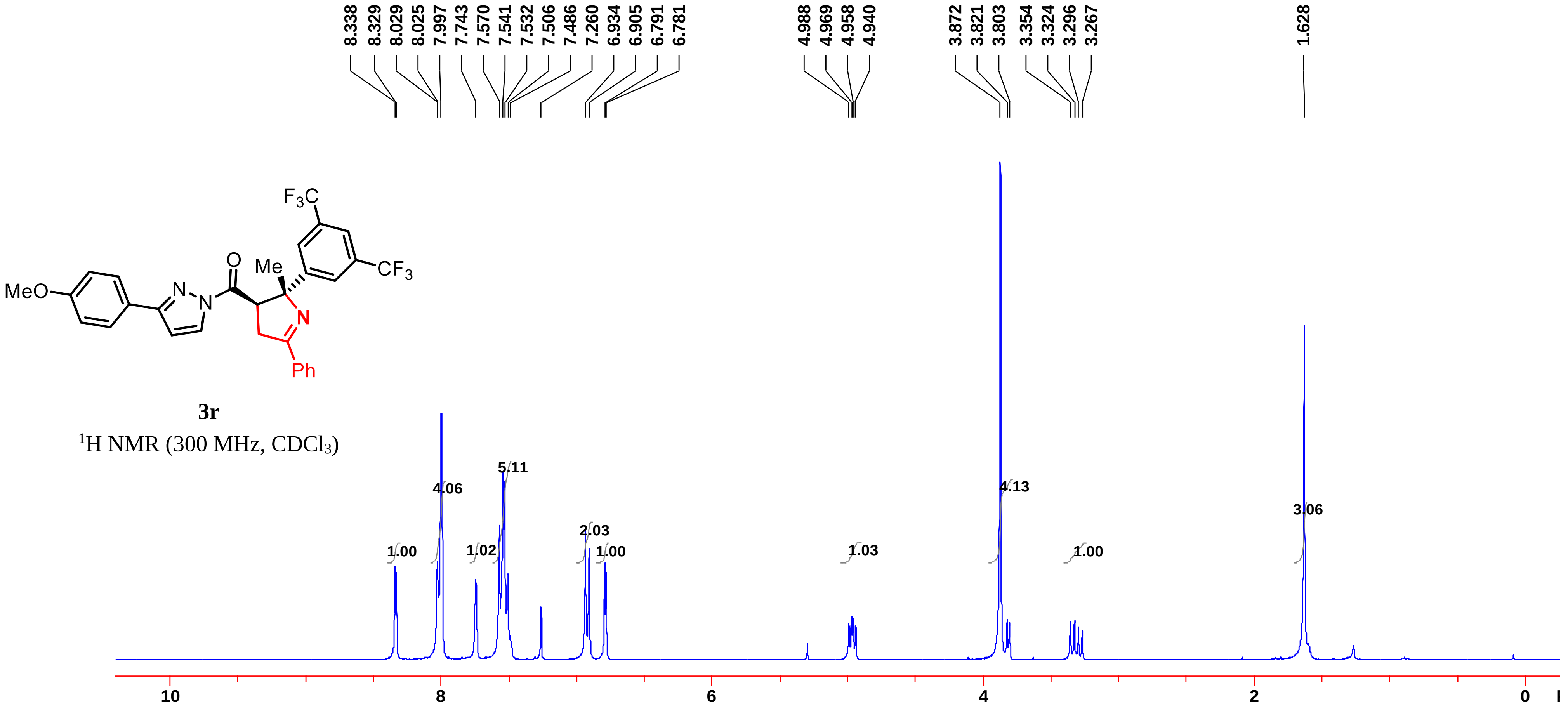

Supplementary Figure 114. ^{1}H NMR spectrum of compound **3r**.

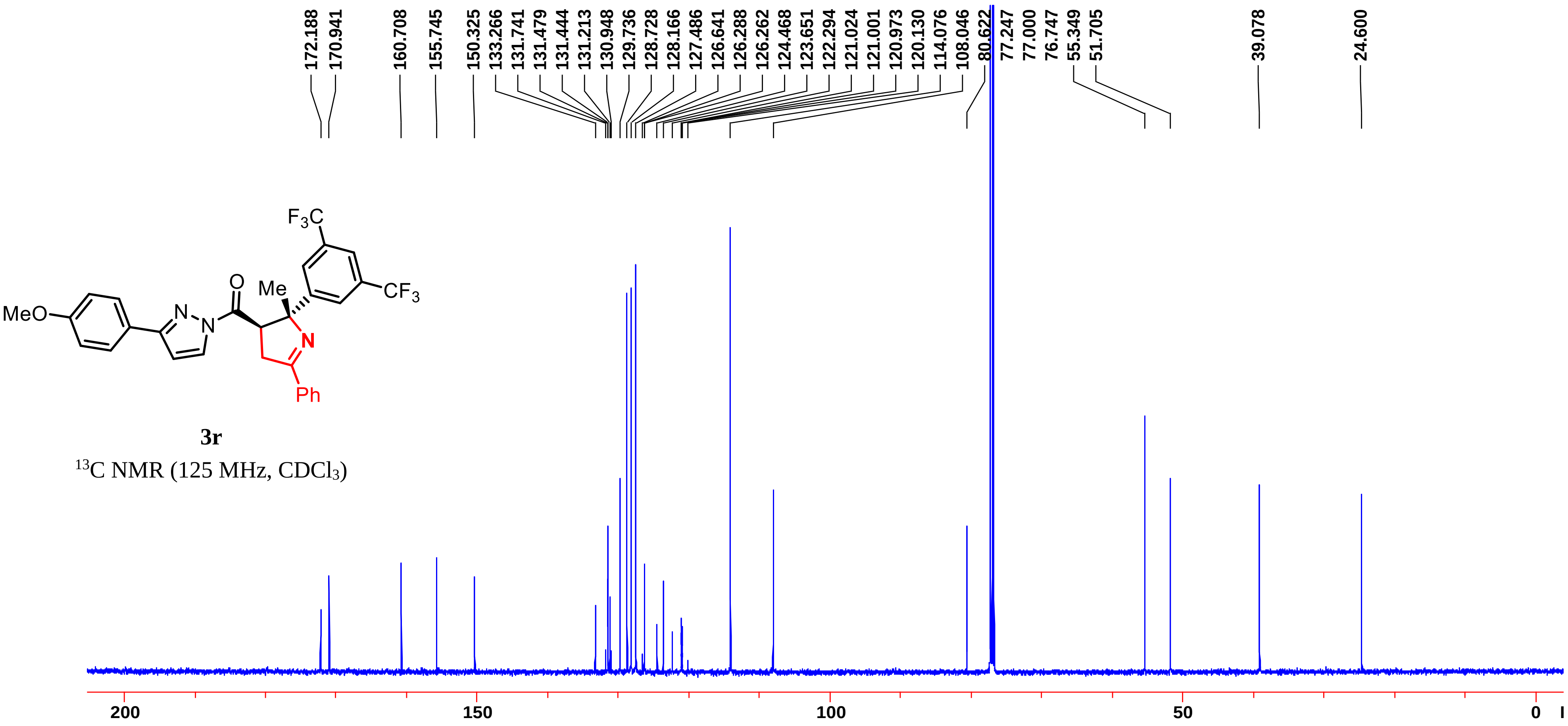

Supplementary Figure 115. ^{13}C NMR spectrum of compound **3r**.

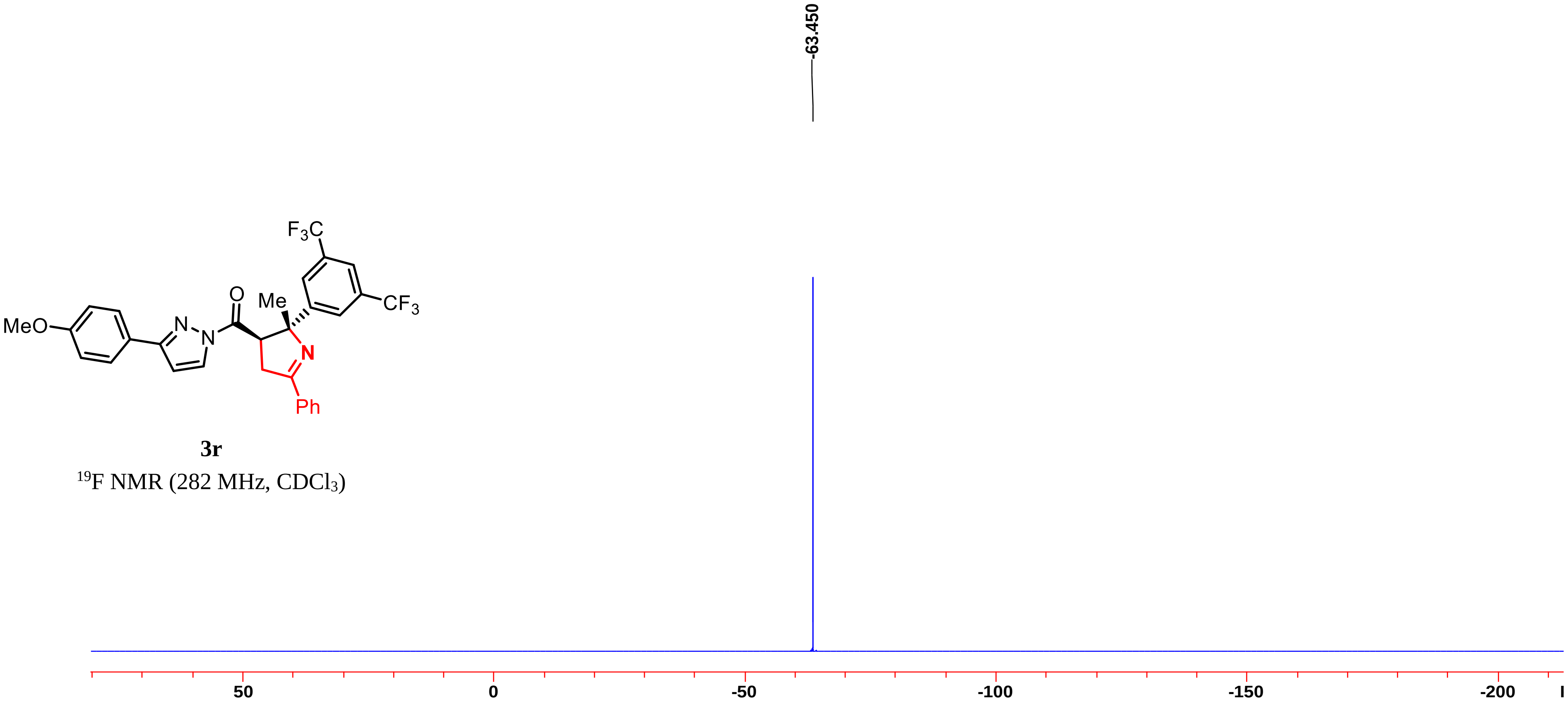

Supplementary Figure 116. ^{19}F NMR spectrum of compound **3r**.

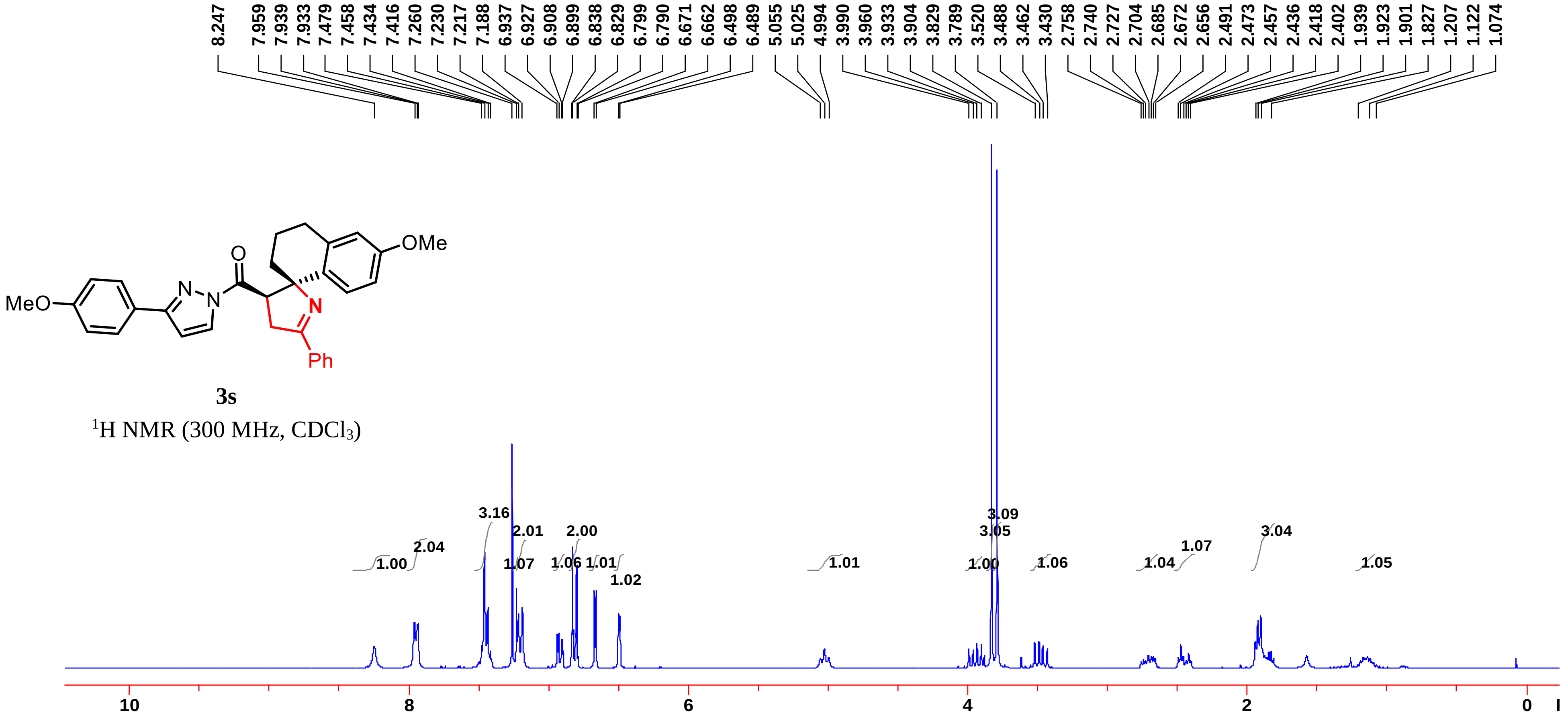

Supplementary Figure 117. ^{1}H NMR spectrum of compound **3s**.

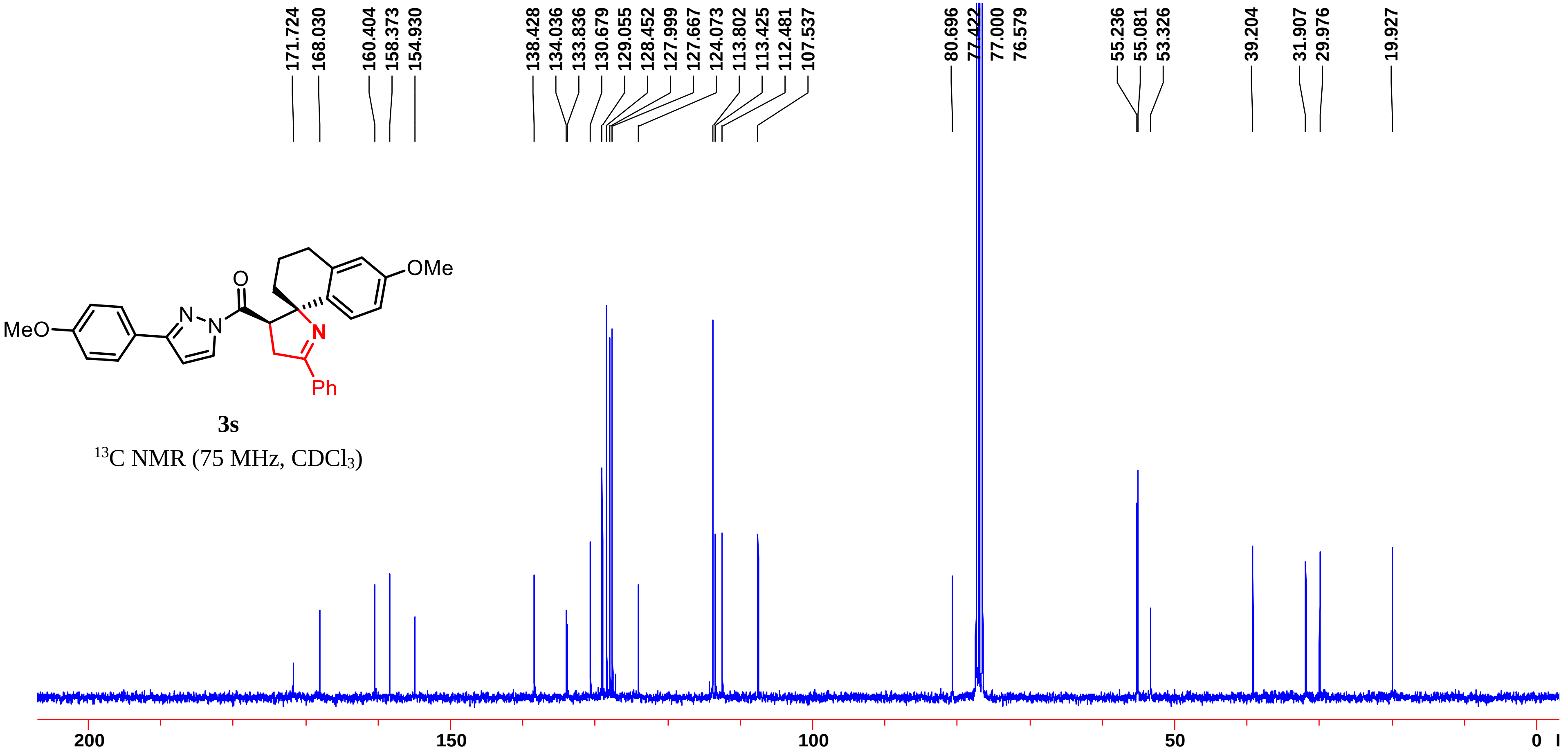

3s

^{13}C NMR (75 MHz, $CDCl_3$)

Supplementary Figure 118. ^{13}C NMR spectrum of compound **3s**.

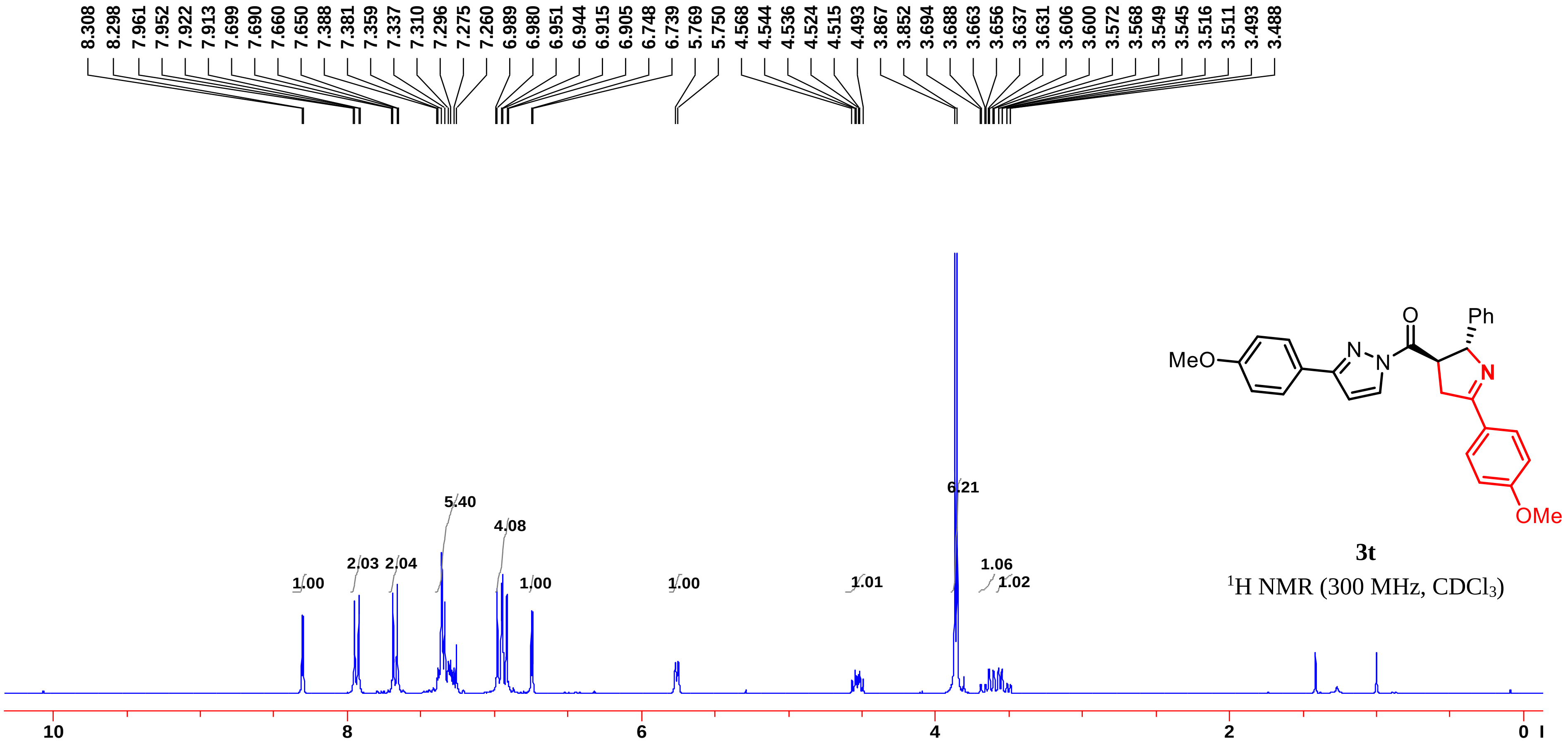

Supplementary Figure 119. ^{1}H NMR spectrum of compound **3t**.

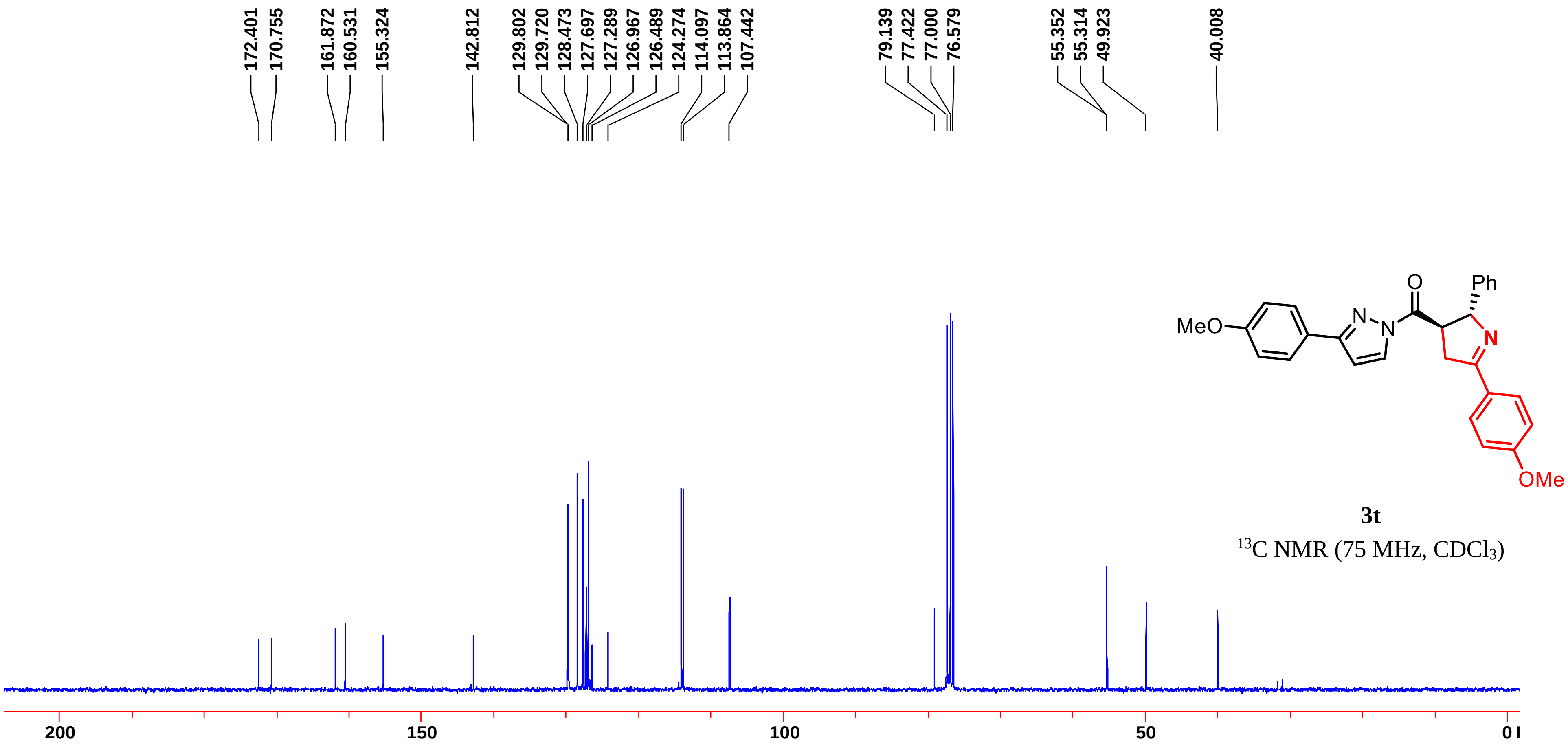

Supplementary Figure 120. ^{13}C NMR spectrum of compound **3t**.

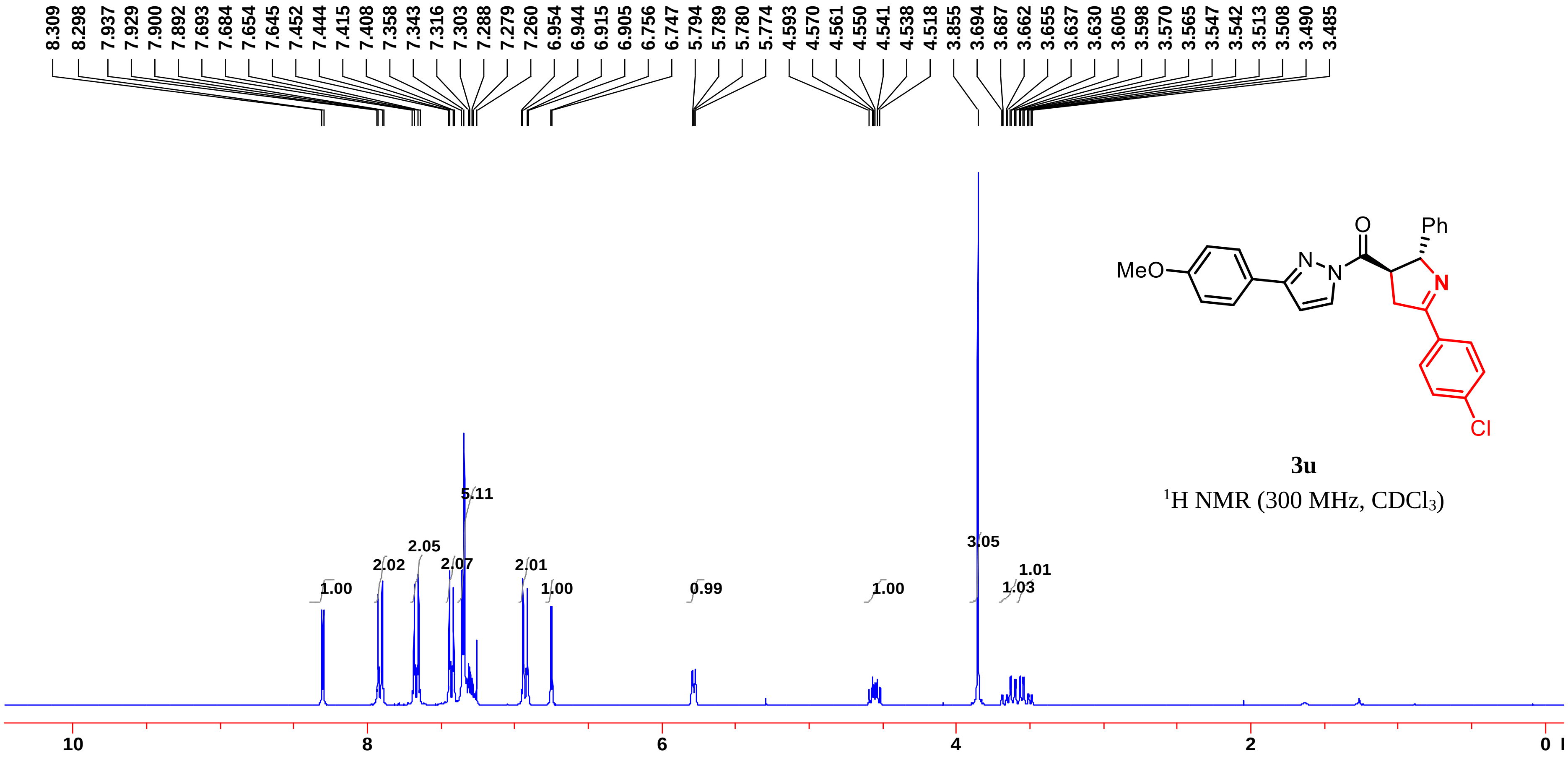

Supplementary Figure 121. ^{1}H NMR spectrum of compound **3u**.

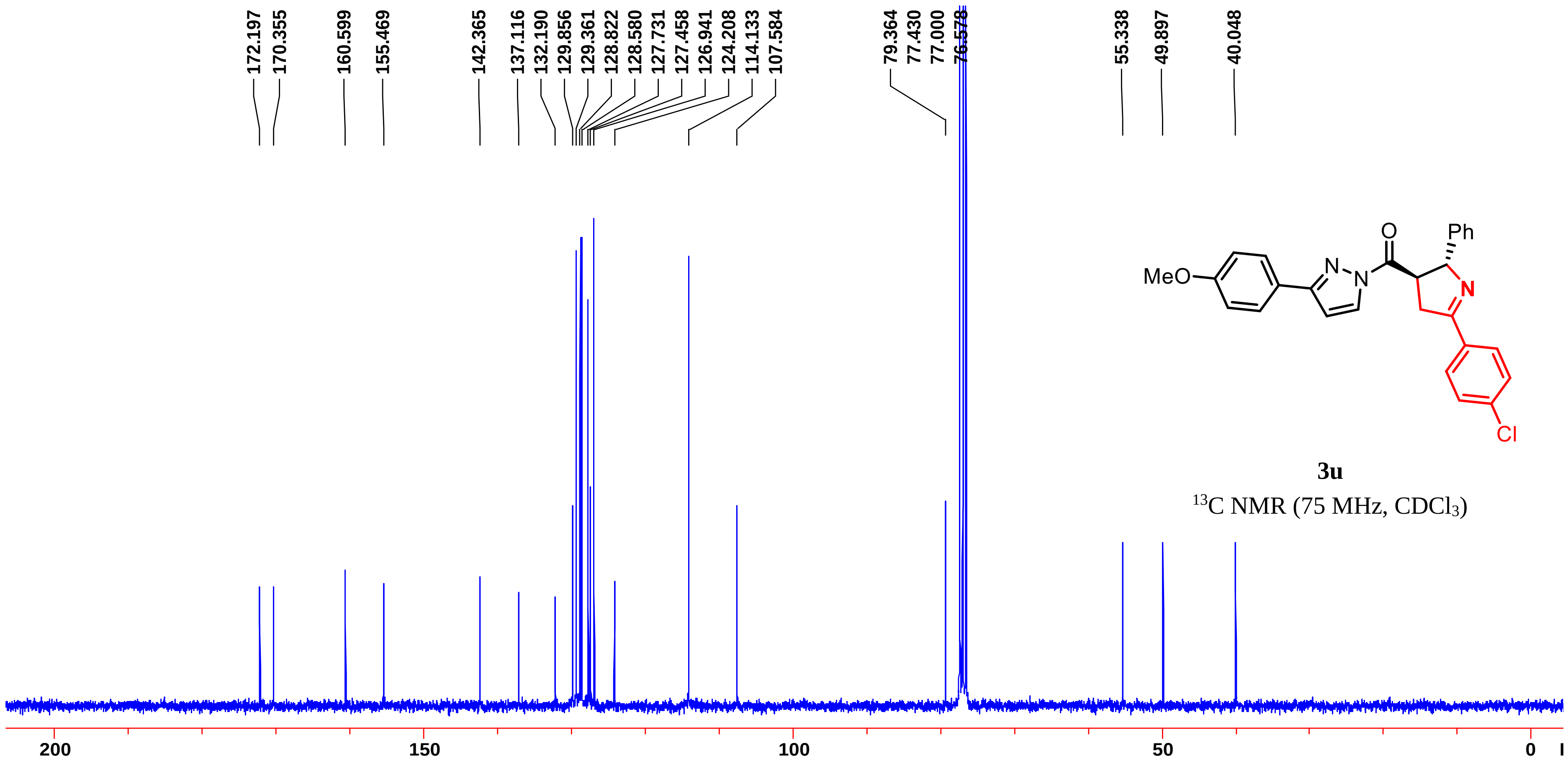

Supplementary Figure 122. ^{13}C NMR spectrum of compound **3u**.

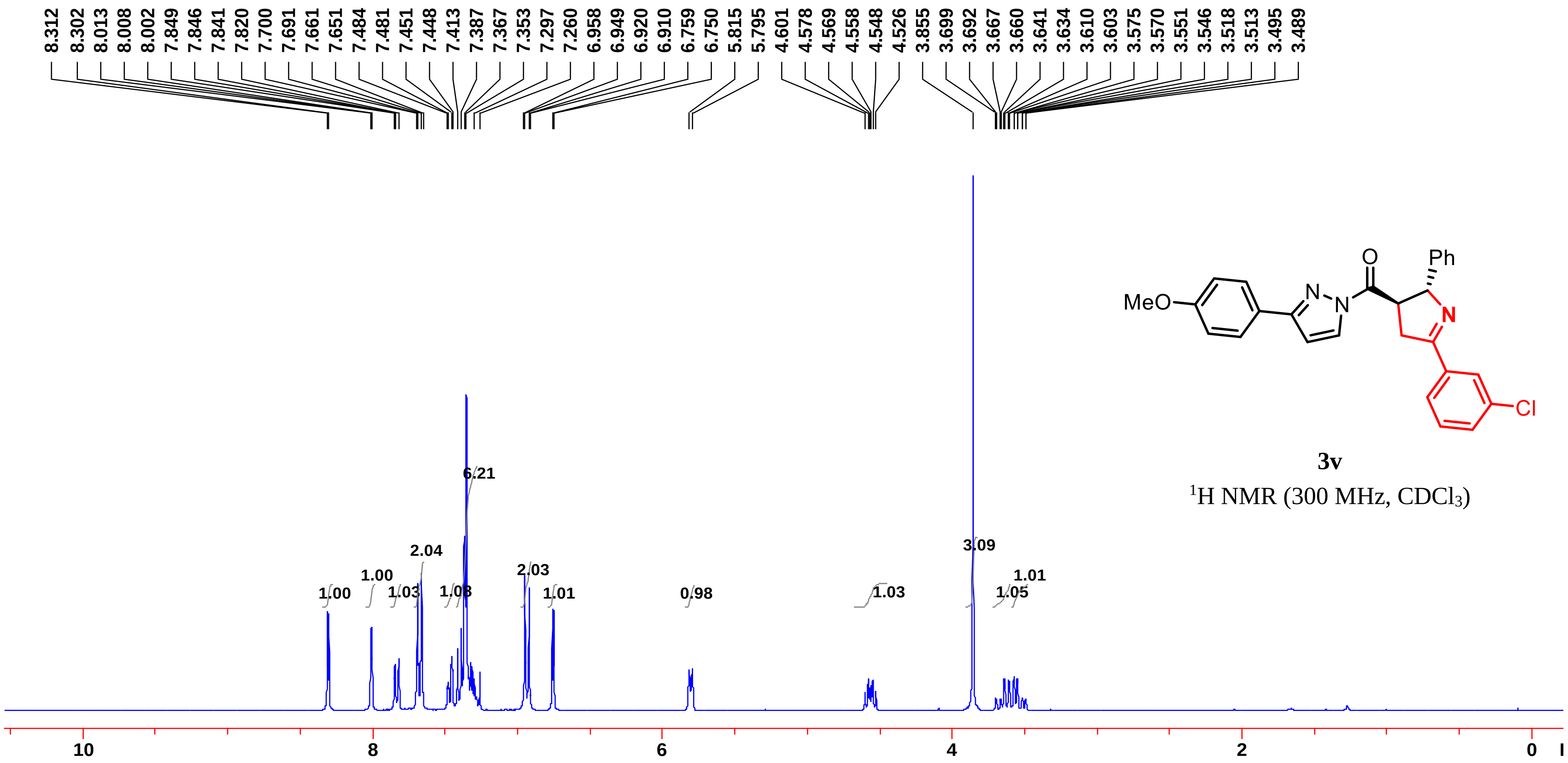

Supplementary Figure 123. ^{1}H NMR spectrum of compound **3v**.

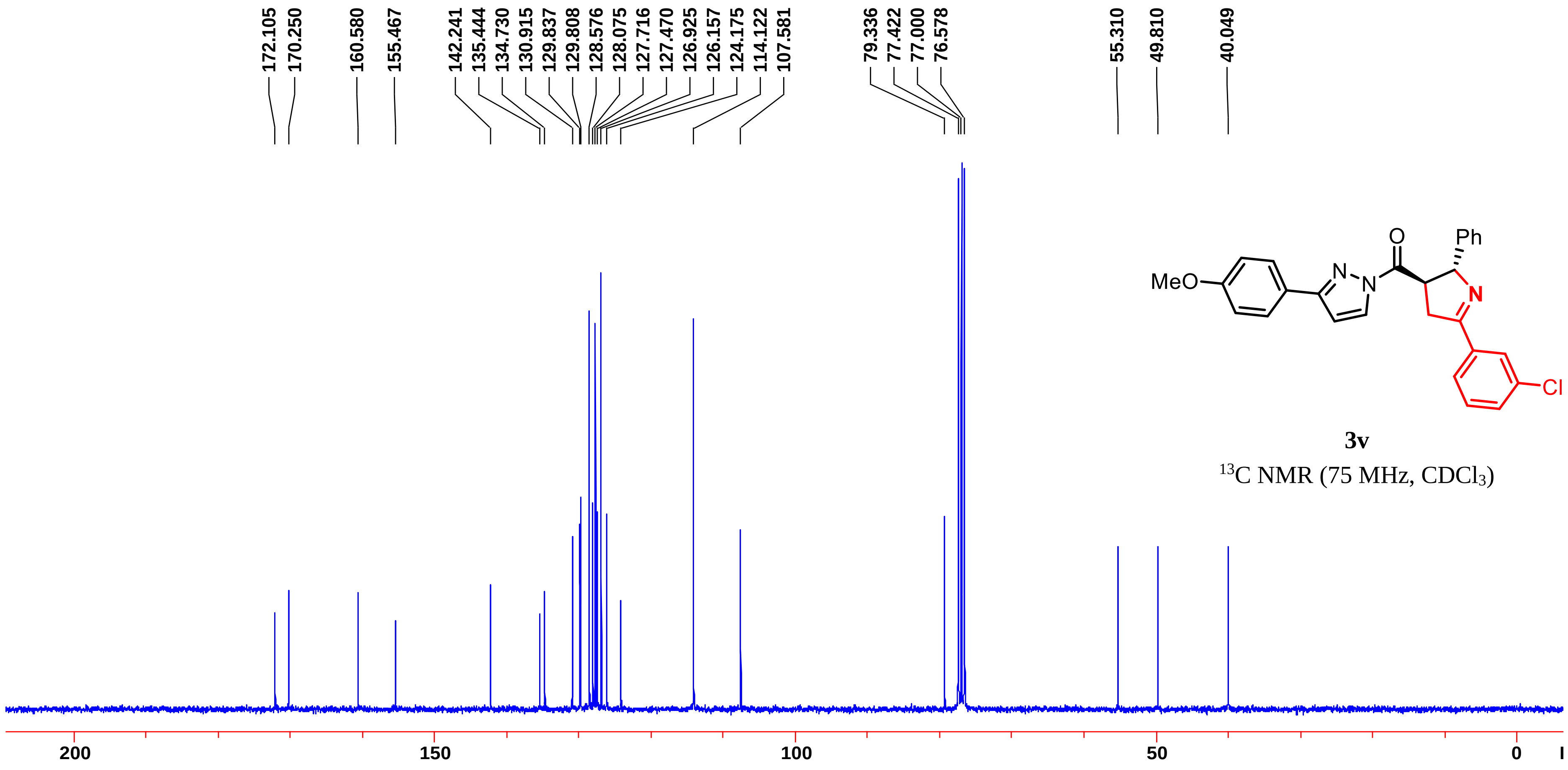

Supplementary Figure 124. ^{13}C NMR spectrum of compound **3v**.

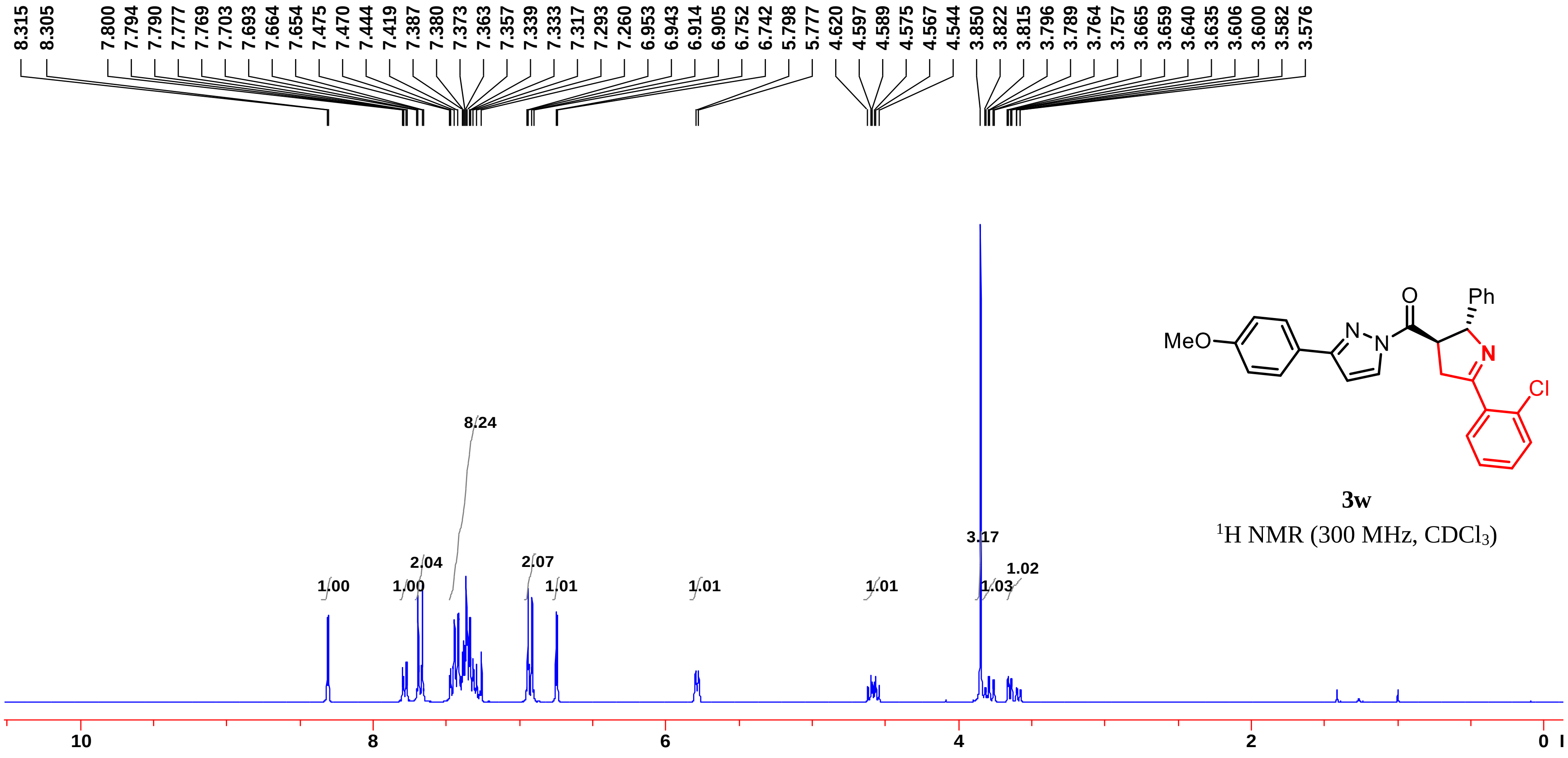

Supplementary Figure 125. ^{1}H NMR spectrum of compound **3w**.

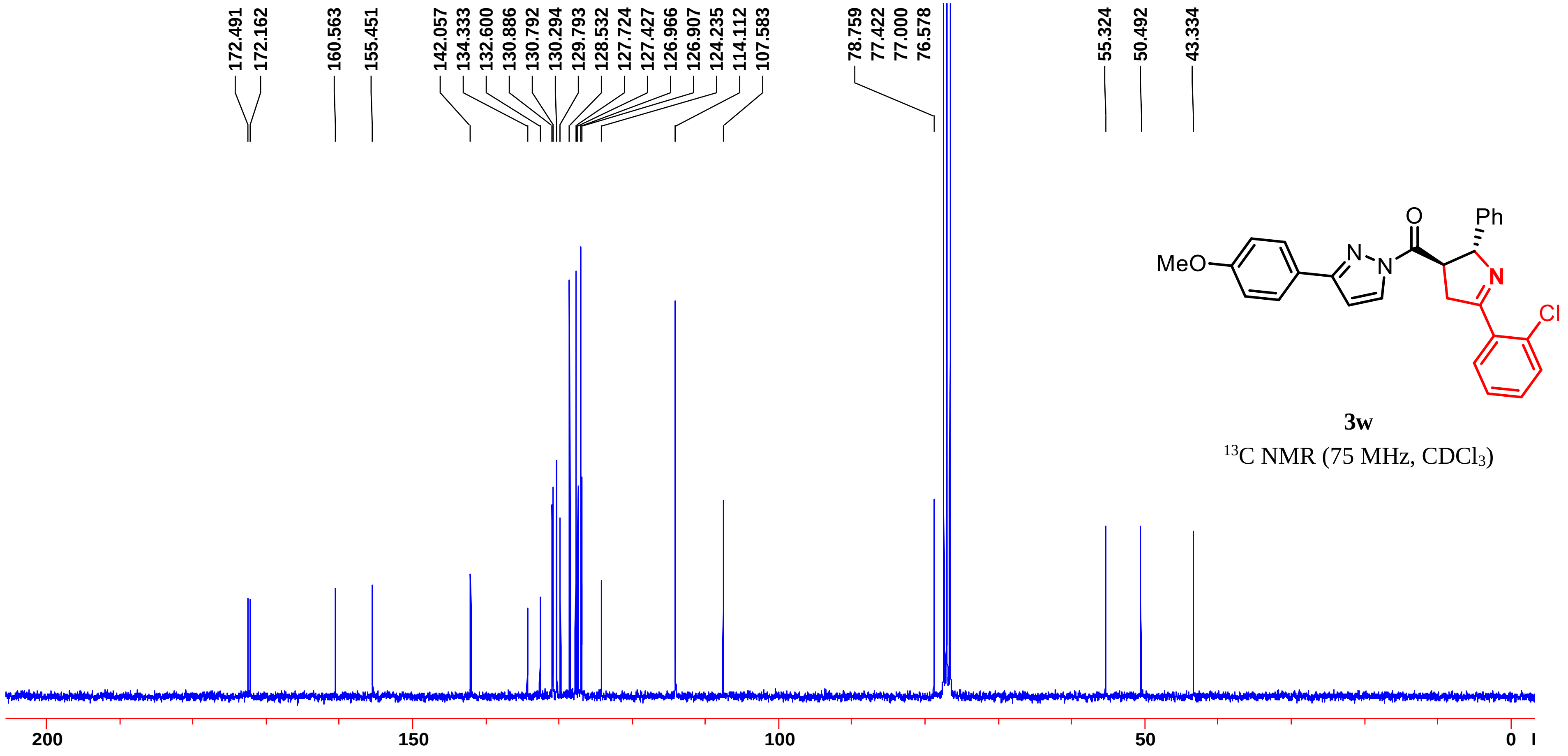

Supplementary Figure 126. ^{13}C NMR spectrum of compound **3w**.

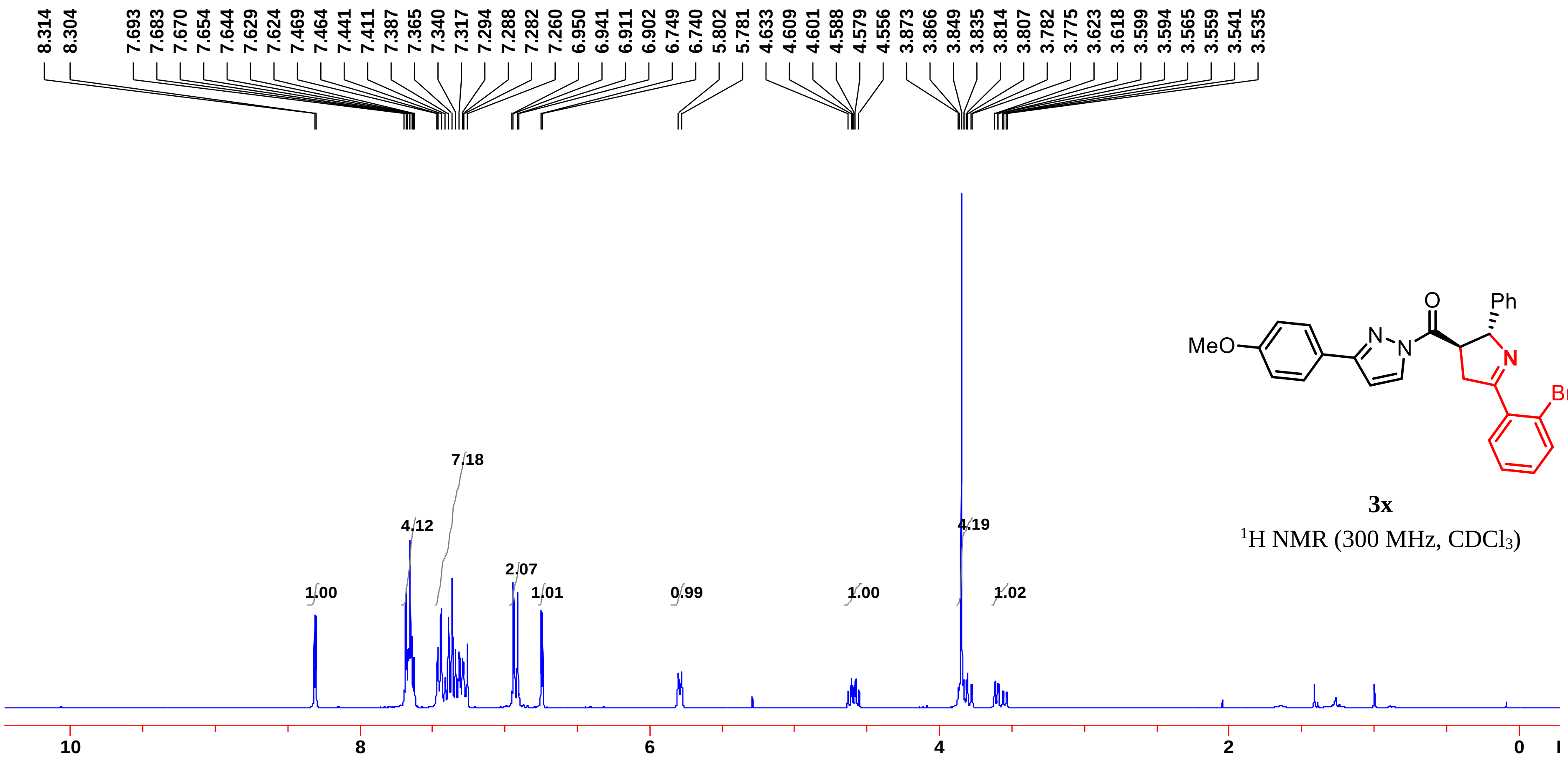

Supplementary Figure 127. ^{1}H NMR spectrum of compound **3x**.

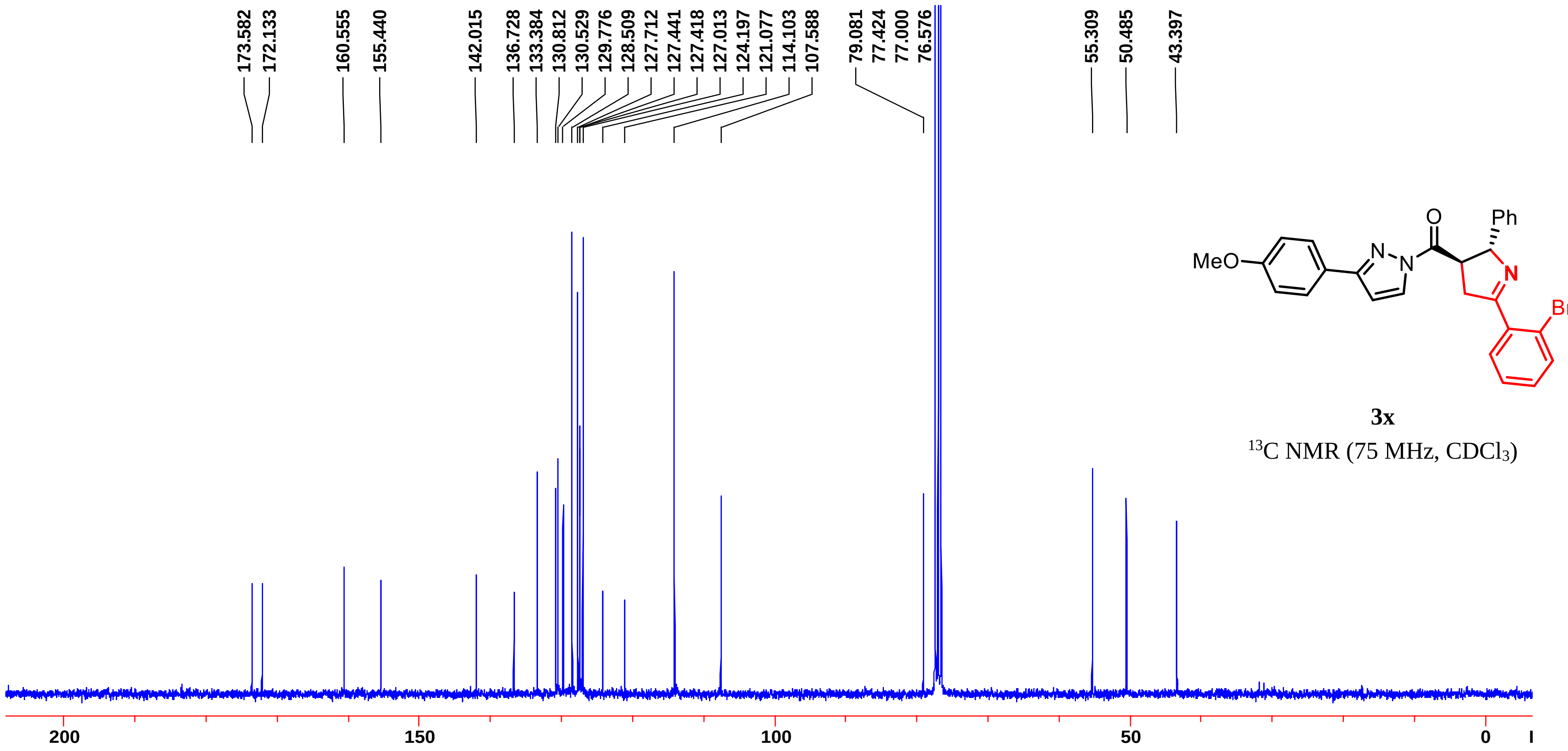

3x

^{13}C NMR (75 MHz, $CDCl_3$)

Supplementary Figure 128. ^{13}C NMR spectrum of compound **3x**.

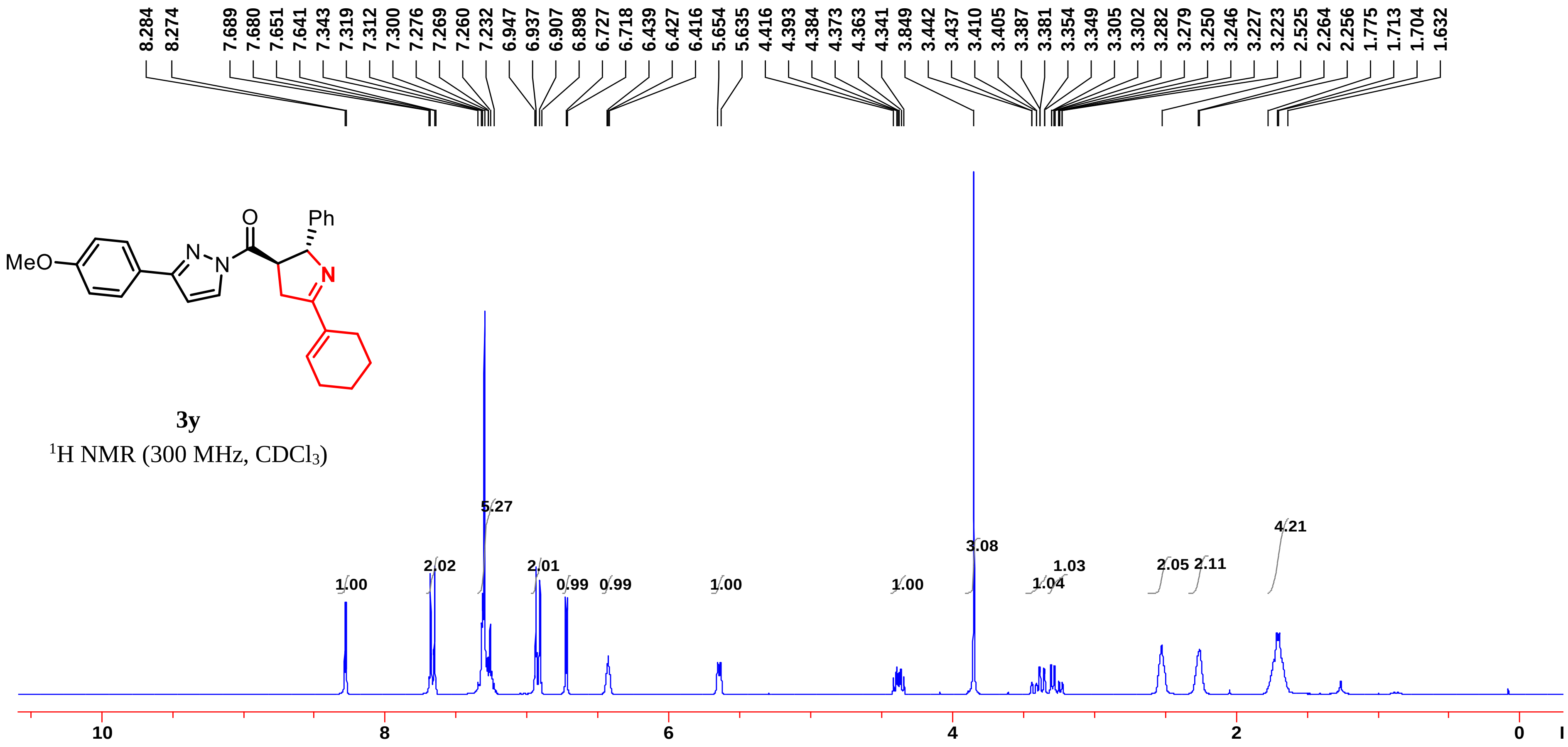

Supplementary Figure 129. ^{1}H NMR spectrum of compound **3y**.

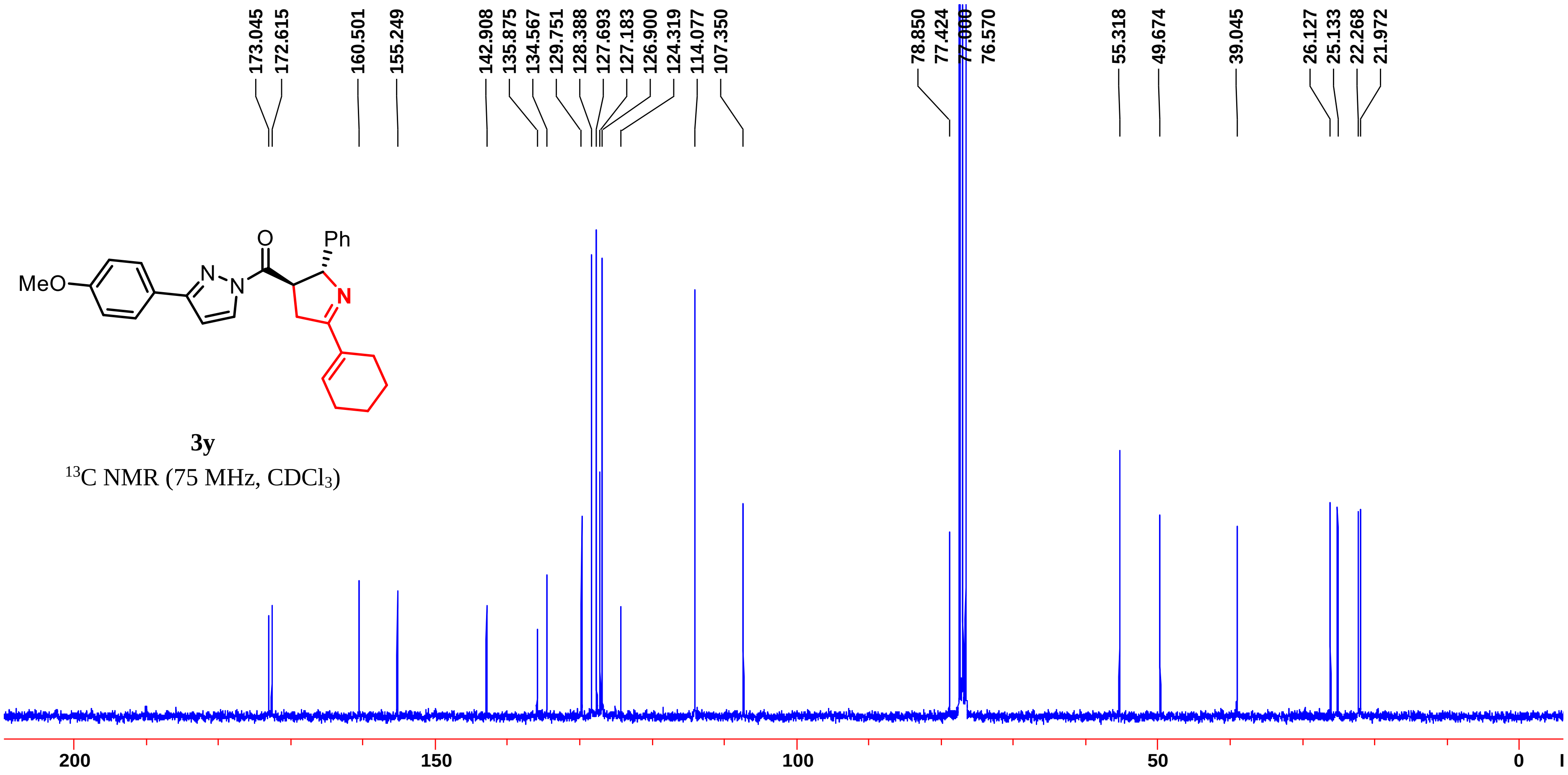

Supplementary Figure 130. ^{13}C NMR spectrum of compound **3y**.

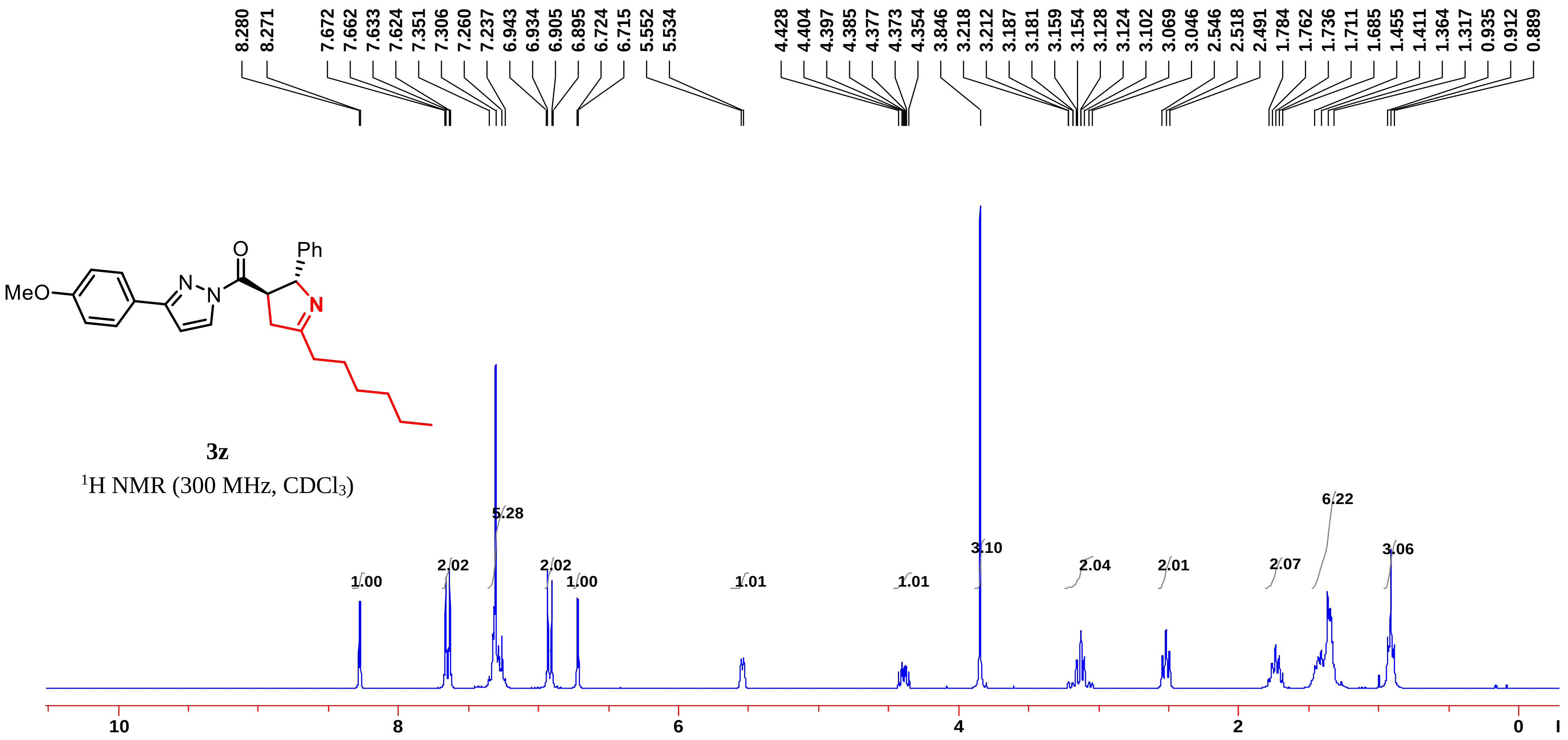

Supplementary Figure 131. ^{1}H NMR spectrum of compound **3z**.

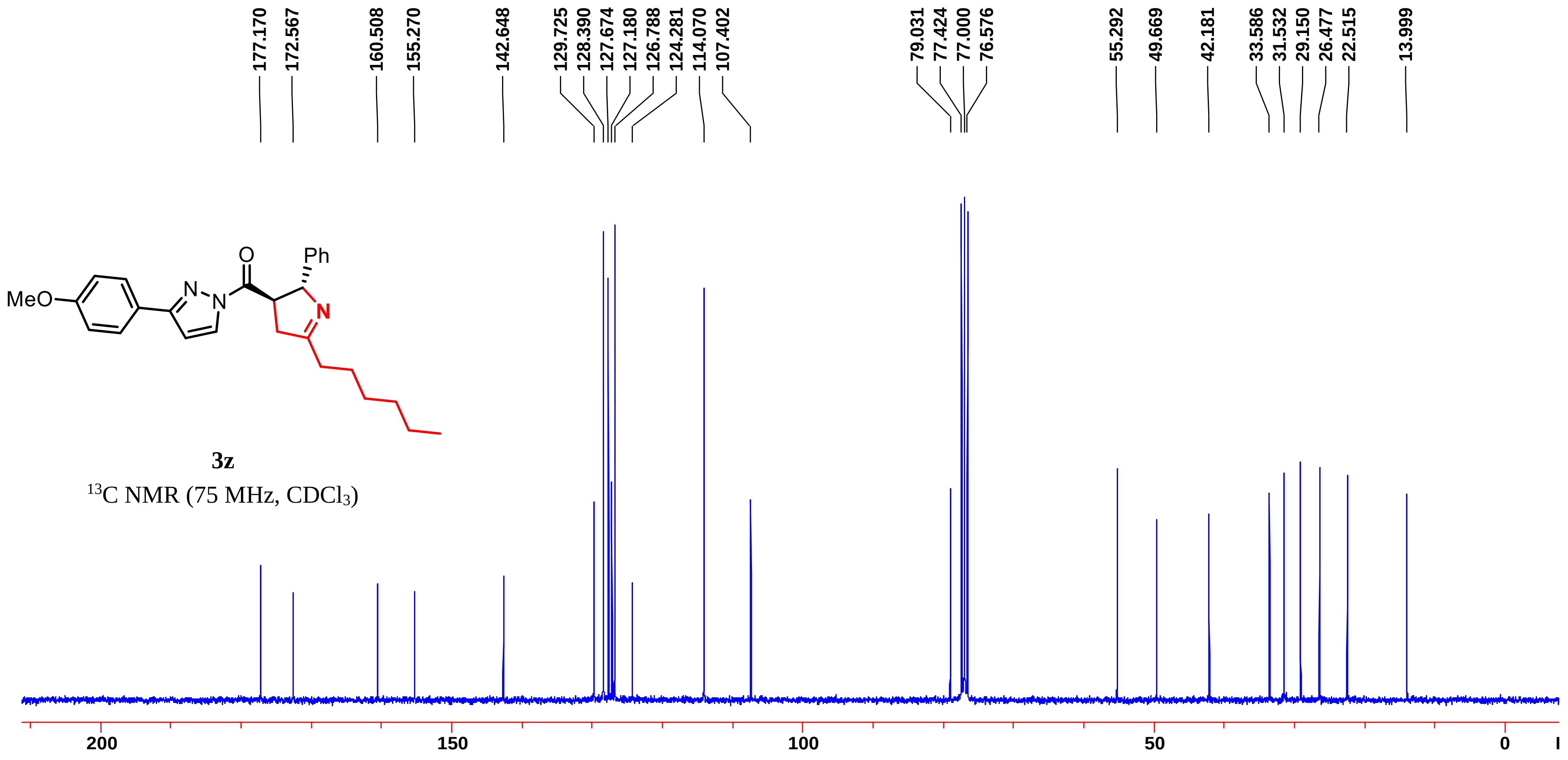

Supplementary Figure 132. ^{13}C NMR spectrum of compound **3z**.

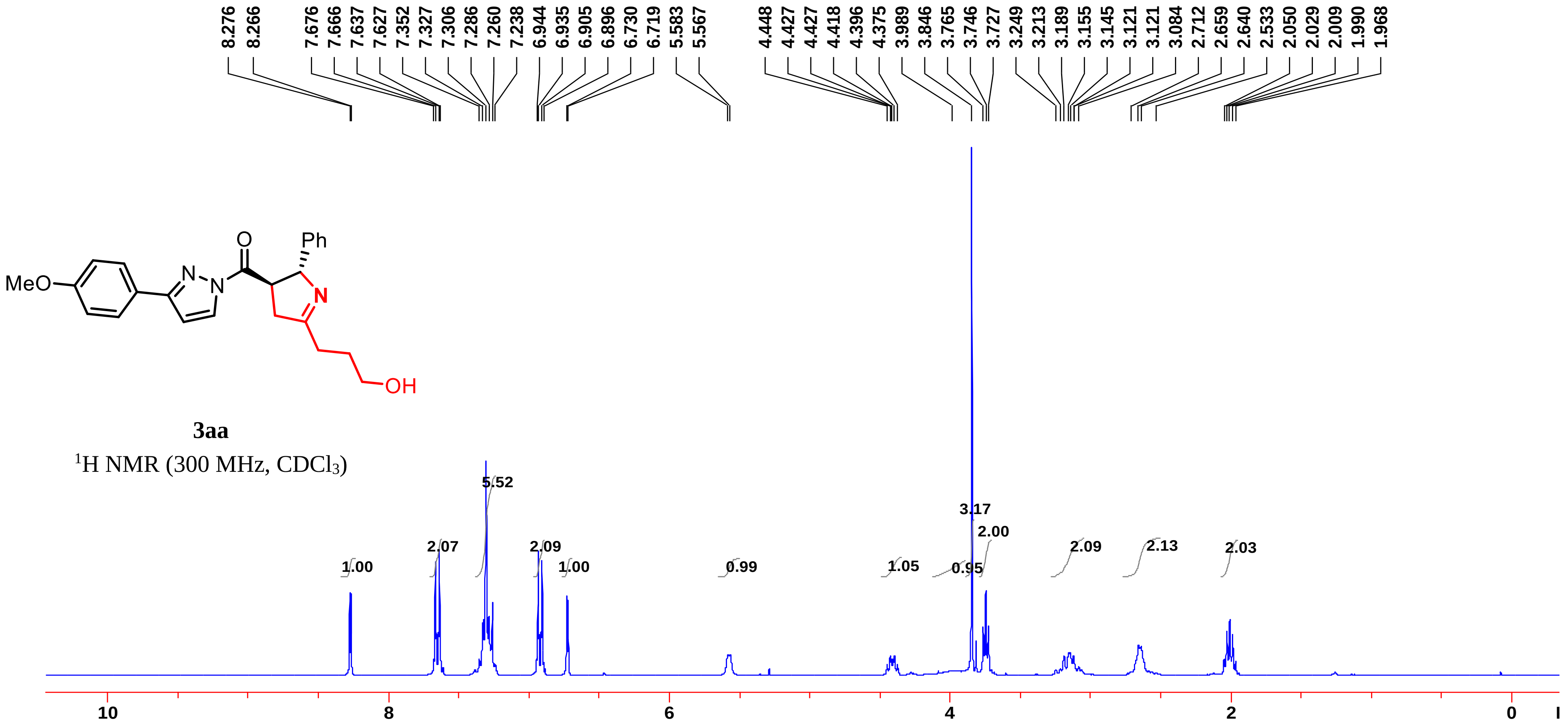

Supplementary Figure 133. ^{1}H NMR spectrum of compound **3aa**.

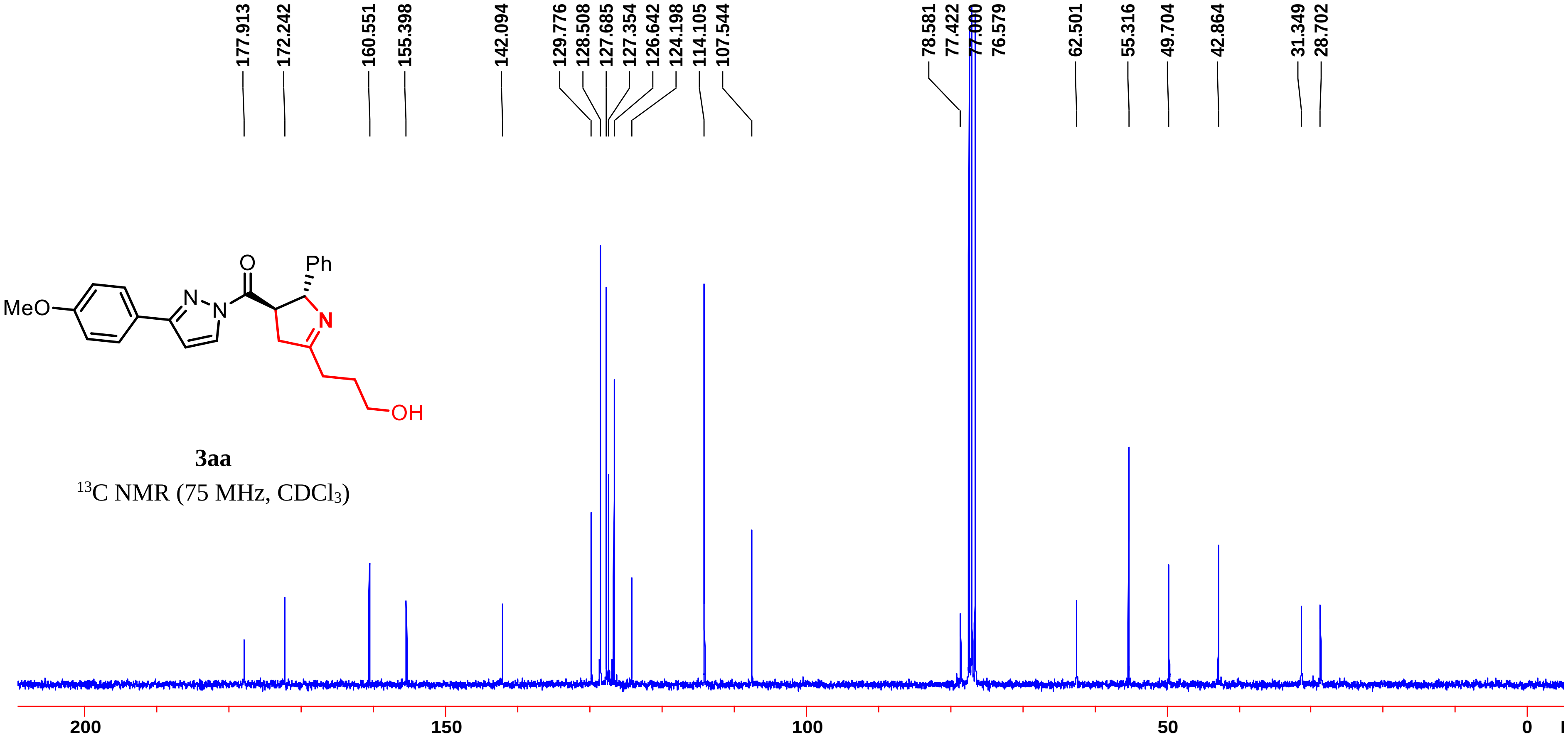

Supplementary Figure 134. ^{13}C NMR spectrum of compound **3aa**.

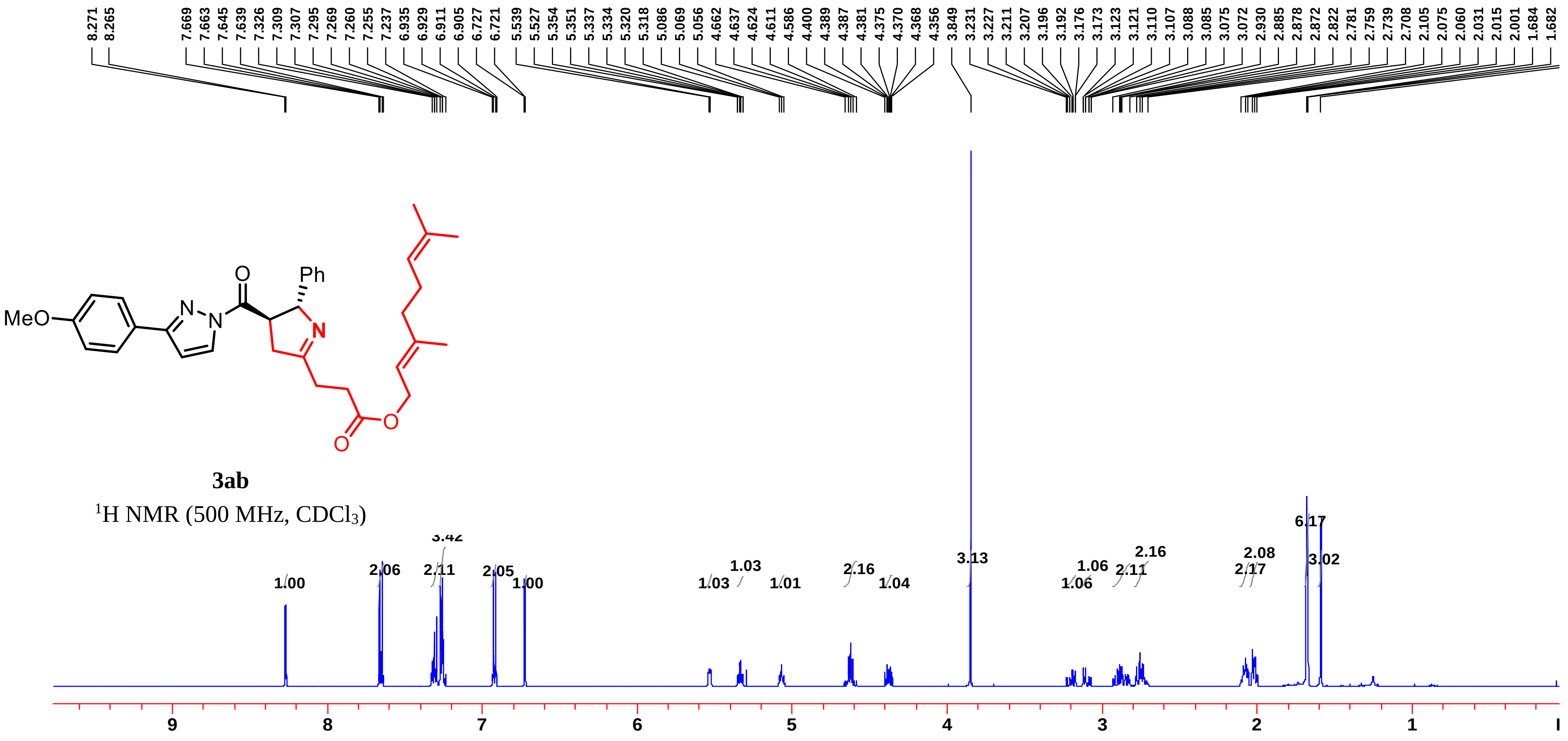

Supplementary Figure 135. ^{1}H NMR spectrum of compound **3ab**.

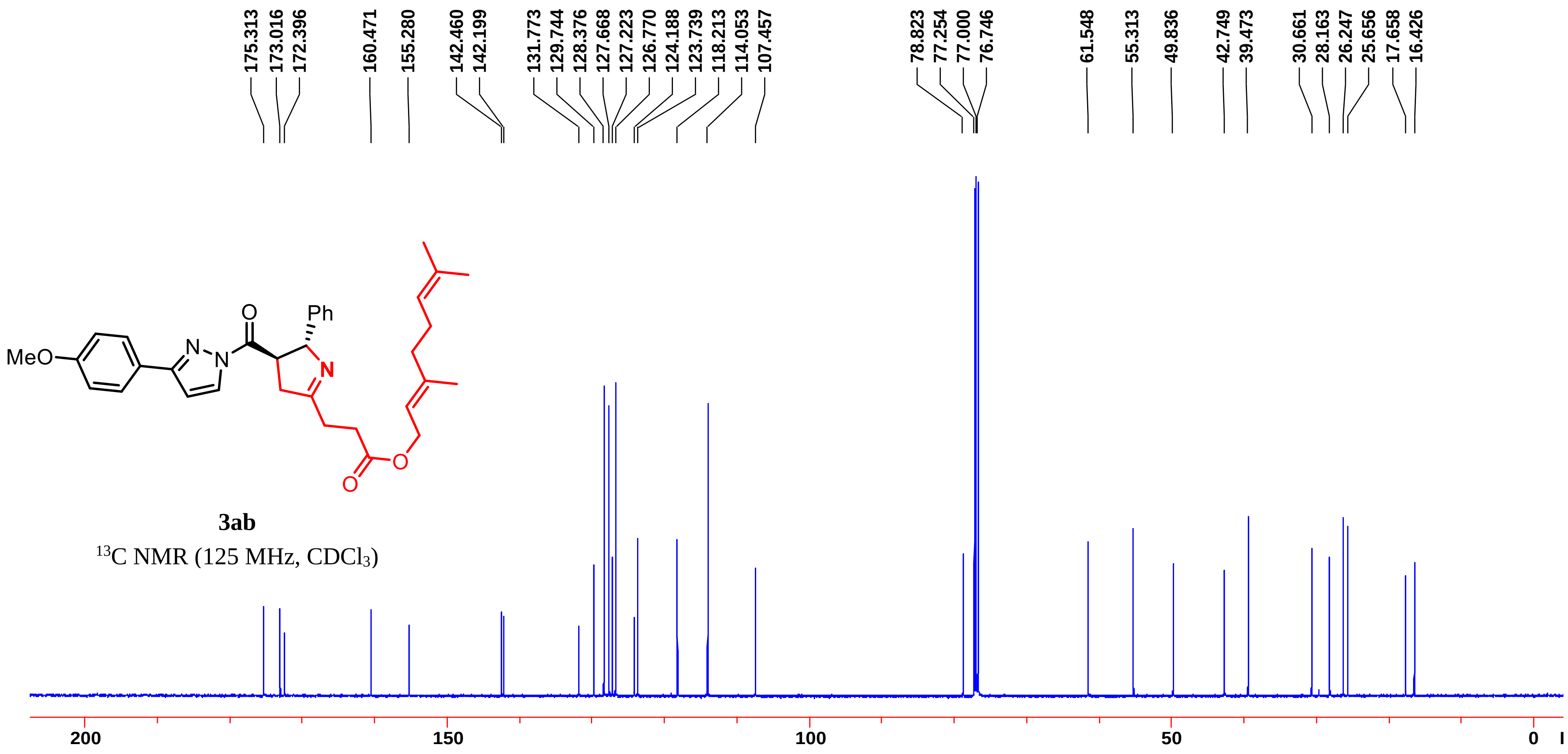

Supplementary Figure 136. ^{13}C NMR spectrum of compound **3ab**.

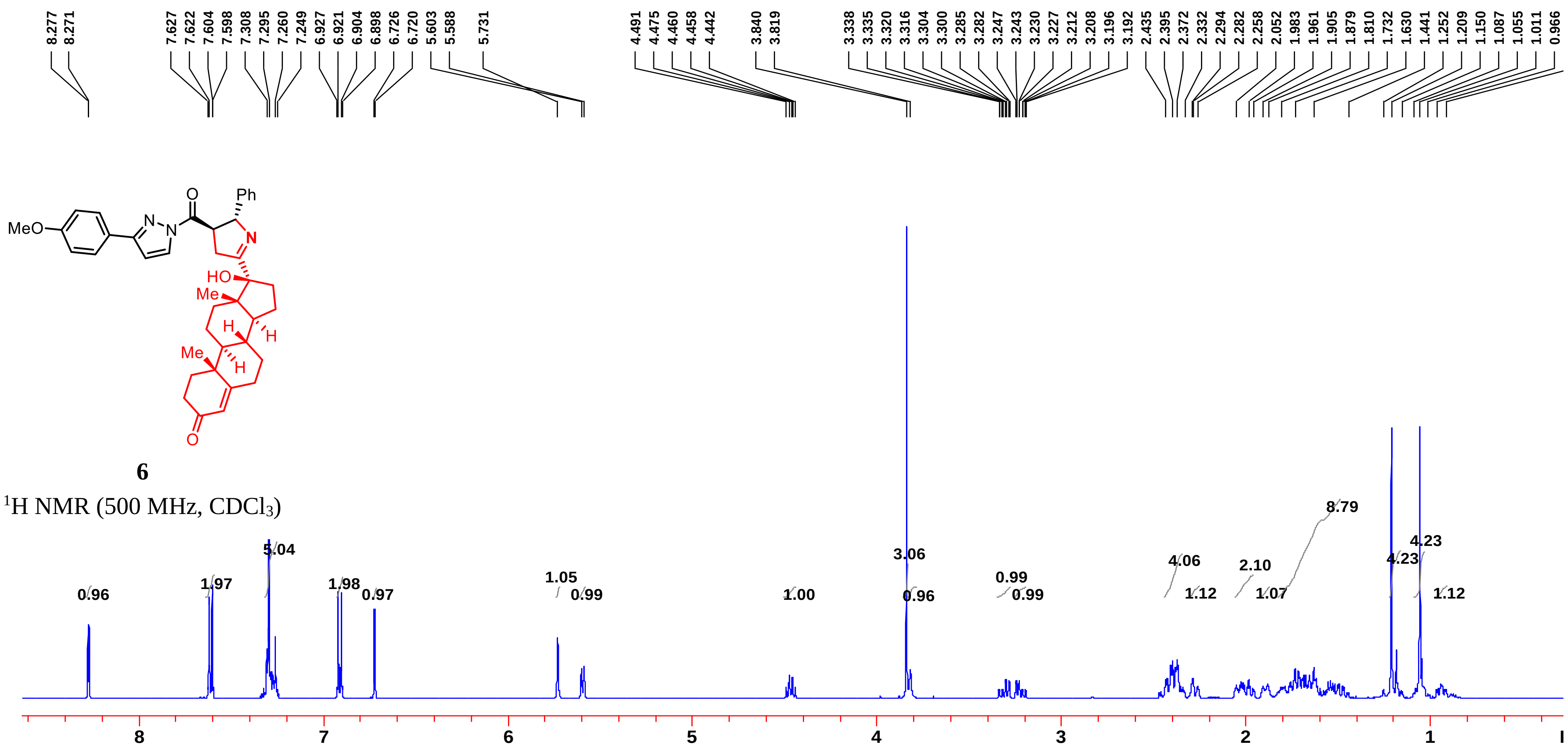

Supplementary Figure 137. ^{1}H NMR spectrum of compound **6**.

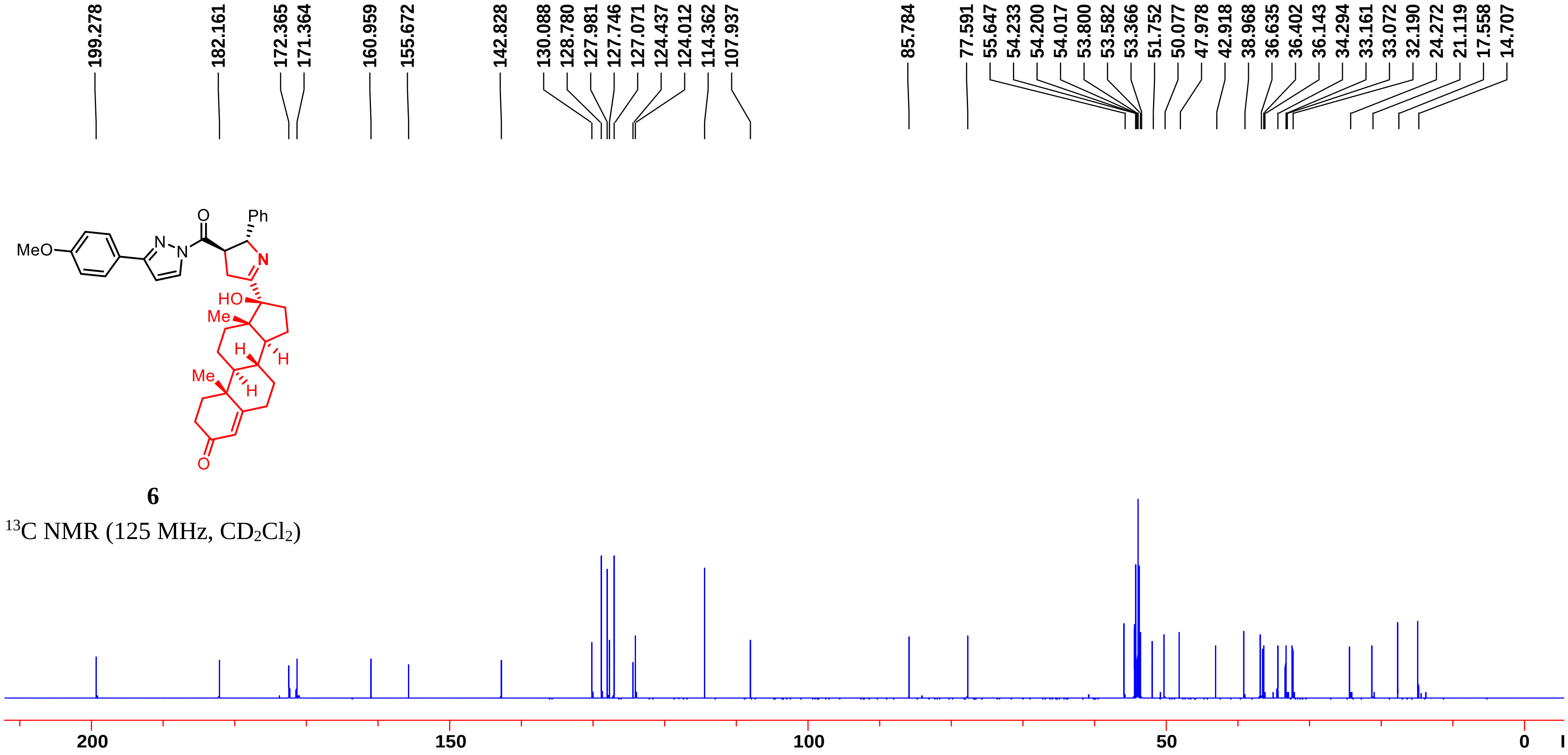

Supplementary Figure 138. ^{13}C NMR spectrum of compound **6**.

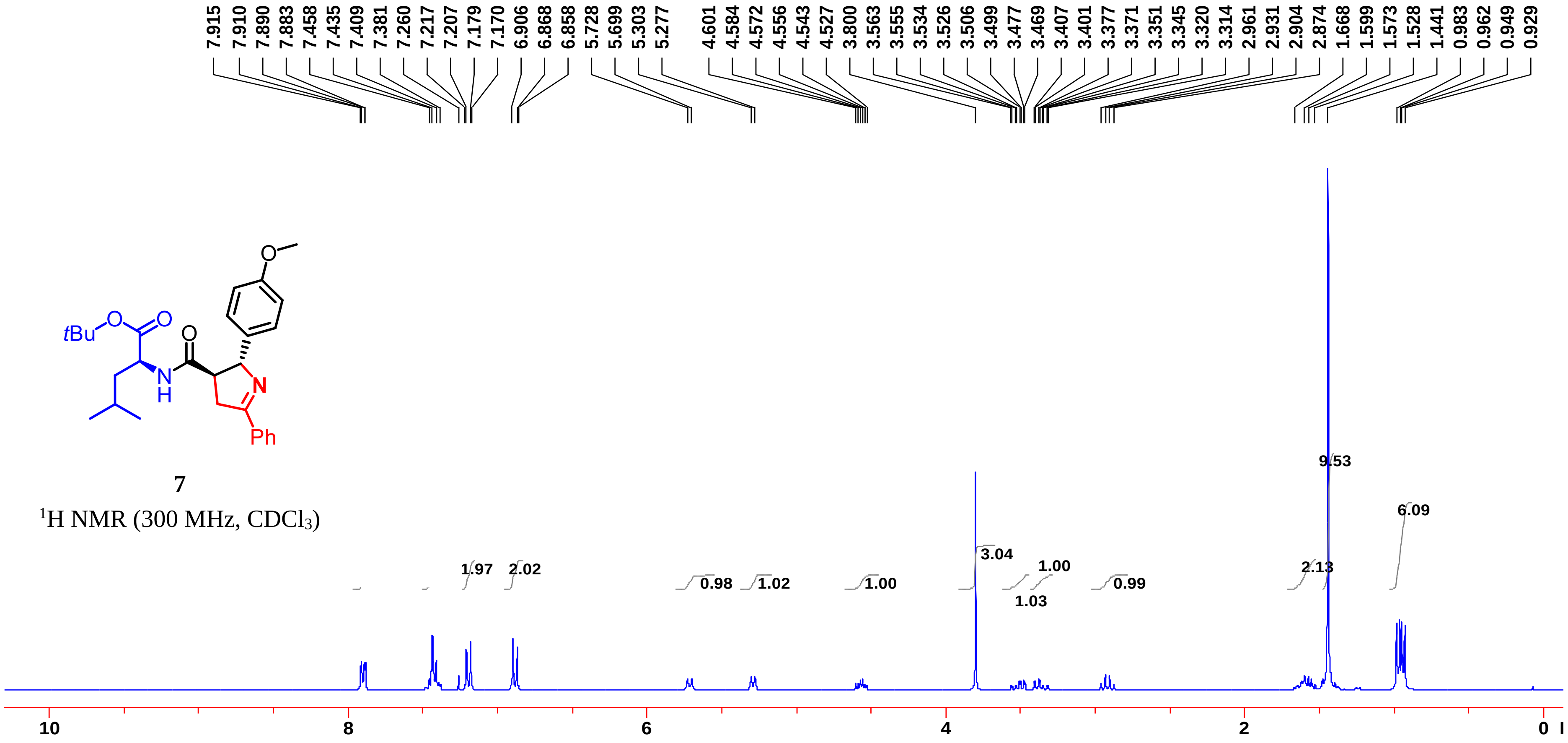

Supplementary Figure 139. ^{1}H NMR spectrum of compound **7**.

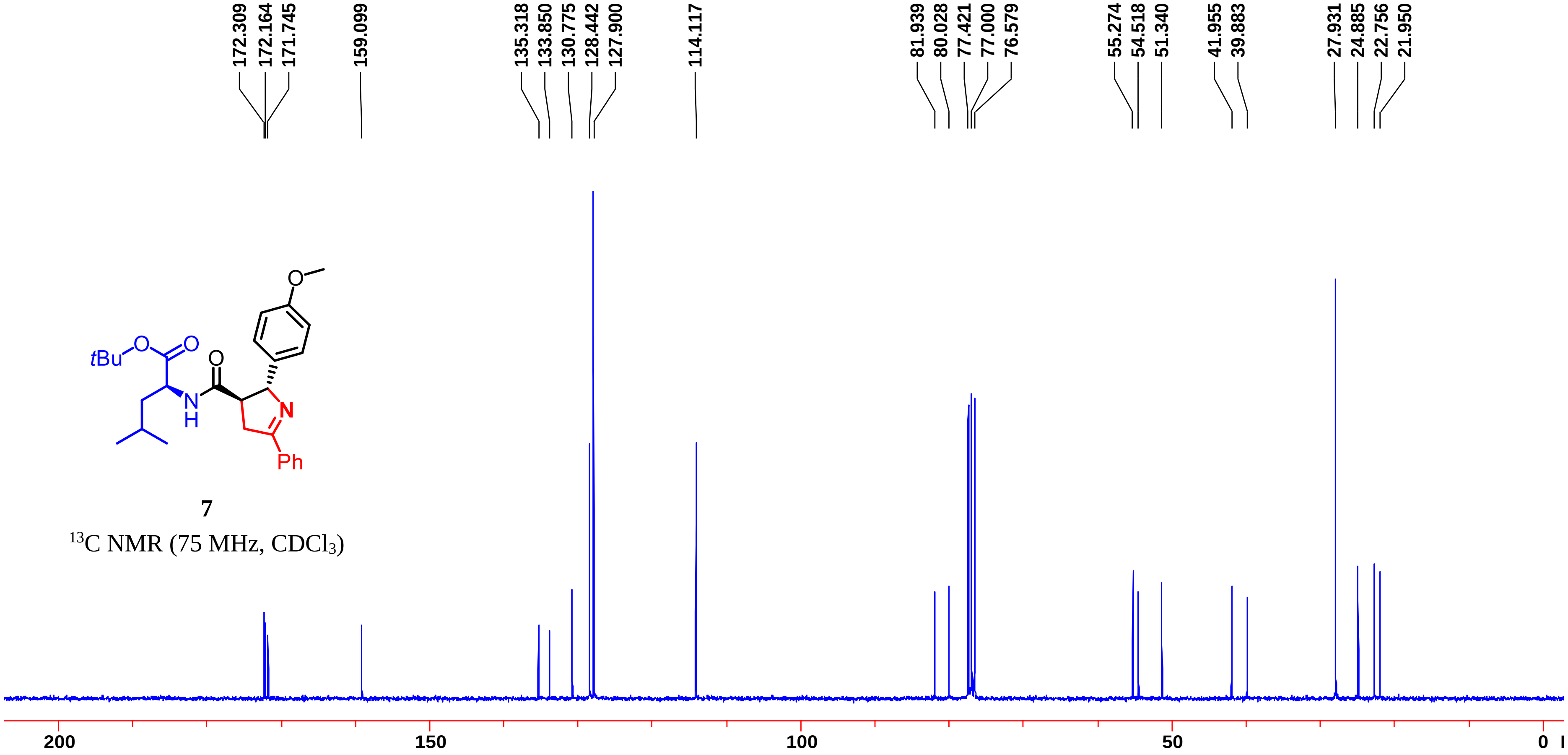

7

^{13}C NMR (75 MHz, $CDCl_3$)

Supplementary Figure 140. ^{13}C NMR spectrum of compound **7**.

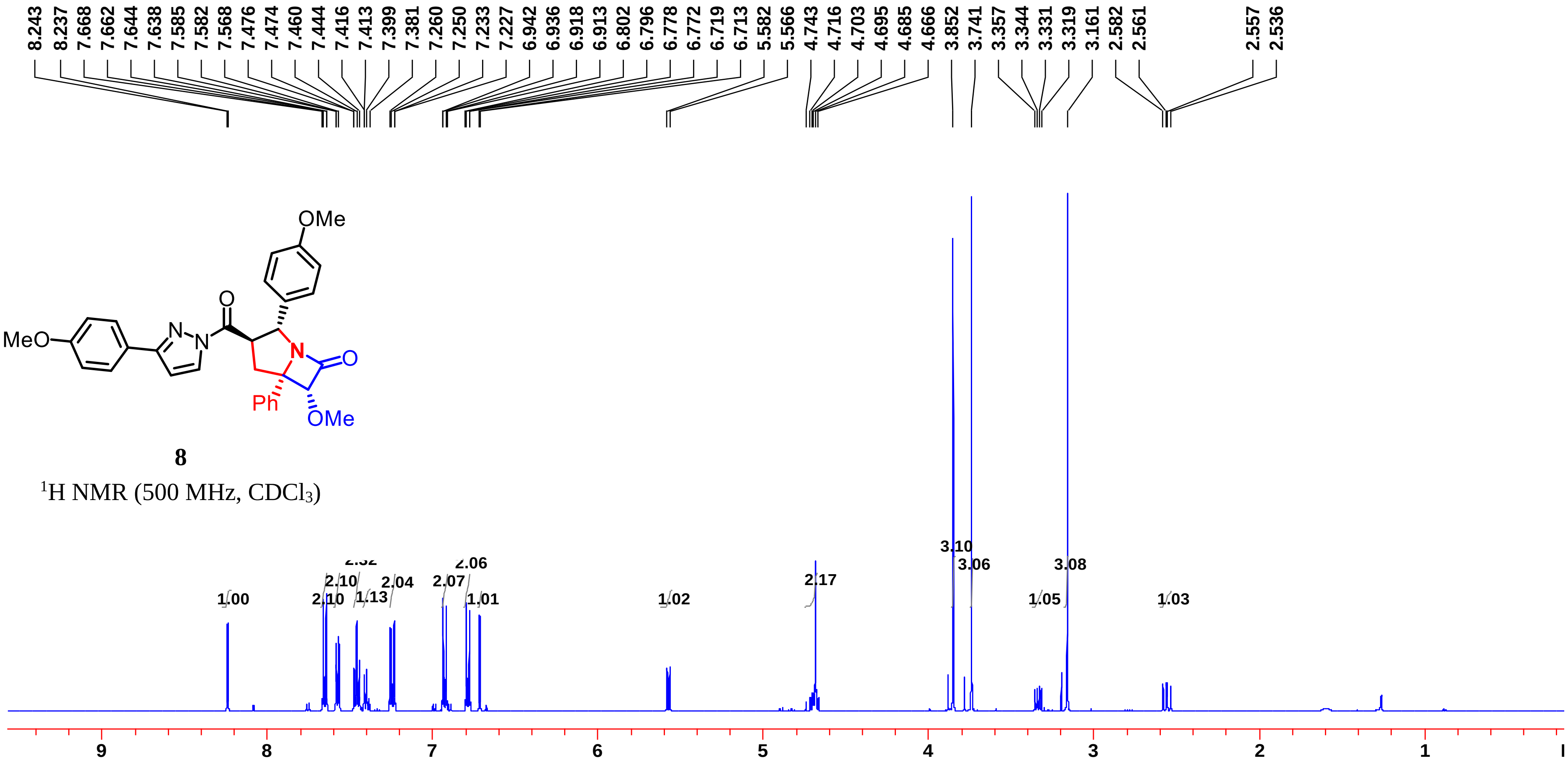

Supplementary Figure 141. ^{1}H NMR spectrum of compound **8**.

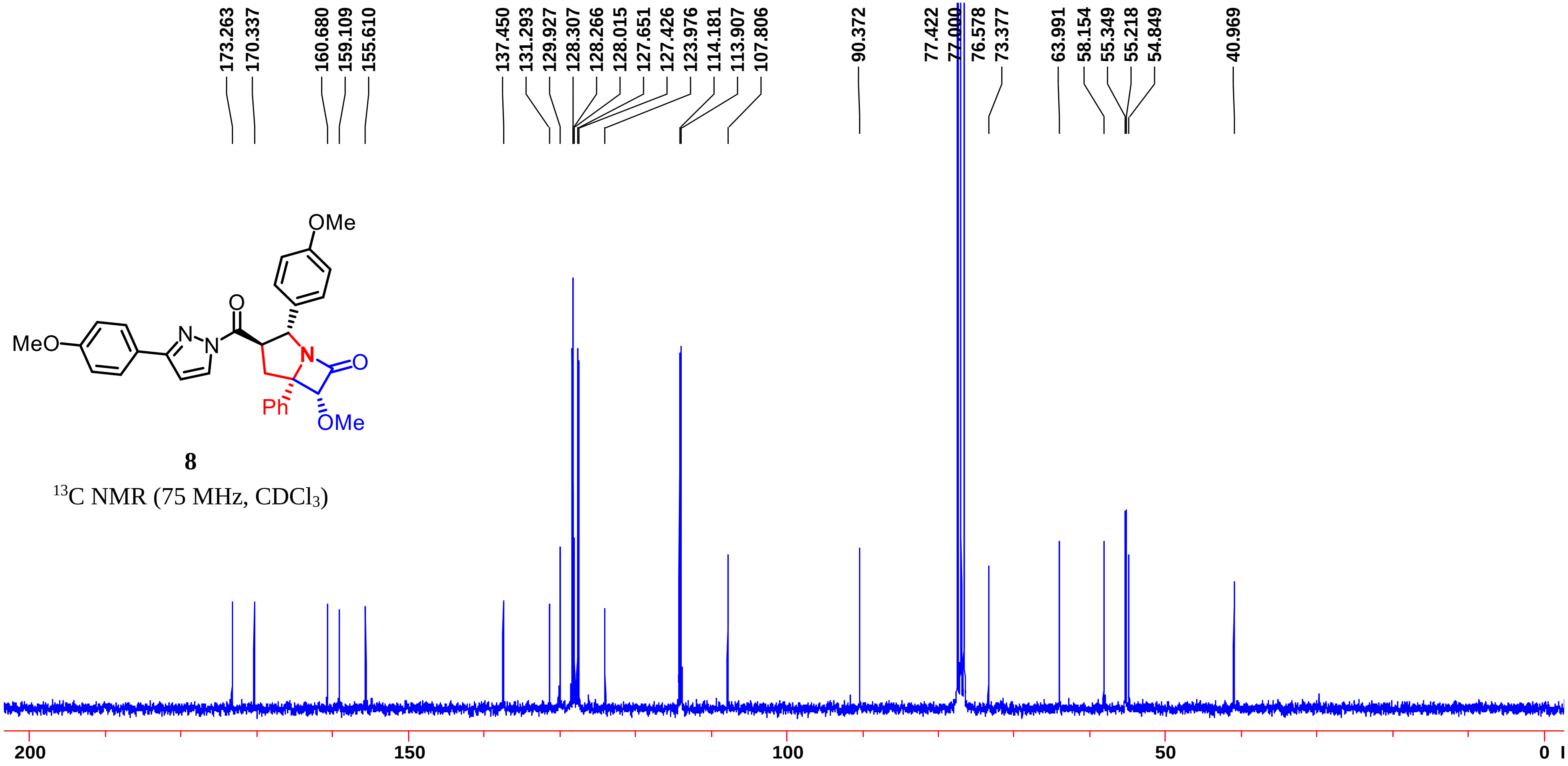

Supplementary Figure 142. ^{13}C NMR spectrum of compound **8**.

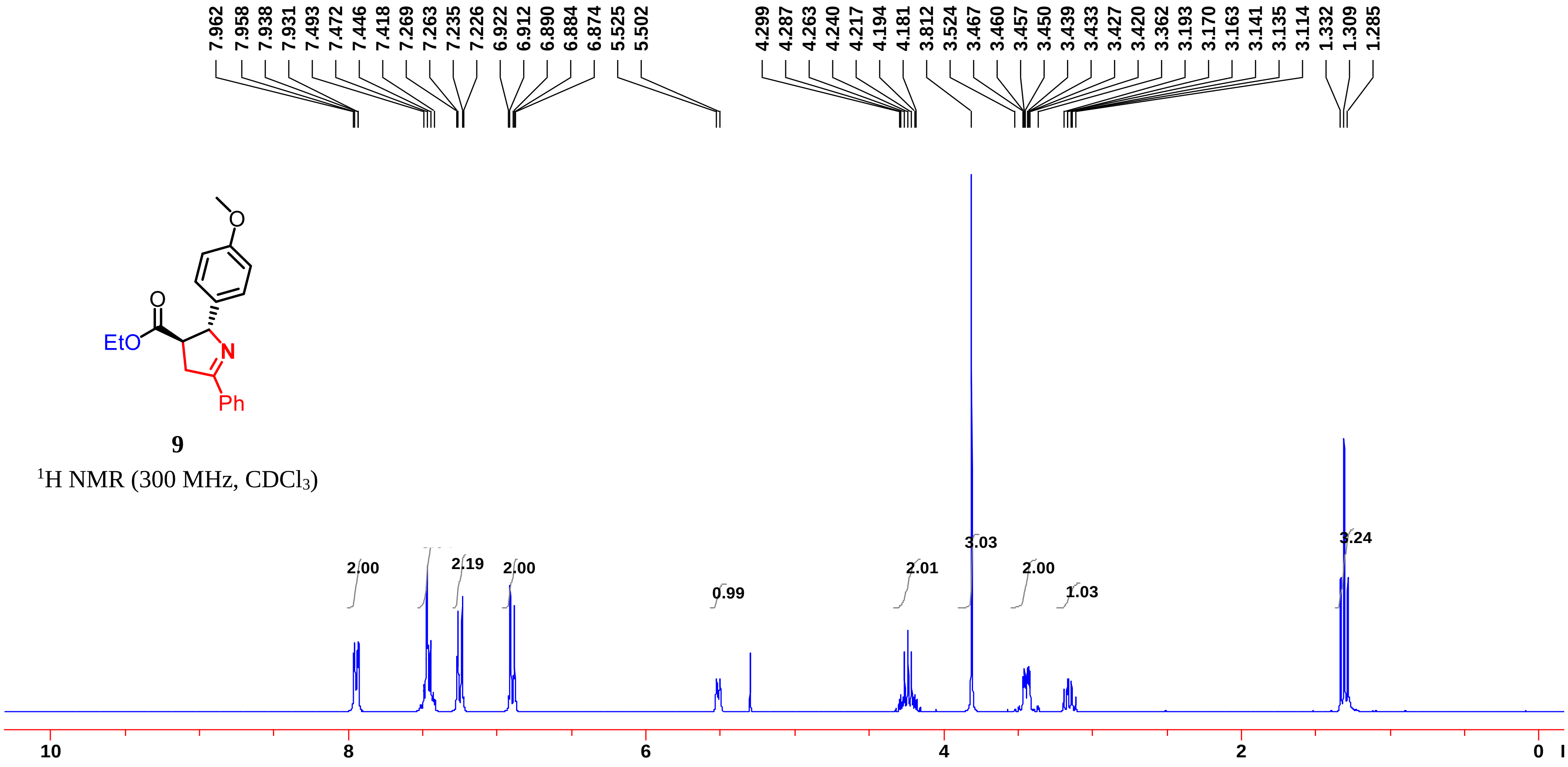

9

^{1}H NMR (300 MHz, $CDCl_3$)

Supplementary Figure 143. ^{1}H NMR spectrum of compound **9**.

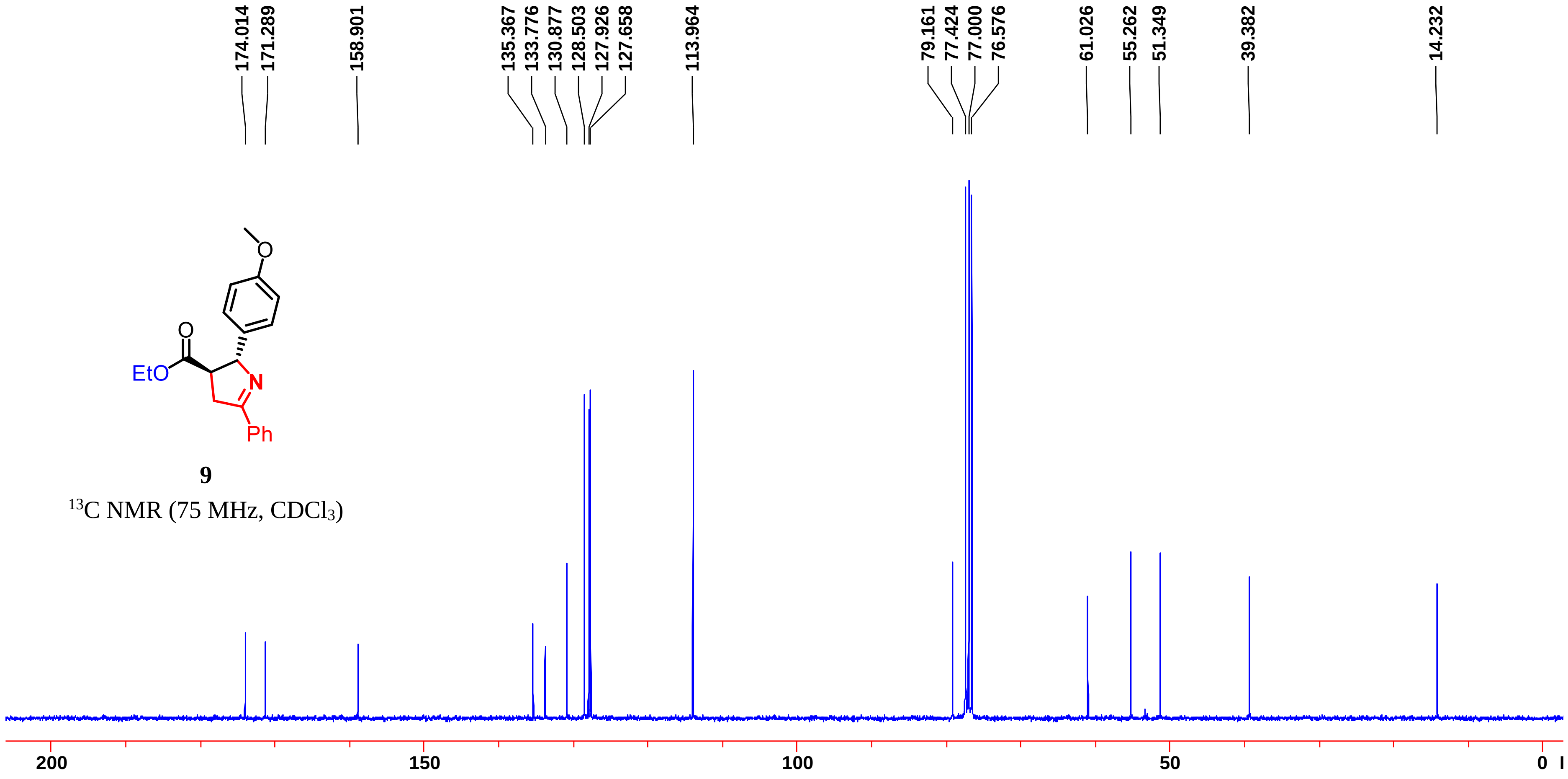

Supplementary Figure 144. ^{13}C NMR spectrum of compound **9**.

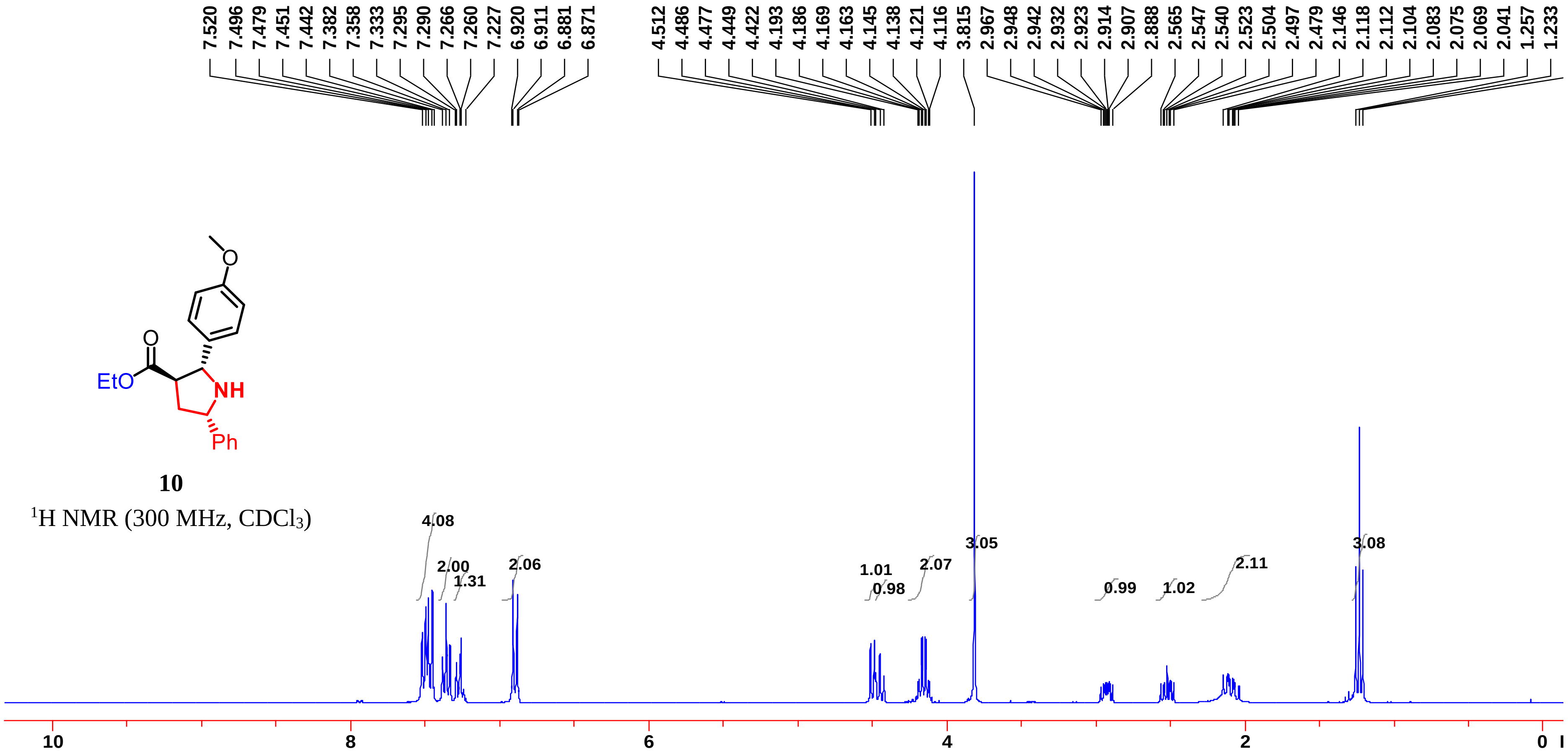

Supplementary Figure 145. ^{1}H NMR spectrum of compound **10**.

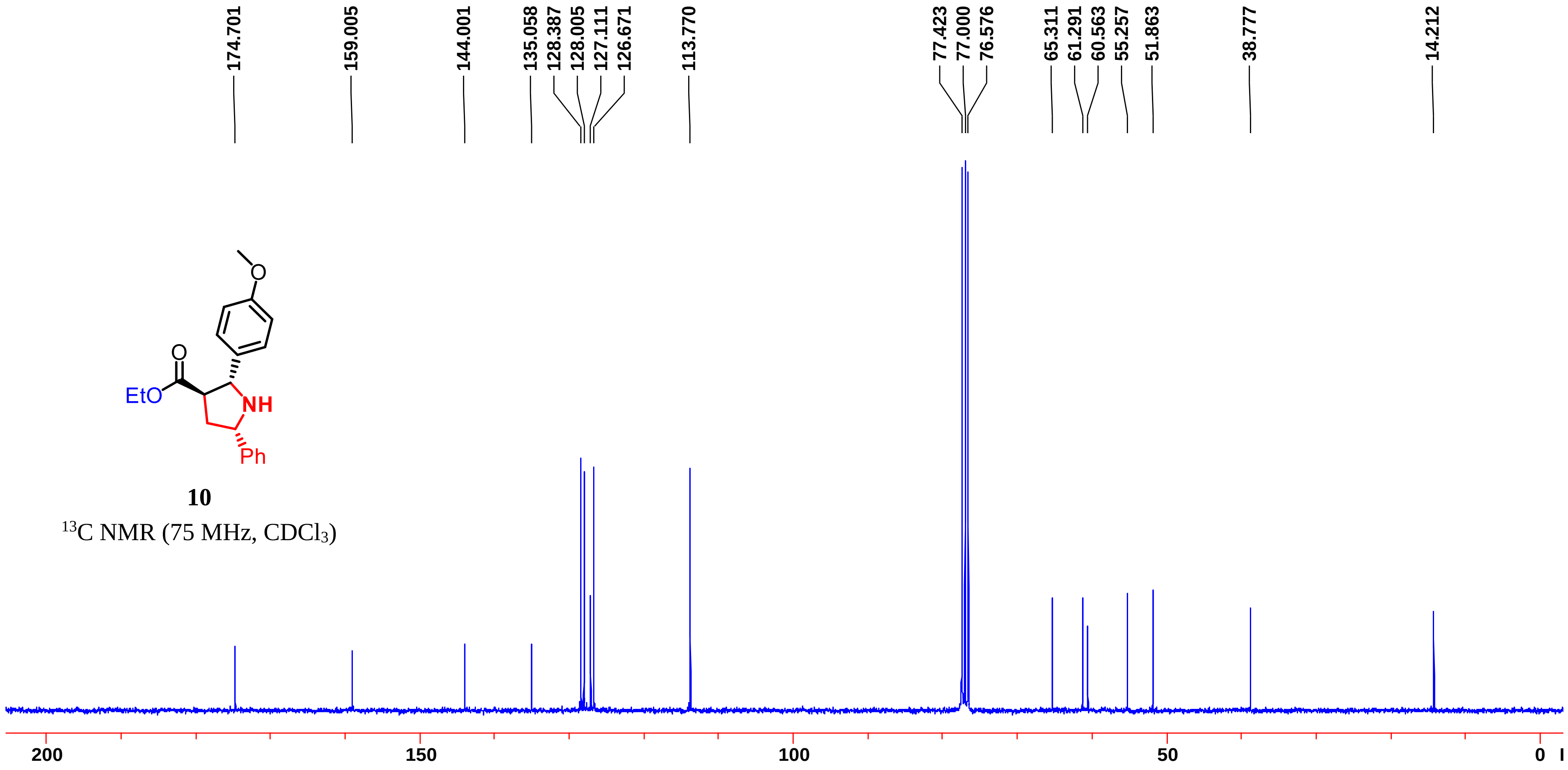

Supplementary Figure 146. ^{13}C NMR spectrum of compound **10**.

Supplementary Tables

Supplementary Table 1. Screening with α,β-unsaturated 2-acylimidazole[a]

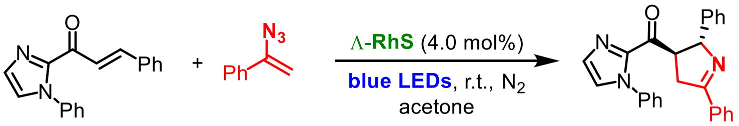

1', 0.1 mmol   **2a**, 3.0 equiv

entry	variations	results
1	None	41% yield; 49% ee; 42% **1'**
2	Without Λ-**RhS**	60% yield; 20% **1'**
3	Dark	No reaction
4	Λ-**IrS** instead of Λ-**RhS**	76% yield; 0% ee
5	CH_2Cl_2 instead of acetone	30% yield; 64% ee;
6	CH_2Cl_2, 1.25 equiv of **2a**	24% yield; 74% ee;

[a]Reaction conditions: α,β-unsaturated 2-acylimidazole **1'** (0.10 mmol), **2a** (0.30 mmol) and Λ-**RhS** (4.0 mol%) in acetone (0.5 mL) were stirred at room temperature under an atmosphere of nitrogen with irradiation of blue LEDs (24 W) for 16 h; NMR yields; enantiomeric excess determined by HPLC analysis on chiral stationary phase.

Supplementary Table 2. Screening with α,β-unsaturated *N*-acylpyrazole 1a[a]

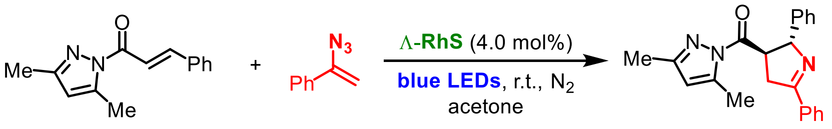

1a, 0.1 mmol   **2a**, 3.0 equiv   **3a**

entry	variations	results
1	None	63% yield; 49% ee
2	Without Λ-**RhS**	Not observed
3	Dark	No reaction
4	MeCN instead of acetone	80% yield; 6% ee
5	CH_2Cl_2 instead of acetone	28% yield; 73% ee
6	Other solvents as THF, DMSO, NMP, PhCl	< 44% ee
7	CH_2Cl_2, 1.5 equiv of **2a**	27% yield; 91% ee
8	CH_2Cl_2, 1.25 equiv of **2a**, 8.0 mol% of Λ-**RhS**	34% yield; 93% ee

[a]Reaction conditions: **1a** (0.10 mmol), **2a** (0.30 mmol) and Λ-**RhS** (4.0 mol%) in acetone (0.5 mL) were stirred at room temperature under an atmosphere of nitrogen with irradiation of blue LEDs (24 W) for 16 h; NMR yields; enantiomeric excess determined by HPLC analysis on chiral stationary phase.

Supplementary Table 3. Effect of reaction concentration[a]

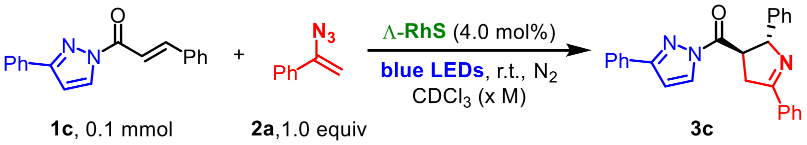

entry	variations	results
1	$CDCl_3$ (0.4 M)	69% yield; 75% ee
2	$CDCl_3$ (0.2 M)	70% yield; 87% ee
3	$CDCl_3$ (0.1 M)	73% yield; 93% ee
4	$CDCl_3$ (0.2 M), Λ-**RhS** (8.0 mol%)	90% yield; 92% ee

[a]Reaction conditions: **1c** (0.10 mmol), **2a** (0.10 mmol) and Λ-**RhS** (4.0 mol%) in $CDCl_3$ were stirred at room temperature under an atmosphere of nitrogen with irradiation of blue LEDs (24 W) for 16 h; NMR yields; enantiomeric excess determined by HPLC analysis on chiral stationary phase.

Supplementary Table 4. Limitations on substrate scope[a]

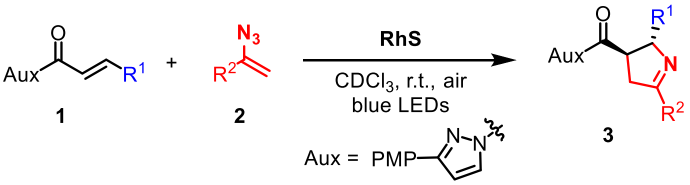

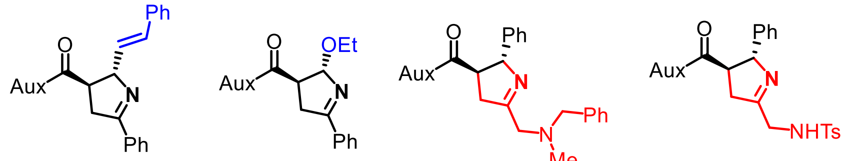

62% yield, 88% ee | No reaction | No reaction | No reaction

[a]Reaction conditions: **1** (0.10 mmol), **2** (0.125 mmol) and **RhS** in $CDCl_3$ were assembled in air and stirred at room temperature under irradiation with blue LEDs (24 W) for 16 h; isolated yield; enantiomeric excess determined by HPLC analysis on chiral stationary phase.

Supplementary Table 5. Crystal data and structure refinement for RhS-1f.

Crystal data

Identification code	hxqH72	
Habitus, colour	block, yellow	
Crystal size	0.20 x 0.18 x 0.10 mm^3	
Crystal system	Monoclinic	
Space group	$P2_1/c$	Z = 4
Unit cell dimensions	a = 13.7758(2) Å	α = 90°.
	b = 15.1202(1) Å	β = 96.139(1)°.
	c = 26.0113(3) Å	γ = 90°.
Volume	5386.9(1) $Å^3$	
Cell determination	56919 peaks with Theta 3.2 to 70.3°.	
Empirical formula	$C_{55.89}$ $H_{55.04}$ $Cl_{0.74}$ F_6 N_4 $O_{2.63}$ P Rh S_2	
Moiety formula	C_{53} H_{48} N_4 O_2 Rh S_2, F_6 P, 0.63(C_4 H_{10} O), 0.37(C H_2 Cl_2)	
Formula weight	1163.07	
Density (calculated)	1.434 Mg/m^3	
Absorption coefficient	4.476 mm^{-1}	
F(000)	2392	

Data collection:

Diffractometer type	STOE STADIVARI
Wavelength	1.54184 Å
Temperature	100(2) K
Theta range for data collection	3.227 to 69.719°.
Index ranges	-16<=h<=7, -18<=k<=16, -31<=l<=30
Data collection software	X-Area Pilatus3_SV 1.31.127.0 (STOE, 2016)[1]
Cell refinement software	X-Area Recipe 1.33.0.0 (STOE, 2015)[2]
Data reduction software	X-Area Integrate 1.71.0.0 (STOE, 2016)[3]
	X-Area LANA 1.68.2.0 (STOE, 2016)[4]

Solution and refinement:

Reflections collected	49551
Independent reflections	10012 [R(int) = 0.0208]
Completeness to theta = 67.684°	99.5 %
Observed reflections	9039[$I > 2\sigma(I)$]
Reflections used for refinement	10012
Absorption correction	Semi-empirical from equivalents[4]
Max. and min. transmission	1.0000 and 0.3908
Largest diff. peak and hole	0.871 and -0.406 e.$Å^{-3}$
Solution	dual space algorithm
Refinement	Full-matrix least-squares on F^2
Treatment of hydrogen atoms	Calculated positions, constr ref.
Programs used	XT V2014/1 (Bruker AXS Inc., 2014)[5]
	SHELXL-2016/6 (Sheldrick, 2016)[6]
	DIAMOND (Crystal Impact)[7]
	ShelXle (Hübschle, Sheldrick, Dittrich, 2011)[8]
Data / restraints / parameters	10012 / 49 / 730
Goodness-of-fit on F^2	1.069
R index (all data)	wR2 = 0.0741
R index conventional [I>2sigma(I)]	R1 = 0.0266

Supplementary Table 6. Crystal data and structure refinement for 3k.

Crystal data

Identification code	hxqH27	
Habitus, colour	plate, colorless	
Crystal size	0.17 x 0.17 x 0.04 mm^3	
Crystal system	Monoclinic	
Space group	$P2_1$	Z = 2
Unit cell dimensions	a = 10.5089(2) Å	α = 90°.
	b = 7.7040(1) Å	β = 101.167(1)°.
	c = 14.2627(2) Å	γ = 90°.
Volume	1132.85(3) $Å^3$	
Cell determination	28484 peaks with Theta 3.2 to 69.6°.	
Empirical formula	$C_{27} H_{22} Br N_3 O_2$	
Moiety formula	$C_{27} H_{22} Br N_3 O_2$	
Formula weight	500.38	
Density (calculated)	1.467 Mg/m^3	
Absorption coefficient	2.713 mm^{-1}	
F(000)	512	

Data collection:

Diffractometer type	STOE STADIVARI
Wavelength	1.54184 Å
Temperature	100(2) K
Theta range for data collection	4.288 to 69.163°.
Index ranges	-10<=h<=12, -9<=k<=8, -17<=l<=8
Data collection software	X-Area Pilatus3_SV 1.31.127.0 (STOE, 2016)[1]
Cell refinement software	X-Area Recipe 1.33.0.0 (STOE, 2015)[2]
Data reduction software	X-Area Integrate 1.71.0.0 (STOE, 2016)[3]
	X-Area LANA 1.68.2.0 (STOE, 2016)[4]

Solution and refinement:

Reflections collected	17616
Independent reflections	4091 [R(int) = 0.0176]
Completeness to theta = 67.684°	99.3 %
Observed reflections	4006[$I > 2\sigma(I)$]
Reflections used for refinement	4091
Absorption correction	Semi-empirical from equivalents[4]
Max. and min. transmission	1.0000 and 0.4410
Flack parameter (absolute struct.)	-0.024(10)[9]
Largest diff. peak and hole	0.285 and -0.244 e.$Å^{-3}$
Solution	dual space algorithm
Refinement	Full-matrix least-squares on F^2
Treatment of hydrogen atoms	Calculated positions, constr. ref.
Programs used	XT V2014/1 (Bruker AXS Inc., 2014)[5]
	SHELXL-2016/6 (Sheldrick, 2016)[6]
	DIAMOND (Crystal Impact)[7]
	ShelXle (Hübschle, Sheldrick, Dittrich, 2011)[8]
Data / restraints / parameters	4091 / 1 / 299
Goodness-of-fit on F^2	1.050
R index (all data)	wR2 = 0.0547
R index conventional [I>2sigma(I)]	R1 = 0.0208

Supplementary Table 7. Excited singlet state of 1f, RhS, and RhS-1f

	Excited State	TD-DFT SP wavelength	Oscillator strength
**1f**	1	379.74 nm	f= 0.4364
	2	309.53 nm	f= 0.0818
	3	299.11 nm	f= 0.6852
**RhS**	1	402.24 nm	f=0.0614
	2	393.71 nm	f=0.0014
	3	371.94 nm	f=0.0429
**RhS-1f**	1	499.36 nm	f=0.0008
	2	435.40 nm	f=0.0073
	3	429.95 nm	f=0.1423
	4	415.93 nm	f=0.0047

Supplementary Table 8. NOESY cross peaks and molecular structure of compound 8.

No.	Positions	Configuration	No.	Positions	Configuration
1	H-6 – CH_3-8		6	2’’’’/6’’’’ – H-4α	α-substitution of 5-Ph
2	H-2 – H-4β	H-4β	7	2’’’’/6’’’’ – CH_3-8	cis-config. 5-Ph / 6-OMe
3	H-6 – H-4β	α-substitution of H-6	8	2’’’/6’’’ – H-3	trans-config. H-2 / H-3
4	H-3 – H-4α	H-4α	9	2’’’’/6’’’’ – H-3	α-substitution at 5-Ph
5	2’’’/6’’’ – 2’’’’/6’’’’	α-substitution of 5-Ph			

Supplementary Methods

General Information

All catalytic reactions were performed in a Schlenk tube (10 mL) with magnetic stirring. The

catalysts Λ-**IrS**[10] and Λ-**RhS**[11] were synthesized according to our published procedures. THF, toluene were distilled under nitrogen from sodium/benzophenone. HPLC grade of acetone, ethanol, $CHCl_3$, and CH_2Cl_2 were used without further purification. Reagents including $CDCl_3$ that were purchased from commercial suppliers were used without further purification. Flash column chromatography was performed with silica gel 60 M from Macherey-Nagel (irregular shaped, 230-400 mesh, pH 6.8, pore volume: 0.81 mL × g^{-1}, mean pore size: 66 Å, specific surface: 492 m^2 × g^{-1}, particle size distribution: 0.5% < 25 µm and 1.7% > 71 µm, water content: 1.6%). ^1H NMR, ^{19}F NMR and proton decoupled ^{13}C NMR spectra were recorded on Bruker Avance 300 (300 MHz), Bruker AM (500 MHz) spectrometers at ambient temperature. NMR yields were determined using 1,1,2,2-tetrachloroethane as internal standard. NMR standards were used as follows: ^1H NMR spectroscopy: δ = 7.26 ppm ($CDCl_3$), 5.32 (CD_2Cl_2), ^{13}C NMR spectroscopy: δ = 77.0 ppm ($CDCl_3$), 53.8 (CD_2Cl_2), ^{19}F NMR spectroscopy: δ = 0 ppm ($CFCl_3$). IR spectra were recorded on a Bruker Alpha FT-IR spectrophotometer. High-resolution mass spectra were recorded on a Bruker En Apex Ultra 7.0 TFT-MS instrument using ESI technique. Enantiomeric purities of the reaction products were determined by HPLC chromatography which was performed with an Agilent 1200 or Agilent 1260 HPLC system with a Daicel Chiralpak AS-H, AD-H, OD-H, IC, IG, or Chiralcel OJ-H (250 × 4.6 mm) column as chiral stationary phase using *n*-hexane/isopropanol as mobile phase. Optical rotations were measured on a Krüss P8000-T polarimeter with $[\alpha]_D^{22}$ values reported in degrees with concentrations reported in g/100 mL. UV/Vis absorbance spectra were recorded on a Spectra Max M5 microplate reader in a 10.0 mm quartz cuvette.

Synthesis of Substrates

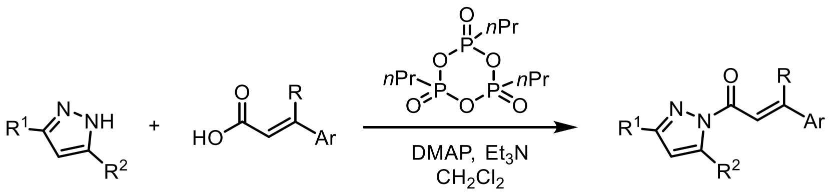

α,β-Unsaturated *N*-acylpyrazoles 1 were synthesized according to published procedures with some modification.[12] To a solution of pyrazole (1.0 equiv) and α,β-unsaturated carboxyl acid (1.5 equiv) in CH_2Cl_2 (0.2 M) at room temperature, was added 1-propanephosphonic acid cyclic anhydride (CAS number: 68957-94-8; 50% solution in EtOAc; 1.5 equiv) dropwise. After stirring for 1 hour at room temperature, the mixture was cooled to 0 ^{o}C followed by the addition of DMAP (0.2 equiv) and Et_3N (3.0 equiv; dropwise). Then the reaction mixture was allowed to warm to room temperature with stirring. After a full conversion of pyrazole was detected by TLC (5-12 h), the mixture was poured into HCl solution (1 M) and extracted with EtOAc for three times. The combined organic layers were washed with NaOH solution (2 M), saturated $NaHCO_3$ solution and brine. After dried with anhydrous Na_2SO_4, filtration and concentration under reduced pressure, the crude residue was purified by flash chromatography on silica gel (*n*-hexane/EtOAc) to afford the substrate **1** with yields from 75% to 95%. To obtain with better quality, the isolated product could be washed with Et_2O/*n*-hexane.

Vinyl azides 2 were prepared according to published procedures.[13,14]

The data of unprecedented substrates **1c-s** and **2i-j** are shown below.

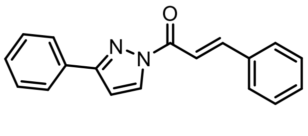

(*E*)-3-Penyl-1-(3-phenyl-1*H*-pyrazol-1-yl)prop-2-en-1-one (1c)

A white solid.

^{1}H NMR (500 MHz, $CDCl_3$) δ 8.43 (d, J = 3.0 Hz, 1H), 8.07 (d, J = 16.0 Hz, 1H), 8.03 (d, J = 16.0 Hz, 1H), 7.97-7.94 (m, 2H), 7.75-7.71 (m, 2H), 7.51-7.40 (m, 6H), 6.84 (d, J = 3.0 Hz, 1H).

^{13}C NMR (75 MHz, $CDCl_3$) δ 163.7, 155.4, 147.6, 134.6, 132.0, 131.0, 130.0, 129.2, 129.0, 128.84, 128.79, 126.4, 116.0, 107.7.

IR (film): ν (cm^{-1}) 1698, 1612, 1532, 1499, 1452, 1400, 1345, 1301, 1213, 1095, 1072, 1036, 995, 954, 915, 874, 755, 681, 564.

HRMS (ESI, *m*/*z*) calcd for $C_{18}H_{14}N_2ONa$ $[M+Na]^+$: 297.1009, found: 297.0999.

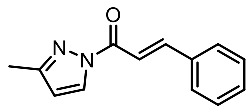

(*E*)-1-(3-Methyl-1*H*-pyrazol-1-yl)-3-phenylprop-2-en-1-one (1d)

A white solid.

^{1}H NMR (300 MHz, $CDCl_3$) δ 8.28 (d, J = 3.0 Hz, 1H), 8.00 (d, J = 15.9 Hz, 1H), 7.87 (d, J = 15.9 Hz, 1H), 7.73-7.67 (m, 2H), 7.46-7.40 (m, 3H), 6.30 (d, J = 2.7 Hz, 1H), 2.38 (s, 3H).

^{13}C NMR (75 MHz, $CDCl_3$) δ 163.4, 153.8, 147.3, 134.6, 130.9, 129.4, 128.9, 128.8, 116.2, 110.6, 14.0.

IR (film): ν (cm^{-1}) 2958, 1685, 1613, 1551, 1446, 1408, 1343, 1203, 1046, 989, 940, 861, 761, 713, 675, 569.

HRMS (ESI, *m*/*z*) calcd for $C_{13}H_{12}N_2ONa$ $[M+Na]^+$: 235.0853, found: 235.0842.

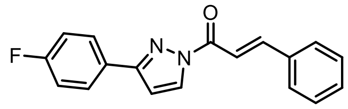

(*E*)-1-(3-(4-Fluorophenyl)-1*H*-pyrazol-1-yl)-3-phenylprop-2-en-1-one (1e)

A white solid.

^{1}H NMR (300 MHz, $CDCl_3$) δ 8.42 (d, J = 3.3 Hz, 1H), 8.06 (d, J = 16.2 Hz, 1H), 7.98 (d, J = 15.9 Hz, 1H), 7.95-7.89 (m, 2H), 7.75-7.70 (m, 2H), 7.48-7.43 (m, 3H), 7.21-7.12 (m, 2H), 6.78 (d, J = 2.7 Hz, 1H).

^{13}C NMR (75 MHz, $CDCl_3$) δ 163.6, 163.4 (d, J = 247.1 Hz), 154.4, 147.7, 134.6, 131.0, 130.1, 129.0, 128.8, 128.204 (d, J = 8.3 Hz), 128.205 (d, J = 2.9 Hz), 115.85, 115.79 (d, J = 21.6 Hz), 107.5.

IR (film): ν (cm^{-1}) 1688, 1612, 1507, 1430, 1398, 1340, 1298, 1219, 1155, 1092, 1052, 980, 949, 885, 840, 815, 758, 679, 624, 567.

HRMS (ESI, *m*/*z*) calcd for $C_{18}H_{13}FN_2ONa$ $[M+Na]^+$: 315.0904, found: 315.0905.

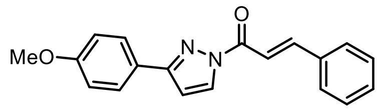

(*E*)-1-(3-(4-Methoxyphenyl)-1*H*-pyrazol-1-yl)-3-phenylprop-2-en-1-one (1f)

A white solid.

^{1}H NMR (300 MHz, $CDCl_3$) δ 8.40 (d, J = 2.7 Hz, 1H), 8.06 (d, J = 15.9 Hz, 1H), 8.00 (d, J = 16.2 Hz, 1H), 7.92-7.85 (m, 2H), 7.76-7.70 (m, 2H), 7.48-7.41 (m, 3H), 7.04-6.96 (m, 2H), 6.77 (d, J = 3.0 Hz, 1H), 3.88 (s, 3H).

^{13}C NMR (75 MHz, $CDCl_3$) δ 163.6, 160.5, 155.2, 147.4, 134.7, 130.9, 129.9, 129.0, 128.8, 127.8, 124.7, 116.2, 114.2, 107.5, 55.4.

IR (film): ν (cm^{-1}) 2964, 1692, 1613, 1510, 1401, 1352, 1301, 1251, 1220, 1174, 1094, 1024, 950, 875, 833, 762, 678, 629, 567.

HRMS (ESI, *m*/*z*) calcd for $C_{19}H_{17}N_2O_2$ $[M+H]^+$: 305.1296, found: 305.1284.

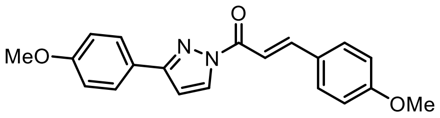

(*E*)-3-(4-Methoxyphenyl)-1-(3-(4-methoxyphenyl)-1*H*-pyrazol-1-yl)prop-2-en-1-one (1g)

A white solid.

^{1}H NMR (300 MHz, $CDCl_3$) δ 8.40 (d, J = 2.7 Hz, 1H), 8.01 (d, J = 15.9 Hz, 1H), 7.91-7.83 (m, 3H), 7.71-7.65 (m, 2H), 7.02-6.93 (m, 4H), 6.75 (d, J = 3.0 Hz, 1H), 3.87 (s, 6H).

^{13}C NMR (75 MHz, $CDCl_3$) δ 163.9, 162.0, 160.4, 155.0, 147.2, 130.7, 129.8, 127.7, 127.5, 124.8, 114.4, 114.2, 113.5, 107.2, 55.4, 55.3.

IR (film): ν (cm^{-1}) 2999, 2932, 1690, 1596, 1568, 1509, 1428, 1395, 1342, 1294, 1251, 1216, 1173, 1092, 1032, 996, 955, 825, 767, 726, 627, 549.

HRMS (ESI, *m*/*z*) calcd for $C_{20}H_{19}N_2O_3$ $[M+H]^+$: 335.1401, found: 335.1390.

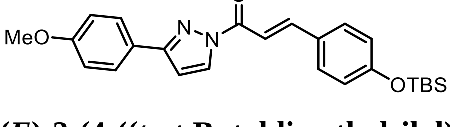

(*E*)-3-(4-((*tert*-Butyldimethylsilyl)oxy)phenyl)-1-(3-(4-methoxyphenyl)-1*H*-pyrazol-1-yl)prop-2-en-1-one (1h)

A white solid.

^{1}H NMR (300 MHz, $CDCl_3$) δ 8.39 (d, J = 2.7 Hz, 1H), 8.00 (d, J = 15.9 Hz, 1H), 7.91-7.82 (m, 3H), 7.66-7.59 (m, 2H), 7.02-6.95 (m, 2H), 6.94-6.86 (m, 2H), 6.74 (d, J = 3.0 Hz, 1H), 3.85 (s, 3H),

1.01 (s, 9H), 0.25 (s, 6H).

^{13}C NMR (75 MHz, CDCl$_3$) δ 163.8, 160.4, 158.5, 154.9, 147.1, 130.5, 129.8, 128.0, 127.7, 124.7, 120.5, 114.1, 113.7, 107.1, 55.2, 25.6, 18.2, -4.4.

IR (film): ν (cm^{-1}) 3144, 2932, 2893, 2857, 1696, 1594, 1508, 1466, 1428, 1400, 1348, 1250, 1220, 1170, 1094, 1035, 990, 950, 909, 831, 775, 733, 690, 630, 567, 527.

HRMS (ESI, *m/z*) calcd for $C_{25}H_{30}N_2O_3SiNa$ [M+Na]$^+$: 457.1918, found: 457.1911.

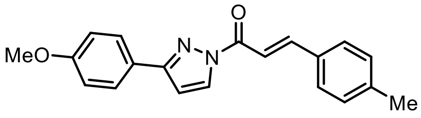

(*E*)-1-(3-(4-Methoxyphenyl)-1*H*-pyrazol-1-yl)-3-(*p*-tolyl)prop-2-en-1-one (1i)

A white solid.

^{1}H NMR (300 MHz, CDCl$_3$) δ 8.40 (d, J = 3.0 Hz, 1H), 8.03 (d, J = 15.9 Hz, 1H), 7.95 (d, J = 15.9 Hz, 1H), 7.93-7.85 (m, 2H), 7.62 (d, J = 8.1 Hz, 2H), 7.25 (d, J = 7.8 Hz, 2H), 7.03-6.96 (m, 2H), 6.76 (d, J = 2.7 Hz, 1H), 3.87 (s, 3H), 2.41 (s, 3H).

^{13}C NMR (75 MHz, CDCl$_3$) δ 163.8, 160.5, 155.1, 147.5, 141.5, 132.0, 129.9, 129.7, 128.9, 127.7, 124.7, 115.0, 114.2, 107.3, 55.3, 21.6.

IR (film): ν (cm^{-1}) 3130, 2967, 1691, 1610, 1508, 1426, 1399, 1349, 1301, 1249, 1219, 1179, 1092, 1030, 951, 885, 838, 807, 767, 728, 688, 628, 526.

HRMS (ESI, *m/z*) calcd for $C_{20}H_{19}N_2O_2$ [M+H]$^+$: 319.1452, found: 319.1442.

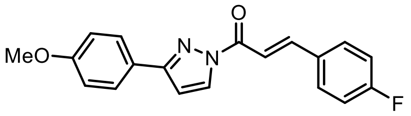

(*E*)-3-(4-Fluorophenyl)-1-(3-(4-methoxyphenyl)-1*H*-pyrazol-1-yl)prop-2-en-1-one (1j)

A white solid.

^{1}H NMR (300 MHz, CDCl$_3$) δ 8.40 (d, J = 2.7 Hz, 1H), 8.00 (d, J = 15.9 Hz, 1H), 7.93 (d, J = 15.9 Hz, 1H), 7.93-7.84 (m, 2H), 7.75-7.69 (m, 2H), 7.17-7.10 (m, 2H), 7.02-6.97 (m, 2H), 6.77 (d, J = 2.7 Hz, 1H), 3.87 (s, 3H).

^{13}C NMR (75 MHz, CDCl$_3$) δ 164.3 (d, J = 251.0 Hz), 163.5, 160.5, 155.3, 146.0, 130.9 (d, J = 3.7 Hz), 130.8 (d, J = 9.1 Hz), 129.9, 127.8, 124.6, 116.2 (d, J = 22.0 Hz), 115.9 (d, J = 2.2 Hz), 114.2, 107.5, 55.3.

IR (film): ν (cm^{-1}) 3085, 2960, 1710, 1592, 1505, 1426, 1394, 1341, 1292, 1240, 1210, 1162, 1091, 1032, 1007, 957, 877, 826, 763, 731, 626.

HRMS (ESI, *m/z*) calcd for $C_{19}H_{15}FN_2O_2Na$ [M+Na]$^+$: 345.1021, found: 345.1010.

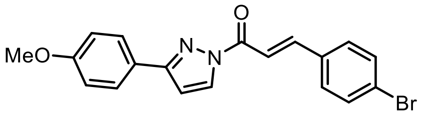

(*E*)-3-(4-Bromophenyl)-1-(3-(4-methoxyphenyl)-1*H*-pyrazol-1-yl)prop-2-en-1-one (1k)

A white solid.

^{1}H NMR (300 MHz, CDCl$_3$) δ 8.39 (d, *J* = 3.0 Hz, 1H), 8.00 (d, *J* = 16.2 Hz, 1H), 7.94 (d, *J* = 15.9 Hz, 1H), 7.90-7.83 (m, 2H), 7.58-7.57 (m, 4H), 7.02-6.97 (m, 2H), 6.77 (d, *J* = 2.7 Hz, 1H), 3.87 (s, 3H).

^{13}C NMR (75 MHz, CDCl$_3$) δ 163.3, 160.6, 155.3, 145.8, 133.5, 132.2, 130.1, 129.9, 127.8, 125.3, 124.5, 116.8, 114.2, 107.6, 55.3.

IR (film): ν (cm^{-1}) 3081, 2963, 1691, 1611, 1511, 1485, 1403, 1345, 1299, 1243, 1218, 1169, 1095, 1036, 984, 944, 821, 767, 626, 528.

HRMS (ESI, *m/z*) calcd for $C_{19}H_{15}BrN_2O_2Na$ [M+Na]$^+$: 405.0209, found: 405.0204.

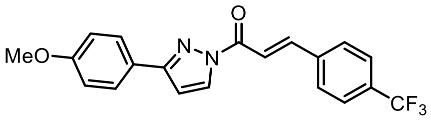

(*E*)-1-(3-(4-Methoxyphenyl)-1*H*-pyrazol-1-yl)-3-(4-(trifluoromethyl)phenyl)prop-2-en-1-one (1l)

A white solid.

^{1}H NMR (300 MHz, CDCl$_3$) δ 8.39 (d, *J* = 3.0 Hz, 1H), 8.08 (d, *J* = 16.2 Hz, 1H), 8.00 (d, *J* = 16.5 Hz, 1H), 7.90-7.84 (m, 2H), 7.81 (d, *J* = 8.1 Hz, 2H), 7.70 (d, *J* = 8.4 Hz, 2H), 7.03-6.96 (m, 2H), 6.78 (d, *J* = 3.0 Hz, 1H), 3.87 (s, 3H).

^{13}C NMR (75 MHz, CDCl$_3$) δ 163.1, 160.6, 155.5, 145.1, 137.9 (q, *J* = 1.3 Hz), 132.2 (q, *J* = 32.8 Hz), 130.0, 128.8, 127.8, 125.9 (q, *J* = 3.7 Hz), 124.4, 123.8 (q, *J* = 270.8 Hz), 118.8, 114.2, 107.8, 55.3.

IR (film): ν (cm^{-1}) 3061, 2936, 1689, 1617, 1577, 1513, 1404, 1349, 1317, 1248, 1222, 1160, 1109, 1046, 983, 945, 878, 828, 791, 763, 739, 625, 594.

HRMS (ESI, *m*/*z*) calcd for $C_{20}H_{15}F_3N_2O_2Na$ [M+Na]$^+$: 395.0978, found: 395.0972.

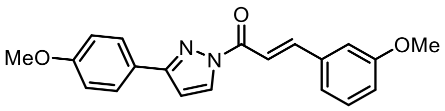

(*E*)-3-(3-Methoxyphenyl)-1-(3-(4-methoxyphenyl)-1*H*-pyrazol-1-yl)prop-2-en-1-one (1m)

A white solid.

^{1}H NMR (300 MHz, $CDCl_3$) δ 8.40 (d, J = 3.0 Hz, 1H), 8.02 (d, J = 16.5 Hz, 1H), 7.97 (d, J = 15.9 Hz, 1H), 7.91-7.84 (m, 2H), 7.40-7.29 (m, 2H), 7.27-7.22 (m, 1H), 7.03-6.96 (m, 3H), 6.77 (d, J = 2.7 Hz, 1H), 3.88 (s, 3H), 3.87 (s, 3H).

^{13}C NMR (75 MHz, $CDCl_3$) δ 163.5, 160.5, 160.0, 155.2, 147.3, 136.0, 129.94, 129.90, 127.7, 124.6, 121.5, 116.7, 116.4, 114.2, 113.8, 107.5, 55.4, 55.3.

IR (film): ν (cm^{-1}) 2967, 2928, 1696, 1613, 1577, 1514, 1399, 1349, 1251, 1216, 1162, 1099, 1034, 988, 949, 830, 765, 726, 676, 628, 576.

HRMS (ESI, *m*/*z*) calcd for $C_{20}H_{19}N_2O_3$ [M+H]$^+$: 335.1390, found: 335.1386.

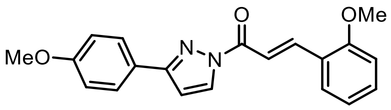

(*E*)-3-(2-Methoxyphenyl)-1-(3-(4-methoxyphenyl)-1*H*-pyrazol-1-yl)prop-2-en-1-one (1n)

A white solid.

^{1}H NMR (300 MHz, $CDCl_3$) δ 8.41 (d, J = 15.9 Hz, 1H), 8.40 (d, J = 2.7 Hz, 1H), 8.05 (d, J = 16.2 Hz, 1H), 7.91-7.85 (m, 2H), 7.77 (dd, J_1 = 7.8 Hz, J_2 = 1.5 Hz, 1H), 7.45-7.37 (m, 1H), 7.07-6.93 (m, 4H), 6.76 (d, J = 3.0 Hz, 1H), 3.94 (s, 3H), 3.87 (s, 3H).

^{13}C NMR (75 MHz, $CDCl_3$) δ 164.0, 160.4, 158.8, 154.9, 142.6, 132.2, 129.9, 129.1, 127.7, 124.8, 123.7, 120.7, 116.2, 114.2, 111.3, 107.1, 55.6, 55.3.

IR (film): ν (cm^{-1}) 2963, 2936, 1692, 1607, 1511, 1461, 1432, 1397, 1344, 1322, 1245, 1214, 1165, 1096, 1018, 990, 949, 834, 805, 750, 627, 583, 516.

HRMS (ESI, *m*/*z*) calcd for $C_{20}H_{19}N_2O_3$ [M+H]$^+$: 335.1390, found: 335.1386.

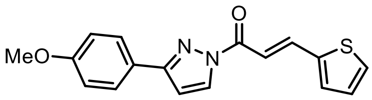

(*E*)-1-(3-(4-Methoxyphenyl)-1*H*-pyrazol-1-yl)-3-(thiophen-2-yl)prop-2-en-1-one (1o)

A white solid.

^{1}H NMR (300 MHz, $CDCl_3$) δ 8.38 (d, J = 3.0 Hz, 1H), 8.14 (d, J = 15.6 Hz, 1H), 7.91-7.84 (m, 2H), 7.78 (d, J = 15.6 Hz, 1H), 7.49 (d, J = 5.1 Hz, 1H), 7.43 (d, J = 3.6 Hz, 1H), 7.12 (dd, J_1 = 5.4 Hz, J_2 = 3.9 Hz, 1H), 7.03-6.97 (m, 2H), 6.76 (d, J = 2.7 Hz, 1H), 3.87 (s, 3H).

^{13}C NMR (75 MHz, $CDCl_3$) δ 163.5, 160.5, 155.2, 140.1, 139.6, 132.2, 129.8, 129.7, 128.3, 127.7, 124.7, 114.8, 114.2, 107.4, 55.3.

IR (film): ν (cm^{-1}) 3077, 2913, 1683, 1597, 1507, 1424, 1396, 1338, 1280, 1239, 1215, 1167, 1089, 1031, 948, 828, 768, 706, 681, 626, 592, 522.

HRMS (ESI, *m*/*z*) calcd for $C_{17}H_{15}N_2O_2S$ [M+H]$^+$: 311.0849, found: 311.0844.

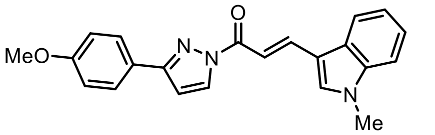

(*E*)-1-(3-(4-Methoxyphenyl)-1*H*-pyrazol-1-yl)-3-(1-methyl-1*H*-indol-3-yl)prop-2-en-1-one (1p)

A yellow solid.

^{1}H NMR (500 MHz, $CDCl_3$) δ 8.43 (d, J = 3.0 Hz, 1H), 8.27 (d, J = 16.0 Hz, 1H), 8.13-8.08 (m, 1H), 7.96-7.89 (m, 3H), 7.54 (s, 1H), 7.42-7.34 (m, 3H), 7.04-7.00 (m, 2H), 6.75 (d, J = 3.0 Hz, 1H), 3.88 (s, 3H), 3.87 (s, 3H).

^{13}C NMR (125 MHz, $CDCl_3$) δ 164.7, 160.3, 154.6, 140.9, 138.2, 134.6, 129.8, 127.7, 126.3, 125.0, 123.3, 121.8, 120.9, 114.2, 113.2, 110.1, 109.9, 106.7, 55.3, 33.4.

IR (film): ν (cm^{-1}) 3098, 2951, 2913, 1677, 1595, 1509, 1463, 1397, 1369, 1344, 1281, 1240, 1217, 1178, 1097, 1067, 1036, 950, 830, 766, 734, 654, 630.

HRMS (ESI, *m*/*z*) calcd for $C_{22}H_{20}N_3O_2$ [M+H]$^+$: 358.1550, found: 358.1545.

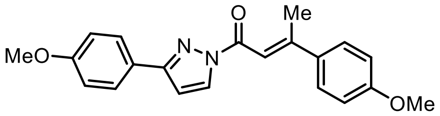

(*E*)-3-(4-Methoxyphenyl)-1-(3-(4-methoxyphenyl)-1*H*-pyrazol-1-yl)but-2-en-1-one ((*E*)-1q)

A white solid.

^{1}H NMR (300 MHz, $CDCl_3$) δ 8.38 (d, J = 2.7 Hz, 1H), 7.89-7.82 (m, 2H), 7.70 (q, J = 1.5 Hz, 1H),

7.67-7.61 (m, 2H), 7.01-6.93 (m, 4H), 6.72 (d, *J* = 2.7 Hz, 1H), 3.86 (s, 3H), 3.85 (s, 3H), 2.74 (d, *J* = 1.2 Hz, 3H).

^{13}C NMR (75 MHz, $CDCl_3$) *δ* 163.3, 161.0, 160.3, 159.3, 154.4, 134.4, 129.6, 128.2, 127.6, 124.9, 114.1, 113.9, 113.0, 106.7, 55.32, 55.26, 18.5.

IR (film): *ν* (cm^{-1}) 3001, 2930, 2838, 1683, 1594, 1567, 1509, 1435, 1396, 1348, 1286, 1246, 1217, 1176, 1093, 1023, 928, 829, 766, 736, 698, 536, 493.

HRMS (ESI, *m*/*z*) calcd for $C_{21}H_{21}N_2O_3$ [M+H]$^+$: 349.1547, found: 349.1542.

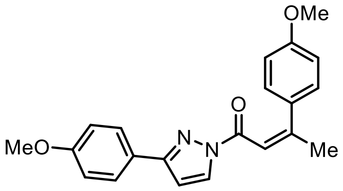

(*Z*)-3-(4-Methoxyphenyl)-1-(3-(4-methoxyphenyl)-1*H*-pyrazol-1-yl)but-2-en-1-one ((*Z*)-1q))

(*Z*)-**1q** was synthesized according to our previous procedure.[17]

A white solid.

^{1}H NMR (500 MHz, $CDCl_3$) *δ* 8.26-8.22 (m, 1H), 7.89-7.83 (m, 2H), 7.36-7.33 (m, 1H), 7.32-7.27 (m, 2H), 7.01-6.95 (m, 2H), 6.94-6.89 (m, 2H), 6.69 (d, *J* = 3.0 Hz, 1H), 3.87 (s, 3H), 3.83 (s, 3H), 2.37 (d, *J* = 1.0 Hz, 3H).

^{13}C NMR (125 MHz, $CDCl_3$) *δ* 162.3, 160.4, 160.3, 159.8, 154.8, 132.6, 129.8, 128.7, 127.7, 124.8, 114.9, 114.1, 113.4, 106.8, 55.3, 55.2, 28.1.

IR (film): *ν* (cm^{-1}) 3121, 2962, 2928, 1704, 1613, 1509, 1436, 1401, 1369, 1333, 1289, 1238, 1173, 1103, 1050, 1023, 943, 885, 829, 814, 789, 730, 686, 639, 555, 521.

HRMS (ESI, *m*/*z*) calcd for $C_{21}H_{21}N_2O_3$ [M+H]$^+$: 349.1547, found: 349.1543.

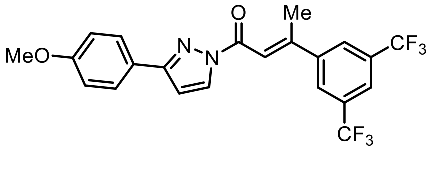

(*E*)-3-(3,5-Bis(trifluoromethyl)phenyl)-1-(3-(4-methoxyphenyl)-1*H*-pyrazol-1-yl)but-2-en-1-one (1r)

A white solid.

^{1}H NMR (500 MHz, $CDCl_3$) *δ* 8.39 (d, *J* = 3.0 Hz, 1H), 8.01 (s, 2H), 7.93 (s, 1H), 7.85-7.80 (m,

2H), 7.69 (q, J = 1.5 Hz, 1H), 7.00-6.95 (m, 2H), 6.77 (d, J = 3.0 Hz, 1H), 3.86 (s, 3H), 2.76 (d, J = 1.5 Hz, 3H).

^{13}C NMR (125 MHz, $CDCl_3$) δ 162.6, 160.5, 155.6, 155.2, 144.6, 132.1 (q, J = 33.8 Hz), 129.8, 127.7, 126.9-126.6 (m), 124.3, 123.1 (q, J = 271.3 Hz), 123.0-122.7 (m), 118.3, 114.2, 107.6, 55.3, 18.9.

^{19}F NMR (282 MHz, $CDCl_3$) δ –62.81 (s, 6F).

IR (film): ν (cm^{-1}) 3054, 2932, 1696, 1613, 1513, 1441, 1403, 1379, 1354, 1283, 1250, 1216, 1168, 1119, 1090, 1032, 938, 883, 836, 774, 679, 619, 580, 523.

HRMS (ESI, *m*/*z*) calcd for $C_{22}H_{16}F_6N_2O_2Na$ $[M+Na]^+$: 477.1008, found: 477.1001.

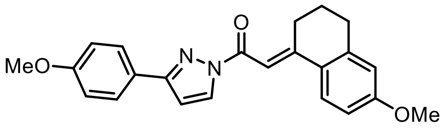

(*E*)-2-(6-Methoxy-3,4-dihydronaphthalen-1(2*H*)-ylidene)-1-(3-(4-methoxyphenyl)-1*H*-pyrazol-1-yl)ethan-1-one (1s)

A white solid.

^{1}H NMR (300 MHz, $CDCl_3$) δ 8.37 (d, J = 2.7 Hz, 1H), 7.91 (d, J = 9.0 Hz, 1H), 7.89-7.82 (m, 3H), 7.02-6.95 (m, 2H), 6.72 (dd, J_1 = 9.0 Hz, J_2 = 2.4 Hz, 1H), 6.73-6.87 (m, 2H), 3.863 (s, 3H), 3.856 (s, 3H), 3.36 (td, J_1 = 7.8 Hz, J_2 = 1.8 Hz, 2H), 2.82 (d, J = 6.0 Hz, 2H), 1.95-1.85 (m, 2H).

^{13}C NMR (75 MHz, $CDCl_3$) δ 163.4, 161.4, 160.3, 159.4, 154.2, 143.1, 129.5, 127.6, 127.4, 127.2, 125.0, 114.1, 113.3, 113.1, 108.0, 106.5, 55.3(two MeO), 20.7, 29.3, 22.6.

IR (film): ν (cm^{-1}) 2927, 2837, 1683, 1579, 1504, 1434, 1397, 1331, 1299, 1221, 1174, 1097, 1036, 955, 889, 864, 828, 767, 716, 629, 594, 527, 413.

HRMS (ESI, *m*/*z*) calcd for $C_{23}H_{23}N_2O_3$ $[M+H]^+$: 375.1703, found: 375.1699.

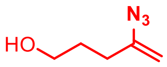

4-Azidopent-4-en-1-ol (2i)

A yellow liquid.

^{1}H NMR (300 MHz, $CDCl_3$) δ 4.72-4.69 (m, 1H), 4.68-4.65 (m, 1H), 3.68 (t, J = 6.2 Hz, 2H), 2.22-2.15 (m, 2H), 1.81-1.69 (m, 2H).

^{13}C NMR (75 MHz, $CDCl_3$) δ 146.3, 98.3, 61.7, 30.2, 30.1.

IR (film): ν (cm^{-1}) 3318, 2931, 2875, 2092, 1627, 1441, 1265, 1048, 916, 843, 657, 541, 456.

HRMS (ESI, *m*/*z*) calcd for $C_5H_{10}N_3O$ $[M+H]^+$: 128.0818, *not found probably due to the decomposition.*

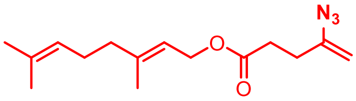

(*E*)-3,7-Dimethylocta-2,6-dien-1-yl 4-azidopent-4-enoate (2j)

A yellow liquid.

^{1}H NMR (300 MHz, CD_2Cl_2) δ 5.35-5.28 (m, 1H), 5.14-5.05 (m, 1H), 4.76-4.72 (m, 1H), 4.68-4.65 (m, 1H), 4.59 (d, *J* = 7.2 Hz, 2H), 2.52-2.44 (m, 2H), 2.41-2.33 (m, 2H), 2.16-2.00 (m, 4H), 1.70 (s, 3H), 1.69-1.66 (m, 3H), 1.61 (s, 3H).

^{13}C NMR (75 MHz, CD_2Cl_2) δ 172.4, 146.0, 142.7, 132.1, 124.1, 118.8, 98.7, 61.8, 39.9, 32.5, 29.4, 26.7, 25.7, 17.7, 16.5.

IR (film): ν (cm^{-1}) 2968, 2920, 2857, 2102, 1733, 1629, 1441, 1379, 1277, 1169, 1048, 955, 846, 634.

HRMS (ESI, *m*/*z*) calcd for $C_{15}H_{23}N_3O_2Na$ $[M+Na]^+$: 300.1682, found: 300.1679.

Typical Procedure

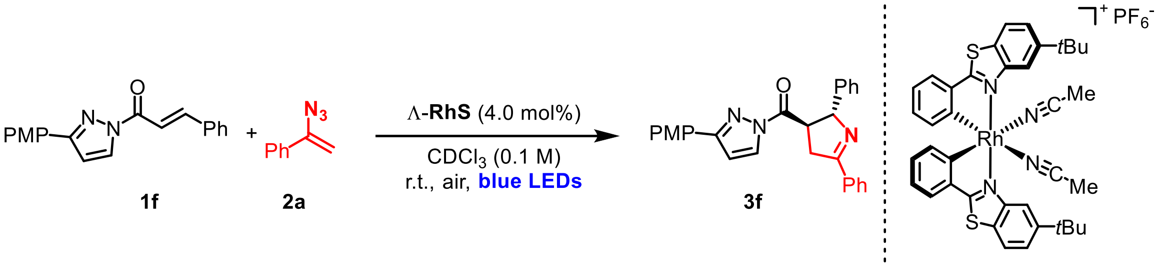

An oven-dried 10 mL Schlenk tube was charged with α,β-unsaturated *N*-acylpyrazole **1f** (30.4 mg, 0.10 mmol) and Λ-**RhS** (3.5 mg, 4 mol%) under air. Then, $CDCl_3$ (1.0 mL, 0.1 M) was added via syringe, followed by vinyl azide **2a** (18.2 mg, 1.25 equiv) under open air atmosphere with stirring. The tube was sealed and positioned at approximately 8 cm from a 24 W blue LEDs lamp. After stirring for the indicated time (monitored by TLC), the mixture was directly subjected to ^{1}H NMR to determine the d.r. value. Then, all the mixture was collected and purified by flash

chromatography on silica gel (*n*-hexane/EtOAc) to afford the product **3f**. The enantiomeric excess was determined by HPLC analysis on a chiral stationary phase. Racemic samples were obtained by carrying out the reactions with *rac*-**RhS**.

For products **3y-3ab** which might decompose in silica gel column, a fast flash chromatography is recommended.

Mechanistic Studies

Identification of Substrate-Bound Rhodium Intermediate

Following our established procedure,[15] **RhS-1f** was synthesized in quantitative yield. Single crystals of **RhS-1f** suitable for X-ray diffraction were obtained by slow diffusion of a solution of **RhS-1f** (20 mg) in CH_2Cl_2 (0.5 mL) layered with Et_2O (0.5 mL) at room temperature for several days in a NMR tube. Crystal structure, data and details of the structure determination for **RhS-1f** are presented in the Supplementary Fig. 3 and Supplementary Table 5.

UV/Vis Absorption Spectra

The absorption spectra of **RhS**, **RhS-1f**, **1f** and **2a** are shown in Fig. 3b. **1f** has a weak absorption in visible light region, thus explaining the background racemic reaction without catalyst (Table 1, entry 14). **2a** does not absorb visible light but could also be activated through energy transfer which would lead to the generation of 2*H*-azirine **4** in consistent with literature report.[16] In the contrast, strong absorption of **RhS-1f** appears at near UV and visible region. These results support that the in-situ generated substrate bound catalyst[17] serves as the visible light harvesting antenna in the present catalysis.

Control Experiment with 2*H*-Azirine

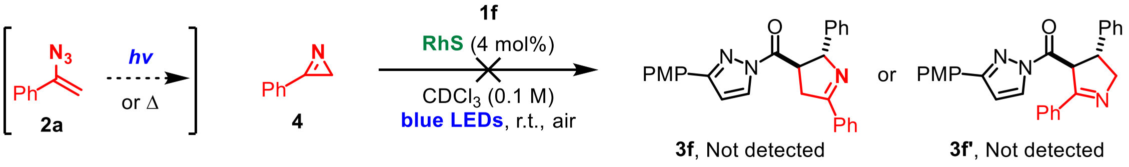

Padwa reported in pioneering work[18,19] that the UV-photolysis of aryl azirines, accessible from vinyl azides by photolysis or heat, could form nitrile ylides which could be subsequently trapped by ground state electron-deficient alkenes to afford racemic 1-pyrrolines (Fig. 1b). To evaluate the

possibility of 2*H*-azirine acting as intermediate, **4** was synthesized[20] and subjected to the standard reaction conditions. As shown, under the irradiation of visible light, ***NO*** new product formed in the reaction of **4** with **1f**. This result rules out the possibility that such azirines are viable intermediates in this catalytic cycle.

Reactions Employing Diastereomeric Substrates

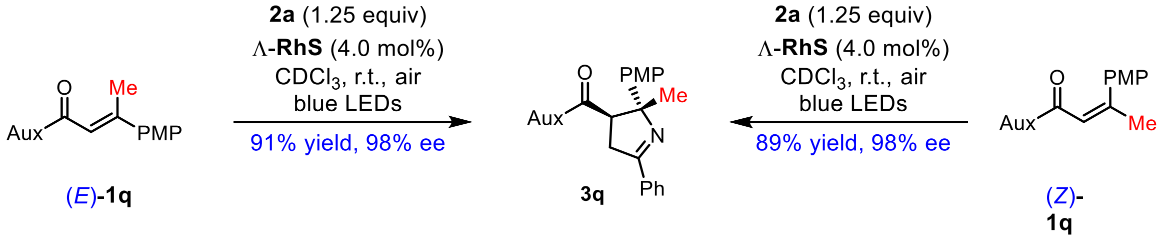

To support the involvement of diradical intermediates **II/III** (Fig. 2), (*E*)-**1q** and (*Z*)-**1q** were synthesized and subjected to the standard conditions, respectively. According to the typical procedure, the reactions of (*E*)-**1q** and (*Z*)-**1q**, respectively, afforded **3q** with identical stereochemistry (judged by ^{1}H NMR and HPLC). These results indicate the formation of diradical intermediates and are in consistent with DFT calculation which illustrates that a large amount of the spin of the excited rhodium / substrate complex is located at the alkene carbons leading to configurational lability of the alkene. Furthermore, these results demonstrate the practicality of the protocol since the preparation of starting materials could be easier without the concern of *E*/*Z* isomers.

Quantum Yield Measurement

The quantum yield was measured according to published procedures with slight modifications.[17,21] A 150 W xenon lamp (50% of light intensity, 420 ± 5 nm bandpass filter) was used as the light source. All the light sensitive operations were processed in the dark room with a 1.1 W red LED. And the photon flux was determined as 1.221×10^{-9} mol s^{-1} according to our previously reported method.[17]

Determination of Response Factor for GC Analysis

n-Dodecane was chosen as the internal standard, the amount of which remained constant for every GC measurement (FID detector, column: HP-5). The amount of product **3f** is related to the ratio of integrated areas. The relation formula was yielded as:

Equation 1: moles of **3f** = $6.229 \times 10^{-5} \times A_{\mathbf{3f}}/A_{n\text{-dodecane}}$ (mol)

Determination of Quantum Yield for Model Reaction

The model reaction **1f** + **2a** → **3f** was chosen to determine the quantum yield under open air conditions. The Newport instrument for quantum yield determination was set up at a fixed position in a dark room.

A screw-top cuvette (10.0 mm) with a small magnetic stir bar was charged with **1f** (30.4 mg, 0.10 mmol), **2a** (18.2 mg, 1.25 equiv), *rac*-**RhS** (3.5 mg, 4 mol%) and $CDCl_3$ (1.0 mL, 0.1 M) under air. Then, *n*-dodecane (10 µL) was added. The cuvette was sealed and fixed at the same position as the measurement of photon flux. The reaction mixture was stirred and irradiated for 4 h. After irradiation, the reaction mixture was analysed by GC.

Experiment 1: the amount of **3f** formed was determined as 3.233×10^{-6} mol. The quantum yield was calculated as 0.184 according to the Supplementary equation 2.

Equation 2:

$$\text{Quantum Yield} = \frac{moles\ of\ \mathbf{3f}}{moles\ of\ photons\ absorbed} = \frac{moles\ of\ \mathbf{3f}}{photon\ flux \times t \times f}$$

Where *f* is the fraction of light absorbed. As the absorbance of the reaction mixture at 420 nm is > 3, the fraction of light absorbed is > 0.999 ($f = 1 - 10^{-A}$).

Experiment 2: the amount of **3f** formed was determined as 3.534×10^{-6} mol. Accordingly, the quantum yield was calculated as 0.201.

Therefore, **the average quantum yield was determined as 0.19**

Computational Studies

Computational Methodology

All calculations were performed using Gaussian 09[22] and the M06[23] functional with Grimme's D3 empirical dispersion correction.[24] Geometries were optimized without constraints for the singlet and triplet states with the LANL2DZ[25] basis set for rhodium and the 6-31G(d) basis set for all other atoms. Frequency analysis was performed to ensure all geometries were minimal. Single-point energies were calculated using the LANL2DZ basis set for rhodium and the 6-311++G(d,p) basis set for all other atoms at the optimized gas-phase geometries for spin densities and triplet state

energies, while using the CPCM[26,27] approach as implemented in Gaussian 09, employing chloroform as a solvent for ligand exchange. The performance of this methodology was validated for the system under study by comparison (see Supplementary Table 7) of the UV/Vis absorption spectra of **1f**, **RhS**, and **RhS-1f** calculated by TD-DFT using the same method and basis sets to the experimental absorption spectra shown in Fig. 3b**.**

As shown in Supplementary Fig. 5, computational studies reveal that the release of ligand in **IrS** is kinetically unfavourable than that in **RhS**, which is consistent with our previous experimental results[28,29] and also supports our observation of low reactivity of **IrS** in current transformation.

For the detailed spin densities and coordinates of intermediates, see Supplementary Data.

Experimental and Characterization Data of Products

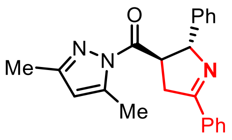

(3,5-Dimethyl-1*H*-pyrazol-1-yl)((2*R*,3*R*)-2,5-diphenyl-3,4-dihydro-2*H*-pyrrol-3-yl)methanone (3a)

As shown in Table 1 entry 1, the reaction of (*E*)-1-(3,5-dimethyl-1*H*-pyrazol-1-yl)-3-phenylprop-2-en-1-one **1a** (22.6 mg, 0.10 mmol), (1-azidovinyl)benzene **2a** (18.2 mg, 1.25 equiv) and Λ-**RhS** (6.9 mg, 8 mol%) in acetone (0.5 mL, 0.2 M) under nitrogen atmosphere (degassed with freeze-pump-thaw) with blue LEDs for 18 hours, afforded **3a** as a white solid (54% NMR yield). Only one single diastereoisomer was observed through ^{1}H NMR of crude materials. Enantiomeric excess of **3a** was established by HPLC analysis using a Chiralpak AS-H column, ee = 86% (HPLC: AS-H, 254 nm, *n*-hexane/isopropanol = 90:10, flow rate 1 mL/min, 40 °C, t_r (major) = 5.2 min, t_r (minor) = 8.6 min). $[\alpha]_D^{22}$ = –37.6° (*c* 0.5, CH_2Cl_2).

^{1}H NMR (500 MHz, $CDCl_3$) δ 7.99-7.95 (m, 2H), 7.50-7.42 (m, 3H), 7.37-7.30 (m, 4H), 7.28-7.23 (m, 1H), 5.99-5.97 (m, 1H), 5.81-5.76 (m, 1H), 4.43 (ddd, J_1 = 9.5 Hz, J_2 = 6.5 Hz, J_3 = 6.0 Hz, 1H), 3.66 (ddd, J_1 = 17.5 Hz, J_2 = 10.0 Hz, J_3 = 2.0 Hz, 1H), 3.40 (ddd, J_1 = 17.0 Hz, J_2 = 7.0 Hz, J_3 = 1.5 Hz, 1H), 2.57 (d, J = 1.0 Hz, 3H), 2.19 (s, 3H).

^{13}C NMR (125 MHz, $CDCl_3$) δ 173.9, 171.5, 152.2, 144.3, 142.7, 133.7, 130.9, 128.5, 128.4, 128.0, 127.2, 127.0, 111.4, 78.3, 51.1, 40.5, 14.5, 13.8.

IR (film): ν (cm^{-1}) 3028, 2925, 1713, 1621, 1581, 1489, 1437, 1408, 1375, 1337, 1316, 1250, 1168, 1139, 1010, 958, 917, 864, 801, 759, 695, 613.

HRMS (ESI, *m*/*z*) calcd for $C_{22}H_{22}N_3O$ $[M+H]^+$: 344.1757, found: 344.1757.

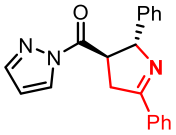

((2*R*,3*R*)-2,5-Diphenyl-3,4-dihydro-2*H*-pyrrol-3-yl)(1*H*-pyrazol-1-yl)methanone (3b)

As shown in Table 1 entry 2, the reaction of 3-phenyl-1-(1*H*-pyrazol-1-yl)prop-2-en-1-one **1b** (19.8 mg, 0.10 mmol), (1-azidovinyl)benzene **2a** (18.2 mg, 1.25 equiv) and Λ-**RhS** (6.9 mg, 8 mol%) in acetone (0.5 mL, 0.2 M) under nitrogen atmosphere (degassed with freeze-pump-thaw) with blue LEDs for 24 hours, afforded **3b** as a white solid (75% NMR yield).

Only one single diastereoisomer was observed through ^1H NMR of crude materials. Enantiomeric excess of **3b** was established by HPLC analysis using a Chiralpak AS-H column, ee = 72% (HPLC: AS-H, 254 nm, *n*-hexane/isopropanol = 80:20, flow rate 1 mL/min, 40 °C, t_r (major) = 16.2 min, t_r (minor) = 7.5 min). $[\alpha]_D^{22}$ = –32.4° (*c* 1.0, CH_2Cl_2).

^1H NMR (300 MHz, $CDCl_3$) δ 8.31 (d, J = 2.7 Hz, 1H), 7.99-7.94 (m, 2H), 7.70 (d, J = 0.9 Hz, 1H), 7.52-7.41 (m, 3H), 7.37-7.24 (m, 5H), 6.48 (dd, J_1 = 2.7 Hz, J_2 = 1.2 Hz, 1H), 5.83 (dt, J_1 = 6.3 Hz, J_2 = 1.5 Hz, 1H), 4.46 (ddd, J_1 = 9.9 Hz, J_2 = 7.2 Hz, J_3 = 6.6 Hz, 1H), 3.71 (ddd, J_1 = 17.1 Hz, J_2 = 9.6 Hz, J_3 = 1.8 Hz, 1H), 3.43 (ddd, J_1 = 17.1 Hz, J_2 = 7.2 Hz, J_3 = 1.8 Hz, 1H).

^{13}C NMR (125 MHz, $CDCl_3$) δ 172.5, 171.2, 144.2, 142.4, 133.6, 131.0, 128.7, 128.6, 128.0, 127.4, 126.8, 110.0, 78.6, 50.0, 40.7. (Missing one ^{13}C signal)

IR (film): ν (cm^{-1}) 3124, 3091, 2924, 1719, 1613, 1573, 1535, 1491, 1448, 1419, 1381, 1312, 1270, 1247, 1198, 1101, 1028, 946, 915, 863, 832, 802, 751, 689, 641, 595.

HRMS (ESI, *m*/*z*) calcd for $C_{20}H_{18}N_3O$ $[M+H]^+$: 316.1455, found: 316.1445.

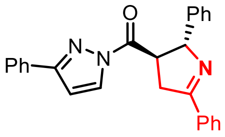

((2*R*,3*R*)-2,5-Diphenyl-3,4-dihydro-2*H*-pyrrol-3-yl)(3-phenyl-1*H*-pyrazol-1-yl)methanone (3c)

As shown in Table 1 entry 7, the reaction of (*E*)-3-phenyl-1-(3-phenyl-1*H*-pyrazol-1-yl)

prop-2-en-1-one **1c** (27.4 mg, 0.10 mmol), (1-azidovinyl)benzene **2a** (18.2 mg, 1.25 equiv) and Λ-**RhS** (3.5 mg, 4 mol%) in $CDCl_3$ (0.5 mL, 0.2 M) under nitrogen atmosphere (degassed with freeze-pump-thaw) with blue LEDs for 18 hours, afforded **3c** as a white solid (80% NMR yield). Only one single diastereoisomer was observed through ^{1}H NMR of crude materials. Enantiomeric excess of **3c** was established by HPLC analysis using a Chiralpak OD-H column, ee = 92% (HPLC: OD-H, 254 nm, *n*-hexane/isopropanol = 90:10, flow rate 1 mL/min, 25 °C, t_r (major) = 11.6 min, t_r (minor) = 15.4 min). $[\alpha]_D^{22}$ = +36.4° (*c* 1.0, CH_2Cl_2).

^{1}H NMR (500 MHz, $CDCl_3$) δ 8.36 (d, J = 3.0 Hz, 1H), 8.04-7.99 (m, 2H), 7.79-7.75 (m, 2H), 7.53-7.35 (m, 10H), 7.34-7.29 (m, 1H), 6.83 (d, J = 2.5 Hz, 1H), 5.86-5.83 (m, 1H), 4.59 (ddd, J_1 = 9.5 Hz, J_2 = 6.5 Hz, J_3 = 6.0 Hz, 1H), 3.71 (ddd, J_1 = 17.0 Hz, J_2 = 10.5 Hz, J_3 = 2.0 Hz, 1H), 3.59 (ddd, J_1 = 17.0 Hz, J_2 = 6.5 Hz, J_3 = 1.5 Hz, 1H).

^{13}C NMR (125 MHz, $CDCl_3$) δ 172.3, 171.4, 155.5, 142.5, 133.6, 131.4, 130.9, 129.8, 129.2, 128.6, 128.5, 128.0, 127.3, 126.9, 126.3, 107.7, 79.2, 49.8, 40.0. (Missing one ^{13}C signal)

IR (film): ν (cm^{-1}) 3059, 3031, 1719, 1619, 1540, 1450, 1407, 1344, 1230, 1077, 1036, 944, 908, 799, 756, 688, 613, 555.

HRMS (ESI, *m*/*z*) calcd for $C_{26}H_{22}N_3O$ $[M+H]^+$: 392.1757, found: 392.1754.

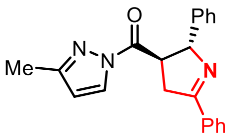

((2*R*,3*R*)-2,5-Diphenyl-3,4-dihydro-2*H*-pyrrol-3-yl)(3-methyl-1*H*-pyrazol-1-yl)methanone (3d)

As shown in Table 1 entry 8, the reaction of (*E*)-1-(3-methyl-1*H*-pyrazol-1-yl)-3-phenylprop-2-en-1-one **1d** (21.2 mg, 0.10 mmol), (1-azidovinyl)benzene **2a** (18.2 mg, 1.25 equiv) and Λ-**RhS** (3.5 mg, 4 mol%) in $CDCl_3$ (1.0 mL, 0.1 M) under nitrogen atmosphere (degassed with freeze-pump-thaw) with blue LEDs for 24 hours, afforded **3d** as a white solid (82% NMR yield). Only one single diastereoisomer was observed through ^{1}H NMR of crude materials. Enantiomeric excess of **3d** was established by HPLC analysis using a Chiralpak AS-H column, ee = 92% (HPLC: AS-H, 254 nm, *n*-hexane/isopropanol = 90:10, flow rate 1 mL/min, 40 °C, t_r (major) = 13.2 min, t_r (minor) = 9.3 min). $[\alpha]_D^{22}$ = –44.2° (*c* 1.0, CH_2Cl_2).

^{1}H NMR (500 MHz, $CDCl_3$) δ 8.18 (d, J = 3.0 Hz, 1H), 7.98-7.94 (m, 2H), 7.51-7.41 (m, 3H),

7.36-7.30 (m, 4H), 7.29-7.24 (m, 1H), 6.28-6.26 (m, 1H), 5.81 (dt, J_1 = 6.0 Hz, J_2 = 2.0 Hz, 1H), 4.39 (ddd, J_1 = 10.0 Hz, J_2 = 7.5 Hz, J_3 = 6.5 Hz, 1H), 3.70 (ddd, J_1 = 16.5 Hz, J_2 = 9.5 Hz, J_3 = 1.5 Hz, 1H), 3.41 (ddd, J_1 = 17.0 Hz, J_2 = 7.0 Hz, J_3 = 1.5 Hz, 1H), 2.28 (s, 3H).

^{13}C NMR (125 MHz, $CDCl_3$) δ 172.0, 171.2, 154.1, 142.5, 133.6, 130.9, 129.3, 128.52, 128.46, 128.0, 127.3, 126.9, 110.8, 78.5, 50.0, 40.7, 13.9.

IR (film): ν (cm^{-1}) 3134, 2923, 1720, 1616, 1551, 1442, 1407, 1365, 1334, 1287, 1248, 1201, 1054, 1016, 951, 899, 862, 768, 691, 609, 550.

HRMS (ESI, *m*/*z*) calcd for $C_{21}H_{20}N_3O$ [M+H]$^+$: 330.1601, found: 330.1597.

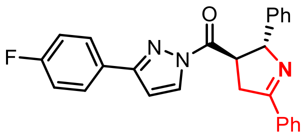

((2*R*,3*R*)-2,5-Diphenyl-3,4-dihydro-2*H*-pyrrol-3-yl)(3-(4-fluorophenyl)-1*H*-pyrazol-1-yl) methanone (3e)

As shown in Table 1 entry 9, the reaction of (*E*)-1-(3-(4-fluorophenyl)-1*H*-pyrazol-1-yl)-3-phenylprop-2-en-1-one **1e** (29.2 mg, 0.10 mmol), (1-azidovinyl)benzene **2a** (18.2 mg, 1.25 equiv) and Λ-**RhS** (3.5 mg, 4 mol%) in $CDCl_3$ (1.0 mL, 0.1 M) under nitrogen atmosphere (degassed with freeze-pump-thaw) with blue LEDs for 24 hours, afforded **3e** as a white solid (80% NMR yield). Only one single diastereoisomer was observed through ^{1}H NMR of crude materials. Enantiomeric excess of **3e** was established by HPLC analysis using a Chiralcel OJ-H column, ee = 92% (HPLC: OJ-H, 254 nm, *n*-hexane/isopropanol = 60:40, flow rate 1 mL/min, 40 °C, t_r (major) = 17.4 min, t_r (minor) = 11.6 min). $[\alpha]_D^{22}$ = +36.6° (*c* 1.0, CH_2Cl_2).

^{1}H NMR (500 MHz, $CDCl_3$) δ 8.33 (d, J = 3.0 Hz, 1H), 8.01-7.96 (m, 2H), 7.72-7.66 (m, 2H), 7.54-7.43 (m, 3H), 7.37-7.27 (m, 5H), 7.12-7.05 (m, 2H), 6.77 (d, J = 3.0 Hz, 1H), 5.78 (dt, J_1 = 6.0 Hz, J_2 = 1.5 Hz, 1H), 4.55 (ddd, J_1 = 10.0 Hz, J_2 = 7.0 Hz, J_3 = 6.0 Hz, 1H), 3.68 (ddd, J_1 = 17.5 Hz, J_2 = 10.0 Hz, J_3 = 2.0 Hz, 1H), 3.59 (ddd, J_1 = 17.0 Hz, J_2 = 6.5 Hz, J_3 = 1.5 Hz, 1H).

^{13}C NMR (125 MHz, $CDCl_3$) δ 172.3, 171.4, 163.4 (d, J = 247.4 Hz), 154.6, 142.5, 133.6, 131.0, 130.0, 128.6, 128.5, 128.1 (d, J = 8.4 Hz), 128.0, 127.7 (d, J = 3.1 Hz), 127.4, 127.0, 115.7 (d, J = 21.5 Hz), 107.6, 79.4, 49.9, 40.1.

IR (film): ν (cm^{-1}) 3029, 2887, 1714, 1607, 1511, 1432, 1399, 1333, 1282, 1224, 1156, 1092, 1044,

936, 909, 837, 759, 691, 616, 555.

HRMS (ESI, *m*/*z*) calcd for $C_{26}H_{21}FN_3O$ [M+H]$^+$: 410.1663, found: 410.1658.

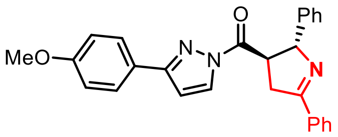

((2*R*,3*R*)-2,5-Diphenyl-3,4-dihydro-2*H*-pyrrol-3-yl)(3-(4-methoxyphenyl)-1*H*-pyrazol-1-yl) methanone (3f)

As shown in Table 1 entry 10, the reaction of (*E*)-1-(3-(4-methoxyphenyl)-1*H*-pyrazol-1-yl)-3-phenylprop-2-en-1-one **1f** (30.4 mg, 0.10 mmol), (1-azidovinyl)benzene **2a** (18.2 mg, 1.25 equiv) and Λ-**RhS** (3.5 mg, 4 mol%) in $CDCl_3$ (1.0 mL, 0.1 M) under nitrogen atmosphere (degassed with freeze-pump-thaw) with blue LEDs for 24 hours, afforded **3f** as a white solid (92% NMR yield, 90% isolated yield).

Only one single diastereoisomer was observed through ^{1}H NMR of crude materials. Enantiomeric excess of **3f** was established by HPLC analysis using a Chiralpak OD-H column, ee = 94% (HPLC: OD-H, 254 nm, *n*-hexane/isopropanol = 95:5, flow rate 1 mL/min, 40 °C, t_r (major) = 20.3 min, t_r (minor) = 24.0 min). $[\alpha]_D^{22}$ = +14.4° (*c* 1.0, CH_2Cl_2).

^{1}H NMR (500 MHz, $CDCl_3$) δ 8.31 (d, *J* = 3.0 Hz, 1H), 8.02-7.97 (m, 2H), 7.71-7.64 (m, 2H), 7.53-7.43 (m, 3H), 7.39-7.32 (m, 4H), 7.31-7.27 (m, 1H), 6.96-6.91 (m, 2H), 6.75 (d, *J* = 3.0 Hz, 1H), 5.82-5.78 (m, 1H), 4.55 (ddd, J_1 = 9.5 Hz, J_2 = 6.5 Hz, J_3 = 6.0 Hz, 1H), 3.86 (s, 3H), 3.69 (ddd, J_1 = 17.0 Hz, J_2 = 9.5 Hz, J_3 = 2.0 Hz, 1H), 3.57 (ddd, J_1 = 17.0 Hz, J_2 = 6.5 Hz, J_3 = 1.5 Hz, 1H).

^{13}C NMR (125 MHz, $CDCl_3$) δ 172.3, 171.5, 160.5, 155.3, 142.5, 133.6, 131.0, 129.8, 128.54, 128.52, 128.0, 127.7, 127.4, 127.0, 124.2, 114.1, 107.5, 79.2, 55.3, 49.9, 40.1.

IR (film): *v* (cm^{-1}) 2921, 2854, 1714, 1608, 1512, 1440, 1397, 1345, 1284, 1247, 1228, 1175, 1089, 1020, 937, 901, 836, 809, 770, 726, 691, 614, 558.

HRMS (ESI, *m*/*z*) calcd for $C_{27}H_{24}N_3O_2$ [M+H]$^+$: 422.1863, found: 422.1857.

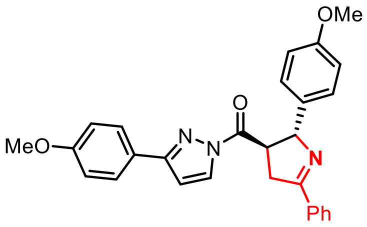

(3-(4-Methoxyphenyl)-1*H*-pyrazol-1-yl)((2*R*,3*R*)-2-(4-methoxyphenyl)-5-phenyl-3,4-dihydro-2*H*-pyrrol-3-yl)methanone (3g)

According to the typical procedure, the reaction of (*E*)-3-(4-methoxyphenyl)-1-(3-(4-methoxyphenyl)-1*H*-pyrazol-1-yl)prop-2-en-1-one **1g** (33.4 mg, 0.10 mmol), (1-azidovinyl)benzene **2a** (18.2 mg, 1.25 equiv) and Λ-**RhS** (3.5 mg, 4 mol%) in $CDCl_3$ (1.0 mL, 0.1 M) under open air atmosphere with blue LEDs for 24 hours, afforded **3g** as a white solid (40.2 mg, 89% isolated yield).

Only one single diastereoisomer was observed through ^{1}H NMR of crude materials. Enantiomeric excess of **3g** was established by HPLC analysis using a Chiralpak OD-H column, ee = 99% (HPLC: OD-H, 254 nm, *n*-hexane/isopropanol = 95:5, flow rate 1 mL/min, 40 °C, t_r (major) = 29.4 min, t_r (minor) = 36.1 min). $[\alpha]_D^{22}$ = +51.6° (*c* 1.0, CH_2Cl_2).

^{1}H NMR (300 MHz, $CDCl_3$) δ 8.30 (d, J = 2.7 Hz, 1H), 8.01-7.95 (m, 2H), 7.72-7.65 (m, 2H), 7.52-7.41 (m, 3H), 7.32-7.26 (m, 2H), 6.97-6.84 (m, 4H), 6.75 (d, J = 2.7 Hz, 1H), 5.75-5.71 (m, 1H), 4.57-4.47 (m, 1H), 3.85 (s, 3H), 3.79 (s, 3H), 3.66 (ddd, J_1 = 17.1 Hz, J_2 = 9.6 Hz, J_3 = 2.1 Hz, 1H), 3.56 (ddd, J_1 = 17.1 Hz, J_2 = 7.2 Hz, J_3 = 1.5 Hz, 1H).

^{13}C NMR (75 MHz, $CDCl_3$) δ 172.4, 171.2, 160.5, 159.0, 155.3, 134.8, 133.7, 130.9, 129.8, 128.5, 128.1, 128.0, 127.7, 124.3, 114.1, 113.9, 107.5, 78.9, 55.32, 55.26, 50.0, 40.0.

IR (film): ν (cm^{-1}) 3057, 2962, 2911, 1713, 1610, 1511, 1435, 1405, 1352, 1334, 1300, 1246, 1173, 1029, 938, 905, 826, 771, 733, 692, 564.

HRMS (ESI, *m*/*z*) calcd for $C_{28}H_{26}N_3O_3$ $[M+H]^+$: 452.1969, found: 452.1963.

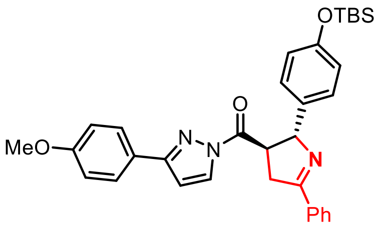

((2*R*,3*R*)-2-(4-((*tert*-Butyldimethylsilyl)oxy)phenyl)-5-phenyl-3,4-dihydro-2*H*-pyrrol-3-yl)(3-(4-

methoxyphenyl)-1*H*-pyrazol-1-yl)methanone (3h)

According to the typical procedure, the reaction of (*E*)-3-(4-((*tert*-butyldimethylsilyl)oxy)phenyl)-1-(3-(4-methoxyphenyl)-1*H*-pyrazol-1-yl)prop-2-en-1-one **1h** (43.4 mg, 0.10 mmol), (1-azidovinyl)benzene **2a** (18.2 mg, 1.25 equiv) and Λ-**RhS** (3.5 mg, 4 mol%) in $CDCl_3$ (1.0 mL, 0.1 M) under open air atmosphere with blue LEDs for 24 hours, afforded **3h** as a yellow oil (39.0 mg, 71% isolated yield).

Only one single diastereoisomer was observed through ^{1}H NMR of crude materials. Enantiomeric excess of **3h** was established by HPLC analysis using a Chiralpak OD-H column, ee = 99% (HPLC: OD-H, 254 nm, *n*-hexane/isopropanol = 80:20, flow rate 1 mL/min, 40 °C, t_r (major) = 5.7 min, t_r (minor) = 6.5 min). $[\alpha]_D^{22}$ = +27.6° (*c* 1.0, CH_2Cl_2).

^{1}H NMR (300 MHz, $CDCl_3$) δ 8.30 (d, J = 3.0 Hz, 1H), 8.01-7.96 (m, 2H), 7.74-7.67 (m, 2H), 7.52-7.41 (m, 3H), 7.25-7.18 (m, 2H), 6.98-6.91 (m, 2H), 6.84-6.78 (m, 2H), 6.75 (d, J = 2.7 Hz, 1H), 5.76-5.70 (m, 1H), 4.57-4.47 (m, 1H), 3.86 (s, 3H), 3.67 (ddd, J_1 = 17.1 Hz, J_2 = 9.3 Hz, J_3 = 1.8 Hz, 1H), 3.55 (ddd, J_1 = 16.8 Hz, J_2 = 6.6 Hz, J_3 = 2.1 Hz, 1H), 0.99 (s, 9H), 0.19 (s, 3H), 0.18 (s, 3H).

^{13}C NMR (75 MHz, $CDCl_3$) δ 172.4, 171.1, 160.5, 155.4, 155.0, 135.3, 133.8, 130.9, 129.8, 128.5, 128.03, 128.01, 127.7, 124.3, 120.0, 114.1, 107.4, 79.0, 55.3, 49.9, 40.0, 25.7, 18.2, -4.4, -4.5.

IR (film): ν (cm^{-1}) 2954, 2932, 2893, 2857, 1719, 1611, 1509, 1435, 1403, 1354, 1335, 1247, 1173, 1097, 1032, 908, 834, 801, 771, 730, 690, 559.

HRMS (ESI, *m*/*z*) calcd for $C_{33}H_{38}N_3O_3Si$ $[M+H]^+$: 552.2677, found: 552.2669.

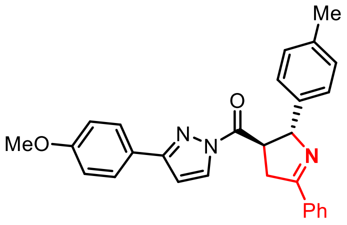

(3-(4-Methoxyphenyl)-1*H*-pyrazol-1-yl)((2*R*,3*R*)-5-phenyl-2-(*p*-tolyl)-3,4-dihydro-2*H*-pyrrol-3-yl)methanone (3i)

According to the typical procedure, the reaction of (*E*)-1-(3-(4-methoxyphenyl)-1*H*-pyrazol-1-yl)-3-(*p*-tolyl)prop-2-en-1-one **1i** (31.8 mg, 0.10 mmol), (1-azidovinyl)benzene **2a** (18.2 mg, 1.25 equiv) and Λ-**RhS** (3.5 mg, 4 mol%) in $CDCl_3$ (1.0 mL, 0.1 M) under open air atmosphere with blue LEDs

for 24 hours, afforded **3i** as a white solid (38.1 mg, 87% isolated yield).

Only one single diastereoisomer was observed through ^{1}H NMR of crude materials. Enantiomeric excess of **3i** was established by HPLC analysis using a Chiralpak IC column, ee = 97% (HPLC: IC, 254 nm, *n*-hexane/isopropanol = 80:20, flow rate 1 mL/min, 40 °C, t_r (major) = 14.9 min, t_r (minor) = 13.8 min). $[\alpha]_D^{22}$ = +42.4° (*c* 1.0, CH_2Cl_2).

^{1}H NMR (500 MHz, $CDCl_3$) δ 8.30 (d, J = 3.0 Hz, 1H), 8.00-7.96 (m, 2H), 7.69-7.65 (m, 2H), 7.51-7.43 (m, 3H), 7.26-7.23 (m, 2H), 7.17-7.12 (m, 2H), 6.95-6.90 (m, 2H), 6.74 (d, J = 3.0 Hz, 1H), 5.77-5.72 (m, 1H), 4.57-4.50 (m, 1H), 3.86 (s, 3H), 3.66 (ddd, J_1 = 17.0 Hz, J_2 = 9.5 Hz, J_3 = 2.0 Hz, 1H), 3.60-3.52 (m, 1H), 2.34 (s, 3H).

^{13}C NMR (125 MHz, $CDCl_3$) δ 172.4, 171.3, 160.5, 155.3, 139.6, 137.0, 133.7, 130.9, 129.8, 129.2, 128.5, 128.1, 127.7, 126.9, 124.3, 114.1, 107.5, 79.1, 55.3, 50.0, 40.0, 21.1.

IR (film): ν (cm^{-1}) 3058, 2953, 2914, 1717, 1613, 1511, 1434, 1398, 1339, 1292, 1245, 1176, 1091, 1027, 934, 902, 846, 813, 769, 728, 688, 559.

HRMS (ESI, *m*/*z*) calcd for $C_{28}H_{26}N_3O_2$ $[M+H]^+$: 436.2020, found: 436.2013.

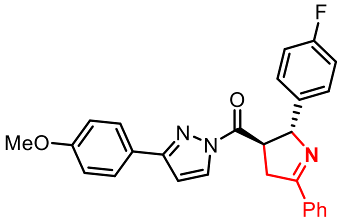

((2*R*,3*R*)-2-(4-Fluorophenyl)-5-phenyl-3,4-dihydro-2*H*-pyrrol-3-yl)(3-(4-methoxyphenyl)-1*H*-pyrazol-1-yl)methanone (3j)

According to the typical procedure, the reaction of (*E*)-3-(4-fluorophenyl)-1-(3-(4-methoxyphenyl)-1*H*-pyrazol-1-yl)prop-2-en-1-one **1j** (32.2 mg, 0.10 mmol), (1-azidovinyl)benzene **2a** (18.2 mg, 1.25 equiv) and Λ-**RhS** (3.5 mg, 4 mol%) in $CDCl_3$ (1.0 mL, 0.1 M) under open air atmosphere with blue LEDs for 24 hours, afforded **3j** as a white solid (39.0 mg, 89% isolated yield).

Only one single diastereoisomer was observed through ^{1}H NMR of crude materials. Enantiomeric excess of **3j** was established by HPLC analysis using a Chiralpak OD-H column, ee = 97% (HPLC: OD-H, 254 nm, *n*-hexane/isopropanol = 90:10, flow rate 1 mL/min, 40 °C, t_r (major) = 11.8 min, t_r (minor) = 14.6 min). $[\alpha]_D^{22}$ = +33.4° (*c* 1.0, CH_2Cl_2).

^{1}H NMR (300 MHz, $CDCl_3$) δ 8.31 (d, J = 2.7 Hz, 1H), 8.00-7.95 (m, 2H), 7.70-7.64 (m, 2H),

7.53-7.42 (m, 3H), 7.37-7.30 (m, 2H), 7.07-6.98 (m, 2H), 6.98-6.90 (m, 2H), 6.76 (d, J = 2.7 Hz, 1H), 5.78-5.72 (m, 1H), 4.56-4.45 (m, 1H), 3.86 (s, 3H), 3.68 (ddd, J_1 = 17.4 Hz, J_2 = 9.3 Hz, J_3 = 2.1 Hz, 1H), 3.57 (ddd, J_1 = 17.1 Hz, J_2 = 6.9 Hz, J_3 = 1.2 Hz, 1H).

^{13}C NMR (75 MHz, $CDCl_3$) δ 172.2, 171.7, 162.2 (d, J = 243.8 Hz), 160.6, 155.5, 138.4 (d, J = 3.1 Hz), 133.6, 131.1, 129.9, 128.61 (d, J = 8.0 Hz), 128.59, 128.1, 127.7, 124.2, 115.3 (d, J = 21.2 Hz), 114.2, 107.6, 78.6, 55.3, 50.0, 40.1.

IR (film): v (cm^{-1}) 3138, 3061, 2964, 2913, 1719, 1608, 1506, 1400, 1336, 1246, 1224, 1177, 1028, 936, 904, 832, 772, 727, 689, 561.

HRMS (ESI, *m*/*z*) calcd for $C_{27}H_{23}FN_3O_2$ [M+H]$^+$: 440.1769, found: 440.1762.

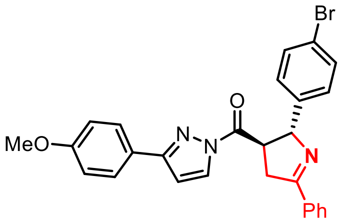

((2*R*,3*R*)-2-(4-Bromophenyl)-5-phenyl-3,4-dihydro-2*H*-pyrrol-3-yl)(3-(4-methoxyphenyl)-1*H*-pyrazol-1-yl)methanone (3k)

According to the typical procedure, the reaction of (*E*)-3-(4-bromophenyl)-1-(3-(4-methoxyphenyl)-1*H*-pyrazol-1-yl)prop-2-en-1-one **1k** (38.3 mg, 0.10 mmol), (1-azidovinyl)benzene **2a** (18.2 mg, 1.25 equiv) and Λ-**RhS** (3.5 mg, 4 mol%) in $CDCl_3$ (1.0 mL, 0.1 M) under open air atmosphere with blue LEDs for 24 hours, afforded **3k** as a white solid (43.6 mg, 87% isolated yield).

Only one single diastereoisomer was observed through ^{1}H NMR of crude materials. Enantiomeric excess of **3k** was established by HPLC analysis using a Chiralpak OD-H column, ee = 97% (HPLC: OD-H, 254 nm, *n*-hexane/isopropanol = 90:10, flow rate 1 mL/min, 40 °C, t_r (major) = 13.7 min, t_r (minor) = 15.8 min). $[\alpha]_D^{22}$ = +50.8° (*c* 1.0, CH_2Cl_2).

^{1}H NMR (300 MHz, $CDCl_3$) δ 8.31 (d, J = 3.0 Hz, 1H), 8.01-7.94 (m, 2H), 7.67-7.60 (m, 2H), 7.55-7.42 (m, 5H), 7.27-7.21 (m, 2H), 7.00-6.92 (m, 2H), 6.76 (d, J = 3.0 Hz, 1H), 5.73-5.68 (m, 1H), 4.51 (ddd, J_1 = 9.0 Hz, J_2 = 7.5 Hz, J_3 = 6.3 Hz, 1H), 3.87 (s, 3H), 3.67 (ddd, J_1 = 17.1 Hz, J_2 = 9.3 Hz, J_3 = 1.8 Hz, 1H), 3.57 (ddd, J_1 = 17.4 Hz, J_2 = 7.5 Hz, J_3 = 1.8 Hz, 1H).

^{13}C NMR (75 MHz, $CDCl_3$) δ 172.00, 171.96, 160.6, 155.5, 141.8, 133.5, 131.6, 131.1, 129.8, 128.8, 128.6, 128.0, 127.7, 124.1, 121.3, 114.2, 107.6, 78.8, 55.3, 49.9, 40.0.

IR (film): ν (cm^{-1}) 2953, 2921, 1709, 1612, 1512, 1431, 1400, 1336, 1295, 1239, 1173, 1017, 945, 905, 834, 767, 727, 692, 620, 555.

HRMS (ESI, *m*/*z*) calcd for $C_{27}H_{23}BrN_3O_2$ $[M+H]^+$: 500.0968, found: 500.0961.

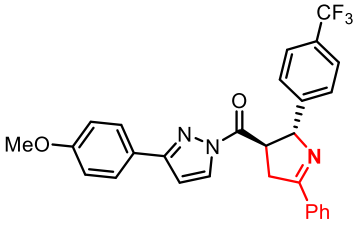

(3-(4-Methoxyphenyl)-1*H*-pyrazol-1-yl)((2*R*,3*R*)-5-phenyl-2-(4-(trifluoromethyl)phenyl)-3,4-dihydro-2*H*-pyrrol-3-yl)methanone (3l)

According to the typical procedure, the reaction of (*E*)-1-(3-(4-methoxyphenyl)-1*H*-pyrazol-1-yl)-3-(4-(trifluoromethyl)phenyl)prop-2-en-1-one **1l** (37.2 mg, 0.10 mmol), (1-azidovinyl)benzene **2a** (18.2 mg, 1.25 equiv) and Λ-**RhS** (3.5 mg, 4 mol%) in $CDCl_3$ (1.0 mL, 0.1 M) under open air atmosphere with blue LEDs for 24 hours, afforded **3l** as a white solid (44.2 mg, 90% isolated yield). Only one single diastereoisomer was observed through ^{1}H NMR of crude materials. Enantiomeric excess of **3l** was established by HPLC analysis using a Chiralpak AD-H column, ee = 95% (HPLC: AD-H, 254 nm, *n*-hexane/isopropanol = 90:10, flow rate 1 mL/min, 40 °C, t_r (major) = 11.3 min, t_r (minor) = 13.1 min). $[\alpha]_D^{22}$ = +27.6° (*c* 1.0, CH_2Cl_2).

^{1}H NMR (500 MHz, $CDCl_3$) δ 8.32 (d, J = 3.0 Hz, 1H), 8.01-7.96 (m, 2H), 7.62-7.56 (m, 4H), 7.55-7.49 (m, 1H), 7.49-7.44 (m, 4H), 6.94-6.90 (m, 2H), 6.76 (d, J = 2.5 Hz, 1H), 5.81-5.77 (m, 1H), 4.53 (ddd, J_1 = 9.5 Hz, J_2 = 7.0 Hz, J_3 = 6.5 Hz, 1H), 3.85 (s, 3H), 3.69 (ddd, J_1 = 17.5 Hz, J_2 = 9.5 Hz, J_3 = 2.0 Hz, 1H), 3.64 (ddd, J_1 = 17.0 Hz, J_2 = 7.5 Hz, J_3 = 1.5 Hz, 1H).

^{13}C NMR (125 MHz, $CDCl_3$) δ 172.3, 171.9, 160.6, 155.5, 146.6, 133.3, 131.3, 130.0, 129.6 (q, J = 32.0 Hz), 128.6, 128.1, 127.6, 127.4, 125.5 (q, J = 3.8 Hz), 124.2 (q, J = 270.4 Hz), 123.9, 114.1, 107.8, 78.9, 55.3, 49.8, 40.1.

IR (film): ν (cm^{-1}) 3061, 2933, 1718, 1614, 1514, 1405, 1355, 1323, 1247, 1165, 1114, 1065, 1024, 908, 834, 800, 767, 691, 601.

HRMS (ESI, *m*/*z*) calcd for $C_{28}H_{23}F_3N_3O_2$ $[M+H]^+$: 490.1737, found: 490.1729.

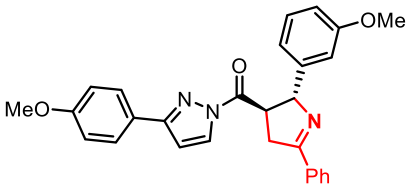

(3-(4-Methoxyphenyl)-1*H*-pyrazol-1-yl)((2*R*,3*R*)-2-(3-methoxyphenyl)-5-phenyl-3,4-dihydro-2*H*-pyrrol-3-yl)methanone (3m)

According to the typical procedure, the reaction of (*E*)-3-(3-methoxyphenyl)-1-(3-(4-methoxyphenyl)-1*H*-pyrazol-1-yl)prop-2-en-1-one **1m** (33.4 mg, 0.10 mmol), (1-azidovinyl)benzene **2a** (18.2 mg, 1.25 equiv) and Λ-**RhS** (3.5 mg, 4 mol%) in $CDCl_3$ (1.0 mL, 0.1 M) under open air atmosphere with blue LEDs for 24 hours, afforded **3m** as a yellow solid (38.0 mg, 84% isolated yield).

Only one single diastereoisomer was observed through ^{1}H NMR of crude materials. Enantiomeric excess of **3m** was established by HPLC analysis using a Chiralpak AD-H column, ee = 98% (HPLC: AD-H, 254 nm, *n*-hexane/isopropanol = 80:20, flow rate 1 mL/min, 40 °C, t_r (major) = 22.7 min, t_r (minor) = 15.1 min). $[\alpha]_D^{22}$ = +11.8° (*c* 1.0, CH_2Cl_2).

^{1}H NMR (300 MHz, $CDCl_3$) δ 8.31 (d, J = 2.7 Hz, 1H), 8.02-7.95 (m, 2H), 7.73-7.66 (m, 2H), 7.54-7.41 (m, 3H), 7.26 (t, J = 7.8 Hz, 1H), 6.99-6.90 (m, 4H), 6.87-6.80 (m, 1H), 6.75 (d, J = 3.0 Hz, 1H), 5.80-5.76 (m, 1H), 4.61-4.51 (m, 1H), 3.85 (s, 3H), 3.75 (s, 3H), 3.69 (ddd, J_1 = 17.4 Hz, J_2 = 9.9 Hz, J_3 = 1.8 Hz, 1H), 3.55 (ddd, J_1 = 17.1 Hz, J_2 = 6.9 Hz, J_3 = 1.8 Hz, 1H).

^{13}C NMR (75 MHz, $CDCl_3$) δ 172.3, 171.4, 160.6, 159.8, 155.4, 144.2, 133.7, 131.0, 129.8, 129.5, 128.5, 128.0, 127.7, 124.3, 119.3, 114.1, 112.8, 112.7, 107.5, 79.1, 55.3, 55.2, 49.8, 40.3.

IR (film): ν (cm^{-1}) 3056, 2955, 2915, 1717, 1610, 1583, 1511, 1488, 1436, 1400, 1331, 1242, 1174, 1149, 1028, 943, 903, 833, 802, 768, 729, 692, 558.

HRMS (ESI, *m/z*) calcd for $C_{28}H_{26}N_3O_3$ $[M+H]^+$: 452.1969, found: 452.1962.

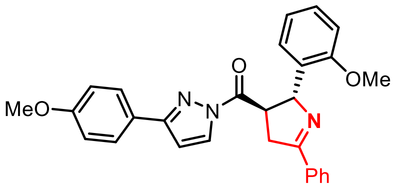

(3-(4-Methoxyphenyl)-1*H*-pyrazol-1-yl)((2*R*,3*R*)-2-(2-methoxyphenyl)-5-phenyl-3,4-dihydro-2*H*-pyrrol-3-yl)methanone (3n)

According to the typical procedure, the reaction of (*E*)-3-(3-methoxyphenyl)-1-(3-(4-methoxyphenyl)-1*H*-pyrazol-1-yl)prop-2-en-1-one **1n** (33.4 mg, 0.10 mmol), (1-azidovinyl)benzene **2a** (18.2 mg, 1.25 equiv) and Λ-**RhS** (3.5 mg, 4 mol%) in $CDCl_3$ (1.0 mL, 0.1 M) under open air atmosphere with blue LEDs for 24 hours, afforded **3n** as a yellow oil (42.3 mg, 94% isolated yield).

Only one single diastereoisomer was observed through ^{1}H NMR of crude materials. Enantiomeric excess of **3n** was established by HPLC analysis using a Chiralpak AD-H column, ee = 94% (HPLC: AD-H, 254 nm, *n*-hexane/isopropanol = 80:20, flow rate 1 mL/min, 40 °C, t_r (major) = 13.5 min, t_r (minor) = 11.5 min). $[\alpha]_D^{22}$ = +60.4° (*c* 1.0, CH_2Cl_2).

^{1}H NMR (300 MHz, $CDCl_3$) δ 8.38 (d, J = 3.0 Hz, 1H), 8.02-7.95 (m, 2H), 7.62-7.55 (m, 2H), 7.54-7.42 (m, 3H), 7.38 (dd, J_1 = 7.5 Hz, J_2 = 1.5 Hz, 1H), 7.24 (td, J_1 = 7.8 Hz, J_2 = 1.8 Hz, 1H), 6.97 (dd, J_1 = 7.5 Hz, J_2 = 0.9 Hz, 1H), 6.93-6.84 (m, 2H), 6.78-6.72 (m, 2H), 6.05-6.00 (m, 1H), 4.62-4.52 (m, 1H), 3.84 (s, 3H), 3.64 (ddd, J_1 = 17.4 Hz, J_2 = 10.5 Hz, J_3 = 2.4 Hz, 1H), 3.53 (ddd, J_1 = 17.1 Hz, J_2 = 7.5 Hz, J_3 = 1.8 Hz, 1H), 3.42 (s, 3H).

^{13}C NMR (75 MHz, $CDCl_3$) δ 173.9, 170.8, 160.4, 156.4, 154.8, 134.0, 131.7, 130.8, 129.5, 128.5, 128.1, 128.0, 127.6, 127.0, 124.4, 120.5, 114.1, 110.0, 107.3, 75.8, 55.3, 54.7, 48.0, 41.5.

IR (film): ν (cm^{-1}) 3059, 3002, 2936, 1719, 1611, 1513, 1490, 1457, 1435, 1403, 1357, 1334, 1292, 1241, 1174, 1098, 1027, 953, 907, 838, 803, 756, 729, 692.

HRMS (ESI, *m*/*z*) calcd for $C_{28}H_{26}N_3O_3$ $[M+H]^+$: 452.1969, found: 452.1961.

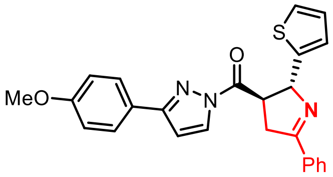

(3-(4-Methoxyphenyl)-1*H*-pyrazol-1-yl)((2*R*,3*R*)-5-phenyl-2-(thiophen-2-yl)-3,4-dihydro-2*H*-pyrrol-3-yl)methanone (3o)

According to the typical procedure, the reaction of (*E*)-1-(3-(4-methoxyphenyl)-1*H*-pyrazol-1-yl)-3-(thiophen-2-yl)prop-2-en-1-one **1o** (31.0 mg, 0.10 mmol), (1-azidovinyl)benzene **2a** (18.2 mg, 1.25 equiv) and Λ-**RhS** (3.5 mg, 4 mol%) in $CDCl_3$ (1.0 mL, 0.1 M) under open air atmosphere with blue LEDs for 24 hours, afforded **3o** as a yellow solid (32.9 mg, 77% isolated yield).

Only one single diastereoisomer was observed through ^{1}H NMR of crude materials. Enantiomeric

excess of **3o** was established by HPLC analysis using a Chiralpak OD-H column, ee = 90% (HPLC: OD-H, 254 nm, *n*-hexane/isopropanol = 90:10, flow rate 1 mL/min, 40 °C, t_r (major) = 15.1 min, t_r (minor) = 17.4 min). $[\alpha]_D^{22}$ = +5.6° (*c* 1.0, CH_2Cl_2).

^{1}H NMR (300 MHz, $CDCl_3$) δ 8.31 (d, J = 3.0 Hz, 1H), 8.00-7.94 (m, 2H), 7.82-7.74 (m, 2H), 7.54-7.41 (m, 3H), 7.24 (dd, J_1 = 5.1 Hz, J_2 = 1.2 Hz, 1H), 7.12-7.07 (m, 1H), 7.02-6.93 (m, 3H), 6.77 (d, J = 3.0 Hz, 1H), 6.09-6.05 (m, 1H), 4.73-4.64 (m, 1H), 3.86 (s, 3H), 3.71 (ddd, J_1 = 17.1 Hz, J_2 = 9.6 Hz, J_3 = 1.8 Hz, 1H), 3.55 (ddd, J_1 = 17.1 Hz, J_2 = 6.9 Hz, J_3 = 1.2 Hz, 1H).

^{13}C NMR (75 MHz, $CDCl_3$) δ 171.9, 171.5, 160.6, 155.6, 145.9, 133.5, 131.1, 129.9, 128.5, 128.1, 127.8, 126.8, 124.5, 124.32, 124.25, 114.2, 107.7, 75.0, 55.3, 50.0, 40.1.

IR (film): ν (cm^{-1}) 2956, 2935, 1714, 1607, 1513, 1434, 1399, 1346, 1288, 1233, 1177, 1087, 1020, 968, 898, 835, 807, 771, 695, 557.

HRMS (ESI, *m*/*z*) calcd for $C_{25}H_{22}N_3O_2S$ [M+H]$^+$: 428.1427, found: 428.1422.

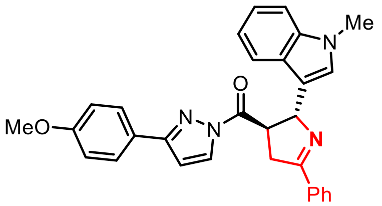

(3-(4-Methoxyphenyl)-1*H*-pyrazol-1-yl)((2*R*,3*R*)-2-(1-methyl-1*H*-indol-3-yl)-5-phenyl-3,4-dihydro-2*H*-pyrrol-3-yl)methanone (3p)

According to the typical procedure with some modifications, the reaction of (*E*)-1-(3-(4-methoxyphenyl)-1*H*-pyrazol-1-yl)-3-(1-methyl-1*H*-indol-3-yl)prop-2-en-1-one **1p** (35.7 mg, 0.10 mmol), (1-azidovinyl)benzene **2a** (18.2 mg, 1.25 equiv) and Λ-**RhS** (6.9 mg, 8 mol%) in $CDCl_3$ (2.0 mL, 0.05 M) under nitrogen atmosphere (degassed with freeze-pump-thaw) with blue LEDs for 24 hours, afforded **3p** as a yellow oil (42.0 mg, 88% isolated yield).

Only one single diastereoisomer was observed through ^{1}H NMR of crude materials. Enantiomeric excess of **3p** was established by HPLC analysis using a Chiralpak OD-H column, ee = 97% (HPLC: OD-H, 254 nm, *n*-hexane/isopropanol = 60:40, flow rate 1 mL/min, 40 °C, t_r (major) = 20.4 min, t_r (minor) = 25.5 min). $[\alpha]_D^{22}$ = +20.4° (*c* 1.0, CH_2Cl_2).

^{1}H NMR (300 MHz, $CDCl_3$) δ 8.31 (d, J = 3.0 Hz, 1H), 8.04-7.98 (m, 2H), 7.67 (d, J = 7.8 Hz, 1H), 7.62-7.56 (m, 2H), 7.51-7.42 (m, 3H), 7.33-7.20 (m, 2H), 7.10-7.03 (m, 2H), 6.93-6.87 (m, 2H), 6.73 (d, J = 2.7 Hz, 1H), 6.10-6.04 (m, 1H), 4.88-4.78 (m, 1H), 3.85 (s, 3H), 3.77 (ddd, J_1 = 16.8

Hz, J_2 = 9.6 Hz, J_3 = 1.8 Hz, 1H), 3.70 (s, 3H), 3.59 (ddd, J_1 = 17.1 Hz, J_2 = 7.2 Hz, J_3 = 1.5 Hz, 1H).

^{13}C NMR (75 MHz, $CDCl_3$) δ 172.9, 170.3, 160.4, 155.2, 137.6, 134.0, 130.8, 129.7, 128.5, 128.0, 127.7, 126.8, 126.6, 124.3, 121.6, 119.8, 119.2, 115.7, 114.0, 109.2, 107.4, 73.2, 55.3, 48.6, 40.4, 32.6.

IR (film): ν (cm^{-1}) 3147, 3056, 2932, 1716, 1612, 1513, 1433, 1402, 1332, 1293, 1244, 1176, 1095, 1027, 904, 836, 800, 769, 730, 692, 644.

HRMS (ESI, *m/z*) calcd for $C_{30}H_{27}N_4O_2$ [M+H]$^+$: 475.2129, found: 475.2122.

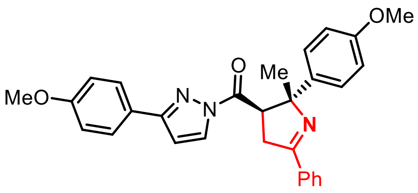

(3-(4-Methoxyphenyl)-1*H*-pyrazol-1-yl)((2*R*,3*R*)-2-(4-methoxyphenyl)-2-methyl-5-phenyl-3,4-dihydro-2*H*-pyrrol-3-yl)methanone (3q)

According to the typical procedure, the reaction of (*E*)-3-(4-methoxyphenyl)-1-(3-(4-methoxyphenyl)-1*H*-pyrazol-1-yl)but-2-en-1-one (*E*)-**1q** (34.8 mg, 0.10 mmol), (1-azidovinyl)benzene **2a** (18.2 mg, 1.25 equiv) and Λ-**RhS** (3.5 mg, 4 mol%) in $CDCl_3$ (1.0 mL, 0.1 M) under open air atmosphere with blue LEDs for 24 hours, afforded **3q** as a yellow solid (42.6 mg, 91% isolated yield).

Only one single diastereoisomer was observed through ^{1}H NMR of crude materials. Enantiomeric excess of **3q** was established by HPLC analysis using a Chiralpak AD-H column, ee = 98% (HPLC: AD-H, 254 nm, *n*-hexane/isopropanol = 80:20, flow rate 1 mL/min, 40 °C, t_r (major) = 12.6 min, t_r (minor) = 10.2 min). $[\alpha]_D^{22}$ = +210.4° (*c* 1.0, CH_2Cl_2).

^{1}H NMR (300 MHz, $CDCl_3$) δ 8.31 (d, J = 2.7 Hz, 1H), 8.04-7.99 (m, 2H), 7.68-7.61 (m, 2H), 7.52-7.42 (m, 5H), 6.98-6.92 (m, 2H), 6.90-6.83 (m, 2H), 6.77 (d, J = 2.7 Hz, 1H), 4.94 (dd, J_1 = 9.0 Hz, J_2 = 5.1 Hz, 1H), 3.87 (s, 3H), 3.80 (dd, J_1 = 17.1 Hz, J_2 = 5.4 Hz, 1H), 3.79 (s, 3H), 3.28 (dd, J_1 = 17.1 Hz, J_2 = 9.0 Hz, 1H), 1.58 (s, 3H).

^{13}C NMR (75 MHz, $CDCl_3$) δ 172.1, 170.0, 160.5, 158.4, 155.1, 140.0, 133.9, 130.8, 129.6, 128.5, 128.1, 127.7, 126.9, 124.3, 114.1, 113.4, 107.5, 81.3, 55.3, 55.2, 52.0, 39.0, 24.4.

IR (film): ν (cm^{-1}) 3061, 2962, 2934, 1715, 1612, 1511, 1436, 1402, 1351, 1335, 1295, 1241, 1176,

1100, 1028, 911, 831, 767, 729, 692.

HRMS (ESI, *m*/*z*) calcd for $C_{29}H_{28}N_3O_3$ $[M+H]^+$: 466.2125, found: 466.2118.

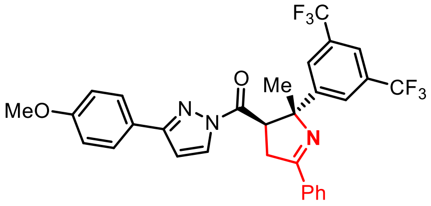

((2*R*,3*R*)-2-(3,5-Bis(trifluoromethyl)phenyl)-2-methyl-5-phenyl-3,4-dihydro-2*H*-pyrrol-3-yl)(3-(4-methoxyphenyl)-1*H*-pyrazol-1-yl)methanone (3r)

According to the typical procedure with some modifications, the reaction of (*E*)-3-(3,5-bis(trifluoromethyl)phenyl)-1-(3-(4-methoxyphenyl)-1*H*-pyrazol-1-yl)but-2-en-1-one **1r** (45.4 mg, 0.10 mmol), (1-azidovinyl)benzene **2a** (18.2 mg, 1.25 equiv) and Λ-**RhS** (6.9 mg, 8 mol%) in $CDCl_3$ (1.0 mL, 0.1 M) under nitrogen atmosphere (degassed with freeze-pump-thaw) with blue LEDs for 45 hours, afforded **3r** as a yellow oil (28.4 mg, 50% isolated yield).

Only one single diastereoisomer was observed through ^1H NMR of crude materials. Enantiomeric excess of **3r** was established by HPLC analysis using a Chiralpak AD-H column, ee = 97% (HPLC: AD-H, 254 nm, *n*-hexane/isopropanol = 90:10, flow rate 1 mL/min, 40 °C, t_r (major) = 5.7 min, t_r (minor) = 4.0 min). $[\alpha]_D^{22}$ = +199.8° (*c* 1.0, CH_2Cl_2).

^1H NMR (300 MHz, $CDCl_3$) δ 8.33 (d, J = 2.7 Hz, 1H), 8.04-7.98 (m, 4H), 7.74 (s, 1H), 7.59-7.47 (m, 5H), 6.95-6.89 (m, 2H), 6.79 (d, J = 3.0 Hz, 1H), 4.96 (dd, J_1 = 9.0 Hz, J_2 = 5.7 Hz, 1H), 3.87 (s, 3H), 3.84 (dd, J_1 = 17.4 Hz, J_2 = 5.4 Hz, 1H), 3.31 (dd, J_1 = 17.4 Hz, J_2 = 9.0 Hz, 1H), 1.63 (s, 3H).

^{13}C NMR (75 MHz, $CDCl_3$) δ 172.2, 170.9, 160.7, 155.7, 150.3, 133.3, 131.4, 131.3 (q, J = 33.3 Hz), 129.7, 128.7, 128.2, 127.5, 126.3-126.2 (m), 123.7, 123.4 (q, J = 271.8 Hz), 121.1-120.9 (m), 114.1, 108.0, 80.6, 55.3, 51.7, 39.1, 24.6.

^{19}F NMR (282 MHz, $CDCl_3$) δ –63.45 (s, 6F).

IR (film): ν (cm^{-1}) 2979, 2936, 1718, 1614, 1515, 1405, 1366, 1276, 1241, 1172, 1125, 1029, 936, 904, 839, 767, 733, 684, 525.

HRMS (ESI, *m*/*z*) calcd for $C_{30}H_{24}F_6N_3O_2$ $[M+H]^+$: 572.1767, found: 572.1758.

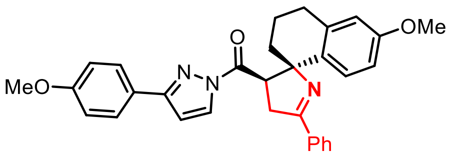

((1*R*,3'*R*)-6-Methoxy-5'-phenyl-3,3',4,4'-tetrahydro-2*H*-spiro[naphthalene-1,2'-pyrrol]-3'-yl)(3-(4-methoxyphenyl)-1*H*-pyrazol-1-yl)methanone (3s)

According to the typical procedure, the reaction of (*E*)-2-(6-methoxy-3,4-dihydronaphthalen-1(2*H*)-ylidene)-1-(3-(4-methoxyphenyl)-1*H*-pyrazol-1-yl)ethan-1-one **1s** (37.4 mg, 0.10 mmol), (1-azidovinyl)benzene **2a** (18.2 mg, 1.25 equiv) and Λ-**RhS** (3.5 mg, 4 mol%) in $CDCl_3$ (1.0 mL, 0.1 M) under open air atmosphere with blue LEDs for 24 hours, afforded **3s** as a yellow solid (46.2 mg, 94% isolated yield).

Only one single diastereoisomer was observed through ^1H NMR of crude materials. Enantiomeric excess of **3s** was established by HPLC analysis using a Chiralpak OD-H column, ee = 99.6% (HPLC: OD-H, 254 nm, *n*-hexane/isopropanol = 80:20, flow rate 1 mL/min, 40 °C, t_r (major) = 8.1 min, t_r (minor) = 6.7 min). $[\alpha]_D^{22}$ = +144.6° (*c* 1.0, CH_2Cl_2).

^1H NMR (300 MHz, $CDCl_3$) δ 8.25 (br s, 1H), 7.97-7.92 (m, 2H), 7.49-7.41 (m, 3H), 7.25 (d, J = 9.0 Hz, 1H), 7.22-7.17 (m, 2H), 6.92 (dd, J_1 = 8.7 Hz, J_2 = 3.0 Hz, 1H), 6.84-6.78 (m, 2H), 6.67 (d, J = 2.7 Hz, 1H), 6.49 (d, J = 2.7 Hz, 1H), 5.03 (t, J = 9.0 Hz, 1H), 3.95 (dd, J_1 = 17.1 Hz, J_2 = 9.0 Hz, 1H), 3.83 (s, 3H), 3.79 (s, 3H), 3.48 (dd, J_1 = 17.4 Hz, J_2 = 9.6 Hz, 1H), 2.77-2.64 (m, 1H), 2.45 (dt, J_1 = 16.5 Hz, J_2 = 5.1 Hz, 1H), 1.95-1.81 (m, 3H), 1.22-1.06 (m, 1H).

^{13}C NMR (75 MHz, $CDCl_3$) δ 171.7, 168.0, 160.4, 158.4, 154.9, 138.4, 134.0, 133.8, 130.7, 129.1, 128.5, 128.0, 127.7, 124.1, 113.8, 113.4, 112.5, 107.5, 80.7, 55.2, 55.1, 53.3, 39.2, 31.9, 30.0, 19.9. (Missing one ^{13}C signal)

IR (film): ν (cm^{-1}) 3000, 2935, 2836, 1712, 1611, 1579, 1507, 1435, 1403, 1335, 1293, 1238, 1173, 1120, 1089, 1030, 942, 911, 837, 804, 765, 728, 691, 631, 559.

HRMS (ESI, *m*/*z*) calcd for $C_{31}H_{30}N_3O_3$ $[M+H]^+$: 492.2282, found: 492.2276.

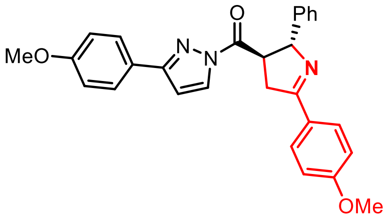

(3-(4-Methoxyphenyl)-1*H*-pyrazol-1-yl)((2*R*,3*R*)-5-(4-methoxyphenyl)-2-phenyl-3,4-dihydro-2*H*-pyrrol-3-yl)methanone (3t)

According to the typical procedure with some modifications, the reaction of (*E*)-1-(3-(4-methoxyphenyl)-1*H*-pyrazol-1-yl)-3-phenylprop-2-en-1-one **1f** (30.4 mg, 0.10 mmol), 1-(1-azidovinyl)-4-methoxybenzene **2b** (21.9 mg, 1.25 equiv) and Λ-**RhS** (6.9 mg, 8 mol%) in $CDCl_3$ (1.0 mL, 0.1 M) under nitrogen atmosphere (degassed with freeze-pump-thaw) with blue LEDs for 24 hours, afforded **3t** as a yellow oil (33.7 mg, 75% isolated yield).

Only one single diastereoisomer was observed through ^{1}H NMR of crude materials. Enantiomeric excess of **3t** was established by HPLC analysis using a Chiralpak AD-H column, ee = 90% (HPLC: AD-H, 254 nm, *n*-hexane/isopropanol = 60:40, flow rate 1 mL/min, 40 °C, t_r (major) = 16.3 min, t_r (minor) = 23.4 min). $[\alpha]_D^{22}$ = +35.6° (*c* 1.0, CH_2Cl_2).

^{1}H NMR (300 MHz, $CDCl_3$) δ 8.30 (d, J = 3.0 Hz, 1H), 7.97-7.91 (m, 2H), 7.71-7.64 (m, 2H), 7.41-7.25 (m, 5H), 7.00-6.90 (m, 4H), 6.74 (d, J = 2.7 Hz, 1H), 5.79-5.73 (m, 1H), 4.58-4.48 (m, 1H), 3.87 (s, 3H), 3.85 (s, 3H), 3.65 (ddd, J_1 = 16.8 Hz, J_2 = 9.3 Hz, J_3 = 1.8 Hz, 1H), 3.53 (ddd, J_1 = 16.8 Hz, J_2 = 6.9 Hz, J_3 = 1.2 Hz, 1H).

^{13}C NMR (75 MHz, $CDCl_3$) δ 172.4, 170.8, 161.9, 160.5, 155.3, 142.8, 129.8, 129.7, 128.5, 127.7, 127.3, 127.0, 126.5, 124.3, 114.1, 113.9, 107.4, 79.1, 55.4, 55.3, 49.9, 40.0.

IR (film): ν (cm^{-1}) 3004, 2959, 2837, 1717, 1608, 1512, 1431, 1403, 1336, 1296, 1245, 1173, 1096, 1027, 945, 908, 834, 802, 770, 729, 697, 556.

HRMS (ESI, *m*/*z*) calcd for $C_{28}H_{26}N_3O_3$ $[M+H]^+$: 452.1980, found: 452.1969.

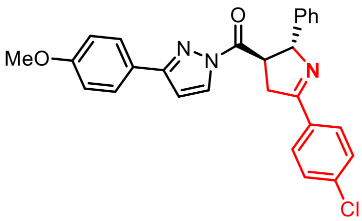

((2*R*,3*R*)-5-(4-Chlorophenyl)-2-phenyl-3,4-dihydro-2*H*-pyrrol-3-yl)(3-(4-methoxyphenyl)-1*H*-pyrazol-1-yl)methanone (3u)

According to the typical procedure, the reaction of (*E*)-1-(3-(4-methoxyphenyl)-1*H*-pyrazol-1-yl)-3-phenylprop-2-en-1-one **1f** (30.4 mg, 0.10 mmol), 1-(1-azidovinyl)-4-chlorobenzene **2c** (22.5 mg, 1.25 equiv) and Λ-**RhS** (3.5 mg, 4 mol%) in $CDCl_3$ (1.0 mL, 0.1 M) under open air atmosphere

with blue LEDs for 24 hours, afforded **3u** as a yellow solid (36.5 mg, 80% isolated yield).

Only one single diastereoisomer was observed through ^{1}H NMR of crude materials. Enantiomeric excess of **3u** was established by HPLC analysis using a Chiralpak AD-H column, ee = 95% (HPLC: AD-H, 254 nm, *n*-hexane/isopropanol = 80:20, flow rate 0.6 mL/min, 40 °C, t_r (major) = 34.0 min, t_r (minor) = 31.6 min). $[\alpha]_D^{22}$ = +21.0° (*c* 1.0, CH_2Cl_2).

^{1}H NMR (300 MHz, $CDCl_3$) δ 8.30 (d, *J* = 3.3 Hz, 1H), 7.94-7.88 (m, 2H), 7.70-7.64 (m, 2H), 7.46-7.40 (m, 2H), 7.37-7.27 (m, 5H), 6.96-6.89 (m, 2H), 6.75 (d, *J* = 2.7 Hz, 1H), 5.80-5.76 (m, 1H), 4.60-4.51 (m, 1H), 3.86 (s, 3H), 3.65 (ddd, J_1 = 17.1 Hz, J_2 = 9.6 Hz, J_3 = 2.1 Hz, 1H), 3.53 (ddd, J_1 = 17.1 Hz, J_2 = 6.9 Hz, J_3 = 1.5 Hz, 1H).

^{13}C NMR (75 MHz, $CDCl_3$) δ 172.2, 170.4, 160.6, 155.5, 142.4, 137.1, 132.2, 129.9, 129.4, 128.8, 128.6, 127.7, 127.5, 126.9, 124.2, 114.1, 107.6, 79.4, 55.3, 49.9, 40.0.

IR (film): *v* (cm^{-1}) 3029, 2928, 2836, 1716, 1612, 1513, 1431, 1402, 1355, 1333, 1293, 1246, 1175, 1091, 1034, 946, 909, 832, 801, 769, 725, 697.

HRMS (ESI, *m*/*z*) calcd for $C_{27}H_{23}ClN_3O_2$ $[M+H]^+$: 456.1473, found: 456.1467.

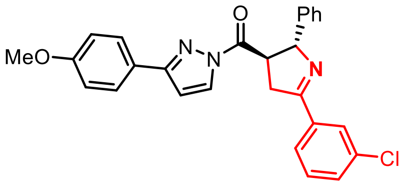

((2*R*,3*R*)-5-(3-Chlorophenyl)-2-phenyl-3,4-dihydro-2*H*-pyrrol-3-yl)(3-(4-methoxyphenyl)-1*H*-pyrazol-1-yl)methanone (3v)

According to the typical procedure, the reaction of (*E*)-1-(3-(4-methoxyphenyl)-1*H*-pyrazol-1-yl)-3-phenylprop-2-en-1-one **1f** (30.4 mg, 0.10 mmol), 1-(1-azidovinyl)-3-chlorobenzene **2d** (22.5 mg, 1.25 equiv) and Λ-**RhS** (3.5 mg, 4 mol%) in $CDCl_3$ (1.0 mL, 0.1 M) under open air atmosphere with blue LEDs for 24 hours, afforded **3v** as a yellow oil (41.8 mg, 92% isolated yield).

Only one single diastereoisomer was observed through ^{1}H NMR of crude materials. Enantiomeric excess of **3v** was established by HPLC analysis using a Chiralpak IG column, ee = 95% (HPLC: IG, 254 nm, *n*-hexane/isopropanol = 80:20, flow rate 1 mL/min, 40 °C, t_r (major) = 22.2 min, t_r (minor) = 18.3 min). $[\alpha]_D^{22}$ = +23.4° (*c* 1.0, CH_2Cl_2).

^{1}H NMR (300 MHz, $CDCl_3$) δ 8.31 (d, *J* = 3.0 Hz, 1H), 8.01 (t, *J* = 1.5 Hz, 1H), 7.83 (dt, J_1 = 7.8 Hz, J_2 = 1.5 Hz, 1H), 7.71-7.64 (m, 2H), 7.49-7.44 (m, 1H), 7.43-7.28 (m, 6H), 6.97-6.90 (m, 2H),

6.75 (d, J = 2.7 Hz, 1H), 5.84-5.78 (m, 1H), 4.62-4.51 (m, 1H), 3.86 (s, 3H), 3.65 (ddd, J_1 = 17.1 Hz, J_2 = 9.3 Hz, J_3 = 2.1 Hz, 1H), 3.53 (ddd, J_1 = 17.1 Hz, J_2 = 7.2 Hz, J_3 = 1.5 Hz, 1H).

^{13}C NMR (75 MHz, $CDCl_3$) δ 172.1, 170.3, 160.6, 155.5, 142.2, 135.4, 134.7, 130.9, 129.84, 129.81, 128.6, 128.1, 127.7, 127.5, 126.9, 126.2, 124.2, 114.1, 107.6, 79.3, 55.3, 49.8, 40.0.

IR (film): ν (cm^{-1}) 3064, 3029, 2931, 1717, 1613, 1514, 1430, 1403, 1355, 1331, 1294, 1244, 1174, 1093, 1032, 949, 908, 837, 770, 729, 691.

HRMS (ESI, *m*/*z*) calcd for $C_{27}H_{23}ClN_3O_2$ $[M+H]^+$: 456.1473, found: 456.1467.

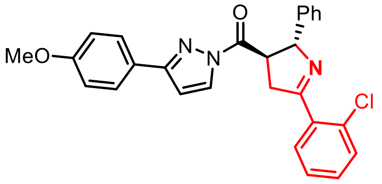

((2*R*,3*R*)-5-(2-Chlorophenyl)-2-phenyl-3,4-dihydro-2*H*-pyrrol-3-yl)(3-(4-methoxyphenyl)-1*H*-pyrazol-1-yl)methanone (3w)

According to the typical procedure, the reaction of (*E*)-1-(3-(4-methoxyphenyl)-1*H*-pyrazol-1-yl)-3-phenylprop-2-en-1-one **1f** (30.4 mg, 0.10 mmol), 1-(1-azidovinyl)-2-chlorobenzene **2e** (22.5 mg, 1.25 equiv) and Λ-**RhS** (3.5 mg, 4 mol%) in $CDCl_3$ (1.0 mL, 0.1 M) under open air atmosphere with blue LEDs for 24 hours, afforded **3w** as a yellow oil (36.3 mg, 80% isolated yield).

Only one single diastereoisomer was observed through ^{1}H NMR of crude materials. Enantiomeric excess of **3w** was established by HPLC analysis using a Chiralpak IG column, ee = 98% (HPLC: IG, 254 nm, *n*-hexane/isopropanol = 80:20, flow rate 1 mL/min, 40 °C, t_r (major) = 22.9 min, t_r (minor) = 15.5 min). $[\alpha]_D^{22}$ = +10.6° (*c* 1.0, CH_2Cl_2).

^{1}H NMR (300 MHz, $CDCl_3$) δ 8.31 (d, J = 3.0 Hz, 1H), 7.81-7.96 (m, 1H), 7.71-7.65 (m, 2H), 7.48-7.27 (m, 8H), 6.96-6.90 (m, 2H), 6.75 (d, J = 3.0 Hz, 1H), 5.81-5.76 (m, 1H), 4.63-4.53 (m, 1H), 3.85 (s, 3H), 3.81 (ddd, J_1 = 17.4 Hz, J_2 = 9.6 Hz, J_3 = 2.1 Hz, 1H), 3.62 (ddd, J_1 = 17.7 Hz, J_2 = 7.5 Hz, J_3 = 1.8 Hz, 1H).

^{13}C NMR (75 MHz, $CDCl_3$) δ 172.5, 172.2, 160.6, 155.5, 142.1, 134.3, 132.6, 130.9, 130.8, 130.3, 129.8, 128.5, 127.7, 127.4, 127.0, 126.9, 124.2, 114.1, 107.6, 78.8, 55.3, 50.5, 43.3.

IR (film): ν (cm^{-1}) 3062, 2933, 2836, 1717, 1610, 1513, 1431, 1403, 1355, 1331, 1294, 1246, 1175, 1028, 908, 836, 802, 755, 728, 700.

HRMS (ESI, *m*/*z*) calcd for $C_{27}H_{23}ClN_3O_2$ $[M+H]^+$: 456.1473, found: 456.1467.

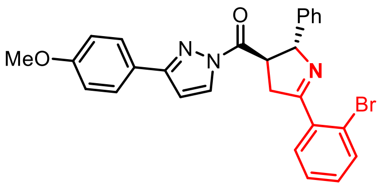

((2*R*,3*R*)-5-(2-Bromophenyl)-2-phenyl-3,4-dihydro-2*H*-pyrrol-3-yl)(3-(4-methoxyphenyl)-1*H*-pyrazol-1-yl)methanone (3x)

According to the typical procedure, the reaction of (*E*)-1-(3-(4-methoxyphenyl)-1*H*-pyrazol-1-yl)-3-phenylprop-2-en-1-one **1f** (30.4 mg, 0.10 mmol), 1-(1-azidovinyl)-2-Bromobenzene **2f** (28.0 mg, 1.25 equiv) and Λ-**RhS** (3.5 mg, 4 mol%) in $CDCl_3$ (1.0 mL, 0.1 M) under open air atmosphere with blue LEDs for 24 hours, afforded **3x** as a yellow solid (44.6 mg, 89% isolated yield).

Only one single diastereoisomer was observed through ^{1}H NMR of crude materials. Enantiomeric excess of **3x** was established by HPLC analysis using a Chiralpak AD-H column, ee = 98% (HPLC: AD-H, 254 nm, *n*-hexane/isopropanol = 80:20, flow rate 1 mL/min, 40 °C, t_r (major) = 16.9 min, t_r (minor) = 10.4 min). $[\alpha]_D^{22}$ = +8.4° (*c* 1.0, CH_2Cl_2).

^{1}H NMR (300 MHz, $CDCl_3$) δ 8.31 (d, J = 3.0 Hz, 1H), 7.71-7.61 (m, 4H), 7.48-7.27 (m, 7H), 6.96-6.90 (m, 2H), 6.74 (d, J = 2.7 Hz, 1H), 5.82-5.76 (m, 1H), 4.64-4.55 (m, 1H), 3.85 (s, 3H), 3.82 (ddd, J_1 = 18.0 Hz, J_2 = 9.6 Hz, J_3 = 2.1 Hz, 1H), 3.58 (ddd, J_1 = 17.4 Hz, J_2 = 7.2 Hz, J_3 = 1.5 Hz, 1H).

^{13}C NMR (75 MHz, $CDCl_3$) δ 173.6, 172.1, 160.6, 155.4, 142.0, 136.7, 133.4, 130.8, 130.5, 129.8, 128.5, 127.7, 127.44, 127.42, 127.0, 124.2, 121.1, 114.1, 107.6, 79.1, 55.3, 50.5, 43.4.

IR (film): ν (cm^{-1}) 3061, 2958, 2931, 2836, 1716, 1610, 1513, 1430, 1402, 1355, 1330, 1293, 1245, 1174, 1095, 1024, 947, 908, 836, 801, 751, 696.

HRMS (ESI, *m*/*z*) calcd for $C_{27}H_{23}BrN_3O_2$ $[M+H]^+$: 500.0968, found: 500.0962.

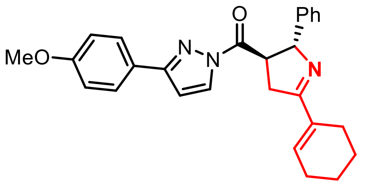

((2*R*,3*R*)-5-(Cyclohex-1-en-1-yl)-2-phenyl-3,4-dihydro-2*H*-pyrrol-3-yl)(3-(4-methoxyphenyl)-1*H*-pyrazol-1-yl)methanone (3y)

According to the typical procedure with some modifications, the reaction of (*E*)-1-(3-(4-methoxyphenyl)-1*H*-pyrazol-1-yl)-3-phenylprop-2-en-1-one **1f** (30.4 mg, 0.10 mmol), 1-(1-azidovinyl)cyclohex-1-ene **2g** (18.7 mg, 1.25 equiv) and Λ-**RhS** (6.9 mg, 8 mol%) in $CDCl_3$ (1.0 mL, 0.1 M) under nitrogen atmosphere (degassed with freeze-pump-thaw) with blue LEDs for 24 hours, afforded **3y** as a yellow oil (31.8 mg, 75% isolated yield).

Only one single diastereoisomer was observed through ^{1}H NMR of crude materials. Enantiomeric excess of **3y** was established by HPLC analysis using a Chiralpak AD-H column, ee = 93% (HPLC: AD-H, 254 nm, *n*-hexane/isopropanol = 90:10, flow rate 1 mL/min, 40 °C, t_r (major) = 13.3 min, t_r (minor) = 11.7 min). $[\alpha]_D^{22}$ = –26.4° (*c* 1.0, CH_2Cl_2).

^{1}H NMR (300 MHz, $CDCl_3$) δ 8.28 (d, J = 3.0 Hz, 1H), 7.70-7.63 (m, 2H), 7.35-7.22 (m, 5H), 6.96-6.89 (m, 2H), 6.72 (d, J = 2.7 Hz, 1H), 6.43 (t, J = 3.6 Hz, 1H), 5.67-5.62 (m, 1H), 4.42-4.33 (m, 1H), 3.85 (s, 3H), 3.40 (ddd, J_1 = 16.8 Hz, J_2 = 9.6 Hz, J_3 = 1.5 Hz, 1H), 3.26 (ddd, J_1 = 16.5 Hz, J_2 = 6.9 Hz, J_3 = 0.9 Hz, 1H), 2.57-2.48 (m, 2H), 2.28-2.21 (m, 2H), 1.80-1.61 (m, 4H).

^{13}C NMR (75 MHz, $CDCl_3$) δ 173.0, 172.6, 160.5, 155.2, 142.9, 135.9, 134.6, 129.8, 128.4, 127.7, 127.2, 126.9, 124.3, 114.1, 107.4, 78.9, 55.3, 49.7, 39.0, 26.1, 25.1, 22.3, 22.0.

IR (film): ν (cm^{-1}) 2930, 2837, 1717, 1608, 1513, 1432, 1402, 1354, 1333, 1293, 1244, 1175, 1094, 1027, 909, 836, 799, 766, 728, 697.

HRMS (ESI, *m/z*) calcd for $C_{27}H_{28}N_3O_2$ $[M+H]^+$: 426.2176, found: 426.2170.

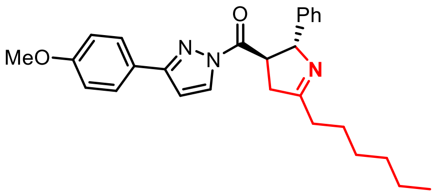

((2*R*,3*R*)-5-Hexyl-2-phenyl-3,4-dihydro-2*H*-pyrrol-3-yl)(3-(4-methoxyphenyl)-1*H*-pyrazol-1-yl) methanone (3z)

According to the typical procedure, the reaction of (*E*)-1-(3-(4-methoxyphenyl)-1*H*-pyrazol-1-yl)-3-phenylprop-2-en-1-one **1f** (30.4 mg, 0.10 mmol), 2-azidooct-1-ene **2h** (19.2 mg, 1.25 equiv) and Λ-**RhS** (3.5 mg, 4 mol%) in $CDCl_3$ (1.0 mL, 0.1 M) under open air atmosphere with blue LEDs for 24 hours, afforded **3z** as a yellow oil (30.1 mg, 70% isolated yield).

Only one single diastereoisomer was observed through ^{1}H NMR of crude materials. Enantiomeric excess of **3z** was established by HPLC analysis using a Chiralpak IG column, ee = 97% (HPLC: IG,

254 nm, *n*-hexane/isopropanol = 80:20, flow rate 1 mL/min, 40 °C, t_r (major) = 15.1 min, t_r (minor) = 12.5 min). $[\alpha]_D^{22}$ = +49.0° (*c* 1.0, CH_2Cl_2).

^{1}H NMR (300 MHz, $CDCl_3$) δ 8.28 (d, J = 2.7 Hz, 1H), 7.68-7.62 (m, 2H), 7.36-7.22 (m, 5H), 6.95-6.89 (m, 2H), 6.72 (d, J = 2.7 Hz, 1H), 5.57-5.52 (m, 1H), 4.43-4.34 (m, 1H), 3.85 (s, 3H), 3.22-3.04 (m, 2H), 2.52 (t, J = 8.3 Hz, 2H), 1.79-1.67 (m, 2H), 1.46-1.30 (m, 6H), 0.91 (t, J = 6.9 Hz, 3H).

^{13}C NMR (75 MHz, $CDCl_3$) δ 177.2, 172.6, 160.5, 155.3, 142.6, 129.7, 128.4, 127.7, 127.2, 126.8, 124.3, 114.1, 107.4, 79.0, 55.3, 49.7, 42.2, 33.6, 31.5, 29.2, 26.5, 22.5, 14.0.

IR (film): *v* (cm^{-1}) 2924, 2856, 1718, 1645, 1610, 1512, 1431, 1402, 1353, 1294, 1247, 1175, 1094, 1031, 950, 908, 837, 771, 698, 619, 525.

HRMS (ESI, *m/z*) calcd for $C_{27}H_{32}N_3O_2$ $[M+H]^+$: 430.2489, found: 430.2484.

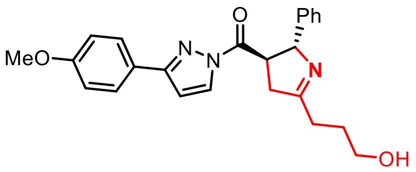

((2*R*,3*R*)-5-(3-Hydroxypropyl)-2-phenyl-3,4-dihydro-2*H*-pyrrol-3-yl)(3-(4-methoxyphenyl)-1*H*-pyrazol-1-yl)methanone (3aa)

According to the typical procedure, the reaction of (*E*)-1-(3-(4-methoxyphenyl)-1*H*-pyrazol-1-yl)-3-phenylprop-2-en-1-one **1f** (30.4 mg, 0.10 mmol), 4-azidopent-4-en-1-ol **2i** (15.9 mg, 1.25 equiv) and Λ-**RhS** (6.9 mg, 8 mol%) in $CDCl_3$ (1.0 mL, 0.1 M) under nitrogen atmosphere (degassed with freeze-pump-thaw) with blue LEDs for 24 hours, afforded **3aa** as a yellow solid (38.0 mg, 69% isolated yield; 94% NMR yield, not stable in silica gel column).

Only one single diastereoisomer was observed through ^{1}H NMR of crude materials. Enantiomeric excess of **3aa** was established by HPLC analysis using a Chiralpak AD-H column, ee = 98% (HPLC: AD-H, 254 nm, *n*-hexane/isopropanol = 90:10, flow rate 1 mL/min, 40 °C, t_r (major) = 23.7 min, t_r (minor) = 26.4 min). $[\alpha]_D^{22}$ = –45.2° (*c* 1.0, CH_2Cl_2).

^{1}H NMR (300 MHz, $CDCl_3$) δ 8.27 (d, J = 3.0 Hz, 1H), 7.68-7.62 (m, 2H), 7.36-7.23 (m, 5H), 6.95-6.89 (m, 2H), 6.72 (d, J = 3.3 Hz, 1H), 5.60-5.55 (m, 1H), 4.46-4.37 (m, 1H), 3.99 (br s, 1H), 3.85 (s, 3H), 3.75 (t, J = 5.7 Hz, 2H), 3.25-3.07 (m, 2H), 2.72-2.51 (m, 2H), 2.06-1.96 (m, 2H).

^{13}C NMR (75 MHz, $CDCl_3$) δ 177.9, 172.2, 160.6, 155.4, 142.1, 129.8, 128.5, 127.7, 127.4, 126.6,

124.2, 114.1, 107.5, 78.6, 62.5, 55.3, 49.7, 42.9, 31.3, 28.7.

IR (film): ν (cm^{-1}) 3120, 2958, 2919, 2840, 1724, 1646, 1611, 1515, 1445, 1348, 1245, 1174, 1025, 955, 906, 836, 768, 730, 699, 609, 524.

HRMS (ESI, *m*/*z*) calcd for $C_{24}H_{26}N_3O_3$ $[M+H]^+$: 404.1969, found: 404.1964.

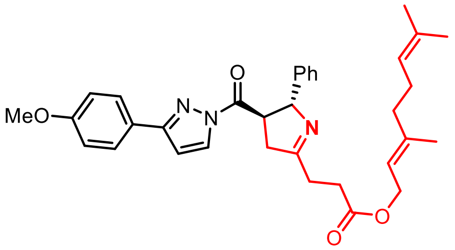

(*E*)-3,7-Dimethylocta-2,6-dien-1-yl 3-((2*R*,3*R*)-3-(3-(4-methoxyphenyl)-1*H*-pyrazole-1-carbonyl)-2-phenyl-3,4-dihydro-2*H*-pyrrol-5-yl)propanoate (3ab)

According to the typical procedure with some modifications, the reaction of (*E*)-1-(3-(4-methoxyphenyl)-1*H*-pyrazol-1-yl)-3-phenylprop-2-en-1-one **1f** (30.4 mg, 0.10 mmol), (*E*)-3,7-dimethylocta-2,6-dien-1-yl 4-azidopent-4-enoate **2j** (34.7 mg, 1.25 equiv) and Λ-**RhS** (6.9 mg, 8 mol%) in $CDCl_3$ (1.0 mL, 0.1 M) under nitrogen atmosphere (degassed with freeze-pump-thaw) with blue LEDs for 24 hours, afforded **3ab** as a yellow solid (45.7 mg, 83% isolated yield; 92% NMR yield, not stable in silica gel column).

Only one single diastereoisomer was observed through ^{1}H NMR of crude materials. Enantiomeric excess of **3ab** was established by HPLC analysis using a Chiralpak OD-H column, ee = 98% (HPLC: OD-H, 254 nm, *n*-hexane/isopropanol = 90:10, flow rate 1 mL/min, 40 °C, t_r (major) = 10.5 min, t_r (minor) = 12.7 min). $[\alpha]_D^{22}$ = +80.6° (*c* 1.0, CH_2Cl_2).

^{1}H NMR (500 MHz, $CDCl_3$) δ 8.27 (d, J = 3.0 Hz, 1H), 7.68-7.63 (m, 2H), 7.33-7.29 (m, 2H), 7.28-7.23 (m, 3H), 6.94-6.90 (m, 2H), 6.72 (d, J = 3.0 Hz, 1H), 5.56-5.50 (m, 1H), 5.36-5.31 (m, 1H), 5.09-5.04 (m, 1H), 4.67-4.58 (m, 2H), 3.38 (ddd, J_1 = 9.5 Hz, J_2 = 6.5 Hz, J_3 = 5.5 Hz, 1H), 3.85 (s, 3H), 3.20 (ddd, J_1 = 17.0 Hz, J_2 = 9.5 Hz, J_3 = 1.5 Hz, 1H), 3.10 (ddd, J_1 = 17.5 Hz, J_2 = 6.5 Hz, J_3 = 1.0 Hz, 1H), 2.94-2.81 (m, 2H), 2.80-2.70 (m, 2H), 2.12-2.05 (m, 2H), 2.04-1.99 (m, 2H), 1.69-1.67 (m, 6H), 1.59 (s, 3H).

^{13}C NMR (125 MHz, $CDCl_3$) δ 175.3, 173.0, 172.4, 160.5, 155.3, 142.5, 142.2, 131.8, 129.7, 128.4, 127.7, 127.2, 126.8, 124.2, 123.7, 118.2, 114.1, 107.5, 78.8, 61.5, 55.3, 49.8, 42.7, 39.5, 30.7, 28.2, 26.2, 25.7, 17.7, 16.4.

IR (film): ν (cm^{-1}) 2961, 2918, 2848, 1722, 1651, 1611, 1513, 1432, 1402, 1354, 1293, 1247, 1173, 1095, 1031, 945, 905, 836, 771, 698.

HRMS (ESI, *m/z*) calcd for $C_{34}H_{40}N_3O_4$ $[M+H]^+$: 554.3013, found: 554.3003.

Synthetic Applications

Modification of a Drug Derivative

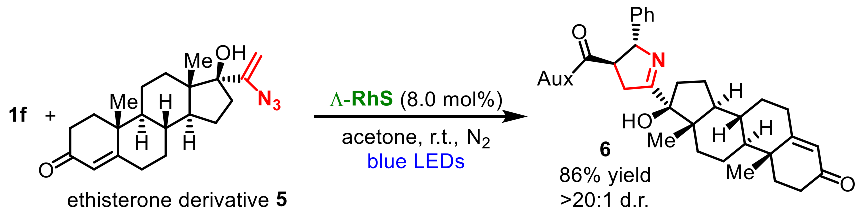

(8*R*,9*S*,10*R*,13*S*,14*S*,17*S*)-17-(1-Azidovinyl)-17-hydroxy-10,13-dimethyl-1,2,6,7,8,9,10,11,12,13,14,15,16,17-tetradecahydro-3*H*-cyclopenta[*a*]phenanthren-3-one **5** was synthesized according to Bi's procedure.[30] Ethisterone derived vinyl azide **5** is unstable in $CDCl_3$. So acetone is chosen as solvent for this transformation.

According to the typical procedure with some modifications, the reaction of (*E*)-1-(3-(4-methoxyphenyl)-1*H*-pyrazol-1-yl)-3-phenylprop-2-en-1-one **1f** (30.4 mg, 0.10 mmol), **5** (44.4 mg, 1.25 equiv) and Λ-**RhS** (6.9 mg, 8 mol%) in **acetone** (1.0 mL, 0.1 M) under nitrogen atmosphere (degassed with freeze-pump-thaw) with blue LEDs for 48 hours, afforded **6** as a yellow solid (54.6 mg, 86%).

A d.r. value of >20:1 was observed through ^{1}H NMR of crude materials. Reference sample was obtained by carrying out the reaction with *rac*-**RhS**. $[\alpha]_D^{22}$ = +27.8° (*c* 1.0, CH_2Cl_2).

^{1}H NMR (500 MHz, $CDCl_3$) δ 8.27 (d, J = 3.0 Hz, 1H), 7.63-7.59 (m, 2H), 7.33-7.23 (m, 5H), 6.93-6.89 (m, 2H), 6.72 (d, J = 3.0 Hz, 1H), 5.73 (s, 1H), 5.62-5.56 (m, 1H), 4.50-4.43 (m, 1H), 3.84 (s, 3H), 3.82 (br s, 1H), 3.31 (ddd, J_1 = 17.0 Hz, J_2 = 9.0 Hz, J_3 = 1.5 Hz, 1H), 3.22 (ddd, J_1 = 17.5 Hz, J_2 = 8.5 Hz, J_3 = 2.0 Hz, 1H), 2.44-2.32 (m, 4H), 2.30-2.24 (m, 1H), 2.06-1.94 (m, 2H), 1.92-1.84 (m, 1H), 1.82-1.42 (m, 8H), 1.26-1.14 (m, 1H), 1.21 (s, 3H), 1.09-1.00 (m, 1H), 1.06 (s, 3H), 0.99-0.89 (m, 1H).

^{13}C NMR (125 MHz, CD_2Cl_2) δ 199.3, 182.2, 172.4, 171.4, 161.0, 155.7, 142.8, 130.1, 128.8, 128.0, 127.7, 127.1, 124.4, 124.0, 114.4, 107.9, 85.8, 77.6, 55.6, 54.2, 51.8, 50.1, 48.0, 42.9, 39.0, 36.6, 36.4, 36.1, 34.3, 33.2, 33.1, 32.2, 24.3, 21.1, 17.6, 14.7.

IR (film): ν (cm^{-1}) 3385, 2940, 2860, 1719, 1662, 1613, 1514, 1432, 1404, 1356, 1294, 1245, 1177, 1027, 951, 909, 836, 802, 773, 728, 698.

HRMS (ESI, *m*/*z*) calcd for $C_{40}H_{46}N_3O_4$ $[M+H]^+$: 632.3483, found: 632.3473.

Gram Scale Synthesis

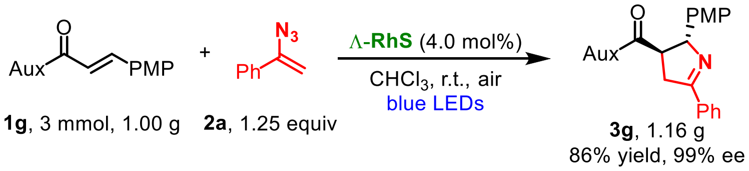

According to typical procedure with some modifications, a 100 mL round-button flask equipped with a stirring bar was charged with **1g** (1.00 g, 3.0 mmol), **2a** (0.55 g, 1.25 equiv), Λ-**RhS** (104 mg, 4 mol%) and $CHCl_3$ (30 mL, 0.1 M) under open air atmosphere. Then the flask was sealed with a rubber stopper, connected to an air balloon, and illuminated with blue LEDs for 24 hours (Supplementary Fig. 2). Since the reaction proceeds with the exclusion of N_2, a balloon is suggested for large scale reaction. The desired product **3g** could be obtained by flash chromatography in 1.16 g (86% yield) with 99% ee.

Functional Group Conversions

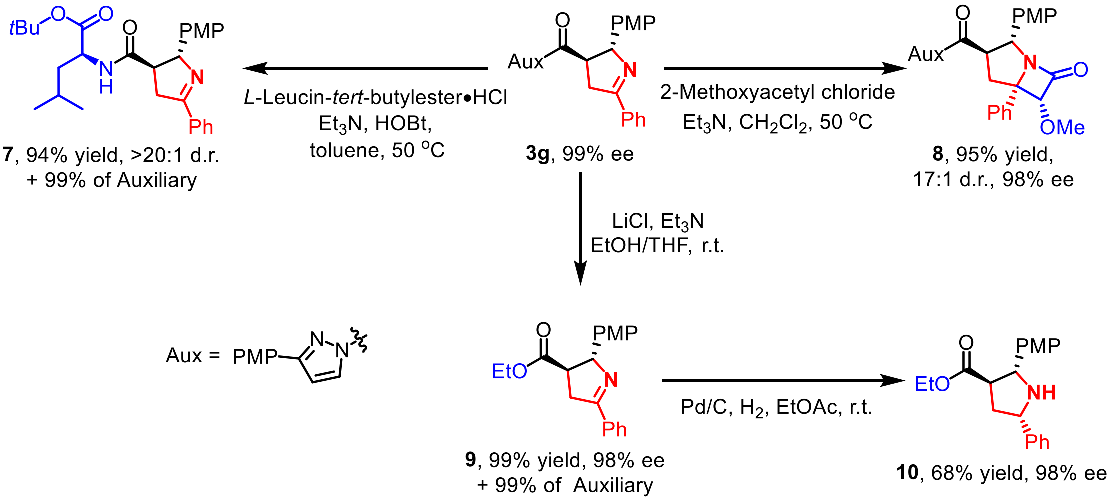

Formation of Amide 7

According to literature report[31], *L*-leucin-*tert*-butylester hydrochlorid (44.7 mg, 0.2 mmol), toluene (0.5 mL), and Et_3N (20.2 mg, 0.2 mmol) was added into a 10 mL Schlenk tube equipped with a stirring bar. After stirred at room temperature for 10 min, **3g** (45.2 mg, 0.1 mmol),

1-hydroxybenzotriazole (27.0 mg, 0.2 mmol), and toluene (1.0 mL) was added continuously. Then the tube was sealed and heated at 50 ^{o}C for 16 hours. The mixture was concentrated under reduced pressure and the crude residue was subjected to ^{1}H NMR to determine the d.r. value. Then target product **7** was isolated by flash chromatography (*n*-Hexane/EtOAc) in 94% yield (43.8 mg). At the same time the auxiliary 3-(4-methoxyphenyl)-1*H*-pyrazole could be obtained in 99% yield (17.4 mg).

***tert*-Butyl ((2*R*,3*R*)-2-(4-methoxyphenyl)-5-phenyl-3,4-dihydro-2*H*-pyrrole-3-carbonyl)-*L*-leucinate (7)**

Only one single diastereoisomer (>20:1 d.r.) was observed through ^{1}H NMR of crude materials. Reference sample was obtained by carrying out the reaction with *rac*-**3g**. $[\alpha]_D^{22}$ = –23.4^{o} (*c* 1.0, CH_2Cl_2).

^{1}H NMR (300 MHz, $CDCl_3$) δ 7.93-7.86 (m, 2H), 7.47-7.36 (m, 3H), 7.23-7.15 (m, 2H), 6.93-6.84 (m, 2H), 5.71 (d, J = 8.7 Hz, 1H), 5.32-5.26 (m, 1H), 4.56 (td, J_1 = 9.0 Hz, J_2 = 4.8 Hz, 1H), 3.80 (s, 3H), 3.52 (ddd, J_1 = 17.1 Hz, J_2 = 9.0 Hz, J_3 = 2.4 Hz, 1H), 3.36 (ddd, J_1 = 16.8 Hz, J_2 = 9.0 Hz, J_3 = 1.8 Hz, 1H), 2.98-2.85 (m, 1H), 1.69-1.51 (m, 2H), 1.44 (s, 9H), 0.97 (d, J = 6.3 Hz, 3H), 0.94 (d, J = 6.0 Hz, 3H). (Missing the N-H signal)

^{13}C NMR (75 MHz, $CDCl_3$) δ 172.3, 172.2, 171.7, 159.1, 135.3, 133.9, 130.8, 128.4, 127.9, 114.1, 81.9, 80.0, 55.3, 54.5, 51.3, 42.0, 39.9, 27.9, 24.9, 22.8, 22.0. (Missing one ^{13}C singal)

IR (film): ν (cm^{-1}) 3287, 3062, 2857, 2823, 1731, 1644, 1616, 1539, 1512, 1453, 1371, 1335, 1297, 1243, 1147, 1037, 917, 825, 762, 731, 690, 578, 536.

HRMS (ESI, *m*/*z*) calcd for $C_{28}H_{37}N_2O_4$ $[M+H]^+$: 465.2748, found: 465.2743.

Formation of Carbapenem Analogue 8

According to literature report[32], **3g** (22.6 mg, 0.05 mmol), Et_3N (15.2 mg, 0.15 mmol), and CH_2Cl_2 (1.0 mL) was added into a 10 mL Schlenk tube equipped with a stirring bar. 2-Methoxyacetyl chloride (16.3 mg, 0.15 mmol, in 1.0 mL CH_2Cl_2) was added to the mixture dropwise at room temperature. Then the tube was sealed and heated at 50 ^{o}C for 16 hours. The mixture was concentrated under reduced pressure and the crude residue was subjected to ^{1}H NMR to determine the d.r. value. Then target product **8** was isolated by flash chromatography (*n*-Hexane/EtOAc) in 95% yield (24.9 mg).

(2*R*,3*R*,5*S*,6*R*)-6-Methoxy-2-(4-methoxyphenyl)-3-(3-(4-methoxyphenyl)-1*H*-pyrazole-1-carbonyl)-5-phenyl-1-azabicyclo[3.2.0]heptan-7-one (8)

A d.r. value of 17:1 was determined through ^{1}H NMR of crude materials. Reference sample was obtained by carrying out the reaction with *rac*-**3g**. Enantiomeric excess of **8** was established by HPLC analysis using a Chiralpak OD-H column, ee = 98% (HPLC: OD-H, 254 nm, *n*-hexane/isopropanol = 80:20, flow rate 1 mL/min, 40 °C, t_r (major) = 20.0 min, t_r (minor) = 12.6 min). $[\alpha]_D^{22}$ = +188.8° (*c* 1.0, CH_2Cl_2).

^{1}H NMR (500 MHz, $CDCl_3$) δ 8.24 (d, J = 3.0 Hz, 1H), 7.68-7.63 (m, 2H), 7.60-7.55 (m, 2H), 7.50-7.43 (m, 2H), 7.42-7.37 (m, 1H), 7.26-7.22 (m, 2H), 6.95-6.90 (m, 2H), 6.81-6.76 (m, 2H), 6.72 (d, J = 3.0 Hz, 1H), 5.57 (d, J = 8.0 Hz, 1H), 4.75-4.66 (m, 1H), 4.69 (s, 1H), 3.85 (s, 3H), 3.74 (s, 3H), 3.34 (dd, J_1 = 13.0 Hz, J_2 = 6.5, 1H), 3.16 (s, 3H), 2.56 (dd, J_1 = 12.5 Hz, J_2 = 10.5, 1H).

^{13}C NMR (75 MHz, $CDCl_3$) δ 173.3, 170.3, 160.7, 159.1, 155.6, 137.5, 131.3, 129.9, 128.31, 128.27, 128.0, 127.7, 127.4, 124.0, 114.2, 113.9, 107.8, 90.4, 73.4, 64.0, 58.2, 55.3, 55.2, 54.8, 41.0.

IR (film): ν (cm^{-1}) 2999, 2884, 2835, 1763, 1716, 1673, 1612, 1550, 1514, 1433, 1406, 1335, 1295, 1246, 1217, 1175, 1142, 1093, 1023, 936, 907, 833, 774, 727, 700, 644, 577.

HRMS (ESI, *m*/*z*) calcd for $C_{31}H_{30}N_3O_5$ $[M+H]^+$: 524.2180, found: 524.2188.

Configuration of 8 was Determined by NMR Spectroscopy

Sample of **8** dissolved in 0.6 mL of $CDCl_3$ were performed on a Bruker AVHD 500 MHz spectrometer equipped with a 5 mm TXI probe with z-gradient. NOESY experiments were performed with mixing time of 1.5 s. Chemical shifts are referenced with the rest solvent signal.

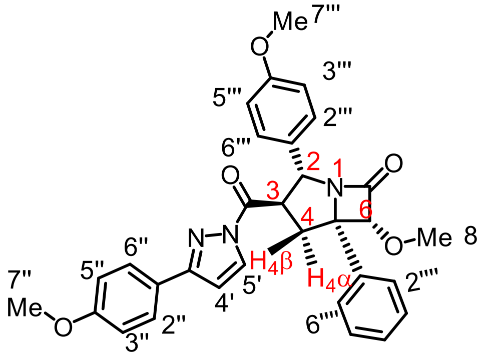

The NOESY cross peaks characteristic for the stereostructure of the molecule are presented in Supplementary Table 8. With the known configuration at positions 2 and 3, through the observation of the NOE contacts among the substituents the configuration at positions 5 and 6 is unequivocally determined. There are additional NOESY cross peaks observed. A whole assignment to these signals are presented in Supplementary Fig. 6. The vicinal coupling constants observed are $^3J_{H2H3}$ = 8 Hz, $^3J_{H3H4\alpha}$ = 6 Hz, and $^3J_{H3H4\beta}$ = 10 Hz. These values are consistent with the determined structure.

Formation of Ester 9

According to literature report[31], **3g** (45.2 mg, 0.1 mmol), EtOH (0.8 mL), THF (0.2 mL), LiCl (21.2 mg, 0.5 mmol), and Et_3N (50.5 mg, 0.5 mmol) was added in sequence to a 10 mL Schlenk tube equipped with a stirring bar. After stirred at room temperature for 16 hours, the mixture was concentrated under reduced pressure and the crude residue was subjected to ^{1}H NMR to determine the d.r. value. Then target product **9** was isolated by flash chromatography (*n*-Hexane/EtOAc) in 99% yield (32.0 mg) at the same time the auxiliary 3-(4-methoxyphenyl)-1*H*-pyrazole could be obtained in 99% yield (17.4 mg).

Ethyl (2*R*,3*R*)-2-(4-methoxyphenyl)-5-phenyl-3,4-dihydro-2*H*-pyrrole-3-carboxylate (9)

Only one single diastereoisomer was observed through ^{1}H NMR of crude materials. Reference sample was obtained by carrying out the reaction with *rac*-**3g**. Enantiomeric excess of **9** was established by HPLC analysis using a Chiralpak AD-H column, ee = 98% (HPLC: AD-H, 254 nm, *n*-hexane/isopropanol = 80:20, flow rate 1 mL/min, 40 °C, t_r (major) = 13.1 min, t_r (minor) = 9.5 min). $[\alpha]_D^{22}$ = +4.2° (*c* 1.0, CH_2Cl_2).

^{1}H NMR (300 MHz, $CDCl_3$) δ 7.98-7.91 (m, 2H), 7.51-7.40 (m, 3H), 7.29-7.20 (m, 2H), 6.94-6.85 (m, 2H), 5.55-5.49 (m, 1H), 4.31-4.17 (m, 2H), 3.81 (s, 3H), 3.53-3.35 (m, 2H), 3.15 (td, J_1 = 9.0 Hz, J_2 = 6.6 Hz, 1H), 1.31 (t, J = 7.1 Hz, 3H).

^{13}C NMR (75 MHz, $CDCl_3$) δ 174.0, 171.3, 158.9, 135.4, 133.8, 130.9, 128.5, 127.9, 127.7, 114.0, 79.2, 61.0, 55.3, 51.3, 39.4, 14.2.

IR (film): ν (cm^{-1}) 2969, 2930, 2904, 2838, 1724, 1612, 1579, 1511, 1447, 1373, 1332, 1301, 1242, 1166, 1112, 1025, 870, 823, 762, 690, 586, 545.

HRMS (ESI, *m*/*z*) calcd for $C_{20}H_{22}NO_3$ $[M+H]^+$: 324.1594, found: 324.1590.

Formation of Pyrrolidine 10

9 (32.3 mg, 0.1 mmol), Pd/C (16 mg, 50%), and EtOAc (1.0 mL) was added in sequence to a 10 mL flask equipped with a stirring bar. After bubbling with H_2 for 5 minutes, the flask was allowed to stir at room temperature for 12 hours. The mixture was filtered and concentrated under reduced pressure. Then target product **10** was isolated by flash chromatography (*n*-Hexane/EtOAc) in 68% yield (22.2 mg, a white solid) as a single diastereoisomer.

Ethyl (2*R*,3*R*,5*S*)-2-(4-methoxyphenyl)-5-phenylpyrrolidine-3-carboxylate (10)

Reference sample was obtained by carrying out the reaction with *rac*-**9**. Enantiomeric excess of **10** was established by HPLC analysis using a Chiralpak IC column, ee = 98% (HPLC: IC, 220 nm, *n*-hexane/isopropanol = 90:10, flow rate 1 mL/min, 40 °C, t_r (major) = 8.2 min, t_r (minor) = 7.2 min). $[\alpha]_D^{22}$ = –74.8° (*c* 1.0, CH_2Cl_2).

^1H NMR (300 MHz, $CDCl_3$) δ 7.55-7.42 (m, 4H), 7.40-7.32 (m, 2H), 7.31-7.21 (m, 1H), 6.94-6.86 (m, 2H), 5.50 (d, J = 7.8 Hz, 1H), 4.45 (t, J = 8.3 Hz, 1H), 4.21-4.10 (m, 2H), 3.82 (s, 3H), 2.98-2.87 (m, 1H), 2.28-2.46 (m, 1H), 2.25-2.05 (br s, 1H), 2.16-2.02 (m, 1H), 1.23 (t, J = 7.2 Hz, 3H).

^{13}C NMR (75 MHz, $CDCl_3$) δ 174.7, 159.0, 144.0, 135.1, 128.4, 128.0, 127.1, 126.7, 113.8, 65.3, 61.3, 60.6, 55.3, 51.9, 38.8, 14.2.

IR (film): ν (cm^{-1}) 3315, 2975, 2941, 2834, 1724, 1609, 1508, 1451, 1375, 1345, 1299, 1242, 1168, 1099, 1033, 828, 755, 700, 579, 537.

HRMS (ESI, *m*/*z*) calcd for $C_{20}H_{24}NO_3$ $[M+H]^+$: 326.1751, found: 326.1746.

Configuration of 10 was Determined by NMR Spectroscopy:

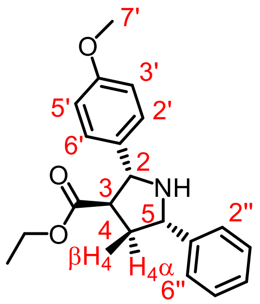

An assignment of the main NOESY cross peaks are given in Supplementary Fig. 7, with the known configuration at positions 2 and 3, we observed strong NOE cross peak between H-3 and H-4α,

which enabled the assignment of H-4α. Strong NOE was observed between H-4α and H-2”/H-6”, which determined an α-substitution of phenyl group at position 5. In addition, a medium NOE contact was observed between H-2 and H-5, which verified the assigned configuration. The vicinal coupling constants observed are $^3J_{H2H3}$ = 7.8 Hz, $^3J_{H3H4\alpha}$ = 10.5 Hz, $^3J_{H3H4\beta}$ = 5.1 Hz, $^3J_{H4\alpha H4\beta}$ = 13.0 Hz, $^3J_{H4\alpha H5}$ = 8.1 Hz, $^3J_{H4\beta H5}$ = 8.1 Hz.

Stereochemical Assignments via Single Crystal X-Ray Diffraction

X-ray data were collected with a STOE 4 circuit StadiVari diffractometer with CuKα radiation (microfocus tube with multilayer optics) and Dectris Pilatus 300K detector at 100 K. Scaling and absorption correction was performed by using the X-AREA/LANA software package of STOE. Structures were solved using direct methods in SHELXT and refined using the full matrix least squares procedure in SHELXL-2017. The hydrogen atoms were placed in calculated positions and refined as riding on their respective C atom, and Uiso(H) was set at 1.2 Ueq(Csp2) and 1.5 Ueq(Csp3). Disorder was refined using restraints for both the geometry and the anisotropic displacement factors. The relative and absolute configuration of **3k** has been determined.

Single crystals of **3k** suitable for X-ray diffraction were obtained by slow diffusion of a solution of **3k** (30 mg) in CH_2Cl_2 (0.5 mL) layered with *n*-hexane (0.5 mL) at room temperature for several days in a NMR tube.

Crystal structure, data and details of the structure determination for **3k** are presented in the Supplementary Fig. 4 and Supplementary Table 6.

Supplementary References

1. *X-Area Pilatus3_SV*, STOE & Cie GmbH, Darmstadt, Germany, 2016.
2. *X-Area Recipe*, STOE & Cie GmbH, Darmstadt, Germany, 2015.
3. *X-Area Integrate*, STOE & Cie GmbH, Darmstadt, Germany, 2016.
4. *X-Area LANA*, STOE & Cie GmbH, Darmstadt, Germany, 2016.
5. Sheldrick, G. SHELXT - Integrated space-group and crystal-structure determination. *Acta Crystallographica Section A* **71**, 3-8 (2015).
6. Sheldrick, G. Crystal structure refinement with SHELXL. *Acta Crystallographica Section C* **71**, 3-8 (2015).
7. Brandenburg, K. *Diamond - Crystal and Molecular Structure Visualization*, Crystal Impact - Dr. H. Putz & Dr. K. Brandenburg GbR, Bonn, Germany, 2014.
8. Hubschle, C. B., Sheldrick, G. M. & Dittrich, B. ShelXle: A Qt graphical user interface for SHELXL. *J. Appl. Crystallogr.* **44**, 1281-1284 (2011).
9. Parsons, S., Flack, H. D. & Wagner, T. Use of intensity quotients and differences in absolute structure refinement. *Acta Crystallographica Section B* **69**, 249-259 (2013).
10. Huo, H. *et al.* Asymmetric photoredox transition-metal catalysis activated by visible light. *Nature* **515**, 100-103 (2014).
11. Ma, J., Shen, X., Harms, K. & Meggers, E. Expanding the family of bis-cyclometalated chiral-at-metal rhodium(III) catalysts with a benzothiazole derivative. *Dalton Trans.* **45**, 8320-8323 (2016).
12. Boehringer Ingelheim Pharma, K. G., Ed. 2003. Patent number: US2003/162788.
13. Liu, Z., Liao, P. & Bi, X. General silver-catalyzed hydroazidation of terminal alkynes by combining TMS-N_3 and H_2O: Synthesis of vinyl azides. *Org. Lett.* **16**, 3668-3671 (2014).
14. Xiang, L., Niu, Y., Pang, X., Yang, X. & Yan, R. I_2-catalyzed synthesis of substituted imidazoles from vinyl azides and benzylamines. *Chem. Commun.* **51**, 6598-6600 (2015).
15. Huo, H., Harms, K. & Meggers, E. Catalytic, enantioselective addition of alkyl radicals to alkenes via visible-light-activated photoredox catalysis with a chiral rhodium complex. *J. Am. Chem. Soc.* **138**, 6936-6939 (2016).
16. Rajam, S. *et al.* Triplet sensitized photolysis of a vinyl azide: Direct detection of a triplet vinyl

azide and nitrene. *J. Org. Chem.* **79**, 9325-9334 (2014).

17. Huang, X. *et al.* Direct visible-light-excited asymmetric Lewis acid catalysis of intermolecular [2+2] photocycloadditions. *J. Am. Chem. Soc.* **139**, 9120-9123 (2017).
18. Padwa, A. & Smolanoff, J. Photocycloaddition of arylazirenes with electron-deficient olefins. *J. Am. Chem. Soc.* **93**, 548-550 (1971).
19. Padwa, A., Dharan, M., Smolanoff, J. & Wetmore, S. I. Photochemical transformations of small ring heterocyclic compounds. XlVII. Electronic details of the photocycloaddition of arylazirines. *J. Am. Chem. Soc.* **95**, 1954-1961 (1973).
20. Wang, Y. F., Toh, K. K., Lee, J. Y. & Chiba, S. Synthesis of isoquinolines from α-aryl vinyl azides and internal alkynes by Rh–Cu bimetallic cooperation. *Angew. Chem., Int. Ed.* **50**, 5927-5931 (2011).
21. Cismesia, M. A. & Yoon, T. P. Characterizing chain processes in visible light photoredox catalysis. *Chem. Sci.* **6**, 5426-5434 (2015).
22. Frisch, M. J., Trucks, G. W., Schlegel, H. B., Scuseria, G. E., Robb, M. A., Cheeseman, J. R., Zakrzewski, V. G., Montgomery Jr., J. A., Stratmann, R. E., Burant, J. C., Dapprich, S., Millam, J. M., Daniels, A. D., Kudin, K. N., Strain, M. C., Farkas, O., Tomasi, J., Barone, V., Cossi, M., Cammi, R., Mennucci, B., Pomelli, C., Adamo, C., Clifford, S., Ochterski, J., Petersson, G. A., Ayala, P. Y., Cui, Q., Morokuma, K., Salvador, P., Dannenberg, J. J., Malick, D. K., Rabuck, A. D., Raghavachari, K., Foresman, J. B., Cioslowski, J., Ortiz, J. V., Baboul, A. G., Stefanov, B. B., Liu, G., Liashenko, A., Piskorz, P., Komaromi, I., Gomperts, R., Martin, R. L., Fox, D. J., Keith, T., Al-Laham, M. A., Peng, C. Y., Nanayakkara, A., Challacombe, M., Gill, P. M. W., Johnson, B., Chen, W., Wong, M. W., Andres, J. L., Gonzalez, C., Head-Gordon, M., Replogle, E. S. & Pople, J. A. *Gaussian 98* (Gaussian, Inc., Pittsburgh, PA, 1998).
23. Zhao, Y. & Truhlar, D. G. The M06 suite of density functionals for main group thermochemistry, thermochemical kinetics, noncovalent interactions, excited states, and transition elements: two new functionals and systematic testing of four M06-class functionals and 12 other functionals. *Theor. Chem. Acc.* **120**, 215-241 (2008).
24. Grimme, S., Antony, J., Ehrlich, S. & Krieg, H. A consistent and accurate ab initio parametrization of density functional dispersion correction (DFT-D) for the 94 elements H-Pu. *J. Chem. Phys.* **132**, 154104 (2010).

25. Hay, P. J. & Wadt, W. R. Ab initio effective core potentials for molecular calculations. Potentials for the transition metal atoms Sc to Hg. *J. Chem. Phys.* **82**, 270-283 (1985).
26. Becke, A. D. Density-functional thermochemistry. III. The role of exact exchange. *J. Chem. Phys.* **98**, 5648-5652 (1993).
27. Lee, C., Yang, W. & Parr, R. G. Development of the Colle-Salvetti correlation-energy formula into a functional of the electron density. *Phys. Rev. B* **37**, 785-789 (1988).
28. Wang, C. *et al.* Asymmetric Lewis acid catalysis directed by octahedral rhodium centrochirality. *Chem. Sci.* **6**, 1094-1100 (2015).
29. Shen, X., Harms, K., Marsch, M. & Meggers, E. A rhodium catalyst superior to iridium congeners for enantioselective radical amination activated by visible light. *Chem. Eur. J.* **22**, 9102-9105 (2016).
30. Liu, Z. *et al.* Silver(I)-catalyzed hydroazidation of ethynyl carbinols: Synthesis of 2-azidoallyl alcohols. *Angew. Chem., Int. Ed.* **53**, 5305-5309 (2014).
31. Uraguchi, D., Yamada, K. & Ooi, T. Highly *E*-selective and enantioselective Michael addition to electron-deficient internal alkynes under chiral iminophosphorane catalysis. *Angew. Chem., Int. Ed.* **54**, 9954-9957 (2015).
32. Burgemeister, T., Dannhardt, G. & Mach-Bindl, M. Carbapename und carbacephame: Addition von ketenen an fünf- und sechsgliedrige cycloimine. *Arch. Pharm.* **321**, 521-525 (1988).
