# Supplementary material for: Catalytic asymmetric synthesis of a nitrogen heterocycle through stereocontrolled direct photoreaction from electronically excited state
Source: Nat Commun. 2017 Dec 21;8:2245. doi: 10.1038/s41467-017-02148-1 (PMC5740077; doi:10.1038/s41467-017-02148-1)
Supplement: Supplementary file 3 — Description of Additional Supplementary Files [file 41467_2017_2148_MOESM3_ESM.pdf]

Description of Additional Supplementary Files

File Name: Supplementary Data 1
Description: Calculated spin densities of triplet excited states and coordinates.
