# Supplementary material for: Catalytic asymmetric synthesis of a nitrogen heterocycle through stereocontrolled direct photoreaction from electronically excited state
Source: Nat Commun. 2017 Dec 21;8:2245. doi: 10.1038/s41467-017-02148-1 (PMC5740077; doi:10.1038/s41467-017-02148-1)
Supplement: Supplementary file 4 — Supplementary Data 1 [file 41467_2017_2148_MOESM4_ESM.pdf]

Spin densities of 1f in Triplet State

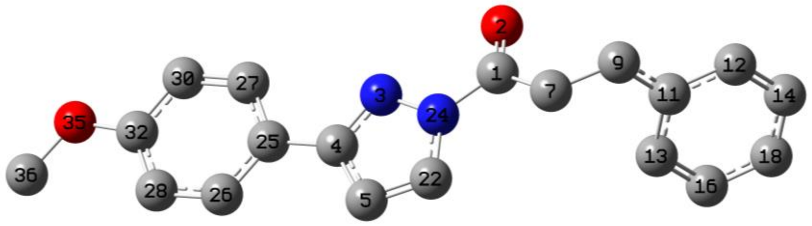

1	C	-0.03789	21	H	-0.01174
**2**	**O**	**0.171902**	22	C	0.024499
3	N	-0.03283	23	H	0.004456
4	C	0.00458	24	N	0.027671
5	C	-0.00361	25	C	-0.00251
6	H	-0.00026	26	C	0.006955
**7**	**C**	**0.931667**	27	C	0.003013
8	H	-0.02006	28	C	-0.00161
**9**	**C**	**0.716444**	29	H	-0.00046
10	H	0.012064	30	C	-0.00282
**11**	**C**	**-0.28775**	31	H	-0.00032
**12**	**C**	**0.130876**	32	C	0.005818
**13**	**C**	**0.373472**	33	H	0.000159
14	C	-0.11021	34	H	0.000133
15	H	-0.01453	35	O	0.001343
16	C	-0.14276	36	C	-0.0002
17	H	-0.02029	37	H	0.000137
**18**	**C**	**0.264024**	38	H	-3E-06
19	H	0.005525	39	H	0.000191
20	H	0.004908			

Spin densities of RhS-1f in Triplet State

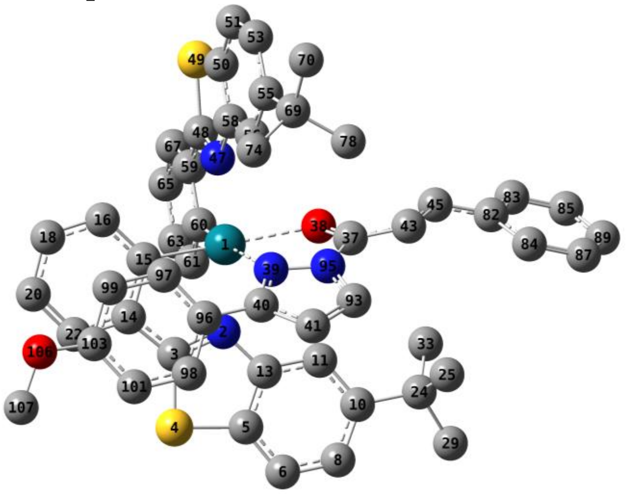

**1**	**Rh**	**0.075647**	**38**	**O**	**0.215158**	75	H	-0.000085
2	N	-0.007706	39	N	-0.030234	76	H	-0.00203
3	C	-0.000705	40	C	0.042749	77	H	0.000239
4	S	0.001781	41	C	0.011854	78	C	-0.012248
5	C	0.003916	42	H	-0.001288	79	H	0.000159
6	C	-0.002063	**43**	**C**	**0.676651**	80	H	-0.001947
7	H	0.000002	44	H	-0.074146	81	H	0.020672
8	C	-0.016601	**45**	**C**	**0.483079**	**82**	**C**	**-0.152753**
9	H	0.000354	46	H	-0.040459	**83**	**C**	**0.189387**
10	C	0.044239	47	N	0.000932	**84**	**C**	**0.321203**
11	C	0.08166	48	C	0.003656	85	C	-0.102695
12	H	0.00757	49	S	-0.001963	86	H	-0.010664
13	C	-0.000795	50	C	-0.000747	87	C	-0.104568
14	C	-0.006259	51	C	0.000794	88	H	-0.004609
15	C	-0.017741	52	H	0.000011	**89**	**C**	**0.383949**
16	C	0.000775	53	C	0.00226	90	H	0.003598
17	H	-0.000405	54	H	-0.000127	91	H	0.005109
18	C	0.001761	55	C	-0.004594	92	H	-0.015046
19	H	-0.000025	56	C	-0.004546	93	C	0.060593
20	C	0.003005	57	H	0.014821	94	H	0.013958
21	H	0.000048	58	C	-0.003096	95	N	0.044315
22	C	-0.00119	59	C	-0.000298	96	C	-0.000598
23	H	0.000106	60	C	0.001029	97	C	0.012936
24	C	-0.140123	61	C	-0.005492	98	C	0.005073
25	C	0.197211	62	H	-0.000139	99	C	0.000333
26	H	0.007788	63	C	-0.000433	100	H	0.002185
27	H	0.008002	64	H	-0.000025	101	C	-0.001647
28	H	-0.253231	65	C	-0.000281	102	H	-0.000324
29	C	-0.01587	66	H	-0.000012	103	C	0.009731
30	H	-0.000475	67	C	0.000383	104	H	0.000101
31	H	0.000907	68	H	-0.000055	105	H	0.000024
32	H	0.000829	69	C	0.00643	106	O	0.00406
33	C	0.062787	70	C	0.000478	107	C	-0.000336
34	H	0.005552	71	H	-0.00002	108	H	-0.000016
35	H	-0.021954	72	H	-0.000101	109	H	0.000309
36	H	0.001784	73	H	0.000178	110	H	0.000329
37	C	0.021328	74	C	-0.00301			

Spin densities of IrS-1f in Triplet State

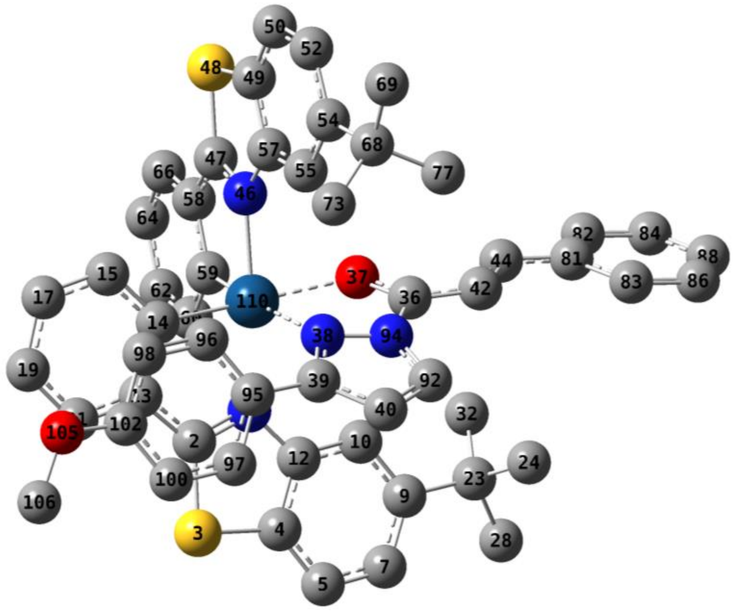

1	N	0.000258	38	N	-0.0432	75	H	-0.00153
2	C	0.011921	39	C	0.086342	76	H	-0.00024
3	S	0.002367	40	C	-0.00996	77	C	-0.00251
4	C	0.007067	41	H	0.000086	78	H	-8.2E-05
5	C	-0.00933	**42**	**C**	**-0.10699**	79	H	-0.00044
6	H	0.000282	43	H	-0.00422	80	H	0.001177
7	C	0.001849	**44**	**C**	**0.470752**	**81**	**C**	**-0.11913**
8	H	-0.00049	45	H	-0.04121	**82**	**C**	**0.165679**
9	C	0.012664	46	N	0.032337	**83**	**C**	**0.1494**
10	C	0.032298	47	C	-0.0037	84	C	-0.05423
11	H	0.056615	48	S	-0.00363	85	H	-0.00645
12	C	-0.00336	49	C	0.014054	86	C	-0.07779
13	C	0.081886	50	C	-0.00839	87	H	-0.00517
14	C	-0.12248	51	H	0.000363	**88**	**C**	**0.181559**
15	C	0.102515	52	C	0.022133	89	H	0.002844
16	H	-0.00229	53	H	-0.00095	90	H	0.002776
17	C	-0.04902	54	C	-0.01459	91	H	-0.00703
18	H	0.001053	55	C	0.00707	92	C	0.085005
19	C	0.135809	56	H	0.024548	93	H	-0.00189
20	H	-0.00399	57	C	-0.01552	94	N	-0.04263
21	C	-0.05524	58	C	0.071339	95	C	-0.02362
22	H	0.003425	59	C	-0.03593	96	C	0.028997
23	C	-0.06472	60	C	0.062253	97	C	0.028263
24	C	0.028122	61	H	-0.00314	98	C	-0.01328
25	H	0.004276	62	C	-0.03824	99	H	0.001693
26	H	0.001268	63	H	0.000836	100	C	0.004602
27	H	-0.03387	64	C	0.102725	101	H	0.001068

28	C	-0.00396	65	H	-0.00385	102	C	0.026274
29	H	-6.3E-05	66	C	-0.04523	103	H	0.000207
30	H	0.000187	67	H	0.001407	104	H	0.000031
31	H	0.000328	68	C	0.009826	105	O	0.002978
32	C	0.033324	69	C	-0.00127	106	C	0.000251
33	H	0.002753	70	H	-5.9E-05	107	H	-0.00015
34	H	-0.01924	71	H	-1.7E-05	108	H	0.000114
35	H	0.001375	72	H	0.00009	109	H	0.000338
36	C	0.220452	73	C	-0.00186	**110**	**Ir**	**0.607806**
**37**	**O**	**0.166651**	74	H	0.000173			

Coordinates of 1f in Triplet State				Coordinates of 1f in Singlet State			
C	-1.68926700	-0.93934000	0.56445600	C	-1.46742700	0.81790100	0.09190500
O	-1.73562800	-1.81252700	-0.27561600	O	-1.36489200	2.02041000	0.14521500
N	0.67741500	-0.65099700	0.28596600	N	0.91443500	0.56212600	0.02384800
C	1.57631800	0.26986000	0.60561100	C	1.77978700	-0.43467300	0.12768400
C	0.97919600	1.33843800	1.33941400	C	1.10950800	-1.68186400	0.31079300
H	1.44851200	2.23396800	1.72738700	H	1.54282900	-2.66653900	0.43564600
C	-2.82269400	-0.55757400	1.39283800	C	-2.74532900	0.09665900	-0.01771600
H	-2.65847400	0.03716900	2.29522400	H	-2.74796500	-0.97412800	-0.20840700
C	-4.16034800	-1.00822500	1.04373500	C	-3.89316100	0.78691200	0.08552000
H	-4.51650600	-1.95710000	1.45361900	H	-3.79764400	1.86035800	0.26777100
C	-5.00510600	-0.28855400	0.17308100	C	-5.24773800	0.26547000	-0.01732000
C	-6.30639500	-0.76687400	-0.12911900	C	-6.32261900	1.14238600	0.17927100
C	-4.58958500	0.92681300	-0.43193100	C	-5.52619900	-1.07867200	-0.30711700
C	-7.13828800	-0.06799800	-0.98236400	C	-7.63453500	0.69390500	0.09546500
H	-6.63783700	-1.70179700	0.32315800	H	-6.11422400	2.18910400	0.40181400
C	-5.43031300	1.61709100	-1.28420800	C	-6.83443900	-1.52715700	-0.39209300
H	-3.58815000	1.30692700	-0.22027900	H	-4.70852700	-1.77911800	-0.47240600
C	-6.70931700	1.12815700	-1.56575700	C	-7.89355200	-0.64249600	-0.19008900
H	-8.13254000	-0.45344500	-1.20272600	H	-8.45617700	1.39033400	0.25222200
H	-5.09098200	2.54524700	-1.74141600	H	-7.03428300	-2.57297600	-0.61905700
H	-7.36691100	1.67498300	-2.23895700	H	-8.92031600	-0.99782600	-0.25763400
C	-0.33553600	0.99659500	1.44798600	C	-0.21615300	-1.37109400	0.30222300
H	-1.17120900	1.51896900	1.89675400	H	-1.08652800	-1.99894900	0.43604100
N	-0.48645100	-0.21242100	0.81025100	N	-0.30752100	-0.00989200	0.12267700
C	2.97507900	0.11342300	0.21143000	C	3.21878100	-0.18933400	0.04593100
C	3.93192700	1.07782900	0.52151800	C	4.13896800	-1.22972500	0.15733200
C	3.38392700	-1.02578100	-0.49962800	C	3.70689300	1.11219000	-0.14971000
C	5.26310800	0.92910800	0.14345600	C	5.50922700	-1.00078100	0.07713800
H	3.64362200	1.97309900	1.07199200	H	3.78993500	-2.25056800	0.31054200
C	4.70024000	-1.18678700	-0.87974900	C	5.06293400	1.35346400	-0.23239700
H	2.64018000	-1.78127500	-0.74545300	H	2.99280800	1.92896600	-0.23575800
C	5.65203200	-0.21010100	-0.56091800	C	5.97666400	0.29801300	-0.12108000
H	5.98170700	1.70308600	0.40160500	H	6.19601500	-1.83814600	0.16973400
H	5.02949600	-2.06517000	-1.43130100	H	5.45450800	2.35745800	-0.38372900
O	6.91645800	-0.46216300	-0.97622500	O	7.28455500	0.63794900	-0.21978500
C	7.90627400	0.48939900	-0.67978200	C	8.23903700	-0.38539700	-0.09883600
H	8.02115700	0.62976900	0.40593800	H	8.11792800	-1.14822700	-0.88329000
H	8.84185000	0.10137600	-1.09092000	H	9.21772100	0.08878600	-0.20949600
H	7.68517600	1.46275600	-1.14383400	H	8.18433000	-0.87528400	0.88546000

Coordinates of RhS-1f in Triplet State

Rh	0.57458600	0.06875500	1.01194700
N	0.52615000	-2.00302500	0.75553700
C	1.72457900	-2.54988200	0.75524500
S	1.75514800	-4.22137100	0.26551200
C	0.01428200	-4.16014400	0.04226400
C	-0.86230300	-5.15448400	-0.37419600
H	-0.50680800	-6.15179600	-0.62442800
C	-2.21309500	-4.84585400	-0.46756800
H	-2.88917200	-5.62825700	-0.80375600
C	-2.72421700	-3.57691400	-0.13264600
C	-1.83170300	-2.60040800	0.30152500
H	-2.16767900	-1.61336400	0.61071700
C	-0.46912700	-2.88283400	0.37066000
C	2.84876900	-1.71728400	1.09222700
C	2.50980200	-0.36000200	1.29430600
C	3.52731800	0.53250500	1.61941700
H	3.31004500	1.58767300	1.78236500
C	4.84369300	0.08333200	1.72466400
H	5.62877400	0.79365500	1.98084400
C	5.17253700	-1.25432500	1.50225800
H	6.20642400	-1.58417600	1.57387900
C	4.17253400	-2.15901300	1.18246100
H	4.41535200	-3.20704900	0.99754300
C	-4.20054400	-3.22689800	-0.32035900
C	-4.32983700	-2.46027400	-1.64346900
H	-5.38285200	-2.21574100	-1.84793800
H	-3.94160400	-3.05117900	-2.48530200
H	-3.76677800	-1.51417300	-1.60216100
C	-5.08688600	-4.47023600	-0.39524200
H	-6.14093100	-4.16793700	-0.44978300
H	-4.96654800	-5.10759400	0.49124100
H	-4.88220200	-5.07618600	-1.28769100
C	-4.71654900	-2.34434900	0.82014700
H	-5.77799200	-2.10698700	0.65573700
H	-4.18493700	-1.38460000	0.88836200
H	-4.62573100	-2.85165800	1.79003300
C	-1.94275400	0.64728600	-0.65383000
O	-1.58876300	0.63272000	0.54283600
N	0.27839900	0.05470400	-1.28890800
C	0.86682700	-0.55191800	-2.31834600
C	-0.07967500	-0.80020700	-3.34964600
H	0.11053400	-1.26067700	-4.31086400
C	-3.23805200	1.04192500	-1.07204200
H	-3.43830300	1.15742600	-2.13706200
C	-4.26768500	1.30559900	-0.09056600
H	-3.93479500	1.54608200	0.91883300
N	0.80292700	2.08962300	1.41006200
C	0.77150500	2.38272700	2.69446800
S	0.91186400	4.08293200	3.04564100
C	0.96601200	4.40981200	1.32016400
C	1.02842300	5.62427900	0.64908500
H	1.08935900	6.56540400	1.19135500
C	0.98870200	5.61530900	-0.74066500
H	1.02135300	6.57204900	-1.25566000
C	0.89758400	4.42407600	-1.48208200
C	0.87130700	3.21626900	-0.79087900
H	0.80644800	2.26648000	-1.31588400

Coordinates of RhS-1f in Singlet State

Rh	0.59682900	0.05346900	1.01329600
N	0.72696100	-2.00338500	0.66618500
C	1.96920500	-2.44250500	0.64149300
S	2.14273000	-4.09370400	0.11421800
C	0.40117400	-4.18200700	-0.09064000
C	-0.38645700	-5.24544800	-0.51225400
H	0.05614700	-6.19940600	-0.79116500
C	-1.76252400	-5.06617500	-0.56654900
H	-2.36913500	-5.90466100	-0.89870500
C	-2.38405100	-3.86035100	-0.19123200
C	-1.57576400	-2.80685800	0.23177300
H	-1.99680300	-1.85974700	0.56444300
C	-0.19055400	-2.95970000	0.27027900
C	3.01985700	-1.52200800	0.98833400
C	2.56100300	-0.20838700	1.23740400
C	3.49747300	0.76899300	1.56274600
H	3.18439800	1.79364600	1.76098600
C	4.85234900	0.44437100	1.62295000
H	5.57403000	1.21918000	1.87863600
C	5.29957900	-0.85070000	1.35623800
H	6.36146900	-1.08299600	1.39451500
C	4.38115900	-1.83835600	1.03633400
H	4.71589900	-2.85461700	0.81938400
C	-3.89824400	-3.66985700	-0.29085400
C	-4.19767800	-2.84643200	-1.55051900
H	-5.28098200	-2.68972500	-1.66103900
H	-3.82934700	-3.35562100	-2.45252800
H	-3.72126300	-1.85741600	-1.49583000
C	-4.64339600	-5.00067700	-0.39342100
H	-5.72574900	-4.81698500	-0.39209500
H	-4.41503000	-5.66007500	0.45513600
H	-4.41206400	-5.53985700	-1.32178000
C	-4.43587300	-2.92652800	0.93658700
H	-5.52704100	-2.82092700	0.85887900
H	-4.01797400	-1.91520400	1.03447100
H	-4.21173700	-3.47309100	1.86251100
C	-2.01943300	0.47281300	-0.59766600
O	-1.64267400	0.50549400	0.57329300
N	0.23541400	0.08371300	-1.28512600
C	0.84579800	-0.44298100	-2.34114100
C	-0.10462800	-0.77293200	-3.35013900
H	0.10173000	-1.20447700	-4.32140100
C	-3.37835900	0.70096800	-1.03113400
H	-3.57629600	0.81737900	-2.09472800
C	-4.37243700	0.69260600	-0.11407000
H	-4.07099600	0.56884300	0.92990500
N	0.63481400	2.06318300	1.50044200
C	0.58791600	2.30673700	2.79369200
S	0.57201100	4.00085500	3.20123900
C	0.58845300	4.39057700	1.48686300
C	0.54497000	5.62512900	0.85014700
H	0.52467800	6.55209100	1.41912500
C	0.50977100	5.65278300	-0.54026400
H	0.45976100	6.62298500	-1.02874700
C	0.52477800	4.48031200	-1.31594400
C	0.59851200	3.25619300	-0.65943700
H	0.61740400	2.31938900	-1.21014100

C	0.89364400	3.20536200	0.60002600	C	0.62045100	3.20809700	0.72961500
C	0.59792800	1.29919700	3.62730300	C	0.53965400	1.17268500	3.68247600
C	0.47256700	0.03552800	3.00658700	C	0.54533100	-0.06991600	3.00577200
C	0.24530900	-1.07998200	3.80396200	C	0.47167300	-1.23764500	3.75595200
H	0.14108600	-2.06767500	3.35638100	H	0.47956400	-2.21081400	3.26636100
C	0.14646300	-0.93557500	5.18845900	C	0.38893300	-1.16785000	5.14693900
H	-0.03317300	-1.81564100	5.80438400	H	0.33442300	-2.08935800	5.72498500
C	0.27920600	0.31336000	5.79571000	C	0.38392100	0.06048200	5.80823200
H	0.20440000	0.40591100	6.87676000	H	0.32249000	0.09690200	6.89343100
C	0.50198500	1.43729900	5.01541900	C	0.45593700	1.23619200	5.07658500
H	0.59631500	2.42012200	5.47921500	H	0.44434700	2.20106800	5.58527300
C	0.78544100	4.39627800	-3.00511000	C	0.43440100	4.48096500	-2.84020900
C	0.81471900	5.79662900	-3.61313700	C	0.33691200	5.89281900	-3.41265200
H	0.72974800	5.72655800	-4.70525800	H	0.27099400	5.84496500	-4.50740300
H	1.75462800	6.32010700	-3.39010100	H	1.21983800	6.49721300	-3.16309400
H	-0.01980500	6.41654500	-3.25754700	H	-0.55678800	6.42022900	-3.05155000
C	1.94408800	3.58816700	-3.60004200	C	1.67552500	3.80266800	-3.43251300
H	1.88594400	3.59499400	-4.69735500	H	1.63222200	3.82654800	-4.53026900
H	1.92419900	2.53716700	-3.27965800	H	1.75721400	2.74812200	-3.13164500
H	2.91483300	4.01202300	-3.30940200	H	2.59480300	4.31548300	-3.11865700
C	-0.54435800	3.73220000	-3.38995300	C	-0.81811500	3.70053000	-3.26384000
H	-0.66461300	3.72567900	-4.48242400	H	-0.92052300	3.71163500	-4.35804100
H	-1.39590600	4.27421900	-2.95502500	H	-1.72536700	4.14505500	-2.83085100
H	-0.59401400	2.68815500	-3.04470100	H	-0.77193900	2.64735400	-2.94661200
C	-5.62561200	1.11459800	-0.32903300	C	-5.79336500	0.77716300	-0.36078400
C	-6.55898600	1.26120800	0.75176700	C	-6.66867400	0.64484700	0.72850800
C	-6.15397000	0.72539400	-1.60666600	C	-6.33116700	0.95365100	-1.64688200
C	-7.89799200	1.02663900	0.56784100	C	-8.04264200	0.69017000	0.54149100
H	-6.17350900	1.55610800	1.72706500	H	-6.25314100	0.50273100	1.72651200
C	-7.50006300	0.49212500	-1.77234900	C	-7.70173200	1.00168900	-1.83188500
H	-5.48217200	0.61401400	-2.45599200	H	-5.67012400	1.05768300	-2.50621200
C	-8.38631900	0.63576700	-0.69421400	C	-8.55960500	0.87006600	-0.73822200
H	-8.58921900	1.14172300	1.40011200	H	-8.71219500	0.58722800	1.39250100
H	-7.88137900	0.19877400	-2.74845200	H	-8.11141800	1.14181000	-2.82995900
H	-9.44964200	0.45390300	-0.83451300	H	-9.63681400	0.90802800	-0.88790500
C	-1.26287400	-0.30184200	-2.88758100	C	-1.31398100	-0.39566100	-2.85181200
H	-2.25036400	-0.28437900	-3.33145800	H	-2.31037600	-0.46971200	-3.26803900
N	-1.02474400	0.22174700	-1.64250500	N	-1.09152800	0.13277900	-1.60166400
C	2.29019200	-0.86288700	-2.30461600	C	2.29267000	-0.61530700	-2.37447700
C	3.22139300	0.05927300	-1.80060400	C	3.14719800	0.37962400	-1.87369100
C	2.74373200	-2.09979800	-2.76398000	C	2.84650700	-1.79846400	-2.86304400
C	4.56324100	-0.25215500	-1.76309600	C	4.51152900	0.18467400	-1.85570800
H	2.87807900	1.02869100	-1.43739400	H	2.72822500	1.31430700	-1.49833800
C	4.09005100	-2.43920400	-2.69508100	C	4.21815500	-2.02058700	-2.81508700
H	2.02584900	-2.82899100	-3.14238800	H	2.19147400	-2.58057500	-3.24903100
C	5.00572400	-1.51356400	-2.18556100	C	5.05638400	-1.02874700	-2.29751100
H	5.30020400	0.44871000	-1.37717600	H	5.19015100	0.94194100	-1.46953600
H	4.41427700	-3.42061600	-3.03126100	H	4.61733400	-2.96543300	-3.17369800
O	6.32404600	-1.74339000	-2.04204800	O	6.39101300	-1.14511100	-2.16789400
C	6.82295600	-3.00907400	-2.41259900	C	6.99243000	-2.36453200	-2.54130400
H	7.89439700	-2.99044700	-2.20254300	H	8.05989300	-2.25540100	-2.33834900
H	6.35107000	-3.81097300	-1.82504300	H	6.59227700	-3.20204500	-1.94993100
H	6.66738200	-3.20520000	-3.48310400	H	6.84724600	-2.57443900	-3.61060300

Coordinates of IrS-1f in Triplet State

N	0.37630600	-2.05415300	0.57770300
C	1.56876100	-2.62222900	0.54551900
S	1.56589600	-4.25140000	-0.04677700
C	-0.17331700	-4.14948300	-0.25752200
C	-1.06659200	-5.11336300	-0.70981000
H	-0.72652800	-6.09823200	-1.02211600
C	-2.41423100	-4.78687500	-0.74875300
H	-3.10766800	-5.54444400	-1.10588000
C	-2.91112800	-3.53333300	-0.33381300
C	-2.00079900	-2.58040200	0.11488700
H	-2.31437800	-1.59747600	0.46116800
C	-0.64028900	-2.88586200	0.14102400
C	2.70274900	-1.82751000	0.95132500
C	2.36678500	-0.47679700	1.25430800
C	3.39226300	0.36233300	1.71382700
H	3.17828900	1.40647000	1.94435100
C	4.68609700	-0.11853800	1.86250300
H	5.47054900	0.54416800	2.22374900
C	4.99755300	-1.44461200	1.54401800
H	6.01852000	-1.80451500	1.65102900
C	4.00552500	-2.30326100	1.08595200
H	4.24806200	-3.33809400	0.84063800
C	-4.39689400	-3.19123000	-0.43999200
C	-4.60578200	-2.44052000	-1.76263500
H	-5.66298700	-2.16393400	-1.88445300
H	-4.30928600	-3.06036100	-2.62103300
H	-4.01444400	-1.51384800	-1.78077900
C	-5.27533300	-4.44280500	-0.43760200
H	-6.33221800	-4.14709800	-0.42359700
H	-5.08944100	-5.06758300	0.44703000
H	-5.13109000	-5.05948500	-1.33448900
C	-4.84363700	-2.29743600	0.72083700
H	-5.91366000	-2.06970100	0.62091800
H	-4.31979300	-1.33273300	0.73528500
H	-4.68686300	-2.79186900	1.68977800
C	-1.89098300	0.58039900	-0.78498700
O	-1.52825300	0.58676400	0.46333200
N	0.38400200	0.04973200	-1.29091800
C	1.07762100	-0.49049100	-2.31404700
C	0.21353700	-0.72834600	-3.39572300
H	0.48613200	-1.12507700	-4.36536500
C	-3.17077600	0.87606100	-1.22739100
H	-3.35466000	0.87060200	-2.30120900
C	-4.22111400	1.10805800	-0.34217700
H	-3.97301100	1.13768000	0.72068000
N	0.67040000	1.97342100	1.46971700
C	0.52187000	2.24157700	2.75803700
S	0.69800900	3.91918600	3.16121300
C	0.93886200	4.28140200	1.45953100
C	1.13225900	5.50724600	0.83538700
H	1.18450800	6.43023700	1.40838400
C	1.23937400	5.53060000	-0.55021300
H	1.38081900	6.49488400	-1.03228400
C	1.15656300	4.36454000	-1.33420500
C	0.98686200	3.14515900	-0.68802300
H	0.90124800	2.21696000	-1.24722600
C	0.87871800	3.10163000	0.69870500

Coordinates of IrS-1f in Triplet State

N	0.83164400	-1.96645200	0.56302700
C	2.09634400	-2.35496900	0.56569000
S	2.34228300	-4.01210700	0.09646400
C	0.60807200	-4.18318200	-0.11988800
C	-0.12716500	-5.29419500	-0.51089500
H	0.36097500	-6.23645300	-0.75110300
C	-1.51005800	-5.17984600	-0.58409100
H	-2.07547400	-6.05580700	-0.89149900
C	-2.18607400	-3.99064100	-0.25696700
C	-1.42872200	-2.88932300	0.13808600
H	-1.89606400	-1.95455300	0.43902900
C	-0.04013400	-2.97740600	0.19337200
C	3.09451600	-1.37860000	0.89627100
C	2.55138800	-0.08671500	1.10908500
C	3.44620000	0.94107800	1.41961800
H	3.07896700	1.95337700	1.59269800
C	4.81349000	0.69032100	1.49543200
H	5.49188200	1.50735000	1.73849400
C	5.33347500	-0.58566600	1.26008800
H	6.40601500	-0.75916400	1.30990200
C	4.47043700	-1.62534000	0.95940000
H	4.85805700	-2.62834100	0.77005700
C	-3.70511600	-3.85900800	-0.37193500
C	-4.02101900	-3.01806900	-1.61587100
H	-5.10781900	-2.89300100	-1.73293100
H	-3.62796100	-3.49420200	-2.52540200
H	-3.57670300	-2.01595900	-1.53598700
C	-4.39710200	-5.21364400	-0.51291900
H	-5.48544600	-5.07081400	-0.53353300
H	-4.16400500	-5.87841900	0.33014300
H	-4.12291300	-5.72699000	-1.44426400
C	-4.28167800	-3.16323200	0.86636300
H	-5.37425000	-3.08451500	0.77508600
H	-3.88952800	-2.14521000	0.99757800
H	-4.05560500	-3.72790400	1.78082900
C	-2.07910800	0.37666600	-0.63171100
O	-1.68646400	0.37382100	0.53952000
N	0.18360600	0.08816500	-1.32859100
C	0.81434400	-0.37666600	-2.40319000
C	-0.12596400	-0.68385100	-3.42880600
H	0.09853700	-1.06504400	-4.41685600
C	-3.44951600	0.56667600	-1.03417000
H	-3.66842200	0.71331000	-2.08983400
C	-4.42864800	0.48885300	-0.10243600
H	-4.10715500	0.33383500	0.93124600
N	0.47664400	2.07037900	1.42442500
C	0.46368100	2.31113400	2.72472400
S	0.39517500	3.99969800	3.13765400
C	0.33460800	4.39561800	1.42679600
C	0.21803600	5.63298400	0.80666900
H	0.18099600	6.55203900	1.38757400
C	0.13146600	5.67373600	-0.58088500
H	0.02425500	6.64582100	-1.05608100
C	0.16766100	4.51023300	-1.36803100
C	0.31719000	3.28310500	-0.72785000
H	0.35994900	2.35676300	-1.29440300
C	0.38979300	3.22067300	0.65982600

C	0.20870300	1.13928700	3.62605800	C	0.49080600	1.17933000	3.61168400
C	0.11564100	-0.09997900	2.93240800	C	0.53372100	-0.05892100	2.92150300
C	-0.22846200	-1.24229900	3.66323100	C	0.53204100	-1.22339300	3.68951400
H	-0.31820500	-2.20571200	3.16239900	H	0.57053600	-2.19936300	3.20515400
C	-0.46256000	-1.15335800	5.02953500	C	0.48297400	-1.15568000	5.07996300
H	-0.73037200	-2.04707300	5.59032600	H	0.48280400	-2.07735400	5.66051800
C	-0.35599900	0.07153300	5.69842500	C	0.44100200	0.07261300	5.74343500
H	-0.54100000	0.12263400	6.76896200	H	0.40648200	0.10788400	6.82983700
C	-0.02205700	1.22291300	4.99937400	C	0.44291700	1.24621500	5.00804000
H	0.04894900	2.17855900	5.51908900	H	0.40323300	2.21181500	5.51419800
C	1.18835600	4.38283300	-2.86032900	C	0.02078500	4.52587500	-2.88815000
C	1.50841000	5.76896600	-3.41537900	C	-0.15731300	5.93914500	-3.43767200
H	1.54576500	5.72768100	-4.51150100	H	-0.26302300	5.90164000	-4.52977500
H	2.48417700	6.13503600	-3.06630500	H	0.70891200	6.57660100	-3.21375200
H	0.74285600	6.50905400	-3.14537900	H	-1.05760100	6.42437200	-3.03620400
C	2.24465300	3.39979300	-3.37757000	C	1.26603200	3.90919200	-3.53526100
H	2.29955500	3.45105900	-4.47333000	H	1.18092400	3.94344600	-4.63028500
H	2.00864200	2.35873200	-3.11565400	H	1.40256300	2.85632000	-3.25068700
H	3.24152600	3.63364500	-2.97783000	H	2.17404000	4.45648300	-3.24816700
C	-0.19265900	3.95391900	-3.37456600	C	-1.21331100	3.70007100	-3.27795600
H	-0.20710300	3.95903400	-4.47330700	H	-1.35300400	3.71788200	-4.36794700
H	-0.97693800	4.63588200	-3.01898100	H	-2.12224000	4.10364000	-2.80983400
H	-0.45313200	2.93869400	-3.03931100	H	-1.11440300	2.64663100	-2.97438000
C	-5.60458500	1.22919700	-0.68770400	C	-5.85433000	0.53368400	-0.32373200
C	-6.55788600	1.34490700	0.34768300	C	-6.70503600	0.30003400	0.76859300
C	-6.08194400	1.19200300	-2.01683400	C	-6.42047300	0.76841500	-1.58852600
C	-7.91242200	1.41802500	0.07203800	C	-8.08246800	0.29639300	0.60413400
H	-6.20582300	1.36778400	1.37985100	H	-6.26742500	0.11620400	1.75019500
C	-7.43715500	1.26873100	-2.28784600	C	-7.79469600	0.76929100	-1.75008800
H	-5.37689200	1.10292100	-2.84315600	H	-5.77935900	0.96008800	-2.44782200
C	-8.36273300	1.38147000	-1.24827000	C	-8.62755800	0.53132800	-0.65489300
H	-8.62733800	1.50488400	0.88844600	H	-8.73240800	0.11130100	1.45638100
H	-7.78183200	1.24084900	-3.32025900	H	-8.22684300	0.95451200	-2.73116100
H	-9.42708200	1.43973800	-1.46697700	H	-9.70770700	0.53107400	-0.78701800
C	-1.03297400	-0.31823100	-2.97913200	C	-1.34705800	-0.36115600	-2.92306300
H	-1.99330800	-0.33544600	-3.47751700	H	-2.33856700	-0.43700800	-3.35018300
N	-0.90585500	0.15368100	-1.71127000	N	-1.14659800	0.11758500	-1.64924600
C	2.51275900	-0.75364900	-2.24303200	C	2.26385800	-0.51448300	-2.44926500
C	3.40601500	0.18651600	-1.70721300	C	3.10377100	0.49834800	-1.96034500
C	3.02059200	-1.96447200	-2.71666500	C	2.83414500	-1.68672300	-2.94635300
C	4.75692900	-0.08597700	-1.63959700	C	4.47200100	0.33268200	-1.96747000
H	3.03005000	1.14641200	-1.35015400	H	2.67260300	1.41955300	-1.56726100
C	4.37669400	-2.26093700	-2.63204900	C	4.21042600	-1.87938800	-2.92110900
H	2.33474100	-2.70622500	-3.12810200	H	2.18883600	-2.48381500	-3.31797900
C	5.25099400	-1.32144900	-2.07866900	C	5.03561200	-0.86839900	-2.41899200
H	5.46351200	0.63279000	-1.22930800	H	5.13975000	1.10213700	-1.58681100
H	4.73799900	-3.22064000	-2.99213700	H	4.62388200	-2.81642000	-3.28406500
O	6.57432000	-1.51361400	-1.90788300	O	6.37373300	-0.95572900	-2.31198700
C	7.11715800	-2.75772600	-2.28813000	C	6.99599500	-2.16320200	-2.69076100
H	8.18288600	-2.71394100	-2.05383200	H	8.06382300	-2.02888900	-2.50561800
H	6.65402700	-3.58268900	-1.72487200	H	6.62494700	-3.00674000	-2.08928600
H	6.99059600	-2.94056700	-3.36475600	H	6.83863800	-2.38049200	-3.75681300
Ir	0.44697000	-0.01111400	0.97661600	Ir	0.56743300	0.06644800	0.92031900

Coordinates of RhS

Rh	-0.03142000	-0.71901700	-0.00629500
N	-2.03676300	-0.78378400	-0.54657700
C	-2.30323600	-1.66307500	-1.49021500
S	-3.92207900	-1.56060500	-2.13017900
C	-4.22883500	-0.19359500	-1.06819800
C	-5.35784200	0.60764300	-0.94888300
H	-6.25295600	0.41186700	-1.53539400
C	-5.31629500	1.68184800	-0.06577400
H	-6.20186600	2.30760600	0.01232700
C	-4.18044000	1.97434200	0.71160700
C	-3.06805000	1.14504600	0.59406000
H	-2.16180400	1.32088800	1.16857900
C	-3.08924800	0.07311300	-0.29085800
C	-1.22892700	-2.51259200	-1.93559500
C	0.01541300	-2.21041200	-1.33592200
C	1.13651400	-2.93325900	-1.72383700
H	2.11042600	-2.72421800	-1.28412800
C	1.01664600	-3.93633000	-2.68656300
H	1.90088200	-4.50039400	-2.97983300
C	-0.21389500	-4.23246000	-3.27314900
H	-0.28876400	-5.02007600	-4.01926900
C	-1.34127100	-3.51664900	-2.90273800
H	-2.30703000	-3.73496500	-3.36038900
C	-4.09292800	3.18041200	1.64440400
C	-3.03439700	4.14552800	1.09331500
H	-2.92795600	5.01536300	1.75655700
H	-3.32030200	4.51718700	0.09853400
H	-2.04875600	3.66328300	1.00730000
C	-5.41898600	3.92861600	1.75550900
H	-5.30858500	4.77580800	2.44467900
H	-6.21758400	3.28557700	2.15012700
H	-5.74771000	4.33533900	0.78922000
C	-3.67878700	2.72343200	3.04810800
H	-3.64136900	3.58619300	3.72745600
H	-2.68632400	2.25180200	3.05870900
H	-4.39800200	2.00150900	3.45731700
N	1.97006900	-0.87502800	0.54306100
C	2.18318900	-1.75851500	1.49810400
S	3.79913700	-1.73500000	2.14868700
C	4.18029000	-0.39121400	1.08469600
C	5.35293500	0.35320100	0.97811400
H	6.23146400	0.10976000	1.57178400
C	5.36737700	1.42910600	0.10486000
H	6.28188400	2.01586600	0.02988200
C	4.24847700	1.78750300	-0.67886000
C	3.10169800	1.00897500	-0.58778300
H	2.21009400	1.21938200	-1.17172000
C	3.06531000	-0.07100600	0.29678400
C	1.06170200	-2.53513600	1.95999400
C	-0.16705000	-2.16059000	1.36976700
C	-1.33132300	-2.78889500	1.79416900
H	-2.29459700	-2.51702000	1.36513100
C	-1.26767300	-3.77536800	2.77924800
H	-2.18547100	-4.26423800	3.10269000
C	-0.05099900	-4.14935800	3.35046400
H	-0.02021300	-4.92444100	4.11260500
C	1.11841700	-3.52467000	2.94680400
H	2.07332400	-3.80161300	3.39550900
C	4.32299200	3.00466000	-1.59551200
C	5.35842000	2.74077600	-2.69477200
H	5.42388200	3.60320900	-3.37245200
H	5.08454100	1.85905000	-3.28981800
H	6.35942500	2.56740100	-2.27872200
C	2.97644400	3.30552200	-2.25303300
H	3.05979200	4.21292600	-2.86532900
H	2.19002200	3.47854300	-1.50111500
H	2.64681000	2.49088500	-2.91328100
C	4.73634300	4.23825200	-0.78463900
H	4.79520500	5.11855700	-1.43902300
H	5.71713400	4.11926000	-0.30774800
H	4.00191600	4.45754800	0.00466600
C	0.89661800	1.82824600	1.93738700
N	0.14856000	1.06829800	1.48280400
C	-0.79567600	1.73184800	-2.13883500
N	-0.10276300	0.99361400	-1.57499700
C	1.88131200	2.74923600	2.46619500
H	2.73412500	2.79113000	1.77378900
H	1.45789500	3.75327500	2.57626700
H	2.23796600	2.40468800	3.44307700
C	-1.70845700	2.63134100	-2.81248700
H	-1.25331800	3.61839600	-2.94802700
H	-1.98448100	2.22829600	-3.79297700
H	-2.61714500	2.73697600	-2.20547500

Coordinates of RhS-L

Rh	0.02705900	-0.41186500	-0.09139800
N	1.88073400	-0.45242700	0.83136200
C	1.90633000	-1.17022700	1.93482800
S	3.45907900	-1.23372700	2.71435600
C	4.10091600	-0.18181300	1.46157000
C	5.37791700	0.34285200	1.31284400
H	6.17202500	0.10670700	2.01766800
C	5.61773700	1.19379900	0.24113200
H	6.61746300	1.60720200	0.13602800
C	4.62242600	1.53577500	-0.69403800
C	3.35343800	0.98170400	-0.53902900
H	2.54645700	1.20711900	-1.23789000
C	3.09375400	0.13439800	0.53389000
C	0.66942700	-1.77386000	2.35896400
C	-0.42311700	-1.50975200	1.50452000
C	-1.68530600	-1.98421500	1.82844100
H	-2.54433800	-1.79268500	1.18861500
C	-1.85223300	-2.71747200	3.00561000
H	-2.84410900	-3.08756500	3.25975600
C	-0.77686100	-2.98848000	3.84966200
H	-0.92869200	-3.56644900	4.75800800
C	0.48617300	-2.51359800	3.53111600
H	1.33134400	-2.70877300	4.19200900
C	4.87363700	2.50952800	-1.84311200
C	3.96496300	3.73213100	-1.65429600
H	4.12154300	4.45469800	-2.46703000
H	4.18539100	4.24007200	-0.70434300
H	2.90034000	3.45753900	-1.65449900
C	6.32089400	2.99402500	-1.89092900
H	6.45017400	3.68149800	-2.73661900
H	7.02640900	2.16399700	-2.03246500
H	6.60483800	3.54019600	-0.98081700
C	4.55192100	1.82974900	-3.17865800
H	4.73363500	2.52568700	-4.00889200
H	3.50438000	1.50784200	-3.24292700
H	5.18546500	0.94615000	-3.33308400
N	-1.84397800	-0.65410500	-0.97109400
C	-1.94580800	-1.76441200	-1.67824900
S	-3.53340700	-2.07506000	-2.30639200
C	-4.09928000	-0.60106300	-1.54154200
C	-5.37839400	-0.05045600	-1.52147400
H	-6.20627800	-0.52878500	-2.04011200
C	-5.57075000	1.12411700	-0.81456600
H	-6.57255900	1.55033600	-0.79233700
C	-4.52628500	1.77617400	-0.12405000
C	-3.25966500	1.20939700	-0.15439400
H	-2.41758400	1.65611300	0.36508800
C	-3.04440600	0.02736400	-0.86475500
C	-0.77645300	-2.60082700	-1.79706900
C	0.32974900	-2.14453100	-1.04429400
C	1.51358600	-2.87206600	-1.09042100
H	2.38777000	-2.55500200	-0.52247600
C	1.59393600	-4.02216800	-1.87911100
H	2.52727200	-4.58203500	-1.91109400
C	0.49943300	-4.46496800	-2.61848800
H	0.57649800	-5.36465600	-3.22445500
C	-0.69155000	-3.75328500	-2.58105600
H	-1.55007000	-4.08668300	-3.16525100
C	-4.81906700	3.06042500	0.64507400
C	-5.78958900	2.74539200	1.78956100
H	-6.01890400	3.65728300	2.35819600
H	-5.35739900	2.01075300	2.48296900
H	-6.73970500	2.33842200	1.41984000
C	-3.55145500	3.67226100	1.24119500
H	-3.80447400	4.60006400	1.77085300
H	-2.82201200	3.92526500	0.45635600
H	-3.06928500	2.99917600	1.96555200
C	-5.44718000	4.09604100	-0.29450000
H	-5.65039800	5.02678800	0.25265600
H	-6.39950600	3.75214400	-0.71686600
H	-4.77508300	4.33184900	-1.13101300
C	0.16820000	2.79192400	0.98508300
N	-0.08704000	1.70512500	0.67715900
C	0.49476100	4.14989100	1.36543600
H	0.07736900	4.85748200	0.64053700
H	0.08170800	4.37447400	2.35508700
H	1.58310400	4.27559400	1.39710900

Coordinates of RhS-0L

Rh	-0.01294000	-0.18156000	0.00018700
N	-1.79707100	-0.33775200	-1.03862800
C	-1.76933900	-1.24865900	-1.99172200
S	-3.26267100	-1.43242900	-2.85309700
C	-3.97568400	-0.19035200	-1.83490000
C	-5.26605800	0.32379300	-1.83505400
H	-6.01313400	-0.02188600	-2.54592100
C	-5.58385600	1.29433400	-0.89364100
H	-6.59607200	1.69010900	-0.89733800
C	-4.65728900	1.76988800	0.05418500
C	-3.36669500	1.24602900	0.03139000
H	-2.61005900	1.57400700	0.74545100
C	-3.02869300	0.27757700	-0.90911800
C	-0.53870900	-1.98651100	-2.16242500
C	0.47396000	-1.64743300	-1.23688200
C	1.69907300	-2.29475300	-1.27127500
H	2.48367700	-2.05747000	-0.55527300
C	1.92340500	-3.26557900	-2.25208600
H	2.88678800	-3.77118800	-2.28658000
C	0.93601600	-3.59531100	-3.17660900
H	1.12740900	-4.35490500	-3.93074000
C	-0.29807600	-2.95851000	-3.13481300
H	-1.07304900	-3.21347200	-3.85809900
C	-5.01805300	2.82357800	1.09959800
C	-4.10469300	4.04324400	0.92535500
H	-4.36054200	4.81379700	1.66526900
H	-4.21854100	4.48336800	-0.07461700
H	-3.04452700	3.79310000	1.06556500
C	-6.46635100	3.29166700	0.97666700
H	-6.67223100	4.04728300	1.74548800
H	-7.18012600	2.47003300	1.12668500
H	-6.66744100	3.75464400	0.00074700
C	-4.82303400	2.22775100	2.49913700
H	-5.08292000	2.97111200	3.26506900
H	-3.78458200	1.91800200	2.67896400
H	-5.46739600	1.35048500	2.64733000

N	1.76967200	-0.19048600	1.06079100
C	1.72249400	-0.93730400	2.14715700
S	3.20597700	-0.99628700	3.04076000
C	3.94013100	0.05973700	1.84627300
C	5.23952600	0.55663900	1.78049000
H	5.97503500	0.32340500	2.54692100
C	5.57243900	1.35812500	0.70278900
H	6.58875700	1.74474500	0.64936000
C	4.65820200	1.68662700	-0.32414700
C	3.36592200	1.18674800	-0.23593400
H	2.61631500	1.39847400	-0.99691800
C	3.00953700	0.38232100	0.84878600
C	0.48401600	-1.62762100	2.42297200
C	-0.52035300	-1.43266700	1.44901900
C	-1.75110300	-2.05726000	1.57832800
H	-2.52994600	-1.93013400	0.82921200
C	-1.99003500	-2.85674000	2.70006900
H	-2.95877200	-3.34123500	2.80862900
C	-1.01027800	-3.04213300	3.67158700
H	-1.21401300	-3.66889500	4.53642100
C	0.22926900	-2.42989300	3.53658400
H	0.99760100	-2.57280600	4.29708800
C	5.12012200	2.56387500	-1.48345800
C	6.27709600	1.86849800	-2.21083300
H	6.61467000	2.48521400	-3.05479300
H	5.96344200	0.89315000	-2.60765100
H	7.14273000	1.70367300	-1.55680400
C	4.00466000	2.81700400	-2.49521300
H	4.38394600	3.44852600	-3.30855100
H	3.15176800	3.34348000	-2.04366700
H	3.64104000	1.88388900	-2.94895100
C	5.58856200	3.91847900	-0.93772200
H	5.92270000	4.56077100	-1.76383400
H	6.42838500	3.82040500	-0.23807400
H	4.77353400	4.43651000	-0.41386600

Coordinates of IrS

N	-1.84982400	-0.56103000	-0.95861600
C	-1.86634700	-1.48414400	-1.90607900
S	-3.39829600	-1.66468400	-2.70940800
C	-4.05693800	-0.39604800	-1.69068300
C	-5.33775000	0.13951800	-1.66891900
H	-6.10814500	-0.21696700	-2.34925400
C	-5.61728700	1.14531200	-0.75151900
H	-6.62230900	1.55954000	-0.73874200
C	-4.65165500	1.62814900	0.14836000
C	-3.37220600	1.07893900	0.10595300
H	-2.59086600	1.41210500	0.78252200
C	-3.07139100	0.07562700	-0.80777200
C	-0.65664600	-2.21503400	-2.16513100
C	0.41827500	-1.82558600	-1.33045700
C	1.64174400	-2.46830300	-1.52182800
H	2.50444500	-2.20422500	-0.90967100
C	1.78212800	-3.45119700	-2.49959700
H	2.74739200	-3.93850200	-2.63096500
C	0.70850200	-3.82518300	-3.31019600
H	0.83480000	-4.59780500	-4.06516800
C	-0.51760500	-3.20408500	-3.14511300
H	-1.36632200	-3.48374100	-3.77081700
C	-4.95061000	2.71412400	1.18004200
C	-3.98972400	3.89201900	0.97645900
H	-4.18868100	4.67797600	1.71865800
H	-4.11086300	4.33223600	-0.02272500
H	-2.93715800	3.59178800	1.08079500
C	-6.37847600	3.24379800	1.07219300
H	-6.54368600	4.01995600	1.83084100
H	-7.12329500	2.45481700	1.24340400
H	-6.57301500	3.69645400	0.09014400
C	-4.75811100	2.13112000	2.58526900
H	-4.94395400	2.90035000	3.34825100
H	-3.73982900	1.74370400	2.73358200
H	-5.45337400	1.30020200	2.76501700
N	1.83871200	-0.55070900	0.95960200
C	1.85401000	-1.46527800	1.91627000
S	3.38876500	-1.64766000	2.71054000
C	4.04838700	-0.39564400	1.67503800
C	5.33706500	0.12969000	1.64262800
H	6.10682900	-0.22617000	2.32396400
C	5.61558000	1.11914600	0.71530500
H	6.62366300	1.53032500	0.68964300
C	4.64789400	1.60207200	-0.19050000
C	3.36655000	1.06828300	-0.13764000
H	2.58028500	1.39825700	-0.80820400
C	3.06430700	0.07424700	0.79423800
C	0.64263800	-2.18967600	2.18745900
C	-0.43261600	-1.80940100	1.34975100
C	-1.65756500	-2.44588900	1.55098200
H	-2.52012600	-2.18705500	0.93636500
C	-1.79916900	-3.41570100	2.54167500
H	-2.76540400	-3.89889400	2.68079800
C	-0.72504900	-3.78152900	3.35511800
H	-0.85180400	-4.54422200	4.12006700
C	0.50249300	-3.16556300	3.18024400
H	1.35110800	-3.43955600	3.80859200
C	5.03241600	2.68778400	-1.19206400
C	6.18996700	2.19261700	-2.06719200
H	6.46667000	2.96332900	-2.79987400
H	5.90768600	1.28473000	-2.61773500
H	7.08749900	1.96220700	-1.47982300
C	3.86945300	3.05235300	-2.11315000
H	4.18811000	3.83026400	-2.81947100
H	3.01076800	3.44631300	-1.54857500
H	3.53867900	2.18254800	-2.70051600
C	5.45889900	3.94934000	-0.43308900
H	5.73503900	4.74433900	-1.13961100
H	6.32631800	3.76727300	0.21403300
H	4.64157900	4.32331700	0.19913700
C	-0.45498800	1.86890500	2.43731400
N	-0.39322000	1.17221700	1.51544000
C	0.36003400	1.84960500	-2.45872200
N	0.36108900	1.16168100	-1.52828600
C	-0.55676100	2.74601100	3.58507800
H	0.43121500	2.90493400	4.03066500
H	-0.96953900	3.71563200	3.28432400
H	-1.21637900	2.30210000	4.33903200
C	0.37154400	2.71259800	-3.62153400
H	0.72691100	3.71231000	-3.34785300
H	1.03793100	2.30101600	-4.38771300
H	-0.63716400	2.79789900	-4.03998500
Ir	-0.00643800	-0.37732900	0.00120300

Coordinates of IrS-L

N	-1.72290400	-0.92257400	-0.86188400
C	-1.62601700	-2.12532700	-1.41039200
S	-3.05987800	-2.66776800	-2.21918200
C	-3.86096200	-1.17494200	-1.75969100
C	-5.16227200	-0.75985100	-2.00990600
H	-5.84812500	-1.37505700	-2.58809700
C	-5.57446300	0.46394200	-1.49649600
H	-6.59442700	0.78163700	-1.69718100
C	-4.72520300	1.27896500	-0.72780000
C	-3.42315500	0.84378000	-0.49386300
H	-2.73276400	1.42508900	0.11020400
C	-2.98492400	-0.36612200	-1.01787500
C	-0.38870100	-2.84545000	-1.24634000
C	0.57173100	-2.13938200	-0.47855300
C	1.79119700	-2.77380300	-0.23776300
H	2.56045200	-2.28025500	0.35676000
C	2.04793300	-4.04255000	-0.75719800
H	3.00859800	-4.51654400	-0.56147600
C	1.09385100	-4.71454700	-1.52020100
H	1.30742700	-5.70413700	-1.91739800
C	-0.13295500	-4.11572400	-1.76671500
H	-0.88775300	-4.63291700	-2.36020600
C	-5.16539200	2.61956000	-0.14301000
C	-4.28643200	3.73178800	-0.72864400
H	-4.57644500	4.70690000	-0.31244000
H	-4.38942200	3.78429900	-1.82081600
H	-3.22191500	3.56990700	-0.50505300
C	-6.62217100	2.94670300	-0.46283200
H	-6.88690900	3.91703600	-0.02297700
H	-7.31190400	2.19970200	-0.04697400
H	-6.79939800	3.01875100	-1.54455800
C	-5.00454700	2.58712500	1.38157500
H	-5.32285100	3.54475800	1.81683500
H	-3.96380000	2.40890700	1.68866300
H	-5.62001300	1.79354300	1.82603300
N	1.82245300	0.08961900	1.01655500
C	1.83353900	-0.21003000	2.30319400
S	3.36207500	0.05395400	3.08129500
C	4.02119100	0.60519000	1.54936500
C	5.30740300	1.04115400	1.24229300
H	6.08397000	1.09199300	2.00219700
C	5.57428500	1.40751300	-0.06596800
H	6.58132000	1.74496800	-0.30630800
C	4.60323500	1.35513300	-1.09084400
C	3.32518100	0.92086400	-0.76360300
H	2.53679200	0.84678300	-1.51164600
C	3.03893000	0.55074500	0.55173200
C	0.61371400	-0.72966300	2.86158700
C	-0.42532800	-0.86941100	1.91167600
C	-1.65843300	-1.36576500	2.31864200
H	-2.47110100	-1.49087600	1.60498100
C	-1.85257500	-1.70116200	3.65831900
H	-2.82119200	-2.08617900	3.97309100
C	-0.83043900	-1.55690500	4.59760400
H	-1.00301300	-1.82653800	5.63676500
C	0.40537900	-1.07183200	4.20149300
H	1.20950200	-0.95545500	4.92881600
C	4.98779200	1.76798100	-2.50787400
C	6.11887400	0.86138500	-3.00857700
H	6.40192600	1.14248100	-4.03216400
H	5.80364100	-0.19092800	-3.02046100
H	7.01947700	0.93516700	-2.38591800
C	3.81665300	1.64918100	-3.48038500
H	4.13771200	1.96375900	-4.48157200
H	2.97416000	2.29290400	-3.18916500
H	3.45365500	0.61492000	-3.56267200
C	5.45839800	3.22732800	-2.49988200
H	5.74072700	3.53774500	-3.51514100
H	6.33236300	3.37907700	-1.85386000
H	4.66104100	3.89752400	-2.14976800
C	-0.53652000	2.92848800	0.76100000
N	-0.43624600	1.80912600	0.48159200
C	-0.67949400	4.32749800	1.10426700
H	0.24107000	4.70050600	1.56633200
H	-0.88778300	4.91740200	0.20454500
H	-1.50782700	4.45913100	1.80997600
Ir	0.02985400	-0.28492600	0.06497400

Coordinates of IrS-0L

N	-1.75990700	1.12547900	-0.04609100
C	-1.71429000	2.31092700	-0.63153800
S	-3.18445800	3.21651300	-0.53548300
C	-3.91737600	1.89176900	0.35540800
C	-5.20589100	1.77232500	0.85827300
H	-5.93554100	2.57060400	0.74329400
C	-5.54606500	0.59554000	1.51214800
H	-6.55673400	0.50647700	1.90143800
C	-4.64212100	-0.47106600	1.68088500
C	-3.35195500	-0.32624800	1.17499300
H	-2.61216400	-1.12202800	1.27362800
C	-2.99319000	0.84737200	0.52013900
C	-0.47969100	2.66443300	-1.29020200
C	0.50688800	1.65001200	-1.22902600
C	1.72983900	1.84959800	-1.86061700
H	2.49784800	1.07795700	-1.84385800
C	1.97374100	3.05713200	-2.51869800
H	2.93520900	3.21148500	-3.00525000
C	1.00819700	4.06017300	-2.56274100
H	1.21514600	4.99331900	-3.08116100
C	-0.22310200	3.86660100	-1.94969500
H	-0.98289800	4.64772300	-1.98577000
C	-5.03035500	-1.77227000	2.37931500
C	-4.09930800	-2.00551700	3.57550700
H	-4.37424900	-2.93580400	4.09085000
H	-4.17290900	-1.18340100	4.30033600
H	-3.04701300	-2.09618900	3.27464000
C	-6.46846700	-1.74904500	2.89293200
H	-6.69538500	-2.70254900	3.38669200
H	-7.19569400	-1.62245900	2.07922300
H	-6.62930500	-0.95168800	3.63163200
C	-4.89417700	-2.93130900	1.38408000
H	-5.16436800	-3.87904300	1.86944100
H	-3.86904600	-3.03444700	1.00308800
H	-5.56092400	-2.79028900	0.52248100
N	1.74520600	-1.01724200	-0.45875800
C	1.69587400	-1.91766300	-1.42752900
S	3.17262300	-2.78200700	-1.67486000
C	3.90908800	-1.85465100	-0.37899500
C	5.20656800	-1.91216500	0.12353000
H	5.93835800	-2.60973400	-0.27753000
C	5.54077200	-1.05082000	1.15375200
H	6.55527600	-1.09536600	1.54627200
C	4.62787300	-0.12566600	1.70838900
C	3.33885500	-0.08477600	1.19332600
H	2.59377100	0.61154900	1.57674000
C	2.98343700	-0.94965000	0.15632800
C	0.45036400	-2.03013600	-2.14887800
C	-0.53944000	-1.11425300	-1.71475400
C	-1.77735500	-1.09724700	-2.34823700
H	-2.54750200	-0.38925500	-2.04702400
C	-2.03297400	-2.00203100	-3.38053500
H	-3.00566400	-1.98921800	-3.86892400
C	-1.06386800	-2.91262600	-3.79536000
H	-1.27955900	-3.60839900	-4.60256300
C	0.18239200	-2.92865600	-3.18156400
H	0.94398600	-3.63839300	-3.50551300
C	5.08675500	0.78870200	2.83942400
C	6.23992900	1.66792100	2.33980300
H	6.57547400	2.33850800	3.14249900
H	5.92509000	2.28814300	1.48927800
H	7.10820200	1.07662500	2.02178400
C	3.96651700	1.70215000	3.33130600
H	4.33918800	2.33403000	4.14747600
H	3.11417200	1.13064700	3.72530700
H	3.60412400	2.37346200	2.53973800
C	5.56186600	-0.06570700	4.02108800
H	5.88773800	0.58228300	4.84594100
H	6.40950500	-0.71029800	3.75639500
H	4.75347100	-0.70915200	4.39434100
Ir	-0.00845900	0.03995100	-0.19271500
