# Supplementary material for: Catalytic asymmetric synthesis of a nitrogen heterocycle through stereocontrolled direct photoreaction from electronically excited state
Source: Nat Commun. 2017 Dec 21;8:2245. doi: 10.1038/s41467-017-02148-1 (PMC5740077; doi:10.1038/s41467-017-02148-1)
Supplement: Supplementary file 2 — Peer Review File [file 41467_2017_2148_MOESM2_ESM.pdf]

Editorial Note: This manuscript has been previously reviewed at another journal that is not operating a transparent peer review scheme. The manuscript was considered suitable for publication without further review at *Nature Communications.*
